# Minutellins E-I and daldinone L, new secondary metabolites from different species of the Hypoxylaceae (Xylariales, Ascomycota)

**DOI:** 10.1080/21501203.2025.2512962

**Published:** 2025-06-18

**Authors:** Christopher Lambert, Mohammad Javad Pourmoghaddam, Esteban Charria-Girón, Frank Surup, Seyed Akbar Khodaparast, Hermann Voglmayr, Irmgard Krisai-Greilhuber, Marc Stadler

**Affiliations:** a Microbial Drugs, Helmholtz-Centre for Infection Research GmbH, Braunschweig, Germany; b Cell Biology, Helmholtz-Centre for Infection Research GmbH, Braunschweig, Germany; c Institute of Microbiology, Technical University Carolo-Wilhelmina Braunschweig, Braunschweig, Germany; d German Centre for Infection Research (DZIF), Partner Site Hannover-Braunschweig, Braunschweig, Germany; e Department of Plant Protection, Faculty of Agricultural Sciences, University of Guilan, Rasht, Iran; f Department of Botany and Biodiversity Research, University of Vienna, Wien, Austria

**Keywords:** Azaphilones, molecular phylogeny, taxonomy, secondary metabolites, structure elucidation

## Abstract

Fungi of the family Hypoxylaceae (Xylariales, Ascomycota) are ubiquitously distributed and fulfil important ecological roles as saprobes, pathogens, and endophytes. Members of this family tend to store large amounts of secondary metabolites in their carbonaceous stromatal tissue commonly formed on colonised wood. This feature is of both taxonomic and chemical value by serving as chemotaxonomic markers and sources of potential new and bioactive compounds. Despite tremendous progress in the characterisation of Hypoxylaceae both in terms of genomics and secondary metabolomics, many unknown metabolites remain to be identified or elucidated. Here, we report on the polyphasic, i.e. morphological, chemical, and genetical analysis of *Hypoxylon* and *Annulohypoxylon* spp. collected from the Iranian shore of the Caspian Sea and a chemotaxonomic study of the constituents of a concurrently studied specimen of *A. michelianum*. A new daldinone derivative and five new minutellin-type azaphilones from stromatal tissues of *A. substygium* and *H. lateripigmentum*, respectively, were identified in the course of this study by high-performance liquid chromatography/mass spectrometry coupling (HPLC-MS) and structurally elucidated by nuclear magnetic resonance (NMR) spectroscopy. We realised that the structure of daldinone A was misassigned and in fact equals daldinone C. Finally yet importantly, the distribution of minutellin-type azaphilones throughout the Hypoxylaceae is discussed.

## 1. Introduction

Fungi of the family Hypoxylaceae (Xylariales, Ascomycota) are distributed worldwide and may occur as both, opportunistic plant pathogens and saprobes, in addition to their endophytic lifestyle (Helaly et al. 2018). They form a wide array of morphologically conspicuous, carbonised teleomorphs, along with several distinct types of anamorph structures in culture, which can occasionally also be observed in nature (Ju and Rogers 1996; Wendt et al. 2018). While teleomorph morphology has long been regarded as an important character in the systematics of the Hypoxylaceae, it became clear that this phenotypic feature alone is insufficient, as it is inconsistent with the phylogenetic evidence acquired over the years (Hsieh and Rogers 2005; Jaklitsch et al. 2016). Indeed, it has repeatedly been confirmed that the anamorph morphology first used by Ju and Rogers (1996) to segregate the “hypoxyloid” (nodulisporium-like) and “xylarioid” (geniculosporium-like anamorph) taxa is a better predictor of the phylogenetic affiliation of a given species. Still, teleomorph morphology repeatedly proves to have merit in delimiting individual species from each other. Nowadays, taxonomic assessment of members of the Hypoxylaceae involves a combinatorial analysis of both teleo- and anamorph morphology, genetic analysis, which is further complemented by chemical analysis within a chemotaxonomic framework (Wendt et al. 2018). Chemical analysis usually comprises an analysis of secondary metabolites in stromata present in almost all members of Hypoxylaceae. Together with secondary metabolites produced in culture, these chemical constituents repeatedly served as good indicators for phylogenetic affiliations (such as mitorubrins for the “red” *Hypoxylon*, Stadler et al. 2008; see also Stadler et al. 2014; Kuhnert et al. 2015, 2017a; Wendt et al. 2018; Lambert et al. 2019 for additional examples). Genetic analysis nowadays usually includes data derived from the internal transcribed spacer and large subunit ribosomal DNA (ITS and LSU rDNA), and the DNA-directed RNA polymerase II ribosomal and β-tubulin (*rpb2*, *tub2*) genes, both used in multi-locus genealogies for phylogenetic inference (Wendt et al. 2018). This polyphasic approach allowed the resurrection and segregation of the Hypoxylaceae, for example, from Xylariaceae based on phylogenetic and chemical evidence (Wendt et al. 2018). Another example comprises the recently described genus *Parahypoxylon*, which was erected to accommodate phylogenetically distinct specimens of *Hypoxylon* s. Ju and Rogers (Cedeño-Sanchez et al. 2023). In the latter study, sequences generated from an ascospore-derived isolate of a specimen collected in the Democratic Republic of Congo were shown to cluster with the aberrant *Hypoxylon papillatum* (now: *Parahypoxylon papillatum*), which typically resolved in a position basal to other Hypoxylaceae (Wendt et al. 2018; Lambert et al. 2019, 2021; Pourmoghaddam et al. 2020). State-of-the-art metabolomics were then used for the first time for Hypoxylaceae to propose an unprecedented chemical diversity of cohaerin-type azaphilone compounds in stromatal extracts, which are commonly found in the hypoxylaceous genus *Jackrogersella* (Quang et al. 2005, 2006; Surup et al. 2013). The collected specimen was described as *Parahypoxylon ruwenzoriense* sp. nov.; however, paucity of the available material prevented any in-depth chemical characterisation (Cedeño-Sanchez et al. 2023). Similar compounds were previously described to be extant in *A. michelianum* and we further noted similar compounds, besides other unidentifiable compounds, in a collection of Iranian specimens showing affiliations to *Hypoxylon* and *Annulohypoxylon* collected near the Caspian Sea. This motivated us to embark on studying the taxonomic affiliations of the Iranian specimen and to characterise these potentially new stromatal secondary metabolites.

## 2. Materials and methods

All scientific names listed throughout the manuscript follow the respective entries in Index Fungorum (http://www.indexfungorum.org) and are hence given without authorities or year or publication. Herbaria and culture collections are abbreviated as recommended by Index Herbariorum (http://sweetgum.nybg.org/science/ih).

### 2.1. Morphological observation

The fungal specimens were collected in northern Iran (Guilan and Mazandaran Provinces). For light microscopy, fresh collections, single ascospore isolations, and cultures were examined for macro- and micromorphological characteristics, according to Pourmoghaddam et al. (2020). Dried specimens were deposited in the University of Guilan Mycological Herbarium (GUM). Living cultures were deposited in the culture collection MUCL (Louvain la-Neuve, Belgium) and in the Iranian Fungal Culture Collection, Iranian Research Institute of Plant Protection, Tehran, Iran (IRAN).

### 2.2. DNA extraction, PCR, and sequencing

DNA extraction of fresh cultures and amplification of the ITS (nuc rDNA internal transcribed spacer region containing ITS1-5.8S-ITS2), LSU (5’ 1,200 bp of the large subunit nuc 28S rDNA), *rpb2* (partial second largest subunit of the DNA-directed RNA polymerase II), and *tub2* (partial β-tubulin) loci were carried out as described by Wendt et al. (2018).

### 2.3. Phylogenetic analyses

Published sequences of a single accession for each *Annulohypoxylon* and *Hypoxylon* species served as basis for the sequence matrix. Information on all used strains, their corresponding sequences and GenBank accession numbers can be found in Table 1. To reveal the phylogenetic position of the Iranian *Annulohypoxylon* and *Hypoxylon* accessions, the newly generated sequences were aligned with the GenBank sequences. All alignments were produced with the server versions of MAFFT v. 7.490 (www.ebi.ac.uk/Tools/mafft or http://mafft.cbrc.jp/alignment/server/; Katoh et al. 2019) and checked and refined using BioEdit v. 7.0.4.1 (Hall 1999).

**Table 1.** Isolation and accession numbers of sequences used in the phylogenetic analyses.

Species	Strain number	Origin	Status	GenBank accession number				References
				ITS	LSU	*rpb2*	*tub2*	
*Annulohypoxylon annulatum*	CBS 140775	Texas	ET	KY610418	KY610418	KY624263	KX376353	Kuhnert et al. (2017a; *tub2*), Wendt et al. (2018; ITS, LSU, *rpb2*)
*Annulohypoxylon fusisporum*	UADY 83	Mexico	HT	OR807998	OR807987	OR825472	OR825468	Reyes et al. (2024)
*Annulohypoxylon michelianum*	CBS 119993	Spain		KX376320	KY610423	KY624234	KX271239	Kuhnert et al. (2014; ITS, *tub2*), Wendt et al. (2018; LSU, *rpb2*)
*Annulohypoxylon moriforme*	CBS 123579	Martinique		KX376321	KY610425	KY624289	KX271261	Kuhnert et al. (2017a; ITS, *tub2*), Wendt et al. (2018; LSU, *rpb2*)
*Annulohypoxylon nitens*	MFLUCC 12-0823	Thailand		KJ934991	KJ934992	KJ934994	KJ934993	Daranagama et al. (2015)
*Annulohypoxylon stygium*	MUCL 54601	French Guiana		KY610409	KY610475	KY624292	KX271263	Wendt et al. (2018)
*Annulohypoxylon substygium*	MUCL 51708	Iran	ET	KC968915	N/A	N/A	KC977285	Kuhnert et al. (2014)
*Annulohypoxylon substygium*	STMA 14066	Argentina		KU604575	N/A	N/A	KU159526	Sir et al. (2016; ITS), Kuhnert et al. (2017a; *tub2*)
*Annulohypoxylon substygium*	**MUCL 57734**	**Iran**		**PV522280**	**PV522289**	**PV524395**	**PV524404**	**This study**
*Annulohypoxylon substygium*	**IRAN 3727C**	**Iran**		**PV522281**	**PV522290**	**PV524396**	**PV524405**	**This study**
*Annulohypoxylon substygium*	**IRAN 3728C**	**Iran**		**PV522282**	**PV522291**	**PV524397**	**PV524406**	**This study**
*Annulohypoxylon substygium*	**318**	**Iran**		**PV522283**	**PV522292**	**PV524398**	**PV524407**	**This study**
*Annulohypoxylon substygium*	**465**	**Iran**		**PV522284**	**PV522293**	**PV524399**	**PV524408**	**This study**
*Annulohypoxylon substygium*	**485**	**Iran**		**PV522285**	**PV522294**	**PV524400**	**PV524409**	**This study**
*Annulohypoxylon truncatum*	CBS 140778	Texas	ET	KY610419	KY610419	KY624277	KX376352	Kuhnert et al. (2017a; *tub2*), Wendt et al. (2018; ITS, LSU, *rpb2*)
*Biscogniauxia nummularia*	MUCL 51395	France	ET	KY610382	KY610427	KY624236	KX271241	Wendt et al. (2018)
*Daldinia andina*	CBS 114736	Ecuador	HT	AM749918	KY610430	KY624239	KC977259	Bitzer et al. (2008; ITS), Kuhnert et al. (2014; *tub2*), Wendt et al. (2018; LSU, *rpb2*)
*Daldinia bambusicola*	CBS 122872	Thailand	HT	KY610385	KY610431	KY624241	AY951688	Hsieh and Rogers (2005; *tub2*), Wendt et al. (2018; ITS, LSU, *rpb2*)
*Daldinia childiae*	CBS 122881	France	HT	KU683757	MH874773	KU684290	KU684129	U’Ren et al. (2016; ITS, *rpb2*, *tub2*), Vu et al. (2019; LSU)
*Daldinia loculatoides*	CBS 113279	UK	ET	AF176982	KY610438	KY624247	KX271246	Wendt et al. (2018)
*Daldinia petriniae*	MUCL 49214	Austria	ET	AM749937	KY610439	KY624248	KC977261	Bitzer et al. (2008; ITS), Kuhnert et al. (2014; *tub2*), Wendt et al. (2018; LSU, *rpb2*)
*Daldinia theissenii*	CBS 113044	Argentina	PT	KY610388	KY610441	KY624251	KX271247	Wendt et al. (2018)
*Daldinia vernicosa*	CBS 119316	Germany	ET	KY610395	KY610442	KY624252	KC977260	Kuhnert et al. (2014; *tub2*), Wendt et al. (2018; ITS, LSU, *rpb2*)
*Daldinia* sp.	ATCC 46302	USA		KY610389	KY610443	KY624253	KX271248	Wendt et al. (2018)
*Durotheca comedens*	YMJ 90071615	Taiwan of China	HT	EF026128	N/A	JX507793	EF025613	Ju et al. (2003)
*Durotheca crateriformis*	GMBC0205	China	HT	MH645426	MH645425	MH645427	MH049441	de Long et al. (2019)
*Durotheca guizhouensis*	GMBC0065	China	HT	MH645423	MH645421	MH645422	MH049439	de Long et al. (2019)
*Durotheca rogersii*	YMJ 92031201	Taiwan of China	HT	EF026127	N/A	JX507794	EF025612	Ju et al. (2007)
*Entonaema cinnabarinum*	CNF 2/11046	Croatia		OQ863621	OQ863622	OQ877102	OQ877113	Pošta et al. (2023)
*Entonaema cinnabarinum*	CNF 2/11047	Croatia		OQ863735	OQ864983	OQ877103	OQ877114	Pošta et al. (2023)
*Entonaema liquescens*	CNF 2/11263	USA		OQ869784	OQ865124	OQ877106	OQ877117	Pošta et al. (2023)
*Entonaema liquescens*	ENCB: RV_19274	Mexico		OR807997	OR807993	OR825474	OR825466	Reyes et al. (2024)
*Graphostroma platystomum*	CBS 270.87	France		JX658535	DQ836906	KY624296	HG934108	Zhang et al. (2006; LSU), Stadler et al. (2014; ITS), Koukol et al. (2015; *tub2*), Wendt et al. (2018; *rpb2*)
*Hypomontagnella barbarensis*	STMA 14081	Argentina	HT	MK131720	MK131718	MK135891	MK135893	Lambert et al. (2019)
*Hypomontagnella monticulosa*	MUCL 54604	French Guiana	ET	KY610404	KY610487	KY624305	KX271273	Wendt et al. (2018)
*Hypoxylon aveirense*	MUM 19.40	Portugal	HT	MN053021	ON954142	OP251028	MN066636	Vicente et al. (2021; ITS, *tub2*), Cedeño-Sanchez et al. (2023; LSU, *rpb2*)
*Hypoxylon baruense*	UCH9545	Panama	HT	MN056428	ON954143	PP732079	MK908142	Cedeño-Sanchez et al. (2020; ITS, *tub2*), Cedeño-Sanchez et al. (2023; LSU); Cedeño-Sanchez et al. (2025; *rpb2*)
*Hypoxylon canariense*	MUCL 47224	Spain	PT	ON792787	ON954140	OP251029	ON813073	Cedeño-Sanchez et al. (2023)
*Hypoxylon carneum*	MUCL 54177	France		KY610400	KY610480	KY624297	KX271270	Wendt et al. (2018)
*Hypoxylon cercidicola*	CBS 119009	France		KC968908	KY610444	KY624254	KC977263	Kuhnert et al. (2014; ITS, *tub2*), Wendt et al. (2018; LSU, *rpb2*)
*Hypoxylon chionostomum*	STMA 14060	Argentina		KU604563	ON954144	OP251030	ON813072	Sir et al. (2016; ITS), Cedeño-Sanchez et al. (2023; LSU, *rpb2*, *tub2*)
*Hypoxylon chrysalidosporum*	FCATAS 2710	China	HT	OL467294	OL615106	OL584222	OL584229	Ma et al. (2022)
*Hypoxylon crocopeplum*	CBS 119004	France		KC968907	KY610445	KY624255	KC977268	Kuhnert et al. (2014; ITS, *tub2*), Wendt et al. (2018; LSU, *rpb2*)
*Hypoxylon cyclobalanopsidis*	FCATAS 2714	China	HT	OL467298	OL615108	OL584225	OL584232	Ma et al. (2022)
*Hypoxylon damuense*	FCATAS 4207	China	HT	ON075427	ON075433	ON093251	ON093245	Song et al. (2022b)
*Hypoxylon delonicis*	MFLU 16–1031	Thailand	HT	MT215503	MT386008	N/A	MT212215	Perera et al. (2020)
*Hypoxylon duranii*	ATCC 58730	Mexico	HT	PP718984	PP729636	PP732085	PP721316	Cedeño-Sanchez et al. (2025)
*Hypoxylon dussii*	MUCL 53766	Guadeloupe	HT	PP718981	PP729635	PP732081	PP721315	Cedeño-Sanchez et al. (2025)
*Hypoxylon erythrostroma*	MUCL 53759	Martinique		KC968910	ON954154	OP251031	N/A	Kuhnert et al. (2014; ITS), Cedeño-Sanchez et al. (2023; LSU, *rpb2*)
*Hypoxylon eurasiaticum*	MUCL 57720	Iran	HT	MW367851	N/A	MW373852	MW373861	Lambert et al. (2021)
*Hypoxylon fendleri*	MUCL 54792	French Guiana		KF234421	KY610481	KY624298	KF300547	Kuhnert et al. (2014; ITS, *tub2*), Wendt et al. (2018; LSU, *rpb2*)
*Hypoxylon ferrugineum*	CBS 141259	Austria		KX090079	N/A	N/A	KX090080	Friebes and Wendelin (2016)
*Hypoxylon fragiforme*	MUCL 51264	Germany	ET	KC477229	KM186295	MK887342	KX271282	Stadler et al. (2013; ITS), Daranagama et al. (2015; LSU), Wendt et al. (2018; *tub2*), Sir et al. (2019; *rpb2*)
*Hypoxylon fuscoides*	MUCL 52670	France	HT	ON792789	ON954145	OP251038	ON813076	Cedeño-Sanchez et al. (2023)
*Hypoxylon fuscum*	CBS 113049	France	ET	KY610401	KY610482	KY624299	KX271271	Wendt et al. (2018)
*Hypoxylon gibriacense*	MUCL 52698	France	HT	KC968930	ON954146	N/A	ON813074	Kuhnert et al. (2014; ITS), Cedeño-Sanchez et al. (2023; LSU, *rpb2*, *tub2*)
*Hypoxylon griseobrunneum*	CBS 331.73	India	HT	KY610402	KY610483	KY624300	KC977303	Kuhnert et al. (2014; *tub2*), Wendt et al. (2018; ITS, LSU, *rpb2*)
*Hypoxylon guilanense*	MUCL 57726	Iran	HT	MT214997	MT214992	MT212235	MT212239	Pourmoghaddam et al. (2020)
*Hypoxylon haematostroma*	MUCL 53301	Martinique	ET	KC968911	KY610484	KY624301	KC977291	Kuhnert et al. (2014; ITS, *tub2*), Wendt et al. (2018; LSU, *rpb2*)
*Hypoxylon hainanense*	FCATAS2712	China	HT	OL467296	OL616132	OL584224	OL584231	Ma et al. (2022)
*Hypoxylon hinnuleum*	MUCL 3621	USA	HT	MK287537	MK287549	MK287562	MK287575	Sir et al. (2019)
*Hypoxylon howeanum*	MUCL 47599	Germany		AM749928	KY610448	KY624258	KC977277	Bitzer et al. (2008; ITS), Kuhnert et al. (2014; *tub2*), Wendt et al. (2018; LSU, *rpb2*)
*Hypoxylon hypomiltum*	MUCL 51845	Guadeloupe		KY610403	KY610449	KY624302	KX271249	Wendt et al. (2018)
*Hypoxylon inaequale*	KUNCC 22–10798	China	HT	ON329812	ON329815	N/A	N/A	Jayawardena et al. (2022)
*Hypoxylon invadens*	MUCL 51475	France	HT	MT809133	MT809132	MT813037	MT813038	Becker et al. (2020)
*Hypoxylon investiens*	CBS 118183	Malaysia		KC968925	KY610450	KY624259	KC977270	Kuhnert et al. (2014; ITS, *tub2*), Wendt et al. (2018; LSU, *rpb2*)
*Hypoxylon investiens*	TBRC 16251	Thailand		OP856531	OP856521	OQ108848	OQ144968	Suetrong et al. (2023)
*Hypoxylon isabellinum*	MUCL 53308	Martinique	HT	KC968935	ON954155	N/A	KC977295	Kuhnert et al. (2014; ITS, *tub2*), Cedeño-Sanchez et al. (2023; LSU, *rpb2*)
*Hypoxylon jianfengense*	FCATAS 845	China	HT	MW984546	MZ029707	MZ047260	MZ047264	Song et al. (2022a)
*Hypoxylon larissae*	FCATAS844	China	HT	MW984548	MZ029706	MZ047258	MZ047262	Song et al. (2022a)
*Hypoxylon laschii*	MUCL 52796	France		JX658525	ON954147	OP251027	ON813075	Stadler et al. (2014; ITS), Cedeño-Sanchez et al. (2023; LSU, *rpb2*, *tub2*)
*Hypoxylon lateripigmentum*	MUCL 53304	Martinique	HT	KC968933	KY610486	KY624304	KC977290	Kuhnert et al. (2014; ITS, *tub2*), Wendt et al. (2018; LSU, *rpb2*)
*Hypoxylon lateripigmentum*	**MUCL 57716**	**Iran**		**PV522286**	**PV522295**	**PV524401**	**PV524410**	**This study**
*Hypoxylon lateripigmentum*	**MUCL 57717**	**Iran**		**PV522287**	**PV522296**	**PV524402**	**PV524411**	**This study**
*Hypoxylon lateripigmentum*	**MUCL 57718**	**Iran**		**PV522288**	**PV522297**	**PV524403**	**PV524412**	**This study**
*Hypoxylon lechatii*	MUCL 54609	French Guiana		KF923407	ON954148	OP251033	KF923405	Kuhnert et al. (2014; ITS, *tub2*), Cedeño-Sanchez et al. (2023; LSU, *rpb2*)
*Hypoxylon lenormandii*	CBS 119003	Ecuador		KC968943	KY610452	KY624261	KC977273	Kuhnert et al. (2014; ITS, *tub2*), Wendt et al. (2018; LSU, *rpb2*)
*Hypoxylon lienhwacheense*	MFLUCC 14-1231	Thailand		KU604558	MK287550	MK287563	KU159522	Sir et al. (2016; ITS, *tub2*), Sir et al. (2019; LSU, *rpb2*)
*Hypoxylon lignicola*	MFLUCC 16-0926	China	HT	MK828609	MK835808	MN156534	N/A	Luo et al. (2019)
*Hypoxylon lividipigmentum*	BCRC 34077	Mexico	IT	JN979433	N/A	N/A	AY951735	Hsieh and Rogers (2005)
*Hypoxylon lividipigmentum*	STMA 14045	Argentina		ON792788	ON954149	PP732080	ON813077	Cedeño-Sanchez et al. (2023; ITS, LSU, *tub2*), Cedeño-Sanchez et al. (2025; *rpb2*)
*Hypoxylon luteogranulatum*	BCC 79720	Thailand	HT	PP955304	PP955701	PP968827	PQ000351	Wongkanoun et al. (2025)
*Hypoxylon macrocarpum*	CBS 119012	Germany		ON792785	ON954151	N/A	ON813071	Cedeño-Sanchez et al. (2023)
*Hypoxylon mangrovei*	MFLU 18-0559	Thailand	HT	MN047116	MN017880	N/A	MN077053	Dayarathne et al. (2020)
*Hypoxylon medogense*	FCATAS 4061	China	HT	ON075425	ON075431	ON093249	ON093243	Song et al. (2022b)
*Hypoxylon munkii*	MUCL 53315	Martinique		KC968912	ON954153	OP251035	KC977294	Kuhnert et al. (2014; ITS, *tub2*), Cedeño-Sanchez et al. (2023; LSU, *rpb2*)
*Hypoxylon musceum*	MUCL 53765	Guadeloupe		KC968926	KY610488	KY624306	KC977280	Kuhnert et al. (2014; ITS, *tub2*), Wendt et al. (2018; LSU, *rpb2*)
*Hypoxylon ochraceum*	MUCL 54625	Martinique	ET	KC968937	N/A	KY624271	KC977300	Kuhnert et al. (2014; ITS, *tub2*), Wendt et al. (2018; *rpb2*)
*Hypoxylon olivaceopigmentum*	DSM 107924	USA	HT	MK287530	MK287542	MK287555	MK287568	Sir et al. (2019)
*Hypoxylon perforatum*	STMA 23134	USA	ET	PP718982	PP729634	PP732084	PP721314	Cedeño-Sanchez et al. (2025)
*Hypoxylon perforatum*	CBS 115281	France		KY610391	KY610455	KY624224	KX271250	Wendt et al. (2018)
*Hypoxylon petriniae*	CBS 114746	France	HT	KY610405	KY610491	KY624279	KX271274	Wendt et al. (2018)
*Hypoxylon phuphaphetense*	TBRC 16277	Thailand	HT	OP856538	OP856528	OQ108849	OQ144973	Preedanon et al. (2023)
*Hypoxylon pilgerianum*	STMA 13455	Martinique		KY610412	KY610412	KY624308	KY624315	Wendt et al. (2018)
*Hypoxylon porphyreum*	CBS 119022	France		KC968921	KY610456	KY624225	KC977264	Kuhnert et al. (2014; ITS, *tub2*), Wendt et al. (2018; LSU, *rpb2*)
*Hypoxylon pseudofuscum*	DSM 112038	Germany	HT	MW367857	MW367848	MW373858	MW373867	Lambert et al. (2021)
*Hypoxylon pulicicidum*	CBS 122622	Martinique	HT	JX183075	KY610492	KY624280	JX183072	Bills et al. (2012; ITS, *tub2*), Wendt et al. (2018; LSU, *rpb2*)
*Hypoxylon rickii*	MUCL 53309	Martinique	ET	KC968932	KY610416	KY624281	KC977288	Kuhnert et al. (2014; ITS, *tub2*), Wendt et al. (2018; LSU, *rpb2*)
*Hypoxylon rubiginosum*	MUCL 52887	Germany	ET	KC477232	KY610469	KY624266	KY624311	Stadler et al. (2013; ITS), Wendt et al. (2018; LSU, *rpb2*, *tub2*)
*Hypoxylon samuelsii*	MUCL 51843	Guadeloupe	ET	KC968916	KY610466	KY624269	KC977286	Kuhnert et al. (2014; ITS, *tub2*), Wendt et al. (2018; LSU, *rpb2*)
*Hypoxylon sofainense*	MUCL 54170	Guadeloupe	HT	PP718983	PP729633	PP732083	PP721313	Cedeño-Sanchez et al. (2025)
*Hypoxylon sporistriatatunicum*	UCH9542	Panama	HT	MN056426	ON954150	OP251036	MK908140	Cedeño-Sanchez et al. (2020; ITS, *tub2*); Cedeño-Sanchez et al. (2023; LSU, *rpb2*)
*Hypoxylon subgilvum*	STMA 24034	Panama		PP718985	PP729637	PP732082	PP721317	Cedeño-Sanchez et al. (2025)
*Hypoxylon teeravasati*	NFCCI-4216	India	HT	KY863509	MF385274	MG986895	MG986894	Phookamsak et al. (2019)
*Hypoxylon texense*	DSM 107933	USA	HT	MK287536	MK287548	MK287561	MK287574	Sir et al. (2019)
*Hypoxylon ticinense*	CBS 115271	France		JQ009317	KY610471	KY624272	AY951757	Hsieh and Rogers (2005; ITS, *tub2*), Wendt et al. (2018; LSU, *rpb2*)
*Hypoxylon trugodes*	MUCL 54794	Sri Lanka	ET	KF234422	KY610493	KY624282	KF300548	Kuhnert et al. (2014; ITS, *tub2*), Wendt et al. (2018; LSU, *rpb2*)
*Hypoxylon vogesiacum*	CBS 115273	France		KC968920	KY610417	KY624283	KX271275	Kuhnert et al. (2014; ITS), Kuhnert et al. (2017a; *tub2*), Wendt et al. (2018; LSU, *rpb2*)
*Hypoxylon wuzhishanense*	FCATAS 2708	China	HT	OL467292	OL615104	OL584220	OL584227	Ma et al. (2022)
*Hypoxylon xmatkuilense*	UADY:PR_3	Mexico	HT	OR807999	OR807990	OR825476	OR825467	Reyes et al. (2024)
*Hypoxylon xmatkuilense*	UADY:PR_118	Mexico	PT	OR807996	OR807995	PP239283	PP239284	Reyes et al. (2024)
*Hypoxylon zangii*	FCATAS 4029	China	HT	ON075423	ON075429	ON093247	ON093241	Song et al. (2022b)
*Hypoxylon zhaotongensis*	GMBCC1168	China	HT	OP597690	OP598100	N/A	N/A	Zhang et al. (2023)
*Jackrogersella cohaerens*	CBS 119126	Germany		KY610396	KY610497	KY624270	KY624314	Wendt et al. (2018)
*Jackrogersella minutella*	CBS 119015	Portugal		KY610381	KY610424	KY624235	KX271240	Kuhnert et al. (2017a; *tub2*), Wendt et al. (2018; ITS, LSU, *rpb2*)
*Jackrogersella multiformis*	CBS 119016	Germany	ET	KC477234	KY610473	KY624290	KX271262	Kuhnert et al. (2014; ITS), Kuhnert et al. (2017a; *tub2*), Wendt et al. (2018; LSU, *rpb2*)
*Parahypoxylon papillatum*	ATCC 58729	USA	HT	KC968919	KY610454	KY624223	KC977258	Kuhnert et al. (2014; ITS, *tub2*), Wendt et al. (2018; LSU, *rpb2*)
*Parahypoxylon ruwenzoriense*	MUCL 51392	D. R. Congo	HT	ON792786	ON954156	OP251039	ON813078	Cedeño-Sanchez et al. (2023)
*Phylacia globosa*	STMA 18042	Argentina		OQ437889	OQ437885	OQ453168	OQ453172	Lambert et al. (2023)
*Phylacia lobulata*	STMA 18032	Argentina	HT	OQ437892	OQ437882	OQ453166	N/A	Lambert et al. (2023)
*Phylacia surinamensis*	STMA 18044	Argentina		OQ437891	OQ437887	OQ453167	N/A	Lambert et al. (2023)
*Pyrenopolyporus bambusicola*	BCC89355	Thailand	HT	OP304856	OP304876	OP981624	OQ101839	Wongkanoun et al. (2023)
*Pyrenopolyporus cinereopigmentosus*	BCC89382	Thailand	HT	OP304860	OP304882	OP981627	OQ101843	Wongkanoun et al. (2023)
*Pyrenopolyporus hunteri*	MUCL 52673	Ivory Coast	ET	KY610421	KY610472	KY624309	KU159530	Kuhnert et al. (2017a; *tub2*), Wendt et al. (2018; ITS, LSU, *rpb2*)
*Pyrenopolyporus laminosus*	MUCL 53305	Martinique	HT	KC968934	KY610485	KY624303	KC977292	Kuhnert et al. (2014; ITS, *tub2*), Wendt et al. (2018; LSU, *rpb2*)
*Pyrenopolyporus macrosporus*	BCC89373	Thailand	HT	OP304870	OP304879	OP981621	OQ101844	Wongkanoun et al. (2023)
*Pyrenopolyporus nicaraguensis*	CBS 117739	Burkina Faso		AM749922	KY610489	KY624307	KC977272	Bitzer et al. (2008; ITS), Kuhnert et al. (2014; *tub2*), Wendt et al. (2018; LSU, *rpb2*)
*Rhopalostroma angolense*	CBS 126414	Ivory Coast		KY610420	KY610459	KY624228	KX271277	Wendt et al. (2018)
*Ruwenzoria pseudoannulata*	MUCL 51394	D. R. Congo	HT	KY610406	KY610494	KY624286	KX271278	Wendt et al. (2018)
*Thamnomyces dendroideus*	CBS 123578	French Guiana	HT	FN428831	KY610467	KY624232	KY624313	Stadler et al. (2010; ITS), Wendt et al. (2018; LSU, *rpb2*, *tub2*)
*Xylaria arbuscula*	CBS 126415	Germany		KY610394	KY610463	KY624287	KX271257	Fournier et al. (2011; ITS), Wendt et al. (2018; LSU, *rpb2*, *tub2*)
*Xylaria hypoxylon*	CBS 122620	Sweden	ET	KY610407	KY610495	KY624231	KX271279	Wendt et al. (2018)

Type specimens are labelled with HT (holotype), ET (epitype), and PT (paratype). Isolates/sequences in bold were isolated/sequenced in present study.

For the phylogenetic analyses, 120 accessions from 107 species of Hypoxylaceae and four outgroup taxa from Graphostromataceae (*Biscogniauxia nummularia*, *Graphostroma platystomum*) and Xylariaceae (*Xylaria arbuscula*, *X. hypoxylon*) were included. The sequence matrices of ITS, LSU, *rpb2*, and *tub2* were combined; after exclusion of ambiguously aligned and gap-rich regions, the resulting combined data matrix contained 3,863 alignment positions from four loci (547 from ITS, 942 from LSU, 1,045 from *rpb2*, and 1,329 from *tub2*).

Maximum likelihood (ML) analyses were performed with RAxML as implemented in raxmlGUI v. 2.0 (Edler et al. 2021) using the ML + rapid bootstrap setting with 1,000 bootstrap replicates and the GTRGAMMA substitution.

Maximum Parsimony (MP) analyses were performed with PAUP v. 4.0a169 (Swofford 2002). All molecular characters were unordered and given equal weight; analyses were performed with gaps treated as missing data; the COLLAPSE command was set to MINBRLEN. MP analysis of the combined multilocus matrix was done using 1,000 replicates of heuristic search with random addition of sequences and subsequent TBR branch swapping (MULTREES option in effect, steepest descent option not in effect). Bootstrap analyses with 1,000 replicates were performed in the same way, but using 10 rounds of random sequence addition and subsequent branch swapping during each bootstrap replicate. Bootstrap values ≤ 70% are considered low, between 70% and 90% intermediate, and ≥ 90% high.

### 2.4. HPLC profiling

Stromata of *Hypoxylon* and *Annulohypoxylon* specimens were extracted essentially as described in Kuhnert et al. (2017a). Briefly, stromatal tissue was scraped with a spatula and collected in an Eppendorf tube, covered with acetone, centrifuged and 60–100 µL collected for subsequent HPLC-DAD-UV/Vis-ESI-MS analysis. System parameters and settings were identical to Kuhnert et al. (2014). Resulting spectra were analysed using DataAnalysis V4.4 (Bruker Daltonics) and compared with internal databases for identification of discernible compound peaks and stromata-fingerprints.

### 2.5. Extraction and isolation of secondary metabolites

To study the chemical constituents identified by HPLC fingerprinting as described above, stromata of *Hypoxylon lateripigmentum* (GUM 1594, GUM 1595), *Annulohypoxylon michelianum* (KR-M-0002761), and *A. substygium* (GUM 1621, GUM 1619, GUM 1620, GUM 1624) were scraped and extracted by covering the material with acetone, followed by evaporation and transfer to a separate vial. This process was repeated three times, yielding in total 40 mg (GUM 1594; **I**), 26.1 mg (GUM 1595; **II**), 32.4 mg (STMA 16011; **III**), and 516.5 mg (GUM 1621, GUM 1619, GUM 1620, and GUM 1624; **IV**).

Extract **I** was purified on a reversed-phase (RP) HPLC system (Gilson, Middleton, WI, USA; GX-271 liquid handler, two pumps; 305 and 306, DAD wavelengths set to 220, 255, and 350 nm) using a C18-Gemini 10 µm; 250 × 21 mm column (A, Acetonitrile; B, H_2_O; flow rate set to 20 mL/min). The mobile phase was set to 50% B in isocratic conditions for 3 min, then increasing to 65% B in 45 min, another 2 min to 100% B and 100% B isocratic condition for 10 min. This yielded 3.9 mg of compound **2** at RT = 19.4–22.7 min and 0.3 mg of **4** at RT = 40.1–41.1 min.

Extract **II** was purified on the RP-HPLC system described above, with identical settings for the mobile phase. This yielded 0.3 mg of **3** at RT = 8.7–9 min, 0.8 mg of compound **1** at RT = 10.4 min, and 1.2 mg of **2** at RT = 11.4 min.

Extract **III** was purified using the same equipment, with the mobile phase set to 55% B in isocratic conditions for 3 min; increasing B to 85% in 40 min, then increasing to 100% in 3 min and 10 min of 100% B at again isocratic condition. This yielded 0.5 mg of compound **3** at RT = 13.2–14.5 min, 2.2 mg of **1** at RT = 19.1–20 min, 0.5 mg of **5** at RT = 27.7–28.4 min, and 2.6 mg of minutellin D (**6**) at RT = 33–34 min.

Extract **IV** was pre-purified by open normal-phase (NP) chromatography using 20 g of packed silica gel 60 (0.05–0.2 mm). Subsequent extraction with in total eight different solvent mixtures followed: 200 mL of 100% dichloromethane (DCM); 200 mL of 98% DCM and 2% acetone; 200 mL of 95% DCM and 5% acetone; 200 mL of 93% DCM and 7% acetone; 200 mL of 90% DCM and 10% acetone; 200 mL of 80% DCM and 20% acetone; 200 mL of 100% acetone and 50 mL of 100% methanol. This process yielded 40.1 mg of fraction **V** following extraction with 90% DCM and 10% acetone, which was further purified using the same RP-HPLC equipment as stated above. The mobile phase was set to 15% B at isocratic condition for 10 min; increasing to 25% in 15 min, remaining isocratic for 5 min; increasing to 40% B in 15 min and increasing to 80% B for 15 min. This yielded 0.7 mg of compound **7** at RT = 20.4–21.7 min and 1.7 mg of **8** at RT = 25–26 min.

### 2.6. Spectral data

***Minutellin E*** ***(1):*** yellow oil; ${\left[\alpha \right]}_{D}^{20}$ +62 (c 0.1, acetonitrile); CD (acetonitrile): λ (Δε) 213 (+66.90), 275 (−42.40), 354 (+39.44), 417 (−25.80); UV-Vis (acetonitrile): λmax (log ε) 193 (0.62), 229 (0.58), 267 (0.64), 339 (0.56); NMR data (^1^H: 700 MHz, ^13^C: 175 MHz, acetone-*d*_6_) see Tables 1 and 2; HR-ESI-MS: *m/z* 495.2378 [M+H]^+^ (calcd. 495.5840 for C_29_H_35_O_7_^+^); tR = 15.26 min (HR-ESI-MS); C_29_H_34_O_7_ (494.5761 g mol^−1^).

**Table 2.** ^1^H data (700 MHz) of **1**–**5**.

Pos.	1^a^	2^b^	3^b^	4^a^	5^a^
1	8.77, br s	8.81, br s	6.87, br s	8.74, s	7.16, m
4	6.40, s	6.10, s	6.08, s	6.49, s	6.42, s
5	5.40 d (1.1)	5.32 d (1.1)	5.46 d (1.1)	5.31, m	5.44 d (1.1)
8			3.32, dt (9.7, 1.9)		3.32, dt (9.5, 2.3)
9	1.67, s	1.73, s	1.12, s	1.67, s	1.07, s
10		2.97, d (12.1)		3.46, d (12.5)	
12	2.73, dd (15.9, 3.7) 2.54, dd (15.9, 7.3)	2.91, ddd (13.5, 5.0, 2.0) 2.46, dd (13.5, 11.6)	2.81, m 2.61, m	6.05, dd (10.1, 2.2)	6.82, d (8.1)
13	4.33, m	4.07, tt (11.5, 4.7)	4.39, m	7.18, m	7.19, t (8.1)
14	2.90, m 2.63, dd (18.4, 6.1)	2.35, m 1.62, m	2.81, m 2.62, m	2.63, m 2.32, ddt (19.0, 10.8, 2.6)	6.79, br d (8.1)
15		2.13, m		2.56, m	
16	2.11, s	1.09, d (6.5)	2.06, s	1.06, d (6.7)	2.23, s
18			3.19, dd (17.2, 2.2) 2.72, dd (17.2, 9.7)		3.12, dd (17.9, 2.3) 2.99, dd (17.9, 9.5)
20	3.11, ddd (18.3, 8.3, 6.3) 2.88, m	3.20, ddd (18.3, 9.0, 6.2) 2.83, ddd (18.3, 8.9, 5.5)	2.54, m	3.09, m 2.89, m	2.65, t (7.4)
21	1.60, m	1.63, m 1.56, m	1.61, m	1.59, m	1.61, m
22–25	1.20–1.35, m	1.23–1.33, m	1.23–1.35, m	1.20–1.35, m	1.26–1.34, m
26	1.27, m	1.24, m	1.26, m	1.26, m	1.26, m
27	1.29, m	1.28, m	1.30, m	1.29, m	1.29, m
28	0.87, t (7.1)	0.87, t (7.1)	0.89, t (7.1)	0.87, t (6.9)	0.87, t (7.1)

^a^ measured in acetone-*d*_6_, ^b^ measured in CHCl_3_-*d*.

***Minutellin F (2):*** yellow amorphous solid; ${\left[\alpha \right]}_{D}^{20}$-39 (c 0.3, acetonitrile); CD (acetonitrile): λ (Δε) 213 (+42.90), 274 (−26.61), 349 (+30.38), 414 (−21.94); UV-Vis (a33cetonitrile): λmax (log ε) 262 (1.14), 336 (0.91); NMR data (^1^H: 700 MHz, ^13^C: 175 MHz, CDCl_3_-*d*) see Tables 1 and 2; HR-ESI-MS: *m/z* 497.2533 [M+H]^+^ (calcd. 497.599 for C_29_H_37_O_7_^+^), 529.2425 [M+Na]^+^ (calcd. 529.5817 for C_29_H_36_NaO_7_^+^); tR = 15.49 min (HR-ESI-MS); C_29_H_36_O_7_ (496.5919 g mol^−1^).

***Minutellin G (3):*** yellow amorphous solid; ${\left[\alpha \right]}_{D}^{20}$-39 (c 0.1, acetonitrile); CD (acetonitrile): λ (Δε) 203 (+12.06), 249 (−6.80), 323 (+17.95), 422 (−1.54); UV-Vis (acetonitrile): λmax (log ε) 229 (0.299), 345 (0.30); NMR data (^1^H: 700 MHz, ^13^C: 175 MHz, CDCl_3_-*d*) see Tables 1 and 2; HR-ESI-MS: *m/z* 471.2743 [M+H]^+^ (calcd. 471.6057 for C_28_H_39_O_6_^+^); tR = 14.14 min (HR-ESI-MS); C_28_H_38_O_6_ (470.5977 g mol^−1^).

***Minutellin H (4):*** yellow amorphous solid; ${\left[\alpha \right]}_{D}^{20}$-39 (c 0.1, acetonitrile); CD (acetonitrile): λ (Δε) 212 (+9.49), 272 (−8.15), 335 (+6.09), 415 (−3.83); NMR data (^1^H: 700 MHz, ^13^C: 175 MHz, CDCl_3_-*d*) see Tables 1 and 2; HR-ESI-MS: *m/z* 479.2434 [M+H]^+^ (calcd. 479.5846 for C_29_H_35_O_6_^+^), 501.2246 [M+Na]^+^ (calcd. 501.5664 for C_29_H_34_NaO_6_^+^); tR = 14.83 min (HR-ESI-MS); C_29_H_34_O_6_ (478.5767 g mol^−1^).

***Minutellin I (5):*** yellow amorphous solid; ${\left[\alpha \right]}_{D}^{20}$-39 (c 0.1, acetonitrile); UV-Vis (acetonitrile): λmax (log ε) 348 (0.076); NMR data (^1^H: 700 MHz, ^13^C: 175 MHz, CDCl_3_-*d*) see Tables 1 and 2; *m/z* 453.2645 [M+H]^+^ (calcd. 453.5904 for C_28_H_37_O_5_^+^), 475.2453 [M+Na]^+^ (calcd. 475.5722 for C_28_H_36_NaO_5_^+^); tR = 14.15 min (HR-ESI-MS); C_28_H_36_O_5_ (452.5824 g mol^−1^).

***Daldinone L (8):*** Brown amorphous solid; ${\left[\alpha \right]}_{D}^{20}$ +275 (c 0.1, acetonitrile); NMR data (^1^H: 700 MHz, ^13^C: 175 MHz, CDCl_3_-*d*) see Table 4; *m/z* 335.0911 [M-H_2_O+H]^+^ (calcd. 335.3301 for C_20_H_15_O_5_^+^), 353.1016 [M+H]^+^ (calcd. 353.3454 for C_20_H_17_O_6_^+^), 375.0837 [M+Na]^+^ (calcd. 375.3272 for C_20_H_16_NaO_6_^+^); tR = 7.05 min (HR-ESI-MS); C_20_H_16_O_6_ (352.374 g mol^−1^).

### 2.7. Antimicrobial activity of compounds 1–5

Compounds **1–5** were subjected to a serial dilution assay in 96-well microtiter plate format to determine the minimal inhibitory concentration (MIC) against *Escherichia coli* (DSM 1116), *Bacillus subtilis* (DSM 10), and *Mucor hiemalis* (DSM 2656) essentially as described by Surup et al. (2015).

## 3. Results

### 3.1. Molecular phylogeny

**Figure 1.**
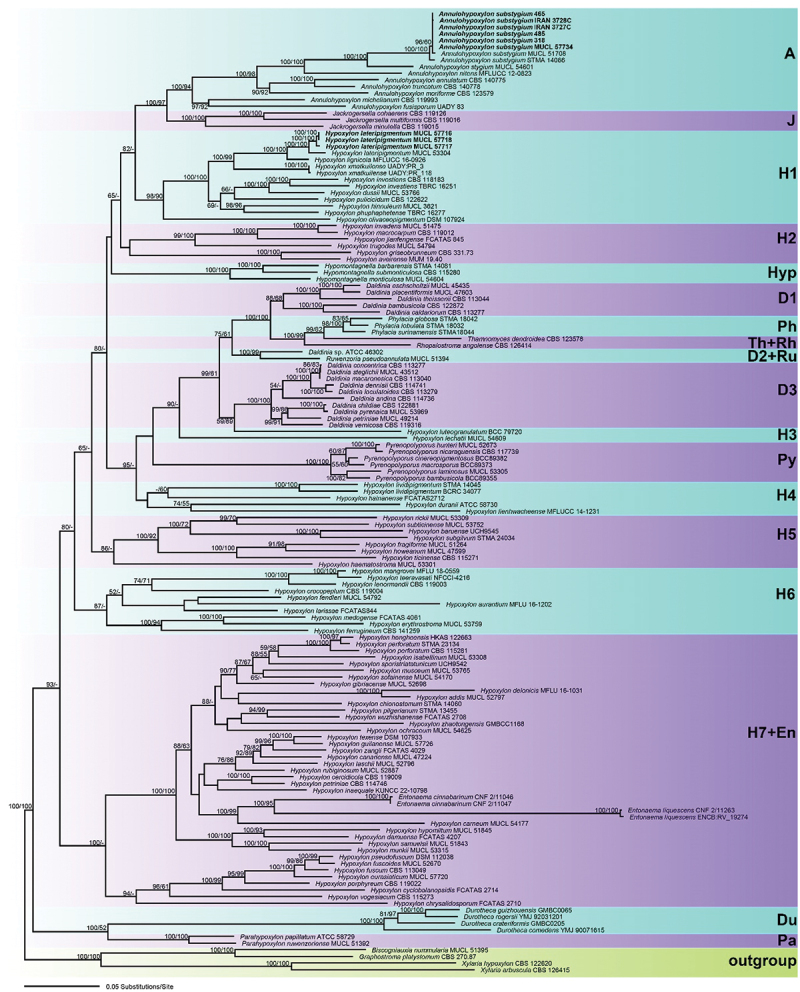
Phylogram of the best ML trees (lnL = −107,247.1419) revealed by RAxML from an analysis of the combined ITS-LSU-*rpb2-tub2* matrix of selected Hypoxylaceae. Strains in bold were sequenced in the current study. ML and MP bootstrap support above 50% are given at the first and second positions, respectively, above or below the branches.

Of the 3,863 characters of the combined matrix, 1,734 were parsimony informative (345 in ITS, 201 in LSU, 515 in *rpb2*, and 673 in *tub2*). The phylogram of the best ML tree (lnL = −107,247.1419) obtained by RAxML is shown as Figure 1. Maximum parsimony analyses resulted in five parsimonious tree [tree length = 24,727; Consistency Index (CI) = 0.158; Retention Index (RI) = 0.564 and Homoplasy Index (HI) = 0.842] (not shown). The alignment is provided as Table S1 in the Supporting Information.

The phylogenies revealed a paraphyly of *Hypoxylon*, with the genera *Annulohypoxylon*, *Daldinia*, *Durotheca*, *Entonaema*, *Jackrogersella*, *Hypomontagnella*, *Parahypoxylon*, *Phylacia*, *Pyrenopolyporus*, *Rhopalostroma*, *Ruwenzoria*, and *Thamnomyces* embedded within the former, as reported in previous studies (e.g. Pourmoghaddam et al. 2020; Lambert et al. 2021; Cedeño-Sanchez et al. 2023). All of the latter genera appeared monophyletic except for *Daldinia* (Figure 1).

In our phylogenetic tree, *Hypoxylon* resolved into seven groups (H1–H7 in Figure 1). The *Annulohypoxylon* and *Hypoxylon* species examined in this study clustered in two subclades (A and H1). All major groups and deeper, highly supported nodes were consistent between the ML and MP analyses, but topologies of deeper unsupported nodes differed in the MP tree; as these differences are not relevant within the context of our species, they are not further considered here.

In subclade A, the sequences of all Iranian strains of *Annulohypoxylon substygium* clustered together with maximum support and were shown to be almost identical to those of the ex-epitype culture (MUCL 51708) and *A. substygium* (STMA 14066).

In subclade H1, the Iranian *Hypoxylon* species clustered as sister species to *H. lateripigmentum* (MUCL 53304) with maximum ML and MP BS support, and both form a sister group to *H. lignicola* (MFLUCC 16-0926), also with maximum ML and MP BS support (Figure 1).

### 3.2. HPLC profiling

**Figure 2.**
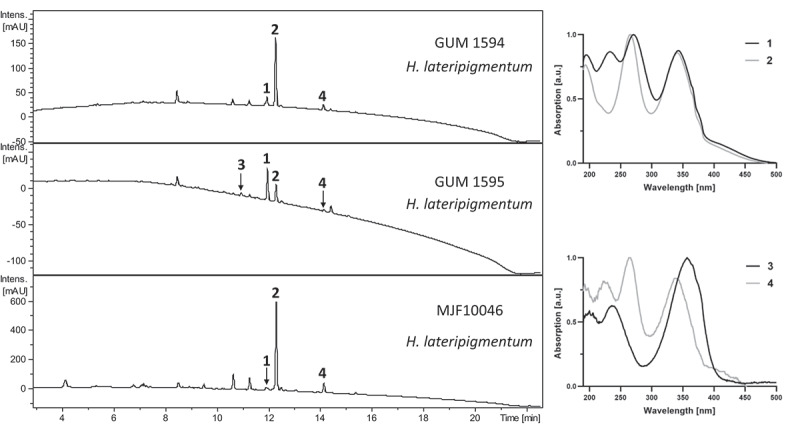
HPLC-UV/Vis chromatogram (210 nm) of an acetone stroma extract of specimen GUM 1594 and GUM 1595 (top and middle panel) and the holotype of *Hypoxylon lateripigmentum*, MJF10046 (below). Right panels: UV/Vis absorption spectra of designated compounds.

The HPLC chromatograms of *Hypoxylon lateripigmentum* specimens GUM 1594, GUM 1595 (shown in Figure 2), and GUM 1596 (not shown) appeared to be highly similar, containing severable compounds with UV/Vis absorption patterns resembling the minutellin-type azaphilone family. However, the compounds were not identifiable. The bottom panel of Figure 2 shows a stromatal acetone extract of the holotype of *H. lateripigmentum*, which confirms the phylogenetic affinities of the isolated strains.

**Figure 3.**
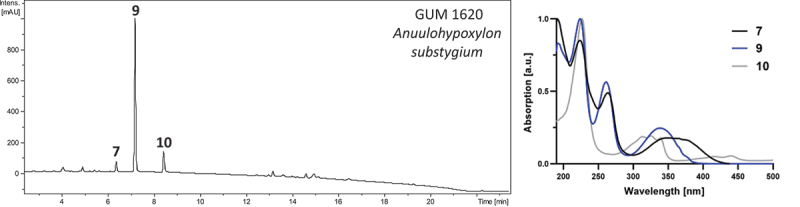
Representative HPLC-UV/Vis chromatogram (210 nm) of an acetone stroma extract of specimen *Annulohypoxylon substygium* (GUM 1620). Right panel: UV/Vis absorption spectra of designated compounds. Compounds **7**, **9**, and **10** were identified as daldinone F, daldinone A, and BNT, respectively.

The HPLC chromatogram of *Annulohypoxylon substygium* specimens GUM 1620 (Figure 3), GUM 1618, GUM 1619, GUM 1621, GUM 1622, GUM 1624 (not shown) appeared practically identical, featuring two compounds (**7** and **10**) in minor and one compound (**9**) in higher abundance. Compounds **9** and **10** could be identified as daldinone A and BNT, respectively, while compound **7** showed affinities to the daldinone family.

As we had access to a considerable amount of stromata for chemical extraction, we next attempted to characterise the unknown components detected in the specimen identified as *H. lateripigmentum* and potential novel minor components in *A. substygium*. Concurrently, we analysed the stromatal constituents of a specimen of *A. michelianum* (KR-M-0002761) that was reported to feature similar minutellin-type azaphilones (Kuhnert et al. 2017a), which we hence included in this study.

### 3.3. Structure elucidation of minutellins 1–6

Minutellin E (**1**) was isolated as a yellow oil; its molecular formula C_29_H_34_O_7_ was deduced from the molecular ion cluster at *m/z* 495.2378 in the HR-ESI-MS spectrum. ^1^H and HSQC NMR spectra revealed the presence of three olefinic methine, six resolved methylene and two methyl signals (Tables 2 and 3). Further unresolved methylenes in the region of *δ*_H_ 1.30/*δ*_C_ 30.0 ppm indicated a long fatty acid side chain. NMR data including chemical shifts of **1** were virtually identical to those of minutellin A, so a common scaffold including stereochemistry was assigned (Kuhnert et al. 2017b). However, the molecular formula of **1** entails two additional methylene carbons in the fatty acid side chain compared to minutellin A.

**Table 3.** ^13^C data (175 MHz) of **1**–**5**.

Pos.	1^a^	2^b^	3^b^	4^a^	5^a^
1	154.4, CH	153.5, CH	145.1, CH	153.4, CH	146.5, CH
3	154.9, C	156.7, C	152.9, C	158.3, C^x1^	155.5, C
4	113.8, CH	111.9, CH	111.8, CH	112.1, CH	112.0, CH
4a	144.5, C	143.1, C	146.7, C	n.o.	148.4, C
5	106.4, CH	105.8, CH	105.4, CH	105.5, CH	105.4, CH
6	190.7, C	190.5, C	199.2, C	190.4, C	199.0, C
7	88.4, C	87.7, C	73.1, C	87.8, C	73.7, C
8	165.9, C	165.0, C	40.6, CH	165.1, C	41.6, CH
8a	112.0, C	111.2, C	120.5, C	111.2, C	120.9, C
9	26.3, CH_3_	26.3, CH_3_	21.3, CH_3_	25.6, CH_3_	21.6, CH_3_
10	130.7, C	64.5, CH	130.8, C	59.3, CH	121.2, C
11	194.8, C	202.8, C	193.7, C	194.6, C	156.3, C
12	47.1, CH_2_	50.4, CH_2_	46.3, CH_2_	128.7, CH	114.4, CH
13	65.8, CH	67.8, CH	65.4, CH	151.1, CH	131.8, CH
14	41.9, CH_2_	42.4, CH_2_	40.9, CH_2_	33.9, CH_2_	122.3, CH
15	162.6, C	30.6, CH	160.1, C	33.3, CH	139.6, C
16	23.0, CH_3_	20.5, CH_3_	23.0, CH_3_	19.4, CH_3_	20.1, CH_3_
17	169.3, C	168.0, C		167.8, C	
18	124.6, C	123.7, C	38.9, CH_2_	124.0, C	39.2, CH_2_
19	197.6, C	197.2, C	209.8, C	197.0, C	209.3, C
20	42.4, CH_2_	42.2, CH_2_	42.8, CH_2_	41.8, CH_2_	43.2, CH_2_
21	24.2, CH_2_	23.3, CH_2_	24.0, CH_2_	23.6, CH_2_	24.6, CH_2_
22	29.9, CH_2_	29.0, CH_2_	29.2, CH_2_	*	29.0, CH_2_
23–25	*	29.2, 29.40, 29.41	29.2, 29.38, 29.40	*	*
26	32.7, CH_2_	31.8, CH_2_	31.8, CH_2_	32.1, CH_2_	32.7, CH_2_
27	23.4, CH_2_	22.6, CH_2_	22.6, CH_2_	22.8, CH_2_	23.4, CH_2_
28	14.4, CH_3_	14.1, CH_3_	14.1, CH_2_	13.8, CH_3_	14.4, CH_3_

* overlapped by solvent signal, n.o.: not observed, x1: chemical shift extracted from HMBC crosspeak, ^a^ measured in acetone-*d*_6_, ^b^ measured in CHCl_3_-*d*.

For minutellin F (**2**), the molecular formula of C_28_H_40_O_6_, generated from the molecular ion cluster at *m/z* 497.2533 in the HR-ESI-MS spectrum, indicated the formal addition of two hydrogen atoms compared to **1**. NMR data of **2** were similar to those of **1** with these key differences: two methines replaced the olefinic carbons C–10 and C–16, additionally the methyl CH_3_–16 multiplicity in the ^1^H NMR spectrum changed to doublet. Consequently, a 2-oxo-4-hydroxy-6-methylhexanyl- moiety was deduced for **2**. The large coupling constant *J*_10,15_ = 12.1 hz indicated a *trans* configuration of these protons with pseudo-axial orientations, and the ROESY correlation between H–15 and H–13 confirmed H–13 to be also axial and on the same side as H–15.

Minutellin G (**3**) had the molecular formula C_28_H_38_O_6_, indicating the formal loss of one carbon and one oxygen atom and the formal addition of four protons compared to **1**. 1D and 2D NMR data indicated the loss of C–17 and the replacement of olefinic carbons C–8 and C–18 by a methine and a methylene, respectively, as key differences.

Minutellin H (**4**) resembled **2** in its NMR data, with the exemption of the replacement of methylene C–12 and oxymethine C–13 by the *δ*^12,13^ double bond. This modification was confirmed by the molecular formula C_29_H_34_O_6_, denoting a loss of H_2_O.

The molecular formula C_28_H_36_O_5_ of minutellin I (**5**) indicated a formal loss of H_2_O compared to **3**. The 2-hydroxy-6-methyl-benzyl moiety was confirmed by the aromatic signals for H–12 to H–14 in the ^1^H and C–10 to C–15 in the ^13^C spectrum.

Minutellin D (**6**) was identified based on comparison of its NMR and HRMS data to the literature (Kuhnert et al. 2017b). Analogous to other minutellins, the CD curves of **1**–**5** with local minima around 250 and 275 nm and a characteristic maximum around 350 nm confirm a 7*R* absolute configuration (Pyser et al. 2019).

### 3.4. Structure elucidation of daldinones 7–9

Daldinone F (**7**) was identified by comparison of their NMR and HRMS data with previous reports (Wang et al. 2015). However, a close inspection of the NMR data of **7** revealed that various signals had been assigned incorrectly by Wang et al. (2015). Most notably, signals for H_2_-8 and H_2_-18 had been confused. However, chemical shift analysis as well as COSY and HMBC correlations (see Figure S32 for details) require the reassignment of the signals for C–1, C–4, C–8, H_2_–8, C–11, C–15, C–17, H_2_–17, C–20 (Table 4).

**Table 4.** NMR data of daldinones F (**7**^b^; 700 MHz, CHCl_3_-*d*) and L (**8**; 700 MHz, CHCl_3_-*d*), comparison with data of Wang et al. (2015).

	7^a^ as assigned by Wang et al. (2015)		7^b^ as assigned in this study		8^b^	
Pos.	*δ*_C_, mult.	*δ*_H_, mult.	*δ*_C_, mult.	*δ*_H_, mult.	*δ*_C_, mult.	*δ*_H_, mult.
1	**142.8**		120.3, C		115.9, C	
2	161.7	OH: 12.39	161.5, C	OH: 12.41, br s	156.2, C	OH: 11.85, s
3	115.5	6.82, d (8.5)	117.5, CH	6.89, m	118.5, CH	6.77, d (9.0)
4	**123.8**	7.45, d (8.5)	137.7, CH	7.50, ps t (8.1)	127.6, CH	7.17, d (9.0)
5	**117.3**	**6.87,** **d** **(8.5)**	120.3, CH	7.20, dd (7.6, 1.0)	148.5, C	OH: 8.79, br s
6	143.2		143.0, C		128.2, C	
7	203.2		203.2, C		204.4, C	
8	**27.2**	**2.42, dd (13.5, 4.0) 2.24 (dd, 13.5, 4.9)**	36.1, CH_2_	3.26, dd (17.4, 5.6) 2.88, dd (17.4, 2.0)	37.3, CH_2_	3.27, dd (16.4, 5.0) 2.85, dd (16.4, 2.6)
9	56.2	2.46–2.51	56.5, CH	2.68, ddd (11.0, 5.6, 2.0)	55.6, CH	2.68, ddd (10.3, 5.0, 2.6)
10	79.8		79.5, C		82.0, C	OH: 5.45, br s
11	**120.2**		137.7, C		138.0, C	
12	123.8	7.55, d (8.0)	123.9, CH	7.54, d (8.2)	135.2, CH	8.17, d (8.6)
13	**120.1**	**7.21,** **d** **(8.0)**	115.6, CH	6.86, br d (8.2)	116.1, CH	6.76, d (8.6)
14	156.7		156.4, C		161.7, C	OH: 11.15, s
15	**138.0**		120.3, C		114.4, C	
16	69.8	5.08, q (8.5)	69.5, CH	5.10, m	204.9, C	
17	**35.8**	**3.24, dd (15.0, 5.5) 2.85,** **d** **(15.0)**	33.8, CH_2_	2.42, m	38.6, CH_2_	2.80, m 2.64, m
18	**33.7**	**1.69, q** **(8.0) 1.47, q** **(8.0)**	27.3, CH_2_	2.05, m 1.46, m	29.3, CH_2_	2.49, m 1.83, m
19	42.5	2.59 (dt, 11.0, 4.5)	42.7, CH	2.89, td (11.0, 4.5)	43.1, CH	2.89, ddd (11.2, 10.3, 4.5)
20	**114.4**		142.6, C		151.0, C	

^a^ measured in CHCl_3_-*d*, ^b^ measured in acetone-*d*_6_. Previously misassigned signals are highlighted in bold.

The NMR data of compound **8** was highly similar to those of daldinone F, with the replacement of an aromatic methine by an oxygenised sp^2^ hybridised carbon as well as oxymethine CH–16 by a keton. Consequently, **8** was elucidated as (6b*S*,12a*R*,12b*R*)-4,6b,7,10-tetrahydroxy-1,2,6b,12,12a,12b-hexahydrobenzo[*j*]fluoranthene-3,11-dione, for which we propose the trivial name daldinone L.

^1^H and ^13^C NMR data of **9** matched those of not only daldinone A (Quang et al. 2002), but also daldinone C (Gu et al. 2007). This was surprising, since opposite stereo configurations of C–19 had been published for these metabolites. We addressed the stereochemistry of **9** by ROESY NMR data and observed a strong TOCSY artefact between H–9 and H–19. We conclude that Quang et al. (2002) misinterpreted this TOCSY artefact as a conventional cross peak, since they wrote: “NOE correlations were observed between … H–9 and H–19 … ” Based on this misinterpretation, a wrong relative stereochemistry was assigned between these protons. Gu et al. (2007) on the other hand assigned the correct structure, confirmed by X-ray data, but unfortunately did not realise that they had reisolated daldinone A and coined the name daldinone C instead of revising the structure of daldinone A. Additionally, the absolute stereochemistry of daldinone C was revised by Podlech and Gutsche (2023) by reinterpretation of CD data.

Taken together, we propose that daldinone A and C are identical and suggest only using the name daldinone A with its revised structure in the future.

### 3.5. Bioactivity of compounds 1–5

Subsequent testing for antimicrobial activity of compounds **1**–**5** revealed moderate activity exerted by **5** against *B. subtilis* (MIC = 8.34 µg/mL). No growth inhibitory activity was recorded for compounds **1**–**5** against the other tested organisms.

### 3.6. Production of minutellins

**Figure 4.**
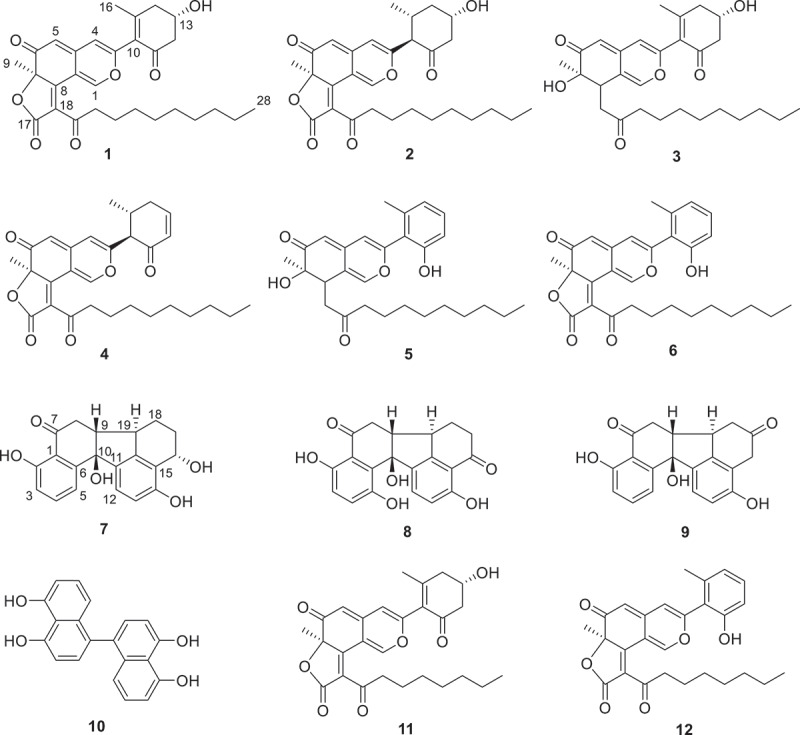
Structures of the new minutellins E–I (**1**–**5**), minutellin D (**6**), daldinone F, and its new derivative L (**7** and **8**), revised structure of daldinone A (**9**), binaphthalene-tetrol (BNT; **10**), and minutellins A and C (**11** and **12**) discussed in the present study.

We realised that the stroma of the neotropical *H. pulicicidum* was reported to harbour similar minutellin-type azaphilones and since we had access to the holotype specimen, compared the stromatal constituents of the holotype with our pure standards. We were able to correlate in total three detectable compounds, i.e. minutellin A, C, and D with components of its stromatal extract (Figure S39). Table 5 summarises the distribution of the analysed minutellin-derivatives detected in the here studied specimen of *Hypoxylon* and *Annulohypoxylon*. Table 5 summarises the distribution of the analysed minutellin-derivatives detected in the here studied specimen of *Hypoxylon* and *Annulohypoxylon* and Figure 4 shows the structures of all discussed compounds in this section.

**Table 5.** Minutellins E–I, A, C, and D (**1**–**5**, **11**, **12**, **6**) detected in *Annulohypoxylon michelianum*, *Hypoxylon lateripigmentum*, and *H. pulicicidum* stromatal extracts.

Specimen	Species	1	2	3	4	5	6	11	12
KR-M-0002761	*A. michelianum*	**+**	–	**+**	–	**+**	**+**	–	–
GUM 1594	*H. lateripigmentum*	**+**	**+**	**+**	**+**	–	**+**	–	–
GUM 1595	*H. lateripigmentum*	**+**	**+**	**+**	**+**	–	**+**	–	–
GUM 1596	*H. lateripigmentum*	**+**	**+**	**+**	**+**	–	**+**	–	–
MJF10046	*H. lateripigmentum*	**+**	**+**	**+**	**+**	–	**+**	–	–
MJF07147	*H. pulicicidum*	–	–	–	–	–	**+**	**+**	**+**

### 3.7. Taxonomy

**Figure 5.**
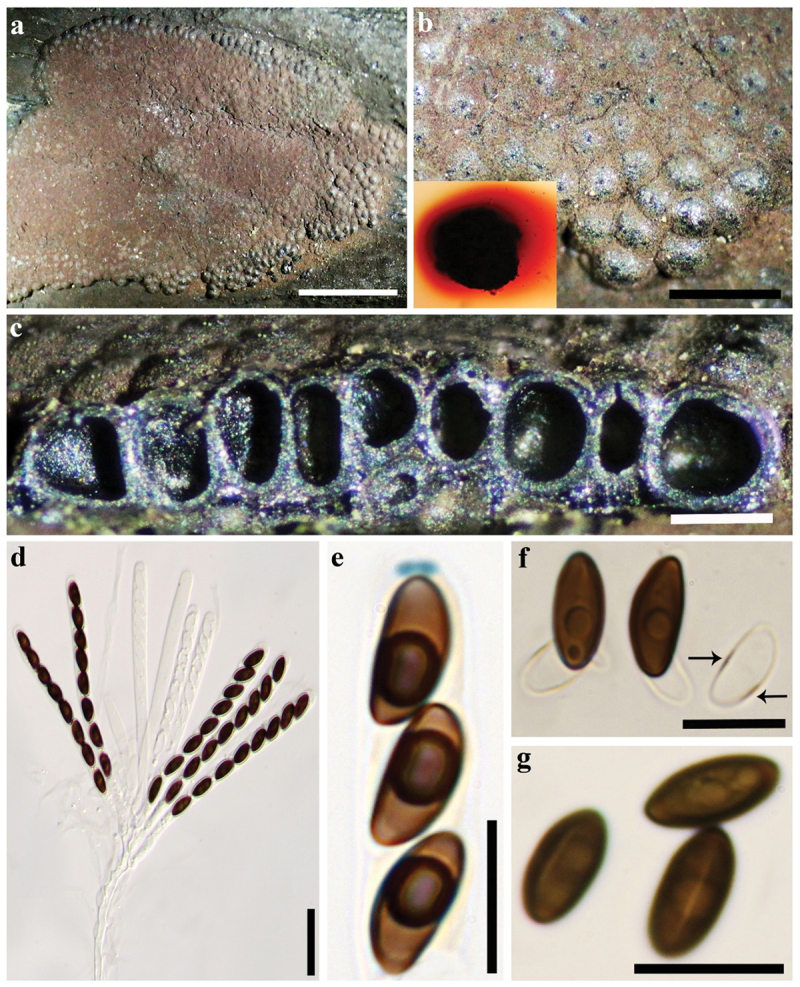
*Hypoxylon lateripigmentum* (GUM 1594). (a, b) Close-up view of stromatal surface, with stromatal pigments in 10% KOH. (c) Stroma in vertical section showing perithecia and ostioles. (d) Immature and mature asci in water. (e) Mature ascus tip in Melzer’s reagent. (f) Ascospores in 10% KOH with dehiscent perispore with thickened (black arrow). (g) Ascospore in water, with straight germ-slit. Scale bars: a = 5 mm, b = 1 mm, c = 0.5 mm, d = 20 µm, e – g = 10 µm.

**Figure 6.**
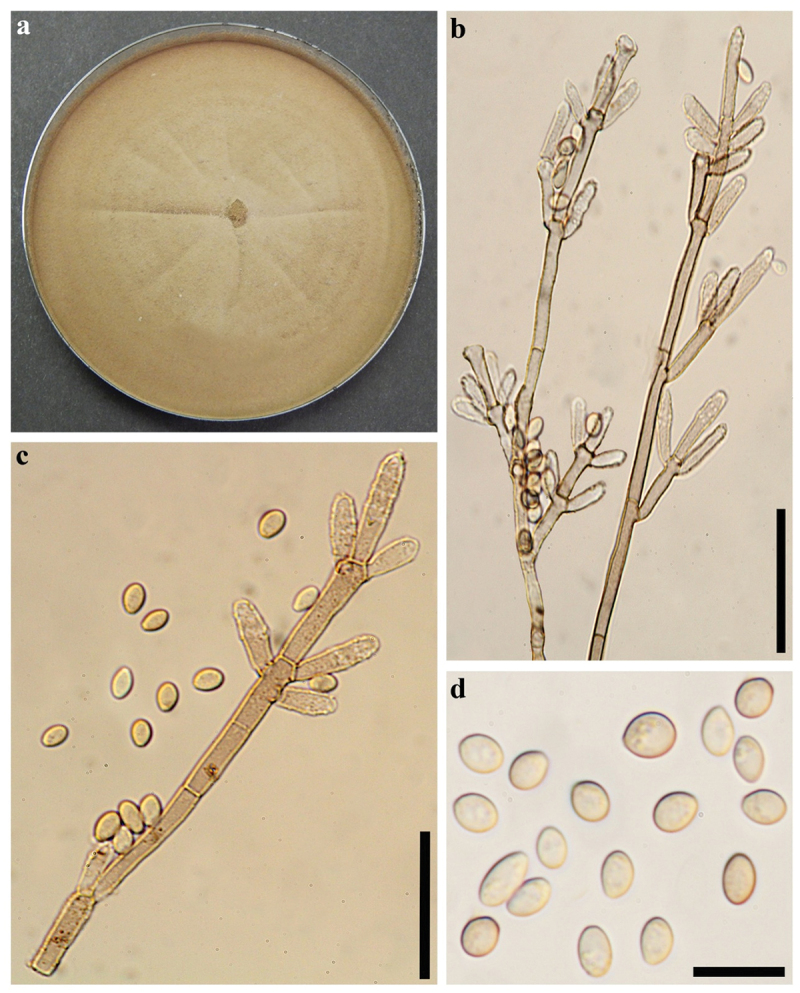
Culture and anamorphic structures of *Hypoxylon lateripigmentum* (MUCL 57716) on OA. (a) Surface of colony after 6 weeks of incubation. (b, c) General view of anamorph structure with periconiella-like branching patterns, conidiogenous cells, immature and mature conidia. (d) Conidia. Scale bars: b, c = 20 µm, d = 10 µm.

***Hypoxylon lateripigmentum*** J. Fourn., Kuhnert & M. Stadler, Fungal Diversity 64: 192 (2014). Figures 5 and 6.

**Teleomorph**. Stromata superficial, effused-pulvinate, up to 2 cm long × 0.6–1.1 cm wide, surface Vinaceaous (57) to Dark Brick (60) becoming black with conspicuous perithecial mounds; Brown Vinaceaus (84) granules beneath the surface and between the perithecia, with KOH-extractable pigments Red (2) to Scarlet (5). Perithecia obovoid to tubular, 0.36–0.43 mm high × 0.23–0.35 mm wide. Ostioles umbilicate, inconspicuous. Asci 8-spored, cylindrical, with amyloid, discoid apical apparatus, 0.5–0.75 µm high × 1.5–2 µm wide, stipe up to 190 µm, and spore-bearing portion 60–85 × 4.5–6 µm. Ascospores smooth, unicellular, brown to dark brown, ellipsoid, inequilateral to slightly equilateral with narrowly rounded ends, 8–12(−13) × 3.5–5 µm, with straight to rarely sigmoid germ slit spore-length on convex side; perispore dehiscent in 10% KOH, smooth with a thickening on one or both side; epispore smooth.

**Cultures and anamorph**. Colonies on OA covering a 9 cm Petri dish in 2 wk, at first white, becoming pale brown, felty, zonate with diffuse margins; reverse becoming Hazel (88). Conidiogenous structure approaching a periconiella-like branching pattern as defined by Ju and Rogers (1996), (Figure 6b,c). Conidiophores hyaline, smooth to finely roughed. Up to three conidiogenous cells at the end of each terminus; hyaline, smooth to finely roughened, 11–16 × 2–3.5 µm. Conidia hyaline, smooth, ellipsoid to obovoid, 4–7 × 2–4 µm.

**Secondary metabolites**. New minutellin-type azaphilones minutellin E–H and minutellin D.

**Specimens examined**. Iran, Guilan Province, Shaft County, Rahimabad (Sefidab) forest, 36°34’10.94“N, 50°20’26”E, 400 m elev., on fallen branch of *Parrotia persica*, 21 September 2016, leg. M.J. Pourmoghaddam (GUM 1594; living culture MUCL 57716); Guilan Province, Shaft County, Rahimabad (Sefidab) forest, 36°34’10.94“N, 50°20’26”E, 400 m elev., on fallen branch of *Pterocarya fraxinifolia*, 21 September 2016, leg. M.J. Pourmoghaddam (GUM 1595; living culture MUCL 57717); Guilan Province, Rezvanshahr County, 37°37’52“N, 40°02’18”E, 7 m elev., on fallen branch of *Gleditschia caspica*, 6 October 2016, leg. M.J. Pourmoghaddam (GUM 1596; living culture MUCL 57718).

**Note**. These specimens show high similarities to *H. lateripigmentum* in teleomorphic and anamorphic appearance. Nonetheless, Iranian specimens have variations in the size of perithecia (0.36–0.43 × 0.23–0.35 vs. 0.6–0.7 × 0.35–0.5 mm) and ascospores (8–13 × 3.5–5 vs. 7.7–9.5 × 3.8–4.2 µm). A comparison of Iranian sequence data revealed differences between the three Iranian strains of *H. lateripigmentum* (MUCL 57716/MUCL 57717/MUCL 57718) and the ex-type strain of *H. lateripigmentum* [19/8/19 bp differences of 432 nucleotide characters in the ITS: 6/6/6 substitutions, 13/2/13 indels; 6/6/6 bp differences (substitutions) of 1,249 nucleotide characters in the LSU; 11/11/11 bp differences (substitutions) of 942 nucleotide characters in the *rpb2*; and 36/43/36 bp differences of 1,216 nucleotide characters in the *tub2*: 32/37/33 substitutions, 4/6/3 indels]. Nonetheless, chemotaxonomic analysis showed virtually identical chemotypes when compared with an ex-holotype-derived acetone extract, leading us to assign these specimens to *H. lateripigmentum*. This ought to be changed when a larger number of isolates becomes available to further delineate potential differences in population studies. *Hypoxylon lignicola* and *H. phuphaphetense* have recently been published according to asexual morphs and there is no evidence of a sexual morph in the latter species (Luo et al. 2019). Table 6 compares morphological characters of some other taxa that may be confused with this species.

**Table 6.** Diagnostic characters of *Hypoxylon lateripigmentum* and closely related species.

Taxon	Stromatal shape	Stromatal surface	KOH extractable pigments	Ascospores (µm)	Germ slit	Perispore in KOH	Anamorph		Secondary metabolites
*Hypoxylon croceum*	Glomerate to pulvinate	Fulvous when young, Sepia or Fuscous when aged	Isabelline or Hazel	8.5–12(−13.5) × 4–5	Straight less than spore-length	Indehiscent	Virgariella-like	Unknown minutellin-type azaphilones	
*Hypoxylon hinnuleum*	Glomerate to pulvinate or effused-pulvinate	Dark Vinaceous to Sepia, becoming Black	Dark Brick	(7.2–)7.6–9.5(−11.7) × (2.8–)3.7–4.5(−4.8)	Straight, 2/3 spore length	Indehiscent	Virgariella-like, nodulisporium-like, and periconiella-like	BNT, minutellin D, various unknown cohaerins	
*Hypoxylon investiens*	Effused-pulvinate	Brown Vinaceous, Sepia, Dark Brick, or Chestnut	Greenish Yellow, Dull Green, or Dark Green	6.5–9.5(−10) × 3–4.5	Straight less than to spore-length	Indehiscent	Periconiella-like	BNT, daldinone A, 5-methylmellein	
*Hypoxylon lateripigmentum*	Effused-applanate	Dark Vinaceous	Sienna to Bay	7.7–9.5 × 3.8–4.2	Straight spore-length	Dehiscent	Periconiella-like	BNT, new minutellins **1**–**4** and minutellin D(**6**), 1–8-naphthol	
**Iranian *Hypoxylon lateripigmentum* specimens described here**	**Effused-pulvinate**	**Vinaceaous to Dark Brick, becoming black**	**Red to Scarlet**	**8–12(−13) × 3.5–5**	**Straight to rarely sigmoid spore-length**	**Dehiscent**	**Periconiella-like**	**1–4 and minutellin D (6)**	
*Hypoxylon olivaceopigmentum*	Glomerate to pulvinate or effused-pulvinate	Brown Vinaceous, becoming black	Olivaceous	(9.3–)9.7–11.9(−15.7) × (4.5–) 5.0–6.6(−7.2)	Straight 1/2 to 2/3 of spore length	Indehiscent	Periconiella-like	BNT, an unknown cohaerin-type compound with m/z = 498	
*Hypoxylon pulicicidum*	Irregularly effused-pulvinate to elongate	Dark Vinaceous to Brown Vinaceous	Olivaceous Buff	7.5–9.4 × 3.2–4.2	Straight less than to spore-length	Indehiscent	Nodulisporium-like	BNT, minutellins A, C, and D	

Specimens are in bold described in present study.

**Figure 7.**
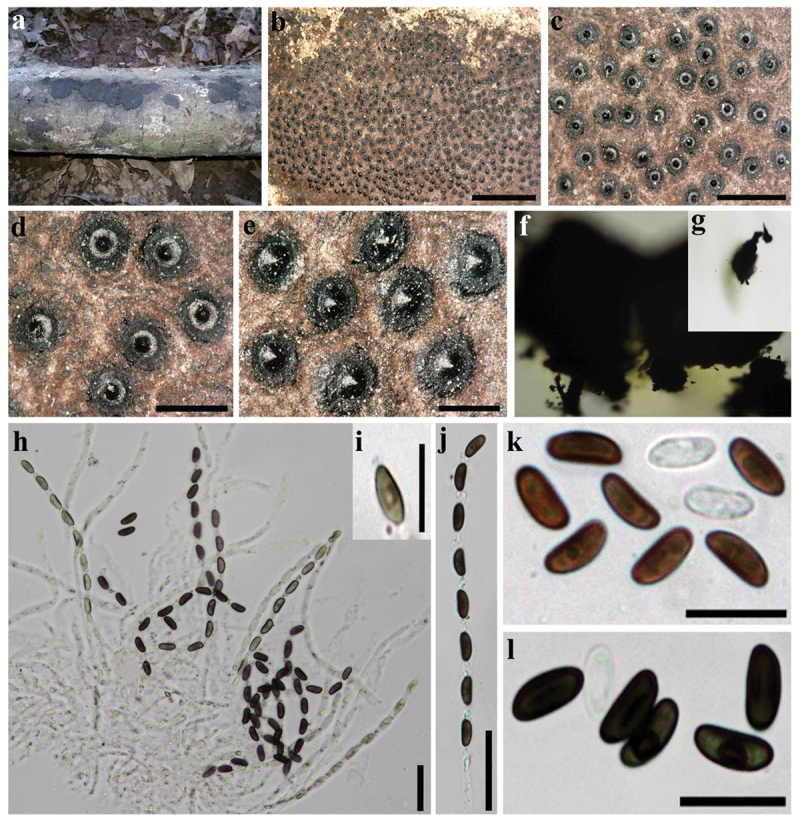
*Annulohypoxylon substygium* (GUM 1618). (a) Stromatal habit. (b, c) Close-up view of stromatal surface. (d, e) Close-up view on ostiolar discs. (f, g) Stromatal pigments in 10% KOH. (h) Immature and mature asci in water. (i) Ascus tip in Melzer’s reagent. (j) Mature ascus in water. (k, l) Ascospores in 10% KOH with dehiscent perispore showing thickening. Scale bars: b = 4 mm, c = 1 mm, d = 0.5 mm, e = 0.3 mm, h, j = 20 µm, i, k, l = 10 µm.

***Annulohypoxylon substygium*** Sir & Kuhnert, Fungal Diversity 85: 20 (2017). Figure 7

**Teleomorph**. Stromata superficial, effused-pulvinate; up to 20 cm long × 0.2–3 cm wide; with inconspicuous perithecial mounds; surface Vinaceous Gray (116) when young, becoming blackish with Brown Vinaceous (84) to Fawn (87), black when old, pruinose; blackish granules immediately beneath surface; with KOH-extractable pigments Greenish Yellow (16) or Fawn (87). Perithecia spherical to ovoid 0.3–0.65 mm high × 0.25–0.6 mm wide; ostioles conical papillate, encircled with a bovei-type disc 0.25–0.35 mm diam. Asci 8-spored, cylindrical, with weakly amyloid, discoid apical apparatus, 0.5 µm high × 1–1.5 µm wide, stipe up to 50 µm, and spore-bearing portion 65–75 × 4–5 µm. Ascospores light brown to brown, unicellular, ellipsoid nearly equilateral, with broadly rounded ends, 6.0–9.0(−10) × 2.5–3.5 µm, with straight germ slit spore-length on the more flattened side; perispore dehiscent in 10% KOH, smooth with a thickening on the more flattened side; epispore smooth.

**Cultures and anamorph**. Colonies on OA covering a 9 cm Petri dish in 2 wk, at first whitish, becoming Pale Green (19) in centre, Grey Olivaceous (107) and Flesh (37), whitish in margins, concentric zones, velvety to felty, with entire margins; finally, Isabelline (65) to Dark Brick (60). Conidiogenous structure branching periconiella-like as defined by Ju and Rogers (1996). Conidiophores brown to pale brown, smooth to finely roughened. Conidiogenous cell brown to pale brown, smooth to finely roughened, 12–17 × 2–3 µm. Conidia cylindrical to ellipsoid, hyaline to pale brown, smooth to finely roughened, 5–7 × 2–3 µm.

**Secondary metabolites**. BNT, daldinone A, daldinone F, the new daldinone L.

**Specimens examined**. Iran, Guilan Province, Lahijan County, Kuhbijar forest, 37°11ʹ26”N, 50°01ʹ46”E, 350 m elev., on fallen branch of *Acer* sp., 20 September 2016, leg. M.J. Pourmoghaddam (GUM 1618; living culture MUCL 57734); Guilan Province, Fouman County, Ghalerodkhan forest, 37°04ʹ05.02”N, 49°14ʹ40.08”E, 500 m elev., on fallen branch of *Quercus castaneifolia*, 29 October 2016, leg. M.J. Pourmoghaddam (GUM 1624; living culture IRAN 3727C); Guilan Province, Siahkal County, Deilaman forest, 36°57’25“N, 49°51’54”E, 1,100 m elev., on fallen branch of *Quercus castaneifolia*, 3 October 2016, leg. M.J. Pourmoghaddam (GUM 1619; living culture IRAN 3728C); Guilan Province, Rasht County, Saravan forest, 37°04’26“N, 49°38’13”E, 183 m elev., on fallen branch of *Quercus castaneifolia*, 3 October 2016, leg. M.J. Pourmoghaddam (GUM 1620); Guilan Province, Siahkal County, Ziaratgah forest, 37°08ʹ07”N, 49°55ʹ35”E, 263 m elev., on fallen branch of *Quercus castaneifolia*, 3 October 2016, leg. M.J. Pourmoghaddam (GUM 1622).

**Note**. *Annulohypoxylon substygium* was hitherto only reported from the northern hemisphere and the Neotropics (e.g. Argentina, France, Georgia, La Réunion) (Kuhnert et al. 2017a). Most of the characters of the Iranian specimens are in accordance with previous descriptions (Kuhnert et al. 2017a), aside from insignificant variations in the size of ascospores (6.0–10.0 × 2.5–3.5 vs. 6.5–8.5 × 2.8–3.5 µm) and in the colour of the KOH-extractable pigments (greenish yellow or fawn vs diluted fuscous black to fawn). A comparison of Iranian sequence data revealed similarities between the six Iranian strains of *A. substygium* (MUCL 57734/IRAN 3727C/IRAN 3728C/318/465/485) and the epi-type strain (MUCL 51708) [6/6/6/6/6/6 bp differences (indels) of 627 nucleotide characters in the ITS; and no differences of 1,346 nucleotide characters in the *tub2*]. We also included the newly sequenced LSU and *rpb2* loci of this species for the first time. This species can be differentiated from *A. stygium* by morphological characters (larger ostiolar discs, larger perithecia, and KOH-extractable pigments), stromatal secondary metabolites (chemotaxonomy), and molecular phylogeny.

## 4. Discussion

In this study, we report on the identification and polyphasic characterisation of a collection of Iranian *Annulohypoxylon* and *Hypoxylon* spp. from near the Caspian Sea, and on a chemotaxonomic isolation campaign including both the newly collected specimens and a previously reported specimen of *A. michelianum* (Kuhnert et al. 2017a). A collection of Iranian *Annulohypoxylon* could unequivocally be assigned to *A. substygium*. The other specimen showed affinities to *Hypoxylon lateripigmentum*, but showed differences in the size of perithecia and ascospores (Table 6). Chemotaxonomic analysis revealed virtually identical chemotypes when compared with a holotype-derived acetone extract, which settled its identity as *H. lateripigmentum*.

During species identification, after thorough comparison with internal databases, featuring UV/Vis absorption maxima, retention time, and accurate mass of authentic standards (also called dereplication; see Bitzer et al. 2007), we noted the presence of several unidentifiable compounds. Concurrently, while examining the stromatal constituents of *A. michelianum*, we detected similar compounds, and decided to integrate these efforts into the current study. Subsequently, we isolated and herewith describe new azaphilones of the minutellin/cohaerin family from the stroma of *A. michelianum* and *H. lateripigmentum*. Structural key difference of the new minutellins **1**–**5** to their most closely known azaphilones relatives is the decanoyl fatty acid side chain moiety, in contrast to i.e. the 2-methyloctyl or the 2-methybutyl side chain of the cohaerins and multiformins, respectively (Surup et al. 2013; Jansen-Olliges et al. 2022). However, the decanoyl fatty acid side chain is also part of biscogniazaphilones A and B, which have been isolated from the endophytic fungus *Biscogniauxia formosana* (Cheng et al. 2012). Structural difference of **1**–**6** to them is the cyclohexyl moiety attached to C–3, in contrast to the propenyl residue of biscogniazaphilones A and B. Moreover, analogous variations of this cyclohexanyl ring structure with various patterns of hydrogenation or elimination/dehydration have been observed for cohaerins and multiformins, too.

Secondary metabolite production patterns, or chemotypes, have served as useful information for chemotaxonomic purposes and have also been shown to be specific for different lineages in the Hypoxylaceae. Orsellenic-acid derived mitorubrin-derivatives and rubiginosins, for instance, are widespread in the *Hypoxylon rubiginosum* complex (Stadler et al. 2008), and the *H. fuscum* complex features species producing BNT, daldinin derivatives, and other undescribed naphthalenes (Quang et al. 2004; Lambert et al. 2021). Moreover, members of *Annulohypoxylon* regularly include species that additionally contain truncatone-type derivatives, which are typically absent in its sister genus *Jackrogersella* (Kuhnert et al. 2017a; Wendt et al. 2018). The latter was even segregated from *Annulohypoxylon* by Wendt et al. (2018) based on phylogenetic and chemotaxonomic evidence, i.e. the presence of minutellin/cohaerin-type azaphilones, such as minutellins A–D reported from *J. minutella* (Kuhnert et al. 2017b) or cohaerins A–K from *J. cohaerens* (Quang et al. 2005, 2006; Surup et al. 2013). Occasionally however, chemotype and phylogenetic placement may differ. Recently, we proposed – based on comparisons with high-resolution HPLC-MS data – that the stroma of *H. dussii* contains mitorubrin-type derivatives (Cedeño-Sanchez et al. 2025). Concurrently, we sequenced an ex-type culture of this species, which then clustered in a phylogenetic lineage with *H. investiens*, *H. pulicicidum* and allies, in which these derivatives are absent. However, *H. investiens* was shown to contain naphthalene-type compounds, such as BNT and daldinone A usually encountered in *Daldinia*, or *Annulohypoxylon* (Stadler et al. 2014; Kuhnert et al. 2017a; Becker and Stadler 2021). Another interesting case is *Annulohypoxylon michelianum*, which had been shown to contain minutellin A, C, and D, identified based on a comparative approach (Kuhnert et al. 2017a). Morphologically, it shows typical characteristics of *Annulohypoxylon* and resolves in a basal position to other *Annulohypoxylon*. Together with the chemotaxonomic evidence, this taxon shows a rather intermediate phenotype between *Annulohypoxylon* and *Jackrogersella* (Wendt et al. 2018). It is currently not clear how the production of minutellin/cohaerin-type azaphilones has evolved and why this trait is missing in other *Annulohypoxylon* and *Hypoxylon* spp. phylogenetically related to *H. investiens*. A possible explanation could be the loss of secondary metabolite production capability of a former ancestor of the remaining *Annulohypoxylon* and a lineage that later evolved into *H. investiens* and related species. However, low bootstrap support, potentially due to a conflicting, underlying phylogenetic signal, for the final placement of *H. investiens* prevents any solid hypothesis associated with this phenomenon. It will be interesting in the future to study the recently sequenced genomes in detail to put these potential conflicts of phylogenetic signal into context, unravel evolutionary patterns of the secondary metabolism and further explore the evolutionary history of this clade.

Here, we report the results of an analysis and characterisation of stromatal constituents from environmental samples due to access to sufficient amounts for preparative work, but this is not always the case. In instances where stroma is scarce (or valuable types are studied), it is possible to assign compounds directly by comparison with a co-analysed authentic sample of a given natural product. This was, for example, done for stroma extracts derived from *H. aeruginosum, Chlorostroma subcubisporum*, and *C. cyaninum*, which were compared to lepraric acid derivatives (Læssøe et al. 2010). Unidentified, similar compounds to the ones reported here were previously reported from stromatal extracts of the predominantly endophytic *H. pulicicidum* (Bills et al. 2012). A comparison of the holotype with our compounds yielded identical UV/Vis and mass patterns, which lead us to the assignment of three distinct peaks to compounds **6**, **11**, and **12**. Similar compounds had also been detected in *H. croceum*, *H. hinnuleum*, *H. olivaceopigmentum* (Sir et al. 2019) and *Parahypoxylon* (Cedeño-Sanchez et al. 2023), which can, in principle, be identified following a similar approach.

In a previous study, we tentatively assigned stromatal compounds of *Parahypoxylon papillatum* and *P. ruwenzoriense* to the minutellin azaphilone family based on molecular networking, which even suggested the presence of many other, not identified and never isolated derivatives (Cedeño-Sanchez et al. 2025). However, whether these compounds are indeed representing stable and isolatable compounds or merely represent degradation or extraction artefacts or instable intermediates is an open question. A full study based on stromatal material is unrealistic due to potentially requiring multi-gram scale of stroma material, but since candidate biosynthetic gene clusters are available (Kuhnert et al. 2021), the biosynthetic machinery can in future be studied complementarily by heterologous gene expression. Curiously, a recent study reported on the production of similar compounds from the new species *Tengochaeta bulbillosa*, accommodated in the Chaetomiaceae in culture, which should be an interesting candidate to further study the chemical space of these interesting azaphilones (Barrera-Adame et al. 2025). Until then, fresh specimens need to be collected to isolate and unambiguously assign the identity of individual compounds for chemotaxonomic purposes.

## Supplementary Material

Supplemental Material
