# Supplementary material for: Minutellins E-I and daldinone L, new secondary metabolites from different species of the Hypoxylaceae (Xylariales, Ascomycota)
Source: Mycology. 2025 Jun 18;17(1):2512962. doi: 10.1080/21501203.2025.2512962 (PMC13007406; doi:10.1080/21501203.2025.2512962)
Supplement: Supplemental Material [file TMYC_A_2512962_SM8722.pdf]

Supplementary information for

Minutellins E-I and daldinone L, new secondary metabolites from different species of the Hypoxylaceae (Xylariales, Ascomycota)

Christopher Lambert[a,b,c,d], Mohammad Javad Pourmoghaddam[e], Esteban Charria-Giron[a,c,d], Frank Surup[a,c,d], Seyed Akbar Khodaparast[e], Hermann Voglmayr[f], Irmgard Krisai-Greilhuber[f] and Marc Stadler*[a,c,d]

[a]*Microbial Drugs, Helmholtz-Centre for Infection Research GmbH, Braunschweig, Germany;* [b]*Cell Biology, Helmholtz-Centre for Infection Research GmbH, Braunschweig, Germany;* [c]*Institute of Microbiology, Technical University Carolo-Wilhelmina Braunschweig, Braunschweig, Germany;* [d]*German Centre for Infection Research (DZIF), Partner Site Hannover-Braunschweig, Braunschweig, Germany;* [e]*Department of Plant Protection, Faculty of Agricultural Sciences, University of Guilan, Rasht, Iran;* [f]*Department of Botany and Biodiversity Research, University of Vienna, Rennweg 14, 1030 Wien, Austria*

* Correspondence to Prof. Marc Stadler: marc.stadler@helmholtz-hzi.de

Table S1. Multigene alignment used fort he phylogenetic study
>Biscogniauxia_nummularia

-CATTAGCGAGTTAA-ACAAACTCCAAACCCATGTGAACATACCTACTGTTGCCTCGGCA---
GGTGTGCGTGCGGGGTGCCTACCCTGTAGGAACCTACCCTGTAGTGACCTACCCTGGAGCTACCCT
GC-AGCACGCAACCAGGCCTGCCAAAGGACCCCTAAACTCTGTT----
ATAACGTACCTCTGAGTCAACTATACAAATAAGTTAAAACTTTCAACAACGGATCTCTTGGTTCTG
GCATCGATGAAGAACGCAGCGAAATGCGATAAGTAATGTGAATTGCAGAATTCAGTGAATCATCG
AATCTTTGAACGCACATTGCGCCTAATAGTATTCTGTTAGGCATGCCTGTTCGAGCGTCATTTCAAC
CCCCAAGGCTATTTAGCTTGACGTTGGGAATTTACAGCTG----
TGTAATTCCTCAAATTTAGTGGCGGAGCTAGGTCATGCTCTGAGCGTAGTAATTGTTCTCGCTCCTG
AAGCTGCCCTATAT-CCTGCCGTAAAACCCCT----
TATAATCTGGTTGACCTCGGATCAGGTAGGAATACCCGCTGAACTTAAGCATATGAAGCGGCAACA
GCTCAAA-TTTGAAATCTGGCC----TTC-----G-
GGTCCGAGTTGTAATTTGCAGAGGATGCTTTTGGCGAGGT-
GCCTTCCGAGTTCCCTGGAACGGGACGCCTTAGAGGGTGAGAGCCCCGTACGGTTGGA-
CACCGAGCCTCTGTAAAGCTCCTTCGACGAGTCGAGTAGTTTGGGAATGCTGCTCTAAATGGGAGG
TAAATTTCTTCTAAAGCTAAATACCGGCCAGAGACC-
GATAGCGCACAAGTAGAGTGATCGAAAGATGAAAAGCACTTTGAAAAGAGGGTTAAATAGCACGT
GAAATTGTTGAAAGGGAAGCG-TTTACGGCCAGACCTTCTCCTGGCGGATCATCCGGTGTTC--
TCACCGGTGCACTTCGCCAGG--TTTAGGCCAGCATCGGCTTC---
TGTAGGGGGATAAAAGCAGTGGGAAAGTAGCTC----TCTCGGGAGTGTT-
ATAGCCCTAAGCATAATACCTTTAC-GGGGGCCGAGGACCGCGCT-TCG---
GCAAGGATGCTGGCGTAATGGTCGTCAACGACCCGTCTTGAAACACGGACCAAGGAGTCGAACAT
TTGTGCGAGTGTTTGGGTG--

TTAAACCCTCACGCGTAATGAAAGTGAACGGAGGTGAGAGCCCTCT---AGG---
GGGTGCATCATCGACCGATCCTGATG-
TCTTCGGATGGATTTGAGTAGGAGCATTAATGTTCGGACCCGAAAGATGGTGAACTATGCGTGGAT
AGGGTGAAGCCAGAGGAAACTCTGGTGGAGGCTCGCAGCGGTTCTGACGTGCAAATCGATCGTCA
AATCTGCGCATGGGGGCGAAAGACTTATCGA-
ACCATCTAGTAGCTGGTTACCGCCGAAGTTTCCCTCAGGATAGCAGTGT--TG-
TATTCAGTTTTATGAGGTAAAGCGAATGATTAGGGACTCGGGGGCGCTTTTTAGCCTTCATCCATTC
TCAAACTTTAAATATGTAAGAAGCCCTTGTTACTTAATTGAACGTGGGCATTCGAATGTACCAACA
CTAGTGGGCCATTTTTGGTAAGCAGAACTGGCGATGCGGGATGAACCGAACGCGGGGTTAAGGTG
CCGGAGTGGACGCTCATCAGACACCACAAAAGGTGTTAGTACATCTAGACAGCAGGACGGTGGCC
ATGGAAGTCGGAATCCGCTAAGGACTGTGTAACAACTCACCTGCCGAATGTACTAGCCCTGAAAAT
GGATGGCGCTCAAGCGTCC-CACCCATACCTCGCCCTCAGGGTAGAAACGATGCCCTGAGG-----------

----

GATTCACCGATACACAT------TTTCCTCTA---
CCCTCTCTCATTTGAGACGTACGAACACTCCTGTCGGAAGAGACGGCAAGCTCGCCAAGCCGCGAC
AACTGCACAATACTCATTGGGGTCTTGTCTGTCCTGCGGAGACGCCCGAAGGTCAAGCTTGTGGGT
TGGTGAAGAACTTGTCACTGATGTGCTCTATCAGCGTGGGTACGTCGACGGAGCCAATCATTGATT
ACATGATTACTCGAAACATGGAAGTGCTTGAAGAATACGAGCCCATGCGATATCCTCACGCTACCA
AGATCTTCCTCAACGGCTCTTGGATCGGTGTTCACCAGGACCCGAAGGCACTTGTTAGGGATGTCC
AGCAACTGCGTCGGAGCAATCAAATTCCCGCGGAGGTGTCCCTGGTTCGAGACATCAGGGACCGC
GAGTTCAAGATTTTCTCAGATGCTGGTCGAGTCATGCGGCCTTTGTTCGTTGTCGAGCAGGAAGAT--

----

ATGCCTGACACCGGTGTCGAAAAGGGTTCGCTAGCTCTCAACAAGAACATGGTGAGAAGGTTAGA
GCTTGACCAAACGCTACCTCCTGGAAGCGATGAGTACTATGGCTGGGAAGGTTTGGTGGGCGATGG
TGTCATCGAATATCTCGATGCGGAAGAGGAAGAGACGGCAATGATATGCATGACACCGGAGGATC
TCGAAATCTTTCGGATGGTCAAGCTTGGTCACGAGATGGTGAATGATAACGGG------
GACGATCTGAATAAGCGACTCAAGACAAAGATAAATCCAACGACCCATATGTACACGCATTGCGA
AATCCACCCCAGTATGCTGCTCGGTATCTGCGCCAGCATTATCCCATTCCCCGATCATAACCAGGTA
TCCCTGAACGCGTCC---AAACCCCTGACTTGCTACCCCTCACAAACCGGCAAC------ACAACAC-
TCTCTCCTGGCGATATAGCACTATCCTGATACAAAGCCAC-
GTATTGATGGGCATCGCAGCTAACCGCGTCTTTCTTCTTTTATAGGTTCACCTTCAAACCGGCCAAT
GCGTAAGTAAATACCC----
GACAACAATAGACAACAAAGCGCGCACAATTATTGCGATACTCACATA--
TTACGTAGGGTAACCAAATTGGTGCTGCCTTCTGGCAAACCATCTCCGGCGAGCACGGTCTCGACA
GCAATGGCGTGTATGTGTACTTCCCCCCTATTCACACGGCAAGCATCAAAACTGACCGGCTATCAA
CAGTTACAACGGAACGTCTGAGCTCCAATTGGAGCGGATGAGTGTGTACTTCAACGAGGTAATGTG
GTCGGATCATCGGACTTTGGC----
ACCTCTTGTGCCTAATCTAACCGTGGTGTGCAGGCCTCCGGCAACAAGTATGTCCCTCGTGCCGTCC
TGGTCGATCTCGAGCCCGGTACCATGGACGCCGTCCGCGCCGGCCCCTTCGGTCAGCTCTTCCGCCC
CGACAACTTCGTCTTCGGTCAGTCCGGTGCTGGCAACAACTGGGCCAAGGGTCATTACACTGAGGG
TGCCGAGCTGGTTGACCAGGTTCTCGATGTTGTCCGTCGCGAGGCTGAGGGTTGTGACTGCCTCCA
GGGCTTCCAGATCACCCACTCTCTCGGTGGTGGTACTGGTGCTGGTATGGGTACCCTGTTGATCTCC
AAGATCCGCGAGGAGTTCCCCGACCGAATGATGGCCACCTTCTCCGTCGTTCCCTCTCCCAAGGTCT
CCGACACCGTCGTCGAGCCCTACAACGCCACCCTTTCCGTCCACCAGCTGGTCGAGAACTCGGACG
AGACTTTCTGCATTGACAACGAGGCTCTGTACGACATCTGCATGCGTACCCTGAAGCTATCCAACC
CCTCGTACGGCGACCTGAACCACCTGGTTTCTGCCGTCATGTCTGGTGTCACCACCTGTCTACGTTT
CCCTGGTCAGCTCAACTCTGACCTCCGAAAGCTGGCCGTCAACATGGTGCCCTTCCCTCGTCTGCAC
TTCTTCATGGTCGGCTTCGCTCCTCTTACCAGCCGTGGTGCTCACTCTTTCCGCGCTGTCACCGTTCC
CGAGTTGACCCAGCAGATGTTCGACCCCAAGAACATGATGGCTGCCTCTGACTTCCGCAACGGTCG
CTACCTGACGTGCTCCGCCATCTTGTAAGATTTTCCGGCCTGGTC---
TTTTGGTGTCTACCTGCTAACTTTTTCCTTCTAGCCGTGGCAAGGTCTCGA

>Graphostroma_platystomum

-CATTAGCGAGTTAA-ACAACTCCAAAACCCATGTGAACATACCTATCGTTGCCTCGGCA---
GGCGCGCTCG---GGCGGTTACCCTGAAGGAG-CTACCCTGTAGCTACCCTATAGGAGCTACCCTCGT-
AAGCTGCTCTAAAGCCTGCCGGCGGACCCCTAAACTCTGA---
ATTTACTGTATCTCTGAGTGTAAAACAAAAATAAGTTAAAACTTTCAACAACGGATCTCTTGGTTCT

GGCATCGATGAAGAACGCAGCGAAATGCGATAAGTAATGTGAATTGCAGAATTCAGTGAATCATC
GAATCTTTGAACGCACATTGCGCCTAATAGTATTCTGTTAGGCATGCCTGTTCGAGCGTCATTTCGA
CCATTAAGCCCTGTTTGCTTAGCGTTGGGAACTTACGCCTG----
CGTAATTCCTTAAATTCAGTGGCGGAGCTAGGTCATGCTCTAAGCGTAGTAATTTTCCTCGCTTCTG
TAGCTGGCCTATAT-CCTG---
TGAAGCGGCAACAGCTCAAA-TTTGAAATCTGGCT----CTA-----G-
GGTCCGAATTGTAATTCGTAGAGGATGCTTTTGGCGCGGT-
CACTTCCGAGTTCCCTGGAACGGGACGCCTTAGAGGGTGAGAGCCCCGTACGGTTGGA-
TANCAAGCCTCTGTAAAGCTCCTTCGACGAGTCGAGTAGTTCGGGAATGCTGCTCTAAATGGGAGG
TAANTTTCTTCTNAAGCTAAATNCCGGCCAGAGNCCNGATAGCGCACAAGTAGAGTGATCGAAAG
ATGAAAAGCACTTTGAANAGAGGGTTAAATAGCACGTGAAATTGNTGAAAGGGAAGCGNTTTNCG
GNCAGACCTTTTCCCAGCGGNTCATNCAGTGNTT-CTCACTGGTGCACTCTGCTGGG--
TTTAGNCCAGCATCGGCTTC---TGTAGGGGGATAAAAGCCNNGGGAAAGTAGNTC----
CCTCGGGANTGTTNATNNNNCTAGGCNTNANNCNNTNNC-GGGGGCCGAGGNNCGCNCT-CT----
GCAAGGANGCTGGCGTAATGGTCGTCANCGACCCGTCTTGAAACACGGACCANGGAGTCGAACAT
TTGTGCGAGTGTTTGGNTG--TTAAACCCTCACGCGTAATGAAAGTGAACGGAGGTGAGAGCCCTTT-
--ACG---GGGTGCATCATCGACCGATCCTGATG-
TCTTNGGATGGATTTGAGTAGGAGCATTAATGTTCGGACCCGAAAGATGGTGANCTATGCGTGGAT
AGGGTGAAGCCAGAGGAAACTCTGGTGGAGGCTCGCAGCGGNTCTGACGTGCAAATCGATCGTCA
AATCNGCGCATGGGGGCGAAAGACTTATCGA-
ACCATCTAGTAGCTGGTTACCGCCGAAGTTTCCCTCAGGATAGCAGTGT--
TGTTCTTCAGTTTTATGAGGTAAAGCGAATGATTAGGGACTCGGGGGCGCTTATTAGCCTTCATCCA
TTCTCAAACTTTAAATATGTAAGAAGCCCTTGTTGCTTAGTTGAACGTGGGCATTCGAATGTACCAA
CACTAGTGGGCCATTTTTGGTAAGCAGAACTGNCGATGCGGGATGAACCGAACGCGGGGTTAAGG
TGCCGGAGTGGACGCTCATCAGACACCACAAAAGGTGTTAATACATCCAGACAGCCGGACGGTGG
CCATGGAAGTCGGAATCCGCTAAGGACTGTGTAACAACTCACCGGCCGAATGTATTAGCCCTGAAA
ATGGATGNNGCTCAAGCGTCC-
CACCCATACCTCGNCCTCAGGGNAGAAACGATGCCCTGAGGGCGGAGTATCGAACAGAACCGGGG
TTTCGCTATCGAGCTAGCTGTGAAGCCGACTATCATTACCAACGGTCTGAAGTACTCGCTCGCCACC
GGCAACTGGGGTGACCAGAAGAAAGCCATGAGCTCCACAGCTGGTGTTTCGCAAGTGCTAAACCG
ATACACAT------TTTCCTCGA---
CGCTCTCTCACTTGAGACGTACAAACACTCCCGTAGGGAGAGATGGCAAGCTCGCCAAGCCACGGC
AACTTCATAATACTCACTGGGGTCTTGTCTGTCCCGCCGAAACTCCCGAAGGCCAAGCTTGTGGCCT
GGTGAAGAACCTGTCATTGATGTGCTCCATCAGCGTGGGCACATCGACAGAGCCCATTATCGATTA
CATGATTACCCGAAACATGGAAGTGCTCGAGGAATACGAGCCCATGCGATATCCCCACGCCACCA
AGATCTTCCTCAACGGCTCTTGGATTGGTGTTCACCAGGACCCGAAGGCGCTCGTCAGGGATGTTC
AGCAACTGCGCCGGAGCAATCAGATTCCAGCAGAGGTGTCCCTAGTTCGCGACATCCGAGACCGC
GAGTTTAAGATTTTCTCGGACGCTGGTCGCGTCATGCGACCCTTGTTTGTTGTCGAACAGGAGAGC--
----
GTGCCCGAGACGGGTGTCGAGAAGGGATCACTAGCTCTCAACAAGGACATGGTGAGACGACTTGA
AATCGACCAAACGCTCCCTCCTGGAAGCGAAGAGTACTACGGCTGGCAAGGTTTGGTAAACGACG
GTGTCATTGAATACCTTGATGCCGAGGAGGAAGAGACGGCTATGATATGCATGACGCCAGAAGAT
CTCGAGATCTATCGGAGGACCAAGCTTGGTGAAGAGATTGTGAACGACAACGGA------
GACGATCTTAATAAGCGACTCAAGACAAAAATAAATCCAACCACGCACATGTACACGCATTGCGA
AATTCATCCTAGTATGCTTCTGGGTATCTGCGCCAGCATCATCCCCTTCCCTGATCACA--------
TCCCCGAACGCGTCCCCCAAACCCCTGGCTTGCTACCCCTC----ACAAGCTCTGCAATCATA------------
--------------TGTCATGTAAACACACCTC-
GTGATTCGAACATGGCAAGCTAACCGCGTCTTTTTTTTTTTATAGGTTCACCTCCAGACCGGCCAAT
GCGTAAGTACTTCTGT----AACGACGGCCGACTAA-----
GCGCGCGAACATGGCGAGGCTCACATAATATTCCTAGGGTAACCAAATCGGTGCTGCTTTCTGGCA
AACCATCTCTGGCGAGCACGGTCTCGACAGCAATGGCGTGTACGTATCTACCCTCCTCTTTTCTCCT
TTACATATAAAGGCTGACCGGTTCTCTATAGTTACAACGGCACGTCCGAGCTCCAGCTGGAGCGGA
TGAGCGTGTACTTCAACGAGGTAATGTGGTCGGATG---GGCACCTGGT----
AACTCTTGCCCCTAATTAACCTGTGGTGTGCAGGCCTCTGGTAACAAGTATGTTCCTCGCGCCGTCC
TCGTCGATCTCGAGCCTGGCACCATGGATGCCGTCCGCGCTGGTCCTTTCGGCCAGCTCTTCCGTCC
CGACAACTTCGTCTTCGGTCAGTCTGGTGCTGGCAACAACTGGGCCAAGGGTCATTACACTGAGGG
TGCCGAGCTGGTTGACCAGGTTTTGGATGTTGTTCGTCGTGAGGCTGAGGGCTGCGACTGCCTCCA

GGGCTTCCAGATCACCCACTCCCTCGG---

>Xylaria_hypoxylon

-CATTAAAGAGTTAATACAACTCCCAAACCCATGTGAA-CTTACCTCTGTTGCCTCGGCA---
GGTGTGTTTACCCTGTGGTC-------------CTACCCTGTAGGAC------------CTACCTGGTAGACACG--
GGTACGCCTGCCGGTGGCCCATGAAACTCTGTT--ATTCTATGTTATTCTGAAT-
CTATAACTAAATAAGTTAAAACTTTCAACAACGGATCTCTTGGTTCTGGCATCGATGAAGAACGCA
GCGAAATGCGATAAGTAATGTGAATTGCAGAATTCAGTGAATCATCGAATCTTTGAACGCACATTG
CGCCCATTAGTATTCTAGTGGGCATGCCTGTTCGAGCGTCATTTCAACCCTTAAGCCCCTGTTGCTT
AGCGTTGGGAGCCTACAGCCT----
TGTAGCTCCCCAAAGTTAGTGGCGGAGTCGGTTCACACTCTAGACGTAGTAGACTATCTCG--
TCTGTAGTGAGGCCGGTCCCCTGCCGTAAAACCCCCTA--
TTTTTAAAGGTTGACCTCGGATCAGGTAGGAATACCCGCTGAACTTAAGCATATGAAGCGGCAACA
GCTCAAA-TTTGAAATCTGGCT----TTC-----G-
GGTCCGAGTTGTAATTTGTAGAGGATGCTTTTGGCGCGGT-
GCCTTCCGAGTTCCCTGGAACGGGACGCCTTAGAGGGTGAGAGCCCCGTACGGTTGGA-
CACCAAGCCTCTGTAAAGCTCCTTCGACGAGTCGAGTAGTTTGGGAATGCTGCTCTAAATGGGAGG
TAAATTTCTTCTAAAGCTAAATATTGGCCAGAGACC-
GATAGCGCACAAGTAGAGTGATCGAAAGATGAAAAGCACTTTGAAAAGAGGGTTAAATAGCACGT
GAAATTGTTGAAAGGGAAGCG-TTTGCGACCAGACCTTTTCTTAGCGGATCATCCGGTGTTA--
TCACCGGTGCACTTCGCTAAG--TTTAGGCCAGCATCGGTTTC---
TGTAGGGGGATAAAAGCCTTGGGAACGTAGCTC----CTTCGGGAGTGTT-
ATAGCCCTTTGCATAATACCCTTCT-GGGGACCGAGGACCGCGCTATAT---
GCAAGGATGCTGGCATAATGGTCGTCAACGACCCGTCTTGAAACACGGACCAAGGAGTCGAACAT
TTATGCGAGTGTTTGGGTG--TTAAACCCTCACGCGTAATGAAAGTGAACGGAGGTGAGAGCCCTT--
--AC----GGGTGCATCATCGACCGATCCTGATG-
TCTTCGGATGGATTTGAGTAAGAGCATAACTGTTCGGACCCGAAAGATGGTGAACTATGCGTGGAT
AGGGTGAAGCCAGAGGAAACTCTGGTGGAGGCTCGCAGCGGTTCTGACGTGCAAATCGATCGTCA
AATCTGCGCATGGGGGCGAAAGACTTATCGA-
ACCATCTAGTAGCTGGTTACCGCCGAAGTTTCCCTCAGGATAGCAGTGT--
TGTTCTTCAGTTTTATGAGGTAAAGCGAATGATTAGGGACTCGGGGGCGCTTTTTGCCTTCATCCA
TTCTCAAACTTTAAATATGTAAGAAGCCCTTGTTACTTAATTGAACGTGGGCATTCGAATGTACCAA
CACTAGTGGGCCATTTTTGGTAAGCAGAACTGGCGATGCGGGATGAACCGAACGCGGGGTTAAGG
TGCCGGAGTGGACGCTCATCAGACACCACAAAAGGTGTTAGCACATTTAGACAATAGGACGGTGG
CCATGGAAGTCGGAATCCGCTAAGGACTGTGTAACAACTCACCTATCGAATGTGCTAGCCCTGAAA
ATGGATGGCGCTCAAGCGTCC-CACCCATACCTCGCCCTCAGGGTAGAAACGATGCCCTGAGG-------
----

TTGTCGCATTTGCGAAGAACGAATACTCCAGGTGGTAGAGACGGTAAGCTTGCGAAACCACGGCA
GCTCCACAACAGCCACTGGGGTCTCGTCTGCCCGGCCGAGACCCCCGAAGGTCAGGCTTGTGGCCT
AGTCAAAAACCTGTCCCTCATGTGCTCCATCAGTGTTGGTACGTCCACGGAACCTATTATCGATTAT
ATGATATCGCGTAATATGGAGGTTTTAGAGGAGTACGACCATCACAGATACCCTAATGCCACCAAG
ATCTTTCTCAATGGTGCATGGATCGGCGTCCACCAGGATCCTAAGTCTCTTGTGAGGGATGTGCAA
CAATTGCGCCGAACGAATCAGATTCCCGCTGAAGTATCCTTGATTCGAGATATTCGTGACCGCGAA
TTCAAAATCTTCTCGGATGCCGGCCGTGTCATGCGGCCCCTGTATGTGGTTGAGCAGGAGGAT-------
----

GACGCTGAGAATGGTGTCGAGAAGGGCACACTGGTCTTAACAAAGGACATGGTTCGGAGACTTGA
GATCGACCAGACCCTCCCACCCGGAAGTGACGAATACTTCGGATGGCATGGCCTGGTTCATGCTGG
TGTCATCGAATACATGGATGCTGAAGAGGAGGAGACTGCGATGATTTGCATGACCCCCGAGGACTT
GGAAAGTTTCCGATGCTCCAAGTTAGGTTTACCAGATCCTTTCAACAACGAGGACACTTTCGCCCC
AAACAAGCGGCTGAAGACGAGAATAAACCCGACAACCCATATGTACACTCACTGTGAAATCCACC
CGAGCATGCTTCTTGGCATTTGTGCCAGTATCATCCCCTTCCCCGATCATAATCAAGTATACCGAAA
CGCGTCCCTAAATCCCTTGATTAGCTTCTCCATATACACAAGCATCGCAGTCAAACTAT-

TCGTAGCTGCCAATAACACA-
CTTCCTTGAATTGTCTAGTAATAATATGAGAACTACCTGCTAACCATGCTTTTCC--
TTTTGCAGGTTCACCTCCAAACCGGCCAATGCGTAAGTCGCCCCCC-------
GATCCTCGATAACATGTC----TAGAACCTCCCGAGGCTCACACT--
TACGACAGGGTAACCAAATTGGTGCTGCTTTCTGGCAACAAATTTCCGGCGAGCACGGTCTCGATG
GCAGTGGCGTGTATGTCTATGATCTATGAACTAGACAACCACGACTGTCGACTAACACTTGTGGGC
CAGTTACAACGGAACCTCTGAGCTCCAGCTGGAGCGCATGAGCGTTTACTTCAATGAGGTAGAAAG
CCCCATCAAGTCGCGTTCGTCTCGT-GCAACAACCATTGAT--
GCTGATATTTTCTAGGGTGCTAATAACAAATATGTTCCTCGCGCCGTCCTCGTCGACTTGGAGCCCG
GTACCATGGATGCTGTCCGTTCTGGTCCCTTTGGTCAGCTCTTCCGACCCGACAACTTCATCTTCGG
CCAGTCTGGTGCTGGCAACAACTGGGCCAAGGGTCACTACACAGAGGGTGCTGAGCTCGTTGACGC
CGTTCTTGATGTCGTTCGTCGCGAGGCTGAGGGCTGCGATTGCCTCCAGGGTTTCCAGATCACCCAC
TCGCTCGGTGGTGGTACCGGTGCTGGTATGGGTACTCTGCTGATCTCCAAGATTCGCGAGGAATTC
CCTGACCGCATGATGGCTACCTTCTCCGTCATGCCCTCTCCCAAGGTCTCAGATACCGTCGTCGAGC
CTTACAACGCTACCCTCTCCGTCCACCAGTTGGTTGAGAACTCCGATGAGACCTTCTGTATTGACAA
CGAGGCTCTGTACGATATCTGCATGCGCACCTTGAAGCTATCCAACCCCTCATATGGTGATTTGAAC
CACCTTGTCTCTGCCGTCATGTCTGGCGTAACCACCTGCCTGCGTTTCCCCGGTCAGCTTAACTCTG
ATCTGCGCAAACTAGCCGTCAACATGGTGCCCTTCCCTCGTCTACACTTCTTTATGGTCGGGTTTGC
CCCTCTCACTAGCCGTGGTGCCCACTCTTTCCGTGCTGTCACGGTTCCCGAGCTGACCCAGCAAATG
TTCGACCCCAAGAACATGATGGCTGCCGCTGACTTCCGCAACGGTCGTTACCTCACATGCTCTGCTA
TCTTGTAAGAATTTGCCCTTTTATC---
TTAACACGGCCAATTGCTAACTTATCTTTACCAGCCGTGGCAAGGTTTCCA

>Xylaria_arbuscula

-CATTAAAGAGTTAA-ACAACTCCTAAACCCATGTGAA-CCTACCTTTGTTGCCTCGGCA---
GGTCAACTTACCCGGAGGAC-------------CTACCCTGTAG-----------GGACTTACCCGGTAGTTGCCG-
GCATAACCTGCCGGTGGTCTACTAAACTCTGTT---
TACTATGTTATTCTGAATAATATAACTAAATAAGTTAAAACTTTCAACAACGGATCTCTTGGTTCTG
GCATCGATGAAGAACGCAGCGAAATGCGATAAGTAATGTGAATTGCAGAATTCAGTGAATCATCG
AATCTTTGAACGCACATTGCGCCCATTAGTATTCTAGTGGGCATGCCTGTTCGAGCGTCATTTCAAC
CCTTAAG-
CCCTGTTGCTTAGCGTTGGGAGCCTACAGATACCTCTGTAGTTCCTTAAAGTTAGTGGCGGAGTCGG
TTCACACTCTAGACGTAGTAAATTATCTCG--
CCTATAGATGAGCCGGTCCCTTGCCGTAAAACCCCCTA--
TTTCTAAAGGTTGACCTCGGATCAGGTAGGAATACCCGCTGAACTTAAGCATATGAAGCGGCAACA
GCTCAAA-TTTGAAATCTGGCC----TTC-----G-
GGTCCGAGTTGTAATTTGTAGAGGATGCTTTTGGCGCGGT-
GCCTTCCGAGTTCCCTGGAACGGGACGCCTTAGAGGGTGAGAGCCCCGTACGGTTGGA-
CACCAAGCCTCTGTAAAGCTCCTTCGACGAGTCGAGTAGTTTGGGAATGCTGCTCTAAATGGGAGG
TAAATTTCTTCTAAAGCTAAATATTGGCCAGAGACC-
GATAGCGCACAAGTAGAGTGATCGAAAGATGAAAAGCACTTTGAAAAGAGGGTTAAATAGCACGT
GAAATTGTTGAAAGGGAAGCG-TTTGCGACCAGACTTTTCCCTAGCGGATCATCCGGTGTTC--
TCACCGGTGCACTTCGCTAGG--TTAAGGCCAGCATCGGTTTC---
TGTAGGGGGATAAAAGCTTGGGGAATGTAGCTC----CCTCGGGAGTGTT-
ATAGCCTCTTGTATAATACCCTTAC-GGGGACCGAGGACCGCGCT-TTT---
GCAAGGATGCTGGCGTAATGGTTGTCAACGACCCGTCTTGAAACACGGACCAAGGAGTCGAACAT
TTGTGCGAGTGTTTGGGTG--TTAAACCCTCACGCGTAATGAAAGTGAACGGAGGTGAGAGCCCTT--
--AC----GGGTGCATCATCGACCGATCCTGATG-
TCTTCGGATGGATTTGAGTAAGAGCATAACTGTTCGGACCCGAAAGATGGTGAACTATGCGTGGAT
AGGGTGAAGCCAGAGGAAACTCTGGTGGAGGCTCGCAGCGGTTCTGACGTGCAAATCGATCGTCA
AATCTGCGCATGGGGGCGAAAGACTTATCGA-
ACCATCTAGTAGCTGGTTACCGCCGAAGTTTCCCTCAGGATAGCAGTGT--
TGTTCTTCAGTTTTATGAGGTAAAGCGAATGATTAGGGACTCGGGGGCGCTTTTTAGCCTTCATCCA
TTCTCAAACTTTAAATATGTAAGAAGCCCTTGTTACTTAATTGAACGTGGGCATTCGAATGTACCAA
CACTAGTGGGCCATTTTTGGTAAGCAGAACTGGCGATGCGGGATGAACCGAACGCGGGGTTAAGG
TGCCGGAGTGGACGCTCATCAGACACCACAAAAGGTGTTAGCACATTTAGACAATAGGACGGTGG
CCATGGAAGTCGGAATCCGCTAAGGACTGTGTAACAACTCACCTATCGAATGTGCTAGCCCTGAAA

ATGGATGGCGCTCAAGCGTCC-
CACCCATACCCCGCCCTCAGGGTAGAAACGATGCCCTGAGGGCGGAGCATCGAGCAAGGCAAGCA
ATTCAATATTGCACTAGCTGTGAAGTCTAACATAATCACGAGTGGGTTGAAGTACTCACTCGCTAC
CGGCAACTGGGGTGACCAGAAAAAGGCAATGAGCTCCACAGCCGGTGTTTCGCAAGTGTTGAATC
GATACACAT------TCGCCTCTA---
CCTTGTCACACTTGCGAAGAACAAATACCCCAGTCGGTAGAGATGGCAAGCTTGCCAAACCCCGAC
AACTTCACAATACCCACTGGGGGCTTGTCTGTCCAGCCGAGACCCCAGAAGGACAGGCATGTGGTT
TGGTCAAAAACTTGTCGCTCATGTGCTCTATCAGCGTCGGTACATCAACGGATCCTATTATAGAATA
TATGATCCTTAGGAATATGGAAGTGCTAGAAGAGTATGATCCTGGTAGGTATCCCAACGCCACCAA
GGTGTTTCTTAATGGTGCATGGATCGGCGTCCACCAGGATCCCAAGGCTCTAGTTAAGGATGTGCA
ACAATTGCGCCGAACAAACCAGATCCCAGCTGAAGTATCCCTCATCCGGGATATTCGCGACCGTGA
GTTCAAGATTTTCAGTGACGCCGGTCGCGTCATGCGCCCTCTGTTCGTAGTCGAGCAAGAAGAC-----
----

GACGCTGAAAGGGGCATTGAGAAAAGCACGCTCGTTTTGACCAAAGATATGGTTCGGCGGCTTGA
GGAAGACCAGAGCCTTCCACCCGGACACGAAGATTACTATGGATGGCAAAGTTTGGTTAATGCCG
GTGTGATTGAATATATGGACGCTGAAGAAGAGGAGACGGCGATGATCTGCATGACCCCGGAAGAC
CTAGAGAGTTTCCGATGCAGCAAGTTGGGTCTAGCAGATCCTCACAACAACGATGATGTCTTTGCT
CCCAACAAGCGGCTGAAGACGAGGATAAATCCGACCACACACATGTACACTCACTGTGAAATTCA
TCCGAGCATGCTTCTAGGCATTTGTGCCAGTATCATTCCCTTCCCGGATCACAACCAAGTAGCCCGA
GACGCGTTACTCAATCTTCTCATTCCCATCATCTCATCTGCCAGCATGATTACTAGACAAT-
TTTCTGTCATCAGAGGCATGACTTGATTGAATTATCTGGCAATATTGGGTGAAACCTAAACTAACC
GCG----CTCCTCTCTACAGGTCCACCTCCAGACCGGCCAATGCGTAAGTCGC---------
TACGACCTTCGACGATATACC----
TTGAAACAGCCGAAACTTACATAGAATGATCAGGGTAACCAAATTGGTGCTGCTTTCTGGCAGCAA
ATCTCCGGCGAGCACGGTCTCGATGGCAGTGGCGTGTATGTGTACCTATGCCC--
TTCGGACTATAAGGACAATGACTGATAGTTATGGAATAGTTACCAGGGAACCTCTGACCTCCAGCT
GGAGCGTATGAGGGTTTACTTCAACGAGGTAGGCTCAAATTCCGAGCTACATCGATATCATACAAG
TAA----
TGGTCTCATGGGTTTGTTCAGGGCTCGGGCAACAAGTACGTTCCTCGCGCTGTCCTCGTCGATTTAG
AGCCTGGTACCATGGACGCTGTCCGTGCTGGTCCCTTCGGTCAGCTCTTCCGACCCGACAACATCGT
CTTCGGTCAGTCTGGTGCTGGCAACAACTGGGCCAAGGGTCACTACACTGAGGGTGCTGAGCTTGT
TGACAACGTTCTTGACGTTGTCCGTCGTGAGGCTGAGGGCTGTGACTGCCTCCAGGGTTTCCAGATC
ACCCACTCGCTCGGTGGTGGTACCGGTGCCGGTATGGGTACGCTGCTCATCTCCAAGATCCGTGAG
GAGTTCCCCGACCGCATGATGGCTACCTTCTCCGTCATGCCCTCCCCCAAGGTATCGGACACCGTCG
TCGAACCTTACAACGCCACTCTCTCCGTCCACCAGCTGGTCGAGAACTCCGACGAGACCTTCTGCA
TTGACAACGAGGCTCTCTACGACATCTGCATGCGCACCCTGAAGCTATCCAACCCTTCGTACGGTG
ACCTGAACCACCTTGTCTCCGCTGTCATGTCTGGCGTCACCACTTGCCTTCGTTTCCCTGGACAACT
TAACTCTGACCTGCGCAAGTTGGCCGTCAACATGGTGCCCTTCCCTCGTCTGCACTTCTTCATGGTC
GGCTTCGCCCCTTTGACCAGCCGTGGTGCCCACTCTTTCCGTGCCGTCACGGTTCCTGAGTTGACCC
AGCAAATGTTCGACCCCAAGAACATGATGGCCGCCGCTGACTTCCGCAACGGTCGCTACCTGACAT
GCTCTGCCATCTTGTGAGTACTCTTACTTCGAGC---
CTAACATGTTTGATTACTAATCTGTAATCACTAGCCGTGGCAAGGTCTCTA

>Annulohypoxylon_annulatum

------------------------ACCCTTTGTGAACCTTACCGTCGTTTCCTCGGCGCACTGCGTGG----------
GCTACCCTGTAGCGGTTTACCCTACA------------GGACGCACCCTGCAGCGG-----------
CGCCGAAAGGACTACCAAACTC--TTTTATCCAAGTTACCTC-
GAACAATTTAATACAATAGTTTAAAACTTTCAACAACGGATCTCTTGGTTCTGGCATCGATGAAGA
ACGCAGCGAAATGCGATAAGTAATGTGAATTGCAGAATTCAGTGAATCATCGAATCTTTGAACGCA
CATTGCGCCCATTAGTATTCTAGTGGGCATGCCTATTCGAGCGTCATTTCAACCCTTAAG-
CCCTGTTGCTTAGCGTTGGGAATCTGCTAGCCTCGGCGCAGTTCCTTAAATTCATTGGCGGAGCTGT
GGCACACTCTAGGCGTAGTAGTACACCTCGCCTCTAGAGTGGCCGCGGTTACTGGCCGTAAAACCC
CTT--
ATTTCTAGTGGTTGACCTCGGATTAGGTAGGAATACCCGCTGAACTTAAGCATATGAAGCGGCAAC
AGCTCAAA-TTTGAAATCTGGCC----CTC-----
GCGGTCCGAGTTGTAATTTGCAGAGGATGCTTTTGGTGCGGT-
GCCTTCCGAGTTCCCTGGAACGGGACGCCAGAGAGGGTGAGAGCCCCGTACGGTTGGC-

CGCCTACCCTATATATAGCTCCTTCGACGAGTCGAGTAGTTTGGGAATGCTGCTCTAAATGGGAGG
TAAATTTCTTCTAAAGCTAAATACCGGCCAGAGACC-
GATAGCGCACAAGTAGAGTGATCGAAAGATGAAAAGCACTTTGAAAAGAGGGTTAAATAGCACGT
GAAATTGTTGAAAGGGAAGCG-TTTGCGACCAGACTTTTTCCAGGCGGATCATCCGGTGTTC--
TCACCGGTGCACTTCGCCTGG--TTTAGGCCAGCATCGGTTTC---
TCTAGGGGGATAAAGGCCTGGGGAACGTAGCTC----TTTAGGGAGTGTT-
ATAGCCCCTTGCGTAATACCCCTCG-GGGGACCGAGGACC---

ACGCTGCATCGACGGAAACAAGCGTTTCCAAATCGAACTTGCCGCCAAGCCCGCAATTATCACCAA
CGGCTTGAAGTACTCGCTCGCCACGGGTAACTGGGGCGATCAAAAGAAGGCGATGAGTTCGACTG
CCGGTGTATCGCAGGTCTTGAACCGCTACACTT------TCTCCTCGA---
CGCTTTCTCACTTACGACGAACGAACACGCCCATCGGAAGAGATGGAAAGCTAGCCAAGCCGCGG
CAGCTCCACAACACTCACTGGGGTCTCGTCTGCCCGGCGGAGACTCCCGAGGGCCAGGCTTGCGGC
TTGGTGAAGAATTTATCCTTAATGTGTTCTATTAGCGTTGGAACATCGACAGATCCTATCGTGGACT
ACATGATAACTAGGAATATGGAAGTCCTGGAGGAGTATGAACCGATGAGATATCCCAACGCCACC
AAGATCTTCCTCAACGGCTCTTGGATCGGTGTACACCAGGATCCCAAGACCCTCGTCAGGGACGTT
CAGGCGCTTCGTCGAGCCAACCAGATTCCTGCCGAGGTCTCGCTGGTTCGTGATATCCGAGACCGT
GAGTTCAAGATCTTTTCTGACGCCGGTCGTGTGATGCGTCCCTTGTTTCGCGTACAGCAGGAAGAC--
----
ATTGCCGAGCAGGGTATTGAGAAGGGCACATTGGCTCTTACTAAGCAGATGATCAAACGCTTAGAA
GCAGATGTCGATCTGGACCCGGAGAGCGAAGCATACTTTGGCTGGCAAGGTCTAGTCAACGAGGG
CGTTATCGAGTTCCTCGATGCGGAAGAAGAGGAGACTGCCATGATTTGCATGACTCCCGAAGATTT
GGATACTTATCGTATGACCAAGCTTGGGTACGAGGTGTCCCAGGACAACGGG------
GACGAGGTGAACAAGCGACTCAAGACCAAGGTGAACCCGTCAACGCACATGTATACCCATTGCGA
GATCCATCCC---CCCCTGAACGCGTC-
CTCAAACCCCTTGATTCCTGCCCCTCACGCACAAAAAAC---AACACAACGTCGCA---
TTCTATGTTTAACATCACCATCAAGTCGAGCGATAGTGATTCCACATCAGATAGCTAACCGTGTTTT
TTCATCCAAATAGGTTCACCTTCAGACCGGCCAGTGCGTAAGTACTATAGC----
TCCTACGCCCGACGAAGAATCGCGACGGAGTATAGCGGGGCTCACG--
AATATTATAGGGTAACCAAATTGGTGCTGCTTTCTGGCAAACCATCTCTGGCGAGCACGGCCTCGA
CAGCAATGGCGTGTAAGTACCTGAATCGTCAATTCCAACGCCAAAAAAACAAACTGACCGCCAAT
AAATAGCTACAATGGAACCTCGGAGCTCCAGCTCGAACGCATGAGCGTTTACTTTAACGAGGTACG
CAACCAGGGAAACACATGGCCTGTTCCCAGGAGTAGTTACTAATCACCCCAACATGCACAGGCATC
TGGTAACAAGTATGTTCCCCGAGCCGTCCTCGTCGACCTCGAGCCGGGTACCATGGACGCCGTCCG
TGCTGGGCCTTTCGGCCAACTTTTCCGACCCGACAACTTCGTTTTCGGCCAGTCTGGTGCCGGAAAC
AACTGGGCGAAGGGTCACTACACCGAGGGAGCTGAGCTGGTTGACCAGGTTCTTGATGTCGTTCGT
CGTGAGGCCGAGGGCTGTGACTGCCTCCAGGGTTTCCAGATCACCCACTCTCTCGGCGGTGGTACC
GGTGCCGGTATGGGTACTCTGCTGATCTCCAAGATCCGCGAGGAGTTCCCCGACCGCATGATGGCC
ACCTTCTCCGTCGTTCCCTCTCCTAAGGTTTCCGACACCGTCGTCGAGCCTTACAACGCCACTCTCT
CAGTCCACCAGTTGGTTGAGAACTCGGATGAGACGTTCTGTATCGACAACGAAGCTCTGTACGACA
TCTGCATGCGTACTCTCAAGTTATCCAACCCCTCTTACGGCGACTTGAACCACTTGGTTTCCGCCGT
CATGTCCGGTGTCACCACTTGCCTGCGTTTCCCCGGTCAGCTGAACTCTGACCTGCGCAAGCTTGCC
GTGAACATGGTTCCTTTCCCCCGTCTTCACTTCTTCATGGTCGGCTTTGCTCCCCTGACCAGCCGTGG
CGCTTACTCTTTCCGCGCCGTTACCGTTCCCGAGTTGACGCAGCAGATGTTCGATCCCAAGAACATG
ATGGCCGCGTCCGACTTCCGCAACGGCCGCTACCTAACGTGCTCCGCCATCTTGTAAGATAACACC
ATTGTATTATTTTTAATGGGCAACATGCTAATTCGAACCTTGTAGCCGTGGCAAGATCTCCA

>Annulohypoxylon_truncatum

ACCCTTTGTGAACCTTACCGTCGTTTCCTCGGCGCACTGCGCGACCGCCCCGCAGCTCCCCGCGGGC
AGCTTACCCTGTAC-----------GGATCTACCCTGCAGCAGGGC-
GCAGCGGCGCCGAAAGGACCGCTAAACTC--

TTTTATCCAAGCTACGTCTGAACAATTTAACAAAATAGTTTAAAACTTTCAACAACGGATCTCTTGG
TTCTGGCATCGATGAAGAACGCAGCGAAATGCGATAAGTAATGTGAATTGCAGAATTCAGTGAATC
ATCGAATCTTTGAACGCACATTGCGCCCATTAGTATTCTAGTGGGCATGCCTATTCGAGCGTCATTT
CGACCCTTAAG-
CCCTGTTGCTTAGCGTTGGGAGTCTGCGGCTTCGGCCGCAGTTCCTTAAAGTCAGTGGCGGAGCTGT
GGCACACTCTAGGCGTAGTAGTCCGCCTCGCCTCCAGAGTGGCCCCGGCTGCCTGCCGTAAAACCC
CT-
AATTTTCTAGTGGTTGACCTCGGATTAGGTAGGAATACCCGCTGAACTTAAGCATATGAAGCGGCA
ACAGCTCAAA-TTTGAAATCTGGCC----CTC-----
GCGGTCCGAGTTGTAATTTGCAGAGGATGCTTTTGGTGCGGT-
GCCTTCCGAGTTCCCTGGAACGGGACGCCAGAGAGGGTGAGAGCCCCGTACGGTTGGA-
CACCTACCCTATATATAGCTCCTTCGACGAGTCGAGTAGTTTGGGAATGCTGCTCTAAATGGGAGG
TAAATTTCTTCTAAAGCTAAATACCGGCCAGAGACC-
GATAGCGCACAAGTAGAGTGATCGAAAGATGAAAAGCACTTTGAAAAGAGGGTTAAATAGCACGT
GAAATTGTTGAAAGGGAAGCG-TTTGCGACCAGACTTTTTCCAGGCGGATCATCCGGTGTTC--
TCACCGGTGCACTTCGCCTGG--TTTAGGCCAGCATCGGTTTC---
CTTAGGGGGATAAAGGCCTGGGGAACGTAGCTC----TCTAGGGAGTGTT-
ATAGCCCCTCGCGTAATACCCCTCG-GGGGACCGAGGACCGCGC---

GCGCTGCATCGACGGGAACAAACGTTTCCAGATTGAACTTGCCGCCAAGCCCGCCATTATCACCAA
CGGCTTGAAGTACTCGCTCGCTACGGGTAACTGGGGCGATCAAAAGAAGGCGATGAGCTCGACTG
CCGGTGTATCGCAGGTCTTGAACCGCTACACTT------TCTCGTCAA---
CCCTTTCTCATTTACGACGAACGAACACGCCCATCGGAAGAGATGGAAAGCTAGCCAAGCCACGG
CAGCTCCACAACACACACTGGGGTCTCGTCTGTCCGGCAGAGACTCCCGAAGGCCAGGCTTGTGGC
TTGGTGAAGAATCTATCCTTGATGTGTTCTATCAGTGTTGGAACATCGACAGATCCTATCGTGGACT
ACATGATCACTAGGAATATGGAAGTCCTGGAGGAGTATGAACCGATGAGATATCCCAACGCCACT
AAGATCTTCCTCAACGGCTCCTGGATCGGTGTACACCAGGACCCAAAGACCCTCGTCAGGGACGTC
CAGGCACTTCGTCGAGCCAACCAGATACCTGCTGAGGTCTCGCTAGTTCGTGATATCCGAGACCGT
GAATTCAAGATCTTTTCGGATGCCGGTCGTGTGATGCGCCCCCTGTTCCGCGTACAACAGGAAGAC-
----
ATCGCCGAGCAGGGCATCGAGAAGGGCACCTTGGCTCTTACCAAGCAGATGATCAAACGTCTAGA
AGCAGATGTCGATCTGGACCCGGAGAGCGAGGCGTACTATGGCTGGCAAGGTCTAGTCAACGAGG
GAGTTATCGAGTTCCTCGATGCGGAAGAAGAGGAGACTGCGATGATTTGCATGACGCCGGAAGAT
TTGGACACCTACCGTATGACCAAGCTTGGGTATGAGGTGTCCCAGGACAACGGA------
GATGAGGTGAATAAGCGACTCAAGACTAAAATCAACCCGTCAACGCACATGTATACCCATTGCGA
GATCCATCCCAGTATGCTCCTGGGTATCTGCGCAAGCATCATTCCATTCCCAGATCATAACCAGGTA
--------------------------------TGCCCCTCACGCACAAAAAAC---ACCACAAGATCCTATTC---
TCTGTGTAACATCAGCATCAAATCGAACAACCATGATTCCAGATCAGATAGCTAACCGTGTTTTTC
ACCTCGATAGGTTCACCTCCAGACCGGCCAGTGCGTAAGTACTACAGC----
TACAACCCCCGACGAAGAATCGCGATGCAATATAGCGGGGCTCAC--
GAATATCATAGGGTAACCAAATCGGTGCTGCTTTCTGGCAAACCATCTCTGGCGAGCACGGTCTCG
ACAGCAATGGCGTGTAAGTATCTGAATCGTCAATTCCGACGCCAAAAAAACAAACTGACCGCCAA
TAAACAGCTACAATGGAACCTCGGAGCTCCAGCTCGAACGCATGAGCGTTTACTTTAACGAGGTAC
GCAACCAGGGAAACACATGGTATATCCCGAGGAGTAGTTACTAATCACCCCAACATGCACAGGCA
TCTGGTAACAAGTATGTTCCTCGAGCCGTCCTCGTCGACCTCGAGCCGGGCACCATGGACGCCGTC
CGTGCTGGCCCGTTCGGCCAACTTTTCCGACCCGACAACTTCGTCTTCGGCCAGTCTGGTGCCGGAA
ACAACTGGGCGAAGGGTCACTACACTGAGGGTGCTGAGCTTGTTGACAACGTTCTCGATGTCGTTC
GTCGTGAGGCTGAGGGCTGTGACTGCCTTCAGGGTTTCCAGATCACCCACTCTCTCGGTGGTGGTA
CCGGTGCCGGTATGGGTACTCTGCTGATCTCCAAGATCCGTGAAGAGTTCCCCGACCGTATGATGG
CCACCTTCTCCGTCGTTCCTTCTCCTAAGGTTTCCGACACCGTCGTCGAGCCTTACAACGCCACTCTT
TCAGTTCACCAGTTGGTTGAGAACTCGGACGAGACTTTCTGTATCGACAACGAAGCTCTGTACGAC
ATCTGCATGCGTACTCTCAAGCTGTCCAACCCCTCTTACGGCGACTTGAACCACCTGGTTTCCGCCG

TCATGTCAGGTGTCACCACTTGCTTGCGTTTCCCTGGTCAGCTGAACTCTGACCTGCGCAAACTCGC
CGTGAACATGGTTCCTTTCCCTCGTCTTCACTTCTTCATGGTCGGCTTCGCTCCCTTGACCAGCCGTG
GCGCTTACTCCTTCCGCGCCGTTACCGTCCCCGAGTTGACGCAACAGATGTTCGACCCCAAGAACA
TGATGGCCGCGTCCGACTTCCGCAACGGCCGCTACCTAACGTGCTCTGCCATCTTGTAAGATAACG
CCTTTCTATTATCTTTGATAAGCAATATGCTAATTC----------------------------

>Hypoxylon_cercidicola

-
CATTACTGAGTTCTAAAAACTCCCAACCCTTTGTGAACCCTACCGTCGTTGCCTCGGCGCTGAGCGC
GGCTACCCGGGAGCTACCCTGGAGACACCTACCCTGTAGGTGGCTACCCTGGAGTTACCCTGTAGT
TGCACTTTACGTTCCGCCGGAGGACCACCAAACTCTTCTTATTCTGGTGTATCTCTGAGTACGT-
AACAAAATAAGTTAAAACTTTCAACAACGGATCTCTTGGTTCTGGCATCGATGAAGAACGCAGCGA
AATGCGATAAGTAATGTGAATTGCAGAATTCAGTGAATCATCGAATCTTTGAACGCATATTGCGCC
CATTAGTATTCTAGTGGGCATGCCTATTCGAGCGTCATTTCAACCCTTAAG-
CCCTGTTGCTTAGCGTTGGGACCCTACCCTGTCAGGCGTAGTTCCCTAAAGGTAGTGGCAGTGTTA-
GGTACACTCGTAGCGTAGTAATTTTTCTCGCTCCTGTGGTGGCCCTGACGACTCGCCGTAAAACCCC
CTACACTTCTAGTGG---TGAAGCGGCAACAGCTCAAA-
TTTGAAATCTGGCC----CTC-----GTGGTCCGAGTTGTAATTTGTAGAGGATGCTTTTGGTGCGGT-
GCCTTCCGAGTTCCCTGGAACGGGACGCCAGAGAGGGTGAGAGCCCCGTACGGTTGGA-
CACCTACCCTATATATAGCTCCTTCGACGAGTCGAGTAGTTTGGGAATGCTGCTCTAAATGGGAGG
TAAATTTCTTCTAAAGCTAAATACCGGCCAGAGACC-
GATAGCGCACAAGTAGAGTGATCGAAAGATGAAAAGCACTTTGAAAAGAGGGTTAAATAGCACGT
GAAATTGTTGAAAGGGAAGCG-TTTGCGACCAGACCTTTTCTAGGCGGATCATCCGGTGTTC--
TCACCGGTGCACTTCGCCTAG--TCTAGGCCAGCATCGGTTTT---
CGTAGGAGGATAAAGGCCTGGGGAACGTATCTC----TTTCGGGAGTGTT-
ATAGCCCCTCGTGTAATACTCTTAC-GGGGACCGAGGACCGCGCT-TCG---
GCAAGGATGCTGGCGTAATGGTCGTCAACGACCCGTCTTGAAACACGGACCAAGGAGTCGAACAT
TTGTGCGAGTGTTTGGGTA--TTAAACCCTCACGCGTAATGAAAGTGAACGGAGGTGAGAGCCCTT--
---CG---GGGTGCATCATCGACCGATCCTGATG-
TCTTCGGATGGATTTGAGTAAGAGCATAACTGTTCGGACCCGAAAGATGGTGAACTATGCGTGGAT
AGGGTGAAGCCAGAGGAAACTCTGGTGGAGGCTCGCAGCGGTTCTGACGTGCAAATCGATCGTCA
AATCTGCGCATGGGGGCGAAAGACTTATCGA-
ACCATCTAGTAGCTGGTTACCGCCGAAGTTTCCCTCAGGATAGCAGTGT--TG-
TTTTCAGTTTTATGAGGTAAAGCGAATGATTAGGGACTCGGGGGCGCTTTATTGCCTTCATCCATTC
TCAAACTTTAAATATGTAAGAAGCCCTTGTTGCTTAATTGAACGTGGGCATTCGAATGTATCAACA
CTAGTGGGCCATTTTTGGTAAGCAGAACTGGCGATGCGGGATGAACCGAACGCGGGGTTAAGGTG
CCAGAGTGGACGCTCATCAGACACCACAAAAGGTGTTAGTACATCTAGACAGT-
GGACGGTGGCCATGGAAGTCGGAATCCGCTAAGGACTGTGTAACAACTCACCAACCGAATGTACT
AGCCCTGAAAATGGATGGCGCTCAAGCGTCT-
CACCCATACCTCGCCCTTAGGGTAGAAACGATGCCCTAAGG---
--TGTGTCGCAGGTG-
TGAACCGATACACAT------TCTCCTCGA---
CGCTTTCTCATTTGCGACGAACCAACACGCCCATTGGGCGAGACGGAAAGCTTGCGAAGCCTCGTC
AGCTTCATAATACCCATTGGGGTCTGGTTTGCCCGGCCGAGACGCCTGAGGGTCAGGCTTGCGGCC
TGGTGAAGAACTTGTCATTGATGTGTTCAATCAGCGTGGGTACTTCGACGGATCCTATCGTGGATTA
TATGATTACGAGGAATATGGAGGTTCTCGAGGAGTACGAGCCCTTGCGATACCCCGACGCTACCAA
GATCTTCCTCAACGGCTCTTGGATCGGAGTACATCAGAACCCCAAGGCTCTGGTGAGAGATGTGCA
GAATCTGCGCCGGGCTAATCAGATTCCGGCCGAAGTGTCGTTGGTCCGCGACATTCGTGACCGTGA
ATTCAAGATCTTTTCCGATGCCGGTCGGGTCATGCGTCCCTTGTTTGTCGTACAACAAGAGGAT------
----

ACCGAAGCTGGCACTAAGAAGGGGACGCTAGCTCTTACCAAGGAAATGATCCAGAGGCTCGAGGC
AGACGTTGATCTGGACCCCGACGGCGAGGAGTACTTTGGCTGGCAAGGCTTGGTCAATGAAGGTGT
CATCGATTACCTTGACGCCGAGGAAGAGGAGACGGCTATGATTTGCATGACGCCCGAGGACCTCG
AGATATACCGTCAGACCAAGGCCGGATTCGTAGTGTCCCAGGATAACGGG------
GATGAGATTAACAAGCGACTGCGGACCAAGGTGAACCCGACTACGCACATGTACACTCACTGCGA
GATTCATCCCAGCATGCTCCTAGGTATCTGCGCGAGCATCATTCCGTTCCCGGATC-----------
CCCCTGAACGCGTCCCCCAACCCCCTGATTTCCTGCCCCTCACCCACAAACACCACAGACACAGAA

TCTATCAAATCCCTACATCGC---------AAACCAC--CACA---
TTCTGGAAAACGAAAGCTAACCATATATCTTTATCGGAATAGGTTCACCTTCAGACCGGCCAATGC
GTAAGAACTACCTACACCTACAACCAGCGAT-A-
CCAACGCGGAGAAAGATGGTGGGGCTCACATGAATTTTACAGGGTAACCAAATTGGTGCTGCTTTC
TGGCAAACCATCTCTGGCGAGCACGGTCTCGACAGCAATGGCGTGTACGTATTCCATTGGTCAATT
CCATCGACGAGACT-
GAAACTAATAATTTGTAAATAGCTACAACGGAACCTCTGAGCTCCAGTTGGAGCGCATGAGCGTCT
ACTTCAACGAGGTATGCCACAATCAGGAA-
AGAAATCCGTGCGCGAGACATGGTACTAATTACACCGAAATTTGCAGGCTTCCGGCAACAAGTATG
TACCCCGTGCCGTCCTCGTCGATCTCGAGCCCGGTACCATGGATGCCGTCCGTGCTGGTCCCTTCGG
TCAGCTCTTCCGACCCGACAACTTCGTTTTCGGTCAGTCTGGTGCTGGCAACAACTGGGCCAAGGG
TCACTACACTGAGGGTGCCGAGCTCGTTGACCAGGTTTTGGATGTCGTTCGTCGTGAGGCTGAGGG
CTGCGACTGCCTTCAGGGTTTCCAGATTACCCACTCGCTCGGTGGTGGTACCGGTGCCGGTATGGGT
ACCTTGTTGATCTCCAAGATCCGCGAGGAGTTCCCTGACCGCATGATGGCTACTTTCTCCGTCGTTC
CCTCTCCTAAGGTCTCTGATACCGTTGTCGAGCCTTACAACGCCACCCTCTCCGTCCACCAGCTGGT
CGAGAACTCGGACGAGACCTTCTGCATTGATAACGAGGCCCTGTACGACATCTGCATGCGTACCCT
GAAGCTATCCAACCCCTCGTACGGTGACCTGAACCACCTGGTTTCCGCCGTCATGTCTGGTGTCACC
ACCTGCTTGCGATTCCCTGGCCAGCTGAACTCTGACCTCCGAAAGTTGGCTGTCAACATGGTGCCCT
TCCCTCGTCTGCACTTCTTCATGGTCGGCTTCGCTCCCTTGACCAGCCGTGGTGCTTACTCTTTCCGT
GCTGTCACCGTTCCCGAGTTGACTCAGCAAATGTTCGACCCCAAGAACATGATGGCTGCCTCTGAC
TTCCGTAACGGTCGTTACCTGACGTGCTCTGCCATCTTGTAAGATGCCTGTTTCAAGTTGTCGATTA
GTGCTTATTTGCTGACCTTCA--CTCTAGCCGTGGCAAGATCTCCA

>Hypoxylon_petriniae

-CATTACTGAGTTCT-
AAAACTCCCAACCCTTTGTGAACCTTACCGTCGTTGCCTCGGCGCTGAGCGTAGCTACCTGGGAGC
TACCCTGGAGACACCTACCCTGTAGGTGGCTACCCTGGAGCTACCCTGTAGTTGCACTTTACGCTCC
GCCGGAGGACCACCAAACTCTTCTTTTTATGGTGTATCTCTGAATCGTT-
AACTAAATACGTTAAAACTTTCAACAACGGATCTCTTGGTTCTGGCATCGATGAAGAACGCAGCGA
AATGCGATAAGTAATGTGAATTGCAGAATTCAGTGAATCATCGAATCTTTGAACGCATATTGCGCC
CATTAGTATTCTAGTGGGCATGCCTATTCGAGCGTCATTTCAACCCTTAAGCCCCTGTTGCTTAGCG
TTGGGACTCTACCCTGCCAGGTGTAGTTCCCTAAAGGTAGTGGCAGTGTTA-
GGTACACTCGTAGCGTAGTAATCTTTCTCGCTTCGGTGGTGGCCCTAGCTACTCGCCGTGAAACCCC
CTATACTTCTAGTGGTTGACCTCGGATTAGGTAGGAATACCCGCTGAACTTAAGCATA-----------------
---------------C----CTC-----GTGGTCCGAGTTGTAATTTGTAGAGGATGCTTTTGGTGCGGT-
GCCTTCCGAGTTCCCTGGAACGGGACGCCAGAGAGGGTGAGAGCCCCGTACGGTTGGA-
CACCTACCCTATATGTAGCTCCTTCGACGAGTCGAGTAGTTTGGGAATGCTGCTCTAAATGGGAGG
TAAATTTCTTCTAAAGCTAAATACCGGCCAGAGACC-
GATAGCGCACAAGTAGAGTGATCGAAAGATGAAAAGCACTTTGAAAAGAGGGTTAAATAGCACGT
GAAATTGTTGAAAGGGAAGCG-TTTGCGACCAGACCTTTTCTAGGCGGATCATCCGGTGTTC--
TCACCGGTGCACTTCGCCTGG--TTTAGGCCAGCATCGGTTTT---
CGTAAGAGGATAAAGGCTCGGGGAACGTATCTC----TCTCGGGAGTGTT-
ATAGCCCCTTGTGTAATACTCTTAT-GGGGACCGAGGACCGCGCT-TCG---
GCAAGGATGCTGGCGTAATGGTCGTCAACGACCCGTCTTGAAACACGGACCAAGGAGTCGAACAT
TTGTGCGAGTGTTTGGGTA--TTAAACCCTCACGCGTAATGAAAGTGAACGGAGGTGAGAGCCCTT--
---CG---GGGTGCATCATCGACCGATCCTGATG-
TCTTCGGATGGATTTGAGTAAGAGCATAACTGTTCGGACCCGAAAGATGGTGAACTATGCGTGGAT
AGGGTGAAGCCAGAGGAAACTCTGGTGGAGGCTCGCAGCGGTTCTGACGTGCAAATCGATCGTCA
AATCTGCGCATGGGGGCGAAAGACTTATCGA-
ACCATCTAGTAGCTGGTTACCGCCGAAGTTTCCCTCAGGATAGCAGTGT--TG-
TTTTCAGTTTTATGAGGTAAAGCGAATGATTAGGGACTCGGGGGCGCTTTATTGCCTTCATCCATTC
TCAAACTTTAAATATGTAAGAAGCCCTTGTTGCTTAATTGAACGTGGGCATTCGAATGTATCAACA
CTAGTGGGCCATTTTTGGTAAGCAGAACTGGCGATGCGGGATGAACCGAACGTGGAGTTAAGGTG
CCAGAGTGGACGCTCATCAGACACCACAAAAGGTGTTAGTACATCTAGACAGT-
GGACGGTGGCCATGGAAGTCGGAATCCGCTAAGGACTGTGTAACAACTCACCAACCGAATGTACT
AGCCCTGAAAATGGATGGCGCTCAAGCGTCT-
CACCCATACTTCACCCTTAGGGTAGAAACGATGCCCTAAGG---

--GTGTTGAACCGATACACAT----
--TCGCCTCGA---
CGCTTTCACATTTGCGAAGAACCAACACGCCCATTGGGCGAGATGGAAAGCTCGCGAAGCCTCGGC
AGCTTCATAATACCCATTGGGGTCTGGTCTGCCCAGCCGAGACACCCGAGGGTCAGGCTTGCGGTC
TGGTGAAGAACTTATCATTGATGTGTTCAATCAGTGTCGGCACCTCGACGGATCCTATCGTGGATTA
TATGATTACTAGGAATATGGAGGTCCTCGAGGAGTACGAACCTTTGCGATACCCCGACGCTACCAA
GATCTTCCTCAACGGCTCTTGGATCGGTGTACATCAGAACCCCAAGGCTCTAGTGAGAGACGTGCA
GAATCTGCGCCGGGCTAATCAGATTCCGGCCGAAGTGTCGTTGGTCCGCGACATTCGTGACCGTGA
ATTCAAGATCTTTTCCGATGCCGGTCGGGTCATGCGCCCCTTGTTTGTCGTACAACAAGAGGAT------
----
ACCGAGGCTGGCATTAAGAAGGGGACGCTAGCTCTTACTAAGGACATGATCCAGAGGCTCGAGGC
AGACGTCGATCTGGATCCCGACGGCGAGGAGTACTTTGGCTGGCAAGGCTTGGTCAATGAAGGTGT
CATCGACTACCTTGACGCCGAGGAGGAAGAAACGGCCATGATTTGTATGACGCCCGAAGACCTTG
AGACATACCGCCAGACCAAGGCGGGATTCGCAGTGTCCCAGGATAACGGG------
GATGAGATTAACAAGCGACTGCGGACCAAGGTTAACCCGACTACGCACATGTACACCCACTGCGA
GATTCATCCTAGCATGCTCCTAGGTATCTGCGCGAGCATT-----------------------------
CCCCTGAACGCGTCCCCCAGCCCCCTGATTTCCTGCCCCTCACGCACAAACACCACAAACACAGAA
CCTATCAAATCCCTACGTCGC---------AAACTAC--CACA---
GATTGGAAAATGAAAGCTAACCATATATCTTTATCGGAATAGGTTCACCTTCAGACCGGCCAATGT
GTAAGAACTATCTACACCTACAACC---AGCGA-
TCAACGCGAAGCAAGATGGTGGGGCTCACATGAATTTTATAGGGTAACCAAATTGGTGCTGCTTTC
TGGCAGACCATCTCTGGCGAGCACGGCCTCGACAGCAATGGCGTGTACGTATTCCATTGGTCAATT
CTCCGACG--
AGACCAAAACTAATAATTTGTCAACAGCTACAACGGAACCTCTGAGCTCCAGCTGGAGCGCATGA
GCGTCTACTTCAACGAGGTATGTCACAATCAAGAGCAGGAACCCGTGCGCGAGACCTGTTACTAA--
-
CCCCGACTTGCGCAGGCTTCCGGCAACAAGTATGTACCTCGTGCCGTCCTCGTCGATCTCGAGCCC
GGTACCATGGATGCCGTCCGTGCTGGTCCCTTCGGTCAGCTCTTCCGACCCGACAACTTCGTTTTCG
GTCAGTCCGGTGCTGGCAACAACTGGGCCAAGGGTCATTACACTGAGGGTGCCGAGCTCGTTGACC
AGGTTTTGGATGTCGTTCGTCGTGAGGCTGAGGGCTGCGACTGCCTTCAGGGTTTCCAGATTACCCA
CTCGCTTGGTGGTGGTACCGGTGCCGGTATGGGTACCTTGTTGATCTCTAAGATCCGCGAGGAGTTC
CCCGACCGCATGATGGCTACTTTCTCCGTCGTTCCCTCCCCCAAGGTTTCTGACACCGTTGTCGAGC
CTTACAACGCCACCCTCTCTGTCCACCAGCTGGTCGAGAACTCGGACGAGACCTTCTGCATTGATA
ACGAGGCTCTGTACGACATCTGCATGCGTACCCTGAAGCTATCCAACCCCTCGTACGGTGACCTGA
ACCACCTGGTCTCCGCCGTCATGTCCGGTGTTACCACCTGCTTGCGATTCCCTGGCCAGCTGAACTC
TGACCTCCGAAAGTTGGCTGTCAACATGGTGCCCTTCCCTCGTCTGCACTTCTTCATGGTCGGCTTC
GCTCCCCTGACCAGCCGCGGTGCTTACTCCTTCCGTGCTGTCACCGTTCCCGAGTTGACTCAGCAGA
TGTTCGACCCCAAGAACATGATGGCCGCCTCCGACTTCCGCAACGGTCGCTACCTGACGTGCTCTG
CCATCTTGTAAGATACCTGTTCCGATTTGTCGATTAGTGCT--TTTGCTAAC--------------------------------

>Hypoxylon_texense

-
CATTACTGAGTTCTCAAAACTCCCAACCCTTTGTGAACCTTACCGTCGTTGCCTCGGCGCCGAGCGC
GGCTACCCTGGAGCTACCCGGGAGCCACCTACCCCGTAGGTGGCTACCCTGGAGCTACCCTGTAGT
TGCTCTTTACGCTCCGCCGGTGGACCTCTACACTCTGTTTTGTATAGTGTATCTCTGAAACCTATAA
CTTAATACGTTAAAACTTTCAACAACGGATCTCTTGGTTCTGGCATCGATGAAGAACGCAGCGAAA
TGCGATAAGTAATGTGAATTGCAGAATTCAGTGAATCATCGAATCTTTGAACGCATATTGCGCCCA
TTAGTATTCTAGTGGGCATGCCTATTCGAGCGTCATTTCAACCCTTACG-
CCCTGCTGCGTAGTGTTGGGAACCTACAAGCCAAAGTGTAGCTCCCTAAAGGTAGTGGCGGTGTTA
-GGTACACTCGTAGCGTAGTACGTTTTCTCGCTTCTGTAGTGCACCTAG-
GGCCTGCCGTGAAACCCCTTATACTTCTAGTGGTTGACCTCGGATTAGGTAGGAATACCCGCTGAA
CTTAAGCATA--
AATTGTAATTTGTAGAGGATGCTTTTGGTGCGGC-
GCCTTCCGAGTTCCCTGGAACGGGACGCCAGAGAGGGTGAGAGCCCCGTACGGTTGGA-
TACCTAGCCTCTATATAGCTCCTTCGACGAGTCGGGTAGTTTGGGAATGCTGCTCTAAACGGGAGG
TAAATTTCTTCTAAAGCTAAATACCGGCCAGAGACC-
GATAGCGCACAAGTAGAGTGATCGAAAGATGAAAAGCACTTTGAAAAGAGGGTTAAATAGCACGT

GAAATTGTTGAAAGGGAAGCG-TTTGCGACCAGACCTTTTCCGGGCGGATCATCCGGTGTTC--
TCACCGGTGCACTTCGCCCGG--TTTAGGCCAGCATCGGTTTT---
CGTAGGGGGATAAAGGCCTGGGGAACGTATCTC----CTTCGGGAGTGTT-
ATAGCCCCTCGCGTAATACCCTTAC-GGGGACCGAGGACCGCGCT-CT----
GCAAGGATGCTGGCGTAATGGTCGTCAACGACCCGTCTTGAAACACGGACCAAGGAGTCGAACAT
TTGTGCGAGTGTTTGGGTG--TTAAACCCTCACGCGTAATGAAAGTGAACGGAGGTGAGAGCCCTT--
---CG---GGGTGCATCATCGACCGATCCTGATG-
TCTTCGGATGGATTTGAGTAAGAGCATAACTGTTCGGACCCGAAAGATGGTGAACTATGCGTGGAT
AGGGTGAAGCCAGAGGAAACTCTGGTGGAGGCTCGCAGCGGTTCTGACGTGCAAATCGATCGTCA
AATCTGCGCATGGGGGCGAAAGACTTATCGA-
ACCATCTAGTAGCTGGTTACCGCCGAAGTTTCCCTCAGGATAGCAGTGT--TG-
TTTTCAGTTTTATGAGGTAAAGCGAATGATTAGGGACTCGGGGGCGCTTTATTGCCT-
CATCCATTCTCAAACTTTAAATATGT---

CAAGCATTTCGAAATCGCTCTAGCCGCCAAGCCGGCTATTATTACGAATGGCCTCAAGTACTCGCT
CGCCACAGGAAACTGGGGCGATCAGAAGAAGGCCATGAGTTCGACGGCTGGTGTCTCGCAGGTGT
TGAACCGATATACAT------TCGCCTCGA---
CTCTCTCTCATTTGAGAAGAACCAATACGCCCATCGGCCGAGACGGAAAGCTGGCGAAGCCCCGTC
AGCTTCACAATACCCATTGGGGCTTGGTCTGCCCGGCCGAAACGCCCGAGGGCCAGGCCTGCGGTC
TCGTGAAGAATTTGTCATTGATGTGCTCAATCAGTGTCGGTACGTCTACTGATCCTATCGTCGACTA
TATGATCACGAGGAATATGGAGGTTCTTGAGGAGTACGAGCCGCTACGATATCCAGATGCCACCAA
GATCTTTCTCAACGGTTCTTGGATCGGTGTGCATCAGAACCCCAAGGCTCTGGTAAGAGATGTCCA
GAACCTGCGCCGAACCAATCAGATCCCGGCCGAGGTGTCGTTGGTCCGCGATATTCGCGATCGTGA
ATTCAAGATCTTTTCGGATGCCGGTCGGGTCATGCGCCCCCTGTTTGTGGTACAGCAAGAGGAT-----
----
ACCGAGGCTGGCATCAAGAAAGGAACGTTAGCTCTTACCAAGGAGATGATCCAGAGGCTCGAGGC
GGATGTCGATATCGATCCCGATAGCGACGAGTATTTTGGCTGGCAAGGCCTGGTCAACGAAGGTGT
CATCGATTATCTTGACGCCGAGGAAGAAGAGACGGCGATGATATGTATGACGCCCGAGGACCTCG
AGACCTACCGCCAGACCAAGGCCGGTTACGCGGTGTCCCAGGATAACGGG------
GATGAGATTAACAAGCGACTGAGGACCAAGGTTAACCCGACCACCCACATGTACACGCACTGCGA
AATTCATCCTAGTATGCTGCTAGGTATCTGCGCCAGTATTATTCCGTTCCCAGACCACAATCAGGTA
CCCCTGAACGCGTCCCCCAACCCCCTGATTTCCTACCCCTCACGCACAAACACCACAAGCACGAAA
CGTTTCAATTCTCTATATCGCG--------AAACCAC--
CACGATATCTTGGAATAGGAAAGCTAACCATATATCTTCATTTCAATAGGTTCACCTTCAGACCGG
CCAATGCGTAAGAACTACCCACACCAACAACCACCAACCA-
TCGACGCGAAAGAATATGGCGGGGCTCACACGAATTATTTAGGGTAACCAAATCGGTGCTGCTTTC
TGGCAAACCATCTCTGGCGAGCACGGTCTCGACAGCAATGGCGTGTACGTATATGGTTGGGCAATT
CCTCGAAAGGAGTACAAAATTAATAGTTTGTCAACAGCTACAACGGAACCTCTGAGCTCCAGCTGG
AGCGAATGAGTGTCTACTTTAACGAGGTACGCCATAATCAAGACTAGGAATACATGCGCAAATCCC
TTTACTAATTACATCGATTTGCGCAGGCTTCCGGAAACAAGTATGTGCCCCGTGCCGTCCTCGTCGA
TCTCGAGCCCGGCACCATGGATGCCGTCCGTGCTGGTCCTTTCGGTCAGCTCTTCCGACCCGACAAC
TTTGTCTTCGGTCAGTCCGGTGCCGGCAACAACTGGGCCAAGGGTCACTACACTGAGGGTGCCGAG
CTCGTTGACCAGGTTTTGGATGTCGTTCGTCGTGAGGCTGAGGGCTGCGACTGCCTTCAGGGTTTCC
AGATCACCCACTCGCTCGGTGGTGGTACTGGTGCCGGTATGGGTACCCTGTTGATCTCCAAGATCC
GCGAGGAGTTCCCCGACCGCATGATGGCTACTTTCTCCGTCGTGCCTTCCCCCAAGGTCTCTGACAC
CGTCGTCGAGCCTTACAACGCCACTCTCTCTGTCCACCAGCTGGTCGAGAACTCCGACGAGACCTT
CTGCATTGACAACGAGGCTCTGTACGACATCTGCATGCGTACCCTCAAGCTATCCAACCCCTCGTAT
GGTGACCTGAACCACCTGGTCTCGGCCGTTATGTCTGGTGTCACCACCTGCTTGCGATTCCCAGGCC
AGCTGAACTCTGATCTCCGCAAGTTGGCTGTGAACATGGTGCCCTTCCCTCGTCTGCACTTCTTCAT
GGTTGGCTTCGCTCCCCTGACCAGCCGCGGTGCTTACTCTTTCCGTGCCGTCACCGTTCCCGAGTTG
ACTCAGCAGATGTTCGACCCCAAGAACATGATGGCTGCCTCTGACTTCCGCAACGGTCGTTACTTG
ACGTGCTCTGCCATCTTGTAAGATACCTGCTCCAAGTTATTAATATTT---CGTATGCTGACCCT--
AATTCTAGCCGTGGCAAGATCTCCA

>Hypoxylon_guilanense

-
CATTACTGAGTTCTAAAAACTCCCAACCCTTTGTGAACCTTACCGTCGTTGCCTCGGCGCCGAGCGC
GGCTACCCTGGAGCTACCCGGGAGCCACCTACCCTGTAGGTGGCTACCCTGGAGCTACCCTGTAGT
TGCATTCTACGCTCCGCCGGCGGACCTCTACACTCTGTTTTGTATAGTGTATCTCTGAAACCTATAA
CGTAATACGTTAAAACTTTCAACAACGGATCTCTTGGTTCTGGCATCGATGAAGAACGCAGCGAAA
TGCGATACGTAATGTGAATTGCAGAATTCAGTGAATCATCGAATCTTTGAACGCATATTGCGCCCA
TTAGTATTCTAGTGGGCATGCCTATTCGAGCGTCATTTCAACCCTTACGCCCCTGTTGCGTAGTGTT
GGGAACCTACAGGCCAGAGTGTAGCTCCCTAAAGGTAGTGGCGGTGTTA-
GGTACACTCGTAGCGTAGTAACTTTTCTCGCTCCTGCAGTGTACCTAA-
GGCCTGCCGTGAAACCCCCTATACTTCTAGTGGTTGACCTCGGATTAGGTAGGAATACCCGCTGAA
CTTAAGCATATGAAGCGGCAACAGCTCAAA-TTTGAAATCTGGCC----CTC-----
GTGGTCCGAATTGTAATTTGTAGAGGATGCTTTGGGTGCGGT-
GCCTTCCGAGTTCCCTGGAACGGGACGCCAGAGAGGGTGAGAGCCCCGTACGGTTGGC-
CACCTAGCCTCTATATAGCTCCTTCGACGAGTCGGGTAGTTTGGGAATGCTGCTCTAAATGGGAGG
TAAATTTCTTCTAAAGCTAAATACCGGCCAGAGACC-
GATAGCGCACAAGTAGAGTGATCGAAAGATGAAAAGCACTTTGAAAAGAGGGTTAAATAGCACGT
GAAATTGTTGAAAGGGAAGCG-TTTACGACCAGACCTTCTCCAGGCGGATCATCCGGTGTTC--
TCACCGGTGCACTTCGTCTGG--TGTAGGCCAGCATCGGTTTT---
CGTAAGGGGATAAAGGCCTGGGGAACGTATCTC----CTTCGGGAGTGTT-
ATAGCCCCTCGCGTAATACCCTTAC-GGGGACCGAGGACCGCGCT-CTAC--
GCAAGGATGCTGGCGTAATGGTCGTCAACGACCCGTCTTGAAACACGGACCAAGGAGTCGAACAT
TTGTGCGAGTGTTTGGGTG--GTAAACCCTCACGCGTAATGAAAGTGAACGGAGGTGAGAGCCCCT--
--CCG---GGGTGCATCATCGACCGATCCTGATG-
TCTTCGGATGGATTTGAGTAAGAGCATAACTGTTCGGACCCGAAAGATGGTGAACTATGCGTGGAT
AGGGTGAAGCCAGAGGAAACTCTGGTGGAGGCTCGCAGCGGTTCTGACGTGCAAATCGATCGTCA
AATCTGCGCATGGGGGCGAAAGACTTATCGA-
ACCATCTAGTAGCTGGTTACCGCCGAAGTTTCCCTCAGGATAGCAGTGT--TG-
TTTTCAGTTTTATGAGGTAAAGCGAATGATTAGGGACTCGGGGGCGCTTTATTGCCTTCATCCATTC
TCAAACTTTAAATATGTAAGAAGCCCTTGTTACTTAACTGAACGTGGGCATTCGAATGTATCAACA
CTAGTGGGCCATTTTTGGTAAGCAGAACTGGCGATGCGGGATGAACCGAACGTGGGGTTAAGGTG
CCAGAGTGGACGCTCATCAGACACCACAAAAGGTGTTAGTACATCTAGACAGT-
GGACGGTGGCCATGGAAGTCGGAATCCGCTAAGGACTGTGTAACAACTCACCAACCGAATGTACT
AGCCCTGAAAATGGATGGCGCTCAAGCGTCT-
CACCCATACCTCACCCTTAGGGTAGAAACGATGCCCTAAGGGCGATGCATCGACACCAACAAACAT
TTCGAAATCGCCCTAGCCGCCAAGCCGGCTATTATTACGAATGGCCTCAAGTACTCGCTCGCTACA
GGAAACTGGGGCGATCAGAAGAAGGCCATGAGTTCGACAGCTGGTGTCTCGCAGGTCCTGAACCG
ATATACAT------TCGCCTCGA---
CTCTCTCTCATTTGAGAAGAACCAATACGCCCATTGGCCGAGACGGAAAGCTGGCGAAGCCCCGTC
AGCTTCACAATACCCATTGGGGCTTGGTCTGCCCGGCCGAAACGCCCGAGGGTCAGGCTTGCGGTC
TCGTGAAGAACTTGTCATTGATGTGCTCAATCAGTGTCGGTACGTCTACTGACCCTATCGTCGACTA
TATGATCACTAGAAATATGGAGGTTCTCGAGGAGTACGAACCGCTACGATATCCCGATGCCACCAA
GATCTTTCTCAACGGTTCTTGGATCGGTGTCCATCAGAACCCCAAGGCTCTGGTGAGAGATGTCCA
GAATCTGCGTCGAACCAATCAGATCCCGGCCGAGGTGTCGTTGGTCCGCGACATCCGCGATCGTGA
ATTCAAGATCTTTTCGGATGCCGGTCGGGTCATGCGCCCCCTGTTTGTAGTCCAGCAAGAGGAT------
----
ACCGAGGCTGGCATTAAGAAGGGAACGTTAGCTCTTACCAAGGAGATGATCCAGAGGCTCGAGGC
GGATGTCGATATCGATCCCGATAGCGATGAGTATTTTGGCTGGCAGGGTCTGGTCAACGAAGGTGT
CATCGATTATCTTGACGCCGAGGAAGAAGAGGGGGCGATGATATGTATGACGCCCGAGGACCTCG
AGACCTACCGCCAAACCAAGGCCGGTTACGCGGTGTCCCAGGATAACGGG------
GATGAGATTAACAAGCGACTGAGGACCAAGGTTAACCCGACCACCCACATGTACACGCACTGCGA
GATTCATCCTAGTATGCTCCTAGGTATCTGCGCCAGTATTATTCCGTTCCCGGATCATAATCAGGTA
CCCCTGAACGCGTCCCCCAACCCCCTGATTTCCTACCCCTCACGCACAAACACCACAAGCACGAAA
CGTTTCAATTCTCTATATCGCG--------AAACCAC--
CACGATATCTTGGAATATGAAAGCTAACCATATATCTTCATTTCAATAGGTTCACCTTCAGACCGGC
CAATGCGTAAGAACTACCTACACCAACAACCACCAACCA-
TCGACGCGAAAGAATATGGCGGGGCTCACACGAATTATTTAGGGTAACCAAATCGGAGCTGCTTTC
TGGCAAACCATCTCTGGCGAGCACGGTCTCGACAGCAATGGCGTGTACGTATATGATTGGGCAATT

CCTCGAAAGGAGTACAAAATTAATAGTTTATCAATAGCTACAACGGAACCTCTGAGCTCCAACTGG
AGCGAATGAGCGTCTACTTCAACGAGGTATGCCATAATTAAGCCTAGAAATACATGCGCAAAACCC
TTTACTAATTACATCGATTCGCGCAGGCTTCCGGAAACAAGTATGTGCCTCGTGCCGTCCTCGTCGA
TCTCGAGCCCGGCACCATGGATGCCGTTCGTGCTGGTCCCTTCGGTCAGCTCTTCCGACCCGACAAC
TTTGTCTTCGGTCAGTCCGGTGCCGGCAACAACTGGGCGAAGGGTCACTACACTGAGGGTGCCGAG
CTCGTTGACCAGGTTTTGGATGTCGTTCGTCGTGAGGCTGAGGGCTGCGATTGCCTTCAGGGTTTCC
AGATCACCCACTCGCTCGGAGGTGGTACTGGTGCCGGTATGGGTACCCTGTTGATCTCCAAGATCC
GTGAGGAGTTCCCCGACCGCATGATGGCCACTTTCTCCGTTGTGCCTTCCCCCAAGGTCTCTGACAC
CGTCGTCGAGCCTTACAACGCCACTCTTTCTGTCCACCAGCTGGTCGAGAACTCTGACGAGACCTTC
TGCATTGACAACGAGGCTCTGTACGACATCTGCATGCGTACCCTCAAGCTATCCAACCCCTCGTAT
GGTGATCTGAACCATCTGGTCTCCGCTGTCATGTCCGGTGTCACCACCTGTCTGCGATTCCCTGGCC
AGCTGAACTCTGATCTCCGCAAGTTGGCCGTGAACATGGTGCCCTTCCCTCGTCTGCACTTCTTCAT
GGTTGGCTTTGCTCCCCTGACCAGCCGCGGTGCTTACTCTTTCCGTGCCGTCACCGTTCCCGAGTTG
ACTCAGCAGATGTTCGACCCCAAGAACATGATGGCTGCTTCTGACTTCCGCAACGGTCGTTACCTG
ACGTGCTCCGCCATCTTGTAAGAAACCTGCTCCGAGTCGTTAATGAATGCT-------------------------------
----

>Hypoxylon_zangii_FCATAS4029

-
CATTACTGAGTTCTCAAAACTCCCAACCCTTTGTGAACCTTACCGTCGTTGCCTCGGCGCTGAGCGC
GGCTACCCGGGAGCTACTCGGGAGCCACCTACCCTGTAGGTGGCTACCCGGGAGCTACCCTGTAGT
TGCACTTCACGCTCCGCCGGTGGACCTCTACACTCTGTTTATTATAGTGTATCTCTGATATTCT-
AACTTAATACGTTAAAACTTTCAACAACGGATCTCTTGGTTCTGGCATCGATGAAGAACGCAGCGA
AATGCGATAAGTAATGTGAATTGCAGAATTCAGTGAATCATCGAATCTTTGAACGCATATTGCGCC
CATTAGTATTCTAGTGGGCATGCCTATTCGAGCGTCATTTCAACCCTTAAG-
CCCTGTTGCTTAGTGTTGGGAACCTACAGGCCAAGACGTAGCTCCCTAAAGGTAGTGGCGGTGTTA-
GGTACACTCGTAGCGTAGTAAATTTTCTCGCTCCTGCAGTGTACCTAG-
GGCCTGCCGTGAAACCCCCTATACTTCTAGTGGTTGACCTCGGATTAG---------------------------
TGAAGCGGCAACAGCTCAAA-TTTGAAATCTGGCC----CTC-----
GTGGTCCGAATTGTAATTTGTAGAGGATGCTTTTGGTGCGGT-
GCCTTCCGAGTTCCCTGGAACGGGACGCCAGAGAGGGTGAGAGCCCCGTACGGTTGGA-
CACCTACCCTATATATAGCTCCTTCGACGAGTCGGGTAGTTTGGGAATGCTGCTCTAAATGGGAGG
TAAATTTCTTCTAAAGCTAAATACCGGCCAGAGACC-
GATAGCGCACAAGTAGAGTGATCGAAAGATGAAAAGCACTTTGAAAAGAGGGTTAAATAGCACGT
GAAATTGTTGAAAGGGAAGCG-TTTGCGACCAGACCTTTTCCAGGCGGATCATCCGGTGTTC--
TCACCGGTGCACTTCGCCTGG--TTTAGGCCAGCATCGGTTCT---
CGTAGGGGGATAAAGGCCTGGGGAACGTATCTC----CTTCGGGAGTGTT-
ATAGCCCCTCGCGTAATACCCTTAC-GGGGACCGAGGACCGCGCT-CT----
GCAAGGATGCTGGCGTAATGGTCGTCAACGACCCGTCTTGAAACACGGACCAAGGAGTCGAACAT
TTGTGCGAGTGTTTGGGTA--TCAAACCCTCACGCGTAATGAAAGTGAACGGAGGTGAGAGCCCTT--
---CG---GGGTGCATCATCGACCGATCCTGATG-
TCTTCGGATGGATTTGAGTAAGAGCATAACTGTTCGGACCCGAAAGATGGTGAACTATGCGTGGAT
AGGGTGAAGCCAGAGGAAACTCTGGTGGAGGCTCGCAGCGGTTCTGACGTGCAAATCGATCGTCA
AATCTGCGCATGGGGGCGAAAG--
---AGGCCCTCTCTCCTCTACAGGATGCAG-------------------------------------
--TAGACAGT----------

GCGATGCATCGATACCAACAAACATTTCGAAATCGCCCTGGCCGCCAAACCGGCTATTATTACCAA
CGGCCTGAAGTACTCGCTCGCTACAGGAAACTGGGGCGATCAGAAGAAGGCCATGAGTTCGACGG
CTGGTGTCTCGCAGGTCTTGAACCGATATACGT------TCGCTTCGA---
CTCTGTCTCATTTGAGAAGAACCAATACGCCCATCGGCCGAGACGGAAAGCTGGCGAAACCCCGTC
AGCTTCACAATACCCATTGGGGCTTGGTCTGCCCGGCCGAAACGCCCGAGGGTCAGGCCTGCGGTC
TCGTGAAGAACTTGTCATTGATGTGCTCAATCAGTGTCGGTACGTCTACTGACCCTATCGTCGACTA
TATGATCACTAGGAACATGGAGGTTCTTGAGGAGTACGAACCGCTGCGATATCCCGATGCAACCAA
AATTTTCCTCAACGGTTCTTGGATCGGTGTCCATCAGAACCCCAAGGCTCTAGTGAGAGACGTCCA
GAATCTGCGCCGAACCAATCAGATCCCGGCCGAGGTGTCGTTGGTTCGCGACATTCGCGATCGTGA
ATTCAAGATCTTTTCGGATGCCGGTCGGGTCATGCGCCCCCTGTTTGTAGTACAGCAGGAGGAT-----

----
ACCGAGGCTGGCATTAAGAAGGGAACGCTAGCTCTTACCAAGGAGATGATCCAGAGGCTCGAGGC
GGATGTTGATATCGACCCCGATAGCGATGAGTATTTTGGTTGGCAAGGTCTAGTCAACGAAGGTGT
CATCGATTATCTTGACGCCGAGGAAGAAGAGGGGGCGATGATATGTATGACACCCGAGGATCTCG
AGACCTACCGCCAGACCAAGGCCGGTTACGCTGTGTCCCAGGATAACGGG------
GATGAGATTAACAAGCGACTGAGGACCAAGGTTAACCCGACCACGCACATGTACACCCACTGCGA
GATTCATCCTAGCATGCTTCTAGGTATTTGCGCGAGTATTATTCCGTTCCCGGATCATAATCA----
CCCCTGAACGCGTCCCCCAACCCCCTGATTTCCTACCCCTCACGCACAAACACCACAAGCACGAAA
CGTTCCAGTTCTTGATATCGCG--------AAACCAT--CATAA--
TCTTGGAATATGAAAGCTAACCATATATCTTCATTCCAATAGGTTCACCTTCAGACTGGCCAATGCG
TAAGAACTACCTACACCAAAAACCGCCAATCA-
TCGACGCGAAAGAATATGGCGGGGCTCACATGAATTATTTAGGGTAACCAAATTGGTGCTGCTTTC
TGGCAAACCATCTCGGGCGAGCACGGTCTCGACAGTAATGGCGTGTACGTATTTGGGTTGGGAATT
GTTTAAAGAGAGTACAAAATTAATAGTTTGTCAACAGCTACAACGGAACCTCTGAGCTCCAACTGG
AGCGCATGAGCGTTTACTTCAACGAGGTATGCCACCATTACGAGCGAAAATACACGCGCCAACAC
ATTTACTGATTACTCTGATATGCACAGGCTTCCGGAAACAAGTATGTGCCCCGTGCTGTCCTTGTCG
ATCTCGAGCCCGGTACCATGGATGCCGTCCGTGCTGGTCCTTTCGGTCAGCTCTTCCGACCCGACAA
CTTCGTCTTCGGCCAATCCGGTGCCGGCAACAACTGGGCGAAGGGTCACTACACTGAGGGTGCCGA
GCTCGTTGACCAGGTTTTGGATGTCGTTCGTCGCGAGGCCGAGGGCTGCGACTGCCTCCAGGGTTT
CCAGATCACCCACTCGCTCGGTGGTGGTACCGGTGCCGGTATGGGTACCCTGTTGATCTCCAAGAT
CCGTGAGGAGTTCCCCGACCGCATGATGGCCACTTTCTCCGTCGTGCCTTCCCCCAAGGTCTCTGAC
ACCGTCGTCGAGCCGTACAATGCCACTCTCTCCGTTCACCAGCTGGTCGAGAACTCGGACCAGACC
TTCTGCATTGATAACGAGGCTCTGTACGACATCTGCATGCGTACCCTAAAGCTATCCAACCCCTCGT
ATGGTGACCTGAACCACCTGGTCTCGGCCGTCATGTCTGGTGTCACCACCTGTTTGCGATTCCCTGG
CCAGCTGAACTCTGACCTTCGCAAGTTGGCTGTGAACATGGTGCCCTTCCCTCGTCTGCACTTCTTT
ATGGTCGGCTTCGCCCCCCTGACCAGCCGCGGTGCTTACTCTTTCCGTGCCGTCACCGTTCCCGAGT
TGACTCAGCAGATGTTTGACCCCAAGAACATGATGGCTGCCTCTGACTTCCGCAACGGTCGTTACC
TAACATGCTCTGCCATCTTGTAAGATACCTGTTCCGAGCCGTTGATAGGTGCTTATATGCTAACCCC
CAAATTCTAGCCGTGGCAAGATCTCCA

>Hypoxylon_rubiginosum

-
CATTACTGAGTTCTAAAAACTCCCAACCCTTTGTGAACCTTACCGTCGTTGCCTCGGCGCCGAGCGC
GGCTACCCGGGAGCTACCCTGTAGACCACTACCCTGTAGGTGCCTACCCTGTAGCTACCCTGTAGTT
GCACTTTACGCTCCGCCGGTGGACTACCCAACTCTGTTTTTAATGGTGTATCTCTGAAAACAAAAAC
AAAATAAGTTAAAACTTTCAACAACGGATCTCTTGGTTCTGGCATCGATGAAGAACGCAGCGAAAT
GCGATAAGTAATGTGAATTGCAGAATTCAGTGAATCATCGAATCTTTGAACGCATATTGCGCCCAC
TAGTATTCTGGTGGGCATGCCTATTCGAGCGTCATTTCAACCCTTAAG-
CCCTGTTGCTTAGTGTTAGGAGCCTACTACCTACGGCGTAGCTCCCTAAAGGCAGTGGCGGTGTTA-
GGTACACTCGTAGCGTAGTAATTTTTCTCGCTCCTGCTGTGTACCTAG-
GGCCTGCCGTAAAACCCCT-ATACTTCTAGTG---
TGAAGCGGCAACAGCTCAAA-TTTGAAATCTGGCC----CTA-----
GCGGTCCGAATTGTAATTTGTAGAGGATGCTTTTGGTGCGGT-
GCCTTCCGAGTTCCCTGGAACGGGACGCCAGAGAGGGTGAGAGCCCCGTACGGTTGGA-
CACCTACCCTATATATAGCTCCTTCGACGAGTCGAGTAGTTTGGGAATGCTGCTCTAAATGGGAGG
TAAATTTCTTCTAAAGCTAAATACCGGCCAGAGACC-
GATAGCGCACAAGTAGAGTGATCGAAAGATGAAAAGCACTTTGAAAAGAGGGTTAAATAGCACGT
GAAATTGTTGAAAGGGAAGCG-TTTACGACCAGACCTCTTCCAGGCGGATCATCCGGTGTTC--
TCACCGGTGCACTTCGTCTGG--TTTAGGCCAGCATCGGTTTT---
CGTAGGGGGATAAAGACCTGGGGAACGTATCTC----CTTCGGGAGTGTT-
ATAGCCCCTCGTGTAATACCTCTAC-GGGGACCGAGGACCGCGCA-CT----
GCAAGGATGCTGGCGTAATGGTCGTCAACGACCCGTCTTGAAACACGGACCAAGGAGTCGAACAT
TTGTGCGAGTGTTTGGGTA--TTAAACCCTCACGCGTAATGAAAGTGAACGGAGGTGAGAGCCCTT--
---CG---GGGTGCATCATCGACCGATCCTGATG-
TCTTCGGATGGATTTGAGTAAGAGCATAACTGTTCGGACCCGAAAGATGGTGAACTATGCGTGGAT
AGGGTGAAGCCAGAGGAAACTCTGGTGGANGCTCGCAGCGGTTCTGACGTGCAAATCGATCGTCA
AATCTGCGCATGGGGGCGAAAGACTTATCGA-

ACCATCTAGTAGCTGGTTACCGCCGAAGTTTCCCTCAGGATAGCAGTGT--TG-
TGTTCAGTTTTATGAGGTAAAGCGAATGATTAGGGACTCGGGGGCGCTTTATTGCCTTCATCCATTC
TCAAACTTTAAATATGTAAGAAGCCCTTGTTACTTAATTGAACGTGGGCATTCGAATGTATCAACA
CTAGTGGGCCATTTTTGGTAAGCAGAACTGGCGATGCGGGATGAACCGAACGTGGGGTTAAGGTG
CCAGAGTGGACGCTCATCAGACACCACAAAAGGTGTTAGTACATCTAGACAGTAGGACGGTGGCC
ATGGAAGTCGGAATCCGCTAAGGACTGTGTAACAACTCACCAACCGAATGTACTAGCCCTGAAAAT
GGATGGCGCTCAAGCGTCT-CACCCATACCTCACCCTTAGGGTAGAAACGATGCCCTAAGG-
CGCTGCATCGACACTAACAAGCATTTTGAAATCGCCCTAGCCGCCAAGCCGGCTATTATTACGAAC
GGCCTCAAGTACTCGCTCGCTACAGGAAACTGGGGCGATCAGAAGAAGGCCATGAGTTCAACGGC
CGGTGTGTCGCAGGTCCTGAATCGATACACGT------TCGCCTCGA---
CGCTTTCTCATTTGAGAAGAACCAATACGCCCATCGGGCGAGACGGAAAGCTTGCGAAGCCCCGTC
AGCTTCACAATACTCATTGGGGCCTGGTCTGCCCGGCCGAAACGCCCGAGGGTCAGGCCTGCGGTC
TGGTGAAGAACTTATCATTGATGTGTTCAATCAGCGTCGGTACGTCCACGGATCCTATCGTGGATTA
TATGATTACTAGGAATATGGAGGTTCTCGAGGAGTACGAGCCGCTGCGATATCCCGATGCTACCAA
GATCTTCCTGAACGGCTCTTGGATCGGTGTACATCAGAACCCCAAGGCTCTGGTGAGAGATGTCCA
GAATCTGCGCCGAACTAATCAGATCCCGTCCGAGGTGTCGTTGGTCCGCGACATTCGCGATCGTGA
ATTCAAGATCTTTTCGGATGCCGGTCGGGTCATGCGCCCCTTGTTTGTGGTACAGCAAGAGGAT------
----
ACCGAAGCTGGCGCCAAGAAGGGAACGTTAGCTCTTACCAAGGACATGATCCAGAGGCTCGAGGC
GGATGTCGAAATAGATCCCGACAGTGATGAGTATTTTGGCTGGCAAGGTTTGGTCAACGAAGGTGT
CATCGATTATCTCGACGCCGAGGAAGAAGAGGGGGCGATGATATGTATGACGCCCGAGGACCTCG
AGACCTACCGCCAGACCAAGGCCGGATACGCGGTGTCTCAGGATAACGGG------
GATGAGATTAACAAGCGACTGAGGACCAAGGTTAACCCGACCACGCACATGTACACTCACTGCGA
GATTCATCCTAGCATGCTCTTGGGTATTTGCGCCAGCATCATTCCATTC------------------
CCCCTGAACGCGTCCCCCAATCCCCTGATTTCCTACCCCTCACGCACAAACACCAGAGGCACGAAA
CGTCTCCATTGCCAATTGCGTA-TATCGCGAAA-----
CCACAAGACCTTGGAATACAAAAGCTAACCATACATCTTTATTTCGATAGGTTCATCTTCAGACCG
GCCAATGCGTAAGAATTACCTATACCAAGAACCACCGATCA-
ACAACGCGAAAAGATATGGCGGGGCTCACATGAATTTGTTAGGGTAACCAAATTGGTGCTGCTTTC
TGGCAAACCATCTCTGGCGAGCACGGTCTCGACAGCAATGGCGTGTATGTGTTTGACCAGCGAATT
CCCCGACGAGAGTTCAAAATTAATATTTGTCAACAGCTACAACGGAACCTCTGAGCTCCAGCTGG
AGCGCATGAGCGTCTACTTCAACGAGGTACGCCACAATCAAATCCGACAATCCATGCGCAGGACTC
GTTACTAATTACTTCGATATGCGCAGGCTTCCGGAAACAAGTATGTGCCCCGTGCCGTCCTCGTCGA
TCTCGAGCCTGGTACCATGGATGCCGTCCGCGCTGGTCCCTTCGGTCAGCTCTTCCGACCCGACAAC
TTCGTTTTCGGTCAGTCCGGTGCTGGCAACAACTGGGCCAAGGGTCACTACACTGAGGGTGCCGAA
CTCGTTGACCAGGTTTTGGATGTCGTTCGTCGTGAGGCTGAGGGCTGCGACTGCCTTCAGGGTTTCC
AGATCACTCACTCGCTCGGTGGTGGTACCGGTGCCGGTATGGGTACCTTGTTGATCTCCNAGATCC
GCGAGGANTTCCCCGACCGCATGATGGCTACTTTCTCCGTCGTCCCCTCCCC------------------------------
----

>Hypoxylon_laschii_MUCL52796

TTGTGAACCTTACCGTCGTTGCCTCGGCGCCGAGCGCGGCTACCCTGGAGCTACCCTGTACCTGCG
AAGTTACTAAGTAGCTACCTTGTAGCTACCCTGGAGTTGCACTCTACGCTCCGCCGGAGGACCACT
ACACTCTGT-TTTTCTAGTGTATCTCTGAAATCTT-
AACTTAATACGTTAAAACTTTCAACAACGGATCTCTTGGTTCTGGCATCGATGAAGAACGCAGCGA
AATGCGATAAGTAATGTGAATTGCAGAATTCAGTGAATCATCGAATCTTTGAACGCATATTGCGCC
CATTAGTATTCTAGTGGGCATGCCTATTCGAGCGTCATTTCAACCCTTAAG-
CCCTGTAGCTTAGCGTTGGGAACTACGGCCCTGGGGCGTAGCTCCCTAAAGGTAGTGGCGGTGTTA-
GGTACACTCGTAGCGTAGTAGCTCTTCTCG---
--------------------TGAAGCGGCAACAGCTCAAA-TTTGAAATCTGGCC----CTC-----
GTGGTCCGAGTTGTAATTTGTAGAGGATGCTTTTGGTGCGGT-
ACCTTCCGAGTTCCCTGGAACGGGACGCCAGAGAGGGTGAGAGCCCCGTACGGTCGGA-
TACCTACCCTATATATAGCTCCTTCGACGAGTCGGGTAGTTTGGGAATGCTGCTCTAAATGGGAGG

TAAATTTCTTCTAAAGCTAAATACCGGCCAGAGACC-
GATAGCGCACAAGTAGAGTGATCGAAAGATGAAAAGCACTTTGAAAAGAGGGTTAAATAGCACGT
GAAATTGTTGAAAGGGAAGCG-TTTACGACCAGACCTCTTCCAGGCGGATCATCCGGTGTTC--
TCACCGGTGCACTTCGCCTGG--TTTAGGCCAGCATCGGTTTT---
CGTAGGGGGATAAAGGCCTGGGGAACGTATCTC----TCTAGGGAGTGTT-
ATAGCCCCTCGTGTAATACCCTTAC-GGGGACCGAGGACCGCGCT-CT----
GCAAGGATGCTGGCGTAATGGTCGTCAACGACCCGTCTTGAAACACGGACCAAGGAGTCGAACAT
TTGTGCGAGTGTTTGGGTA--TAAAACCCTCACGCGTAATGAAAGTGAACGGAGGTGAGAGCCCTT--
---CG---GGGTGCATCATCGACCGATCCTGATG-
TCTTCGGATGGATTTGAGTAAGAGCATAACTGTTCGGACCCGAAAGATGGTGAACTATGCGTGGAT
AGGGTGAAGCCAGAGGAAACTCTGGTGGAGGCTCGCAGCGGTTCTGACGTGCAAATCGATCGTCA
AATCTGCGCATGGGGGCGAAAGACTTATCGA-
ACCATCTAGTAGCTGGTTACCGCCGAAGTTTCCCTCAGGATAGCAGTGT--TG-
TTTTCAGTTTTATGAGGTAAAGCGAATGATTAGGGACTCGGGGGCGCTTTATTGCCTTCATCCATTC
TCAAACTTTAAATATGTAAGAAGCCCTTGTTACTTTATTGAACGTGGGCATTCGAATGTATCAACAC
TAGTGGGCCATTTTTGGTAAGCAGAACTGGCGATGCGGGATGAACCGAACGCGGGGTTAAGGTGC
CAGAGTGGACGCTCATCTGACACCACAAAAGGTGTTAGTACATCTAGACAGT-
GGACGGTGGCCATGGAAGTCGGAATCCGCTAAGGACTGTGTAACAACTCACCAACCGAATGTACT
AGCCCTGAAAATGGATGGCGCTCAAGCGTCT-
CACCCATACCTCGCCCTTAGGGTAGAAACGATGCCCTAAGGGCGCTGCATCGACACCAACAAGCAT
TTCGAAATCGCCCTAGCCGCCAAGCCGGCTATCATTACGAACGGCCTCAAGTACTCGCTCGCTACG
GGAAACTGGGGCGATCAGAAGAAGGCTATGAGCTCGACGGCTGGTGTGTCGCAGGTCCTGAACCG
ATATACGT------TCTCCTCGA---
CGCTTTCTCACTTGAGAAGAACCAATACGCCCATCGGGCGAGACGGAAAGTTGGCCAAGCCCCGTC
AGCTTCACAACACCCATTGGGGCCTGGTATGCCCGGCCGAAACGCCCGAGGGCCAGGCCTGCGGC
CTCGTGAAGAACTTGTCATTGATGTGTTCAATCAGTGTCGGTACGTCTACTGACCCTATCGTCGACT
ATATGATCACTAGAAATATGGAGGTTCTCGAGGAGTACGAGCCGTTGCGATATCCCGATGCCACCA
AGATCTTCCTCAACGGCTCTTGGATCGGTGTCCACCAGAACCCCAAAGCTCTGGTGAGAGACGTCC
AGAATCTGCGCCGAACCAATCAGATCCCGGCCGAGGTGTCGTTGGTCCGCGACATTCGCGATCGTG
AATTCAAGATCTTTTCGGATGCCGGCCGGGTCATGCGCCCCTTGTTTGTAGTACAGCAAGAGGAT---
----
ACCGAGACTGGTCCTAAGAAGGGAACGTTGGCTCTTACCAAGGAGATGATCCAGAGGCTGGAGGC
GGATGTCGATATAGACCCCGATAGCGATGAGTATTTCGGCTGGCAAGGTCTGGTCAACGAAGGTGT
CATCGATTATCTCGACGCCGAGGAAGAAGAGACGGCGATGATATGTATGACGCCCGAGGATCTCG
AGACCTACCGCCAGGCCAAGGCCGGTTACGCCGTGTCCCAGGATAACGGG------
GATGAGATTAACAAGCGACTGAGGACCAAGGTTAATCCGACCACGCACATGTATACCCACTGCGA
GATCCATCCCAGCATGCTGCTAGGGATTTGCGCAAGTATTATTCCGTTCCCGGATCACAATCAGGT
ACCCCTGAACGCGTTCCCCAATCCCCTGATCTCCTACCCCTCACGCACAAACACCACAAGCACGAA
ACGTTCCGATTCTCTGTATCGCG--------AAACCAC--CTTGA--
ACTTGAAAAATGAAAGCTAACCATATATCTTCATCTGGATAGGTTCACCTTCAGACCGGCCAATGC
GTAAGAACTACCTACATCAATAACCACCCATCA-
TCAACGCGAAAGAACATAGCGGGGCTCACATGGATTTTTTAGGGTAACCAAATTGGTGCTGCTTTC
TGGCAAACCATCTCTGGCGAGCACGGTCTCGACAGCAATGGCGTGTACGTATCTGGCTGGGCAATC
CCTCAACAGGAGTGCAAAATTAATATTTCGTCAACAGCTACAACGGAACCTCTGAGCTCCAACTGG
AGCGCATGAGCGTCTACTTCAACGAGGTACGCCACAATCAAGATCTGGAATACATGCGCAAGACTC
GTTACTGATTACTTGGATATGCGTAGGCTTCCGGCAACAAGTACGTACCCCGTGCTGTTCTCGTCGA
TCTCGAGCCCGGTACCATGGATGCCGTCCGTGCTGGTCCTTTCGGTCAGCTCTTCCGACCCGACAAC
TTCGTCTTCGGTCAGTCGGGTGCTGGCAACAACTGGGCCAAGGGTCACTACACTGAGGGTGCCGAG
CTCGTTGACCAGGTTTTGGATGTCGTTCGTCGCGAGGCCGAGGGCTGCGACTGCCTTCAGGGTTTCC
AGATCACCCACTCGCTCGGTGGTGGTACCGGTGCCGGTATGGGTACCTTGTTGATCTCCAAGATCC
GCGAGGAGTTCCCCGACCGCATGATGGCTACTTTCTCCGTCGTGCCCTCCCCCAAGGTCTCTGACAC
GGTCGTCGAGCCTTACAACGCCACCCTCTCGGTCCACCAGCTGGTCGAGAACTCGGACGAGACCTT
CTGCATTGACAACGAGGCTCTGTACGACATCTGCATGCGTACCCTGAAGCTATCCAACCCCTCGTA
TGGTGACCTGAACCACCTAGTCTCGGCCGTCATGTCCGGTGTCACCACCTGCTTGCGATTCCCCGGC
CAGCTGAACTCTGATCTCCGCAAGTTGGCTGTCAACATGGTGCCCTTCCCTCGTCTGCACTTCTTCA
TGGTCGGCTTCGCTCCTCTGACCAGCCGCGGTGCTTACTCCTTCCGTGCTGTCACCGTTCCCGAGTT
GACTCAGCAGATGTTCGACCCCAAGAACATGATGGCTGCTTCTGACTTCCGCAACGGTCGTTACCT

GACGTGCTCTGCCATCTTGTAAGATACCCGCCCCAAGTCAGTAACAAGTGCTTATTYGCTGACCCT--
AACTCTAGCCGTGGCAAGATCTCCA

>Hypoxylon_musceum

-
CATTACTGAGTTCTAAAAACTCCCAACCCTTTGTGAATTCTACTCTTGTTGCCTCGGCGCCGAGCGC
AGCTACCCGGGAGCTACCCGGGAGTCAGCTACCCTGTAGTTGGCTACCCGGTAGCTACCCTGTAGA
TGCGC-
CTACGCTCCGCCGGTGGACCATGCAACTCTTGTTTTTATAGTGTATCTCTGAACCATATAACTAAAT
TCGTTAAAACTTTCAACAACGGATCTCTTGGTTCTGGCATCGATGAAGAACGCAGCGAAATGCGAT
AAGTAATGTGAATTGCAGAATTCAGTGAATCATCGAATCTTTGAACGCATATTGCGCCCATTAGTA
TTCTAGTGGGCATGCCTATTCGAGCGTCATTTCAACCCTTAAG-
CCCTGTTGCTTAGTGTTGGGAATCTACCCTGTGGGAGGTAGTTCCCGAAAATCAGTGGCGGTGTTC-
GGTACACACATAGCGTAGTAAATTATCTCGCTTCTGTAGTGGCCTGAATATCCCGCCGTGAAACCC
CCTA--TTTTCCATGGTTGACCTCGGATTAGGTAGGAATACCCGCTGAACTTAAGC----------------------
-TTGAAATCTGGCC----CTC-----G-GGTCCGAATTGTAATTTGTAGAGGATGCTTTTGGTGCGGT-
GCCTTCCGAGTTCCCTGGAACGGGACGCCATAGAGGGTGAGAGCCCCGTACGGTTGGA-
AGCCAAGCCTATATATAGCTCCTTCGACGAGTCGAGTAGTTTGGGAATGCTGCTCTAAATGGGAGG
TAAATTTCTTCTAAAGCTAAATACCGGCCAGAGACC-
GATAGCGCACAAGTAGAGTGATCGAAAGATGAAAAGCACTTTGAAAAGAGGGTTAAATAGCACGT
GAAATTGTTGAAAGGGAAGCG-TTTATGACCAGACCTCTTCCAGGCGGATCATCCGGTGTTC--
TCACCGGTGCACTTCGTCTGG--TTTAGGCCAGCATCGGTTTC---
CTTAGGGGGATAAAAGCCTGGGGAATGTGGCTC----TTTCGAGAGTGTT-
ATAGCCCCTAGCATAATACCCTTCA-GGGGACCGAGGACCGCGCT-TCG---
GCATGGATGCTGGCGTAATGGTTATCAACGACCCGTCTTGAAACACGGACCAAGGAGTCGAACATT
TGTGCGAGTGTTTGGGTG--TTAAACCCTCACGCGTAATGAAAGTGAACGGAGGTGAGAGCCCCT----
-CG---GGGTGCATCATCGACCGATCCTGATG-
TCTTCGGATGGATTTGAGTAAGAGCATAACTGTTCGGACCCGAAAGATGGTGAACTATGCGTGGAT
AGGGTGAAGCCAGAGGAAACTCTGGTGGAGGCTCGCAGCGGTTCTGACGTGCAAATCGATCGTCA
AATCTGCGCATGGGGGCGAAAGACTTATCGA-
ACCATCTAGTAGCTGGTTACCGCCGAAGTTTCCCTCAGGATAGCAGTGT--TG-
TATTCAGTTTTATGAGGTAAAGCGAATGATTAGGGACTCGGGGGCGCTATATTGCCTTCATCCATTC
TCAAACTTTAAATATGTAAGAAGCCCTTGTTACTTCATTGAACGTGGGCATTCGAATGTATCAACAC
TAGTGGGCCATTTTTGGTAAGCAGAACTGGCGATGCGGGATGAACCGAACGCGAGGTTAAGGTGC
CAGAGTGGACGCTCATCAGACACCACAAAAGGTGTTAGTACATCTAGACAGT-
GGACGGTGGCCATGGAAGTCGGAATCCGCTAAGGACTGTGTAACAACTCACCAACCGAATGTACT
AGCCCTGAAAATGGATGGCGCTCAAGCGTCT-
CACCCATACCTCGCCCTTAGGGTAGAAACGATGCCTTAAGG--

CGCTTTCTCACTTGAGAAGAACCAACACGCCTATTGGGCGAGATGGAAAGTTGGCGAAGCCCCGGC
AGCTTCACAATACCCATTGGGGTCTAGTCTGCCCGGCCGAGACGCCCGAGGGTCAGGCTTGCGGTC
TGGTGAAGAACTTGTCGCTGATGTGTTCGATCAGTGTTGGTACCTCTACGGATCCTATCGTAGACTA
CATGATTACCAGAAATATGGAGGTTCTCGAGGAGTACGAACCGCTGCGATACCCCGATGCTACTAA
GATCTTCCTCAACGGCTCTTGGATCGGCGTACATCAGAATCCCAAGGCTCTAGTGAGGGATGTTCA
GAATTTACGTCGTACCAATCAGATCCCGGCCGAGGTGTCGTTGGTCCGCGACATTCGCGATCGTGA
ATTCAAGATCTTTTCAGATGCCGGTCGTGTCATGCGCCCCATGTTCGTTGTACACCAAGAAGAC------
----

ACCGAGGATGGTATTAAGAAGGGAACGCTTGCTCTCACCAAGGACATGATCGCGAGGCTCGAGGC
AGATCTCGACGTAGATCCTGATAGTGAGGAGTACTTTGGCTGGGAAGGCCTGGTCAATGAAGGTGC
CATCGATTATCTTGATGCCGAGGAGGAAGAAACGTGCATGATTTGCATGACGCCCGAGGAACTCGA
GGGCTACCGCCAGCGTAAGGCCGGATTCGAAGTTTCTCAGGATAACGGG------
GATGAGATTAATAAGCGACTCAAGACTAAGATCAATCCGACGACACATATGTATACTCATTGCGAG
ATCCATCCCAGTATGCTTCTAGGTATTTGCGCAAGCATTATTCCGTTCCCGGATCATAATCAGGTAC
CCCTGAACGCGTCCCCCAACCCCCTGATCTCCTACCCCTCAAGCACAAACACCACAAACCCAAAAC
GTCTCAGTGCCCAGCATCGC---------AGACTAC--ATCG---
TATTTGAAGCTAAAAGCTAACCTTATTTCTTCGTCTCAATAGGTTCACCTCCAGACCGGCCAATGCG
TAAGAACCACCTATACCTACGACCATCGATCG-TCAACGCGAAAAAATATAGCGGGGCTCACGA--

ATTTTATAGGGTAACCAAATTGGTGCCGCTTTCTGGCAAACCATCTCTGGCGAGCACGGTCTCGAC
AGCAATGGCGTGTATGTGCTTGATTGGACAATTCTCATATGGGGATCGAAAATTAATAATTTCTGA
ATAGCTACAACGGAACCTCTGAGCTTCAGTTGGAGCGCATGAGCGTCTACTTCAACGAGGTACGTC
GGAATTAAGATAAAAGCTACATGCGCAAGAATTGTCACTAATTA--
TTGATATGCGCAGGCTTCCGGCAACAAGTATGTCCCCCGTGCCGTCCTCGTCGATCTCGAGCCCGG
CACCATGGATGCCGTCCGTGCTGGTCCTTTCGGTCAGCTCTTCCGACCCGACAACTTCGTCTTTGGT
CAATCCGGTGCTGGCAACAACTGGGCCAAGGGTCACTACACTGAGGGTGCTGAGCTTGTTGACAAC
GTTTTGGATGTCGTTCGTCGTGAGGCTGAGGGCTGCGACTGCCTTCAGGGTTTCCAGATTACCCACT
CGCTCGGTGGTGGTACCGGTGCCGGTATGGGTACCTTGTTGATCTCCAAGATCCGCGAGGAGTTCC
CCGACCGAATGATGGCCACCTTCTCCGTCGTTCCCTCCCCCAAGGTCTCCGACACCGTCGTCGAGCC
CTACAACGCCACCCTGTCCGTTCACCAGCTGGTCGAGAACTCGGACGAGACCTTCTGCATTGATAA
CGAGGCTCTGTACGATATCTGCATGCGTACCCTTAAGCTATCCAACCCCTCGTATGGTGACCTGAAC
CACCTGGTCTCCGCCGTTATGTCGGGTGTCACCACTTGCTTGCGATTCCCTGGTCAGCTAAACTCTG
ACCTGCGCAAGTTGGCCGTCAACATGGTGCCCTTCCCTCGTCTGCACTTCTTCATGGTTGGCTTCGC
TCCTCTGACCAGCCGTGGTGCTTACTCTTTCCGTGCTGTCACCGTTCCCGAGTTGACACAGCAGATG
TTCGACCCCAAGAACATGATGGCTGCCTCTGACTTCCGTAACGGTCGCTACCTGACGTGCTCTGCCA
TCTTGTAAGAGACCTGCTTTAAAGTTTTGATAAGTGCTTATTTGCTAACTCCTAATCTTTAGCCGTG
GCAAGATCTCCA

>Hypoxylon_chionostomum_STMA14060

-CATTACTGAGTTCTCAAAACTCC-
CACCCTTTGTGAATTATACTCCTGTTGCCTCGGCGCCGAGCGCAGCTACCCTGGA------------------------
------GCTACCCGGTAGCTACCCGGTAGTTGCGC-CCACGCTCCGCCGGTGGACCATTACACTCTTG-
TTATACACTGTATCTCTGAGACATT-
AACTAAATACGTTAAAACTTTCAACAACGGATCTCTTGGTTCTGGCATCGATGAAGAACGCAGCGA
AATGCGATAAGTAATGTGAATTGCAGAATTCAGTGAATCATCGAATCTTTGAACGCATATTGCGCC
CATTAGTATTCTAGTGGGCATGCCTATTCGAGCGTCATTTCAACCCTTACG-
CCCTGTTGCGTAGTGTTGGGAGTCTACCCTGTAAGGGGTAGTTCCCGAAAACCAGTGGCGGTGTTC-
GGTACACACATAGCGTAGTAATATTTCTCGCTTCTGTCGTGGCCTGAGCTACCCGCCGTAAAACCCC
CTATACTTCTAA---TGAAGCGGCAACAGCTCAAA-
TTTGAAATCTGGCC----CTC-----GCGGTCCGAATTGTAATTTGTAGAGGATGCTTTTGGTGCGGT-
GCCTTCCGAGTTCCCTGGAACGGGACGCCAGAGAGGGTGAGAGCCCCGTACGGTTGGA-
CACCTACCCTATATATAGCTCCTTCGACGAGTCGAGTAGTTTGGGAATGCTGCTCTAAATGGGAGG
TAAATTTCTTCTAAAGCTAAATACCGGCCAGAGACC-
GATAGCGCACAAGTAGAGTGATCGAAAGATGAAAAGCACTTTGAAAAGAGGGTTAAATAGCACGT
GAAATTGTTGAAAGGGAAGCG-TTTGCGACCAGACCTTTTCTAGGCGGATCATCCGGTGTTC--
TCACCGGTGCACTTCGCCTGG--TTTAGGCCAGCATCGGTTTT---
CGTAGGGGGACAAAGGCCTGGGGAACGTATCTC----CCTCGGGAGTGTT-
ATAGCCCCTAGCGTAATACCCCTAC-GGGGACCGAGGACCGCGCT-TCG---
GCAAGGATGCTGGCATAATGGTCGTCAACGACCCGTCTTGAAACACGGACCAAGGAGTCGAACAT
TTGTGCGAGTGTTTGGGTG--TTAAACCCTCACGCGTAATGAAAGTGAACGGAGGTGAGAGCCCCT--
---CG---GGGTGCATCATCGACCGATCCTGAAG-
TCTTCGGATGGATTTGAGTAAGAGCATAACTGTTCGGACCCGAAAGATGGTGAACTATGCGTGGAT
AGGGTGAAGCCAGAGGAAACTCTGGTGGAGGCTCGCAGCGGTTCTGACGTGCAAATCGATCGTCA
AATCTGCGCATGGGGGCGAAAGACTTATCGA-
ACCATCTAGTAGCTGGTTACCGCCGAAGTTTCCCTCAGGATAGCAGTGT--TG-
TTTTCAGTTTTATGAGGTAAAGCGAATGATTAGGGACTCGGGGGCGCTATATTGCCTTCATCCATTC
TCAAACTTTAAATATGTAAGAAGCCCTTGTTGCTTAATTGAACGTGGGCATTCGAATGTATCAACA
CTAGTGGGCCATTTTTGGTAAGCAGAACTGGCGATGCGGGATGAACCGAACGCGGGGTTAAGGTG
CCAGAGTGGACGCTCATCAGACACCACAAAAGGTGTTAGTACATCTAGACAGT-
GGACGGTGGCCATGGAAGTCGGAATCCGCTAAGGACTGTGTAACAACTCACCAACCGAATGTACT
AGCCCTGAAAATGGATGGCGCTCAAGCGTCT-
CACCCATACCTCGCCCTTAGGGTAGAAACGATGCCCTAAGGACGCTGCATCGACACGAACAAGCAT
TTCGAGATTGCCCTGGCTGCCAAGCCGGCCATCATCACGAACGGTATCAAGTACTCGCTCGCTACA
GGAAACTGGGGCGACCAGAAGAAGGCCATGAGCTCGACCGCCGGCGTGTCGCAGGTCTTGAACAG
ATACACGT------TCTCCTCGA---
CGCTTTCCCATCTGAGAAGAACCAACACGCCCATCGGGCGAGACGGAAAGCTGGCGAAGCCCCGT

CAGCTTCACAATACCCATTGGGGTCTGGTCTGCCCGGCCGAGACGCCCGAGGGTCAAGCCTGCGGT
CTGGTCAAGAACTTATCCTTGATGTGTTCGATCAGCGTCGGTACTTCCACAGATCCTATCGTCGACT
ATATGATTACGAGGAACATGGAGGTTTTGGAGGAGTACGAACCGCTGCGATACCCCGACGCCACC
AAGATTTTCCTCAACGGCTCGTGGATTGGCGTACACCAGAACCCCAAGGCCCTGGTCAGAGACGTC
CAGAACCTGCGCCGAACCAACCAGATCCCTGCCGAGGTGTCGCTGGTTCGCGACATACGCGACCGC
GAGTTCAAGATATTTTCTGATGCCGGCCGCGTCATGCGCCCGTTGTTCGTTGTGCAGCAAGAGGAT-
----

ACCGAGGCCGGCATCAAGAAGGGAACGCTGGCTCTCACCAAGGAGATGATCCAGAGACTCGAGGA
CGATGCTGAGATAGATCCCGACAGCGATGAGTACTTTGGCTGGCAAGGCTTGGTCAACGAAGGTGT
CATCGATTATCTCGATGCCGAGGAGGAAGAGACGGCCATGATCTGCATGACGCCCGAGGACCTCG
AGACCTACCGCCAGGCGAAGGCCGGCTTCGACGTGTCCCAGGATAACGGC------
GACGAGATTAACAAGCGCCTGAGGACCAAGATCAACCCGACCACGCACATGTACACCCACTGCGA
GATCCATCCCAGCATGCTTCTGGGTATCTGCGCGAGCATCATTCCGTTCCCGGATCATAACCAGGTA
CCCCTGAACGCGTCCCCCAACCCCCTGATCTCCTACCCCTCACGCACAAACACCACAAGCCCAAAA
CGTTCCGATTCCCAACACCGC---------AAAATAC--CACA---
TTCGAGAAATTAGAAGCTAACCATATCTCTTCATCGTAATAGGTTCACCTCCAGACCGGCCAATGC
GTAAGAACCACCTACACCCTCAACTACCGTCGA-
TCGACGCGAGAAAACATGGCGGGGCTCACACGGATTGTATAGGGTAACCAAATTGGTGCTGCTTTC
TGGCAGACCATCTCTGGCGAGCACGGCCTCGACAGCAATGGCGTGTATGTATTCCATTGGTCAATT
CTCCGGCGGGACTCCAAAGCTAATTGTTCGTCAACAGCTACAACGGTACTTCTGAGCTCCAGCTGG
AGCGCATGAGCGTCTACTTCAACGAGGTATGTCACGATCGAAAGCAAAAATACCCGCGCATGATCC
GCTACTAATTTCTTCGACGTGTGCAGGCTTCCGGCAACAAGTACGTTCCCCGTGCCGTCCTCGTCGA
TCTCGAGCCCGGTACCATGGACGCTGTCCGTGCCGGTCCTTTCGGTCAGCTCTTCCGACCCGACAAC
TTCGTCTTTGGTCAATCCGGTGCCGGCAACAACTGGGCCAAGGGTCACTACACGGAGGGTGCCGAG
CTTGTCGACCAGGTTCTGGATGTCGTCCGTCGCGAGGCTGAGGGCTGCGACTGCCTCCAGGGTTTC
CAGATCACCCACTCACTCGGTGGCGGTACCGGTGCCGGTATGGGTACCTTGTTGATCTCCAAGATC
CGCGAGGAGTTCCCCGACCGCATGATGGCCACTTTCTCCGTCGTTCCCTCCCCCAAGGTCTCCGACA
CGGTCGTCGAGCCCTACAACGCCACCCTCTCGGTCCACCAGCTGGTTGAGAACTCGGACGAGACGT
TCTGTATTGATAACGAGGCCCTATACGACATCTGCATGCGTACCCTCAAGCTATCCAACCCCTCGTA
CGGCGACCTGAACCACCTGGTCTCGGCCGTCATGTCGGGCGTCACCACTTGCTTGCGATTCCCCGG
CCAGCTGAACTCTGACCTGCGCAAGTTGGCTGTCAACATGGTGCCCTTCCCTCGTCTGCACTTCTTC
ATGGTCGGCTTCGCTCCCCTGACCAGCCGCGGTGCTTACTCTTTCCGTGCCGTCACCGTTCCCGAGT
TGACGCAGCAGATGTTCGACCCCAAGAACATGATGGCTGCCTCCGACTTCCGCAACGGTCGCTACC
TGACGTGCTCTGCCATCTTGTAGGATGCCCGCTCCTCATGGCCAATACGTGTTTCCTTGCTGACCCT
AA--TTCTAGCCGTGGCAAGATCTCCA

>Hypoxylon_addis_MUCL52797

-
CATTACTGAGTTCTAAAAACTCCCAACCCTTTGTGAACCATACTTCAGTTGCCTCGGCGCTGAGCGT
AGCTACCCGGGAGCTACCCTGGAGAAAT------------------
CCGCGACCTACCCTGTAGTTGCACCTAACGCTCCGCCGGTGGACCATTACACTCTGTTTTAACCACT
GTATCTCTGAAATACTTAACGAAATACGTTAAAACTTTCAACAACGGATCTCTTGGTTCTGGCATCG
ATGAAGAACGCAGCGAAATGCGATAAGTAATGTGAATTGCAGAATTCAGTGAATCATCGAATCTTT
GAACGCATATTGCGCCCATTAGTATTCTAGTGGGCATGCCTATTCGAGCGTCATTTCAACCCTTAAG
-
CCCTGTTGCTTAGTGTTAGGAGCCTGCTTTCAAGAGGGCAGCTCCCTAAAGATAGTGGCAGTGTTC-
GGTACACTCGTAGCGTAGTAATCTATCTCGCTTCTGTAGTGGCCCGAATTTCTCGCCGTAAAACCCC
CTAT-TTTCTCAAGGTTGACCTCGGATTAGGTAGGAATACCCGCTGAACTTAAGCAT-
TGAAGCGGCAACAGCTCAAA-TTTGAAATCTGGCC----CTM-----
GCGGTCCGAGTTGTAATTTGTAGAGGATGCTTTTGGTGCGGT-
GCCTTCTGAGTTCCCTGGAACGGGACGCCAGAGAGGGTGAGAGCCCCGTACGGTTGGM-
CACCTASCCTTTGTATAGCTCCTTCGACGAGTCGAGTAGTTTGGGAATGCTGCTCTAAATGGGAGGT
AAATTTCTTCTAAAGCTAAATACCGGCCAGAGACC-
GATAGCGCACAAGTAGAGTGATCGAAAGATGAAAAGCACTTTGAAAAGAGGGTTAAATAGCACGT
GAAATTGTTGAAAGGGAAGCG-TTTRCGACCAGACCTTTTCCAGGCGGATCATCCGGTGTTC--
TCACCGGTGCACTTCGTCGGG--TTTAGGCCAGCATCGGTTTT---
CGTAGGGGGATAAAAGCCCTGGGAACGTAGCTC----TTTCGGGAGTGTT-

ATAGCCCTAAGCATAATACCCTTAC-GGGGACCGAGGACCGCGCT-TCG---
GCAAGGATGCTGGCATAATGGTCGTCAACGACCCGTCTTGAAACACGGACCAAGGAGTCGAACAT
TTGTGCGAGTGTTTGGGTA--TCAAACCCTCACGCGTAATGAAAGTGAACGGAGGTGAGAGCCCCT--
---CG---GGGTGCATCATCGACCGATCCTGATG-
TCTTCGGATGGATTTGAGTAAGAGCATAACTGTTCGGACCCGAAAGATGGTGAACTATGCGTGGAT
AGGGTGAAGCCAGAGGAAACTCTGGTGGAGGCTCGCAGCGGTTCTGACGTGCAAATCGATCGTCA
AATCTGCGCATGGGGGCGAAAGACTTATCGA-
ACCATCTAGTAGCTGGTTACCGCCGAAGTTTCCCTCAGGATAGCAGTGT--TG-
TTTTCAGTTTTATGAGGTAAAGCGAATGATTAGGGACTCGGGGGCGCTATATTGCCTTCATCCATTC
TCAAACTTTAAATATGTAAGAAGCCCTTGTTGCTTAATTGAACGTGGGCATTCGAATGTATCAACA
CTAGTGGGCCATTTTTGGTAAGCAGAACTGGCGATGCGGGATGAACCGAACGCGGGGTTAAGGTG
CCAGAGTGGACGCTCATCAGACACCACAAAAGGTGTTAGTACATCTAGACAGTAGGACGGTGGCC
ATGGAAGTCGGAATCCGCTAAGGACTGTGTAACAACTCACCAACCGAATGTACTAGCCCTGAAAAT
GGATGGCGCTCAAGCGTCT-
CACCCATACCTCGCCCTTAGGGTAGAAACGATGCCCTAAGGGCGATGCATCGAAGCGAACAAGCA
TTTTGAGATTGCTCTGGCCGCTAAACCAGCTATTATCTCAAACGGTCTCAAGTACTCACTCGCTACA
GGAAACTGGGGTGATCAGAAGAAGGCCATGAGCTCGACAGCTGGTGTGTCGCAAGTCTTGAACCG
ATACACGT------TCTCGTCGA---
CACTTTCTCACTTGAGACGAACCAACACGCCTATCGGACGTGATGGAAAGCTTGCCAAACCTCGGC
AGCTTCACAATACCCATTGGGGCTTGGTTTGTCCGGCAGAAACACCCGAGGGCCAGGCTTGCGGCC
TGGTCAAGAACTTGTCATTGATGTGTTCTATCAGTGTCGGTACCTCCACGGATCCTATCGTGGATTA
CATGATTACGAGAAACATGGAAGTTCTCGAGGAGTACGAACCCTTGCGATACCCCGATGCTACCAA
GATCTTTCTCAACGGTTCCTGGATCGGCGTGCACCAAAACCCTAAGGCTCTAGTCAGAGATGTCCA
GAACTTACGCCGAACGAATCAAATCCCAGCCGAGGTGTCGTTAGTCCGAGATATACGTGATCGTGA
ATTCAAGATCTTTTCGGATGCCGGTCGAGTCATGCGTCCCTTGTTCGTCGTACATCAAGAGGAT------
----
ACTGATACTGGCGCCAAGAAAGGGACATTGGCTCTTACCAAGGAGATAATCCAGAGGCTTGAAGC
GGATGTAGATCTAGATCCCGACAGTGATGCGTACTTTGGCTGGCAGGGGTTGGTAAACGAAGGTGT
CATCGACTATCTTGATGCCGAAGAAGAAGAGACAACCATGATCTGCATGACTCCTGAGGACCTTGA
GACTTACCGCCAGGCGAAGGCCGGTATGGAGGTAAACCAGGATAACGGA------
GACGAGGTTAATAAGCGTCTCCGAACCAAGGTTAATCCCACGACACACATGTACACTCACTGCGAG
ATTCATCCTAGCATGCTTCTAGGTATTTGCGCGAGCATTATTCCGTTCCCGGATCATAATCAGGTA---
CTGAACGCGTCCCCCAATTCTCCTGACTCCTACCCCTCACACACATATACCACAAGCAGGAAAAA---
-----------------ATGTCATTGAGTTTTATCGC-
ATACAGAGGAAATGAAAAGCTAATCGTATCTCTTCGTTTAAATAGGTTCACCTTCAGACCGGCCAA
TGTGTAAGAATCAACCT-----AAAACCTACAACGAA-----
ACGAAGGAAGATAGCGGGGCTCACACGATTTTAATAGGGTAACCAAATTGGTGCTGCTTTCTGGCA
GACCATTTCTGGTGAGCACGGCCTTGACAGCAATGGAGTGTATGT----
AATTGGTCAACCCATTGAATAGGTACGGGAGTTAAT---
TGTTTGTCAGGTATAACGGAACCTCCGAGCTTCAGCTGGAGCGCATGAGCGTCTACTTCAACGAGG
TATGTTACAATGAAGAGCGAAAAAAGATGCGCAAGATCAATCACTAATTACTTGAATACGCGCAG
GCTTCCGGTAACAAGTATGTTCCTCGTGCCGTTCTTGTCGATCTCGAACCCGGTACCATGGATGCCG
TTCGTGCTGGTCCCTTTGGTCAGCTCTTCCGACCCGACAACTTCGTTTTCGGTCAATCCGGTGCCGG
AAACAACTGGGCCAAGGGTCACTACACCGAGGGTGCTGAGTTGGTCGACCAGGTCCTTGACGTCGT
TCGTCGTGAGGCTGAGGGTTGCGATTGCCTCCAGGGTTTCCAGATTACCCACTCGCTCGGTGGTGGT
ACCGGTGCCGGTATGGGTACCTTGTTGATCTCCAAGATTCGCGAGGAGTTCCCCGACCGTATGATG
GCTACTTTCTCCGTCGTTCCTTCCCCCAAGGTTTCCGATACCGTTGTCGAGCCTTACAACGCTACCCT
CTCGGTTCACCAGCTGGTCGAAAACTCGGACCAGACCTTCTGCATTGATAACGAGGCTTTGTACGA
CATCTGCATGCGTACCCTTAAGCTATCCAACCCTTCGTACGGTGACCTGAACCACCTGGTCTCGGCC
GTCATGTCGGGTGTTACCACTTGCTTGCGATTCCCTGGTCAGCTAAACTCTGATCTCCGCAAGTTGG
CTGTTAACATGGTGCCTTTCCCTCGTCTGCACTTCTTCATGGTCGGCTTTGCTCCTCTGACTAGCCGT
GGTGCTTACTCTTTCCGTGCTGTTACCGTTCCCGAGTTGACTCAGCAAATGTTCGACCCCAAGAACA
TGATGGCTGCTTCCGACTTCCGTAACGGTCGTTACCTAACGTGCTCTGCCATCTTGTAAGTTTATTG
TATTTTCTA---TAGTAATTGTCCCTCGCTAACTTT--TACTCTAGCCGTGGCAAGATCTCAA

>Hypoxylon_delonicis_MFLU161031

-
CCTTTCTGAGTTTTCAAAACTTCCCACCCTTTGTGAATCTTACTTCAGTTGCCTTGGCGGTGAGCGC
GGCCGCTGGGAACGATCCCCGGACATGT------------------
TCGGGGACGGGCCCGCAGGTGCCACGAACGGTTCGCCGGCGGGCCCCTTAACTTTGTTTTAACCAC
GGTATCTCTGAATTACTTAACGAAATACGTTAAAACTTTCAACAACGGATCTCTTGGTTCTGGCATC
GATGAAGAACGCAGCGAAATGCGATAAGTAATGTGAATTGCAGAATTCAGTGAATCATCGAATCT
TTGAACGCATATTGCGCCCATTAGTATTCTAGTGGGCATGCCTATTCGAGCGTCATTTCAACCCTTA
AG-
CCCTGTTGCTTAGTGTTAGGAGCCTGCTTTCAAGAGGGCAGCTCCCTAAAGATAGTGGCAGTGTTC-
GGTACACTCGTAGCGTAGTAATTTATCTCGCTTCTTCAGTGGCCCGAATCTCTCGCCGTA--------------
---TGAAGCGGCAACAGCTCAAA-TTTGAAATCTGGCC----CTC-----
GTGGTCCGAGTTGTAATTTGTAGAGGATGCTTTTGGTGCGGT-
GCCTTCCGAGTTCCCTGGAACGGGACGCCAGAGAGGGTGAGAGCCCCGTACGGTTGGA-
CACCTACCCTATATATAGCTCCTTCGACGAGTCGAGTAGTTTGGGAATGCTGCTCTAAATGGGAGG
TAAATTTCTTCTAAAGCTAAATACCGGCCAGAGACC-
GATAGCGCACAAGTAGAGTGATCGAAAGATGAAAAGCACTTTGAAAAGAGGGTTAAATAGCACGT
GAAATTGTTGAAAGGGAAGCG-TTTGCGACCAGACCTTTTCCTGGCGGATCATCCGGTGTTC--
TCACCGGTGCACTTCGCTTGG--TTTAGGCCAGCATCGGTTTT---
TGTAGGGGGATAAAAGCCTGGGGAACGTAGCTC----CTTCGGGAGTGTT-
ATAGCCCCTAGCATAATACCCTTAC-GGGGACCGAGGACCGCGCT-TCG---
GCAAGGATGCTGGCGTAATGGTCGTCAACGACCCGTCTTGAAACACGGACCAAGGAGTCGAACAT
TTGTGCGAGTGTTTGGGTA--TTAAACCCTCACGCGTAATGAAAGTGAACGGAGGTGAGAGCCCTT--
---CG---GGGTGCATCATCGACCGATCCTGATG-
TCTTCGGATGGATTTGAGTAAGAGCATAACTGTTCGGACCCGAAAGATGGTGAACTATGCGTGGAT
AGGGTGAAGCCAGAGGAAACTCTGGTGGAGGCTCGCAGCGGTTCTGACGTGCAAATCGATCGTCA
AATCTGCGCATGGGGGCGAAAG--

CCCCTGAACGCGTCCCCCAATTCCCCTGATTCCTACCCCTCACACACATATACCACAAGCAGAAAA
AA---------------------GCTATTGAGTTTTATCGC-
ATACAGAGGAAATGAAAAGCTAATCGTATCTCTTCGTTTAAATAGGTTCACCTTCAGACCGGCCAA
TGTGTAAGAATCAACCT------TAACCTACAACGAA-----
ACGAAGGAAGATAGCGGGGCTCACACGATTTTAATAGGGTAACCAAATTGGTGCTGCTTTCTGGCA
GACCATTTCTGGTGAGCACGGTCTTGACAGCAATGGAGTGTATGT----
AATTGGTCAATTCATTGAATAGGTACGGAAGTTAAT---
TGTTTGTCAGGTATAACGGAACCTCCGAGCTTCAGCTGGAGCGCATGAGCGTCTACTTCAACGAGG
TACGTTACAATGAAGGGCAAAAAAAAATACCCAAAATCAATCCCTAATTACTTGAAAACCCCCAG
GCTTCGGGAAAAAAGTATGTTCCTCGGGCCGTTCTTGTCAATCTCAAACCCGGTACCATGGATGCC
GTTCGGGCTGGTCCCTTTGGTCAGCTCTTCCAACCCGACA--

>Hypoxylon_pilgerianum

-
CATTACTGAGTTCTACAAACTCCCAACCCTTTGTGAATCATATCACTGTTGCCTCGGCGCCGAGCGC
AGCTACCCGGGAGCTACCCTGTAGAAGC-------
GAGAGCATCTACCCTGTAGCTACCCTGTAGTTGCACTCAACGCTCCGCCGGCGGACCATTCAACTC
TGTTTT--
ACAGTGTATCTCTGAGTACTTAAACTAAATAAGTTAAAACTTTCAACAACGGATCTCTTGGTTCTGG
CATCGATGAAGAACGCAGCGAAATGCGATACGTAATGTGAATTGCAGAATTCAGTGAATCATCGA
ATCTTTGAACGCATATTGCGCCCATTAGTATTCTAGTGGGCATGCCTATTCGAGCGTCATTTCAACC
CTTAAG-
CCCTGTAGCTTAGTGTTGGGACTCTACTCTTTAGTGAGCAGTTCCCTAAAACCAGTGGCGGTGCTA-
GGTACACTCATAGCGTAGTAATCTTTCTCGCTTCTGCGGTGGACGTAGCTACCTGCCGTAAAACCCC
CTAT-
TTTCTAATGGTTGACCTCGGATTAGGTAGGAATACCCGCTGAACTTAAGCATATGAAGCGGCAACA
GCTCAAA-TTTGAAATCTGGCC----CTA-----
GCGGTCCGAGTTGTAATTTGTAGAGGATGCTTTTGGTGCGGT-
GCCTTCCGAGTTCCCTGGAACGGGACGCCAGAGAGGGTGAGAGCCCCGTACGGTTGGA-
CACCTACCCTATATATAGCTCCTTCGACGAGTCGAGTAGTTTGGGAATGCTGCTCTAAATGGGAGG
TAAATTTCTTCTAAAGCTAAATACCGGCCAGAGACC-
GATAGCGCACAAGTAGAGTGATCGAAAGATGAAAAGCACTTTGAAAAGAGGGTTAAATAGCACGT
GAAATTGTTGAAAGGGAAGCG-TTTGCGACCAGACTTTTTCAGGGAGGATCATCCGGTGTTC--
TCACCGGTGCACTTCACCCTG--TTTAGGCCAGCATCGGTTTT---
TGCAGGGGGATAAAAACTTGGGGAATGTGGCTC----CTTCGGGAGTGTT-
ATAGCCCCTTGTATAATACCCCTGC-GGAG--

ACGCTGTATCGACACGAACAAGCATTTCGAAATTGCCCTGGCTGCTAAGCCGGCTATCATCACGAA
CGGTCTCAAGTACTCACTCGCCACAGGAAACTGGGGTGATCAGAAGAAGGCCATGAGCTCGACGG
CTGGTGTGTCCCAGGTCTTGAACCGATACACCT------TCTCTTCCA---
CCCTTTCTCATTTAAGAAGAACCAACACACCTATTGGGCGTGACGGCAAGTTGGCGAAGCCCCGAC
AGCTTCACAATACACATTGGGGTCTAGTCTGTCCGGCCGAAACGCCCGAGGGCCAGGCCTGCGGCT
TGGTGAAGAACTTATCATTGATGTGTTCCATCAGCGTCGGTACCTCGACGGATCCCATCGTGGATTA
CATGATTACCAGGAATATGGAGGTTCTCGAAGAGTACGAGCCGCTACGGTATCCCGATGCCACCAA
GATCTTCCTCAACGGTTCCTGGATAGGTGTTCATCAGAATCCCAAGGCGCTGGTGAGAGATGTTCA
GAATCTTCGGCGGACGAATCAGATCCCGGCCGAGGTGTCGTTAGTCCGCGACATACGCGATCGCGA
ATTCAAAATCTTTTCGGATGCCGGTCGCGTCATGCGCCCCCTGTTTGTTGTACATCAAGAGGAC------
----
ACCGAGGCCGGCACTAAGAAGGGAACGTTAGCTCTTACTAAAGACATGATCCAGAGGCTCGAGTC
GGATGTCGAAGTAGATCCTAATAGCGAGGAGTACTTTGGCTGGGAAGGCTTGGTCAACGAAGGTG
CTATCGATTATCTAGACGCCGAGGAGGAAGAAACGGCCATGATATGCATGACGCCCGAGGACTTG
GAAACCTATCGGCAGACCAAGGCCGGGTTCGAAGTATCCCAGGATAACGGG------
GACGAGATTAATAAGCGACTGAGGACCAAGGTGAACCCGACCACGCACATGTACACTCACTGCGA
GATCCATCCTAGCATGCTTCTAGGTAT--
CCCAACCCCCTGAT-TTCCCCCCCTCACGCACAAACACCACAACCACAGACC-
TCTCAATTCCCTACATTGC---------AGATTACCATATA---
TTTTGGAAGACAAAAGCTAACCATATCTTTTCCATATAATAGGTTCACCTCCAGACTGGCCAATGTG
TAAGTACTACCTACACCTACAGCTGCCGACCA-
GCGACGCGAAAGAATATGGCAGGGCTCACATATATTTTATAGGGTAACCAAATTGGTGCTGCTTTC
TGGCAAACCATCTCTGGCGAGCACGGTCTCGACAGCAATGGCGTGTATGTACATCATTGGTCAATT
GGCAGGGA--
GGATCAAAACTAACAGTTGGGTTATAGCTACAACGGAACCTCTGAGCTCCAGCTCGAGCGCATGAG
CGTCTACTTCAACGAGGTATGCCAAAAAAAGAGGCAAGAATACATGCGCAGGACCCGTAACTAAT
TACCTTGACGTTTACAGGCTTCCGGCAACAAGTATGTTCCCCGTGCCGTCCTCGTCGATCTCGAGCC
CGGTACCATGGATGCCGTCCGTGCTGGTCCTTTCGGTCAACTCTTCCGACCCGACAACTTTGTTTTC

GGTCAATCCGGTGCCGGCAACAACTGGGCCAAGGGTCATTACACTGAGGGTGCCGAGCTTGTCGAC
CAGGTTTTGGATGTCGTTCGTCGTGAGGCTGAGGGCTGCGATTGCCTCCAGGGTTTCCAGATTACCC
ACTCGCTCGGTGGTGGTACCGGTGCCGGTATGGGTACCTTGTTGATCTCCAAGATCCGCGAGGAGT
TTCCCGACCGCATGATGGCCACCTTCTCCGTTGTTCCCTCCCCCAAGGTCTCCGATACCGTTGTCGA
GCCTTACAACGCCACTCTCTCCGTCCACCAGCTGGTCGAGAACTCAGACGAGACCTTCTGTATCGA
TAACGAGGCTCTGTACGACATCTGCATGCGTACCCTGAAGCTATCCAACCCTTCGTACGGTGACCT
GAACCACTTGGTCTCCGCCGTCATGTCGGGTGTCACCACTTGCTTGCGATTCCCTGGTCAGCTGAAC
TCTGACCTCCGCAAGTTGGCTGTCAACATGGTGCCCTTCCCTCGTCTGCACTTCTTCATGGTCGGCT
TCGCTCCCCTGACCAGCCGTGGTGCTTACTCCTTCCGTGCTGTCACCGTCCCCGAGTTGACCCAGCA
GATGTTCGACCCCAAGAACATGATGGCTGCCTCTGACTTCCGCAACGGTCGCTACCTAACATGCTC
TGCCATCTTGTAAGCTTCCTCCTTCCCATTGTCGATTAATATTTGTTTCTGACCTA--
AATTCTAGCCGTGGCAAGATCTCCA

>Hypoxylon_wuzhishanense_FCATAS2708

-----------TTCTTCTCAACTCCCACCCTTTGTGA-
TCTTACCCACTGTTGCCTCGGCGCCGAGCGCAGCTACCCGGGAGCTACCCTGGAGACACCTACCCTG
TAGATGGCTACCCTGGAGCTACCCTGTAGTTGCACTCAACGCTCCGCCGGCGGACCATTAAACTCT
GTTTT--
ACATTGTATCTCTGAGTATATAACCAAAATACGTTAAAACTTTCAACAACGGATCTCTTGGTTCTGG
CATCGATGAAGAACGCAGCGAAATGCGATACGTAATGTGAATTGCAGAATTCAGTGAATCATCGA
ATCTTTGAACGCATATTGCGCCCATTAGTATTCTAGTGGGCATGCCTATTCGAGCGTCATTTCAACC
CTTAAGCCCCTGTTGCTTAGTGTTGGGAACCTACTCCTCAGGGTGTAGCTCCCTAAAACCAGTGGCG
GTGCTA-
GGTACACTCGTAGCGTAGTAAATTTTCTCGCTCCTGTAGTGTTCGTAGTTACCGGCCGTAAAACCCT
T-
ATATTTCTAGTGGTTGACCTCGGATTAGGTAGGAATACCCGCTGAACTTAAGCATATGAAGCGGCA
ACAGCTCAAA-TTTGAAATCTGGCC----CTA-----
GCGGTCCGAGTTGTAATTTGTAGAGGATGCTTTTGGTGCGGT-
GCCTTCCGAGTTCCCTGGAACGGGACGCCAGAGAGGGTGAGAGCCCCGTACGGTTGGA-
CACCTACCCTATATATAGCTCCTTCGACGAGTCGAGTAGTTTGGGAATGCTGCTCTAAATGGGAGG
TAAATTTCTTCTAAAGCTAAATACCGGCCAGAGACC-
GATAGCGCACAAGTAGAGTGATCGAAAGATGAAAAGCACTTTGAAAAGAGGGTTAAATAGCACGT
GAAATTGTTGAAAGGGAAGCG-TTTGCGACCAGACCTTTTCCGGGCGGATCATCCGGTGTTC--
TCACCGGTGCACTTCGTCCGG--TTTAGGCCAGCATCGGTTTT---
TGTAGGGGGATAAAAGCCTGGGGAACGTGGCTC----CTTCGGGAGTGTT-
ATAGCCCCTAGCATAATACCCCTAC-AGGGACCGAGGACCGCGCT-TCG---
GCAAGGATGCTGGCGTAATGGTCGTCAACGACCCGTCTTGAAACACGGACCAAGGAGTCGAACAT
TTGTGCGAGTGTTTGGGTA--TTAAACCCTCACGCGTAATGAAAGTGAACGGAGGTGAGAGCCCTT--
---CG---GGGTGCATCATCGACCGATCCTGATG-
TCTTCGGATGGATTTGAGTAAGAGCATAACTGTTCGGACCCGAAAGATGGTGAACTATGCGTGGAT
AGGGTGAAGCCAGAGGAAACTCTGGTGGAGGCTCGCAGCGGTTCTGACGTGCAAATCGATCGTCA
AATCTGCGCATGGGGGCGAAAG--

GCGCTGCATCGACACCAACAAGCACTTCGAGATGGCCCTGGCTGCTAAGCCGGCTATCATTACGAA
CGGTCTCAAGTACTCGCTCGCCACAGGAAACTGGGGCGATCAGAAGAAGGCCATGAGCTCGACCG
CCGGTGTGTCGCAGGTGTTGAACCGATACACCT------TCTCTTCGA---
CACTTTCTCATTTGAGACGAACCAACACGCCTATTGGGCGTGACGGAAAACTGGCGAAGCCTCGAC
AACTTCACAATACTCATTGGGGTCTGGTCTGCCCGGCCGAAACGCCCGAGGGCCAGGCCTGTGGCT
TGGTGAAGAACTTATCGCTGATGTGTTCCATCAGCGTCGGCACCTCTACAGATCCCATCGTGGATTA
TATGATTACTAGAAATATGGAAGTCCTCGAAGAGTACGAGCCGCTGCGGTATCCTGACGCCACCAA
GATCTTCCTGAACGGTTCTTGGATCGGTGTACACCAGAACCCCAAGGCTCTAGTGAGAGATGTTCA
GAATCTTCGGCGAACCAACCAGATCCCGGCCGAGGTGTCGCTAGTCCGCGACATACGCGATCGTGA
ATTCAAGATCTTTTCGGATGCCGGTCGCGTCATGCGACCCCTGTTTGTGGTACAGCAAGAGGAC-----
----

ACCGAGGCTGGCGCTAAGAAGGGAACGCTAGCCCTTACTAAAGAGATGATCCAGAGGCTCGAGGC

GGATGTCGAGGTAGATCCTAATAGCGAGGAGTACTTTGGCTGGGAAGGCTTGGTCAATGAAGGTG
CTATCGATTATCTCGACGCCGAGGAGGAAGAGACGGCGATGATCTGCATGACGCCCGAGGATCTTG
AGACCTATCGCCAGACCAAGGCCGGATACGAAGTGTCTCAGGATAACGGA------
GACGAGATTAACAAGCGACTGAGGACCAAGGTTAATCCGACCACACACATGTATACTCACTGCGA
GATCCATCCGAGTATGCTTCTGGGTATCTGCGCGAGCATTATTCCATTCCCGGATCACAATCAGGTA
--C--AGATTAC--TGCA---
TTCCAGACGACGAAAGCTAACCATATTTCTTA--
AATAATAGGTTCACCTCCAGACTGGCCAATGCGTAAGATTTACCTACACCTGCAGCGGACGATCA-
TCGACGCGAAAGAATATAGTCGGGCTCACATA----
TTATAGGGTAACCAAATTGGTGCCGCTTTCTGGCAGACCATCTCTGGCGAGCACGGTCTGGACAGC
AATGGCGTGTATGTTGTTCATCGGGCAATTGTTAATG---GATTGAAGACTAAT----
TATTCATAGCTACAACGGAACCTCCGAGCTCCAGCTGGAGCGCATGAGCGTCTACTTCAACGAGGT
ACGTCTAATCAAAAACAAAGAATACATGCGCAAGACTCATTACTAATTACCTGGACGTGCGCAGGC
TTCCGGCAACAAATACGTCCCCCGTGCCGTCCTTGTCGATCTCGAGCCCGGTACCATGGATGCCGTC
CGTGCTGGTCCTTTCGGTCAACTCTTCCGCCCAGACAACTTCGTCTTTGGTCAATCCGGTGCCGGCA
ACAACTGGGCCAAGGGTCACTACACTGAGGGTGCCGAACTTGTCGACCAGGTTCTGGATGTCGTCC
GTCGTGAGGCTGAAGGCTGCGACTGCCTGCAGGGTTTCCAGATCACCCACTCGCTCGGTGGTGGTA
CCGGTGCCGGTATGGGTACCTTGTTGATCTCCAAGATCCGCGAGGAGTTCCCCGACCGCATGATGG
CTACTTTCTCCGTCGTTCCCTCCCCCAAGGTCTCCGACACCGTCGTCGAGCCCTACAACGCCACCCT
CTCCGTCCACCAGCTGGTCGAGAACTCGGACGAGACCTTCTGCATTGATAACGAGGCTCTGTACGA
CATCTGCATGCGCACCCTGAAGCTATCCAACCCTTCGTACGGTGACCTGAACCACCTCGTCTCCGCC
GTCATGTCGGGTGTCACCACTTGTTTGCGATTCCCCGGTCAGCTGAACTCTGACCTCCGCAAGTTGG
CCGTCAACATGGTGCCGTTCCCTCGTCTGCACTTCTTCATGGTCGGCTTCGCTCCCCTGACCAGCCG
TGGTGCTTACTCTTTCCGTGCCGTCACCGTCCCCGAGTTGACCCAGCAGATGTTCGACCCCAAGAAC
ATGATGGCTGCTTCTGACTTCCGCAACGGTCGCTACCTGACGTGCTCTGCCATCTTGTAAGTTCTCC
CATT-CAGTTGTCGATCAATTTTTGTTTTCTAACCCG--AAAACTAGCCGTGGCAAGATCTCCA

>Hypoxylon_gibriacense_MUCL52698

-
CATTACCGAGTTCTCAAAACTCCCAACCCTTTGTGAACCTTATTCATGTTGCCTCGGCGCCGAGCGC
ATCTACCCTGCAGCTACTCT---------------------------
GGAGCTACCCTGTAGTTGCGCCAAACGCTCCGCCGGTGGACCATTCAACTCTGTCTT--
ATGGTGTATCTCTGAACTATATAACAAAATTCGTTAAAACTTTCAACAACGGATCTCTTGGTTCTGG
CATCGATGAAGAACGCAGCGAAATGCGATAAGTAATGTGAATTGCAGAATTCAGTGAATCATCGA
ATCTTTGAACGCATATTGCGCCCATTAGTATTCTAGTGGGCATGCCTATTCGAGCGTCATTTCAACC
CTTAAG-
CCCTGTAGCTTAGCGTTGGGACCCTACTCCTCGGGTAGTAGCTCCCTAAAACCAGTGGCGGTGTTC-
GGTACACTCATAGCGTAGTATATATTTTCGCTTCTGCAGTGGACCGGACGGCTCGCCGTAAAACCC
CTTATATTTCTAGTGGTTGACCTCGGATTAGGTAGGAATACCCGCTGAACTTAAGC---
TGAAGCGGCAACAGCTCAAA-TTTGAAATCTGGCC----CTC-----
GTGGTCCGAATTGTAATTTGCAGAGGATGCTTTTGGTGCGGT-
GCCTTCCGAGTTCCCTGGAACGGGACGCCAGAGAGGGTGAGAGCCCCGTACGGTTGGA-
CACCTACCCTATATATAGCTCCTTCGACGAGTCGAGTAGTTTGGGAATGCTGCTCTAAATGGGAGG
TAAATTTCTTCTAAAGCTAAATACCGGCCAGAGACC-
GATAGCGCACAAGTAGAGTGATCGAAAGATGAAAAGCACTTTGAAAAGAGGGTTAAATAGCACGT
GAAATTGTTGAAAGGGAAGCG-TTTGCGACCAGACTTTTTCCAGGCGGATCATCCGGTGTTC--
TCACCGGTGCACTTCGCCTGG--TTTAGGCCAGCATCGGTTTT---
CTTAGGGGGATAAAGGCCTGGGGCACGTAGCTC----CCTCGGGAGTGTT-
ATAGCCCCTAGCGTAATACCTTTCA-GGGGACCGAGGACCGCGCT-TCG---
GCAAGGATGCTGGCGTAATGGTTGTCAACGACCCGTCTTGAAACACGGACCAAGGAGTCGAACAT
TTGTGCGAGTGTTTGGGTA--TTAAACCCTCACGCGTAATGAAAGTGAACGGAGGTGAGAGCCCTT--
---CG---GGGTGCATCATCGACCGATCCTGATG-
TCTTCGGATGGATTTGAGTAAGAGCATAACTGTTCGGACCCGAAAGATGGTGAACTATGCGTGGAT
AGGGTGAAGCCAGAGGAAACTCTGGTGGAGGCTCGCAGCGGTTCTGACGTGCAAATCGATCGTCA
AATCTGCGCATGGGGGCGAAAGACTTATCGA-
ACCATCTAGTAGCTGGTTACCGCCGAAGTTTCCCTCAGGATAGCAGTGT--TG-
TTTTCAGTTTTATGAGGTAAAGCGAATGATTAGGGACTCGGGGGCGCTTTATTGCCTTCATCCATTC

TCAAACTTTAAATATGTAAGAAGCCCTTGTTGCTTAATTGAACGTGGGCATTCGAATGTATCAACA
CTAGTGGGCCATTTTTGGTAAGCAGAACTGGCGATGCGGGATGAACCGAACGCGAGGTTAAGGTG
CCAGAGTGGACGCTCATCAGACACCACAAAAGGTGTTAGTACATCTAGACAGT-
GGACGGTGGCCATGGAAGTCGGAATCCGCTAAGGACTGTGTAACAACTCACCAACCGAATGTACT
AGCCCTGAAAATGGATGGCGCTCAAGCGTCT-
CACCCATACCTCGCCCTTAAGGTAGAAACGATGCCCTAAGG---

CCCCTGAACGCGTCCCCCAACCCCCTGATCTCCTACCCCTCACGCACAAACGCCACAAACACAAAA
CGTCTCAATCCCCTACATCGC---------AGGCCAC--AACA---
TTCTAGAAACTGAAAGCTAACCTTATCTCTTCTTCTCAATAGGTTCACCTCCAGACCGGCCAATGCG
TAAGAACTACCTTTACC----ACCACCGACCA-
TCAGCGCGAAAAAATATGGCGGGGCTCACACGAATATTATAGGGTAACCAAATTGGTGCTGCTTTC
TGGCAAACCATCTCTGGCGAGCACGGTCTCGACAGCAATGGCGTGTATGTATCTCATTGGTCAATT
C-
TCAACGGGAATTGGAAACTAATAGTTGGTCAACAGCTACAACGGAACCTCGGAGCTCCAGCTCGA
GCGCATGAGCGTCTACTTCAACGAGGTATGTCGAA-
TCGAGAGCAAGAATACATGCGCAAGACTCGTTGCTAATAACTTCCACGTGCGCAGGCTTCCGGCAA
CAAGTATGTTCCCCGTGCCGTCCTCGTCGATCTCGAGCCCGGTACCATGGACGCTGTCCGCGCTGGT
CCTTTCGGTCAGCTCTTCCGACCTGACAACTTCGTCTTCGGTCAATCCGGTGCCGGCAACAACTGGG
CCAAGGGTCACTACACCGAGGGTGCTGAGCTTGTTGACCAAGTTTTGGATGTCGTTCGTCGTGAGG
CTGAGGGCTGCGACTGCCTTCAGGGTTTCCAGATTACCCACTCGCTCGGTGGTGGTACCGGTGCCG
GTATGGGTACCTTGTTGATCTCCAAGATCCGCGAGGAGTTCCCCGACCGCATGATGGCCACCTTCTC
TGTCGTTCCCTCCCCCAAGGTCTCTGATACCGTTGTCGAGCCCTACAACGCCACCCTCTCCGTCCAC
CAGCTGGTCGAGAACTCGGACGAGACCTTCTGCATTGATAACGAGGCTCTCTACGATATCTGCATG
CGTACCCTAAAGCTATCCAACCCCTCGTACGGCGACCTGAACCACCTGGTCTCCGCCGTCATGTCG
GGTGTTACCACTTGCTTGCGATTCCCCGGTCAGCTAAACTCTGACCTCCGCAAGTTGGCTGTCAACA
TGGTGCCCTTCCCTCGTCTGCACTTCTTCATGGTTGGCTTCGCTCCCCTGACCAGCCGTGGTGCTTAC
TCTTTCCGCGCWGTCACCGTTCCCGAGTTGACGCAGCAGATGTTCGACCCCAAGAACATGATGGCY
GCCTCCGATTTCCGCAACGGTCGCTACCTGACGTGCTCTGCCATCTTGTAAGATACATACCCCGAGT
TATCGAGAAGTGCTTGTTTGCYAACCTMTAATGTCTAGCCGTGGCAAGATCTCCA

>Hypoxylon_inaequale_KUNCC2210798

-
CATTACTGAGTTCTCAAAACTCCCAACCCTTTGTGAATTATACCGTCGTTGCCTCGGCGAGGGTGGC
ATCTACCCGGGAGCTACCCTG----TACTTACCCTGTAAGAGACT--
TGGGGAGCTACCCTGGAGTTGCCC-
GTACGCCCCGCTGGTGGACCACCAAACTCTGTTTAAACTGGTGTATCTCTGAATTCTTTAACTAAAT
ACGTTAAAACTTTCAACAACGGATCTCTTGGTTCTGGCATCGATGAAGAACGCAGCGAAATGCGAT
AAGTAATGTGAATTGCAGAATTCAGTGAATCATCGAATCTTTGAACGCATATTGCGCCCACTAGTA
TTCTGGTGGGCATGCCTATTCGAGCGTCATTTCAACCCTTAAG-
CCCTGTTGCTTAGCGTTGGGAACCTACCCTGTAGGGCGTAGCTCCCTAAAAGTAGTGGCAGTGTTA-
GGTACACTCGTAGCGTAGTAAATTTTCTCGCTCCTGTAGTGCCCCTGACGACTCGCCGTAAAACCCC
CTATACTTCAAGTGGTTGACCTCGGATTAGGTAGGAATACCCGCTGAACTTAAG----
TGAAGCGGCAACAGCTCAAA-TTTGAAATCTGGCC----CTC-----
GTGGTCCGAATTGTAATTTGTAGAGGATGCTTTTGGTGCGGT-
GCCTTCCGAGTTCCCTGGAACGGGACGCCAGAGAGGGTGAGAGCCCCGTACGGTTGGA-
CACCTACCCTATATATAGCTCCTTCGACGAGTCGGGTAGTTTGGGAATGCTGCTCTAAATGGGAGG
TAAATTTCTTCTAAAGCTAAATACCGGCCAGAGACC-
GATAGCGCACAAGTAGAGTGATCGAAAGATGAAAAGCACTTTGAAAAGAGGGTTAAATAGCACGT
GAAATTGTTGAAAGGGAAGCG-TTTACGACCAGACCTTTTCCAGGCGGATCATCCGATGTTC-

TTCACCGGTGCACTTCGTCTGG--TTTAGGCCAGCATCGGTTTT---
CGTAGGGGGAGAAAGGCCTGGGGAACGTATCTC----CCTCGGGAGTGTT-
ATAGCCCCTAGCGTAATACCCTTAC-GGGGACCGAGGACCGCGCT-TCG---
GCAAGGATGCTGGCATAATGGTCGTCAACGACCCGTCTTGAAACACGGACCAAGGAGTCGAACAT
TTGTGCGAGTGTTTGGGTA--TTAAACCCTCACGCGTAATGAAAGTGAACGGAGGTGAGAGCCCTT--
---CG---GGGTGCATCATCGACCGATCCTGATG-
TCTTCGGATGGATTTGAGTAAGAGCATAACTGTTCGGACCCGAAAGATGGTGAACTATGCGTGGAT
AGGGTGAAGCCAGAGGAAACTCTGGTGGAGGCTCGCAGCGGTTCTGACGTGCAAATCGATCGTCA
AATCTGCGCATGGGGGCGAAAG--

>Daldinia_andina

----------GTTATCTAAACT-CCAACCCTTTGTGAACCTTACCGTCGTTGCCTCGGCG---
GGCGCGCTTACCCTGTAGCTACCCT----------------------------GTAGCTACCCGGTAGGTGCGC-
TCCAAGCCCGCCGGTGGACCACTAAACTCTGTT-
TTAATATCGAATCTCTGAATGCTTCAACTTAATAAGTTAAAACTTTCAACAACGGATCTCTTGGTTC
TGGCATCGATGAAGAACGCAGCGAAATGCGATAAGTAATGTGAATTGCAGAATTCAGTGAATCAT
CGAATCTTTGAACGCACATTGCGCCCATTAGTATTCTAGTGGGCATGCCTATTCGAGCGTCATATCA
ACCCTTAAGCCCTAGCTGCTTAGCGTTGGAAGTCTGCGCTGTGCGCGCAAGTTCCTTAAAGTGATTG
GCGGAGTTGGGGTATACTCTAAGCGTAGTAATCTTTCTCGCTTCTGGAGTTGCCCCGACCTCTTGCC
GTTAAACCCC-TATATTTCTAGTGGTTGACCTC--------------------------------
TGAAGCGGCAACAGCTCAAA-TTTGAAATCTGGCC----CTA-----
GCGGTCCGAGTTGTAATTTGTAGAGGATGCTTTTGGTTAGGT-
GCCTTCCGAGTTCCCTGGAACGGGACGCCAGAGAGGGTGAGAGCCCCGTACGGTTGGA-
CACCGAGCCTCTATATAGCTCCTTCGACGAGTCGAGTAGTTTGGGAATGCTGCTCTAAATGGGAGG
TAAATTTCTTCTAAAGCTAAATACCGGCCAGAGACC-
GATAGCGCACAAGTAGAGTGATCGAAAGATGAAAAGTACTTTGAAAAGAGGGTTAAATAGCACGT
GAAATTGTTGAAAGGGAAGCG-TTTGCGACCAGACTTTTTCCAGGCGGATCATCCGGTGTTC--
TCACCGGTGCACTTCGCCTGG--TTTAGGCCAGCATCGGTTCT---
CTTAGGGGGATAAAGGCCTGGGGAACGTAGCTC----CTTCGGGAGTGTT-
ATAGCCCCTAGCGTAATACCCTTCG-GGGGACCGAGGAACGCGCA-TCT---
GCAAGGATGCTGGCGTAATGGTCGTCAACGACCCGTCTTGAAACACGGACCAAGGAGTCGAACAT
TTGTGCGAGTGTTTGGGTG--TTAAACCCTCACGCGTAATGAAAGTGAACGGAGGTGAGAGCCCTT--
---CG---GGGTGCATCATCGACCGATCCTGATG-
TCTTCGGATGGATTTGAGTAAGAGCATAACTGTTCGGACCCGAAAGATGGTGAACTATGCGTGGAT
AGGGTGAAGCCAGAGGAAACTCTGGTGGAGGCTCGCAGCGGTTCTGACGTGCAAATCGATCGTCA
AATCTGCGCATGGGGGCGAAAGACTTATCGA-

ACCATCTAGTAGCTGGTTACCGCCGAAGTTTCCCTCAGGATAGCAGTGT--TG-
TCTTCAGTTTTATGAGGTAAAGCGAATGATTAGGGACTCGGGGGCGCTATATTGCCTTCATCCATTC
TCAAACTTTAAATATGTAAGAAGCCCTTGTTACTTAATTGAACGTGGGCATTCGAATGTACCAACA
CTAGTGGGCCATTTTTGGTAAGCAGAACTGGCGATGCGGGATGAACCGAACGCGGGGTTAAGGTG
CCAGAGTGGACGCTCATCAGACACCACAAAAGGTGTTAGTACATCTTGACAGCAGGACGGTGGCC
ATGGAAGTCGGAATCCGCTAAGGACTGTGTAACAACTCACCTGCCGAATGTACTAGCCCTGAAAAT
GGATGGCGCTCAAGCGTCT-CACCCATACCTCGCCCTTAGGGTAGAAACGATGCCCTAAGG-----------
----
--------------------------CCTCGA---
CTCTTTCCCATCTGAGGCGCACGAACACGCCCATTGGAAGAGACGGGAAACTCGCGAAGCCCCGA
CAGCTGCATAATACCCATTGGGGTCTGGTCTGTCCGGCCGAAACGCCAGAGGGCCAAGCTTGTGGG
TTAGTGAAGAATCTATCGCTTATGTGCTCTATCAGCGTGGGTACGTCGACGGATCCCATCGTAGACT
ATATGATTACTAGGAATATGGAAGTCTTGGAGGAATACGAACCCATGAGATACCCTAATGCTACCA
AGATCTTTCTCAACGGATCTTGGATCGGTGTGCACCAGGATCCCAAGTCTCTAGTCAGAGACGTCC
AGCAACTTCGTCGGGCCAACCAGATCCCCTCCGAAGTGTCGCTAGTTCGCGATATCCGTGATCGCG
AGTTCAAGATCTTCTCAGATGCCGGTCGTGTCATGCGGCCCTTATTTGTTGTCCAGCAAGAGGAT----
----
AATCCCGAGGCTGGCACTACCAAGGGCTCGCTAGCTCTCAACAAGGAGATGATCCAGAGGCTGGA
AGCGGATGTCGATCTAGACCCCGAAAGCGAGGAGTACTTTGGCTGGCAAGGTCTAGTCAACGAGG
GTGTTATCGAGTATCTCGATGCCGAGGAAGAAGAAACGGCTATGATTTGCATGACCCCTGAAGATT
TAGAAACTTACCGGATGAGCAAGCTCGGATACGATGTATCTCAGGACAACGGA------
GACGAAATTAACAAACGGCTCAAGACTAAGGTGAATCCTACGACGCACATGTATACACATTGTGA
GATCCATCCTAGCATGCTCTTAGGTATCTGCGCAAGCATCATCCCCTTCCCGGATCACAATCAGGTA
CCCCTGAACGCGTCCTCCAAACCCCTTTGGTTCTGCCTCTCGTATACAAACGCC---ACCGCATCTAC-
-----CTTTTTCCATTGCAATTACATCAAATTGTATAGCAGCGGTCGGAAATATTAAAGCTAACCGCG---
--TTTCTTCAATAGGTCCATCTTCAGACTGGCCAATGTGTAAGTAAC---------
AGCGATGATCGAAGAACCATGGACATAAAACACAGCGGGGCTCACACGAAAATGATAGGGTAACC
AAATCGGTGCTGCTTTCTGGCAAACCATCTCTAGCGAGCACGGTCTCGACAGCAATGGCGTGTATG
TATTCGAGTTGTCGATTCTTTCGCCAAGAATATTAACTGACCATGCATCAATAGCTACAACGGTACT
TCCGAGCTCCAGCTCGAGCGTATGAGCGTCTACTTCAACGAGGTATGAATTTGTAGGAACTAGGAG
TAAATAAATGGAGACGGTTGCTAATTACCTCTACGCGTACAGGCTTCTGGCAACAAGTATGTTCCT
CGTGCCGTTCTCGTCGATCTTGAGCCCGGTACCATGGATGCCGTCCGTGCTGGTCCCTTCGGTCAGC
TCTTCCGACCCGACAACTTCGTTTTCGGCCAATCCGGTGCCGGAAACAACTGGGCCAAGGGTCATT
ACACTGAGGGTGCTGAACTAGTTGACGCCGTTCTCGATGTCGTTCGTCGCGAGGCTGAAGGCTGTG
ACTGCCTCCAGGGTTTCCAGATCACCCACTCCCTCGGTGGTGGTACTGGTGCCGGTATGGGTACCCT
GTTGATCTCCAAGATCCGCGAGGAGTTCCCCGACCGCATGATGGCCACCTTCTCCGTCATGCCCTCC
CCCAAGGTTTCCGACACCGTTGTCGAGCCTTACAACGCCACCCTCTCCGTCCACCAGCTGGTCGAG
AACTCCGATGAGACCTTCTGTATCGATAACGAGGCCCTATACGACATCTGCATGCGCACCCTGAAG
CTGTCCAACCCCTCGTACGGTGACCTGAACCACCTGGTCTCTGCTGTCATGTCCGGCGTTACTACTT
GCTTGCGTTTCCCTGGTCAGCTGAACTCTGACCTGCGCAAGCTTGCCGTAAACATGGTTCCTTTCCC
CCGTCTCCATTTCTTTATGGTCGGCTTCGCTCCTCTGACCAGCCGTGGTGCTCACTCTTTCCGTGCTG
TCACCGTCCCTGAGTTGACCCAGCAGATGTTCGACCCTAAGAACATGATGGCTGCTTCTGACTTCCG
TAACGGTCGCTACCTGACGTGCTCAGCCATCTTGTATGATATATCCCCTTAATTTGTATATAGTGCT
TATTTGCTAATTTGAATTCTCCAGCCGTGGCAAGGTCTCAA

>Daldinia_childiae

-CATTACTGAGTTATCTAAACT-CCAACCCTATGTGAACCTTACCGTCGTTGCCTCGGCG---
GGCGCGTCTACCCTGTAGCTACCCT-----------------------------GTAGCTACCCGGTAGGCGTGC-
TACAAGCCCGCCGGTGGACCACTAAACTCTGTTATAAATACTGTATCTCTGAATGCTTCAACTTAAT
AAGTTAAAACTTTCAACAACGGATCTCTTGGTTCTGGCATCGATGAAGAACGCAGCGAAATGCGAT
AAGTAATGTGAATTGCAGAATTCAGTGAATCATCGAATCTTTGAACGCACATTGCGCCCATTAGTA
TTCTAGTGGGCATGCCTATTCGAGCGTCATTTCAACCCTTAAGCCTAAGTTGCTTAGCGTTGGGAAT
CTGCCCTGTACGGGGCAGTTCCCTAAAGTTATCGGCGGAGTTAGGGCATACTCTAAGCGTAGTACT
TATTCTCGCTTCTGCAGTTGTCCCGACGGCTTGCCGCTAAACCCC-
TATATTTCTAGTGGTTGACCTCGGATTAGGTAGGAATACCCGCTGAACTTAAGCATATGAAGCGGC
AACAGCTCAAA-TTTGAAATCTGGCC----CTA-----
GCGGTCCGAGTTGTAATTTGTAGAGGATGCTTTTGGTTAGGT-

GCCTTCCGAGTTCCCTGGAACGGGACGCCAGAGAGGGTGAGAGCCCCGTACGGTTGGA-
CACCGAGCCTCTATATAGCTCCTTCGACGAGTCGAGTAGTTTGGGAATGCTGCTCTAAATGGGAGG
TAAATTTCTTCTAAAGCTAAATACCGGCCAGAGACC-
GATAGCGCACAAGTAGAGTGATCGAAAGATGAAAAGTACTTTGAAAAGAGGGTTAAATAGCACGT
GAAATTGTTGAAAGGGAAGCG-TTTGCGACCAGACTTTTTCCAGGCGGATCATCCGGTGTTC--
TCACCGGTGCACTTCGCCTGG--TTTAGGCCAGCATCGGTTCC---
CTTAGGGGGATAAAGGCCTGGGGAACGTAGCTC----CTTCGGGAGTGTT-
ATAGCCCCTAGCGTAATACCCTTCG-GGGGACCGAGGAACGCGCA-TCT---
GCAAGGATGCTGGCGTAATGGTCGTCAACGACCCGTCTTGAAACACGGACCAAGGAGTCGAACAT
TTGTGCGAGTGTTTGGGTG--TTAAACCCTCACGCGTAATGAAAGTGAACGGAGGTGAGAGCCCTT--
---CG---GGGTGCATCATCGACCGATCCTGATG-
TCTTCGGATGGATTTGAGTAAGAGCATAACTGTTCGGACCCGAAAGATGGTGAACTATGCGTGGAT
AGGGTGAAGCCAGAGGAAACTCTGGTGGAGGCTCGCAGCGGTTCTGACGTGCAAATCGATCGTCA
AATCTGCGCATGGGGGCGAAAGACTTATCGA-ACCATCTAGTAGCTGGTTACCGC----------------------
----

ACGTTGTATCGATTCAGGCAGGCGTTTCCAGATTGAGCTCGCCGCCAAGCCTGCTATTGTAACCAA
CGGGCTGAAGTACTCTCTCGCCACAGGCAACTGGGGTGACCAGAAGAAGGCGATGAGCTCGACAG
CTGGTGTATCGCAAGTTCTGAACCGATACACGT------TCGCCTCGA---
CTCTTTCTCATTTAAGGCGCACGAACACGCCTATCGGAAGAGACGGGAAACTCGCGAAGCCTCGAC
AACTTCACAATACCCATTGGGGCCTGGTCTGTCCGGCCGAAACGCCCGAAGGCCAAGCTTGTGGTT
TGGTGAAGAATTTGTCGCTTATGTGCTCTATCAGCGTGGGTACGTCAACAGATCCTATCGTAGACTA
TATGATTACTAGGAATATGGAAGTCTTGGAGGAATATGAACCGATGAGATACCCCAATGCCACCAA
GATCTTTCTTAACGGATCTTGGATCGGTGTGCACCAGGATCCCAAGTCTCTAGTCAGAGACGTTCA
GCAACTTCGTCGGGCCAACCAGATCCCCTCTGAAGTATCGCTGGTTCGCGACATCCGTGATCGCGA
GTTCAAAATCTTCTCGGATGCTGGTCGTGTCATGCGGCCCTTATTTGTTGTGCAGCAAGAGGAT------
----
AATCCCGAGGCTGGTACTACGAAGGGCTCGCTAGCTATCAATAAGGAGATGATCGGGAGGCTGGA
GGCGGATGTCGATGTAGACCCTGAAAGCGAGGGGTACTTTGGCTGGCAGGGTCTGGTCAACGAGG
GTGTTATCGAGTACCTCGATGCCGAAGAAGAAGAAACGGCTATGATTTGCATGACCCCTGAAGATT
TGGAAACCTACCGGATGAGCAAGCTCGGATACGATGTGTCCCAGGATAATGGA------
GATGAGATTAATAAGCGACTCAAAACTAAGGTGAATCCTACAACGCACATGTATACGCATTGTGAG
ATTCATCCTAGCATGCTCCTGGGTATCTGCGCGAGCATTATTCCCTTCCCAGACC----------------------
----

ATGTATTCGAGTTGTCTACTCTATTATTAAGAATATCAACTAATCGTCAATCAACAGTTACAACGGT
ACTTCCGAGCTCCAGCTCGAGCGCATGAGCGTCTACTTCAACGAGGTACGAATTTATAGAAATTAA
AGATAAATAAATGAAGATGATTGCTAATTGCCTCTACGCGTGCAGGCTTCCGGTAACAAGTATGTT
CCTCGTGCCGTCCTCGTCGATCTCGAGCCCGGTACCATGGACGCCGTCCGTGCTGGTCCCTTCGGTC
AGCTCTTCCGACCCGACAACTTCGTCTTCGGTCAATCCGGTGCCGGAAACAACTGGGCCAAGGGTC
ATTACACTGAGGGTGCCGAATTGGTTGACCAAGTTCTCGATGTCGTTCGTCGTGAGGCTGAAGGCT
GTGACTGCCTCCAGGGTTTCCAGATTACCCACTCCCTCGGTGGTGGTACTGGTGCCGGTATGGGTAC
CCTATTGATCTCCAAGATCCGCGAGGAGTTTCCCGACCGCATGATGGCTACCTTCTCCGTTATGCCT
TCCCCTAAGGTTTCCGATACCGTTGTCGAGCCTTATAACGCCACCCTCTCTGTCCACCAGCTGGTCG
AGAACTCCGATGAGACCTTCTGTATCGACAACGAGGCTCTGTACGACATCTGCATGCGTACGCTGA
AGCTGTCCAACCCCTCGTACGGTGACCTGAACCACCTGGTCTCTGCTGTCATGTCCGGCGTTACTAC
TTGCTTGCGTTTCCCTGGTCAGCTAAACTCTGACCTGCGCAAGCTTGCCGTGAACATGGTTCCTTTC
CCTCGTCTCCACTTCTTCATGGTCGGCTTCGCCCCCCTGACCAGCCGTGGCGCTCACTCTTTCCGTGC
CGTCACCGTCCCTGAATTGACTCAGCAGATGTTCGACCCCAAGAACATGATGGCTGCTTCCGATTTC
CGTAACGGTCGTTACCTGACGTGCTCAGCCATCTTGTATGATTATCCCCTTTAAAATTTATACACTA
CTTGTTTGCTAACTTAAATTCTCTAGCCGTGGCAAGGTCTCGA

>Daldinia_loculatoides

-CATTACTGAGTTATCTAAACTCCCAACCCTTTGTGAACCTTACCGTCGTTGCCTCGGCG---
GGCGTACTTACCCTGTAGCTACCCT----------------------------GTAGCTACCCGGTAGGTGCGC-

TCCAAGCCCGCCGGTGGACCACTAAATTCTATT-
TTACTACTGTATCTCTGAATGCTTCAACTTAATAAGTTAAAACTTTCAACAACGGATCTCTTGGTTC
TGGCATCGATGAAGAACGCAGCGAAATGCGATAAGTAATGTGAATTGCAGAATTCAGTGAATCAT
CGAATCTTTGAACGCACATTGCGCCCATTAGTATTCTAGTGGGCATGCCTATTCGAGCGTCATTTCA
ACCCTTAAG-
CCTAGTTGCTTAGCGTTGGGAATCTGCCCTGTACAGGGCAGTTCCTTAAAGTGATTGGCGGAGTTA
GGGCATACTCTAAGCGTAGTAATTCTTCTCGCTTCTGTAGTTGTCCTGGCGGCTTGCCGTTAAACCC
C-
TATATTTCTAGTGGTTGACCTCGGATTAGGTAGGAATACCCGCTGAACTTAAGCATATGAAGCGGC
AACAGCTCAAA-TTTGAAATCTGGCC----CTA-----
GCGGTCCGAGTTGTAATTTGTAGAGGATGCTTTTGGTTAGGT-
GCCTTCTGAGTTCCCTGGAACGGGACGCCAGAGAGGGTGAGAGCCCCGTACGGTTGGA-
CACCGAGCCTCTATATAGCTCCTTCGACGAGTCGAGTAGTTTGGGAATGCTGCTCTAAATGGGAGG
TAAATTTCTTCTAAAGCTAAATACCGGCCAGAGACC-
GATAGCGCACAAGTAGAGTGATCGAAAGATGAAAAGTACTTTGAAAAGAGGGTTAAATAGCACGT
GAAATTGTTGAAAGGGAAGCG-TTTGCGACCAGACTTTTTCCAGGCGGATCATCCGGTGTTC--
TCACCGGTGCACTTCGCCTGG--TTTAGGCCAGCATCGGTTCT---
CTTAGGGGGATAAAGGCTTGGGGAACGTAGCTC----CCTCGGGAGTGTT-
ATAGCCCCTCGCGTAATACCCTTCG-GGGGACCGAGGAACGCGCA-TCT---
GCAAGGATGCTGGCGTAATGGTCGTCAACGACCCGTCTTGAAACACGGACCAAGGAGTCGAACAT
TTGTGCGAGTGTTTGGGTG--TTAAACCCTCACGCGTAATGAAAGTGAACGGAGGTGAGAGCCCTT--
---CG---GGGTGCATCATCGACCGATCCTGATG-
TCTTCGGATGGATTTGAGTAAGAGCATAACTGTTCGGACCCGAAAGATGGTGAACTATGCGTGGAT
AGGGTGAAGCCAGAGGAAACTCTGGTGGAGGCTCGCAGCGGTTCTGACGTGCAAATCGATCGTCA
AATCTGCGCATGGGGGCGAAAGACTTATCGA-
ACCATCTAGTAGCTGGTTACCGCCGAAGTTTCCCTCAGGATAGCAGTGT--TG-
TCTTCAGTTTTATGAGGTAAAGCGAATGATTAGGGACTCGGGGGCGCTATATTGCCTTCATCCATTC
TCAAACTTTAAATATGTAAGAAGCCCTTGTTACTTAATTGAACGTGGGCATTCGAATGTACCAACA
CTAGTGGGCCATTTTTGGTAAGCAGAACTGGCGATGCGGGATGAACCGAACGCGGGGTTAAGGTG
CCAGAGTGGACGCTCATCAGACACCACAAAAGGTGTTAGTACATCTTGACAGCAGGACGGTGGCC
ATGGAAGTCGGAATCCGCTAAGGACTGTGTAACAACTCACCTGCCGAATGTACTAGCCCTGAAAAT
GGATGGCGCTCAAGCGTCT-CACCCATACCTCGCCCTTAGGGTAGAAACGATGCCCTAAGG----------
----
--------GATACACGT------
TCGCTTCGACTCCTTTCCTCCATCTAAGGCGGACGAACACGCCTATTGGAAGAGATGGAAAACTTG
CGAAACCTCGACAGCTGCATAATACCCATTGGGGTCTGGTCTGTCCGGCCGAAACGCCCGAAGGCC
AAGCTTGTGGGCTAGTGAAAAATCTATCGCTTATGTGCTCTATCAGCGTGGGTACGTCAACGGATC
CTATCGTAGACTATATGATTACTAGGAATATGGAAGTCTTGGAGGAATACGAACCTATGCGATACC
CTAATGCCACCAAGATCTTCCTCAACGGATCTTGGATCGGTGTGCACCAGGATCCCAAGTCTCTAG
TTAGAGACGTCCAGCAACTTCGTCGGGCTAACCAGATCCCCTCTGAAGTGTCGCTAGTTCGCGATA
TCCGTGATCGCGAGTTCAAGATCTTCTCAGATGCTGGTCGTGTCATGCGACCCTTATTTGTTGTGCA
GCAAGAGGAT---
AATCCCGAGGCTGGTACTACGAAGGGCTCGTTGGCTCTCAACAAGGAGATGATCCAGAGGCTGGA
GGCAGATGTCGAACTAGACCCTGAAAGCGAGGAATACTTTGGTTGGCAAGGCCTCGTTAACGAGG
GGGTTATCGAGTATCTCGACGCCGAAGAAGAAGAGACGGCTATGATTTGCATGACTCCTGAAGATT
TAGAAACCTACCGGATGAGCAAGCTCGGATACGATGTGTCGCAGGATAACGGC------
GATGAGATTAACAAGCGTCTAAAGACTAAAGTGAATCCTACGACGCACATGTATACACATTGCGA
GATCCATCCCAGCATGCTCCTGGGTATCTGCGCAAGCATCATTCCCTTCCCGGACCACAATCAGGT
ACCCCTGAACGCGTCCCCCAAACACCCTTAGTTCTACCCCTCATACACAAACGCT---ACCATATCTA-
------CATTTTATATTGCAACTACGTCAAATTGTATACCAACGGTCGGAAAAATCAAAGCTAACCGCG-
----TTTCTTCAATAGGTTCATCTTCAGACTGGCCAATGTGTAAGTAAC---------
AGCGATCATCGAATAACTATGTATATAAGACACAGCGGGGCTCACATGGAGATGATAGGGTAACC
AAATCGGTGCCGCTTTCTGGCAAACCATCTCCAGCGAGCACGGTCTCGACAGCAATGGAGTGTATG
TATTCGAATTGTTGATTCTATGGACAAGAATATCAATTAATCATCCATCAACAGTTACAACGGTACT
TCCGAGCTCCAGCTCGAGCGCATGAGCGTCTACTTCAACGAGGTATGAATTTGTAGGAACTAGGGT
TAAATAAATGGAGACAATGGCTAATTGCTTCAACGCGTGCAGGCTTCTGGCAACAAGTATGTTCCC
CGTGCCGTCCTCGTCGATCTCGAGCCCGGTACCATGGACGCCGTCCGTGCTGGTCCCTTTGGTCAGC

TCTTCCGGCCCGACAACTTCGTTTTCGGTCAGTCCGGTGCCGGAAACAACTGGGCCAAGGGTCATT
ACACTGAGGGTGCTGAGTTGGTTGACCAAGTCCTCGATGTCGTTCGTCGTGAGGCTGAAGGCTGTG
ACTGCCTCCAGGGTTTCCAGATCACCCACTCCCTCGGTGGTGGTACTGGTGCCGGTATGGGTACTCT
GTTGATCTCCAAGATCCGCGAGGAGTTCCCCGATCGCATGATGGCCACCTTCTCCGTCATGCCCTCC
CCTAAGGTTTCCGATACCGTTGTCGAGCCTTACAACGCCACCCTCTCCGTCCACCAGCTGGTCGAGA
ACTCCGATGAGACCTTCTGTATCGATAACGAGGCTCTGTACGACATCTGCATGCGCACGCTAAAGC
TGTCCAACCCCTCGTACGGTGACCTGAACCACCTGGTCTCCGCCGTCATGTCCGGCGTTACTACTTG
CTTGCGTTTCCCTGGTCAGCTGAACTCTGACCTGCGCAAGCTTGCCGTGAACATGGTTCCTTTCCCT
CGTCTCCATTTCTTCATGGTTGGCTTCGCTCCCCTGACCAGCCGTGGCGCTCACTCTTTCCGTGCCGT
CACCGTCCCTGAGTTGACTCAGCAGATGTTCGACCCCAAGAATATGATGGCTGCTTCTGACTTCCGT
AACGGTCGCTACCTGACGTGCTCAGCCATCTTGTAAGATACCCCCTTTAAAT-
TTTATACGTTTATTGTTTGCTAATTCAGGTTCTCTAGCCGTGGAAAGGTTTCAA

>Daldinia_vernicosa

-CATTACTGAGTTATCTAAACTCCCAACCCTATGTGAACCTTACCGTCGTTGCCTCGGCG---
GGCGTGCTTACCCGGTAGCTACCCT----------------------------GTAGCTACCCGGTAGGTACGC-
TGCAAGCCCGCCGGTGGACCACTAAACTCTGTT-
TAATTACTGTATCTCTGAATGCTTCAACTTAATAAGTTAAAACTTTCAACAACGGATCTCTTGGTTC
TGGCATCGATGAAGAACGCAGCGAAATGCGATAAGTAATGTGAATTGCAGAATTCAGTGAATCAT
CGAATCTTTGAACGCACATTGCGCCCGCTAGTATTCTAGCGGGCATGCCTGTCCGAGCGTCATTTCA
ACCCTTAAG-
CCTAGCTGCTTAGTGTTGGGAATCTGCCCTGCGCAGCGCAGTTCCTTAAAGTAATCGGCGGAGTTA
GGGCATACTCTAAGCGTAGTAATTCTTCTCGCTTCTGTAGTTGTCCTGGCGGCTTGCCGTTAAACCC
C-TATATTTCTAGTGGTTGACCTCGGATCAGGTAGGAGTACCCGCTGAACTTAAGCATA-----------------
-----------------------------------GAGTTGTAATTTGTAGAGGATGCTTTTGGTTAGGT-
ACCTTCCGAGTTCCCTGGAACGGGACGCCAGAGAGGGTGAGAGCCCCGTACGGTTGGA-
TACCGAGCCTCTATATAGCTCCTTCAACGAGTCGAGTAGTTTGGGAATGCTGCTCTAAATGGGAGG
TAAATTTCTTCTAAAGCTAAATACCGGCCAGAGACC-
GATAGCGCACAAGTAGAGTGATCGAAAGATGAAAAGTACTTTGAAAAGAGGGTTAAATAGCACGT
GAAATTGTTGAAAGGGAAGCG-TTTGCGACCAGACTTTTTCCGGGCGGATCATCCGGTGTTC--
TCACCGGTGCACTTCGCCCGG--TTTAGGCCAGCATCGGTTCT---
CCTAGGGGGATAAAGGCGGGGGGAACGTGGCTC----CTTCGGGAGTGTT-
ATAGCCCCCCGCGTAATACCCCTCG-GGGGACCGAGGAACGCGCA-TCT---
GCAAGGATGCTGGCGTAATGGTCGCCAACGACCCGTCTTGAAACACGGACCAAGGAGTCGAACAT
TTGTGCGAGTGTTTGGGTG--TCAAACCCTCACGCGTAATGAAAGTGAACGGAGGTGAGAGCCCCT--
---CG---GGGTGCATCATCGACCGATCCTGATG-
TCTTCGGATGGATTTGAGTAAGAGCATAAATGTTCGGACCCGAAAGATGGTGAACTATGCGTGGAT
AGGGTGAAGCCAGAGGAAACTCTGGTGGAGGCTCGCAGCGGTTCTGACGTGCAAATCGATCGTCA
AATCTGCGCATGGGGGCGAAAGACTTATCGA-
ACCATCTAGTAGCTGGTTACCGCCGAAGTTTCCCTCAGGATAGCAGTGT--TG-
TCTTCAGTTTTATGAGGTAAAGCGAATGATTAGGGACTCGGGGGCGCTATATTGCCTTCATCCATTC
TCAAACTTTAAATATGTAAGAAGCCCTTGTTACTTAATTGAACGTGGGCATTCGAATGTACCAACA
CTAGTGGGCCATTTTTGGTAAGCAGAACTGGCGATGCGGGATGAACCGAACGCGGGGTTAAGGTG
CCAGAGTGGACGCTCATCAGACACCACAAAAGGTGTTAGTACATCTTGACAGCAGGACGGTGGCC
ATGGAAGTCGGAATCCGCTAAGGACTGTGTAACAACTCACCTGCCGAATGTACTAGCCCTGAAAAT
GGATGGCGCTCAAGCGTCT-CACCCATACCTCGCCCTTAGGGTAGAAACGATGCCCTAAGG-----------
----
----GACCGATACACGT------TCGCCTCGA---
CTCTTTCTCATTTAAGGCGTACGAACACGCCTATTGGAAGAGACGGGAAACTCGCAAAGCCTCGAC
AACTTCACAATACCCATTGGGGTCTGGTCTGTCCGGCCGAAACGCCCGAGGGCCAAGCTTGCGGGC
TGGTAAAGAATTTGTCGCTTATGTGCTCCATCAGCGTGGGTACGTCAACAGATCCTATCGTAGACT
ATATGATTACTAGGAATATGGAAGTCTTGGAGGAATACGAACCGATGAGATACCCTAATGCCACCA
AGATCTTCCTTAACGGATCTTGGATCGGTGTGCACCAGGATCCCAAGTCTCTAGTTAGAGACGTCC
AGCAACTTCGTCGGGCCAACCAGATCCCCTCTGAAGTGTCGCTGGTTCGCGATATCCGTGATCGCG
AATTCAAGATCTTCTCGGATGCTGGTCGTGTCATGCGGCCCTTATTTGTTGTGCAGCAAGAGGAT----
----
AATCCCGAGGCTGGTACTACGAAGGGCTCGCTAGCTCTCAGTAAAGAGATGATCCAGAGGCTAGA

AGCAGATGTCGAACTAGACCCTGAAAGCGAGGAGTACTTTGGCTGGCAAGGTCTAGTCAACGAGG
GTGTTATCGAGTACCTCGATGCCGAGGAAGAAGAAACGGCCATGATTTGCATGACCCCCGAAGATT
TAGAAACCTACCGGATGAGCAAGCTCGGATACGATGTGTCTCAGGATAATGGA------
GATGAGATTAATAAGCGGCTCAAGACTAAGGTCAATCCTACGACGCACATGTATACGCATTGTGAG
ATCCATCCCAGTATGCTCCTGGGTATCTGCGCGAGTATTATTCCCTTCCCGGACCACAATCAGGTA--
----
----TTTTGTCAATAGGTTCATCTTCAGACTGGCCAATGTGTAAGTAAA---------
AGCGATCATCGAAGAACCATGGATATAAAATACAGCGGGGCTCACATGAAGATGATAGGGTAACC
AAATCGGTGCCGCTTTCTGGCAAACCATCTCCAGCGAGCACGGTCTCGACAGCAATGGCGTGTATG
TATTCGAGTTGCCCATTCTGTCACCAAGAATATCAACTAATCATCAATCAACAGTTACAACGGTACT
TCCGAGCTCCAGCTCGAGCGCATGAGCGTCTACTTCAACGAGGTACGAATTTATAGGAACTAGGGA
TAAATAAATGGAGAGAATTGCTAATTGCCTCTACGCCTGCAGGCTTCCGGCAACAAGTATGTTCCT
CGTGCCGTCCTCGTCGATCTCGAGCCCGGTACCATGGACGCCGTCCGTGCTGGTCCCTTCGGTCAGC
TCTTCCGACCCGACAACTTCGTCTTCGGTCAATCCGGTGCCGGAAACAACTGGGCCAAGGGTCATT
ACACTGAGGGTGCCGAGCTGGTTGACCAAGTTCTCGATGTCGTTCGTCGTGAGGCTGAAGGCTGTG
ACTGCCTTCAGGGTTTCCAGATCACTCACTCCCTCGGTGGTGGTACTGGTGCCGGTATGGGTACCCT
ATTGATCTCCAAGATCCGCGAGGAGTTCCCCGACCGCATGATGGCCACCTTCTCCGTCATGCCCTCT
CCTAAGGTTTCTGACACCGTTGTCGAGCCTTACAACGCCACCCTCTCCGTCCACCAGCTGGTCGAGA
ACTCCGACGAGACCTTCTGTATCGACAACGAGGCTCTGTACGACATCTGCATGCGCACGCTGAAGC
TGTCCAACCCTTCGTACGGTGACCTGAACCACCTGGTCTCTGCTGTCATGTCCGGCGTTACTACTTG
CTTGCGTTTCCCTGGCCAGCTGAACTCTGACCTGCGCAAGCTTGCCGTAAACATGGTTCCTTTCCCT
CGTCTCCACTTCTTCATGGTCGGCTTCGCTCCCCTGACCAGCCGTGGTGCTCACTCTTTCCGTGCCGT
CACCGTCCCTGAGTTGACTCAGCAGATGTTC---

>Daldinia_petriniae

-CATTACTGAGTTATCTAAACTCCCAACCCTATGTGAACCTTACCGTCGTTGCCTCGGCG---
GGCGCGCTTACCCGGTAGCTACCCT----------------------------GTAGCTACCCGGTAGGTGCGC-
TATAGGCCCGCCGGTGGACTACTCAACTCTGTTTTTAATACTGTATCTCTGAATGCTTCAACTTAAT
AAGTTAAAACTTTCAACAACGGATCTCTTGGTTCTGGCATCGATGAAGAACGCAGCGAAATGCGAT
AACTAATGTGAATTGCAGAATTCAGTGAATCATCGAATCTTTGAACGCACATTGCGCCCATTAGTA
TTCTAGTGGGCATGCCTATTCGAGCGTCATTTCAACCCTTAAG-
CCTAGCTGCTTAGTGTTGGGAACCTGCCCCGTACGGGGCAGCTCCCTAAAGTCATCGGCGGAGTTA
GGGCATACTCTAAGCGTAGTACTTCTTCTCGCTTCTGTAGTTGTCCTGGCGGCTTGCCGTTAAACCC
CCTATATTTCTAGTGGTTGACCTCGGATTAGGTAGGAATA----------------------------CAGCTCAAA-
TTTGAAATCTGGCC----CTA-----GCGGTCCGAGTTGTAATTTGTAGAGGATGCTTTTGGTTAGGT-
GCCTTCCGAGTTCCCTGGAACGGGACGCCAGAGAGGGTGAGAGCCCCGTACGGTTGGA-
CGCCGAGCCTCTGTATAGCTCCTTCGACGAGTCGAGTAGTTTGGGAATGCTGCTCTAAATGGGAGG
TAAATTTCTTCTAAAGCTAAATACCGGCCAGAGACC-
GATAGCGCACAAGTAGAGTGATCGAAAGATGAAAAGTACTTTGAAAAGAGGGTTAAATAGCACGT
GAAATTGTTGAAAGGGAAGCG-TTTGCGACCAGACTTTTTCTAGGCGGATCATCCGGTGTTC--
TCACCGGTGCACTTCGCCTGG--TTTAGGCCAGCATCGGCTCT---
CTTAGGGGGATAAAGGCCAGGGGAACGTAGCTC----CTTCGGGAGTGTT-
ATAGCCCCTAGCGTAATACCCTTCG-GGGGGCCGAGGAACGCGCA-TCT---
GCAAGGATGCTGGCGTAATGGTCGTCAACGACCCGTCTTGAAACACGGACCAAGGAGTCGAACAT
TTGTGCGAGTGTTTGGGTG--TCAAACCCTCACGCGTAATGAAAGTGAACGGAGGTGAGAGCCCTT--
---CG---GGGTGCATCATCGACCGATCCTGATG-
TCTTCGGATGGATTTGAGTAAGAGCATAACTGTTCGGACCCGAAAGATGGTGAACTATGCGTGGAT
AGGGTGAAGCCAGAGGAAACTCTGGTGGAGGCTCGCAGCGGTTCTGACGTGCAAATCGATCGTCA
AATCTGCGCATGGGGGCGAAAGACTTATCGA-
ACCATCTAGTAGCTGGTTACCGCCGAAGTTTCCCTCAGGATAGCAGTGT--TG-
TCTTCAGTTTTATGAGGTAAAGCGAATGATTAGGGACTCGGGGGCGCTATATTGCCTTCATCCATTC
TCAAACTTTAAATATGTAAGAAGCCCTTGTTACTTAATTGAACGTGGGCATTCGAATGTACCAACA
CTAGTGGGCCATTTTTGGTAAGCAGAACTGGCGATGCGGGATGAACCGAACGCGGGGTTAAGGTG
CCAGAGTGGACGCTCATCAGACACCACAAAAGGTGTTAGTACATCTTGACAGCAGGACGGTGGCC
ATGGAAGTCGGAATCCGCTAAGGACTGTGTAACAACTCACCTGCCGAATGTACTAGCCCTGAAAAT
GGATGGCGCTCAAGCGTCT-CACCCATACCTCGCCCTTAGGGTAGAAACGATGCCCTAAGG----------

----
TATTGGAAGAGACGGGAAACTCGCGAAGCCTCGACAACTTCACAATACCCATTGGGGTCTGGTCTG
TCCGGCCGAAACGCCCGAAGGCCAAGCTTGNGGTTTGGTGAAGAATTTGTCGCTCATGTGCTCTAT
CAGCGTGGGTACGTCAACGGATCCTATCGTAGACTATATGATTACTAGGAATATGGAAGTCTTGGA
GGAATACGAACCCATGAGATACCCCAACGCCACCAAGATCTTCCTTAACGGATCTTGGATCGGTGT
GCACCAGGATCCCAAGTCTCTAGTTAGAGACGTCCAGCAACTTCGTCGGGCCAACCAGATCCCCTC
CGAAGTGTCGCTGGTTCGCGATATCCGTGATCGCGAGTTCAAGATCTTCTCGGATGCTGGTCGTGTC
ATGCGGCCCTTATTTGTTGTGCAGCAAGAGGAT---
AATCCCGAGGCTGGTACTACGAAGGGCTCGCTAGCTCTCAATAAGGAGATGATCCAGAGGCTGGA
GGCGGATGTCGACTTGGACCCTGAAAGCGATGAGTACTTTGGCTGGCAGGGCCTGGTCAACGAGG
GTGTTATCGAGTACCTCGATGCCGAGGAAGAAGAAACGGCTATGATCTGCATGACGCCTGAAGATC
TAGAAACCTACCGGATGAGCAAGCTCGGATACGATGTGTCCCAGGATAATGGA------
GATGAGATTAATAAGCGGCTCAAGACTAAAGTGAATCCTACGACGCACATGTATACACATTGTGAG
ATTCATCCTAGCATGCTCCTGGGTATCTGCGCAAGCATTATTCCCTTCCCGGACCACAATCAGGTAC
CCCTGAACGCGTCCCTCAAACCCCCTTAGTTCTGCCCCTCATACACAAACACT---ACCGCATCTA-----
--CATTTCATATTGCAACTACATCAAATTGTACAGCAACGGTCGGAAAATTCAAAGCTAACCGCG-----
TTTCTTCAATAGGTTCATCTTCAGACCGGCCAATGTGTAAGTAAC---------
AGCGATGATGGAAGAACCATGGATATAAAATACAGCGGGGCTTACATGAAGATGATAGGGTAACC
AAATTGGTGCTGCTTTCTGGCAAACCATCTCCAGCGAGCACGGTCTCGACAGCAATGGCGTGTATG
TATTCGAGTTATCTATTCTATTACCAAGAATATCAACTAATCATCAATCAACAGTTACAACGGTACT
TCCGAGCTCCAGCTCGAGCGCATGAGCGTCTACTTCAACGAGGTAGGAATTTATAAGAAT-
AGGAATAAATAAATGGAGATAATTGCTAATTGTCTCCATGCGTGCAGGCTTCCGGCAACAAGTATG
TTCCTCGTGCCGTCCTCGTCGATCTCGAGCCCGGTACCATGGACGCCGTCCGTGCTGGTCCCTTCGG
TCAGCTCTTCCGACCCGACAACTTCGTCTTCGGTCAATCCGGTGCCGGAAACAACTGGGCCAAGGG
TCATTACACCGAGGGTGCCGAGTTGGTTGACCAGGTTCTCGATGTCGTCCGTCGTGAGGCTGAGGG
TTGTGATTGCCTCCAGGGTTTCCAGATCACCCACTCCCTCGGTGGTGGTACTGGTGCCGGTATGGGT
ACCCTGTTGATCTCCAAGATCCGCGAGGAGTTCCCCGACCGCATGATGGCCACCTTCTCCGTCATGC
CCTCACCCAAGGTTTCCGACACCGTCGTCGAGCCTTACAACGCCACCCTCTCCGTCCACCAGCTGGT
CGAGAACTCCGATGAGACCTTCTGTATCGACAACGAGGCTCTGTACGACATCTGCATGCGTACGCT
GAAGCTGTCCAACCCCTCGTACGGTGACCTGAACCACTTGGTCTCTGCTGTCATGTCCGGCGTTACT
ACTTGCTTGCGTTTCCCTGGTCAGCTGAACTCTGACCTGCGCAAGCTTGCCGTGAACATGGTTCCTT
TCCCTCGTCTCCACTTCTTCATGGTCGGCTTCGCCCCCCTGACCAGCCGTGGCGCTCACTCTTTCCGT
GCCGTCACCGTCCCCGAGTTGACTCAGCAGATGTTCGACCCCAAGAACATGATGGCTGCTTCTGAT
TTCCGTAACGGTCGCTACCTGACGTGCTCAGCCATTTTGTATGATGATATCCCCTAAATTTTATACG
TTACTTGTTTGCTAACTTGAATTCTCTAGCCGTGGCAAGGTCTCGA

>Entonaema_liquescens

-CATTACTGAGTTATCTAAACT-CCAACCCTATGTGAA-CCTACCGTCGTTGCCTCGGCG---
GGCGCGCTTACCCGGTAGCTACCCT-----------------------------GTAGCTACCCGGT-AGCGCGT-
TCCAAGCCCGCCGGTGGACCACTAAACTCTGTTATATATACTGTATCTCTGAATGCTTCAAACTTAA
TAGTTAAAACTTTCAACAACGGATCTCTTGGTTCTGGCATCGATGAAGAACGCAGCGAAATGCGAT
AAGTAATGTGAATTGCAGAATTCAGTGAATCATCGAATCTTTGAACGCACATTGCGCCCATTAGTA
TTCTAGTGGGCATGCCTATTCGAGCGTCATTTCAACCCTTAAGCCCCTGTTGCTTAGTGTTGGGAAT
CTGCGTCTTAGGGCGCAGTTCCTTAAAGTGATTGGCGGAGTTAGGGCATACTCTGAGCGTAGTAAT
TCTTCTCGCTTCTGAAGTTGTCCTGGCGGCCGGCCGTAAAACCCC-
TATATCTTTAGTGGTTGACCTCGAATTAGGTAGGAATACCC---------------
TGAAGCGGCAACAGCTCAAA-TTTGAAATCTGGCC----CTA-----
GCGGTCCGAGTTGTAATTTGTAGAGGATGCTTTTGGTGAGGT-
GCCTTCCGAGTTCCCTGGAACGGGACGCCAGAGAGGGTGAGAGCCCCGTACGGTTGGA-
CGCCAAACCTCTATATAGCTCCTTCGACGAGTCGAGTAGTTTGGGAATGCTGCTCTAAATGGGAGG
TAAATTTCTTCTAAAGCTAAATACCGGCCAGAGACC-
GATAGCGCACAAGTAGAGTGATCGAAAGATGAAAAGCACTTTGAAAAGAGGGTTAAATAGCACGT
GAAATTGTTGAAAGGGAAGCG-TTTGCGACCAGACTTTTTCCAGGCGGATCATCCGGTGTTC--
TCACCGGTGCACTTCGCCTGG--TTTAGGCCAGCATCGGTTTC---
CTTAGGGGGATAAAGGCTTGGGGAACGTAGCTC----CTTCGGGAGTGTT-
ATAGCCCCTTGCGTAATACCCCTCG-GGGGACCGAGGAACGCGCT-TCG---

GCAAGGATGCTGGCGTAATGGTCGTCAACGACCCGTCTTGAAACACGGACCAAGGAGTCGAACAT
TTGTGCGAGTGTTTGGGTG--TCAAACCCTCACGCGTAATGAAAGTGAACGGAGGTGAGAGCCCTT--
--AC----GGGTGCATCATCGACCGATCCTGATG-
TCTTCGGATGGATTTGAGTAAGAGCATAACTGTTCGGACCCGAAAGATGGTGAACTATGCGTGGAT
AGGGTGAAGCCAGAGGAAACTCTGGTGGAGGCTCGCAGCGGTTCTGACGTGCAAATCGATCGTCA
AATCTGCGCATGGGGGCGAAAGACTTATCGA-
ACCATCTAGTAGCTGGTTACCGCCGAAGTTTCCCTCAGGATAGCAGTGT--TG-
TCTTCAGTTTTATGAGGTAAAGCGAATGATTAGGGACTCGGGGGCGCTATATTGCCTTCATCCATTC
TCAAACTTTAAATATGTAAGAAGCCCTTGTTACTTAATTGAACGTGGGCATTCGAATGTACCAACA
CTAGTGGGCCATTTTTGGTAAGCAGAACTGGCGATGCGGGATGAACCGAACGCGGGGTTAAGGTG
CCAGAGTGGACGCTCATCAGACACCACAAAAGGTGTTAGTACATCTTGACAGCAGGACGGTGGCC
ATGGAAGTCGGAATCCGCTAAGGACTGTGTAACAACTCACCTGCCGAATGTACTAGCCCTGAAAAT
GGATGGCGCTCAAGCGTCT-CACCCATACCTCGCCCTTAGGGTAGAAACGATGCCCTAAGG----------
----
--TTGACCGATACACTT------TCGCCTCGA---
CCCTCTCCCATCTAAGGCGGACGAACACACCTATCGGAAGAGACGGGAAGCTCGCAAAGCCTCGA
CAACTGCACAATACCCATTGGGGTCTGGTCTGTCCGGCCGAAACGCCCGAAGGCCAAGCCTGTGGG
CTGGTGAAGAATTTGTCGCTTATGTGCTCGATCAGTGTCGGTACCTCAACGGATCCTATCGTAGACT
ATATGATTACTAGGAATATGGAAGTCTTGGAGGAATACGAACCCATGAGATACCCTAACGCCACCA
AGATTTTCCTTAATGGATCTTGGATCGGTGTGCACCAGGATCCTAAGTCTCTGGTGAGAGATGTCCA
GCAGCTTCGTCGGGCCAACCAGATCCCCTACGAAGTGTCGCTGGTTCGCGATATCCGTGATCGCGA
GTTCAAGATCTTCTCGGATGCCGGCCGTGTTATGCGACCCTTATTTGTGGTGCAGCAAGAGGAC-----
----
AATCCCGAGGCTGAAACTATGAAGGGCTCCCTGGCTCTCAATAAAGAGATGATCCAGAGACTGGA
GGCGGATGTCGACCTGGACCCGGAAAGCGAAGAATATTTTGGTTGGCAAGGCCTGGTCAACGAGG
GAGTTATTGAGTACCTTGATGCGGAGGAAGAAGAAACTGCTATGATTTGCATGACACCCGAAGATT
TGGAAACCTATCGGCTGTCCAAGCTCGGATATGATGTGTCCCAGGACAACGGG------
GATGAGATTAACAAGCGACTAAAGACTAAGGTGAATCCCACGACGCACATGTATACGCATTGTGA
GATTCATCCTAGTATGCTCCTGGGTATCTGCGCGAGCATCATTCCTTTCCCAGACCACAACCAGGTA
CCCCTGAACGCGTCCCTCAAACCCCCTGAATTCTGCCCCTCATGCATAAACGCC---ACCACATCTA---
----CGTTATACATTGCAGTCACACCAAATTGTATAACAGTAGTCGGAAATATCAAAGCTAACCTTG---
-TTTTCTTCAATAGGTTCATCTCCAGACTGGCCAATGTGTAAGTAGC---------
ATCGATTATCGAAGAACCACGATATAAAAACACAGCGGGGCTCATACGAAGATCATAGGGTAACC
AAATCGGTGCCGCTTTCTGGCAAACCATCTCTAGCGAACACGGCCTCGACAGCAATGGCGTGTATG
TATTTAAGTCGTCAATTTCATCGCCAAGAATATCAACTAATCACCAATCAATAGCTACAACGGTAC
TTCCGAGCTTCAGCTCGAGCGCATGAGCGTCTACTTCAACGAGGTACGAATTTATGAGGCTTATGG
ATAAATAAACGGAGCCAATTACTAATTGCCTCAACGTGTGCAGGCTTCCGGCAACAAGTATGTTCC
TCGTGCCGTCCTCGTCGATCTCGAGCCCGGTACCATGGACGCCGTCCGTGCTGGTCCCTTCGGCCAG
CTCTTCCGTCCCGACAACTTTGTTTTTGGTCAGTCCGGTGCCGGAAACAACTGGGCCAAGGGTCATT
ACACTGAGGGTGCTGAGTTGGTTGACAACGTCCTCGATGTCGTTCGTCGTGAGGCTGAGGGCTGTG
ACTGCCTCCAGGGTTTCCAGATCACCCATTCCCTCGGTGGTGGTACTGGTGCCGGTATGGGTACCCT
GCTGATCTCCAAGATCCGCGAGGAATTCCCCGACCGCATGATGGCCACCTTCTCCGTCATGCCTTCC
CCTAAGGTCTCCGACACCGTCGTTGAGCCTTACAACGCCACCCTCTCCGTCCACCAGCTGGTCGAG
AACTCCGATGAGACCTTCTGTATCGACAACGAGGCTCTGTACGACATCTGCATGCGTACCCTTAAG
CTGTCCAACCCTTCCTACGGTGACCTGAACCACCTGGTCTCCGCTGTCATGTCTGGCGTTACCACTT
GCTTGCGTTTCCCCGGTCAGCTGAACTCTGACCTGCGCAAGCTCGCCGTGAACATGGTTCCTTTCCC
CCGTCTTCACTTCTTCATGGTCGGCTTCGCTCCCCTGACCAGCCGTGGCGCTCACTCCTTCCGTGCC
GTCACCGTTCCCGAGTTGACCCAGCAGATGTTCGACCCCAAGAACATGATGGCTGCTTCTGACTTC
CGTAACGGTCGTTACCTGACGTGCTCTGCCATCTTGTATGTTATCCCCCTCTAATGTGTATACGTTA
CTAGATTGCTAACTTGAACCGTCTAGCCGTGGCAAGGTCTCCA

>Ruwenzoria_pseudoannulata

-CATTACCGAGTTATCTAAAACTCCAACCCTATGTGAA-CTTACCGTCGTTGCCTCGGCG---
GGCGCGCTTGCCCGGTAGCTGCCCTG---------------------------GCGGCTACCCGGTGAGCGCGC-
GCAAGGCCCGCCGGTGGACCACTAAACTCTGTTATATATACTGTATCTCTGAATGCTTCAACTTAAT
AAGTTAAAACTTTCAACAACGGATCTCTTGGTTCTGGCATCGATGAAGAACGCAGCGAAATGCGAT
AAGTAATGTGAATTGCAGAATTCAGTGAATCATCGAATCTTTGAACGCACATTGCGCCCATTAGTA

TTCTAATGGGCATGCCTATTCGAGCGTCATTTCAACCCTTAAGCCCCTGTTGCTTAGTGTTGGGAAT
CTGCGTCTTGGGGCGCAGTTCCTCAAAGTAATCGGCGGAGTTGGGGCTTACTCTGAGCGTAGTAGT
CTTTCTCGCTTCTGTGGTAGCCCTGGCGGCCGGCCGTAAAACCCCTATTATCTTTAGTGGTTGACCT
CGAATTAGGTAGGAATACCCGCTGAACTTAAGCATA-------------GCTCAAA-TTTGAAATCTGGCC----
CTA-----GCGGTCCGAGTTGTAATTTGTAGAGGATGCTTTTGGCGAGGT-
GCCTTCCGAGTTCCCTGGAACGGGACGCCGGAGAGGGTGAGAGCCCCGTACGGTTGGA-
CGCCGAGCCTCTATATAGCTCCTTCGACGAGTCGAGTAGTTTGGGAATGCTGCTCTAAATGGGAGG
TAAATTTCTTCTAAAGCTAAATACCGGCCAGAGACC-
GATAGCGCACAAGTAGAGTGATCGAAAGATGAAAAGCACTTTGAAAAGAGGGTTAAATAGCACGT
GAAATTGTTGAAAGGGAAGCG-TTTGCGACCAGACTTTTTCCGGGCGGATCATCCGGGGTTT--
TCCCCGGTGCACTTCGCCCGG--TTTAGGCCAGCATCGGTTTC---
CTTAGGGGGATAAAGGCTTGGGGAACGTAGCTC----CCTCGGGAGTGTT-
ATAGCCCCTTGCGTAATACCCTTCG-GGGGACCGAGGAACGCGCT-TCG---
GCAAGGATGCTGGCGTAATGGTCGTCAACGACCCGTCTTGAAACACGGACCAAGGAGTCGAACAT
TTGTGCGAGTGTTTGGGTG--TCAAACCCTCACGCGTAATGAAAGTGAACGGAGGTGAGAGCCCTT--
--AC----GGGTGCATCATCGACCGATCCTGATG-
TCTTCGGATGGATTTGAGTAAGAGCATAACTGTTCGGACCCGAAAGATGGTGAACTATGCGTGGAT
AGGGTGAAGCCAGAGGAAACTCTGGTGGAGGCTCGCAGCGGTTCTGACGTGCAAATCGATCGTCA
AATCTGCGCATGGGGGCGAAAGACTTATCGA-
ACCATCTAGTAGCTGGTTACCGCCGAAGTTTCCCTCAGGATAGCAGTGT--TG-
TCTTCAGTTTTATGAGGTAAAGCGAATGATTAGGGACTCGGGGGCGCTATATTGCCTTCATCCATTC
TCAAACTTTAAATATGTAAGAAGCCCTTGTTACTTAATTGAACGTGGGCATTCGAATGTACCAACA
CTAGTGGGCCATTTTTGGTAAGCAGAACTGGCGATGCGGGATGAACCGAACGCGGGGTTAAGGTG
CCAGAGTGGACGCTCATCAGACACCACAAAAGGTGTTAGTACATCTTGACAGCAGGACGGTGGCC
ATGGAAGTCGGAATCCGCTAAGGACTGTGTAACAACTCACCTGCCGAATGTACTAGCCCTGAAAAT
GGATGGCGCTCAAGCGTCT-CACCCATACCTCGCCCTTAGGG---

CCAGAAGAAAGCGATGAGCTCCACGGCTGGTGTGTCACAGGTTTTGAACCGATACACTT------
TCGCCTCGA---
CCCTCTCCCATCTAAGGCGTACCAACACGCCTATCGGAAGAGATGGAAAGCTCGCAAAGCCTCGAC
AGCTGCACAACACACATTGGGGTTTGGTCTGTCCGGCCGAAACGCCCGAAGNCCAAGCCTGTGGGC
TGGTGAAGAACTTGTCGCTGATGTGCTCCATCAGTGTTGGTACCTCAACGGATCCTATCGTAGACTA
TATGATTACTAGGAATATGGAAGTCTTGGAGGAATACGAACCCATGAGGTACCCTAACGCCACCAA
GATCTTCCTTAACGGATCTTGGATCGGTGTGCACCAGGATCCCAAGTCTCTGGTGAGAGATGTCCA
GCAGCTTCGTCGGGCTAACCAGATCCCCTCGGAAGTGTCGCTGGTTCGCGATATCCGTGATCGTGA
GTTCAAGATCTTCTCGGATGCCGGTCGTGTTATGCGGCCCTTATTTGTGGTGCAGCAAGAGGAT------
----
AATATCGAGGCCGGCACTTCAAAGGGCACGCTTGCTCTTAATAAAGAGATGATCCAGAGGCTAGA
GGCTGATGTCGACTTGGATCCGGAGAGTGAGGAATATTTGGTTGGCAAGGCCTAGTCAACGAGGG
TGTTATTGAGTACCTCGATGCGGAGGAAGAAGAAACCGCTATGATTTGCATGACACCCGAAGATTT
GGAAACTTATCGGATGTCCAAACTCGGATATGATGTGTCCCAGGACAATGGA------
GACGAGATTAACAAGCGGCTTAAGACTAAGGTGAATCCCACAACGCACATGTATACGCATTGCGA
GATTCATCCTAGTATGCTCCTGGGTATCTGCGCGAGCATCATTCCCTTCCCAGACCACAACCAGGTA
---------GCGTCCCTCAAACCCCCTGAATTCTGCCCCTCATGCCTAAACGCC---ACCACATCTA-------
CATTGTATACTGCA------------------------GTCGGAAATATCAAAGCTAACCGCG----
TTCTTTTTAATAGGTTCATCTCCAGACTGGCCAATGTGTAAGTAAT---------
AGCGATGACGGAAGAACCACGATATAAAAACACAGCGGGGCTCATACGAAGTTGATAGGGTAACC
AAATCGGTGCCGCTTTCTGGCAAACCATCTCCAGCGAACACGGCCTCGACAGCAATGGCGTGTATG
TATTTCAATTGACAATTTAATTGTTAAGAATGTCAACTGACCACCAATCAATAGCTACAATGGTACT
TCCGAGCTTCAGCTTGAGCGCATGAGCGTCTACTTCAACGAGGTACGAATTAATAGAGCTTACGGA
TAAATAAATGGAACCAATTACTAATTGCCTCTACGTGTGTAGGCTACCGGCAACAAGTATGTTCCT
CGTGCCGTCCTCGTCGATCTCGAGCCCGGTACCATGGACGCCGTCCGTGCTGGTCCTTTCGGTCAGC
TCTTCCGACCCGACAACTTCGTCTTTGGTCAGTCCGGTGCTGGAAACAACTGGGCCAAGGGTCATT
ACACTGAGGGTGCTGAGTTGGTTGACAACGTTCTCGATGTCGTTCGTCGTGAGGCTGAGGGCTGTG
ACTGTCTCCAGGGTTTCCAAATCACCCACTCCCTCGGTGGTGGTACTGGTGCCGGTATGGGTACCTT
GTTGATCTCCAAGATCCGCGAAGAGTTCCCCGACCGCATGATGGCTACCTTCTCCGTCATGCCTTCC
CCCAAGGTCTCCGACACCGTCGTCGAGCCTTACAACGCTACCCTCTCCGTCCACCAGCTGGTCGAG

AACTCCGACGAGACCTTCTGTATCGACAACGAGGCTCTGTACGACATCTGTATGCGTACTCTTAAG
CTTTCCAACCCCTCCTACGGTGACCTGAACTACCTGGTCTCCGCCGTCATGTCTGGCGTTACCACTT
GCTTGCGTTTCCCCGGTCAACTGAACTCTGACCTGCGCAAGCTCGCCGTGAACATGGTTCCTTTTCC
TCGTCTGCACTTCTTCATGGTCGGCTTCGCTCCCCTGACCAGCCGTGGCGCCCACTCTTTCCGTGCT
GTCACCGTTCCCGAGTTGACTCAGCAGATGTTCGACCCCAAGAACATGATGGCTGCTTCTGACTTCC
GTAACGGCCGTTACCTGACGTGCTCTGCCATCTTGTATGTTATTCTTCTCTTTTTATTATAGATTATT
AGTTTGCTAACTTCAATCGTCTAGCCGTGGCAGGGTCTCCA

>Daldinia_bambusicola

-CATTACCGAGTTATCTAAACT-CCAACCCTATGTGAA-CCTACCGCCGTTGCCTCGGCG---
GGCGCGCTTAC---CTGCCCGGT-AGCGCGC-
TACGGGCCCGCCGGTGGACTGCTAAACTCTGTTATT-
TATAAGTATCTCTGAATGCTTCAACTTAATAAGTTAAAACTTTCAACAACGGATCTCTTGGTTCTGG
CATCGATGAAGAACGCAGCGAAATGCGATAAGTAATGTGAATTGCAGAATTCAGTGAATCATCGA
ATCTTTGAACGCACATTGCGCCCATTAGTATTCTAGTGGGCATGCCTGTTCGAGCGTCATTTCAACC
CTTAAG-
CCCTGCTGCTTAGTGTTGGGAGTCTAGGTCCCAGGGCCTAGTTCCTCAAAGTTATTGGCGGAGTTGG
GGTATACTCTGAGCGTAGTAGTTCTTCTCGCTTTTGCAGTAGCCCTGGCGGCTTGCCGTAAAACCCC
-TATATCT-
TAGTGGTTGACCTCGAATCAGGTAGGAATACCCGCTGAACTTAAGCATATGAAGCGGCAACAGCTC
AAA-TTTGAAATCTGGCC----CTA-----
GCGGTCCGAGTTGTAATTTGTAGAGGATGCTTTTGGTGAGGT-
GCCTTCCGAGTTCCCTGGAACGGGACGCCGGAGAGGGTGAGAGCCCCGTACGGTTGGA-
CGCCAAACCTCTGTATAGCTCCTTCGACGAGTCGAGTAGTTTGGGAATGCTGCTCTAAATGGGAGG
TAAATTTCTTCTAAAGCTAAATACCGGCCAGAGACC-
GATAGCGCACAAGTAGAGTGATCGAAAGATGAAAAGCACTTTGAAAAGAGGGTTAAATAGCACGT
GAAATTGTTGAAAGGGAAGCG-TTTGCGACCAGACCTTTTCCAGGCGGATCATCCGGCGTTC--
TCGCCGGTGCACTCCGCCTGG--TTGAGGCCAGCATCGGTTTC---
CTTAGGGGGATAAAGGCCTGGGGAACGTAGCTC----CTTCGGGAGTGTT-
ATAGCCCCTGGCGCAATACCCCTCG-GGGGACCGAGGACCGCGCT-TCG---
GCAAGGATGCTGGCGTAATGGTCGTCAACGACCCGTCTTGAAACACGGACCAAGGAGTCGAACAT
TTGTGCGAGTGTTTGGGTG--TCAAACCCTCACGCGTAATGAAAGTGAACGGAGGTGAGAGCCCTT--
--AC----GGGTGCATCATCGACCGATCCTGATG-
TCTTCGGATGGATTTGAGTAAGAGCATAACTGTTCGGACCCGAAAGATGGTGAACTATGCGTGGAT
AGGGTGAAGCCAGAGGAAACTCTGGTGGAGGCTCGCAGCGGTTCTGACGTGCAAATCGATCGTCA
AATCTGCGCATGGGGGCGAAAGACTTATCGA-
ACCATCTAGTAGCTGGTTACCGCCGAAGTTTCCCTCAGGATAGCAGTGT--TG-
TCTTCAGTTTTATGAGGTAAAGCGAATGATTAGGGACTCGGGGGCGCTATATTGCCTTCATCCATTC
TCAAACTTTAAATATGTAAGAAGCCCTTGTTACTTAGTTGAACGTGGGCATTCGAATGTACCAACA
CTAGTGGGCCATTTTTGGTAAGCAGAACTGGCGATGCGGGATGAACCGAACGCGGGGTTAAGGTG
CCAGAGTGGACGCTCATCAGACACCACAAAAGGTGTTAGTACATCTTGACAGCAGGACGGTGGCC
ATGGAAGTCGGAATCCGCTAAGGACTGTGTAACAACTCACCTGCCGAATGTACTAGCCCTGAAAAT
GGATGGCGCTCAAGCGTCTACACCCATACCTCGCCCTTAGGGTAGAAACGATGCCCTAAGG----------
----
---TTGACCGATATACTT------TCTCTTCAA---
CCCTCTCTCATTTGAGGCGAACGAACACGCCTATCGGAAGAGATGGAAAACTCGCGAAGCCTCGAC
AGCTTCACAACACCCACTGGGGTCTGGTCTGTCCGGCCGAAACGCCCGAAGGCCAGGCTTGCGGGC
TGGTAAAAAACCTATCGCTTATGTGCTCCATCAGCGTAGGTACGTCGACAGATCCCATCGTAGACT
ACATGATTACTAGGAATATGGAAGTCTTGGAGGAATATGAACCGATGCGGTACCCCAACGCCACC
AAGATCTTCCTAAACGGATCCTGGATCGGTGTTCATCAGGATCCTAAGTCTCTCGTGAGAGATGTG
CAGCAGCTTCGTCGGGCCAACCAGATCCCCTCCGAAGTCTCTCTGGTACGCGATATTCGTGATCGT
GAGTTCAAGATCTTTTCAGATGCCGGCCGAGTCATGCGGCCCTTATTCGTGGTGCAGCAAGAGGAT-
----

GATCCCGAGGCTGGTACCACCAAGGGCTCGCTGGCTCTCACTAAGGAGATGATCCAAAGATTGGA
GGCCAGCGTCGATCTAGACCAGGAGAGCGAGGAGTACTTTGGCTGGCAAGGTCTTGTTAACGAAG
GTGTTATCGAGTACCTCGACGCGGAAGAAGAAGAAACGGCCATGATTTGCATGACACCCGAAGAC
TTGGAAACTTACCGCATGTCTAAATTGGGATATGACGTATCTCAGGATAACGGC------

GACGAGATCAACAAGCGACTCAAGACCAAGTTGAATCCCACGACGCATATGTATACGCATTGCGA
GATCCATCCTAGTATGCTCCTAGGTATTTGCGCGAGCATCATTCCTTTCCCCGACCATAATCAGGTA
CCCCTGAACGCGTCTCTCAAACCCCCTGACTTCTGCCCCTCATGCGTAAACAAC---ACCACAATCT---
----
CATTTTACACAGCAACCATGTCAAATTATACGACAACATTGTAAAATATTCAAGCTAACCGCGTTT
C-----TTCAATAGGTTCACCTTCAGACTGGCCAATGTGTAAGTAAC---------AACCATCATCGAAACG-
AACTACGATAAAACATGGCGGGGCTCATACGAAGACGATAGGGTAACCAAATCGGTGCCGCTTTC
TGGCAAACCATCTCCAGCGAGCACGGTCTCGACAGCAATGGCGTGTATGTATTTGAATTGTCAATT
TCATCGTCAAGGATATCGACTAATCACCGATAAATAGCTACAACGGTACTTCCGAGCTTCAGCTCG
AGCGCATGAGCGTTTACTTCAACGAGGTACGAATTCACGCAAATCAAGGATAGGTAAATAGAATC
GGCTACTAATCACCTCTACGCGTGCAGGCTTCCGGCAACAAGTATGTTCCTCGTGCCGTCCTCGTCG
ATCTCGAGCCCGGTACCATGGATGCTGTCCGTGCTGGTCCCTTTGGTCAGCTCTTCCGACCCGACAA
CTTCGTTTTCGGTCAGTCCGGTGCTGGCAACAACTGGGCCAAGGGTCATTACACCGAGGGTGCTGA
GTTGGTTGACAACGTTCTCGACGTCGTTCGCCGTGAGGCTGAAGGCTGCGACTGCCTCCAAGGTTT
CCAGATCACCCACTCCCTCGGTGGTGGTACTGGTGCCGGTATGGGTACCCTATTGATCTCCAAGATC
CGCGAGGAGTTCCCCGACCGCATGATGGCTACTTTCTCCGTCATGCCCTCCCCTAAGGTCTCCGACA
CCGTCGTTGAGCCTTACAACGCCACCCTCTCTGTCCACCAGCTGGTCGAGAACTCCGACGAGACTTT
CTGTATCGACAACGAGGCTCTGTACGACATCTGCATGCGTACTCTGAAGCTGTCCAACCCTTCCTAC
GGTGACCTGAACCACCTCGTCTCCGCCGTCATGTCCGGTGTTACCACTTGCTTGCGTTTCCCCGGTC
AGCTAAACTCTGACCTGCGCAAGCTCGCCGTGAACATGGTTCCTTTCCCTCGTCTCCACTTCTTCAT
GGTCGGCTTCGCTCCCCTGACCAGCCGTGGCGCTCACTCCTTCCGTGCCGTCACCGTTCCCGAGTTG
ACTCAGCAGATGTTCGACCCCAAGAACATGATGGCTGCTTCTGACTTCCGAAACGGTCGTTACCTG
ACGTGCTCTGCCATCTTGTATGATATCCTCCCGTTTCTTTTATGAATTCCCATTTTGCTAACTCGAAT
CGTGTAGCCGTGGCAAGGTCTCCA

>Daldinia_theissenii

-CATTACTGAGTTATCTAAACT-CCAACCCTATGTGAA-CCTACCGCCGTTGCCTCGGCG---
GGCGCGTTTGCCCTGTAGCT------------------------------------TGCTACCTGGC-AGCGCGC-
TACAGGCCCGCCGGTGGACTGCTAAACTCTGTTATA-
CATAAGTATCTCTGAATGCTTCAACTTAATAAGTTAAAACTTTCAACAACGGATCTCTTGGTTCTGG
CATCGATGAAGAACGCAGCGAAATGCGATAAGTAATGTGAATTGCAGAATTCAGTGAATCATCGA
ATCTTTGAACGCACATTGCGCCCATTAGTATTCTAGTGGGCATGCCTGTTCGAGCGTCATTTCAACC
CTTAAGCCCCTGTTGCTTAGTGTTGGGAATCTAGGTCTCAGGGCCTAGTTCCTCAAAGTGATTGGCG
GAGTCGGAGTGTACTCTCAGCGTAGTAATCATTCTCGCTTTTGCAGTAGCCCCGGCGGCTTGCCGTA
AAACCCC-TATATTTTTAGTGGTTGACCTCGAATCAGGTAGGAATACCCGCTGAACTTAAGCAT-
TGAAGCGGCAACAGCTCAAA-TTTGAAATCTGGCC----CTA-----
GCGGTCCGAGTTGTAATTTGTAGAGGATGCTTTTGGTGAGGT-
GCCTTCCGAGTTCCCTGGAACGGGACGCCAGAGAGGGTGAGAGCCCCGTACGGTTGGA-
CGCCAAACCTCTGTATAGCTCCTTCGACGAGTCGAGTAGTTTGGGAATGCTGCTCTAAATGGGAGG
TAAATTTCTTCTAAAGCTAAATACCGGCCAGAGACC-
GATAGCGCACAAGTAGAGTGATCGAAAGATGAAAAGCACTTTGAAAAGAGGGTTAAATAGCACGT
GAAATTGTTGAAAGGGAAGCG-TTTGCGACCAGACTTTTTCCAGGCGGATCATCCGGCGTTC--
TCGCCGGTGCACTCCGCCTGG--TGGAGGCCAGCATCGGTTTC---
CTTAGGGGGATAAAGGCCTGGGGAACGTAGCTC----CTTCGGGAGTGTT-
ATAGCCCCTGGCGTAATACCCCTCG-GGGGACCGAGGAACGCGCT-CT----
GCAAGGATGCTGGCGTAATGGTCGTCAACGACCCGTCTTGAAACACGGACCAAGGAGTCGAACAT
TTGTGCGAGTGTTTGGGTG--TCAAACCCTCACGCGTAATGAAAGTGAACGGAGGTGAGAGCCCTT--
--AC----GGGTGCATCATCGACCGATCCTGATG-
TCTTCGGATGGATTTGAGTAAGAGCATAACTGTTCGGACCCGAAAGATGGTGAACTATGCGTGGAT
AGGGTGAAGCCAGAGGAAACTCTGGTGGAGGCTCGCAGCGGTTCTGACGTGCAAATCGATCGTCA
AATCTGCGCATGGGGGCGAAAGACTTATCGA-
ACCATCTAGTAGCTGGTTACCGCCGAAGTTTCCCTCACGATAGCAGTGT--TG-
TCTTCAGTTTTATGAGGTAAAGCGAATGATTAGGGACTCGGGGGCGCTATATTGCCTTCATCCATTC
TCAAACTTTAAATATGTAAGAAGCCCTTGTTACTTAGTTGAACGTGGGCATTCGAATGTACCAACA
CTAGTGGGCCATTTTTGGTAAGCAGAACTGGCGATGCGGGATGAACCGAACGCGGGGTTAAGGTG
CCAGAGTGGACGCTCATCAGACACCACAAAAGGTGTTAGTACATCTTGACAGCAGGACGGTGGCC
ATGGAAGTCGGAATCCGCTAAGGACTGTGTAACAACTCACCTGCCGAATGTACTAGCCCTGAAAT

GGATGGCGCTCAAGCGTCT-CACCCATACCTCGCCCTTAGGGTAGAAACGATGCCCTAAGG-----------
----
TCTTGACAGATATACTT------TTGCTTCGA---
CCCTTTCTCATTTGAGACGAACGAATACGCCTATCGGAAGAGACGGAAAGCTTGCAAAGCCTCGAC
AGCTGCACAATACCCATTGGGGTTTGGTCTGTCCGGCAGAAACGCCCGAAGGCCAGGCTTGCGGTT
TGGTGAAGAATCTGTCGCTTATGTGCTCGATAAGTGTGGGTACGTCAACGGACCCCATCGTCGACT
ATATGATTACGAGGAACATGGAAGTGTTGGAGGAATACGAACCCATGCGCTATCCTAACGCTACCA
AGATCTTCCTCAACGGATCTTGGATCGGTGTGCACCAAGATCCCAAGTCTCTCGTGAGAGATGTGC
AGCAGCTTCGCCGGGCCAACCAGATCCCCTCCGAAGTCTCTCTGGTTCGCGATATTCGTGATCGCG
AATTCAAGATTTTCTCCGATGCCGGTCGTGTCATGCGGCCCTTGTTCGTGGTGCAGCAAGAAGAT----
----
GATCCCGAGGCCGGTACCACGAAGGGCTCGCTGGCTCTTACCAAGGAGATGATTCAGAGGTTGGA
GGCGAGCGTCGATCTGGATCCGGAAAGCGAGGAGTACTTTGGTTGGCAAGGCCTAGTTAACGAAG
GTGTTATCGAGTACCTTGACGCGGAAGAAGAGGAAACGGCCATGATTTGCATGACACCCGAGGAT
TTGGAAACCTATCGGATGTCCAAACTCGGATATGACGTATCTCAGAATAACGGA------
GATGAGATTAACAAGCGTCTCAAGACGAAGTTGAATCCCACGACGCACATGTATACGCATTGCGA
GATTCATCCTAGTATGCTCCTGGGTATCTGCGCCAGCATCATTCCTTTCCCGGATCACAATCAGGTA
CCCCTGAACGCGTCCCTCAAACCCCCGGATTTTTGCCCCTCAGGCAAAAACCCCCCCCCCACA-------
-
CTACTTTTTACCCTTCACCCCCTTCAAATCGTAAAACAACAGTTGAAAATTTTCAAGCAAACCGGGT
TTT----TTTAAATGGGTCCCCCTTCAAACGGGCCAAGGTGAAATAAAC---------
AGCAATCTTCAAAAAG-GACCGCAATATTACTGGGGGGGGCTCAAACAAA-
AAAAAAGGGAAACAAAATCGGGGCCCTTTTTGGGCAAACCTTCCCCAGCGAGCCCGGTCTCAACA
GCAAGGGGGTGTTTTTTTTTGATTTGTCAATTCCTTGGCCAGGGATTCAAACGGCCCTTTAATCAAT
ACCTACAAGGGTATTTCCAAGTTTCAGTTCGAGGGCAGGAGCTTTTCCTTCAACGGGGTACAAATT
CCCGCGAATTAAGGATACCTAAATCAAATCAGTTACTAATTACCTCTGCGCGTGCAGGCTTCCGGC
AACAAGTATGTTCCTCGTGCCGTCCTCGTCGATCTCGAGCCCGGTACCATGGACCCCGTCCGTGCTG
GTCCCTTCGGTCAGCTCTTCCGACCCGACAACTTCGTTTTTGGTCAGTCCGGTGCTGGCAACAACTG
GGCCAAGGGTCATTACACTGAGGGTGCTGAGTTGGTTGACAACGTTCTCGATGTCGTTCGTCGTGA
GGCTGAAGGCTGTGACTGCCTCCAGGGTTTCCAGATCACCCACTCTCTCGGTGGTGGTACCGGTGC
CGGTATGGGTACTCTGTTGATCTCCAAGATCCGCGAGGAGTTCCCCGACCGTATGATGGCTACCTTC
TCCGTTATGCCCTCCCCCAAGGTCTCCGACACCGTCGTCGAGCCTTACAACGCCACCCTCTCCGTCC
ACCAGCTGGTCGAGAACTCCGATGAGACCTTCTGTATCGACAACGAGGCTCTGTACGACATCTGCA
TGCGTACTCTGAAGCTGTCCAACCCTTCTTACGGTGACCTGAACCACCTAGTTTCCGCAGTCATGTC
CGGTGTTACCACTTGCTTGCGTTTCCCCGGTCAGCTGAACTCTGACCTGCGCAAGCTGGCCGTGAAC
ATGGTTCCTTTCCCTCGTCTCCACTTCTTCATGGTCGGCTTCGCTCCCCTGACCAGCCGTGGCGCTCA
CTCTTTCCGTGCCGTCACCGTTCCTGAGTTGACTCAGCAGATGTTCGACCCCAAGAACATGATGGCT
GCTTCCGACTTCCGCAACGGTCGTTACCTGACGTGCTCTGCCATCTTGTATGATACTCCCCCAAAAT
T---ATGCATGACTATTTTGCTAACTTGAAATGTGTAGCCGTGGCAAGGTCTCCA

>Phylacia_globosa_STMA18042

----------------TAAACTCCAACCCTATGTGAACCTTACCGCTGTTGCCTCGGCG---
GGCGCGCTTACCCGGTAAC--CTACCCGGT-AGCGCGC-
TATAGGCCCGCCAGTGGACCATCAAACTCTGATATT-
ATGGCGTAATTCTGAATGCTTCAACTTAATAAGTTAAAACTTTCAACAACGGATCTCTTGGTTCTGG
CATCGATGAAGAACGCAGCGAAATGCGATAAGTAATGTGAATTGCAGAATTCAGTGAATCATCGA
ATCTTTGAACGCACATTGCGCCCATTAGTATTCTAGTGGGCATGCCTGTTCGAGCGTCATTACGACC
CTTAAG-
CCCTGTGGCTTAGTGTTGGGAGTCTAGGTCCTGGGGCCCAGTTCCTCAAAGCTAGTGGCGGAGTTA
GGGTATACTCTGAGCGTAGTAGTTCTTCTCGCTTCTGTAGTAGCCCTGGCGGCCTGCCGTAAAAGCC
CCCATTTCTTTAGTGGTTGACCTCGAATCAGGTAGGAATACCCGCTGAACTTAAGCATATGAAGCG
GCAACAGCTCAAA-TTTGAAATCTGGCC----CTA-----
GCGGTCCGAGTTGTAATTTGTAGAGGATGCTTCTGGTTAGGT-
GCCTTCCGAGTTCCCTGGAACGGGACGCCGGAGAGGGTGAGAGCCCCGTACGGTTGGA-
CGCCGAGCCTCTGTGTAGCTCCTTCGACGAGTCGAGTAGTTTGGGAATGCTGCTCTAAATGGGAGG
TAAATTTCTTCTAAAGCTAAATACCGGCCAGAGACC-
GATAGCGCACAAGTAGAGTGATCGAAAGATGAAAAGCACTTTGAAAAGAGGGTTAAATAGCACGT

GAAATTGTTGAAAGGGAAGCG-TTTGCGACCAGACTTTCTCTAGGGGGATCATCCGGCGTTC--
TCGCCGGTGCACTTCCCCTAG--TAGAGGCCAGCATCGGTTTC---
CTTAGGGGGATAAAGGCCTAGGGAACGTAGCTC----CTTCGGGAGTGTT-
ATAGCCCCTGGCGTAATACCCCTCG-GGGGACCGAGGAACGCGCT-CT----
GCAAGGATGCTGGCGTAATGGTCGTCAACGACCCGTCTTGAAACACGGACCAAGGAGTCGAACAT
TTGTGCGAGTGTTTGGGTG--TCAAACCCTCACGCGTAATGAAAGTGAACGGAGGTGAGAGCCCTT--
--AC----GGGTGCATCATCGACCGATCCTGATG-
TCTTCGGATGGATTTGAGTAAGAGCATAACTGTTCGGACCCGAAAGATGGTGAACTATGCGTGGAT
AGGGTGAAGCCAGAGGAAACTCTGGTGGAGGCTCGCAGCGGTTCTGACGTGCAAATCGATCGTCA
AATCTGCGCATGGGGGCGAAAGACTTATCGA-
ACCATCTAGTAGCTGGTTACCGCCGAAGTTTCCCTCAGGATAGCAGTGT--TG-
TCTTCAGTTTTATGAGGTAAAGCGAATGATTAGGGACTCGGGGGCGCTATATTGCCTTCATCCATTC
TCAAACTTTAAATATGTAAGAAGCCCTTGTTACTTAGTTGAACGTGGGCATTCGAATGTACCAACA
CTAGTGGGCCATTTTTGGTAAGCAGAACTGGCGATGCGGGATGAACCGAACGCGGGGTTAAGGTG
CCAGAGTGGACGCTCATCAGACACCACAAAAGGTGTTAGTACATCTTGACAGCAGGACGGTGGCC
ATGGAAGTCGGAATCCGCTAAGGACTGTGTAACAACTCACCTGCCGAATGTACTAGCCCTGAAAAT
GGATGGCGCTCAAGCGTCT-CACCCATACCTCGCCCTTAGGGTAGAAACGATGCCCTAAGG-----------
----
TTCCAGATCGAGCTCGCCGCTAAACCCGCAATAGTCAGCAACGGTTTGAAATATTCTCTCGCCACA
GGAAACTGGGGTGATCAGAAGAAGGCGATGAGCTCGACGGCCGGTGTCTCGCAGGTCTTGAACCG
ATATACTT------TCTCGTCGA---
CCCTTTCCCATCTGAGGCGAACCAACACGCCCATCGGGAGAGACGGGAAGCTCGCGAAGCCCCGA
CAGCTGCACAATACCCATTGGGGTCTGGTCTGTCCGGCCGAAACGCCCGAAGGCCAGGCTTGCGGT
CTGGTGAAGAACCTATCGCTCATGTGTTCCATCAGCGTGGGTACCTCGACGGATCCCATCGTAGAC
TACATGATTACTAGGAATATGGAAGTCTTGGAGGAATACGAGCCGATGCGATACCCTAATGCCACC
AAGATCTTTCTTAACGGGTCATGGATCGGTGTGCATCAGGATCCCAAGTCGCTCGTCAGAGATGTC
CAGCAGCTTCGTCGGGCTAACCAAATCCCCTCCGAAGTGTCTCTCGTTCGTGATATCCGCGATCGCG
AGTTCAAGATCTTTTCGGACGCCGGTCGTGTCATGCGGCCCTTGTTCGTGGTGCAGCAAGAGGAT---
----
GTTCCCGAGGCCGGTATCACGAAAGGCTCGCTGGCCCTGACCAAAGAGATGATCCAGAGGTTGGA
GGCGAGTGTCGACGTCGACCCGGAGAGCGAAGAGTACTATGGCTGGCAAAGTCTTGTTAACGAGG
GTGTTATCGAGTACCTCGACGCGGAGGAGGAAGAAACGGCGATGATCTGCATGACCCCCGAAGAT
TTGGAAACCTACCGGATGTCCAAGCTCGGATACGACGTGTCTCAGGACAACGGG------
GATGAGATCAACAAGCGGCTCAAGACTAAGCTGAATCCCACGACGCACATGTACACGCATTGCGA
GATCCACCCCAGCATGCTCCTGGGTATCTGCGCGAGCATCATCCCCTTCCCCGACCACAACCAGGT
A---------------
CTCAAACCCCCTGAGTTCTGCCCCTCATGCATAAACGCCTCAACCACAACATCTCACAACATCTTAC
ACTGCAACCACGTCGAATCGTATTTCAGCAGTTGCAAATAGTCAAGCTAACCACGTTTTTTCTTTTC
GATAGGTTCACCTTCAAACCGGCCAGTGTGTAAGTAGC---------AACGATGATGGAAAGG--
ACCGCGATAGCGAATAGCGGGGCTCATACGAAGATGATAGGGTAACCAAATCGGTGCTGCTTTCTG
GCAAACCATCTCCAGCGAGCACGGCCTCGACAGCAATGGCGTGTACGTATTTGAACCGTCGATTCC
ATCGCCAAGAATGTCCACTGACCAACGATCGATAGCTATAACGGTACTTCGGAGCTCCAGCTCGAG
CGCATGAGCGTCTACTTCAACGAGGTACGAGTTTGCAGATGCCAAGAATAAGTTTACAGAGTCAGC
TATTAATCGCTTCTACGCGTACAGGCTTCCGGCAACAAGTATGTTCCCCGTGCCGTCCTCGTCGATC
TCGAGCCCGGTACCATGGACGCTGTCCGTGCTGGTCCCTTTGGCCAGCTCTTCCGACCCGACAACTT
CGTCTTCGGCCAGTCCGGTGCCGGAAACAACTGGGCCAAGGGTCACTATACCGAGGGTGCTGAGTT
GGTCGACAACGTTCTCGACGTCGTTCGTCGTGAGGCCGAGGGCTGCGACTGCCTCCAGGGTTTCCA
GATCACCCACTCCCTCGGCGGTGGTACTGGTGCCGGTATGGGTACCCTGCTGATCTCCAAGATCCG
CGAGGAGTTCCCCGACCGTATGATGGCTACCTTCTCCGTCATGCCCTCCCCCAAGGTCTCCGACACT
GTCGTCGAGCCTTACAACGCCACCCTCTCCGTCCACCAGCTGGTCGAGAACTCGGACGAGACCTTC
TGTATCGACAACGAGGCTCTGTACGACATCTGCATGCGTACCCTCAAGCTGTCCAACCCCTCGTAC
GGCGACCTGAACCACCTGGTCTCCGCCGTCATGTCGGGCGTTACCACTTGCTTGCGTTTCCCCGGTC
AGCTCAACTCGGACCTGCGCAAGCTTGCCGTGAACATGGTTCCTTTCCCCCGTCTCCACTTCTTCAT
GGTCGGCTTTGCTCCCCTGACCAGCCGTGGCGCTCACTCGTTCCGTGCCGTCACCGTTCCCGAGTTG
ACCCAGCAGATGTTCGACCCCAAGAACATGATGGCTGCTTCTGACTTCCGCAACGGTCGTTACCTG
ACGTGTTCTGCCATCTTGTATGATATCCCCCCCATACCATTATGCGTTACTATCTTGCTAACTTTGGT
TGTGT-------------------

>Phylacia_lobulata_STMA18032

-----------TTATAAAAAACTCCAACCCTATGTGAA-CCTACCGCCGTTGCCTCGGCG---
GGCGCGCTTACCCGGTAGCTACCCTGTAG-------------------------GTAGCTACCCGGT-AGCGCGC-
TACAGGCCCGCCGGTGGACCAGCAAACTCTGATATT-
ATTACGTAATTCTGAATGCTTCAACTTAATAAGTTAAAACTTTCAACAACGGATCTCTTGGTTCTGG
CATCGATGAAGAACGCAGCGAAATGCGATAAGTAATGTGAATTGCAGAATTCAGTGAATCATCGA
ATCTTTGAACGCACATTGCGCCCATTAGTATTCTAGTGGGCATGCCTGTTCGAGCGTCATTACGACC
CTTAAGCCCCTGTAGCTTAGTGTTGGGAGTCTAGGTCCCGCGGCCTAGTTCCTCAAAGTTAGTGGCG
GAGTTAGGGTATACTCTAAGCGTAGTAGTTCTTCTCGCTTCTGTAGTAGCCCTGGCGGCCTGCCGTA
AAAGCCCCTATATCTTTAGTGGTTGACCTCGAATCAGGTAGGAATACCCGCTGAACTTAAGCATAT
GAAGCGGCAACAGCTCAAA-TTTGAAATCTGGCC----CTA-----
GCGGTCCGAGTTGTAATTTGTAGAGGATGCTTTTGGTTAGGT-
GCCTTCCGAGTTCCCTGGAACGGGACGCCGGAGAGGGTGAGAGCCCCGTACGGTTGGA-
CGCCGAGCCTTTGTATAGCTCCTTCGACGAGTCGAGTAGTTTGGGAATGCTGCTCTAAATGGGAGG
TAAATTTCTTCTAAAGCTAAATACCGGCCAGAGACC-
GATAGCGCACAAGTAGAGTGATCGAAAGATGAAAAGCACTTTGAAAAGAGGGTTAAACAGCACGT
GAAATTGTTGAAAGGGAAGCG-TTTGCGACCAGACTTTTTCTAGGGGGATCATCCGGCGTTC--
TCGCCGGTGCACTTCCCCTAG--TAGAGGCCAGCATCGGTTTC---
CTTAGGGGGATAAAGGCCTGGGGAACGTAGCTC----CTTCGGGAGTGTT-
ATAGCCCCTGGCGTAATACCCCTCG-GGGGACCGAGGAACGCGCT-CT----
GCAAGGATGCTGGCGTAATGGTCGTCAACGACCCGTCTTGAAACACGGACCAAGGAGTCGAACAT
TTGTGCGAGTGTTTGGGTG--TCAAACCCTCACGCGTAATGAAAGTGAACGGAGGTGAGAGCCCTC--
--AC----GGGTGCATCATCGACCGATCCTGATG-
TCTTCGGATGGATTTGAGTAAGAGCATAACTGTTCGGACCCGAAAGATGGTGAACTATGCGTGGAT
AGGGTGAAGCCAGAGGAAACTCTGGTGGAGGCTCGCAGCGGTTCTGACGTGCAAATCGATCGTCA
AATCTGCGCATGGGGGCGAAAGACTTATCGA-
ACCATCTAGTAGCTGGTTACCGCCGAAGTTTCCCTCAGGATAGCAGTGT--TG-
TCTTCAGTTTTATGAGGTAAAGCGAATGATTAGGGACTCGGGGGCGCTATATTGCCTTCATCCATTC
TCAAACTTTAAATATGTAAGAAGCCCTTGTTACTTAGTTGAACGTGGGCATTCGAATGTACCAACA
CTAGTGGGCCATTTTTGGTAAGCAGAACTGGCGATGCGGGATGAACCGAACGCGGGGTTAAGGTG
CCAGAGTGGACGCTCATCAGACACCACAAAAGGTGTTAGTACATCTTGACAGCAGGACGGTGGCC
ATGGAAGTCGGAATCCGCTAAGGACTGTGTAACAACTCACCTGCCGAATGTACTAGCCCTGAAAAT
GGATGGCGCTCAAGCGTCT-CACCCATACCTCGCCCTTAGGGT------------------------------
CAGAACCGGCGCTTCCAGATCGAGCTCGCCGCCAAACCCGCCATAGTCAGTAACGGTTTGAAATAT
TCTCTCGCCACAGGAAACTGGGGCGATCAGAAGAAGGCGATGAGTTCGACGGCTGGCGTCTCGCA
GGTCTTGAACCGATATACTT------TCTCGTCGA---
CCCTTTCCCATCTGAGGCGAACCAACACGCCTATCGGGAGAGACGGGAAGCTCGCGAAGCCTCGA
CAGCTGCACAATACCCATTGGGGTCTGGTCTGTCCGGCCGAAACGCCCGAAGGCCAGGCTTGCGGT
TTGGTGAAGAATCTATCGCTCATGTGCTCCATCAGCGTGGGTACCTCGACGGATCCTATCGTAGACT
ACATGATAACTAGGAATATGGAAGTCTTGGAGGAATACGAGCCGATGCGATACCCTAATGCCACC
AAGATCTTTCTTAACGGGTCATGGATCGGTGTGCATCAAGATCCCAAGTCGCTCGTCAGAGATGTC
CAGCAGCTTCGTCGGGCCAACCAAATCCCCTCCGAAGTGTCGCTCGTTCGTGATATCCGTGATCGC
GAGTTCAAGATCTTTTCGGACGCCGGTCGTGTCATGCGGCCCTTGTTCGTGGTGCAGCAAGAGGAC-
----
GATCCCGAGTCCGGTATCACGAAAGGCTCGCTGGCCCTGACAAAAGAGATGATCCAGAGGTTGGA
GGCGAGTGTCGATGTCGACCCGGAGAGCGAAGAATACTATGGCTGGCAGAGTCTCGTTAACGAGG
GTGTTATCGAGTACCTCGACGCGGAGGAGGAAGAAACGGCCATGATCTGCATGACTCCCGAAGAC
TTGGAAACCTACCGGATGTCGAAGCTCGGATATGACGTGTCTCAGGACAACGGG------
GATGAGATCAACAAGCGGCTCAAGACCAAGTTAAATCCCACGACGCACATGTACACGCATTGCGA
GATCCATCCCAGCATGCTCCTGGGTATCTGCGCGAGCATCATCCCCTTCCCCGACCACAATCAGGT
A--

>Phylacia_surinamensis_STMA18044

---TTAGCGAGTTATAGAAACTCCCAACCCTATGTGAA-CTTACCGCCGTTGCCTCGGCG---
GGCGCGCTTACCTGGTAGCTAG---------------------------------CTAGCTACCCGGT-AGCGCAC-
TACAGGCCCGCCGGTGGACTGCTAAACTCTGATATT-
TATATGTCATTCTGAATGCTTCAACTTAATAAGTTAAAACTTTCAACAACGGATCTCTTGGTTCTGG
CATCGATGAAGAACGCAGCGAAATGCGATAAGTAATGTGAATTGCAGAATTCAGTGAATCATCGA
ATCTTTGAACGCACATTGCGCCCATTAGTATTCTAGTGGGCATGCCTGTTCGAGCGTCATTACGACC
CTTAAG-
CCCTGTAGCTTAGTGTTGGGAGTCTAGGTCCTGGGGCCTAGTTCCTCAAAGTTAGTGGCGGAGTTA
GGGTATACTCTAAGCGTAGTAGTTCTTCTCGCTTCTGTAGTAGCCCTGGCGGCCTGCCGTAAAACCC
CCCTATTCTTTAGTGGTTGACCTCGAATCAGGTAGGAATACCCGCTGAACTTAAGCATATGAAGCG
GCAACAGCTCAAA-TTTGAAATCTGGCC----CTA-----
GTGGTCCGAGTTGTAATTTGTAGAGGATGCTTTTGGTTAGGT-
GCCTTCCGAGTTCCCTGGAACGGGACGCCGGAGAGGGTGAGAGCCCCGTACGGTTGGA-
CGCCGAGCCTCTGTATAGCTCCTTCGACGAGTCGAGTAGTTTGGGAATGCTGCTCTAAATGGGAGG
TAAATTTCTTCTAAAGCTAAATACCGGCCAGAGACC-
GATAGCGCACAAGTAGAGTGATCGAAAGATGAAAAGCACTTTGAAAAGAGGGTTAAATAGCACGT
GAAATTGTTGAAAGGGAAGCG-TTTGCGACCAGACTTTTTCTAGGGGGATCATCCGGCGTTC--
TCGCCGGTGCACTTCCCCTAG--TAGAGGCCAGCATCGGTTTC---
CTTAGGGGGATAAAGGCCTTGGGAACGTAGCTC----TTTCGGGAGTGTT-
ATAGCCCTTGGCGTAATACCCCTCG-GGGGACCGAGGAACGCGCT-CT----
GCAAGGATGCTGGCGTAATGGTCGTCAACGACCCGTCTTGAAACACGGACCAAGGAGTCGAACAT
TTGTGCGAGTGTTTGGGTG--TCAAACCCTCACGCGTAATGAAAGTGAACGGAGGTGAGAGCCCTT--
--AC----GGGTGCATCATCGACCGATCCTGATG-
TCTTCGGATGGATTTGAGTAAGAGCATAACTGTTCGGACCCGAAAGATGGTGAACTATGCGTGGAT
AGGGTGAAGCCAGAGGAAACTCTGGTGGAGGCTCGCAGCGGTTCTGACGTGCAAATCGATCGTCA
AATCTGCGCATGGGGGCGAAAGACTTATCGA-
ACCATCTAGTAGCTGGTTACCGCCGAAGTTTCCCTCAGGATAGCAGTGT--TG-
TCTTCAGTTTTATGAGGTAAAGCGAATGATTAGGGACTCGGGGGCGCTATATTGCCTTCATCCATTC
TCAAACTTTAAATATGTAAGAAGCCCTTGTTACTTAATTGAACGTGGGCATTCGAATGTACCAACA
CTAGTGGGCCATTTTTGGTAAGCAGAACTGGCGATGCGGGATGAACCGAACGCGGGGTTAAGGTG
CCAGAGTGGACGCTCATCAGACACCACAAAAGGTGTTAGTACATCTTGACAGCAGGACGGTGGCC
ATGGAAGTCGGAATCCGCTAAGGACTGTGTAACAACTCACCTGCCGAATGTACTAGCCCTGAAAAT
GGATGGCGCTCAAGCGTCT-
CACCCATACCTCGCCCTTAGGGTAGAAACGATGCCCTAAGGACGTTGTATTGACCAGAACAGGCGT
TTCCAGATCGAGCTCGCCGCCAAACCCGCCATAGTCAGTAACGGTTTGAAATATTCTCTGGCCACA
GGAAACTGGGGTGATCAGAAGAAGGCGATGAGCTCGACGGCTGGTGTCTCGCAGGTTTTGAACCG
ATATACTT------TCTCGTCGA---
CTCTTTCCCATCTGAGGCGAACCAACACGCCTATCGGAAGAGACGGGAAGCTCGCGAAGCCTCGAC
AGCTGCACAATACCCATTGGGGTCTGGTCTGTCCGGCCGAAACGCCCGAAGGCCAGGCTTGCGGTT
TGGTGAAGAATCTATCGCTCATGTGCTCCATCAGCGTGGGTACCTCGACGGATCCTATCGTAGACT
ACATGATAACTAGGAATATGGAAGTCTTGGAGGAGTACGAGCCGATGCGATACCCTAATGCCACC
AAGATCTTTCTTAACGGGTCATGGATCGGTGTGCATCAGGATCCCAAGTCGCTCGTCAGAGATGTC
CAGCAGCTTCGTCGGGCTAACCAAATCCCCTCGGAAGTGTCTCTCATCCGTGATATCCGTGATCGC
GAGTTCAAGATCTTTTCGGACGCCGGTCGTGTCATGCGGCCCTTGTTCGTGGTGCAGCAAGAGGAT-
----

GATCCCGAGTCCGGTATTACGAAGGGCTCGCTGGCCCTGACCAAAGAGATGATCCAGAGGTTGGA
GGCGAGTGTCGATGTCGACCCGGAGAGCGAAGAGTACTATGGTTGGCAGAGTCTTGTTAACGAGG
GTGTTATCGAGTACCTCGACGCGGAGGAGGAAGAAACGGCCATGATCTGCATGACCCCCGAAGAT
TTGGAAACTTACCGGATGTCCAAGCTCGGGTATGATGTATCTCAGGACAACGGG------
GATGAGATTAACAAGCGGCTCAAGACTAAGTTGAATCCCACGACGCACATGTACACGCATTGCGA

GATCCATCCCAGCATGCTCTTGGGTATCTGCGCGAGCATCATCCCCTTCCCCGATCACAATCAGGTA

>Hypomontagnella_barbarensis

-CATTACTGAGTTATCAAAACTCCCAACCCTTTGTGAA-CTTACCACTGTTGCCTCGGCG---
AGCGTGC-TACCCTATA------------------------------GCTACCCTGTAGCTACCCGGG-AGCACGC-
TATAAGCTCGCCAGAGGACCACTAAACTCTGTTTT--
ATTCTGTATCTCTGAATCGTATAACTAAATCAGTTAAAACTTTCAACAACGGATCTCTTGGTTCTGG
CATCGATGAAGAACGCAGCGAAATGCGATAAGTAATGTGAATTGCAGAATTCAGTGAATCATCGA
ATCTTTGAACGCACATTGCGCCCATTAGTATTCTAGTGGGCATGCCTATTCGAGCGTCATTTCAACC
CTTAAGCCTCGGTTGCTTAGCGTTGGGATTCTACGGGTTACAGCGTAGTTCCCCAAAAGTAGTGGC
GGAGTTGGTGCTCACTCTCAGCGTAGTAATTCTTCTCGCTTTTGTAGTGGCGCTGGCGGCCGGCCGT
AAAACCCCCTATATTTCTAGTGGTTGACCTCGGATTAGGTAGGAATACCCGCTGAACTTAAGCATA
TGAAGCGGCAACAGCTCAAA-TTTGAAATCTGGCC----TTC-----G-
GGTCCGAGTTGTAATTTGCAGAGGATGCTTTGGGTGCGGT-
GCCTTCCGAGTTCCCTGGAACGGGACGCCTTAGAGGGTGAGAGCCCCGTACGGTTGGA-
CACCTAGCCTATGTATAGCTCCTTCGACGAGTCGAGTAGTTTGGGAATGCTGCTCTAAATGGGAGG
TAAATTTCTTCTAAAGCTAAATACCGGCCAGAGACC-
GATAGCGCACAAGTAGAGTGATCGAAAGATGAAAAGCACTTTGAAAAGAGGGTTAAATAGCACGT
GAAATTGTTGAAAGGGAAGCG-TTTGCGACCAGACCTTTTCCCGGCGGATCATCCGGTGTTC--
TCACCGGTGCACTTCGCCGGG--TTTAGGCCAGCATCGGTTCT---
CTTCGGGGGATAAAGGCTCGGGGCACGTAGCTC----TTTCGGGAGTGTT-
ATAGCCCCTTGCGTAATACCCTGCG-GGGGACCGAGGACCGCGCA-TT----
GCAAGGATGCTGGCGTAATGGTCGTCAACGACCCGTCTTGAAACACGGACCAAGGAGTCGAACAT
TTGTGCGAGTGTTTGGGTG--TCAAACCCTCACGCGTAATGAAAGTGAACGGAGGTGAGAGCCCTT--
---CG---GGGCGCATCATCGACCGATCCTGATG-
TCTTCGGATGGATTTGAGTAAGAGCATAACTGTTCGGACCCGAAAGATGGTGAACTATGCGTGGAT
AGGGTGAAGCCAGAGGAAACTCTGGTGGAGGCTCGCAGCGGTTCTGACGTGCAAATCGATCGTCA
AATCTGCGCATGGGGGCGAAAGACTTATCGA-
ACCATCTAGTAGCTGGTTACCGCCGAAGTTTCCCTCAGGATAGCAGTGT--TG-
TCTTCAGTTTTATGAGGTAAAGCGAATGATTAGGGACTCGGGGGCGCTATATTGCCTTCATCCATTC
TCAAACTTTAAATATGTAAGAAGCCCTTGTTACTTAATTGAACGTGGGCATTCGAATGTACCAACA
CTAGTGGGCCATTTTTGGTAAGCAGAACTGGCGATGCGGGATGAACCGAACGTGGGGTTAAGGTG
CCAGAGTGGACGCTCATCAGACACCACAAAAGGTGTTAGTACATCTTGACAGCAGGACGGTGGCC
ATGGAAGTCGGAATCCGCTAAGGACTGTGTAACAACTCACCTGCCGAATGTACTAGCCCTGAAAAT
GGATGGCGCTCAAGCGTCT-
CACCCATACCTCACCNNNNGGGTAGAAACGATGCCCTAAGGGCGCTGCATCGACCAGAACAAACG
TTTCCAGATCGAGCTGGCCGCTAAGCCCGCTATTATCACTAACGGACTCAAGTACTCGCTCGCTACT
GGCAACTGGGGTGACCAGAAGAAGGCCATGAGCTCGACCGCCGGTGTGTCGCAGGTCTTGAATCG
ATACACGT------TTGCCTCGA---
CCTTATCTCACTTAAGGCGAACGAATACCCCCATCGGAAGAGACGGGAAGCTCGCGAAGCCCCGA
CAGCTTCACAACACCCATTGGGGTCTGGTCTGCCCGGCCGAGACGCCCGAAGGCCAAGCTTGTGGT
TTGGTGAAGAACTTATCCTTGATGTGCTCGATCAGCGTGGGTACATCAACGGATCCTATCGTCGACT
ACATGATCACTAGGAACATGGAAGTGCTGGAGGAGTACGAACCTATGCGATACCCCAACGCCACC
AAGATCTTTTTGAACGGTTCTTGGATCGGGGTGCACCAGGACCCCAAGAACCTCGTCCGAGATGTC
CAACACCTGCGCCGAACCAATCAGATCCCCGCCGAGGTATCGTTGGTCCGCGACATTCGTGACCGT
GAGTTCAAGATCTTCTCGGATGCCGGTCGCGTCATGCGCCCCTTGTTTGTCGTACAGCAAGAAGAT-

----
GACGAGGCCAACGGTATCAACAAGGGCTCGTTAGCACTTAGCAAGAGCATGATCCAACGACTAGA
AGCGGATGCCGACGTCGACCCCAAGAGTGACGAGTACTTCGGGTGGCAAGGACTAGTAGACGAGG
GAGCCATTGAATTTCTCGATGCCGAGGAAGAAGAGACAGCCATGATATGCATGACACCCGAGGAT
CTAGAGATCTATCGCCAGAGCAAGGCCGGAATTGAAGTGTCTCAGGACAACGGC------
GACGAAATTAACAAACGACTCAAGACCAAGTTGAACCCGACGACGCATATGTACACGCATTGTGA
GATCCATCCCAGTATGCTCCTGGGTATCTGCGCGAGCATCATTCCCTTCCCGGATCACAATCAGGTA
------------------CAACCCCTTGATTTCTGCCCCTCACGCCCACAAATC---
ACCACAATAACCCAGCACTCTCTACGCTATGCTATTGCGAACTTGCACAACTTCGTGATGGAATTTT
GAAACTAACTGTGTCTTTTCATGTCGATAGGTTCACCTTCAGACCGGCCAGTGCGTAAGTACTATC--
----
TACGATTCCCGACGAAACATGGCGCGAGGACATAGTGGGGCTCACATAAATATTATAGGGTAACC
AAATTGGTGCTGCTTTCTGGCAAACTATCTCTGGCGAGCATGGTCTCGACAGCGATGGTGTGTACG
TATAGCAATGGTCAATTCGAGGCTTCGGAATATGAACT---
GACCAATCAACAGCTACAACGGTACCTCTGAGCTCCAGCTTGAGCGCATGAGCGTCTACTTCAACG
AGGTATGTAGCTTCAGAAATCCAACATGGATACACAAGATCGGCTGCTAATCACCCTGATATCTAC
AGGGTTCTGGCAACAAGTATGTTCCTCGCGCTGTTCTCGTCGATCTCGAGCCCGGCACCATGGATG
CTGTCCGTGCTGGTCCTTTCGGCCAGCTTTTCCGACCTGACAACTTCGTCTTTGGCCAGTCCGGTGC
CGGCAACAACTGGGCCAAGGGTCACTACACTGAGGGTGCTGAGCTTGTTGACCAGGTCCTCGATGT
CGTTCGTCGTGAGGCTGAAGGCTGTGATTGCCTTCAGGGTTTCCAGATCACCCACTCGCTCGGTGGT
GGTACCGGTGCCGGTATGGGTACTCTGTTGATCTCCAAGATCCGTGAAGAGTTCCCCGACCGAATG
ATGGCCACCTTCTCCGTCGTTCCCTCCCCCAAGGTCTCCGATACCGTTGTCGAGCCTTACAACGCCA
CCCTCTCCGTCCACCAGCTGGTCGAGAACTCTGACGAGACTTTCTGTATCGACAACGAGGCGCTGT
ACGACATCTGCATGCGTACCCTGAAGCTGTCCAACCCCTCTTACGGTGACCTGAACCACCTCGTCTC
CGCCGTCATGTCCGGTGTCACCACCTGCCTGCGTTTCCCTGGTCAGCTGAACTCTGACCTGCGCAAG
CTCGCCGTGAACATGGTTCCTTTCCCTCGTCTCCACTTCTTCATGGTTGGATTCGCTCCTCTGACCAG
CCGCGGCGCGTACTCTTTCCGTGCCGTCACCGTCCCCGAGTTGACCCAGCAGATGTTCGACCCCAA
GAACATGATGGCTGCTTCCGACTTCCGCAATGGTCGCTACCTGACGTGCTCTGCCATCTTGTAGGAT
ACCATCCCTGAATCTTTTTTTCCTGCTAATGTGCTAACTTGAA------------------------

>Hypomontagnella_submonticulosa

-CATTACTGAGTTATAAAAACTCCCAACCCTATGTGAA-CTTACCACTGTTGCCTCGGCG---
AGCGCGC-TACCCTGTA----------------------------GCTACTTTGTAGCGACCTGGG-AGCGCGT-
TATAGGCTCGCCAGAGGACCACTAAACTCTGTTTT--
ATTTTGTATCTCTGAATCTTACAACTAAATCAGTTAAAACTTTCAACAACGGATCTCTTGGTTCTGG
CATCGATGAAGAACGCAGCGAAATGCGATAAGTAATGTGAATTGCAGAATTCAGTGAATCATCGA
ATCTTTGAACGCACATTGCGCCCATTAGTATTCTAGTGGGCATGCCTATTCGAGCGTCATTTCAACC
CTTAAGCCTCAGTAGCTTAGCGTTGGGAATCTACAGGTTACAGCGTAGTTCCTTAAAGGTAGTGGC
GGAGTTAGAGCACACTCTAAGCGTAGTACTGTTTCTCGCTTCTGTAGTGGCTTTGGCGGCCGGCCGT
GAAACCCCCTATATTTCTAATGGTTGACCTCGGATTAGGTAGGAATACCCGCTGAACTTAAGCATA
TGAAGCGGCAACAGCTCAAA-TTTGAAATCTGGCC----TTC-----G-
GGTCCGAGTTGTAATTTGTAGAGGATGCTTTGGGTGCGGT-
GCCTTCCGAGTTCCCTGGAACGGGACGCCTTAGAGGGTGAGAGCCCCGTACGGTTGGA-
CACCTAGCCTATGTATAGCTCCTTCGACGAGTCGAGTAGTTTGGGAATGCTGCTCTAAATGGGAGG
TAAATTTCTTCTAAAGCTAAATACCGGCCAGAGACC-
GATAGCGCACAAGTAGAGTGATCGAAAGATGAAAAGCACTTTGAAAAGAGGGTTAAATAGCACGT
GAAATTGTTGAAAGGGAAGCG-TTTGCGACCAGACCTTTTCCAGGCGGATCATCCGGTGTTC--
TCACCGGTGCACTTCGTCTGG--TCGAGGCCAGCATCGGTTCT---
CCGCAGGGGATAAAGGCTCGGGGCATGTAGCTC----TTTCGGGAGTGTT-
ATAGCCCCTTGCGTAATATCCTTCG-GGGGACCGAGGACCGCGCA-TT----
GCAAGGATGCTGGCGTAATGGTCGTCAACGACCCGTCTTGAAACACGGACCAAGGAGTCGAACAT
TTGTGCGAGTGTTTGGGTG--TCAAACCCTCACGCGTAATGAAAGTGAACGGAGGTGAGAGCCCCT--
---CG---GGGTGCATCATCGACCGATCCTGATG-
TCTTCGGATGGATTTGAGTAAGAGCATAACTGTTCGGACCCGAAAGATGGTGAACTATGCGTGGAT
AGGGTGAAGCCAGAGGAAACTCTGGTGGAGGCTCGCAGCGGTTCTGACGTGCAAATCGATCGTCA
AATCTGCGCATGGGGGCGAAAGACTTATCGA-
ACCATCTAGTAGCTGGTTACCGCCGAAGTTTCCCTCAGGATAGCAGTGT--TG-

TCTTCAGTTTTATGAGGTAAAGCGAATGATTAGGGACTCGGGGGCGCTATATTGCCTTCATCCATTC
TCAAACTTTAAATATGTAAGAAGCCCTTGTTACTTAATTGAACGTGGGCATTCGAATGTACCAACA
CTAGTGGGCCATTTTTGGTAAGCAGAACTGGCGATGCGGGATGAACCGAACGTGGGGTTAAGGTG
CCAGAGTGGACGCTCATCAGACACCACAAAAGGTGTTAGTACATCTTGACAGCAGGACGGTGGCC
ATGGAAGTCGGAATCCGCTAAGGACTGTGTAACAACTCACCTGCCGAATGTACTAGCCCTGAAAAT
GGATGGCGCTCAAGCGTCT-CACCCATACCCCACCCTTAAGGTAGAAACGATGCCCTAAGG----------
----
--------------CGT------TTGCCTCGA---
CCTTATCTCACTTAAGGCGAACGAACACCCCCATCGGAAGAGATGGGAAGCTCGCGAAACCCCGA
CAGCTCCACAACACCCATTGGGGTCTGGTCTGCCCGGCCGAGACGCCCGAAGGCCAAGCTTGTGGT
TTGGTGAAGAATTTGTCCTTGATGTGTTCCATCAGCGTGGGTACGTCGACGGATCCTATCGTAGATT
ACATGATTACTAGGAACATGGAGGTGTTGGAGGAGTACGAACCTATGCGATACCCTAACGCCACC
AAGATCTTCTTGAACGGTTCCTGGATCGGCGTGCATCAGGATCCCAAGACCCTCGTCCGAGATGTT
CAACACCTGCGCCGAACTAACCAGATCCCCTCCGAGGTATCGTTGGTCCGCGACATTCGTGACCGT
GAGTTCAAGATTTCTCGGATGCCGGTCGTGTCATGCGTCCGTTGTTTGTCGTACAGCAAGAGGAT--
----

GATGAGTCCAACGGTATCACCAAGGGCTCGTTGGCGCTTAACAAGAGCATGATCCAACGACTAGA
AGCAGATGCCGACATAGATCCTAAAAGTGAGGAGTATTTTGGCTGGCAAGGCCTGGTAGACGAAG
GAGCTATTGAATTCCTCGATGCCGAGGAGGAGGAGACTGCCATGATTTGCATGACGCCCGAGGATC
TAGAGATCTATCGCCAGAGCAAGGCCGGAATTGAAGTGTCTCAGGACAACGGT------
GACGAGATTAACAAGCGACTCAAGACGAAGTTGAACCCGACGACACATATGTATACACATTGTGA
GATCCATCCCAGTATGCTCCTGGGTATCTGCGCGAGCATTATTCCCTTCCCGGACCACAATCAGGCA
CCCCTGAACGCGTCCACTCAACCCCTTGATTTCTGCCCCTCACACACACAAATC---
ACCACAATAACCCAGACCTCTCTACGATACGCTATTGCGAAATTCTACAACGCTTTGATGAGAATT
TGAAGCTAACTACGTATTTTCATGTCAACAGGTTCACCTTCAGACCGGCCAGTGCGTAAGTACTATC

TACGATTCCCGACAAAACATGGCGCGAGAACATAGTGGGGCTCACATATATATCCTAGGGTAACCA
AATTGGTGCTGCTTTCTGGCAAACTATCTCTGGCGAGCATGGCCTCGATAGCGATGGTGTGTACGT
ATATCAATGGTCAATTTGAGCCTCAAAAATA-
TATCGACTGACCAATAAACAGCTATAACGGTACCTCGGAGCTCCAGCTCGAGCGCATGAGTGTCTA
CTTCAACGAGGTATGTAGCTCCAGAAATCCAGTATGGATATATAAAATCACCTACTAATTATCCCG
GCATCTACAGGGTTCTGGCAACAAGTATGTTCCTCGCGCCGTTCTCGTCGATCTCGAGCCCGGCACC
ATGGACGCCGTCCGTGCTGGTCCTTTCGGCCAGCTTTTCCGACCTGACAACTTCGTCTTTGGCCAGT
CCGGTGCCGGCAACAACTGGGCCAAGGGTCACTACACTGAAGGTGCTGAGCTTGTCGACCAAGTCC
TCGATGTCGTTCGTCGTGAGGCTGAAGGCTGCGACTGCCTTCAGGGCTTCCAGATCACCCACTCGCT
TGGTGGTGGTACCGGTGCCGGTATGGGTACTCTGTTGATCTCCAAGATCCGTGAAGAGTTCCCCGA
CCGAATGATGGCCACCTTCTCCGTCGTTCCCTCCCCCAAGGTCTCCGACACCGTCGTCGAGCCTTAC
AACGCCACCCTCTCCGTCCACCAGCTGGTCGAGAACTCTGACGAGACCTTCTGTATCGACAACGAG
GCTCTGTACGACATCTGCATGCGTACTCTGAAGCTGTCCAACCCCTCTTACGGTGACCTGAACCACC
TAGTCTCCGCCGTCATGTCCGGCGTTACCACCTGCTTGCGTTTCCCCGGTCAGCTGAACTCTGACCT
GCGCAAGCTCGCCGTGAACATGGTTCCTTTCCCTCGTCTCCACTTCTTCATGGTTGGATTCGCTCCTC
TGACCAGCCGTGGCGCGTACTCTTTCCGTGCCGTCACCGTTCCCGAGTTGACCCAGCAGATGTTCGA
CCCCAAGAACATGATGGCTGCCTCTGACTTCCGCAACGGTCGCTACCTGACGTGCTCTGCCATCTTG
TAGGATACCCTCTCCGAATT-
TTTTTGCTACTAATTTTACTAACTTGATATTTCCAGCCGTGGCAAGGTCTCCA

>Hypomontagnella_monticulosa

-CATTACTGAGTTATCAAAACTCCCAACCCTTTGTGAA-CTTACCACTGTTGCCTCGGCG---
AGTGTGC-TACCCTATAGCT-----------------------------ACCCTGTAGCTACCCGGG-AACACAT-
TCCAAGCTCGCCAGAGGACCTACCAACTCTGTTTT--
ATACTGTATCTCTGAACTTTATAACTAAATAAGTTAAAACTTTCAACAACGGATCTCTTGGTTCTGG
CATCGATGAAGAACGCAGCGAAATGCGATAAGTAATGTGAATTGCAGAATTCAGTGAATCATCGA
ATCTTTGAACGCACATTGCGCCCATTAGTATTCTAGTGGGCATGCCTATTCGAGCGTCATTTCAACC
CTTAAGCCTCAGTTGCTTAGTATTGGGACTCTACGACCTATAGCGTAGTTCCTTAAAGGTAGTGGCG
GAGTTATAGCACACTCTAAGCGTAGTAATCTCTCTCGCTTCTGTAGTGGTTATAGTTGCTAGCCATA
AAACACCCCCTA-
TTTTAATGGTTGACCTCGGATTAGGTAGGAATACCCGCTGAACTTAAGCATATGAAGCGGCAACAG

CTCAAA-TTTGAAATCTGGCC----TTC-----G-
GGTCCGAATTGTAATTTGTAGAGGATGCTTTGGGTGCGGT-
ACCTTCCGAGTTCCCTGGAACGGGACGCCGGAGAGGGTGAGAGCCCCGTACGGTTGGA-
TACCAAGCCTATGTATAGCTCCTTCGACGAGTCGAGTAGTTTGGGAATGCTGCTCTAAATGGGAGG
TAAATTTCTTCTAAAGCTAAATACCGGCCAGAGACC-
GATAGCGCACAAGTAGAGTGATCGAAAGATGAAAAGCACTTTGAAAAGAGGGTTAAATAGCACGT
GAAATTGTTGAAAGGGAAGCG-TTTGCGACCAGACCTTTTCCAGGCGGATCATCCGGTGTTC--
TCACCGGTGCACTTCGCCTGG--TTTAGGCCAGCATCGGTTTT---
CTTAGGGGGATAAAGGTTTAGGGCACGTAGCTC----CTTCGGGAGTGTT-
ATAGCCCTCTACGTAATACCCTTCG-GGGGACCGAGGACCGCGCA-TT----
GCAAGGATGCTGGCGTAATGGTCGTCAACGACCCGTCTTGAAACACGGACCAAGGAGTCGAACAT
TTGTGCGAGTGTTTGGGTG--TTAAACCCTCACGCGTAATGAAAGTGAACGGAGGTGAGAGCCCTT--
---CG---GGGTGCATCATCGACCGATCCTGATG-
TCTTCGGATGGATTTGAGTAAGAGCATAACTGTTCGGACCCGAAAGATGGTGAACTATGCGTGGAT
AGGGTGAAGCCAGAGGAAACTCTGGTGGAGGCTCGCAGCGGTTCTGACGTGCAAATCGATCGTCA
AATCTGCGCATGGGGGCGAAAGACTTATCGA-
ACCATCTAGTAGCTGGTTACCGCCGAAGTTTCCCTCAGGATAGCAGTGT--TG-
TCTTCAGTTTTATGAGGTAAAGCGAATGATTAGGGACTCGGGGGCGCTATATTGCCTTCATCCATTC
TCAAACTTTAAATATGTAAGAAGCCCTTGTTACTTAGTTGAACGTGGGCATTCGAATGTACCAACA
CTAGTGGGCCATTTTTGGTAAGCAGAACTGGCGATGCGGGATGAACCGAACGTGGGGTTAAGGTG
CCAGAGTGGACGCTCATCAGACACCACAAAAGGTGTTAGTACATCTTGACAGCAGGACGGTGGCC
ATGGAAGTCGGAATCCGCTAAGGACTGTGTAACAACTCACCTNCCGAATGTACTAGCCCTGAAAAT
GGATGGCGCTCAAGCGTCT-CACCCATACCTCACCCTTAGGGTAGAAACGATGCCCTAAGG-----------
-----
AACAGACGTTTCCAGATCGAACTCGCCGCTNAGCCCGCCATCATCACCAACGGGCTCAAGTACTCG
CTGGCCACGGGTAACTGGGGTGACCAGAAGAAGGCCATGAGCTCGACTGCCGGTGTATCACAGGT
CTTGAATCGATATACGT------TTGCGTCGA---
CCTTGTCTCACTTGAGGCGAACGAATACACCCATTGGAAGAGATGGGAAGCTCGCGAAGCCTCGAC
AGCTCCACAACACCCATTGGGGTTTGGTCTGCCCGGCCGAGACGCCCGAAGGCCAGGCTTGTGGTT
TGGTGAAGAACTTGTCCTTGATGTGCTCAATCAGCGTGGGTACATCGACAGATCCTATAGTAGACT
ACATGATTACGAGGAACATGGAAGTCTTGGAGGAGTATGAGCCAATGCGATACCCTAACGCCACC
AAGATCTTCTTGAATGGTTCTTGGATCGGTGTGCACCAGGATCCCAAGAGCCTCGTCCGAGACGTC
CAGCAGCTGCGCCGAACGAATCAGATCCCTTCCGAAGTATCGCTGGTCCGCGATATTCGTGACCGC
GAATTCAAGATCTTTTCCGATGCAGGCCGTGTCATGCGTCCCTTATTTGTCGTGCAGCAAGAGGAT--
----
GACGAAGCCAACGGTATCACCAAAGGCTCGTTAGCGCTGAATAAGAATATGATTCAGCGACTAGA
GGCGGATGCCGATATCGACCCGAAGAGTGAGGAGTATTTCGGCTGGCAAGGCCTTGTCGACGAGG
GAGCTATCGAATTCCTCGACGCCGAGGAAGAGGAGACCGCCATGATCTGCATGACGCCCGAGGAT
CTGGAAATCTACCGCCAGAGCAAAGCCGGCATCGAAGTATCCCAGGATAATGGT------
GACGAAATCAACAAGCGACTCAAGACCAAGTTGAACCCAACGACACATATGTATACGCATTGTGA
GATCCATCCCAGCATGCTTCTAGGTATCTGTGCGAGCATCATTCCCTTCCCGGACCACAATCAGGTA
CCCCTGAACGCGTCCACTCAACCCCTTGATTTTTGCCCCTCACGCACACAAAAC---
ACCACAAGAGCCTAGATATGTCTACGTTGC--TACCATGGAATCGTATAACA---
TATTGGAAATTTGAAACTAACCATGTCTTTTCATCTCGATAGGTTCACCTCCAGACCGGCCAGTGCG
TAAGTACTAC--C----
TACGATTACCGACGAAACATCGCGAAAACATAGTGGGAGGCTCACATAAATATTGTAGGGTAACC
AAATTGGTGCTGCTTTCTGGCAAACTATCTCTGGCGAGCACGGCCTCGACAGCGATGGTGTGTACG
TATATCAATGGTCAATTCAAGCTCCGA-----
ATAACAACTGACCAATAAACAGCTACAACGGTACCTCTGAGCTCCAGCTCGAGCGCATGAGCGTCT
ACTTCAACGAGGTATGTAGCAACAGAAATCCCGTATAGATATACAAGATCGGCTACTAATCACCCT
GATATGTACAGGGTTCTGGCAACAAGTATGTTCCTCGCGCTGTTCTCGTCGATCTCGAGCCCGGTAC
CATGGATGCCGTCCGTGCTGGTCCCTTCGGCCAGCTTTTCCGACCTGACAACTTCGTCTTTGGCCAG
TCCGGTGCCGGCAACAACTGGGCCAAGGGTCACTACACTGAGGGTGCTGAGCTTGTTGACAACGTT
CTCGATGTCGTTCGTCGTGAGGCTGAAGGCTGCGACTGCCTTCAGGGTTTCCAGATTACCCACTCGC
TCGGTGGTGGTACCGGTGCCGGTATGGGTACTCTGCTGATCTCCAAGATCCGTGAAGAGTTCCCCG
ACCGAATGATGGCCACCTTCTCCGTCGTTCCCTCCCCCAAGGTCTCTGACACCGTTGTCGAGCCTTA
CAACGCTACCCTTTCCGTCCACCAGCTTGTCGAGAACTCCGACGAGACCTTCTGTATCGACAACGA

GGCGCTGTACGATATCTGCATGCGTACCCTGAAGCTATCCAACCCCTCGTATGGTGACCTAAACCA
CCTGGTCTCCGCCGTCATGTCCGGTGTCACCACCTGCTTGCGTTTCCCTGGTCAGTTGAACTCTGAC
CTGCGCAAGCTTGCTGTGAACATGGTTCCTTTCCCCCGTCTCCACTTCTTCATGGTTGGCTTCGCTCC
TCTGACCAGCCGTGGCGCGTACTCTTTCCGTGCCGTCACCGTTCCCGAGTTGACCCAGCAGATGTTC
GACCCCAAGAACATGATGGCTGCTTCTGACTTCCGTAACGGTCGTTACCTGACGTGCTCTGCCATCT
TGTAAGATGCCTCTTTCGAACC--

>Hypoxylon_griseobrunneum

-CATTACTGAGTTCT--AAACT-CCAACCCTATGTGAA-CTTACCACTGTTGCCTCGGCG-----CGTGCC--
----
TGCGAGAGCAGGCCCGCCGGTGGACCACTAAACTCTGCTATACCTACTGTATCTCTGAATTT-
ATAACTAAATACGTTAAAACTTTCAACAACGGATCTCTTGGTTCTGGCATCGATGAAGAACGCAGC
GAAATGCGATAAGTAATGTGAATTGCAGAATTCAGTGAATCATCGAATCTTTGAACGCACATTGCG
CCCATTAGTATTCTAGTGGGCATGCCTATTCGAGCGTCATTTCAACCCTTAAGCCCCTGTTGCTTAG
TGTTGGGAATCTGCG----
ACGGCGCAGTTCCTTAAAGTGATTGGCGGAGCTAGTGCATACTCTAGGCGTAGTAAACATTCTCGC
TTTTGTAGTAGGCCTGGCGGCTTGCCGTAAAACCCC-
TATATTTCTAGTGGTTGACCTCGGATTAGGTAGGAATACCCGCTGAACTTAAGCATA------------------
A-TTTGAAATCTGGCC----CTA-----GTGGTCCGAGTTGTAATTTGTAGAGGATGCTTTGGGTGCGGT-
GCCTTCTGAGTTCCCTGGAACGGGACGCCAGAGAGGGTGAGAGCCCCGTACGGTTGGA-
CACCTAGCCTATATATAGCTCCTTCGACGAGTCGAGTAGTTTGGGAATGCTGCTCTAAATGGGAGG
TATATTTCTTCTAAAGCTAAATACCGGCCAGAGACC-
GATAGCGCACAAGTAGAGTGATCGAAAGATGAAAAGCACTTTGAAAAGAGGGTTAAATAGCACGT
GAAATTGTTGAAAGGGAAGCG-TTTGCGACCAGACCTTTTCCGGGCGGATCATCCGGCGTTC--
TCGCCGGTGCACTTCGCCCGG--TTTAGGCCAGCATCGGTTTT---
CTTAGGGGGATAAAGGCCAGGGGAACGTAGCTC----TTTCGGGAGTGTT-
ATAGCCCCTGGTGTAATACCCTTCA-GGGGACCGAGGACCGCGCT-TTT---
GCAAGGATGCTGGCGTAATGGTCGTCAACGACCCGTCTTGAAACACGGACCAAGGAGTCGAACAT
TGGTGCGAGTGTTTGGGTG--TTAAACCCTCACGCGTAATGAAAGTGAACGGAGGTGAGAGCCCTT--
--AC----GGGTGCATCATCGACCGATCCTGATG-
TCTTCGGATGGATTTGAGTAAGAGCATCACTGTTCGGACCCGAAAGATGGTGAACTATGCGTGGAT
AGGGTGAAGCCAGAGGAAACTCTGGTGGAGGCTCGCAGCGGTTCTGACGTGCAAATCGATCGTCA
AATCTGCGCATGGGGGCGAAAGACTTATCGA-
ACCATCTAGTAGCTGGTTACCGCCGAAGTTTCCCTCAGGATAGCAGTGT--TG-
TCTTCAGTTTTATGAGGTAAAGCGAATGATTAGGGACTCGGGGGCGCTATATTGCCTTCATCCATTC
TCAAACTTTAAATATGTAAGAAGCCCTTGTTACTTAATTGAACGTGGGCATTCGAATGTACCAACA
CTAGTGGGCCATTTTTGGTAAGCAGAACTGGCGATGCGGGATGAACCGAACGCGGGGTTAAGGTG
CCAGAGTGGACGCTCATCAGACACCACAAAAGGTGTTAGTACATCTTGACAGCAGGACGGTGGCC
ATGGAAGTCGGAATCCGCTAAGGACTGTGTAACAACTCACCTGCCGAATGTACTAGCCCTGAAAAT
GGATGGCGCTCAAGCGTCT-CACCCATACCTCGCCCTCAGGGTAGAAACGATGCCCTGAGG-----------
----
AGCTTGCGGCGAAGCCCTCCATCATCACCAACGGTCTGAAGTACTCTCTCGCGACGGGCAACTGGG
GCGACCAGAAGAAGGCGATGAGCTCGACCGCTGGCGTGTCGCAGGTTCTGAACCGATACACTT------
TCGCCTCCA---
CCCTTTCTCATCTGAGAAGAACCAACACGCCTATTGGAAGAGACGGAAAGCTCGCAAAGCCCAGG
CAGCTGCACAACACGCACTGGGGATTGGTCTGTCCGGCCGAGACGCCCGAAGGCCAGGCTTGTGG
GTTGGTGAAGAACCTGTCGCTGATGTGCTCCATCAGCGTGGGAACGTCCACAGACCCTATTGTCGA
TTACATGATCACGAGAAACATGGAGGTTTTGGAAGAGTACGAACCCATGCGTTATCCCAACGCCAC
CAAGATTTTCCTCAACGGTTCTTGGATTGGTGTGCACCAGGATGCCAAGACTCTGGTCAAGGACGT
GCAGGAGCTCCGTCGCTCCAACCAGATTCCCTCGGAAGTATCGCTGATACGCGACATCCGTGACCG
CGAGTTCAAGATCTTCTCGGACGCCGGCCGCGTCATGCGCCCGCTCTTCGTGGTGCAGCAGGAGGA
C---
ATCCCGGAGCGAGGCATCGCCAAAGGCACCCTAGCTCTCACAAAAGAAATGGTTCAGCGATTAGA
GGCGGATCAGGAGCTTGACCCTGAGAGCGACGACTACTTCGGCTGGCAAGGCCTGTGTGAGGCGG
GTGTCATCGAGTACCTGGATGCCGAGGAAGAGGAGACTGCCATGATCTGCATGACGCCCGAAGAC
TTGGAAACTTATCGGATGGCCAAGGCCGGGTACGACATCCCTCAAGACAACGGC------
GACGAAGTCAACAAGCGACTGAAGACCAAGGTCAACCCCTCGACACACATGTATACTCACTGTGA

GATCCATCCCAGCATGCTTTTGGGAATTTGCGCGAG------------------------------
CCCCTGAACGCGTCCATGCAATCCCCTGATATCTACCCCTCACGTACACCAAACACGACCACAATC
G-
CTGTAGCCTTCGCAGTCATGTCGCCGTCTGATTTTACTACGACGGAATTCGAAATGGAAGCTAACTC
AATATTTTC--ATGCATAGGTTCACCTCCAGACCGGCCAGTGCGTAAGTACAACCAA----
AACCTCGATCGAGGAAACGTCGCGAAGAAACATGGC--
AGCTCACACGATATCAACAGGGTAACCAAATTGGTGCTGCTTTCTGGCAGACCATCTCTGGCGAGC
ACGGTCTCGACAGCAACGGTGTGTACGTATCAAGATCGCCCGCTCGATTGTCAAGAACACAAATTA
ACGACCAATCAACAGCTACAATGGCACCTCCGAGCTCCAGCTCGAGCGCATGAGCGTCTACTTCAA
CGAGGTACACAGTCAAAGAA---
GCCTCCCCTTTTGCGAGATCGGTTATTAATCACCCTAACATACACAGGCTTCTGGTAACAAGTATGT
TCCCCGCGCCGTCCTCGTCGATCTCGAGCCCGGTACCATGGACGCCGTCCGTGCCGGTCCCTTCGGT
CAGCTCTTCCGCCCCGACAACTTCGTCTTCGGTCAGTCCGGTGCCGGCAACAACTGGGCCAAGGGT
CACTACACGGAGGGTGCTGAGCTTGTCGACAACGTTCTCGATGTCGTCCGTCGCGAGGCTGAGGGC
TGCGACTGCCTCCAGGGCTTCCAGATCACCCACTCCCTCGGTGGTGGTACCGGTGCCGGTATGGGT
ACCTTGTTGATCTCCAAGATCCGCGAGGAGTTCCCCGACCGCATGATGGCCACCTTCTCCGTCGTGC
CCTCTCCCAAGGTCTCCGACACCGTCGTCGAGCCCTACAACGCCACCCTCTCCGTGCACCAGCTCGT
CGAGAACTCCGACGAGACCTTCTGTATCGACAACGAGGCCCTGTACGACATCTGCATGCGTACCCT
GAAGTTGTCCAACCCCTCGTACGGTGACCTGAACCACCTAGTCTCTGCCGTCATGTCCGGTGTCACC
ACCTGTCTGCGCTTCCCCGGCCAGCTGAACTCGGACCTGCGCAAGCTCGCTGTGAACATGGTTCCTT
TCCCTCGTCTCCACTTCTTCATGGTCGGCTTCGCTCCCCTGACCAGCCGTGGTGCCTACTCCTTCCGC
GCCGTCACCGTTCCCGAGTTGACTCAGCAGATGTTCGACCCCAAGAACATGATGGCTGCCTCTGAC
TTCCGCAACGGTCGCTACCTGACGTGCTCTGCCATCTTGTAAGATTCCCTACCGACAAAACATATAG
TGATTGATGTGCTAACCCAACTTCTCCAGCCGTGGCAAGGTCTCCA

>Hypoxylon_aveirense_MUM1940

GCATTACTGAGTTCT--AAACTCC-AACCCTATGTGAA-CTTACCACTGTTGCCTCGGCGTG--------------
------T--G--CTCGCGA-
CAGCAGCCCGCCAGTGGGCCTTTAAACTCTGATATTACCACTGTATCTCTGAATTTCTTAACTAAAT
ACGTTAAAACTTTCAACAACGGATCTCTTGGTTCTGGCATCGATGAAGAACGCAGCGAAATGCGAT
AAGTAATGTGAATTGCAGAATTCAGTGAATCATCGAATCTTTGAACGCACATTGCGCCCATTAGTA
TTCTAGTGGGCATGCCTATTCGAGCGTCATTTCAACCCTTGCGCCCCTGTTGCGTAGCGTTGGGAAT
CTGCGT---
ACAGCGCAGTTCCTCAAAGTGACTGGCGGAGCTAGTGCATACTCTAAGCGTAGTAAACATTCTCGC
TTCTGTAGTAGGCCTGGCGGCTAGCCGTTAAACCCCCTATACTTCTAGTGGTTGACCTCGGATTAGG
TAGGAATACCCGCTGAACTTAA-----TGAAGCGGCAACAGCTCAAA-TTTGAAATCTGGCC----CTC-----
GTGGTCCGAGTTGTAATTTGTAGAGGATGCTTTGGGTGCGGT-
GCCTTCCGAGTTCCCTGGAACGGGACGCCAGAGAGGGTGAGAGCCCCGTACGGTTGGA-
CACCTAGCCTATCTATAGCTCCTTCGACGAGTCGAGTAGTTTGGGAATGCTGCTCTAAATGGGAGG
TATATTTCTTCTAAAGCTAAATACCGGCCAGAGACC-
GATAGCGCACAAGTAGAGTGATCGAAAGATGAAAAGCACTTTGAAAAGAGGGTTAAATAGCACGT
GAAATTGTTGAAAGGGAAGCG-TTTGCGACCAGACCTTCTCCGGGCGGATCATCCGGCGTTC--
TCGCCGGTGCACTTCGCCCGG--TTTAGGCCAGCATCGGTTCT---
CTTAGGGGGATAAAGGCCAGGGGAACGTAGCTC----TTTCGGGAGTGTT-
ATAGCCCCTGGCGCAATACCCCTCG-GGGGACCGAGGACCGCGCT-TTT---
GCAAGGATGCTGGCGTAATGGTCGTCAACGACCCGTCTTGAAACACGGACCAAGGAGTCGAACAT
TGGTGCGAGTGTTTGGGTG--TCAAACCCTCACGCGTAATGAAAGTGAACGGAGGTGAGAGCCCGT--
--AC----GGGTGCATCATCGACCGATCCTGATG-
TCTTCGGATGGATTTGAGTAAGAGCATCACTGTTCGGACCCGAAAGATGGTGAACTATGCGTGGAT
AGGGTGAAGCCAGAGGAAACTCTGGTGGAGGCTCGCAGCGGTTCTGACGTGCAAATCGATCGTCA
AATCTGCGCATGGGGGCGAAAGACTTATCGA-
ACCATCTAGTAGCTGGTTACCGCCGAAGTTTCCCTCAGGATAGCAGTGT--TG-
TCTTCAGTTTTATGAGGTAAAGCGAATGATTAGGGACTCGGGGGCGCTATATTGCCTTCATCCATTC
TCAAACTTTAAATATGTAAGAAGCCCTTGTTACTTAGTTGAACGTGGGCATTCGAATGTACCAACA
CTAGTGGGCCATTTTTGGTAAGCAGAACTGGCGATGCGGGATGAACCGAACGCGGGGTTAAGGTG
CCAGAGTGGACGCTCATCAGACACCACAAAAGGTGTTAGTACATCTTGACAGCAGGACGGTGGCC
ATGGAAGTCGGAATCCGCTAAGGACTGTGTAACAACTCACCTGCCGAATGTACTAGCCCTGAAAAT

GGATGGCGCTCAAGCGTCT-
CACCCATACCTCGCCCTCAGGGTAGAAACGATGCCCTGAGGGAGGTGTATCGAATCTAACAAACG
ATTTTACGTCGAGCTTGCGGCTAAGCCTTCCATCATCACCAACGGTCTGAAGTACTCTCTTGCGACA
GGCAACTGGGGCGACCAGAAGAAGGCGATGAGCTCGACCGCCGGCGTGTCGCAGGTCCTGAACCG
ATACACTT------TCGCGTCCA---
CCCTTTCTCATTTGAGAAGAACCAACACGCCCATCGGAAGAGATGGAAAGCTTGCGAAGCCCAGA
CAGCTGCACAACACGCACTGGGGATTGGTCTGTCCGGCTGAGACGCCCGAAGGCCAGGCTTGTGG
ACTGGTCAAGAATCTGTCGCTGATGTGCTCCATCAGCGTGGGAACGTCTACGGACCCCATCGTCGA
CTACATGATTACCAGGAACATGGAAGTCTTGGAGGAGTACGAACCCATGCGTTATCCCAACGCCAC
CAAGATTTTCCTCAACGGTTCTTGGATTGGTGTCCACCAGGATGCCAAGACCCTTGTTAAGGATGTC
CAGGAGCTTCGTCGCTCCAACCAAATCCCCTCAGAAGTATCTCTGATTCGCGACATTCGTGACCGT
GAGTTCAAGATCTTCTCGGACGCTGGCCGCGTGATGCGCCCGCTGTTCGTGGTACAGCAGGAGGAC

ATCCCCGAACGAGGCATCGCCAAGGGCACCCTGGCTCTCACCAAAGAAATGGTCCAGCGGCTAGA
AGCAGATCAGGAGCTTAACCCCGACAGCGATGAGTATTACGGCTGGCAAGGTCTGTGCGAGGCGG
GCGCCATCGAGTACCTCGACGCCGAGGAGGAGGAAACGGCCATGATTTGCATGACGCCCGAAGAC
TTGGAAACCTATCGATTGGCCAAGGCCGGGTACGACATCCCTCAGGACAACGGC------
GACGAGGTCAACAAGCGACTGAAGACCAAGGTCAACCCCTCGACGCATATGTACACCCACTGCGA
AATCCATCCCAGCATGCTTCTAGGAATCTGCGCGAGTATCATTCCCTTCCCCGACCACAATCAGTCG
CCCCTGAACGCGTCCCATCAATCCCCTGATGTCTGCCCCTCACGCACACGAAACACGACCGCAGTC
CCTTCCACCCTTCGCCGCCATGTCGCTGTCTGATTTTACTACGATGGAAGTGGAAGTGAAGGCTAAT
CCGATATTTTCTCATATATAGGTTCACCTCCAGACCGGCCAGTGCGTAAGTACAACCAG----
AGCCTCGATCGACGAGACGTCGCGATGGAACATAGC--
AGCTCACACGATGCAAATAGGGTAACCAAATTGGTGCTGCTTTCTGGCAGACCATTTCTGGCGAGC
ACGGTCTCGACAGCAACGGTGTGTACGTATTAGGGTCGCCAATTTGATTGTCCATCACACCAGCTA
ACGACCAATAAACAGGTACAATGGAACCTCGGAGCTCCAGCTCGAGCGCATGAGCGTCTACTTCA
ACGAGGTACGCGGTCGAAGAA---
GCCACCCGTTTTGTGAGATCGGTTATTAATCACCCTAACATACACAGGCTTCTGGTAACAAGTATGT
TCCCCGCGCCGTCCTCGTCGATCTCGAGCCCGGTACCATGGACGCCGTCCGTGCCGGCCCCTTCGGT
CAGCTCTTCCGTCCCGACAACTTCGTCTTCGGTCAGTCCGGTGCCGGCAACAACTGGGCCAAGGGT
CACTACACCGAGGGT--

>Hypoxylon_fendleri

-CATTACAGAGTTATCTAAACTCCAAACCCTATGTGAA-CCTACCACTGTTGCCTTGGCGTG-
TGCGCG----------------G------------------------------CTACCCTGT-AGTGCGC-
ATACGGCCCGCCGAAGGACCACTAAACTCTTTTTTTACTGTGAATATCTGAATGCTTCAACTTAAT
AAGTTAAAACTTTCAACAACGGATCTCTTGGTTCTGGCATCGATGAAGAACGCAGCGAAATGCGAT
AAGTAATGTGAATTGCAGAATTCAGTGAATCATCGAATCTTTGAACGCACATTGCGCCCATTAGTA
TTCTAGTGGGCATGCCTATTCGAGCGTCATTTCAACCCTTAAG-
CCCTGTTGCTTAGCGTTGGGAATCAGCGTCTTTCGGCGCTGTTCCTTAAATTTAGTGGCGGAGTTAT
AGCACACTCTAAGCGTAGTAAATCTTCTCGCTTCTGAAGTTGCCCTAATTCTTAGCCGTAAAACCCC
CTAT-TTTCTAATGGTTGACCTCGGATTAGGTAGGAATACCCGCTGAACTTAAGCATA-------------
CTCAAA-TTTGAAATCTGGCC----CTA-----
GCGGTCCGAGTTGTAATTTGTAGAGGATGCTTTTGGTGAGGT-
GCCTTCTGAGTTCCCTGGAACGGGACGCCAGAGAGGGTGAGAGCCCCGTACGGTCGGC-
CACCGAACCTATATATAGCTCCTTCGACGAGTCGAGTAGTTTGGGAATGCTGCTCTAAATGGGAGG
TAAATTTCTTCTAAAGCTAAATACCGGCCAGAGACC-
GATAGCGCACAAGTAGAGTGATCGAAAGATGAAAAGCACTTTGAAAAGAGGGTTAAATAGCACGT
GAAATTGTTGAAAGGGAAGCG-TTTGCGACCAGACTTTTTCCTGTCGGATCATCCGGTGTTC-
TTCACCGGTGCACTTCGGCAGG--TTTAGGCCAGCATCGGTTCT---
CTTAGGGGGATAAAGGCCTTGGGAACGTAGCTC----TTTAGGGAGTGTT-
ATAGCCCCTGGCGCAATACCCTTCG-GGGGACCGAGGATCGCGCT-CT----

GCAAGGATGCTGGCGTAATGGTCGTCAACGACCCGTCTTGAAACACGGACCAAGGAGTCGAACAT
TTGTGCGAGTGTTTGGGTG--TCAAACCCTCACGCGTAATGAAAGTGAACGGAGGTGAGAGCCTT----
-A-----GGGCGCATCATCGACCGATCCTGATG-
TCTTCGGATGGATTTGAGTAAGAGCATAACTGTTCGGACCCGAAAGATGGTGAACTATGCGTGGAT
AGGGTGAAGCCAGAGGAAACTCTGGTGGAGGCTCGCAGCGGTTCTGACGTGCAAATCGATCGTCA
AATCTGCGCATGGGGGCGAAAGACTTATCGA-
ACCATCTAGTAGCTGGTTACCGCCGAAGTTTCCCTCAGGATAGCAGTGT--TG-
TCTTCAGTTTTATGAGGTAAAGCGAATGATTAGGGACTCGGGGGCGCTATATTGCCTTCATCCATTC
TCAAACTTTAAATATGTAAGAAGCCCTTGTTACTTAGTTGAACGTGGGCATTCGAATGTACCAACA
CTAGTGGGCCATTTTTGGTAAGCAGAACTGGCGATGCGGGATGAACCGAACGCGGGGTTAAGGTG
CCAGAGTGGACGCTCATCAGACACCACAAAAGGTGTTAGTACATCTTGACAGCAGGACGGTGGCC
ATGGAAGTCGGAATCCGCTAAGGACTGTGTAACAACTCACCTGCCGAATGTACTAGCCCTGAAAAT
GGATGGCGCTCAAGCGTCT-CACCCATACCTCGCC------------------------
ACGCTGTATTGAGACCAACAGAAGATTCCAAATCGAGCTTGCTGCTAAGCCTGCCATCGTCACCAA
CGGACTGAAGTATTCACTCGCTACGGGTAACTGGGGTGATCAGAAGAAGGCTATGAGTTCGACAG
CTGGTGTATCTCAAGTTTTGAACCGTTACACAT------TCGCATCGA---
CCCTATCTCATTTAAGAAGAACCAACACTCCTATCGGAAGAGATGGTAAATTGGCCAAACCTCGAC
AACTTCACAATACTCATTGGGGTTTGGTCTGTCCGGCTGAAACCCCTGAAGGTCAAGCTTGTGGAC
TTGTGAAGAATTTGTCACTTATGTGCTCCATCAGCGTGGGTACATCGACAGACCCTATCATCGACTA
CATGATTACCAGGAACATGGAAGTCCTTGAGGAATACGAACCCATGAGATATCCTAATGCTACCAA
GATCTTCCTCAATGGTTCGTGGATCGGTGTACACCAAGATCCCAAGTCTCTTGTCAGGGATGTTCAG
CAGCTACGTCGAGCCAACCAGATTCCCTACGAAGTTTCTCTTGTACGCGACATTCGTGATCGTGAGT
TCAAGATTTTTTCGGATGCTGGTCGTGTCATGCGTCCTTTGTTTGTCGTTCAACAAGAGGA------------
--TTCCGATTCT---
GCCCAGAAAGGTTCTTTGGCCCTCACAAAGGACATGATACAGAGACTCGAGGCAGATGTTGACTTG
GATCCTGAGAGCGAGGATTATTTCGGATGGCAGGGTCTAGTCAACGAAGGTGTTATCGAATATCTC
GATGCCGAAGAAGAAGAGACTGCCATGATTTGCATGACACCTGAAGACTTGGAGAATTACCGATT
GACCAAGGCTGGGGTTGATGTTTACCAGGACAANGGA------
GATGAAATTAACAAACGTCTCAAGACCAAGGTCAACCCAACGACACATATGTATACTCATTGTGAG
ATTCATCCTAGTATGTTGTTGGGTATTTGCGCCAGTATTATTCCATTCCCGGATCATAATCAGGTAC
CCCTGAACGCGTCCCTCAATCCCCTGATTTCCTGCCCCTCACGCACAAAAAAC---
ATCACAACACTTGATAG--
CTATATATTGAGCTGCTATTTAATTACACAATGACATGATAAGGAATTGAAGCTAACCATCTTTTTT
TGTCACTATAGGTTCACCTCCAGACCGGCCAGTGCGTAAGTAAC----------ACGACCATCAACGA-
ATATTGCGATGAGACATTGCGGGGCTCACACGAAGTAAATAGGGTAACCAAATTGGTGCTGCCTTC
TGGCAAACCATCTCTGGCGAGCACGGCCTCGACAGCAATGGCGTGTACGTAAATAATTCGCCAATT
CTATCGAGAGGATAACCAACTAATGGTCAATAAACAGCTACAACGGTACTTCCGAGCTCCAGCTCG
AGCGCATGAGCGTCTACTTCAACGAGGTATGCACAGAGAAGAATTGGGAAAAGATGCAAAAGATT
GGTTATTAATCATCATA------
ACAGGCTTCCGGCAACAAGTATGTTCCTCGCGCCGTCCTCGTCGATCTCGAGCCCGGTACCATGGA
TGCCGTCCGTGCTGGTCCCTTCGGTCAGCTCTTCCGACCCGACAACTTCGTCTTCGGTCAGTCTGGT
GCCGGAAACAACTGGGCCAAGGGTCACTACACTGAGGGTGCTGAGCTTGTTGACAACGTCCTCGAT
GTCGTTCGTCGTGAGGCTGAGGGTTGTGATTGCCTTCAGGGTTTCCAGATCACCCACTCCCTCGGTG
GTGGCACTGGTGCTGGTATGGGTACTTTGTTGATCTCCAAGATCCGTGAGGAGTTCCCCGACCGAA
TGATGGCCACCTTTTCCGTTGTTCCCTCTCCCAAGGTCTCCGACACCGTCGTCGAGCCTTACAACGC
CACCCTCTCGGTCCACCAGCTGGTCGAGAACTCGGACGAGACCTTCTGCATTGACAACGAGGCTCT
GTACGACATCTGCATGCGTACCCTCAAGCTGTCTAACCCCTCCTATGGCGACCTGAACCACCTCGTC
TCCGCTGTCATGTCTGGTGTCACCACTTGCCTGCGTTTCCCCGGTCAGCTGAACTCTGACCTGCGCA
AGCTTGCCGTCAACATGGTTCCCTTCCCTCGTCTGCACTTCTTCATGGTTGGCTTCGCTCCTTTGACC
AGCCGTGGCGCCCACTCTTTCCGCGCCGTCACCGTCCCCGAGTTGACCCAGCAGATGTTCGACCCC
AAGAACATGATGGCTGCTTCTGACTTCCGCAACGGTCGCTACCTGACGTGCTCTGCCATCTTGTGAG
ATATTTATTCCAGCTAACCCGTTTTCGCATATTTGCTAACCCGTATTCTGTAGCCGTGGCAAGGTCT
CCA

>Hypoxylon_invadens

-CATTACAGAGTTACAAAAACTCCCAACCCTTTGTGAA-CTTACCACTGTTGCCTCGGCGCT-
GGCGCGGCTACCCTATAGCTACCCTG--------------TAGGAAACTACCCTGTACCTATCTTGT-

AGTACGC--
TTAGGCCCGTCGGAGGACCATTGAACTCTTGTTTTTACTGTGAATATCTGAATGCTTCAACTAAATA
AGTTAAAACTTTCAACAACGGATCTCTTGGTTCTGGCATCGATGAAGAACGCAGCGAAATGCGATA
AGTAATGTGAATTGCAGAATTCAGTGAATCATCGAATCTTTGAACGCACATTGCGCCCATTAGTAT
TCTAGTGGGCATGCCTATTCGAGCGTCATTTCAACCCTTAAGCCCCTGTTGCTTAGCGTTAGGAGAC
TGAGCCCCGGGGCCCAGCTCCCTAAAACTAGTGGCGATGTTATGGCACATCCTAAGCGTAGTAATT
CTTCTCGCTTCTGTGATTGTCGAACCTATCTGCCGTAAAACCCCCTATA-
TTCTAGTGGTTGACCTCGGATTAGGTAGGAATACCCGCTGAACTTAAGCATA------------------A-
TTTGAAATCTGGCC----CTC-----GTGGTCCGAGTTGTAATTTGTAGAGGATGCTTTTGGTGCGGT-
GCCTTCCGAGTTCCCTGGAACGGGACGCCATAGAGGGTGAGAGCCCCGTACGGTTGGA-
CGCCTACCCTATATATAGCTCCTTCGACGAGTCGAGTAGTTTGGGAATGCTGCTCTAAATGGGAGG
TAAATTTCTTCTAAAGCTAAATACCGGCCAGAGACC-
GATAGCGCACAAGTAGAGTGATCGAAAGATGAAAAGCACTTTGAAAAGAGGGTTAAATAGCACGT
GAAATTGTTGAAAGGGAAGCG-TTTGCGACCAGACTTTTTCCAGGCGGATCATCCGGTGTTC--
TCACCGGTGCACTTCGCCTGG--TTTAGGCCAGCATCGGTTTC---
CTTAGGGGGATAAAGGCCTGGGGAACGTAGCTC----TCTAGGGAGTGTT-
ATAGCCCCTCGCGTAATACCCCTCG-GGGGACCGAGGATCGCGCT-CT----
GCAAGGATGCTGGCGTAATGGTCGTCAACGACCCGTCTTGAAACACGGACCAAGGAGTCGAACAT
TTGTGCGAGTGTTTGGGTG--TTAAACCCTCACGCGTAATGAAAGTGAACGGAGGTGAGAGCCCTT--
--AC----GGGTGCATCATCGACCGATCCTGATG-
TCTTCGGATGGATTTGAGTAAGAGCATAACTGTTCGGACCCGAAAGATGGTGAACTATGCGTGGAT
AGGGTGAAGCCAGAGGAAACTCTGGTGGAGGCTCGCAGCGGTTCTGACGTGCAAATCGATCGTCA
AATCTGCGCATGGGGGCGAAAGACTTATCGA-
ACCATCTAGTAGCTGGTTACCGCCGAAGTTTCCCTCAGGATAGCAGTGT--TG-
TATTCAGTTTTATGAGGTAAAGCGAATGATTAGGGACTCGGGGGCGCTATTTAGCCTTCATCCATTC
TCAAACTTTAAATATGTAAGAAGCCCTTGTTGCTTAGTTGAACGTGGGCATTCGAATGTACCAACA
CTAGTGGGCCATTTTTGGTAAGCAGAACTGGCGATGCGGGATGAACCGAACGCGGGGTTAAGGTG
CCAGAGTGGACGCTCATCAGACACCACAAAAGGTGTTAGTACATCTTGACAGCAGGACGGTGGCC
ATGGAAGTCGGAATCCGCTAAGGACTGTGTAACAACTCACCTGCCGAATGTACTAGCCCTGAAAAT
GGATGGCGCTCAAGCGTCT-CACCCATACCTCGCCC-----------------------
GCGCTGTATCGATCAGAATAGACGTTTCCAGATTGAGCTCGCAGCCAAGCCCTCCATCGTCACCAA
CGGGCTAAAGTATTCCCTGGCTACAGGTAACTGGGGCGACCAGAAAAAGGCGATGAGCTCAACCG
CCGGTGTGTCACAGGTGTTGAACCGATATACAT------TCGCATCAA---
CCTTGTCACATTTGAGGCGAACCAACACCCCCATCGGAAGAGACGGGAAACTAGCGAAGCCTCGA
CAACTTCACAACACCCATTGGGGTCTGGTCTGTCCGGCCGAAACGCCCGAGGGTCAGGCCTGTGGG
TTGGTGAAGAATCTGTCGCTGATGTGTTCTATCAGCGTGGGTACATCGACGGATCCTATCGTGGACT
ATATGATTACTAGGAATATGGAAGTCCTTGAGGAATATGAACCGATGCGATACCCCAACGCCACCA
AGATTTTCCTGAATGGTTCTTGGATTGGTGTACATCAGGACCCCAAGTCTCTGGTTAGAGATGTTCA
GCAGCTTCGCCGGGCTAACCAGATCCCCTCAGAAGTATCGTTGGTCCGCGATATTCGTGATCGCGA
GTTCAAGATCTTCTCAGATGCCGGTCGTGTTATGCGCCCCTTGTTTGTCGTGCAACAAGATGAC------
----
AATCCGGAAGCCGGTATCATCAAAGGCACATTGGCTCTCAACAAGGACATGATCCAACGGCTAGA
GGCCGATGTTGACTTAGATCCCGACAGTGATGAGTACTTTGGCTGGCAGGGTCTGGTTAATGAGGG
TGTTATCGAGTACCTTGATGCCGAGGAAGAGGAAACGGCTATGATATGCATGACGCCTGAAGACTT
GGAAAATTATCGTTTGACCAAGGCTGGTGTCGAGGTGCCCCAGGATAATGGG------
GATGAGGTTAACAAACGACTGAAGACCAAGGTGAATCCTTCGACACATATGTATACGCATTGCGA
GATCCACCCTAGCATGCTCCTGGGTATTTGCGCCAGTATCATTCCCTTCCCGGATCACAATCAAGTA
CCCCTGAACGCGTCCCCCAAACCCTTGATTTACTGCCCCTCATACACTCAAGACATGATT---
ATATCCCCAAGTTATCAACACGATGATATCCTCGAATAGAACTGTAATAATGGAAGATAAGAAAGC
TGACCATAT---TTCATCTCAATAGGTTCACCTCCAGACCGGCCAATGTGTAAGTAACAACAG-------
GACTACCGACGAGATATCGCGTCTGGTTATAGCGATGCTCACACGAAGATAATAGGGTAACCAAAT
TGGTGCTGCTTTCTGGCAGACCATCTCCAGCGAGCACGGTCTCGACAGCAATGGCGTGTACGTATT
CGACTCGTCAATTCTCGTGCCAAGAGCACAAACTAACGACCAATAAACAGCTACAATGGAACCTCT
GAGCTACAGCTCGAGCGTATGAGCGTCTACTTTAACGAGGTACGGGGCACATA-----
ACCCATATACGGAAGAGATCAGTTACTAATCACCCCAACATGCACAGGCATCTGGTAACAAATACG
TTCCTCGCGCCGTCCTCGTCGATCTCGAACCCGGTACCATGGACGCCGTTCGCGCTGGTCCCTTTGG
TCAACTTTTCCGTCCCGACAACTTCGTTTTCGGTCAGTCCGGTGCTGGCAACAACTGGGCCAAGGGT

CACTACACTGAGGGTGCCGAGTTGGTCGACCAGGTTCTCGATGTCGTTCGTCGCGAGGCTGAGGCT
TGCGACTGCCTCCAGGGTTTCCAGATTACTCACTCCCTCGGTGGTGGTACTGGTGCTGGTATGGGTA
CCCTGCTGATCTCCAAGATCCGCGAAGAGTTCCCAGACCGCATGATGGCCACTTTCTCGGTCGTCCC
CTCTCCTAAGGTCTCTGACACTGTCGTTGAGCCTTATAACGCTACCTTATCCGTCCACCAGCTGGTC
GAAAACTCTGACGAGACCTTTTGCATTGATAACGAGGCTCTCTACGATATCTGCATGCGTACTCTCA
AGTTATCTAACCCTTCGTATGGTGACCTGAACTACCTGGTCTCTGCTGTCATGTCCGGCGTCACCAC
TTGCTTACGATTCCCTGGTCAGCTGAACTCCGACCTGCGCAAGCTCGCCGTGAACATGGTTCCGTTC
CCTCGTCTCCACTTCTTCATGGTTGGATTCGCTCCTCTGACCAGCCGTGGCGCTCACTCTTTCCGTGC
CGTCACCGTTCCCGAATTAACTCAGCAGATGTTTGACCCCAAGAACATGATGGCCGCCTCTGATTTC
CGTAATGGTCGCTACCTAACGTGCTCTGCCATCTTGTAAGACACTCTTTTTTAAAAGCGTATTGCTG
TTGATATACTAATTTGATCTTTTTAGCCGTGGCAAGGTTACTG

>Hypoxylon_macrocarpum_CBS119012

-CATTACAGAGTTACAAAAACTCCCAACCCTTTGTGAA-CTTACCACTGTTGCCTCGGCGTT-
GGCGCGGCTACCCTATAGTTACCCTG--------------TAGGCAACTACCCTGTACCTATCTCGT-
AGTTCGC--
TTAGGCCCGTCGGAGGACCACTGAACTCTTGTTTTACTGTGAATATCTGAATGCTTCAACTAAATA
AGTTAAAACTTTCAACAACGGATCTCTTGGTTCTGGCATCGATGAAGAACGCAGCGAAATGCGATA
AGTAATGTGAATTGCAGAATTCAGTGAATCATCGAATCTTTGAACGCACATTGCGCCCATTAGTAT
TCTAGTGGGCATGCCTATTCGAGCGTCATTTCAACCCTTAAGCCCCTGTTGCTTAGCGTTGGGAGAC
TGAGCCCCGGGGCCCAGCTCCTTAAAAGTAGTGGCGATGTCGTGGCACATCCTAAGCGTAGTAATT
CTTCTCGCTTCTGTGATTGTCGCGGCTATCTGCCGTAAAACCCCCTATATTTCTAGTGGTTGACCTCG
GATTAGGTAGGAATACCCGCTGAACTTAAGCATATGAAGCGGCAACAGCTCAAA-
TTTGAAATCTGGCC----CTC-----GTGGTCCGAGTTGTAATTTGTAGAGGATGCTTTTGGTGCGGT-
GCCTTCCGAGTTCCCTGGAACGGGACGCCATAGAGGGTGAGAGCCCCGTACGGTTGGA-
CGCCTACCCTATATATAGCTCCTTCGACGAGTCGAGTAGTTTGGGAATGCTGCTCTAAATGGGAGG
TAAATTTCTTCTAAAGCTAAATACCGGCCAGAGACC-
GATAGCGCACAAGTAGAGTGATCGAAAGATGAAAAGTACTTTGAAAAGAGGGTTAAATAGCACGT
GAAATTGTTGAAAGGGAAGCG-TTTGCGACCAGACTTTTTCCAGGCGGATCATCCGGTGTTC--
TCACCGGTGCACTTCGCCTGG--CTTAGGCCAGCATCGGTTTC---
CTTAGGGGGATAAAGGCCTGGGGAACGTAGCTC----TCTAGGGAGTGTT-
ATAGCCCCTCGCGTAATACCCCTCG-GGGGACCGAGGATCGCGCT-CT----
GCAAGGATGCTGGCGTAATGGTCGTCAACGACCCGTCTTGAAACACGGACCAAGGAGTCGAACAT
TTGTGCAAGTGTTTGGGTG--TTAAACCCTCACGCGTAATGAAAGTGAACGGAGGTGAGAGCCCTT--
--AC----GGGTGCATCATCGACCGATCCTGATG-
TCTTCGGATGGATTTGAGTAAGAGCATAACTGTTCGGACCCGAAAGATGGTGAACTATGCGTGGAT
AGGGTGAAGCCAGAGGAAACTCTGGTGGAGGCTCGCAGCGGTTCTGACGTGCAAATCGATCGTCA
AATCTGCGCATGGGGGCGAAAGACTTATCGA-
ACCATCTAGTAGCTGGTTACCGCCGAAGTTTCCCTCAGGATAGCAGTGT--TG-
TATTCAGTTTTATGAGGTAAAGCGAATGATTAGGGACTCGGGGGCGCTATTTAGCCTTCATCCATTC
TCAAACTTTAAATATGTAAGAAGCCCTTGTTGCTTAATTGAACGTGGGCATTCGAATGTACCAACA
CTAGTGGGCCATTTTTGGTAAGCAGAACTGGCGATGCGGGATGAACCGAACGCGGGGTTAAGGTG
CCAGAGTGGACGCTCATCAGACACCACAAAAGGTGTTAGTACATCTTGACAGCAGGACGGTGGCC
ATGGAAGTCGGAATCCGCTAAGGACTGTGTAACAACTCACCTGCCGAATGTACTAGCCCTGAAAAT
GGATGGCGCTCAAGCGTCT-CACCCATACCTCGCCCTTAGGGTAGAAACGATGCCCTAAGG----------
----

CCCCTGAACGCGTCCCCCAAACCCTTGGTTTACTGCCCCTCATACACTCAAAACATGATTAT-
ATATCCCCAAGTTATCAACACGATGTTATCCTCAAATAAAACAAGAATAATGGAAGACAAGAAAG
CTAACCATATTACTTCATCTCAATAGGTTCACCTCCAGACCGGCCAATGTGTAAGTAACAACAA-----
--

GACTACCGACGAAATATCGCGTCTATTTATAGCGAGACTTACACGAAGATAATAGGGTAACCAAAT
TGGTGCTGCTTTCTGGCAGACCATCTCCAGCGAGCACGGTCTCGACAGCAATGGCGTGTATGTATT
CGACTCGTCAATTCTCGTGCCAAGAGCACAAACTAACCACCAATAAACAGCTACAATGGAACGTCC
GAGCTACAGCTCGAGCGTATGAGCGTCTACTTTAACGAGGTACGGGGCACATA-----
ACCCATATACGGAAGAGATCAGTTACTAATCACCCCAACATGCACAGGCATCTGGTAACAAATACG
TTCCTCGCGCCGTCCTCGTCGATCTCGAACCCGGTACCATGGACGCCGTTCGCGCCGGTCCCTTCGG
TCAACTTTTCCGTCCCGACAACTTCGTCTTCGGTCAGTCCGGTGCTGGCAACAACTGGGCCAAGGGT
CACTACACTGAGGGTGCCGAGTTGGTCGACCAGGTTCTCGATGTCGTTCGTCGCGAGGCTGAGGCT
TGCGATTGCCTCCAGGGTTTCCAGATTACTCACTCCCTCGGTGGTGGTACTGGTGCTGGTATGGGTA
CCCTGCTGATCTCCAAGATCCGCGAAGAGTTCCCAGACCGTATGATGGCCACTTTCTCGGTTGTCCC
CTCTCCTAAGGTCTCCGACACTGTCGTTGAGCCTTATAACGCTACCTTATCCGTCCACCAGCTGGTC
GAAAACTCTGACGAGACCTTTTGCATTGATAACGAGGCTCTCTACGATATCTGCATGCGTACTCTCA
AGTTATCTAACCCTTCATWTGGTGACCTGAACTACCTGGTCTCTGCTGTCATGTCCGGCGTCACCAC
TTGCTTACGATTCCCTGGTCAGCTGAACTCCGACCTGCGCAAGCTCGCCGTGAACATGGTTCCGTTC
CCTCGTCTCCACTTCTTCATGGTTGGATTCGCTCCTCTAACCAGCCGTGGCGCTCACTCTTTCCGTGC
CGTCACCGTTCCCGAATTAACTCAGCAGATGTTTGACCCCAAGAACATGATGGCCGCCTCTGACTT
CCGTAATGGTCGCTACCTAACGTGCTCTGCCATCTTGTAAGATACCCTTTTCTACAAACGTGTTACT
ATTGATATACTAATTTCATCTTTTTAGCCGTGGCAAGGTTACTG

>Hypoxylon_larissae_FCATAS844

-CATTACAGAGTTATAAAAACTCCCAACCCTATGTGAACTTTACCACTGTTGCCTCGGCGCG-
TGCGCGGCTACCCGGGAGCTACCCTGGAGTTGACTACCCTGTAGTCACCTACCCTGGAGCTACCCT
GT-AGTCCGC-
GTTAGGCCCGTCAGGGGACCACCAAATTCTTGTTTTTACTGTGTATTTCTGAATGCTTCAATTAAAT
AAGTTAAAACTTTCAACAACGGATCTCTTGGTTCTGGCATCGATGAAGAACGCAGCGAAATGCGAT
AAGTAATGTGAATTGCAGAATTCAGTGAATCATCGAATCTTTGAACGCACATTGCGCCCATTAGCA
TTCTATTGGGCATGCCTATTCGAGCGTCATTTCAACCCTTAAGCCTTTGTTGCTTAGCGTTGGGAAT
CTACGTT--
ACAGCGTAGTTCCTTAAAGTCAGTGGCGGAGTTATAGCACACTCTAAGCGTAGTAAACTTCCTCGC
TTCTGTAGTTGCCCTAGCTTTCGGCCGTAAAACCCCCC-TAATTCAAAGGT--
GACCTCGGATTAGGTAGAATCCC------------------TGAAGCGGCAACAGCTCAAA-
TTTGAAATCTGGCC----CTA-----GCGGTCCGAGTTGTAATTTGTAGAGGATGCTTTTGGTGAGGT-
GCCTTCTGAGTTCCCTGGAACGGGACGCCAGAGAGGGTGAGAGCCCCGTACGGTCGGC-
CGCCAAACCTGTATATAGCTCCTTCGACGAGTCGAGTAGTTTGGGAATGCTGCTCTAAATGGGAGG
TAAATTTCTTCTAAAGCTAAATACCGGCCAGAGACC-
GATAGCGCACAAGTAGAGTGATCGAAAGATGAAAAGCACTTTGAAAAGAGGGTTAAATAGCACGT
GAAATTGTTGAAAGGGAAGCG-TTTGCGACCAGACTTTTTCCTGGCGGATCATCCGGTGCTC--
TCACCGGTGCACTTCGCCAGG--TCTAGGCCAGCATCGGTTTC---
CTTAGGGGGATAAAGGCCTGGGGAACGTAGCTC----TTTAGGGAGTGTT-
ATAGCCCCTGGCGCAATGCCCTTCG-GGGGACCGAGGATCGCGCT-CT----
GCAAGGATGCTGGCGTAATGGTCGTCAACGACCCGTCTTGAAACACGGACCAAGGAGTCGAACAT
TTGTGCGAGTGTTTGGGTG--TTAAACCCTCACGCGTAATGAAAGTGAACGGAGGTGAGAGCCCT----
-A-----GGGTGCATCATCGACCGATCCTGATG-
TCTTCGGATGGATTTGAGTAAGAGCATAACTGTTCGGACCCGAAAGATGGTGAACTATGCGTGGAT
AGGGTGAAGCCAGAGGAAACTCTGGTGGAGGCTCGCAGCGGTTCTGACGTGCAAATCGATCGTCA
AATCTGCGCATGGGGGCGAAAGACTTATCGAACCCAT---

ACGTTGTATCGACCAGAACAGAAGGTTCCAGATCGAGCTCGCTGCCAAGCCTGCAATCGTCACCAA
CGGACTGAAGTACTCCCTTGCCACGGGTAACTGGGGTGATCAGAAGAAGGCCATGAGCTCGACAG
CAGGTGTCTCTCAGGTGCTCAACCGTTACACAT------TCGCGTCGA---
CCCTCTCCCACTTGAGAAGAACGAACACGCCCATCGGACGAGATGGCAAGCTGGCCAAACCCCGA
CAGCTTCACAACACCCACTGGGGTCTCGTCTGTCCGGCCGAGACGCCCGAAGGCCAGGCCTGTGGA
CTTGTGAAGAACTTGTCGCTGATGTGTTCCATCAGCGTGGGTACAGCAGCGGAGCCCATCATCGAC
TATATGATCACCAGGAATATGGAAGTTCTCGAGGAGTATGAGCCCATGAGATATCCCAATGCCACA
AAGATATTCCTGAATGGTTCGTGGTGCGGTATACACCAAGACCCCAAGGCTCTCGTTCGAGATGTT

CAGCAGCTGCGTCGCGCCAACCAGATCCCCTACGAGGTTTCGCTTGTCCGCGACATTCGTGATCGC
GAGTTCAAGATCTTTTCCGATGCTGGTCGTGTCATGCGCCCCTTGTTTGTCGTCCAGCAGGAGGAT--
----
GTTCCCGAGACTGGTGCCCAGAAGGGTACTCTGGCTCTCACCAAGGACATGATACAGAGACTAGA
GGCAGACGTCGACCTCGATCCCGAGAGCGACGACTACTTTGGTTGGCAGGGTCTGGTCAACGAAG
GCGTCATCGAGTATCTCGACGCAGAGGAAGAAGAGACGACCATGATTTGCATGACACCTGAAGAC
TTGGAGAATTACCGATTGACTAAGGCTGGGTTTGATGTTTCTCAGGACAATGGA------
GACGAGATTAACAAGCGTCTCAAGACCAAAGTCAATCCCACCACACACATGTACACTCATTGCGAG
ATTCATCCTAGCATGTTGCTGGGTATCTGCGCAAGTATCATCCCGTTTCCGGATCACAATCAGGTAC
CCCTGAACGCGTCCCTCAACCCCCTGATTTCCTGCCCCTCACGCACAAAAAAC---
ACCACAACACTTCATAGCTATATATATTGAACTGTTGTCGGATTGTACAACGATAGATGAAGAAGC
TGAAGCTAACCACCTCTTTTTGCCACTATAGGTTCACCTCCAGACCGGCCAGTGCGTAAGTATC------
----ACGACTACCAACGA-
ATATCGCGATGAGACATTGCGGGGCTCACATAAAGTCGATAGGGTAACCAAATTGGTGCTGCCTTC
TGGCAAACTATCTCTGGCGAGCACGGCCTCGATAGCAATGGCGTGTACGTAATTAATTCGCCAATT
CTACCGAGAGAATATTCAACTAATCACCAATAAATAGCTACAACGGAACTTCCGAGCTTCAGCTCG
AGCGCATGAGCGTCTACTTCAACGAGGTATGCACACAGAAGAATTGGGAAAAGATGCGAAAGATT
GGTTACTAATTACCACA------
ACAGGCTTCCGGCAACAAGTATGTTCCTCGCGCTGTCCTCGTCGATCTCGAACCCGGCACCATGGA
TGCCGTCCGTGCTGGTCCCTTCGGTCAGCTCTTCCGACCCGACAACTTCGTCTTCGGCCAGTCTGGT
GCTGGCAACAATTGGGCCAAGGGTCACTACACTGAGGGTGCCGAGCTTGTCGACAATGTCCTTGAC
GTCGTTCGTCGCGAGGCTGAGGGCTGCGACTGCCTTCAGGGCTTCCAGATCACGCACTCTCTCGGT
GGTGGTACCGGTGCCGGTATGGGTACTCTGCTGATCTCCAAGATCCGCGAGGAGTTCCCCGACCGC
ATGATGGCCACATTCTCCGTTGTGCCTTCTCCCAAGGTTTCCGACACCGTCGTCGAGCCCTACAATG
CCACCCTCTCCGTCCACCAGCTGGTCGAGAACTCGGACGAGACCTTCTGTATTGACAACGAGGCTC
TGTACGACATCTGCATGCGTACTCTTAAGCTATCCAACCCCTCCTACGGTGACCTGAACCACCTAGT
TTCAGCTGTCATGTCCGGTGTCACTACTTGCCTGCGCTTCCCCGGTCAGCTGAACTCTGACCTGCGC
AAGCTTGCCGTCAACATGGTTCCCTTCCCTCGTCTGCACTTTTTCATGGTTGGCTTCGCTCCTCTGAC
GAGTCGTGGCGCCCACTCTTTCCGTGCCGTCACCGTTCCCGAGTTGACTCAGCAGATGTTCGACCCC
AAGAACATGATGGCTGCTTCCGACTTCCGTAACGGTCGCTACCTGACGTGCTCTGCCATCTTGTAAG
ATTCCTACTCCCACCAGACTATTTTTGCATATTTGCTAATTCGCATTCTCCAGCCGTGGCAAGGTTTC
CA

>Hypoxylon_medogense_FCATAS4061

-CATTACAGAGTTAC-TAAACTCCCAACCCTTTGTGAACATTACCACTGTTTCCTCGGCGTG-
GTCTCGGCCTCAG--CGG-
TCGAGGCCCGCCGAAGGACCGCTAAACTCTTGTTT--
CTCGAGTATGTCTGAATGCTTCAAAAAAATAAGTTAAAACTTTCAACAACGGATCTCTTGGTTCTG
GCATCGATGAAGAACGCAGCGAAATGCGATAAGTAATGTGAATTGCAGAATTCAGTGAATCATCG
AATCTTTGAACGCACATTGCGCCCATTAGTATTCTAGTGGGCATGCCTATTCGAGCGTCATTTCAAC
CCTTAAG-
CCCTGTAGCTTAGCGTTAGGAGCCTGCGCCTTGGGGCGCAGCTCCCTAAAGTTAGTGGCGGAGTTA
GGGCACACTCTGAGCGTAGTAATTATTCTCGCTTCTGTGGTGGCTCTGGCGACCTGCCGTAAAACCC
CCTATAACTCTAGTGGTTGACCTCGGATAAG---------------------------TGAAGCGGCAACAGCTCAAA-
TTTGAAATCTGGCC----CCA-----GCGGTCCGAGTTGTAATTTGTAGAGGATGCTTTTGGCGCGGT-
GCCTTCCGAGTTCCCTGGAACGGGACGCCGGAGAGGGTGAGAGCCCCGTACGGTTGGC-
CGCCTAGCCTATCTATAGCTCCTTCGACGAGTCGAGTAGTTTGGGAATGCTGCTCTAAATGGGAGG
TAAATTTCTTCTAAAGCTAAATACCGGCCAGAGACC-
GATAGCGCACAAGTAGAGTGATCGAAAGATGAAAAGCACTTTGAAAAGAGGGTTAAATAGCACGT
GAAATTGTTGAAAGGGAAGCG-TTTGCGACCAGACTTTCTCCTGGCGGATCATCCGGTGTTC--
TCACCGGTGCACTTCGCCTGG--TTTAGGCCAGCATCGGTTTC---
CTTAGGGGGATAAAGGCCCTGGGCACGTAGCTC----TTCAGGGAGTGTT-
ATAGCCCTTCGCGTAATCCCCCTCG-GGGGACCGAGGATCGCGCT-CT----
GCAAGGATGCTGGCGTAATGGTCGTCAACGACCCGTCTTGAAACACGGACCAAGGAGTCGAACAT
TTGTGCGAGTGTTTGGGTG--TCAAACCCTCACGCGTAATGAAAGTGAACGGAGGTGAGAGCCCCT--
---CG---GGGTGCATCATCGACCGATCCTGATG-
TCTTCGGATGGATTTGAGTAAGAGCATAACTGTTCGGACCCGAAAGATGGTGAACTATGCGTGGAT

AGGGTGAAGCCAGAGGAAACTCTGGTGGAGGCTCGCAGCGGTTCTGACGTGCAAATCGATCGTCA
AATCTGCGCATGGGGGCGAAAGACTTATCGA-ACC--

ACGCTGCATCGACCAGTCCAAGCGATTTCAAATCGAGCTTGCTGCTAAACCCGCCATCATTACGGG
CTCGTTGAAGTACTCCCTCGCCACAGGTAACTGGGGTGACCAAAAGAAGGCGATGAGCTCTACCGC
CGGTGTCTCCCAGGTTCTCAACCGCTACACGT------TTGCTTCGA---
CTCTGTCTCACTTGAGGAGAACGAACACACCTATTGGAAGAGACGGCAAATTGGCCAAGCCTCGGC
AATTGCACAACACTCATTGGGGTCTTGTCTGCCCGGCCGAGACGCCCGAAGGCCAGGCCTGCGGGC
TGGTGAAGAATCTGTCGCTGATGTGCTCCATCAGTGTGGGTACATCGACGGACCCTATCGTCGAAT
ACATGATAACTAGGAATATGGAAGTGCTCGAGGAGTACGAGCCGATGAGATATCCGAATGCGACC
AAGATCTTCTTGAACGGATCCTGGATCGGTGTCCATCAGGACCCAAAGTCCCTCGTCCGGGATGTT
CAGGCGTTGCGTAGGAGCAACCAGATTCCGGCCGAGGTGTCCTTGGTCCGCGACATCCGTGATCGT
GAGTTCAAGATATTCTCAGACGCAGGTCGTGTCATGCGCCCCTTGTTCGTCGTACAGCAAGAGGAT-
----
AAGGAGGATGTCAAGCAGGGCTCTCTAGCTCTCACCAAAGAGATGATACAGAGACTCGAAGCGGA
CGTCGACCTGGACCCCGGAAGCGATGGTTATTATGGCTGGCAGGGCCTAGTCGACGATGGCGTTAT
CGAGTATCTCGATGCGGAGGAGGAGGAAACCGCCATGATCTGCATGACTCCCGAGGACTTGGAGA
ATTATCGGATGACCAAGGCCGGCATTGAGGTTAACCAAGACAATGGG------
GATGAGATCAATAAACGCCTCAAGACCAAGGTGAATCCTACGACGCACATGTACACTCACTGCGA
GATCCATCCCAGCATGCTTCTGGGCATCTGCGCCAGTATCATTCCATTCCCGGATCACAACCA----
CCCCTGAACGCGTCCCTGAACCTCCTGATTCCGTGCCCCTCACGCACACAAAACCCAAAAACAATA
TCCCACAGCAAGCTATGTCGTGCGATCATCGAATTATACGACGATA-
AGATGGAAATCGATGCTAACCATATCTTTTAATCAATATAGGTTCACCTTCAGACCGGCCAGTGCG
TAAGTAGA-CACC----GACGATCACCAACCAA-
GATAACGATAAAATATTGCGGGGCTCACACGATGCTGGTAGGGTAACCAAATTGGTGCCGCCTTCT
GGCAGACCATCTCTGGCGAGCACGGTCTCGACAGCAATGGCGTGTACGTATATAATTCGTCAATCC
ACCTCTTGAGAATATCAGCTAACGGCCAATAAATAGCTACAACGGTACTTCCGAGCTCCAGCTTGA
GCGCATGAGCGTCTACTTCAACGAGGTACGAACCTATAGAATCTACGAATAGATGGTGAGAGACT
GCTAATCACCACCATCACACGCACAGGCTTCCGGCAACAAGTATGTTCCTCGCGCCGTCCTCGTCG
ATCTCGAGCCCGGTACCATGGATGCCGTCCGTGCTGGTCCCTTTGGCCAGCTCTTCCGACCCGACAA
CTTCGTCTTCGGCCAGTCCGGTGCCGGAAACAACTGGGCCAAGGGTCACTACACTGAGGGTGCTGA
GCTTGTCGACAACGTCCTCGACGTCGTCCGTCGCGAGGCTGAGGGCTGCGACTGCCTCCAGGGCTT
CCAGATCACCCACTCGCTCGGTGGTGGTACCGGTGCTGGTATGGGTACCCTGCTCATCTCCAAGAT
CCGCGAGGAGTTCCCTGATCGCATGATGGCCACTTTCTCCGTTGTCCCCTCCCCCAAGGTCTCTGAC
ACCGTCGTCGAGCCTTACAACGCCACTCTCTCGGTCCACCAGCTGGTCGAGAACTCGGATGAGACC
TTCTGCATCGACAACGAGGCTCTTTACGACATCTGTATGCGTACCCTCAAGTTGTCCAACCCCTCGT
ACGGTGATCTGAACCACCTTGTCTCCGCCGTCATGTCTGGCGTCACTACCTGCCTGCGCTTCCCGGG
CCAGCTCAACTCTGACCTGCGCAAGCTTGCCGTCAACATGGTTCCCTTCCCTCGTCTGCATTTCTTC
ATGGTCGGCTTCGCTCCTCTGACCAGCCGTGGTGCCCACTCCTTCCGCGCCGTCACCGTGCCCGAGT
TGACTCAGCAGATGTTCGACCCCAAGAACATGATGGCTGCCTCTGACTTCCGCAACGGTCGCTACC
TGACCTGCTCTGCCATCTTGTAAGATGCCCTTCTCTTTTATTTATACGTGTCTGTTTTGCTAACTTGT
A-TTCCTAGCCGTGGCAAGGTCTCGA

>Hypoxylon_erythrostroma_MUCL53759

---TTACAGAGTTAC-CAAACTCCAAACCCTTTGTGAACCTTACCACTGTTTCCTCGGCGAG-
GTATCAGGCCCTCAGCGGC---CGAGAACCCGCC-
GAAGACCACTAAACTCGTGTTT--
GTTTAGCGTATCTGAATGCTTCAAACAAATAAGTTAAAACTTTCAACAACGGATCTCTTGGTTCTGG
CATCGATGAAGAACGCAGCGAAATGCGATAAGTAATGTGAATTGCAGAATTCAGTGAATCATCGA
ATCTTTGAACGCACATTGCGCCCATTAGTATTCTAGTGGGCATGCCTATTCGAGCGTCATTTCAACC
CTTAAG-
CCCTGTTGCTTAGCGTTGGGANTCTGCGTCTTAGGGCGCAGCTCCCTAAAGTTAGTGGCGGAGTCG
GAGCACACTCTGAGCGTAGTAATTGTTCTCGCTTCTGTGGTGGCTTTGGCGACCTGCCGTAAAA------
--TGAAGCGGCAACAGCTCAAA-TTTGAAATCTGGCC----
CTA-----GCGGTCCGAGTTGTAATTTGTAGAGGATGCTTTTGGCGCGGT-

GCCTTCTGAGTTCCCTGGAACGGGACGCCAGAGAGGGTGAGAGCCCCGTACGGTTGGC-
CGCCTAGCCTTTATACAGCTCCTTCGACGAGTCGAGTAGTTTGGGAATGCTGCTCTAAATGGGAGG
TAAATTTCTTCTAAAGCTAAATACCGGCCAGAGACC-
GATAGCGCACAAGTAGAGTGATCGAAAGATGAAAAGTACTTTGAAAAGAGGGTTAAATAGCACGT
GAAATTGTTGAAAGGGAAGCG-TTTGCGACCAGACCTTCTCCTGGCGGATCATCCGGTGTTC--
TCACCGGTGCACTTCGCCTGG--TTTAGGCCAGCATCGGTTCT---
CTTAGGGGGATAAAGGTCTTGGGCACGTAGCTC----TTTCGGGAGTGTT-
ATAGCCCCTGGCGTAATACCCTTCG-GGGGACCGAGGACCGCGCT-CT----
GCAAGGATGCTGGCGTAATGGTCGTCAACGACCCGTCTTGAAACACGGACCAAGGAGTCGAACAT
TTGTGCGAGTGTTTGGGTG--TTAAACCCTCACGCGTAATGAAAGTGAACGGAGGTGAGAGCCCCT--
--A-G---GGGTGCATCATCGACCGATCCTGATG-
TCTTCGGATGGATTTGAGTAAGAGCATAACTGTTCGGACCCGAAAGATGGTGAACTATGCGTGGAT
AGGGTGAAGCCAGAGGAAACTCTGGTGGAGGCTCGCAGCGGTTCTGACGTGCAAATCGATCGTCA
AATCTGCGCATGGGGGCGAAAGACTTATCGA-
ACCATCTAGTAGCTGGTTACCGCCGAAGTTTCCCTCAGGATAGCAGTGT--TG-
TTTTCAGTTTTATGAGGTAAAGCGAATGATTAGGGACTCGGGGGCGCTATATTGCCTTCATCCATTC
TCAAACTTTAAATATGTAAGAAGCCCTTGTTGCTTAGTTGAACGTGGGCATTCGAATGTATCAACA
CTAGTGGGCCATTTTTGGTAAGCAGAACTGGCGATGCGGGATGAACCGAACGCGGGGTTAAGGTG
CCAGAGTAAACGCTCATCAGACACCACAAAAGGTGTTAATACATCCTGACAGCAGGACGGTGGCC
ATGGAAGTCGGAATCCGCTAAGGACTGTGTAACAACTCACCTGCCGAATGTATTAGCCCTGAAAAT
GGATGGCGCTCAAGCGTTT-
CACCAATACCTCGCCCTTAGGGTAGAAACGATGCCCTAAGGACGCTGCATCGACCAGTCCAAGCGT
TTCCAGATCGAGCTTGCTGCTAAGCCTGCTATCGTCACGAATGGGCTGAAGTACTCCCTTGCCACA
GGTAACTGGGGTGACCAAAAGAAGGCAATGAGCTCGACCGCTGGTGTGTCCCAAGTTCTCAACCGT
TACACGT------TCGCATCAA---
CCCTGTCACATTTGAGGCGAACGAACACTCCCATCGGACGAGACGGTAAACTGGCCAAGCCTCGGC
AATTGCACAACACCCATTGGGGCCTAGTCTGCCCGGCCGAGACACCCGAAGGTCAGGCCTGCGGG
CTGGTAAAGAATTTGTCGTTGATGTGCTCCATCAGCGTGGGCACATCGACAGATCCCATTGTCGAC
TATATGATAACCAGGAACATGGAAGTGCTTGAGGAGTACGAGCCGATGCGATACCCCAACGCGAC
CAAGATTTTCTTAAACGGATCTTGGATCGGTGTCCATCAAGACCCCAAGTCACTCGTCCGGGATGT
CCAGGCATTGCGTAGGAGCAACCAGATTCCCGCCGAGGTGTCTTTGGTCCATGATATCCGTGATCG
TGAATTCAAGATCTTCTCAGACGCCGGCCGTGTCATGCGCCCCTTGTTCGTCGTACAGCAAGAGGA
T--
ACGGAACAAGTGAAGAAGGGCTTTCTAGCCCTTAACAAAGATATGATACAACGGCTCGAGGCGGA
TTCCGAAACAGACCCTGACAGTGATGGCTATTATGGTTGGCAGGGTCTGGTCGACGATGGCGTCAT
CGAGTATCTCGACGCAGAGGAGGAAGAGACGGCCATGATATGCATGACTCCTGAAGACTTAGAGA
CATATCGAATGACAAAGGCCGGCCAAGCTATTGCGGAAGATCACACA------
GAGGAAACCAACAAGCGCCTCAAGACAAGGATCAATCCAACGACGCACATGTACACCCATTGCGA
GATCCACCCCAGCATGCTTCTGGGCATATGCGCGAGTATTATTCCGTTCCCGGATCATAATCAGGTA

>Hypoxylon_jianfengense_FCATAS845

-CATTACTGAGTTAACAAAACTCCCAACCCTTTGTGAA-CTTACCACTGTTGCCTCGGCGTC-
GGCGCAGCTACCCTGTAGTAACCCTGTAGCTGGCTACCCTGTAGACGGCTACCCGGAACCTACCTC
GT-AGTGCGC-
TCAAGGCCCGCCGGAGGACCATCTAACTCTTGTTTAAACTGTGAATCTCTGAATGCTTCAACTAAAT
AAGTTAAAACTTTCAACAACGGATCTCTTGGTTCTGGCATCGATGAAGAACGCAGCGAAATGCGAT

AAGTAATGTGAATTGCAGAATTCAGTGAATCATCGAATCTTTGAACGCACATTGCGCCCATTAGTA
TTCTAGTGGGCATGCCTATTCGAGCGTCATTTCAACCCTTAAGCCCCTGTTGCTTAGCGTTGGGAGA
CCGAGCCTCCGGGCCTGGCTCCTTAAAATTAGTGGCGATGTTACAGCACATCCTAAGCGTAGTAGC
TTGTCTCGCTTCTGTGGTGGCTGTGGTGGTCTGCCGTTAAACCCCT----
TTATCAAGTGGTGACCTCGGATAGG---TAGATGCCCGT-------------TGAAGCGGCAACAGCTCAAA-
TTTGAAATCTGGCC----CTA-----GTGGTCCGAGTTGTAATTTGTAGAGGATGCTTTAGGTGCGGT-
GCCTTCTGAGTTCCCTGGAACGGGACGCCAGAGAGGGTGAGAGCCCCGTACGGTTGGA-
CGCCTAGCCTATGTATAGCTCCTTCGACGAGTCGAGTAGTTTGGGAATGCTGCTCTAAATGGGAGG
TAAATTTCTTCTAAAGCTAAATACCGGCCAGAGACC-
GATAGCGCACAAGTAGAGTGATCGAAAGATGAAAAGTACTTTGAAAAGAGGGTTAAATAGCACGT
GAAATTGTTGAAAGGGAAGCG-TTTGCGACCAGACTTTTTCCAGGGGGATCATCCGGTGTTT--
TCGTCGGTGCACTTCCCCTGG--TTGAGGCCAGCATCGGTTTC---
CTTAGGGGGATAAAGGCTTGGGGAACGTAGCTC----TTTCGGGAGTGTT-
ATAGCCCCTCGCGTAATACCCCTCG-GGGGACCGAGGATCGCGCT-CT----
GCAAGGATGCTGGCGTAATGGTCGTCAACGACCCGTCTTGAAACACGGACCAAGGAGTCGAACAT
TTGTGCGAGTGTTTGGGTG--TTAAACCCTCACGCGTAATGAAAGTGAACGGAGGTGAGAGCCCTT--
--AC----GGGTGCATCATCGACCGATCCTGATG-
TCTTCGGATGGATTTGAGTAAGAGCATAACTGTTCGGACCCGAAAGATGGTGAACTATGCGTGGAT
AGGGTGAAGCCAGAGGAAACTCTGGTGGAGGCTCGCAGCGGTTCTGACGTGCAAATCGATCGTCA
AATCTGCGCATGGGGGCGAAAGACTTATCGA-ACCAT---

ACGCTGCATTGACCAGAACAGACGTTTCCAGATCGAGCTGGCAGCCAAGCCTTCCATCGTCACCAA
CGGGCTGAAGTATTCCCTTGCCACGGGCAACTGGGGCGACCAGAAGAAGGCGATGAGCTCAACCG
CCGGTGTATCGCAGGTGTTAAACAGATATACGT------TCGCGTCAA---
CCTTGTCACATTTACGACGAACCAACACCCCTATCGGAAGAGATGGGAAGCTTGCGAAACCCCGAC
AGCTTCACAACACTCATTGGGGTCTGGTCTGTCCAGCCGAAACACCCGAAGGCCAGGCTTGTGGGT
TGGTGAAGAATCTGTCGCTGATGTGTTCTATCAGCGTGGGTACATCGACGGATCCTATCGTGGACT
ATATGATTACTAGGAATATGGAAGTCCTGGAGGAATACGAGCCGATGAGATACCCCAACGCCACC
AAAATTTTCCTCAATGGTTCTTGGATTGGCGTACACCAAGACCCCAAGTCTCTGGTTAGAGACGTTC
AGCAGCTTCGTCGAGCCAACCAGATCCCCTCGGAAGTGTCGCTGGTTCGCGATATCCGTGATCGCG
AATTCAAGATTTTCTCGGACGCTGGTCGTGTCATGCGCCCCTTGTTCGTCGTGCAACAAGATGAC----
----
GTTCCGGAAGCAGGTATCGTCAAGGGCACATTGGCCCTCAACAAGGACATGATCCAACGGCTAGA
AGCCGACGTCGACTTAGACCCCGACAGTGATGAGTACTTTGGTTGGCAGGGTCTAGTCAACGAGGG
TGTGATCGAGTACCTTGACGCCGAAGAAGAGGAAACAGCCATGATATGCATGACGCCTGAAGACT
TGGAAAATTACCGGTTGGCCAAGACTGGTGCCGAGGTCCCCCAGGATAATGGG------
GATGAGGTTAATAAACGACTGAAGACCAAGGTCAACCCCTCGACACATATGTACACACACTGCGA
GATCCATCCCAGCATGCTTCTGGGGATCTGCGCCAGCATCATTCCCTTCCCGGACCACAATCAGGT
ACCCCTAAACGCGTCCCTCAAACCCCTCGGGTTCTGCCCCTCATATACACAAACCACGATCATATCC
CCACATGGTCACAAACATGAGATCCTCCTCAAGTTAAACAAGAATGATGGAAGATAAGGAAGCTA
ACCATACATCTTCATCTCAATAGGTTCACCTCCAGACCGGCCAATGCGTAAGTAGCAACAT----
GCCTACTACCCACGATATATCGCGCCTGGCTATAGCAGAACTCACACGATAGTAATAGGGTAACCA
AATTGGTGCTGCTTTCTGGCAGACCATCTCTGGCGAGCACGGTCTCGACAGCAATGGCGTGTACGT
ATCGGAGTGGTCAATTCGCCCGCCAAGAGAGCCAACTAACTACCAATAAACAGCTACAATGGAAC
CTCTGAGCTCCAGCTCGAGCGTATGAGTGTCTACTTTAACGAGGTACGGGGCACATA-----
ACCCATTCACCGAAATGATCAGTTACTAATCACCCCAACATGCACAGGCATCTGGTAACAAGTACG
TTCCTCGCGCCGTCCTCGTCGATCTCGAACCCGGTACCATGGACGCCGTTCGCGCCGGTCCCTTCGG
TCAGCTCTTCCGTCCCGACAACTTCGTCTTCGGCCAGTCCGGTGCTGGAAACAACTGGGCCAAGGG
TCACTACACTGAGGGTGCTGAGTTGGTGGACAACGTTCTCGATGTCGTTCGTCGCGAGGCCGAGGC
TTGCGATTGCCTCCAGGGTTTCCAGATCACCCACTCTCTTGGTGGTGGTACTGGTGCTGGTATGGGT
ACCCTGTTAATTTCCAAGATTCGCGAGGAGTTCCCCGACCGCATGATGGCCACTTTCTCCGTCGTCC
CCTCTCCCAAGGTCTCTGACACTGTCGTCGAGCCTTACAACGCTACACTCTCCGTCCACCAGCTGGT
CGAAAACTCTGACGAGACCTTCTGCATTGACAACGAGGCTCTGTACGACATCTGCATGCGTACTCT
TAAGCTATCTAACCCTTCGTACGGTGACCTGAACTACCTGGTCTCTGCCGTCATGTCCGGCGTTACG
ACTTGCTTACGATTCCCTGGCCAGTTGAACTCTGACCTGCGCAAGCTCGCCGTGAACATGGTTCCTT

TCCCCCGTCTCCACTTCTTCATGGTTGGATTCGCTCCTCTTACCAGCCGTGGTGCTCACTCTTTCCGT
GCCGTCACCGTTCCCGAATTAACTCAACAGATGTTCGACCCCAAGAACATGATGGCCGCCTCTGAC
TTCCGTAACGGCCGTTACCTAACGTGCTCTGCCATCTTGTAAGATTTTTTTCTTTTCT---
ACGCATATTTAATTTACTGACCTTTTCTTCTTAGCCGTGGCAAGGTTACTG

>Annulohypoxylon_michelianum

-CATTAACGAGTTACCAAAACTCCAAACCCTTTGTGAACCTTACC-TAGTTGCCTCGGCGTG-
AGCGCGGTTACCCTGGAGCTACCCTGGAGCGTTTTACCCCGTAAGTGGTCAGGAAGAAGCTACCCT
GG-AACCGGC-
CTACGGCCCGCCGGAGGACCGCTAAACTCTGTCTTTACCACTGTATCTCTGAATTCGTAA-
CTAAATAAGTTAAAACTTTCAACAACGGATCTCTTGGTTCTGGCATCGATGAAGAACGCAGCGAAA
TGCGATAAGTAATGTGAATTGCAGAATTCAGTGAATCATCGAATCTTTGAACGCACATTGCGCCCA
TTAGTATTCTAGTGGGCATGCCTATTCGAGCGTCATTTCGACCCTTAAG-
CCTTGCTGCTTAGCGTTGGGAGTCTACGGCTT-
TGGCGTAGCTCCTTAAAGTCAGTGGCGGAGTTATGGCACACTCTAAGCGTAGTAATACCTCTCGCT
TCTGTAGTTGCCTTAACTGCCTGCCGTAAAACCCCCTATATTTCTAATGGTTGACCTCGGATTAGGT
AGGAATACCCGCTGAACTTAAGCATATGAAGCGGCAACAGCTCAAA-TTTGAAATCTGGCC----CTC-
----GCGGTCCGAGTTGTAATTTGTAGAGGATGCTTTTGGTACGGT-
GCCTTCCGAGTTCCCTGGAACGGGACGCCAGAGAGGGTGAGAGCCCCGTACGGTGGGA-
CACCTATCCTATATATAGCTCCTTCGACGAGTCGAGTAGTTTGGGAATGCTGCTCTAAATGGGAGG
TAAATTTCTTCTAAAGCTAAATACCGGCCAGAGACC-
GATAGCGCACAAGTAGAGTGATCGAAAGATGAAAAGCACTTTGAAAAGAGGGTTAAATAGCACGT
GAAATTGTTGAAAGGGAAGCG-TTTGCGACCAGACCTTTTCCAGGCGGATCATCCGGTGTTC--
TCACCGGTGCACTTCGCCTGG--TTTAGGCCAGCATCGGTTTC---
CTTAGGGGGATAAAGGCCTGGGGAACGTAGCTC----TTTAGGGAGTGTT-
ATAGCCCCTCGCGTAATACCCCTCA-GGGGACCGAGGACCGCGCT-CT----
GCAAGGATGCTGGCGTAATGGTCGTCAACGACCCGTCTTGAAACACGGACCAAGGAGTCGAACAT
TTGTGCGAGTGTTTGGGTG--TTAAACCCTCACGCGTAATGAAAGTGAACGGAGGTGAGAGCCCTT--
--A-G---GGGTGCATCATCGACCGATCCTGATG-
TCTTCGGATGGATTTGAGTAAGAGCATAACTGTTCGGACCCGAAAGATGGTGAACTATGCGTGGAT
AGGGTGAAGCCAGAGGAAACTCTGGTGGAGGCTCGCAGCGGTTCTGACGTGCAAATCGATCGTCA
AATCTGCGCATGGGGGCGAAAGACTTATCGA-ACCAT---

CGATATACCTTCCGCGTCCGACTCC---
TCCTCCTCCATTTGAGACGAACGAACACGCCCATCGGAAGAGACGGAAAGCTAGCCAAGCCGCGG
CAGCTTCACAACACTCATTGGGGTCTGGTCTGTCCGGCAGAGACGCCCGAAGGCCAGGCTTGCGGG
CTGGTGAAGAACTTGTCCCTGATGTGTTCCATCAGCGTTGGTACGTCGACAGATCCCATTGTAGACT
ACATGATTACTAGGAATATGGAAGTCCTCGAGGAATATGAGCCGATGAGATACCCAAACGCCACG
AAGATCTTCCTCAACGGCTCTTGGATCGGCGTGCACCAGGACGCCAAGACCCTCGTCAGAGATGTC
CAGGCGCTTCGCCGGGCCAACCAGATTCCCGCCGAGGTATCGCTGGTTCGTGATATCCGCGACCGC
GAATTCAAGATCTTCTCGGATGCCGGTCGCGTGATGCGTCCCTTGTTCCGCGTACAGCAGGAGGAT-
----

ATCCAGGAGCTGAGCATCGAGAAAGGCACGCTGGCTCTTACCAAGCAGATGATCAAGCGCCTAGA
GACGGACGTCGATTTGGATCCGGAGAGCGAAGGGTACTTCGGCTGGCAAGGCCTGGTCAACGAGG
GTGTTATCGAGTTCCTCGACGCGGAGGAAGAGGAGACCGCCATGATCTGCATGACGCCCGAAGAT
CTAGACATTTACCGCATGACCAAGCTCGGATACGACGTAGCCCAGGACAACGGT------
GACGAGGTTAACAAGCGACTGAAGACCAAAGTGAACCCGTCGACGCACATGTATACCCACTGCGA
AATCCATCCCAGCATGCTCCTAGGCATCTGCGCGAGCATCATCCCGTTCCCGGACCACAACCAGGT
ATCCCTCAATAAGATTTAAAAACCGATTATCGCCTAAACCT--------AAGATC---
TCCAGGATTCCACAACATCCAGAGGGTTACACCAGCATCAAGTTCCACAACGATGAGTTTGTGAAT
CGAAGCTAACCGCA-TATTTCATCTCAATAGGTTCACCTCCAGACCGGCCAATGCGTAAGTACT------
----
ACGATCAACGACGAGATATCGCGCTGGAGTATAGTGGGGCTTACACGAATATCGTAGGGTAACCA
AATTGGTGCTGCTTTCTGGCAAACCATCTCTGGCGAGCACGGTCTCGACAGCAATGGCGTGTAAGT

GTTTGAGTTGCCAATTCGAATGCCAAGAATAGCAACTAATCACCAATAAACAGCTACAACGGAACT
TCCGAGCTCCAGCTCGAGCGCATGAGCGTTTACTTCAACGAGGTACGCAACCAGGGGGACCTTCAA
CACATGTGGAAGAGTAGTTACTAATCACCCCAACATGCACAGGCATCTGGTAACAAATACGTTCCT
CGAGCCGTCCTCGTCGATCTCGAGCCCGGTACCATGGATGCCGTCCGTGCTGGTCCTTTCGGCCAGC
TCTTCCGACCTGACAACTTCGTCTTCGGTCAGTCCGGTGCCGGAAACAACTGGGCCAAGGGTCACT
ACACTGAGGGTGCCGAGCTTGTCGACAACGTTCTGGATGTCGTTCGTCGCGAGGCTGAGGGATGCG
ACTGCCTTCAGGGTTTCCAGATCACCCACTCTCTCGGTGGTGGTACCGGTGCCGGTATGGGTACTCT
GTTGATCTCCAAGATCCGCGAAGAGTTCCCCGACCGCATGATGGCTACGTTCTCCGTCGTTCCCTCC
CCTAAGGTTTCCGACACCGTTGTCGAGCCTTACAACGCCACTCTCTCCGTCCACCAGCTGGTCGAGA
ACTCCGATGAGACCTTCTGCATTGACAACGAGGCTCTCTACGACATCTGCATGCGTACGCTGAAGC
TGTCTAACCCCTCGTACGGCGACCTGAACCATCTGGTCTCCGCCGTCATGTCCGGTGTTACTACCTG
CTTGCGTTTCCCTGGTCAGCTGAACTCTGATCTGCGCAAGCTCGCCGTGAACATGGTTCCTTTCCCT
CGTCTCCACTTCTTCATGGTCGGATTCGCTCCCCTGACCAGCCGTGGCGCTTACTCCTTCCGTGCCG
TCACCGTTCCCGAGTTGACTCAGCAGATGTTCGACCCCAAGAACATGATGGCTGCCTCCGACTTCC
GCAACGGTCGCTACCTGACATGCTCTGCCATCTT--

>Jackrogersella_cohaerens

-CATTACAGAGTTAAACAAACTCCAAACCCTTTGTGAACCTTACCATAGTTGCCTCGGCGTG-
CGCGCGGCTACCCGGTTTCCGGCCC-----------CCCAGAAGG----------GTGGTTACCCTGT-AGCCGGC-
CAACAGCCCGCCGAAGGACCCCTAAACTCTGTTTAAAATGGTGTATTCTGAATTACTTAAACTAAA
TAAGTTAAAACTTTCAACAACGGATCTCTTGGTTCTGGCATCGATGAAGAACGCAGCGAAATGCGA
TAAGTAATGTGAATTGCAGAATTCAGTGAATCATCGAATCTTTGAACGCACATTGCGCCCATTAGT
ACTCTAGTGGGCATGCCTATTCGAGCGTCATTTCGACCATTAAG-
CCCTGCAGCTTAGCGTTGGGAGCCTACGCCCTGCGGCGTAGCTCCTCAAAGTCAGTGGCGGAGTTG
GAGCACACTCTAAGCGTAGTAAATTATCTCGCTTCTGTAGTTGCCCCGGCTGCCTGCCGTAAAACCC
CCTATATTTCTAGTGGTTGACCTCGGATTAGGTAGGAATACCCGCTGAACTTAAGCATATGAAGCG
GCAACAGCTCAAA-TTTGAAATCTGGCC----CTC-----
GTGGTCCGAGTTGTAATTTGTAGAGGATGCTTTTGGTGCGGT-
GCCTTCCGAGTTCCCTGGAACGGGACGCCAGAGAGGGTGAGAGCCCCGTACGGTTGGA-
CACCTACCCTATATATAGCTCCTTCGACGAGTCGAGTAGTTTGGGAATGCTGCTCTAAATGGGAGG
TAAATTTCTTCTAAAGCTAAATACCGGCCAGAGACC-
GATAGCGCACAAGTAGAGTGATCGAAAGATGAAAAGCACTTTGAAAAGAGGGTTAAATAGCACGT
GAAATTGTTGAAAGGGAAGCG-TTTGCGACCAGACTTTTTCCAGGGGGATCATCCGGTGTTC--
TCACCGGTGCACTCCGCCTGG--TTTAGGCCAGCATCGGTTCT---
CTTAGGGGGGATAAAGGCTTGGGGAACGTAGCTC----CTTCGGGAGTGTT-
ATAGCCCCTTGCGTAATACCCTTCG-GGGGACCGAGGATCGCGCT-CT----
GCAAGGATGCTGGCGTAATGGTCGTCAACGACCCGTCTTGAAACACGGACCAAGGAGTCGAACAT
TTGTGCGAGTGTTTGGGTG--TTAAACCCTCACGCGTAATGAAAGTGAACGGAGGTGAGAGCCCTT--
--AC----GGGTGCATCATCGACCGATCCTGATG-
TCTTCGGATGGATTTGAGTAAGAGCATAACTGTTCGGACCCGAAAGATGGTGAACTATGCGTGGAT
AGGGTGAAGCCAGAGGAAACTCTGGTGGAGGCTCGCAGCGGTTCTGACGTGCAAATCGATCGTCA
AATCTGCGCATGGGGGCGAAAGACTTATCGA-
ACCATCTAGTAGCTGGTTACCGCCNAAGTTTCCCTCAGGATAGCAGTGT--TG-
TTTTCAGTTTTATGAGGTAAAGCGAATGATTAGGGACTCGGGGGCGCTATTTAGCCTTCATCCATTC
TCAAACTTTAAATANNTAAGAAGCCCTTNTTACTTAATTGAACGTGGGCATTCGAATGTATCANCA
CTAGTGGGCCATTTTTGGTAAGCAGAACTGGCGATGCGGGATGAACCGAACGCGGGGTTAAGGTG
CCAGAGTGGACGCTCATCAGNCACCACAAAAGGTGTTAGTACATCTTGACAGCAGGACGGTGGCC
ATGGAAGTCGGAATCCGCTAAGGAC---

GCGTTGCATCGATGGGAACAAACGTTTCCAAATTGAACTAGCTGCCAAACCCGCCATCATCACTAA
TGGCTTGAAATATTCTCTCGCCACAGGTAACTGGGGTGATCAGAAAAAGGCGATGAGCTCGACGGC
TGGTGTATCCCAAGTCTTGAACCGTTACACTT------TCTCGTCAA---
CTCTTTCTCATTTGAGAAGAACGAACACACCTATCGGGAGAGATGGGAAACTCGCCAAGCCGCGGC
AACTTCATAACACTCATTGGGGTCTGGTCTGTCCGGCCGAGACACCCGAAGGCCAAGCCTGCGGAC
TGGTGAAGAATCTGTCACTCATGTGTTCTATCAGCGTGGGAACATCGACTGATCCTATTGTGGACTA
TATGATTACTAGAAATATGGAAGTCCTCGAGGAGTATGAACCGATGAGATATCCCAACGCTACCAA

GATCTTTCTAAACGGCTCTTGGATCGGTGTTCACCAGGATCCCAAGACCCTCGTTAGGGATGTACA
AAACCTTCGCCGGAACAATCAAATTCCTTCCGAAGTCTCGTTGGTTCGCGATATCCGCGATCGTGA
GTTCAAGATCTTTTCAGATGCCGGCCGTGTGATGCGTCCATTGTTC---

--------CCCCTGAACGCGTCCTCCAAACCCCTTGATTTCTGCCCCTCACGCACAAAAAAC---
ACCACCATATTCCATCGTTGTTTACTTCACGCCAGCTTCAAATTACACAATCGTGATTCTCGATCAA
TAAGCTAACCATATCTTTTCATCCCAATAGGTTCACCTCCAGACCGGCCAATGCGTAAGTTTTATTA
C----
ACTAACTACAGACGAGATATCGCGCAGGAATATGGCGGGGCTAATATGAAGATGGTAGGGTAACC
AAATTGGTGCTGCTTTCTGGCAAACCATCTCTGGCGAGCACGGCCTCGACAGCAATGGCGTGTAAG
TATCTGAGTCGTCAATTCCAAGGCCAAGGATAGCAACTGACAACCAATAAACAGCTACAACGGAA
CCTCTGAGCTCCAGCTTGAGCGCATGAGCGTCTACTTCAACGAGGTACGCAATCCAAGAAACCGAT
TCAAGATGCAA-
GGAGTAGTTACTAATCACCCCAACATACACAGGCATCTGGTAACAAGTATGTTCCTCGCGCCGTCC
TCGTCGACCTCGAGCCCGGCACCATGGACGCCGTTCGCGCCGGTCCTTTCGGTCAGCTTTTCCGACC
TGACAACTTCGTTTTCGGCCAGTCTGGTGCCGGAAACAACTGGGCCAAGGGTCATTACACCGAGGG
TGCTGAGCTTGTCGACCAGGTTCTTGATGTCGTTCGTCGCGAGGCTGAGGGATGTGATTGCCTCCAG
GGTTTCCAGATCACCCACTCCCTCGGTGGTGGTACCGGTGCCGGTATGGGAACCTTGTTGATCTCCA
AGATCCGCGAGGAGTTCCCCGACCGAATGATGGCTACCTTCTCCGTCGTTCCCTCTCCCAAGGTCTC
CGACACCGTCGTCGAGCCCTACAACGCCACCCTGTCCGTCCATCAGCTGGTCGAGAACTCGGACGA
GACCTTCTGCATTGACAACGAGGCTCTCTACGACATCTGCATGCGCACGCTGAAGCTGTCTAACCC
TTCGTACGGTGACCTGAACCACCTGGTCTCCGCCGTCATGTCTGGTGTCACCACCTGCTTGCGTTTC
CCCGGCCAGCTGAACTCTGACCTGCGCAAACTCGCCGTGAACATGGTTCCTTTCCCCCGTCTCCATT
TCTTCATGGTCGGCTTTGCTCCCCTGACCAGCCGTGGCGCCTACTCTTTCCGTGCCGTTACCGTTCCC
GAGTTAACTCAGCAGATGTTTGACCCCAAGAACATGATGGCTGCTTCTGACTTCCGCAACGGTCGC
TACCTGACGTGCTCTGCCATCTTGTAAGATAAATACACGATACGGTCCGTGATACCTAATTTGCTAA
CCCGAACTTTTTAGCCGTGGCAAGATCTCCA

>Jackrogersella_multiformis

--------------------CTCCAAACCCTTTGTGAACCTTACCTCAGTTGCCTCGGCGCT-
CGCGCGGCTACCCCGTAGTCGCCCTGTAG---------------------AACGGGCGGCTACCCTGT-AGCCGGC-
TCACGGCCCGCCGAAGGACCCCTAAACTCTGTTTAAAATAGTGTATTTCTGAATGCTCCAACAAAA
TAAGTTAAAACTTTCAACAACGGATCTCTTGGTTCTGGCATCGATGAAGAACGCAGCGAAATGCGA
TAAGTAATGTGAATTGCAGAATTCAGTGAATCATCGAATCTTTGAACGCACATTGCGCCCATTAGT
ATTCTAGTGGGCATGCCTATTCGAGCGTCATTTCGACCATTAAG-
CCCTGTAGCTTAGCGTTGGGAGCCTACGGTT-
GTAGCGTAGCTCCTCAAAGTCAGTGGCGGAGTTATGGCGTACTCTGAGCGTAGTAAATTTTCTCGC
TTCTGTAGTCGCCCTAGCTGCCTGC--
-----------------C----CTA-----GCGGTCCGAGTTGTAATTTGCAGAGGATGCTTTTGGTGCGGT-
GCCTTCCGAGTTCCCTGGAACGGGACGCCGGAGAGGGTGAGAGCCCCGTACGGTTGGA-
CACCTACCCTATACATAGCTCCTTCGACGAGTCGAGTAGTTTGGGAATGCTGCTCTAAATGGGAGG
TAAATTTCTTCTAAAGCTAAATACCGGCCAGAGACC-
GATAGCGCACAAGTAGAGTGATCGAAAGATGAAAAGCACTTTGAAAAGAGGGTTAAATAGCACGT
GAAATTGTTGAAAGGGAAGCG-TTTGCGACCAGACCTTTTCCAGGCGGATCATCCGGCGTTC--
TCGCCGGTGCACTCCGCCTGG--TCTAGGCCAGCATCGGTTTC---
CTTAGGGGGATAAAGGCCTGGGGAACGTAGCTC----TTCAGGGAGTGTT-
ATAGCCCCTAGCGTAATACCCTTCA-GGGGACCGAGGACCGCGCT-TCG---
GCAAGGATGCTGGCGTAATGGTCGTCAACGACCCGTCTTGAAACACGGACCAAGGAGTCGAACAT
TTGTGCGAGTGTTTGGGTG--TCAAACCCTCACGCGTAATGAAAGTGAACGGAGGTGAGAGCCCTT--
--AC----GGGTGCATCATCGACCGATCCTGAAG-
TCTTCGGATGGATTTGAGTAAGAGCATAACTGTTCGGACCCGAAAGATGGTGAACTATGCGTGGAT
AGGGTGAAGCCAGAGGAAACTCTGGTGGAGGCTCGCAGCGGTTCTGACGTGCAAATCGATCGTCA
AATCTGCGCATGGGGGCGAAAGACTTATCGA-
ACCATCTAGTAGCTGGTTACCGCCGAAGTTTCCCTCAGGATAGCAGTGT--TG-
TCTTCAGTTTTATGAGGTAAAGCGAATGATTAGGGACTCGGGGGCGCTATTTTGCCTTCATCCATTC

TCAAACTTTAAATATGTAAGAAGCCCTTGTTGCTTAATTGAACGTGGGCATTCGAATGTATCAACA
CTAGTGGGCCATTTTTGGTAAGCAGAACTGGCGATGCGGGATGAACCGAACGCGGGGTTAAGGTG
CCAGAGTGGACGCTCATCAGACACCACAAAAGGTGTTAGTACATCTTGACAGCAGGACGGTGGCC
ATGGAAGTCGGAATCCGCTAAGGACTGTGTAACAACTCACCTGCCGAATGTACTAGCCCTGAAAAT
GGATGGCGCTCAAGCGTCT-CACCCATACCTCGCCCTTAGGGTAGAAACGATGCCCTAAGG-----------
----

GTTTCCNAATCGAACTAGCTGCCAAGCCCGCCATCATCACTAACGGTTTGAAATATTCTCTCGCTAC
AGGTAACTGGGGTGATCAGAAAAAGGCGATGAGCTCGACGGCTGGTGTATCACAAGTCTTGAACC
GCTACACTT------TCTCATCAA---
CTCTTTCCCATTTGAGAAGGACAAACACACCTATCGGAAGAGACGGGAAGCTCGCCAAGCCACGA
CAGCTTCATAACACTCACTGGGGCCTGGTGTGTCCGGCCGAGACGCCCGAAGGCCAAGCCTGCGGG
CTGGTGAAGAATCTGTCGCTCATGTGTTCCATCAGCGTGGGAACCTCGACGGATCCTATTGTCGATT
ATATGATAACGAGAAATATGGAAGTCCTCGAGGAGTATGAGCCGATGAGATATCCCAACGCCACT
AAGATCTTTCTGAACGGTTCTTGGATCGGTGTTCACCAGGACCCCAAGTCCCTCGTTCGAGACGTAC
AGACGCTCCGCCGTAACAACCAAATTCCTTCTGAAGTCTCGTTGGTTCGCGATATCCGCGATCGTG
AATTCAAGATCTTCTCGGATGCCGGTCGTGTGATGCGTCCTCTGTTCCGTGTACAACAGGATGAT----
----

CTTCCCGAGGAGGGCATAAACAAGGGCACCTTAGCTCTAACCAAGGATATGATCCAGCGTTTAGAG
GCCGACCTTGATTTGGATCCGGATAGTGAAGAATACTTCGGTTGGCAAGGACTAGTCAACGAGGGT
GTTGTTGAATTCCTTGATGCCGAGGAAGAGGAGACAGCCATGATTTGCATGACGCCCGAGGATTTA
GAAAACTTCCGACTGGCCAAGGCTGGATACCAGGTAGTTCTAGACAACGGA------
GACGAGGTGAACAAGCGATTGAAGACTAAGTTCAACCCGTCAACGCACATGTACACTCATTGCGA
AATCCACCCCAGTATGCTCCTAGGCATCTGCGCAAGCATCATTCCGTTCCC----------------
CCCCTGAACGCGTCCTCCAAAACCCTTGATTCCTGCCCCTCACGCACACTAAAC---
ACTGCCGTATTTCGCAATTGTTTACTTCACATCATCTTCAAACTACGCAACGATGATGCTCGATCAA
CATGCTAACCATATTTTTTCATCTCGATAGGTTCACCTCCAGACCGGCCAATGCGTAAGTTTTACCG
C----
CTCGACCACGGACGCAACGTCGCGCGGGAATATAGCGGGGCTAACGTGAAGATGGTAGGGTAACC
AAATTGGTGCTGCTTTCTGGCAAACCATCTCTGGCGAGCACGGCCTCGACAGCAATGGCGTGTAAG
TATTTTAGTCGTCAATTCCAACGCCAAGGATAGCAACTGACAGCCAATAAACAGCTACAATGGAAC
TTCGGAGCTCCAGCTTGAGCGCATGAGCGTCTACTTCAACGAGGTACGCAATCCAAGAAACCCACG
ATAGCATCCAAGGAGCAGTTACTAATCACCCCAACATACACAGGCATCTGGTAACAAGTATGTTCC
CCGCGCCGTCCTCGTCGATCTCGAGCCCGGCACCATGGACGCCGTTCGTGCCGGTCCTTTCGGCCA
GCTTTTCCGACCTGACAACTTCGTCTTCGGCCAGTCCGGTGCCGGAAACAACTGGGCCAAGGGTCA
TTACACTGAGGGTGCTGAGCTTGTCGACCAAGTTCTCGATGTCGTCCGTCGCGAGGCTGAGGGCTG
TGACTGCCTCCAGGGTTTCCAGATCACCCACTCCCTCGGTGGTGGTACCGGTGCCGGTATGGGAAC
CCTGTTGATCTCCAAGATCCGCGAGGAGTTCCCTGACCGAATGATGGCTACCTTCTCCGTCGTTCCC
TCTCCAAAGGTCTCCGACACCGTCGTCGAGCCTTACAACGCCACCCTCTCCGTCCATCAGCTAGTCG
AGAACTCCGATGAGACCTTCTGCATCGACAACGAGGCCCTCTACGACATCTGCATGCGCACGCTGA
AGCTGTCTAACCCCTCGTACGGTGACCTGAACCACCTGGTCTCCGCTGTCATGTCTGGTGTCACCAC
CTGCTTGCGTTTCCCCGGCCAGCTGAACTCTGACCTGCGCAAACTCGCCGTGAACATGGTTCCCTTC
CCCCGTCTCCATTTCTTCATGGTCGGCTTCGCTCCTCTGACCAGCCGTGGCGCTTACTCCTTCCGCGC
CGTCACCGTTCCCGAGTTGACTCAGCAGATGTTCGACCCCAAGAACATGATGGCTGCCTCCGACTT
CCGCAACGGTCGCTACCTGACGTGCTCTGCCATCTTGTAAGATGATATGTTCCATCCGTCTATGATA
ACTAATTTGCTAACTTGAATTTTCTAGCCGTGGCAAGATCTCCA

>Jackrogersella_minutella

-CATTACTGAGTAAACAAAACTCCAAACCCTTTGTGAACCTTACCTTAGTTGCCTCGGCGTG-
CGCGCGGCTACCCGGGAGGACCGCTGTAG-------------------------GCGGTTACCCTGT-AGCCGGC-
CCACGGCCCGCCGAAGGACCACTGAACTCTTGTTTTCACATTGCATCTCTGATTTAAACA-
TTAAATAAGTTAAAACTTTCAACAACGGATCTCTTGGTTCTGGCATCGATGAAGAACGCAGCGAAA
TGCGATAAGTAATGTGAATTGCAGAATTCAGTGAATCATCGAATCTTTGAACGCACATTGCGCCCA
TTAGTATTCTAGTGGGCATGCCTATTCGAGCGTCATTTCGACCATTAAGCCCCTGTAGCTTAGCGTT
GGGAGCCTACGTCCTGCGGCGCAGCTCCTCAAAGTCAGTGGCGGAGTCGGGTCGTGCTCTGAGCGT
AGTAGTATATCTCGCTTCTGCGGTGCCCCCGGCTGCCTGCCGTAAAACCCCCTATATTCGTATTGGT
TGACCTCGGATTAGGTAGGAATACCCGCTGAACTTAAGCATATGAAGCGGCAACAGCTCAAA-
TTTGAAATCTGGCC----CTA-----GCGGTCCGAGTTGTAATTTGCAGAGGATGCTTTTGGTGCGGT-

GCCTTCCGAGTTCCCTGGAACGGGACGCCAGAGAGGGTGAGAGCCCCGTACGGTTGGA-
CGCCTACCCTATATATAGCTCCTTCGACGAGTCGAGTAGTTTGGGAATGCTGCTCTAAATGGGAGG
TAAATTTCTTCTAAAGCTAAATACCGGCCAGAGACC-
GATAGCGCACAAGTAGAGTGATCGAAAGATGAAAAGCACTTTGAAAAGAGGGTTAAATAGCACGT
GAAATTGTTGAAAGGGAAGCG-TTTGCGACCAGACCTTCTCCAGGCGGAACATCCGGTGTTC--
TCACCGGTGCACTTCGCCTGG--TCTAGGCCAGCATCGGTTTC---
CTTAGGGGGATAAAGGCCTGGGGAACGTAGCTC----TTTAGGGAGTGTT-
ATAGCCCCTAGCGTAATACCCCTCG-GGGGACCGAGGACCGCGCT-TCG---
GCAAGGATGCTGGCGTAATGGTCGTCAACGACCCGTCTTGAAACACGGACCAAGGAGTCGAACAT
TTGTGCGAGTGTTTGGGTG--TCAAACCCTCACGCGTAATGAAAGTGAACGGAGGTGAGAGCCCTT--
--A-G---GGGTACATCATCGACCGATCCTGATG-
TCTTCGGATGGATTTGAGTAAGAGCATAACTGTTCGGACCCGAAAGATGGTGAACTATGCGTGGAT
AGGGTGAAGCCAGAGGAAACTCTGGTGGAGGCTCGCAGCGGTTCTGACGTGCAAATCGATCGTCA
AATCTGCGCATGGGGGCGAAAGACTTATCGA-ACCAT--

CGATACACTTTCC---TCCGTCCGA---
CCCTTTCCCATCTTAGACGAACGAACACCCCCATCGGAAGAGACGGAAAGTTGGCCAAACCGCGA
CAGCTTCACAACACCCATTGGGGTCTCGTCTGTCCGGCCGAGACGCCCGAAGGTCAAGCTTGCGGG
TTGGTGAAGAACTTGTCTCTGATGTGTTCCATCAGCGTGGGAACATCGACCGATCCTATCGTGGACT
ATATGATTACTAGAAATATGGAAGTCCTCGAGGAGTACGAGCCGATGAGATACCCCAACGCGACC
AAGATCTTCCTCAACGGCTCCTGGATCGGTGTGCACCAGGACGCCAAGACCCTGGTTCGAGACGTC
CAGGCGCTTCGTCGCGCTAATCAGATCCCCTCCGAGGTATCATTAGTGCGTGACATTCGTGATCGC
GAGTTCAAGATCTTCTCAGATGCCGGCCGTGTGATGCGCCCCTTGTTTCGCGTACAACAAGAGGAC-
----
ATCCCCGAGAGCGGCATTAGCAAGGGCACTCTAGCTCTTAGCAAAGACATGATCCAGCGCTTAGAG
GCGGATGTTGATCTGGATCCTGAGAGCGAAGAATACTTTGGCTGGCAAGGCCTGGTCAACGAGGGT
GTCATCGAATTCCTCGATGCGGAGGAGGAGGAGACCGCGATGATTTGCATGACGCCCGAAGATCT
AGAAATCTTTCGCATGACAAAGCTCGGATACGACATGGTCCAAGACAACGGC------
GACGAGGTGAATAAGCGACTGAAGACCAAGGTAAACCCGTCGACACACATGTATACCCACTGCGA
GATCCATCCCAGCATGCTCCTGGGTATCTGCGCGAGCATCATCCCGTTCCCGGATCACAACCAGGT
ACCCCTGAACGCGTCCCTCAAACCCCCTGATTTCTGCCCCTCACGCACAGAAAAAACAACATCAAG
TCCCAATAGCTACCTGCGTTACGCCATCTTGGAAATTCAAAACAATGATTTTCAATCAAAATGCTA
ACCGCATATT---CTCTCAATAGGTTCACCTTCAGACCGGCCAATGCGTAAGTTTTAT-------
CACGACTACAGACGAAATATCGCGATAGAAGATAGCGGGGCTCACACAAATATTGTAGGGTAACC
AAATCGGTGCTGCTTTCTGGCAAACCATCTCTGGCGAGCACGGCCTCGACAGCAATGGCGTGTAAG
TATCTAAGTCGTCAATTCTATCGCCAAGAATATCAACTAATCACCAATGAACAGTTACAATGGAAC
TTCTGAGCTCCAGCTTGAGCGCATGAGCGTCTACTTCAACGAGGTACGCAGCTAGATAAACCATCA
ACATGATTCAGAGAGTAGTTACTAATCACCCCAACATGCACAGGCATCTGGTAACAAGTACGTCCC
TCGTGCCGTCCTTGTCGACCTCGAGCCCGGTACCATGGACGCCGTTCGTGCCGGCCCCTTCGGACA
GCTTTTCCGACCTGACAACTTCGTCTTCGGCCAGTCTGGTGCCGGAAACAACTGGGCCAAAGGTCA
TTACACTGAGGGTGCTGAGCTTGTCGACCAGGTTCTCGATGTCGTTCGTCGCGAGGCTGAGGGATG
CGACTGCCTCCAGGGTTTCCAAATCACTCACTCCCTCGGTGGTGGTACCGGTGCCGGTATGGGTACT
CTGTTGATCTCCAAGATCCGTGAGGAGTTCCCCGACCGAATGATGGCTACCTTCTCCGTCGTTCCCT
CCCCCAAGGTCTCCGACACCGTTGTTGAGCCTTACAACGCTACCCTCTCCGTCCACCAGCTGGTCGA
GAACTCCGACGAGACCTTCTGCATTGACAACGAGGCTCTCTACGACATCTGCATGCGTACGCTGAA
GCTGTCCAACCCCTCGTACGGTGACTTGAACCACCTGGTCTCCGCCGTCATGTCTGGTGTCACCACC
TGTCTACGTTTCCCTGGCCAGCTGAACTCCGACTTGCGCAAGCTCGCCGTGAACATGGTTCCCTTCC
CCCGTCTTCACTTCTTCATGGTCGGCTTCGCTCCTTTGACCAGCCGTGGCGCTTACTCCTTCCGTGCC
GTCACCGTTCCCGAGTTGACGCAGCAGATGTTCGACCCCAAGAACATGATGGCTGCTTCCGACTTC
CGCAACGGTCGTTACCTGACGTGCTCTGCCATCTTGTAAGATACCCTATTCCCACAGTCTTGTAATA
CCAATTCGCTAACACTAAATTTTTAGCCGTGGTAAGATCTCCA

>Hypoxylon_investiens

-CATTACTGAGTTATCAAAACTCCCAACCCTTTGTGAA-CCTACCGCCGTTGCCTCGGCGTG-
AGCGCGGCTACCCTGGAGCTACCCTGGAGCTACCTACCCTGCAGCTAGTCGCTCCGGGGCTACCCT
GT-AGCCGGC-
TTACGGCCCGCCGAAGGACCGCCAAACTCTTGTTTTTGCACTGCATGTCTGAATTTTAAA-CT-
AAATAATTAAAACTTTCAACAACGGATCTCTTGGTTCTGGCATCGATGAAGAACGCAGCGAAATGC
GATAAGTAATGTGAATTGCAGAATTCAGTGAATCATCGAATCTTTGAACGCACATTGCGCCCATTA
GTATTCTAGTGGGCATGCCTATTCGAGCGTCATTACGACCCCTAAGCCCCTGTTGCTTAGCGTTGGG
AATCTAC-------
GGCGTAGTTCCTTAAAATCAGTGGCGGAGTTAGGGTACACTCTCAGCGTAGTAATTTCTCTCGCTCG
TGTGGTGGCCTTGGCTGCTAGCCGTTAAACCCCCTAT-
ATCTTAGTGGTTGACCTCGAATTAGGTAGGAATACCCGCTGAACTTAAGCATATGAAGCGGCAATA
GCTCAAA-TTTGAAATCTGGCC----CTC-----
GCGGTCCGAGTTGTAATTTGTAGAGGATGCTTTTGGTGCGGT-
GCCTTCTGAGTTCCCTGGAACGGGACGCCAAAGAGGGTGAGAGCCCCGTACGGTTGGA-
CACCTAGCCTATATATAGCTCCTTCGACGAGTCGAGTAGTTTGGGAATGCTGCTCTAAATGGGAGG
TAAATTTCTTCTAAAGCTAAATACCGGCCAGAGACC-
GATAGCGCACAAGTAGAGTGATCGAAAGATGAAAAGCACTTTGAAAAGAGGGTTAAATAGCACGT
GAAATTGTTGAAAGGGAAGCG-TTTGCGACCAGACCTTTTCTAGGGGGATCATCCGGCGTTC--
TCGCCGGTGCACTTCCCTTAG--TTTAGGCCAGCATCGGTTCC---
CTTAGGGGGATAAAGGCTTGGGGAACGTAGCTC----TCTAGGGAGTGTT-
ATAGCCCCTTGCGTAATACCCCTCG-GGGGACCGAGGACCGCGCT-CT----
GCAAGGATGCTGGCGTAATGGTCGTCAACGACCCGTCTTGAAACACGGACCAAGGAGTCGAACAT
TTGTGCGAGTGTTTGGGTG--TTAAACCCTCACGCGTAATGAAAGTGAACGGAGGTGAGAGCCCTC--
--GC----GGGTGCATCATCGACCGATCCTGATG-
TCTTCGGATGGATTTGAGTAAGAGCATAACTGTTCGGACCCGAAAGATGGTGAACTATGCGTGGAT
AGGGTGAAGCCAGAGGAAACTCTGGTGGAGGCTCGCAGCGGTTCTGACGTGCAAATCGATCGTCA
AATCTGCGCATGGGGGCGAAAGACTTATCGA-
ACCATCTAGTAGCTGGTTACCGCCGAAGTTTCCCTCAGGATAGCAGTGT--TG-
TCTTCAGTTTTATGAGGTAAAGCGAATGATTAGGGACTCGGGGGCGCTATTTTGCCTTCATCCATTC
TCAAACTTTAAATATGTAAGAAGCCCTTGTTACTTAGTTGAACGTGGGCATTTGAATGTATCAACAC
TAGTGGGCCATTTTTGGTAAGCAGAACTGGCGATGCGGGATGAACCGAACGCGGGGTTAAGGTGC
CAGAGTGGACGCTCATCAGACACCACAAAAGGTGTTAGTACATCCAGACAGCAGGACTGTGGCCA
TGGAAGTCGGAATCAGCTAAGGACTGTGTAACAACTCACCTGCCGAATGTACTAGCCCTGAAAATG
GATGGCGCTCAAGCGTCT-CACCCATACCCCGCCCTTAGGGTAGAAACGAAGCCCTAAGG-------------
----
---T----------
CATTGGGGTCTTGTCTGTCCAGCTGAGACGCCCGAAGGCCAGGCTTGCGGACTGGTGAAGAACTTA
TCGCTGATGTGCTCTATCAGCGTGGGTACATCGACGGATCCTATCGTGGAGTATATGATTACTAGA
AGTATGGAAGTCCTCGAGGAATACGAACCGATGCGATACCCGAATGCCACCAAGATCTTCCTTAAC
GGGTCTTGGATCGGTGTACACCAGGATCCCAAGACTCTTGTCAAGGATATCCAGGCCCTTCGTCGG
GCCAACCAGATCCCCTCCGAGGTTTCCTTGGTCCGCGATATCCGTGATCGCGAGTTCAAGATATTCT
CAGATGCAGGTCGTGTCATGCGCCCCTTGTTTGTCGTGCAACAAGAAGAT----------------------------------
----
ATCCCCGATCAGGGTATTGCTAAGGGTACATTGGCCCTTACCAAAGAGATGATCCAACGACTAGAG
GCCGATGTTGATCTTGATCCTGAAAGCGAGGAGTACTTCGGCTGGCAAGGTCTCGTTAACGAGGGT
GTTATTGAGTTTCTCGACGCGGAGGAAGAGGAAACGGCTATGATTTGCATGACACCGGAAGATCTG
GAAAACTACCGGATGGCCAAGCTCGGCTACGAAGTGGTTCAGGATAACGGA------
GATGAGGTTAACAAACGACTCAAAACCAAGGTCAACCCCTCAACGCACATGTATACTCATTGCGAA
ATCCATCCTAGCATGCTCTTGGGTATCTGCGCGAGTATCATTCCCTTCCCTGACCACAACCAGTCGC
CCCTGAACGCGTCCTCTCAACCCCTGATTTCCTGCCCCTCATGCACACAAAAC---
AGTACAGCTTCATGCAGCTCTTTTCGCAACGATTCAATCAAACCGAACAACGATG-
AGATGAAAATCGCAGCTAAC----
AATGTTTTTCTCAATAGGTTCACCTCCAGACCGGCCAATGCGTAAGTACAACGAT----
CACAACCGCGACCGAGGTAACGCGCTGGAATATAGAGGGGCTTACACAAACTA-
ACAGGGTAACCAAATTGGTGCTGCTTTCTGGCAAACCATCTCTGGCGAGCACGGTCTCGACAGCAA
TGGCGTGTAAGTATATGAGTTGTCAATTCCGATGCCAAGAATAGCACCTAATGACCAATAATTAGC
TACAACGGAACCTCCGAGCTCCAGCTCGAGCGCATGAGCGTCTACTTCAACGAGGTATGCAAGAAT

CGAAGCCAGGGATAGAGATTCAAGGTCGGCTACTAATCAACCTAACCTACACAGGCTTCCGGCAA
CAAGTATGTCCCTCGCGCCGTCCTCGTCGATCTCGAGCCCGGTACCATGGACGCTGTCCGTGCTGGT
CCCTTCGGCCAGCTTTTCCGACCTGACAACTTCGTCTTCGGCCAGTCTGGTGCCGGAAACAACTGGG
CCAAGGGTCACTACACTGAGGGTGCTGAGCTTGTTGACAACGTCCTTGATGTCGTTCGTCGTGAGG
CTGAGGGCTGCGACTGCCTCCAGGGTTTCCAGATCACCCACTCCCTCGGTGGTGGTACTGGTGCCG
GTATGGGTACTCTCTTGATCTCCAAGATCCGTGAGGAGTTCCCCGACCGCATGATGGCCACCTTCTC
CGTTGTGCCTTCCCCTAAGGTTTCCGACACTGTCGTCGAACCCTACAACGCCACCCTCTCGGTCCAC
CAGCTGGTCGAGAACTCTGACGAGACCTTCTGCATTGACAACGAGGCTCTGTACGACATCTGCATG
CGTACCCTGAAGCTATCCAACCCCTCGTACGGTGACCTGAACCACCTGGTCTCTGCTGTCATGTCCG
GTGTCACCACTTGCTTGCGTTTCCCCGGTCAGCTGAACTCTGATCTGCGCAAGCTCGCCGTGAACAT
GGTTCCTTTCCCTCGTCTCCACTTCTTCATGGTCGGCTTCGCTCCTCTGACCAGCCGTGGCGCTCACT
CCTTCCGTGCCGTTACCGTTCCCGAGTTGACTCAGCAGATGTTCGATCCCAAGAACATGATGGCTGC
TTCTGACTTCCGTAACGGTCGTTACCTCACTTGCTCTGCCATCTTGTAAGATACCCAGCCTAAACCT
CCATAAATCTCTGATTCACTAACTCTATTTATCTAGCCGTGGCAAGGTTTCCA

>Hypoxylon_lateripigmentum

-------------------------AAACCTTTGTGAACTTAACCGTCGTTGCCTCGGCGTG-
AGCGCGGCTACCCGGTAGCTACCCT------------------------------GTAGCTACCCTGT-AGCCGGT-
TCACGGCCCGCCGAAGGACAGCTAAACTCTTGTAATACCACTGTATCTCTGAATTGTCAA-
CTAAATAAGTTAAAACTTTCAACAACGGATCTCTTGGTTCTGGCATCGATGAAGAACGCAGCGAAA
TGCGATAAGTAATGTGAATTGCAGAATTCAGTGAATCATCGAATCTTTGAACGCACATTGCGCCCA
TTAGTATTCTAGTGGGCATGCCTATTCGAGCGTCATTTCAACCCTTAAGCCAATGCTGCTTAGTGTT
GGGAGCATACCCTCCCGGGGGTATCTCCTTAAAGTTAGTGGCGGAGTTAGGGCACACTCTCAGCGT
AGTAATTTCTCTCGCTCGG--
-----------A-TTTGAAATCTGGCC----CTC-----
GTGGTCCGAGTTGTAATTTGTAGAGGATGCTTTTGGTGCGGT-
GCCTTCCGAGTTCCCTGGAACGGGACGCCAGAGAGGGTGAGAGCCCCGTACGGTTGGA-
CGCCTAGCCTACATATAGCTCCTTCGACGAGTCGAGTAGTTTGGGAATGCTGCTCTAAATGGGAGG
TAAATTTCTTCTAAAGCTAAATACCGGCCAGAGACC-
GATAGCGCACAAGTAGAGTGATCGAAAGATGAAAAGCACTTTGAAAAGAGGGTTAAATAGCACGT
GAAATTGTTGAAAGGGAAGCG-TTTACGACCAGACCTTCTCCGGGGGGATCATCAGGTGTTC--
TCACCTGTGCACTTCCCCCGG--TTTAGGCCAGCATCGGTTTC---
CTTAGGGGGATAAAGGCTCGGGGAACGTAGCTC----CTTAGGGAGTGTT-
ATAGCCCCTTGCGTAATACCCCTCG-GGGGACCGAGGACCGCGCT-CT----
GCAAGGATGCTGGCGTAATGGTCGTCAACGACCCGTCTTGAAACACGGACCAAGGAGTCGAACAT
TTGTGCGAGTGTTTGGGTG--TTAAACCCTCACGCGTAATGAAAGTGAACGGAGGTGAGAGCCCTT--
--AC----GGGTGCATCATCGACCGATCCTGATG-
TCTTCGGATGGATTTGAGTAAGAGCATAACTGTTCGGACCCGAAAGATGGTGAACTATGCGTGGAT
AGGGTGAAGCCAGAGGAAACTCTGGTGGAGGCTCGCAGCGGTTCTGACGTGCAAATCGATCGTCA
AATCTGCGCATGGGGGCGAAAGACTTATCGA-
ACCATCTAGTAGCTGGTTACCGCCGAAGTTTCCCTCAGGATAGCAGTGT--TG-
TCTTCAGTTTTATGAGGTAAAGCGAATGATTAGGGACTCGGGGGCGCTATTTTGCCTTCATCCATTC
TCAAACTTTAAATATGTAAGAAGCCCTTGTTACTTAGTTGAACGTGGGCATTCGAATGTACCAACA
CTAGTGGGCCATTTTTGGTAAGCAGAACTGGCGATGCGGGATGAACCGAACGCGGGGTTAAGGTG
CCAGAGTGGACGCTCATCAGACACCACAAAAGGTGTTAGTACATCCAGACAGCAGGACGGTGGCC
ATGGAAGTCGGAATCCGCTAAGGACTGTGTAACAACTCACCTGCCGAATGTACTAGCCCTGAAAAT
GGATGGCGCTCAAGCGTCT-
CACCCATACCTCGCCCTTAGGGTAGGATCGATGCCCTAAGGACGGTGTATCGACTCGAACAGACGT
TTCCAGATTGAGCTAGCAGCCAAACCCGCTATCATCACAAATGGATTGAAGTACTCTCTTGCCACA
GGTAACTGGGGTGATCAGAAGAAGGCGGCGAGCTCGACTGCCGGTGTGTCACAGGTCTTGAACCG
TTACACTT------TCGCATCGA---
CTCTATCTCACTTGAGGCGAACAAACACTCCTATCGGGAGAGACGGCAAGCTTGCTAAGCCTCGAC
AACTTCACAACACTCATTGGGGTCTGGTCTGTCCGGCTGAGACGCCTGAAGGCCAGGCTTGTGGAT
TGGTGAAGAACTTGTCGCTCATGTGTTCCATCAGCGTGGGTACATCAACGGATCCTATCGTGGACT
ACATGATTACGAGAAACATGGAAGTTTTGGAGGAATATGAACCCATGCGATACCCTAACGCTACCA
AGATCTTCCTCAATGGTTCTTGGATCGGTGTACATCAGGATCCCAAGACTCTCGTCAGAGATATCCA
GATGCTTCGTCGGGCCAACCAAATTCCCTCTGAAGTTTCTTTGGTCCGCGATATCCGTGATCGTGAG

TTCAAGATCTTCTCGGATGCCGGTCGTGTCATGCGTCCCTTGTTCGTCGTCCACCAAGAGGAT--------
--A---
CTGAGCAAGGCGCTGCTAAGGGAACATTGGCCCTTACCAAAGACATGATCCAGCGACTAGAGGCG
GACGTCGATCTAGATCCCGACAGCGAAGAGTACTTCGGCTGGCAAGGCCTGGTCAACGAGGGTGT
AATTGAGTTCCTCGATGCGGAGGAAGAGGAGACGGCTATGATTTGCATGACACCAGAAGATTTGG
AAGTCTACCGCGCGGCCAAGCTTGGGTATGATGTGGTTCAGGATAACGGT------
GATGAGATTAATAAACGACTCAAGACGAAGATAAACCCCACGACGCACATGTACACGCATTGCGA
GATTCATCCCAGCATGCTTCTCGGCATCTGCGCCAGC--
--------ACGCACACGAAAACC---ACCACAGCTCC-
TGCGGCTGTTTTCGATACGATGTCAACAAACCAAAAGACGNCAGACATAGGAATCAAAGCTAACA-
--GATGTTTGTCTCAATAGGTTCACCTCCAGACCGGCCAATGCGTAAGTACAATGAT----
CACGACCGCAGACGAAGAAACGCGCTGGATTATAGAGGGGCTCACAC--
AAGCTGCAGGGTAACCAAATCGGTGCTGCTTTCTGGCAAACCATCTCTGGCGAGCACGGTCTCGAC
AGCAATGGCGTGTAAGTACCTGAGTTGTCAATTCTGATGCCAAGAACAGGAGCTAACCACCAATAT
TTAGCTACAACGGAACCTCTGAGCTGCAGCTCGAGCGCATGAGCGTCTACTTCAATGAGGTATGCA
GTAATCGAAATCAAGGATGGCCATGCTAGGACAGTCACTAATCACCCTAACCTACACAGGCTTCCG
GCAACAAGTATGTCCCTCGCGCCGTCCTCGTCGATCTCGAGCCCGGTACCATGGATGCCGTCCGTG
CTGGCCCCTTCGGCCAGCTTTTCCGACCTGACAACTTCGTCTTCGGTCAATCCGGTGCCGGAAACAA
CTGGGCCAAGGGTCACTACACTGAGGGTGCTGAGCTGGTTGACAACGTCCTTGACGTCGTTCGTCG
TGAGGCTGAGGGTTGCGACTGCCTCCAGGGTTTCCAGATCACCCACTCCCTCGGTGGTGGTACCGG
TGCCGGTATGGGTACCCTCTTGATCTCCAAGATCCGCGAGGAGTTCCCCGATCGCATGATGGCCAC
CTTCTCCGTCGTTCCTTCCCCTAAGGTCTCCGACACCGTCGTCGAGCCCTACAACGCCACCCTGTCT
GTCCACCAGCTGGTCGAGAACTCGGACGAGACCTTCTGCATTGACAACGAGGCTCTGTACGACATC
TGCATGCGTACCCTGAAGCTATCCAACCCCTCGTATGGTGACCTGAACCACCTGGTCTCTGCCGTCA
TGTCCGGTGTTACCACCTGTTTGCGATTCCCCGGCCAGCTGAACTCCGACCTACGCAAGCTCGCCGT
GAACATGGTTCCTTTCCCTCGTCTTCACTTCTTCATGGTCGGCTTCGCTCCTCTGACCAGCCGTGGCG
CTCACTCCTTCCGTGCCGTCACCGTTCCCGAGTTGACTCAGCAGATGTTCGACCCCAAGAACATGAT
GGCTGCCTCTGACTTCC---

>312

-CATTACTGAGTTATCCAAACTCCAAACCCTTTGTGAACCTTACCGTCGTTGCCTCGGCGTG-
AGCGCGGCTACCCG--GTAGCTACCCTGT-AGCCGGT-
TCACGGCCCGCCGAAGGACAGCTAAACTCTTGTAATACCACTGTATCTCTGAATTGTTAA-
CTAAATAAGTTAAAACTTTCAACAACGGATCTCTTGGTTCTGGCATCGATGAAGAACGCAGCGAAA
TGCGATAAGTAATGTGAATTGCAGAATTCAGTGAATCATCGAATCTTTGAACGCACATTGCGCCCA
TTAGTATTCTAGTGGGCATGCCTATTCGAGCGTCATTTCAACCCTTAAGCCAATGCTGCTTAGTGTT
GGGAGCATACCCTCCGGGGGGTATCTCCTTAAAGTTAGTGGCGGAGTTAGGGTACACTCTCAGCGT
AGTAATTTCTCTCGCTCGGGTAGTGGCCCTGGCTGCTTGCCGTTAAGCCTTTTTTTCTTCTAGTGGTT
GACCTCGGATTAGGTAGGAATACCCGCTGAACTTAAGCATATGAAGCGGCAACAGCTCAAA-
TTTGAAATCTGGCC----CTC-----GTGGTCCGAGTTGTAATTTGTAGAGGATGCTTTTGGTGCGGT-
GCCTTCCGAGTTCCCTGGAACGGGACGCCAGAGAGGGTGAGAGCCCCGTACGGTTGGA-
CGCCTAGCCTATATATAGCTCCTTCGACGAGTCGAGTAGTTTGGGAATGCTGCTCTAAATGGGAGG
TAAATTTCTTCTAAAGCTAAATACCGGCCAGAGACC-
GATAGCGCACAAGTAGAGTGATCGAAAGATGAAAAGCACTTTGAAAAGAGGGTTAAATAGCACGT
GAAATTGTTGAAAGGGAAGCG-TTTACGACCAGACCTTCTCCAGGGGGATCATCAGGTGTTT--
TCACCTGTGCACTTCCCCTGG--TTTAGGCCAGCATCGGTTTC---
TTTAGGGGGATAAAAGCTCGGGGAACGTAGCTC----CTTAGGGAGTGTT-
ATAGCCCCTTGCGTAATACCCCTCG-GGGGACCGAGGACCGCGCT-CT----
GCAAGGATGCTGGCGTAATGGTCGTCAACGACCCGTCTTGAAACACGGACCAAGGAGTCGAACAT
TTGTGCGAGTGTTTGGGTG--TTAAACCCTCACGCGTAATGAAAGTGAACGGAGGTGAGAGCCCTT--
--AC----GGGTGCATCATCGACCGATCCTGATG-
TCTTCGGATGGATTTGAGTAAGAGCATAACTGTTCGGACCCGAAAGATGGTGAACTATGCGTGGAT
AGGGTGAAGCCAGAGGAAACTCTGGTGGAGGCTCGCAGCGGTTCTGACGTGCAAATCGATCGTCA
AATCTGCGCATGGGGGCGAAAGACTTATCGA-
ACCATCTAGTAGCTGGTTACCGCCGAAGTTTCCCTCAGGATAGCAGTGT--TG-
TCTTCAGTTTTATGAGGTAAAGCGAATGATTAGGGACTCGGGGGCGCTATTTGCCTTCATCCATTC

TCAAACTTTAAATATGTAAGAAGCCCTTGTTACTTAGTTGAACGTGGGCATTCGAATGTACCAACA
CTAGTGGGCCATTTTTGGTAAGCAGAACTGGCGATGCGGGATGAACCGAACGCGGGGTTAAGGTG
CCAGAGTGGACGCTCATCAGACACCACAAAAGGTGTTAGTACATCCAGACAGCAGGACGGTGGCC
ATGGAAGTCGGAATCCGCTAAGGACTGTGTAACAACTCACCTGCCGAATGTACTAGCCCTGAAAAT
GGATGGCGCTCAAGCGTCT-CACCCATACCTCGCCCTTAGGGTAGGATCGATGCCCTAAGG-----------
----

GATTGAGCTAGCAGCCAAACCCGCTATCATCACAAATGGACTGAAGTACTCTCTTGCCACAGGTAA
CTGGGGTGATCAGAAGAaGGCGGCGAGCTCGACTGCCGGTGTGTCACAGGTCTTGAACCGTTACAC
TT------TCGCATCGA---
CTCTATCTCACTTGAGGCGAACAAACACTCCTATCGGGAGAGACGGCAAGCTTGCTAAGCCTCGAC
AACTTCACAACACTCATTGGGGTCTGGTCTGTCCGGCTGAGACGCCTGAAGGCCAGGCTTGTGGAC
TGGTGAAGAACTTGTCGCTCATGTGCTCCATCAGCGTGGGTACATCAACGGATCCTATCGTGGACT
ACATGATTACCAGAAACATGGAGGTCTTGGAGGAATACGAACCCATGCGATACCCTAACGCTACC
AAGATCTTCCTCAATGGTTCTTGGATTGGTGTACATCAGGATCCCAAGACTCTCGTCAGAGATATCC
AGATGCTTCGTCGGGCCAACCAAATTCCCTCTGAAGTTTCTTTGGTCCGCGATATCCGTGATCGTGA
GTTCAAGATCTTCTCGGATGCCGGTCGTGTCATGCGTCCCTTGTTCGTCGTCCACCAAGAGGAT------
---A---
CTGAGCAAGGCGTTGCTAAGGGAACATTGGCCCTTACCAAAGACATGATCCAGCGACTAGAGGCG
GACGTCGATCTAAATCCCGACAGCGAAGAGTACTTCGGCTGGCAAGGCCTGGTCAACGAGGGTGT
AATTGAGTTCCTCGATGCGGAGGAAGAGGAGACGGCTATGATTTGCATGACACCAGAAGATTTGG
AAGTCTACCGCGCGGCCAAGCTTGGGTATGATGTGGTTCAGGACAACGGT------
GATGAGATTAATAAACGACTCAAGACGAAGATAAACCCCACGACGCACATGTACACGCATTGCGA
GATTCATCCCAGCATGCTTCTCGGCAT--
CCCCTGAACGCGTCCACTCAACCCCTGATTTTCTGCCCCTCACGCACAAAAACC---ACCACAGCTCC-
TGCAGCTGTTTTCGCTACGATGTCAACAAACCAAAAGACAGTGGACATAGGAATCAAAGCTAACA-
--GATGTTTGTCTCAATAGGTTCACCTCCAGACCGGCCAATGCGTAAGTACAACGAT----
CACGACCGCGGACGAAGCAACGCGCTGGATTATAGAGGGGCTCACAC--
AAGCTGCAGGGTAACCAAATCGGTGCTGCTTTCTGGCAAACCATCTCTGGCGAGCACGGTCTCGAC
AGCAATGGCGTGTAAGTGTCTGAGTTGTCAATTCTGATGCCAAGAAAAGAAGCTAACCACCAATAA
CTAGCTACAACGGAACCTCTGAGCTCCAGCTCGAGCGCATGAGCGTCTACTTCAATGAGGTATGCA
GTAATCGAAATCAAGGGTGGCGATGCGAGGACAGTCACTAATCACCCTAACCTACACAGGCTTCTG
GCAACAAGTATGTCCCTCGCGCCGTCCTCGTCGATCTCGAGCCCGGTACCATGGATGCCGTCCGTG
CTGGTCCCTTCGGCCAGCTTTTCCGACCTGACAACTTCGTCTTCGGTCAATCCGGTGCCGGAAACAA
CTGGGCCAAGGGTCACTACACTGAGGGTGCTGAGCTTGTTGACAACGTCCTTGATGTCGTTCGTCG
TGAGGCTGAGGGTTGCGACTGCCTCCAGGGTTTCCAGATTACCCACTCCCTCGGTGGTGGTACCGG
TGCCGGTATGGGTACCCTGTTGATCTCCAAGATCCGTGAGGAGTTCCCCGACCGCATGATGGCCAC
CTTCTCCGTCGTTCCTTCCCCTAAGGTCTCCGACACCGTCGTCGAGCCCTACAACGCCACCCTGTCC
GTCCACCAGCTGGTCGAGAACTCGGACGAGACCTTCTGCATTGACAACGAGGCTCTGTACGACATC
TGCATGCGTACCCTGAAGCTATCCAACCCCTCGTATGGTGACCTGAACCACCTGGTCTCTGCCGTCA
TGTCCGGTGTTACCACCTGTTTGCGATTCCCCGGCCAGCTGAACTCTGACCTACGCAAGCTCGCCGT
GAACATGGTTCCTTTCCCTCGTCTTCACTTCTTCATGGTCGGCTTCGCCCCTCTGACCAGCCGTGGC
GCTCACTCCTTCCGCGCCGTCACCGTTCCCGAGTTGACTCAGCAGATGTTCGACCCCAAGAACATG
ATGGCTGCCTCTGACTTCCGTAACGGCCGCTACCTAACTTGCTCTGCCATCTTGTAAGAAAGCCCTT
GCGAATTATGCGCTATTTCTGGTTTGCTAACTTGTACTTTTCAGCCGTGGCAAGGTCTCC-

>411

-CATTACTGAGTTATCCAAACTCCAAACCCTTTGTGAACCTTACCGTCGTTGCCTCGGCGTG-
AGCGCGGCTACCCG--GTAGCTACCCTGT-AGCCGGT-
TCACGGCCCGCCGAAGGACAGCTAAACTCTTGTAATACCACTGTATCTCTGAATTGTTAA-
CTAAATAAGTTAAAACTTTCAACAACGGATCTCTTGGTTCTGGCATCGATGAAGAACGCAGCGAAA
TGCGATAAGTAATGTGAATTGCAGAATTCAGTGAATCATCGAATCTTTGAACGCACATTGCGCCCA
TTAGTATTCTAGTGGGCATGCCTATTCGAGCGTCATTTCAACCCTTAAGCCAATGCTGCTTAGTGTT
GGGAGCATACCCTCCGGGGGGTATCTCCTTAAAGTTAGTGGCGGAGTTAGGGTACACTCTCAGCGT
AGTAATTTCTCTCGCTCGGGTAGTGGCCCTGGCTGCTTGCCGTTAAGCCTTTTTTTCTTCTAGTGGTT
GACCTCGGATTAGGTAGGAATACCCGCTGAACTTAAGCATATGAAGCGGCAACAGCTCAAA-
TTTGAAATCTGGCC----CTC-----GTGGTCCGAGTTGTAATTTGTAGAGGATGCTTTTGGTGCGGT-
GCCTTCCGAGTTCCCTGGAACGGGACGCCAGAGAGGGTGAGAGCCCCGTACGGTTGGA-

CGCCTAGCCTATATATAGCTCCTTCGACGAGTCGAGTAGTTTGGGAATGCTGCTCTAAATGGGAGG
TAAATTTCTTCTAAAGCTAAATACCGGCCAGAGACC-
GATAGCGCACAAGTAGAGTGATCGAAAGATGAAAAGCACTTTGAAAAGAGGGTTAAATAGCACGT
GAAATTGTTGAAAGGGAAGCG-TTTACGACCAGACCTTCTCCAGGGGGATCATCAGGTGTTT--
TCACCTGTGCACTTCCCCTGG--TTTAGGCCAGCATCGGTTTC---
TTTAGGGGGATAAAAGCTCGGGGAACGTAGCTC----CTTAGGGAGTGTT-
ATAGCCCCTTGCGTAATACCCCTCG-GGGGACCGAGGACCGCGCT-CT----
GCAAGGATGCTGGCGTAATGGTCGTCAACGACCCGTCTTGAAACACGGACCAAGGAGTCGAACAT
TTGTGCGAGTGTTTGGGTG--TTAAACCCTCACGCGTAATGAAAGTGAACGGAGGTGAGAGCCCTT--
--AC----GGGTGCATCATCGACCGATCCTGATG-
TCTTCGGATGGATTTGAGTAAGAGCATAACTGTTCGGACCCGAAAGATGGTGAACTATGCGTGGAT
AGGGTGAAGCCAGAGGAAACTCTGGTGGAGGCTCGCAGCGGTTCTGACGTGCAAATCGATCGTCA
AATCTGCGCATGGGGGCGAAAGACTTATCGA-
ACCATCTAGTAGCTGGTTACCGCCGAAGTTTCCCTCAGGATAGCAGTGT--TG-
TCTTCAGTTTTATGAGGTAAAGCGAATGATTAGGGACTCGGGGGCGCTATTTTGCCTTCATCCATTC
TCAAACTTTAAATATGTAAGAAGCCCTTGTTACTTAGTTGAACGTGGGCATTCGAATGTACCAACA
CTAGTGGGCCATTTTTGGTAAGCAGAACTGGCGATGCGGGATGAACCGAACGCGGGGTTAAGGTG
CCAGAGTGGACGCTCATCAGACACCACAAAAGGTGTTAGTACATCCAGACAGCAGGACGGTGGCC
ATGGAAGTCGGAATCCGCTAAGGACTGTGTAACAACTCACCTGCCGAATGTACTAGCCCTGAAAAT
GGATGGCGCTCAAGCGTCT-CACCCATACCTCGCCCTTAGGGTAGGATCGATGCCCTAAGG------------
----
GATTGAGCTAGCAGCCAAACCCGCTATCATCACAAATGGACTGAAGTACTCTCTTGCCACAGGTAA
CTGGGGTGATCAGAAGAAGGCGGCGAGCTCGACTGCCGGTGTGTCACAGGTCTTGAACCGTTACAC
TT------TCGCATCGA---
CTCTATCTCACTTGAGGCGAACAAACACTCCTATCGGGAGAGACGGCAAGCTTGCTAAGCCTCGAC
AACTTCACAACACTCATTGGGGTCTGGTCTGTCCGGCTGAGACGCCTGAAGGCCAGGCTTGTGGAC
TGGTGAAGAACTTGTCGCTCATGTGCTCCATCAGCGTGGGTACATCAACGGATCCTATCGTGGACT
ACATGATTACCAGAAACATGGAGGTCTTGGAGGAATACGAACCCATGCGATACCCTAACGCTACC
AAGATCTTCCTCAATGGTTCTTGGATTGGTGTACATCAGGATCCCAAGACTCTCGTCAGAGATATCC
AGATGCTTCGTCGGGCCAACCAAATTCCCTCTGAAGTTTCTTTGGTCCGCGATATCCGTGATCGTGA
GTTCAAGATCTTCTCGGATGCCGGTCGTGTCATGCGTCCCTTGTTCGTCGTCCACCAAGAGGAT------
---A---
CTGAGCAAGGCGTTGCTAAGGGAACATTGGCCCTTACCAAAGACATGATCCAGCGACTAGAGGCG
GACGTCGATCTAAATCCCGACAGCGAAGAGTACTTCGGCTGGCAAGGCCTGGTCAACGAGGGTGT
AATTGAGTTCCTCGATGCGGAGGAAGAGGAGACGGCTATGATTTGCATGACACCAGAAGATTTGG
AAGTCTACCGCGCGGCCAAGCTTGGGTATGATGTGGTTCAGGACAACGGT------
GATGAGATTAATAAACGACTCAAGACGAAGATAAACCCCACGACGCACATGTACACGCATTGCGA
GATTCATCCCAGCATGCTTCTCGGCAT--
CCCCTGAACGCGTCCACTCAACCCCTGATTTCTGCCCCTCACGCACAAAAACC---ACCACAGCTCC-
TGCAGCTGTTTTCGCTACGATGTCAACAAACCAAAAGACAGTGGACATAGGAATCAAAGCTAACA-
--GATGTTTGTCTCAATAGGTTCACCTCCAGACCGGCCAATGCGTAAGTACAACGAT----
CACGACCGCGGACGAAGCAACGCGCTGGATTATAGAGGGGCTCACAC--
AAGCTGCAGGGTAACCAAATCGGTGCTGCTTTCTGGCAAACCATCTCTGGCGAGCACGGTCTCGAC
AGCAATGGCGTGTAAGTGTCTGAGTTGTCAATTCTGATGCCAAGAAAAGAAGCTAACCACCAATAA
CTAGCTACAACGGAACCTCTGAGCTCCAGCTCGAGCGCATGAGCGTCTACTTCAATGAGGTATGCA
GTAATCGAAATCAAGGGTGGCGATGCGAGGACAGTCACTAATCACCCTAACCTACACAGGCTTCTG
GCAACAAGTATGTCCCTCGCGCCGTCCTCGTCGATCTCGAGCCCGGTACCATGGATGCCGTCCGTG
CTGGTCCCTTCGGCCAGCTTTTCCGACCTGACAACTTCGTCTTCGGTCAATCCGGTGCCGGAAACAA
CTGGGCCAAGGGTCACTACACTGAGGGTGCTGAGCTTGTTGACAACGTCCTTGATGTCGTTCGTCG
TGAGGCTGAgGGTTGCGACTGCCTCCAGGGTTTCCAGATTACCCACTCCCTCGGTGGTGGTACCGGT
GCCGGTATGGGTACCCTGTTGATCTCCAAGATCCGTGAGGAGTTCCCCGACCGCATGATGGCCACC
TTCTCCGTCGTTCCTTCCCCTAAGGTCTCCGACACCGTCGTCGAGCCCTACAACGCCACCCTGTCCG
TCCACCAGCTGGTCGAGAACTCGGACGAGACCTTCTGCATTGACAACGAGGCTCTGTACGACATCT
GCATGCGTACCCTGAAGCTATCCAACCCCTCGTATGGTGACCTGAACCACCTGGTCTCTGCCGTCAT
GTCCGGTGTTACCACCTGTTTGCGATTCCCCGGCCAGCTGAACTCTGACCTACGCAAGCTCGCCGTG
AACATGGTTCCTTTCCCTCGTCTTCACTTCTTCATGGTCGGCTTCGCCCCTCTGACCAGCCGTGGCGC
TCACTCCTTCCGCGCCGTCACCGTTCCCGAGTTGACTCAGCAGATGTTCGACCCCAAGAACATGAT

GGCTGCCTCTGACTTCCGTAACGGCCGCTACCTAACTTGCTCTGCCATCTTGTAAGAAAGCCCTTGC
GAATTATGCGCTATTTCTGGTTTGCTAACTTGTACTTTTCAGCCGTGGCAAGGTCTCC-

>319

-CATTACTGAGTTATCCAAACTCCAAACCCTTTGTGAACCTTACCGTCGTTGCCTCGGCGTG-
AGCGCGGCTACCCGGTAGCTACCCT-----------------------------GTAGCTACCCTGT-AGCCGGT-
TCACGGCCCGCCGAAGGACAGCTAAACTCTTGTAATACCACTGTATCTCTGAATTGTTAA-
CTAAATAAGTTAAAACTTTCAACAACGGATCTCTTGGTTCTGGCATCGATGAAGAACGCAGCGAAA
TGCGATAAGTAATGTGAATTGCAGAATTCAGTGAATCATCGAATCTTTGAACGCACATTGCGCCCA
TTAGTATTCTAGTGGGCATGCCTATTCGAGCGTCATTTCAACCCTTAAGCCAATGCTGCTTAGTGTT
GGGAGCATACCCTCCGGGGGGTATCTCCTTAAAGTTAGTGGCGGAGTTAGGGTACACTCTCAGCGT
AGTAATTTCTCTCGCTCGGGTAGTGGCCCTGGCTGCTTGCCGTTAAGCCTTTTTTTCTTCTAGTGGTT
GACCTCGGATTAGGTAGGAATACCCGCTGAACTTAAGCATATGAAGCGGCAACAGCTCAAA-
TTTGAAATCTGGCC----CTC-----GTGGTCCGAGTTGTAATTTGTAGAGGATGCTTTTGGTGCGGT-
GCCTTCCGAGTTCCCTGGAACGGGACGCCAGAGAGGGTGAGAGCCCCGTACGGTTGGA-
CGCCTAGCCTATATATAGCTCCTTCGACGAGTCGAGTAGTTTGGGAATGCTGCTCTAAATGGGAGG
TAAATTTCTTCTAAAGCTAAATACCGGCCAGAGACC-
GATAGCGCACAAGTAGAGTGATCGAAAGATGAAAAGCACTTTGAAAAGAGGGTTAAATAGCACGT
GAAATTGTTGAAAGGGAAGCG-TTTACGACCAGACCTTCTCCAGGGGGATCATCAGGTGTTT--
TCACCTGTGCACTTCCCCTGG--TTTAGGCCAGCATCGGTTTC---
TTTAGGGGGATAAAAGCTCGGGGAACGTAGCTC----CTTAGGGAGTGTT-
ATAGCCCCTTGCGTAATACCCCTCG-GGGGACCGAGGACCGCGCT-CT----
GCAAGGATGCTGGCGTAATGGTCGTCAACGACCCGTCTTGAAACACGGACCAAGGAGTCGAACAT
TTGTGCGAGTGTTTGGGTG--TTAAACCCTCACGCGTAATGAAAGTGAACGGAGGTGAGAGCCCTT--
--AC----GGGTGCATCATCGACCGATCCTGATG-
TCTTCGGATGGATTTGAGTAAGAGCATAACTGTTCGGACCCGAAAGATGGTGAACTATGCGTGGAT
AGGGTGAAGCCAGAGGAAACTCTGGTGGAGGCTCGCAGCGGTTCTGACGTGCAAATCGATCGTCA
AATCTGCGCATGGGGGCGAAAGACTTATCGA-
ACCATCTAGTAGCTGGTTACCGCCGAAGTTTCCCTCAGGATAGCAGTGT--TG-
TCTTCAGTTTTATGAGGTAAAGCGAATGATTAGGGACTCGGGGGCGCTATTTTGCCTTCATCCATTC
TCAAACTTTAAATATGTAAGAAGCCCTTGTTACTTAGTTGAACGTGGGCATTCGAATGTACCAACA
CTAGTGGGCCATTTTTGGTAAGCAGAACTGGCGATGCGGGATGAACCGAACGCGGGGTTAAGGTG
CCAGAGTGGACGCTCATCAGACACCACAAAAGGTGTTAGTACATCCAGACAGCAGGACGGTGGCC
ATGGAAGTCGGAATCCGCTAAGGACTGTGTAACAACTCACCTGCCGAATGTACTAGCCCTGAAAAT
GGATGGCGCTCAAGCGTCT-CACCCATACCTCGCCCTTAGGGTAGGATCGATGCCCTAAGG-----------
----
GATTGAGCTAGCAGCCAAACCCGCTATCATCACAAATGGACTGAAGTACTCTCTTGCCACAGGTAA
CTGGGGTGATCAGAAGAaGGCGGCGAGCTCGACTGCCGGTGTGTCACAGGTCTTGAACCGTTACAC
TT------TCGCATCGA---
CTCTATCTCACTtGAGGCGAACAAACACTCCTATCGGGAGAGACGGCAAGCTTGCTAAGCCTCGAC
AACTTCACAACACTCATTGGGgTCTGGTCTGTCCGGCTGAGACGCcTGAAGGCCAGGCTTGTGGACT
GGTGAAGAACTTGTCGCTCATGTGCTCCATCAGCGTGGGTACATCAACGGATCCTATCGTGGACTA
CATGATTACCAGAAACATGGAGGTCTTGGAGGAATACGAACCCATGCGATACCCTAACGCTACCA
AGATCTTCCTCAATGGTTCTTGGATTGGTGTACATCAGGATCCCAAGACTCTCGTCAGAGATATCCA
GATGCTTCGTCGGGCCAACCAAATTCCCTCTGAAGTTTCTTTGGTCCGCGATATCCGTGATCGTGAG
TTCAAGATCTTCTCGGATGCCGGTCGTGTCATGCGTCCCTTGTTCGTCGTCCACCAAGAGGAT--------
--A---
CTGAGCAAGGCGTTGCTAAGGGAACATTGGCCCTTACCAAAGACATGATCCAGCGACTAGAGGCG
GACGTCGATCTAAATCCCGACAGCGAAGAGTACTTCGGCTGGCAAGGCCTGGTCAACGAGGGTGT
AATTGAGTTCCTCGATGCGGAGGAAGAGGAGACGGCTATGATTTGCATGACACCAGAAGATTTGG
AAGTCTACCGCGCGGCCAAGCTTGGGTATGATGTGGTTCAGGACAACGGT------
GATGAGATTAATAAACGACTCAAGACGAAGATAAACCCCACGACGCACATGTACACGCATTGCGA
GATTCATCCCAGCATGCTTCTCGGCAT-------------------------------------
CCCCTGAACGCGTCCACTCAACCCCTGATTTTCTGCCCCTCACGCACAAAAACC---ACCACAGCTCC-
TGCAGCGGTTTCTGCTACGATGTCAACAAACCAGAAGACGACGGACATAGGAATCAAAGCTAATA-
--TATGTTTGTCTCAATAGGTTCACCTCCAGACCGGCCAATGCGTAAGTACAATGAT----
CACGACCGCAGACGAAGCAACGCGCTGGATTATAGAGGGGCTCACAC--

AAGCTGCAGGGTAACCAAATCGGTGCTGCTTTCTGGCAAACCATCTCTGGCGAGCACGGTCTCGAC
AGCAATGGCGTGTAAGTGTCTGAGTTGTCAATTCTGATGCCAAGAAAAGGAGCTAACCACCAATAA
CTAGGTACAACGGAACCTCTGAGCTCCAGCTCGAGCGCATGAGCGTCTACTTCAATGAGGTATGCA
GTAATCGAAATCAAGGGTGGCGATGCAAGGACAGTCACTAATCACCCTAACCTACACAGGCTTCTG
GCAACAAGTATGTCCCTCGCGCCGTCCTCGTCGATCTCGAGCCCGGTACCATGGATGCCGTCCGTG
CTGGTCCCTTCGGCCAGCTTTTCCGACCTGACAACTTCGTCTTCGGTCAATCCGGTGCCGGAAACAA
CTGGGCCAAGGGTCACTACACTGAGGGTGCTGAGCTTGTTGACAACGTCCTTGATGTCGTTCGTCG
TGAGGCTGAgGGTTGCGACTGCCTCCAGGGTTTCCAGATCACCCACTCCCTCGGTGGTGGTACCGGT
GCCGGTATGGGTACCCTGTTGATCTCCAAGATCCGCGAGGAGTTCCCCGACCGCATGATGGCCACC
TTCTCCGTCGTTCCTTCCCCTAAGGTCTCCGACACCGTCGTCGAGCCCTACAACGCTACCCTGTCCG
TCCACCAGCTGGTCGAGAACTCGGACGAGACCTTCTGCATTGACAACGAGGCTCTGTACGACATCT
GCATGCGTACCCTGAAGCTATCCAACCCCTCGTATGGTGACCTGAACCACCTGGTCTCTGCCGTCAT
GTCCGGTGTTACCACCTGTTTGCGATTCCCCGGCCAGCTGAACTCTGACCTACGCAAGCTCGCCGTG
AACATGGTTCCTTTCCCTCGTCTTCACTTCTTCATGGTCGGCTTCGCCCCTCTGACCAGCCGTGGCGC
TCACTCCTTCCGCGCCGTCACCGTTCCCGAGTTGACTCAGCAGATGTTCGACCCCAAGAACATGAT
GGCTGCCTCTGACTTCCGTAACGGCCGCTACCTAACTTGCTCTGCCATCTTGTAAGAAAGCCCTTGC
GAATTATGCGCTATTTCTGATTTGCTAACTTGTACTTTTCAGCCGTGGCAAGGTCTCC-

>Hypoxylon_lignicola_MFLUCC160926

--CCTTACCGTCGTTGCCTCGGCGTG-
AGCGCGGCTACCCGGGAGCTACCCT------------------------------GTAGCTACCCTGT-AGCCGGC-
TTACGGCCCGCCGAAGGACAGCTAAACTCTTGTAATACCACTGTATCTCTGAATTAATAA-
CTAAATAAGTTAAAACTTTCAACAACGGATCTCTTGGTTCTGGCATCGATGAAGAACGCAGCGAAA
TGCGATAAGTAATGTGAATTGCAGAATTCAGTGAATCATCGAATCTTTGAACGCACATTGCGCCCA
TTAGTATTCTAGTGGGCATGCCTATTCGAGCGTCATTTCAACCCTTAAGCCTGTGTTGCTTAGCGTT
GGGAGCATACCCTCCAGGGGGTATCTCCTTAAAGTCAGTGGCGGAGTTAGGGTACACTCTCAGCGT
AGTAATTTCTCTCGCTCGGGTGGTGGCCCTGGCTGCTTGCCGTTAAGCCTTTTTT--
TTCTAGTGGTTGACCTCGGATTAGGTAGGAATACCCGCTGAACTTAAGCATATGAAGCGGCAACAG
CTCAAA-TTTGAAATCTGGCC----CTC-----
GTGGTCCGAGTTGTAATTTGTAGAGGATGCTTTTGGTGCGGT-
GCCTTCCGAGTTCCCTGGAACGGGACGCCAGAGAGGGTGAGAGCCCCGTACGGTTGGA-
CGCCTAGCCTTTATATAGCTCCTTCGACGAGTCGAGTAGTTTGGGAATGCTGCTCTAAATGGGAGG
TAAATTTCTTCTAAAGCTAAATACCGGCCAGAGACC-
GATAGCGCACAAGTAGAGTGATCGAAAGATGAAAAGCACTTTGAAAAGAGGGTTAAATAGCACGT
GAAATTGTTGAAAGGGAAGCG-TTTACGACCAGACTTTCTCCAGGGGGATCATCAGGTGTTC--
TCACCTGTGCACTTCCCCTGG--TTTAGGCCAGCATCGGTTTC---
CTTAGGGGGATAAAGGCTTGGGGAAAGTAGCTC----CTTAGGGAGTGTT-
ATAGCCCCTTGCGTAATACCCCTCG-GGGGACCGAGGACCGCGCT-CT----
GCAAGGATGCTGGCGTAATGGTCGTCAACGACCCGTCTTGAAACACGGACCAAGGAGTCGAACAT
TTGTGCGAGTGTTTGGGTG--TTAAACCCTCACGCGTAATGAAAGTGAACGGAGGTGAGAGCCCTT--
--AC----GGGTGCATCATCGACCGATCCTGATG-
TCTTCGGATGGATTTGAGTAAGAGCATAACTGTTCGGACCCGAAAGATGGTGAACTATGCGTGGAT
AGGGTGAAGCCAGAGGAAACTCTGGTGGAGGCTCGCAGCGGTTCTGACGTGCAAATCGATCGTCA
AA---

ACGGTGTATTGACTCGAACAGACGTTTCCAGATTGAGCTAGCAGCCAAGCCCGCTATCATCACGAA
TGGGCTGAAATATTCTCTTGCCACAGGTAATTGGGGTGATCAGAAGAAGGCGATGAGCTCGACTGC
CGGTGTGTCGCAGGTCTTAAACCGTTATACCT------TCGCATCGA---
CTCTATCTCACTTGAGGCGAACAAATACTCCTATCGGGAGAGACGGGAAGCTTGCCAAGCCTCGAC
AACTTCACAATACTCACTGGGGTTTGGTCTGTCCGGCTGAGACGCCTGAAGGCCAGGCTTGTGGAC
TGGTGAAGAACTTGTCGCTCATGTGTTCCATCAGCGTGGGCACATCAACGGATCCTATTGTGGACT
ACATGATTACGAGAAACATGGAAGTCCTGGAGGAATACGAACCGATGCGATACCCTAACGCTACC
AAGATCTTCCTCAATGGTTCTTGGATCGGTGTACATCAGGATCCCAAGACTCTCGTCAGAGATATCC
AGATGCTTCGTCGGGCCAACCAAATTCCCTCTGAAGTTTCCTTGGTCCGCGATATCCGTGATCGTGA
GTTCAAGATCTTCTCGGATGCCGGTCGCGTCATGCGCCCTTTGTTCGTCGTACACCAAGAGGAT------

---A---
CTGAGCAAGGCATTGCTAAGGGAACATTGGCCCTTACCAAAGACATGATCCAGCGACTAGAGGCG
GACGTTGATCTAGATCCCGACAGCGAAGAATATTTCGGCTGGCAAGGCCTGGTCAACGAGGGTGTA
ATTGAGTTCCTCGATGC--

>Hypoxylon_phuphaphetense_TBRC16277

--------GAGTTATCTAAACTCCAAACCCTTTGTGAACTTTACCGTCGTTGCCTCGGCGTG-
AGCGCGGCTACCCTGTACCTACCCTGTAC----TTACCCTATAGGTGCCTACCCGGTAGCTACCCTGT-
AGCCGGC-
CCACAGCCCGCCGAAGGACCGCCAAACTCTTGTTTTACCACTGTATCTCTGAACACCTAA-
CTTAATGAGTTAAAACTTTCAACAACGGATCTCTTGGTTCTGGCATCGATGAAGAACGCAGCGAAA
TGCGATAAGTAATGTGAATTGCAGAATTCAGTGAATCATCGAATCTTTGAACGCACATTGCGCCCG
CCAGCATTCTGGCGGGCATGCCTATTCGAGCGTCATTTCGACCCTTAAGCCCCTGTTGCTTAGCGTT
GGGAGTCTACGTCC-
ACAGCGTAGTTCCTTAAAGTTAGTGGCGGAGTTAGGGCACACCCTCAGCGTAGTAACATCTCTCGC
TCGTGTGGTGTCCCTAGCTGCTGGCCGTTAAACCTCCCATAATTCTAGTGGTTGACCTCGGATTAGG
TAGGAATACCCGCT-------------TGAAGCGGCAACAGCTCAAA-TTTGAAATCTGGCC----CTC-----
GTGGTCCGAGTTGTAATTTGTAGAGGATGCTTTTGGTGCGGC-
GCCTTCCGAGTTCCCTGGAACGGGACGCCGGAGAGGGTGAGAGCCCCGTACGGTTGGA-
CGCCTAGCCTTTGTATAGCTCCTTCGACGAGTCGAGTAGTTTGGGAATGCTGCTCTAAATGGGAGG
TAAATTTCTTCTAAAGCTAAATACCGGCCAGAGACC-
GATAGCGCACAAGTAGAGTGATCGAAAGATGAAAAGCACTTTGAAAAGAGGGTTAAATAGCACGT
GAAATTGTTGAAAGGGAAGCG-TTTGCGACCAGACCTTTTCCAGGCGGATCATCCGGTGTTC--
TCACCGGTGCACTTCGCCTGG--TTTAGGCCAGCATCGGTTTT---
CTTAGGGGGATAAAGGCCGGGGGAAAGTAGCTC----CTTCTGGAGTGTT-
ATAGCCCCTGACGTAATACCCTTTG-GGGGACCGAGGACCGCGCT-CT----
GCAAGGATGCTGGCGTAATGGTCGTCAACGACCCGTCTTGAAACACGGACCAAGGAGTCGAACAT
TTATGCGAGTGTTTGGGTG--TTAAACCCTCACGCGTAATGAAAGTGAACGGAGGTGAGAGCCCTT--
--AC----GGGTGCATCATCGACCGATCCTGATG-
TATTCGGATGGATTTGAGTAAGAGCATAACTGTTCGGACCCGAAAGATGGTGAACTATGCGTGGAT
GGGGTGAAGCCAGAGGAAACTCTGGTGGAGGCTCGCAGCGGTTCTGACGTGCAAATCGATCGTCA
AATCTGCGCATGGGGGCGAAAG--

AGCTCGACTGCCGGTGTGTCGCAGGTTCTGAATCGATATACCT------TCGCCTCGA---
CCCTCTCTCACTTGAGACGAACAAACACTCCTATCGGGCGAGACGGAAAACTTGCAAAGCCCCGAC
AGCTTCACAACACTCACTGGGGTCTTGTCTGTCCGGCTGAGACGCCTGAAGGTCAGGCCTGTGGGC
TTGTGAAGAATCTGTCACTGATGTGCTCCATCAGCGTGGGTACATCTACGGATCCTATCGTGGACTA
TATGATTACTAGGAATATGGAAGTCCTAGAGGAATATGAACCCATGAGGTACCCCAACGCCACCA
AGATTTTCCTCAATGGTTCTTGGATCGGTGTGCATCAGGATGCCAAGACTCTTGTCAGAGATATCCA
GGCGCTTCGTCGAGCCAATCAAATTCCCTCCGAAGTATCCTTGGTCCGCGATATCCGTGATCGTGA
GTTCAAGATCTTCTCAGACGCTGGTCGTGTCATGCGCCCCTTGTTTGTGGTGAACCAAGAGGAT------
----

AATCCCGATGAGCACATTGCTAAGGGTACATTGGCCCTTACCAAAGAGATGATCCAGCGGCTAGAG
GCGGATGTTGACCTGGATCCTGAAAGCGAGGAGTACTATGGCTGGCAAGGCCTGGTCAACGACGG
TGTAATCGAGTTCCTCGATGCGGAAGAAGAGGAAACGGCCATGATTTGCATGACACCGGAAGACT
TGGAGATTTATCGCATGACGAAGGCCGGGTACGACGTGGTCCAGGATAACGGC------
GATGAGGTTAACAAGCGACTCAAGACCAAGGTGAATCCGTCGACGCACATGTATACGCATTGCGA
AATCCATCCCAGCATGCTTCTAGGCATCTGCGCCAGCATTATTCCTTTCCCCGACCACAACCAGTCG
CCCCTGAACGCGTCCTCTCAACCCCTGATTTCTGCCCCTCACACGCACAAAAC---
ACCACAGCATCATGCAGCTCTTTTCGCTACGATGCCATCACACTGGACGACAAAAGAGATGAAAAT
GGAAGCTAACA---
GATGTTTGTCTCGATAGGTTCACCTTCAGACCGGCCAATGCGTAAGTACAGCAAC----
CACGACCACAAACGAAGCAACGCGCTGGAACATGGAGGGGCTCACAC--
AAACTACAGGGTAACCAAATCGGTGCTGCTTTCTGGCAAACCATCTCTGGCGAGCACGGTCTCGAC
AGCAATGGCGTGTAAGTACATGAGCTGGCCATTCTGATGCGAGGAGCAGGGACTAACCACCAATA
ATTAGCTACAACGGAACCTCTGAGCTCCAGCTCGAGCGCATGAGCGTCTACTTCAACGAGGTACGC
AAGAATCGAAACCAGGGATGACGATGCAGGGCCAGGTACTAACCACCCTAACCTGCACAGGCTTC
TGGCAACAAGTACGTCCCTCGCGCCGTCCTCGTCGATCTCGAGCCCGGTACCATGGACGCCGTCCG
TGCTGGTCCCTTCGGCCAGCTCTTCCGACCTGACAACTTCGTCTTCGGCCAGTCTGGTGCTGGAAAC
AACTGGGCCAAGGGTCACTACACTGAGGGTGCTGAGCTTGTTGACAACGTTCTTGACGTCGTCCGC
CGTGAGGCTGAGGGCTGCGACTGCCTCCAGGGTTTCCAGATCACCCACTCCCTTGGTGGTGGTACT
GGTGCCGGTATGGGTACTCTCCTCATCTCCAAGATCCGCGAGGAGTTCCCCGACCGCATGATGGCC
ACCTTCTCCGTCGTTCCTTCCCCTAAGGTTTCCGACACCGTTGTTGAGCCCTACAACGCCACTCTCTC
CGTCCACCAGCTGGTCGAGAACTCCGATGAGACCTTCTGCATTGACAACGAGGCTCTGTATGACAT
CTGCATGCGCACCCTGAAGCTATCCAACCCCTCGTACGGTGACCTGAACCACCTGGTCTCCGCCGT
CATGTCCGGTGTCACTACCTGCTTGCGCTTCCCCGGTCAGCTAAACTCTGACCTGCGCAAGCTCGCC
GTGAACATGGTTCCTTTCCCTCGTCTCCACTTCTTCATGGTCGGCTTCGCTCCTTTGACCAGCCGTGG
TGCCCACTCCTTCCGCGCCGTCACCGTTCCCGAGTTGACTCAGCAGATGTTCGACCCCAAGAACAT
GATGGCCGCCTCTGACTTCCGCAACGGTCGTTACCTGACTTGCTCTGCCATCTTGTAAGACGCCCCT
CACGAATTATGTGCCATTATCGGAATGCTAACTTGTACTCTCTAGCCGTGGCAAGGTCTCGA

>Hypoxylon_hinnuleum_MUCL3621

-CATTACAGAGTTACCTAAACTCCAAACCCTTTGTGAACCTTACCACCGTTTCCTCGGCGCG-
AGCGCGGCTACCCGGGAGTTACCCTGCAG----CTACCCTGTAGG----------GGACCTACCCTGT-
AGCCGGC-
CCACGGCCCGCCGAAGGACCGCTAAACTCTTGTTTAACCACTGTATCTCTGAACACCTAACTGAAA
TAAGTTAAAACTTTCAACAACGGATCTCTTGGTTCTGGCATCGATGAAGAACGCAGCGAAATGCGA
TAAGTAATGTGAATTGCAGAATTCAGTGAATCATCGAATCTTTGAACGCACATTGCGCCCGCTAGT
ATTCTGGCGGGCATGCCTATTCGAGCGTCATTTCGACCCTTAAGCCCCTGTTGCTTAGCGTTGGGAC
TCTGCGCCTCAGGGCGCAGTTCCCGAAAGTTAGTGGCGGAGTTAGGGTACACTCTCAGCGTAGTAA
TTCTTCTCGCTCGTGTGGTGGCCCTGGCTGCTGGCCGTTAAACACCCCCCCCCCCAAGTGGTTGACC
TCGGATTAGGTAGGAATACCCGCTGAACTTAAGCATATGAAGCGGCAACAGCTCAAA-
TTTGAAATCTGGCC----CTC-----GCGGTCCGAGTTGTAATTTGTAGAGGATGCTTTGGGCGCGGC-
GCCTTCCGAGTTCCCTGGAACGGGACGCCAGAGAGGGTGAGGGCCCCGTACGGTTGGA-
CGCCTAGCCTATGTATAGCTCCTTCGACGAGTCGAGTAGTTTGGGAATGCTGCTCTAAATGGGAGG
TAAATTTCTTCTAAAGCTAAATACCGGCCAGAGACC-
GATAGCGCACAAGTAGAGTGATCGAAAGATGAAAAGCACTTTGAAAAGAGGGTTAAATAGCACGT
GAAATTGTTGAAAGGGAAGCG-TTTGCGACCAGACCTTCTCCGGGGGGATCATCCGGTGTTC--
TCACCGGTGCACTCCCCTCGG--TCTAGGCCAGCATCGGTTTC---
CTTAGGGGGATAAAGGCCTGGGGAACGTAGCTC----CTTCGGGAGTGTT-
ATAGCCCCTTGCGTAATACCCCTCG-GGGGACCGAGGACCGCGCT-TCG---
GCAAGGATGCTGGCGTAATGGTCGTCAACGACCCGTCTTGAAACACGGACCAAGGAGTCGAACAT
TTGTGCGAGTGTTTGGGTG--TTAAACCCTCACGCGTAATGAAAGTGAACGGAGGTGAGAGCCCTC--
--GC----GGGTGCATCATCGACCGATCCTGATG-
TCTTCGGATGGATTTGAGTAAGAGCATAACTGTTCGGACCCGAAAGATAGTGAACTATGCGTGGAT
AGGGTGAAGCCAGAGGAAACTCTGGTGGAGGCTCGCAGCGGTTCTGACGTGCAAATCGATCGTCA
AATCTGCGCATGGGGGCGAAAGACTAATCGA-
ACTATCTAGTAGCTGGTTACCGCCGAAGTTTCCCTCAGGATAGCAGTGT--TG-
TCTTCAGTTTTATGAGGTAAAGCGAATGATTAGGGACTCGGGGGCGCTATATTGCCTTCATCCATTC

TCAAACTTTAAATATGTAAGAAGCCCTTGTTACTTAGTTGAACGTGGGCATTCGAATGTACCAACA
CTAGTGGGCCATTTTTGGTAAGCAGAACTGGCGATGCGGGATGAACCGAACGCGGGGTTAAGGTG
CCAGAGTGGACGCTCATCAGACACCACAAAAGGTGTTAGTACATCCTGACAGCAGGACTGTGGCC
ATGGAAGTCGGAATCAGCTAAGGACTGTGTAACAACTCACCTGCCGAATGTACTAGCCCTGAAAAT
GGATGGCGCTCAAGCGTCT-CACCCATACCTCGCCCTTAGGGTAGAATCGATGCCCTAAGG------------
----

CTTCCAGATTGAGCTTGCCGCCAAGCCCGCCATCATCACCAATGGGCTGAAGTATTCCCTTGCCAC
GGGTAACTGGGGTGATCAGAAGAAGGCAATGAGCTCGACCGCCGGTGTGTCGCAGGTGTTGAATC
GATACACCT------TCGCCTCGA---
CCCTCTCTCACTTGAGACGAACGAACACTCCCATTGGAAGAGATGGAAAACTCGCGAAGCCCCGAC
AGCTTCACAATACCCACTGGGGCCTTGTCTGTCCGGCTGAGACGCCTGAAGGTCAGGCCTGTGGGC
TTGTGAAGAACTTGTCGCTGATGTGTTCCATCAGCGTGGGAACATCGACGGATCCTATCGTGGACT
ATATGATTACTAGGAATATGGAAGTCTTGGAGGAATATGAACCCATGCGATACCCCAACGCTACTA
AGATCTTCCTCAATGGTTCTTGGATCGGTGTACATCAGGATCCCAAGACGCTCGTCAGAGATATCC
AGGCACTTCGTCGAGCTAATCAGATTCCCTCTGAAGTTTCCTTGGTCCGCGATATCCGTGATCGAGA
GTTCAAGATCTTCTCAGATGCAGGTCGTGTCATGCGCCCCTTGTTTGTCGTAAATCAAGAGGAT------
----

GTCCCCGAGCAGGGCATTGCTAAGGGTACACTGGCTCTTACCAAAGACATGATCCAACGACTAGAG
GCGGACGTTGATCTTGATCCTGAAAGCGAGGAATATTATGGCTGGCAAGGCCTGGTCAACGACGG
AGTTATCGAGTTCCTCGATGCGGAAGAAGAGGAAACGGCCATGATTTGTATGACGCCGGAAGATCT
GGAAATATACCGCATGACTAAGGCTGGGTTCGACGTGATCCAGGACAACAAA------
GATGAGGTTAACAAACGACTCAAGACCAAGGTGAACCCGTCCACGCACATGTATACACATTGCGA
AATCCACCCC---
CCCCTGAACGCGTCCCCTCAACCCCTGATTTCTGCCCCTCACATGCACACAAC---
ATTACAGCATCATACAGCTCTTCTCGCTATCATGGCATCAAACTGGAAAATGAGAGAGAATAGAAT
TACAGCTAACA---
GATCATTTTCTCGATAGGTTCACCTCCAGACCGGCCAATGCGTAAGTACAACAAC----
CACGACCGCGGACGAAGCAACGCGCTGGATCTTAGAGGGGCTCACAC--
AACCTGCAGGGTAACCAAATCGGTGCTGCTTTCTGGCAAACCATCTCTGGCGAGCACGGCCTCGAC
AGCAATGGCGTGTAAGTAGAGTAGCTGCCAATTTTGAGGCCAAGAGTGAGAACTGACCAC--
AACAATAGCTACAACGGAACCTCTGAGCTCCAGCTCGAACGCATGAGCGTCTACTTCAACGAGGTA
TGCAAGGATCAAAACTGGGGATGACGGTGCCAGATCAGGTACTAATCGCCCTACTCTACGCAGGCT
TCCGGTAACAAGTACGTCCCTCGTGCCGTCCTCGTCGATCTCGAGCCCGGTACCATGGACGCCGTC
CGTGCTGGCCCCTTCGGCCAGCTCTTCCGACCTGACAACTTCGTTTTCGGCCAGTCTGGTGCCGGAA
ACAACTGGGCCAAGGGTCATTACACTGAGGGTGCTGAGCTTGTTGACCAGGTCCTTGATGTCGTTC
GCCGTGAGGCTGAGGGCTGCGATTGCCTTCAGGGTTTCCAGATCACCCACTCTCTCGGCGGTGGTA
CTGGTGCCGGTATGGGTACTCTCCTGATCTCCAAGATCCGTGAGGAGTTCCCTGACCGCATGATGG
CCACCTTCTCCGTCGTTCCTTCGCCTAAGGTCTCCGACACTGTCGTTGAGCCCTACAACGCCACCCT
CTCCGTCCACCAGCTGGTCGAGAACTCCGACGAGACCTTCTGTATCGACAACGAGGCCCTGTACGA
CATCTGCATGCGCACCCTGAAGCTATCCAACCCCTCGTACGGTGACCTGAACCACCTGGTCTCCGC
CGTCATGTCCGGTGTCACCACCTGCTTGCGCTTCCCCGGCCAGCTGAACTCCGACCTACGCAAGCTC
GCCGTGAACATGGTTCCTTTCCCTCGTCTCCACTTCTTCATGGTCGGCTTCGCTCCCCTGACCAGCC
GTGGCGCGCACTCCTTCCGCGCCGTCACTGTTCCCGAGTTGACTCAGCAGATGTTCGACCCCAAGA
ACATGATGGCTGCCTCCGACTTCCGCAACGGTCGTTATCTGACCTGCTCCGCCATCTTGTAAGATGC
TCCTCGTAAATTGCGTGATATTACTTGTTGGCTAACATGTGCTCTCTAGCCGTGGTAAGGTCTCCA

>Hypoxylon_haematostroma

-CATTACAGAGTTAT--CAACTCCCAACCCTTTGTGAACCTTACCGCAGTTGCCTCGGCGCG-
AGCGCGGCTACCCTGCAGCTACCCT------------------------------GTAGCTGCCGCGT-AGCACGC-
ACATGGCCCGCCGGTGGACAGCTAAACTCTTGTATTACACAAGTATGTCTGATTGCTTAAATAAAA
TAAGTCAAAACTTTCAACAACGGATCTCTTGGTTCTGGCATCGATGAAGAACGCAGCGAAATGCGA
TAAGTAATGTGAATTGCAGAATTCAGTGAATCATCGAATCTTTGAACGCACATTGCGCCCATTAGT
ATTCTAGTGGGCATGCCTATTCGAGCGTCATTTCAACCCTTAAGCCTCTGTCGCTTAGCGTTGGGAG
CCTACGTCT-
ACAGCGTATCTCCTCAAAGTCAGTGGCGGTGCTGGAGCACACTCTCAGCGTAGTAGTTCTTCTCGCT
TCTGTAGTGGCCCCTGCAGCCTGCCGTAA--
--------------------C----CTC-----GCGGTCCGAGTTGTAATTTGTAGAGGATGCTTTTGGTGCGGC-

GCCTTCCGAGTTCCCTGGAACGGGACGCCGGAGAGGGTGAGAGCCCCGTACGGTTGGA-
CGCCTAGCCTATATATAGCTCCTTCGACGAGTCGAGTAGTTTGGGAATGCTGCTCTAAATGGGAGG
TAAATTTCTTCTAAAGCTAAATACCGGCCAGAGACC-
GATAGCGCACAAGTAGAGTGATCGAAAGATGAAAAGCACTTTGAAAAGAGGGTTAAATAGCACGT
GAAATTGTTGAAAGGGAAGCG-TTTGCGACCAGACTTTTTCCAGGGGAATCATCCGGTGTTC--
TCACCGGTGCACTTCCCCTGG--TTTAGGCCAGCGTCGGTTCT---
CTTAGGGGGATAAAGGCTTGGGGAACGTAGCTC----TTTCGGGAGTGTT-
ATAGCCCCTCGCGTAATACCCTTCG-GGGGACCGAGGATCGCGCT-CT----
GCAAGGACGCTGGCGTAATGGTCGTCAACGACCCGTCTTGAAACACGGACCAAGGAGTCGAACAT
TTGTGCGAGTGTTTGGGTG--TTAAACCCTCACGCGTAATGAAAGTGAACGGAGGTGAGAGCCCTC--
--AC----GGGTGCATCATCGACCGATCCTGATG-
TCTTCGGATGGATTTGAGTAAGAGCATAACTGTTCGGACCCGAAAGATGGTGAACTATGCGTGGAT
AGGGTGAAGCCAGAGGAAACTCTGGTGGAGGCTCGCAGCGGTTCTGACGTGCAAATCGATCGTCA
AATCTGCGCATGGGGGCGAAAGACTTATCGA-
ACCATCTAGTAGCTGGTTACCGCCGAAGTTTCCCTCAGGATAGCAGTGT--TG-
TCTTCAGTTTTATGAGGTAAAGCGAATGATTAGGGACTCGGGGGCGCTATTTTGCCTTCATCCATTC
TCAAACTTTAAATATGTAAGAAGCCCTTGTTACTTAGTTGAACGTGGGCATTCGAATGTACCAACA
CTAGTGGGCCATTTTTGGTAAGCAGAACTGGCGATGCGGGATGAACCGAACGCGGGGTTAAGGTG
CCAGAGTGGACGCTCATCAGACACCACAAAAGGTGTTAGTACATCTTGACAGCAGGACGGTGGCC
ATGGAAGTCGGAATCCGCTAAGGACTGTGTAACAACTCACCTGCCGAATGTACTAGCCCTGAAAAT
GGATGGCGCTCAAGCGTCT-CACCCATACCTCGCCCTTAGGGTAGAAACGAAGCCCTAAGG-----------
-----
AACAGGCGCTTCCAGATCGAGCTCGCTGCAAAACCCGCCATTATTACCAACGGGNTGAAGTATTCC
CTTGCCACTGGTAACTGGGGTGACCAGAANAAGGNNATGAGCTCGACCGCTGGTGTATCGCAGGT
CCTGAATAGATACACAT------TCGCGTCGA---
CTCTTTCTCACTTGAGAAGAACCAACACCCCCATTGGGAGAGACGGGAAATTGGCGAAACCCCGTC
AGCTTCATAACACTCATTGGGGCTTGGTCTGCCCGGCCGAAACGCCCGAAGGCCAGGCCTGTGGAC
TGGTGAAGAACTTGTCATTGATGTGCTCCATCAGCGTTGGCACGTCAACGGACCCTATCGTGGACT
ACATGATTACCAGGAATATGGAGGTCCTAGAGGAATACGAGCCGATGCGATACCCCAACGCCACT
AAGATCTTCCTCAATGGCTCTTGGATCGGTGTTCACCAAGATCCCAAGTCTCTCGTCAGGGATGTCC
AGCAGCTACGTCGGGCCAACCAGATCCCATCAGAGGTGTCATTGGTTCGCGATATTCGAGATCGAG
AGTTCAAGATCTTCTCGGACGCTGGGCGTGTCATGCGCCCCTTGTTTGTCGTGCAGCAAGAGGAT---
----
ATGCCCGAAAAGAATGTTTCCAAGGGGACATTAGCTCTCACCAAGGAGATGATCCAGAGACTAGA
AGCGGATGTCGACATAGACCCGGATAGCGACGAGTATTTCGGTTGGCAGGGTCTGGTTAACTCGGG
TGTTATCGAGTATCTGGACGCCGAGGAAGAGGAGACGGCCATGATATGCATGACACCTGAGGACC
TGGAGACGTATCGCATGGCCAAGGCCGGATATGATGTGACGCAGGATAATGGA------
GACGAGATTAACAGACGACTAAAAACCAAGATAAATCCTACCACGCACATGTATACCCATTGCGA
GATTCACCCCAGCATGCTTCTGGGCATCTGCGCCAGCATTATTCCCTTCCCCGACCACAACCAGGTA
CCCTGCAACGCGTCTCTC---CCCTTGGATTTGCACCCCTCACCTGCACAAACCATCAACGCA-----------
--TTCAACG---------
GTTCCAACCTTATACCGAACATCACGCGAATCGAAGCTAATCATGTCTCTTCCTATCTATAGGTTCA
CCTTCAAACCGGCCAGTGCGTAAGTGCGAGGCC----
GACGACACCCGATGATTTGTAGCAGGAAGGTTCCACAGAACTCATACAAGGATTATAGGGTAACC
AAATCGGTGCTGCTTTCTGGCAAACCATCTCTGGCGAGCATGGTCTCGACAGCAATGGCGTGTACG
TATTTTATTCGACAATTCAATCTTGAGAATCAGTAACTAACAACCAATAAACAGCTACAACGGAAC
CTCTGAGCTCCAGCTCGAGCGCATGAGCGTCTACTTCAACGAGGTACATACCTATCAAGATCCGTA
ACGGATACGCGTGAATAGTCACTGACTGTACTGACACGCACAGGCTTCCGGTAACAAGTATGTTCC
CCGTGCCGTTCTCGTCGATCTCGAGCCCGGTACCATGGACGCCGTCCGTGCTGGTCCCTTCGGTCAG
CTCTTCCGTCCCGACAACTTCGTCTTCGGCCAATCTGGTGCCGGCAACAACTGGGCCAAGGGTCAC
TACACTGAGGGTGCTGAGCTTGTTGACAACGTCCTCGACGTTGTCCGTCGTGAGGCTGAGGGCTGT
GATTGCCTCCAGGGTTTCCAGATCACCCACTCACTCGGTGGTGGTACCGGTGCCGGTATGGGTACC
TTGTTGATCTCCAAGATTCGCGAGGAGTTCCCCGACCGCATGATGGCAACCTTCTCCGTCGTTCCCT
CCCCCAAAGTTTCCGACACCGTCGTCGAGCCTTACAATGCCACTCTCTCCATCCACCAGCTGGTTGA
GAACTCGGACGAGACCTTCTGCATTGATAACGAGGCCCTCTACGACATCTGCATGCGCACGCTTAA
GTTGTCCAACCCGTCTTACGGCGACCTGAACCACCTGGTCTCCGCCGTCATGTCTGGCGTTACCACT
NGCTTGCGATTCCCCGGCCAGTTGAACTCCGATCTGCGCAAGCTTGCCGTCAACATGGTTCCCTTCC

CTCGTCTCCACTTCTTCATGGTCGGCTTTGCTCCCCTGACCAGCCGCGGCGCCTACTCCTTCCGCGC
CGTTACCGTCCCCGAGTTGACCCAGCAGATGTTCGACCCCAAGAACATGATGGCTGCTTCCGACTT
CCGCAGCGGTCGATACCTGACATGCT-TGCCATCTTGTAAGATATTCACCCCATT--------------------------
----

>Hypoxylon_trugodes

-CATTACTGAGTTATCAAAACTCCCAACCCTTTGTGAACCTTACCACTGTTGCCTCGGCGTC-
AGCGCGGCTACCCTGGGGCTACCCTGGAGTGCACTACCTGGTAGGT-
GCTACCTGGTAGTTACCCTGT-AGCCCGC-
GTATAGCGCGCCGGTGGACCAATAAACTCTGTTTTTACCTGAGAATCTCTGAATGCTTCAACTAAAT
TAGTTAAAACTTTCAACAACGGATCTCTTGGTTCTGGCATCGATGAAGAACGCAGCGAAATGCGAT
AAGTAATGTGAATTGCAGAATTCAGTGAATCATCGAATCTTTGAACGCACATTGCGCCCATTAGTA
TTCTAGTGGGCATGCCTATTCGAGCGTCATTTCAACCCTTAAGCCTTAGTTGCTTAGCGTTGGGACT
CTGAGCCTTACGGCCTAGTTCCTTAAAGTTAGTGGCGGAGTTATAGCACACTCTCAGCGTAGTAAT
CTATCTCGCTTTTGTGGTGGCTGTGGCGACTTGCCGTAAAACCCCTAAT---------------------------------------
------------------TGAAGCGGCAACAGCTCAAA-TTTGAAATCTGGCC----CTT-----
GCGGTCCGAATTGTAATTTGTAGAGGATGCTTTTGGTGCGGT-
GCCTTCTGAGTTCCCTGGAACGGGACGCCAGAGAGGGTGAGAGCCCCGTACGGTTGGA-
CACCTAGCCTATATATAGCTCCTTCGACGAGTCGAGTAGTTTGGGAATGCTGCTCTAAATGGGAGG
TAAATTTCTTCTAAAGCTAAATACCGGCCAGAGACC-
GATAGCGCACAAGTAGAGTGATCGAAAGATGAAAAGCACTTTGAAAAGAGGGTTAAATAGCACGT
GAAATTGTTGAAAGGGAAGCG-TTTGCGACCAGACTTTTTCCAGGCGGATCATCCGGTGTTC--
TCACCGGTGCACTTCGTCTGG--TTTAGGCCAGCATCGGTTTT---
CTTAGGGGGATAAAGGCTTGGGGAACGTAGCTC----TTTCGGGAGTGTT-
ATAGCCCCTTGCGTAATACCCCTCG-GGGGACCGAGGAACGCGCT-CT----
GCAAGGATGCTGGCGTAATGGTCGTCAACGACCCGTCTTGAAACACGGACCAAGGAGTCGAACAT
TTGTGCGAGTGTTTGGGTG--TTAAACCCTCACGCGTAATGAAAGTGAACGGAGGTGAGAGCC-TT---
-A-----GGGTGCATCATCGACCGATCCTGATG-
TCTTCGGATGGATTTGAGTAAGAGCATAACTGTTCGGACCCGAAAGATGGTGAACTATGCGTGGAT
AGGGTGAAGCCAGAGGAAACTCTGGTGGAGGCTCGCAGCGGTTCTGACGTGCAAATCGATCGTCA
AATCTGCGCATGGGGGCGAAAGACTTATCGA-ACCAT---

ACGCTGTATTAGCGACAACAAACGTTTCCAGGTCGAATTGGCAGCCAAGCCTGCTATCGTCACAAA
TGGTCTCAAGTATTCCCTCGCGACGGGTAACTGGGGTGACCAGAAGAAGGCCATGAGCTCAACGG
CTGGTGTGTCGCAGGTCTTGAACAGATATACAT------TCGCATCAA---
CTCTTTCCCACTTGAGGCGAACGAACACGCCTATCGGACGAGACGGAAAATTAGCTAAGCCCCGAC
AACTTCACAACACTCATTGGGGTCTTGTCTGCCCGGCCGAAACGCCCGAAGGCCAAGCTTGTGGGT
TGGTCAAGAATTTATCGTTGATGTGTTCTATCAGTGTGGGTACCTCCACAGAACCCATCATTGACTA
TATGATCACGAGAAGTATGGAAGTGCTCGAGGAATATGAGCCCATGCGCTACCCCAATGCCACGA
AGATCTTCCTCAATGGTTCTTGGATTGGTGTACACCAAGATCCTAAGTCGCTTGTCAGAGATGTCCA
ACAGCTTCGCCGAGCCAACCAAATTCCCTCGGAAGTTTCATTGGTTCGTGACATTCGTGATCGTGA
GTTCAAGATATTCTCAGATGCAGGCCGTGTCATGCGTCCCTTGTTCGTTGTTCAACAAGAAGAC------
----

GCCCCTGAAGCTGGCATCGAGAAAGGTACACTGGCTTTGAACAAGGAAATGATCCAACGACTAGA
GGCGGACGTCGATCTCGATCCGGAAAGCGAAGAGTATTTTGGTTGGCAAGGTCTAGTCAATGAGG
GTGTTATTGAGTATCTTGATGCCGAGGAAGAAGAGACGGCCATGATTTGCATGACTCCCGAAGACC
TAGAAAACTACCGAATGACGAAGCTCGGCCACGAAGCACCACAGGACAACGGC------
GATGAAGTTAACAAACGACTCAAGACCAAGGTGAATCCTTCGACACACATGTATACGCACTGTGA
GATCCATCCTAGCATGCTTCTAGGTATCTGCGCCAGCATCATTCCTTTCCCGGATCACAATCAGGTA
CCCTTGAACGCGTCCCTCAAACCCCTTGATTTCTGCCCCTCACGCACACGAAACACGATT---GTAC-
CCCATATTATTTACACCATGATACCCTCAAATCGTGTGATA---
CGATCAAGGATAATAGCTAACCATATTTTTTTATCTAAATAGGTTCACCTCCAGACCGGCCAATGC
GTGAGTACA----------
ACCATAACCGATAAAATATCATCTCTGGGTATAGCGGGGAACTCATAAGTATTATAGGGTAACCAA
ATTGGTGCCGCCTTCTGGCAAACCATCTCTGGCGAGCACGGTCTCGACAGCAATGGCGTGTACGTA

TTCGACTCGCCAATTCATATGCCAAGGATGCGAACTAATCAGCAATAAACAGCTACAACGGAACTT
CCGAGCTCCAGCTCGAGCGCATGAGCGTCTACTTTAACGAGGTACGGGGCTCAAT-----
GCCTATACACGGAAGGGATCAGTTACTAATCACCCCAACATGCACAGGCATCTGGTAACAAATACG
TTCCTCGCGCCGTCCTCGTCGATCTCGAACCCGGTACCATGGACGCCGTTCGCGCCGGTCCTTTCGG
ACAACTTTTCCGCCCCGACAACTTCGTCTTCGGTCAGTCCGGTGCTGGAAACAACTGGGCCAAGGG
TCACTACACGGAAGGTGCTGAGCTAGTCGACAACGTTCTTGACGTCGTTCGTCGTGAGGCCGAGGC
TTGCGACTGCCTCCAGGGTTTCCAGATCACCCACTCTCTCGGTGGTGGTACTGGTGCTGGTATGGGT
ACTCTATTGATCTCCAAGATCCGCGAAGAGTTCCCCGACCGCATGATGGCTACTTTCTCGGTCGTTC
CCTCTCCTAAGGTTTCCGACACCGTCGTTGAGCCTTACAATGCTACTCTCTCCGTCCACCAGCTGGT
CGAGAACTCCGACGAGACCTTCTGCATTGACAACGAGGCTCTCTACGACATCTGCATGCGTACTCT
TAAGCTATCCAACCCCTCGTACGGTGACCTGAACCACCTGGTTTCCGCTGTCATGTCCGGTGTTACT
ACTTGCTTGCGATTCCCTGGTCAGCTAAACTCAGATCTACGCAAGCTCGCTGTAAACATGGTTCCGT
TCCCTCGTCTCCACTTCTTCATGGTTGGATTCGCTCCTCTAACCAGCCGTGGTGCTTACACTTTCCGT
GCTGTCACCGTTCCTGAGTTGACTCAGCAAATGTTCGACCCCAAGAACATGATGGCTGCTTCTGACT
TCCGTAACGGTCGCTACCTAACGTGCTCTGCCATCTTGTAAGATACTCTCTTCCCGAAATTTGTTAC
TACC--ATTACTAACATA--TTTTCTAGCCGTGGCAAGGTCACTG

>Hypoxylon_crocopeplum

------CAGAGTTATCTAAACTCCAAACCCTTTGTGAACCTTACCACTGTTGCCTCGGCGTA-
AGCGCAGTTACCCTATAGCTACCCTGTAACTATCTACCCTGTA----
GCTACCCTGGAGTTACCCTGTAGCTATGCAAAATAGCCCGCCGAAGGACCACTAAACTCTTGTTTC
TACACTGTATCTCTGAATGCTTCAACT-
AAATAATTAAAACTTTCAACAACGGATCTCTTGGTTCTGGCATCGATGAAGAACGCAGCGAAATGC
GATAAGTAATGTGAATTGCAGAATTCAGTGAATCATCGAATCTTTGAACGCACATTGCGCCCATTA
GTATTCTAGTGGGCATGCCTATTCGAGCGTCATTTCAACCCTTAAGCCCTTGTTGCTTAGCGTTGGG
AATCTACGATTTACAGCGTAGTTCCTTAAAGTTAGTGGCGGAGTTAGGGCATACTCTAAGCGTAGT
AATCTATCTCGCTTTTGTAGTAGCCTTGGCTTCTTGCCGTAAAGCCCCCTA---------------------------------
--------------------TGAAGCGGCAACAGCTCAAA-TTTGAAATCTGGCC----CTC-----
GTGGTCCGAGTTGTAATTTGCAGAGGATGCTTTTGGTGAGGT-
GCCTTCCGAGTTCCCTGGAACGGGACGCCAGAGAGGGTGAGAGCCCCGTACGGTTGGA-
CGCCTAGCCTCTTTATAGCTCCTTCGACGAGTCGAGTAGTTTGGGAATGCTGCTCTAAATGGGAGGT
AAATTTCTTCTAAAGCTAAATACCGGCCAGAGACC-
GATAGCGCACAAGTAGAGTGATCGAAAGATGAAAAGCACTTTGAAAAGAGGGTTAAATAGCACGT
GAAATTGTTGAAAGGGAAGCG-TTTGCGACCAGACTTTTTCCAGGCGGATCATCCGGTGTTC--
TCACCGGTGCACTTCGTCTGG--TTTAGGCCAGCATCGGTTTT---
CTTAGGGGGATAAAGGCTTAGGGAACGTAGCTC----TTTAGGGAGTGTT-
ATAGCCCTTTGCGTAATACCTTTCA-GGGGACCGAGGATCGCGCT-CT----
GCAAGGATGCTGGCGTAATGGTCGTCAACGACCCGTCTTGAAACACGGACCAAGGAGTCGAACAT
TTGTGCGAGTGTTTGGGTG--TTAAACCCTCACGCGTAATGAAAGTGAACGGAGGTGAGAGCCCTT--
--AC----GGGTGCATCATCGACCGATCCTGATG-
TCTTCGGATGGATTTGAGTAAGAGCATAACTGTTCGGACCCGAAAGATAGTGAACTATGCGTGGAT
AGGGTGAAGCCAGAGGAAACTCTGGTGGAGGCTCGCAGCGGTTCTGACGTGCAAATCGATCGTCA
AATCTGCGCATGGGGGCGAAAGACTTATCGA-
ACCATCTAGTAGCTGGTTACCGCCGAAGTTTCCCTCAGGATAGCAGTGT--TG-
TCTTCAGTTTTATGAGGTAAAGCGAATGATTAGGGACTCGGGGGCGCTATTTTGCCTTCATCCATTC
TCAAACTTTAAATATGTAAGAAGCCCTTGTTACTTAATTGAACGTGGGCATTCGAATGTACCAACA
CTAGTGGGCCATTTTTGGTAAGCAGAACTGGCGATGCGGGATGAACCGAACGCGGGGTTAAGGTG
CCAGAGTGGACGCTCATCAGACACCACAAAAGGTGTTAGTACATCTTGACAGCAGGACGGTGGCC
ATGGAAGTCGGAATCCGCTAAGGACTGTGTAACAACTCACCTGCCGAATGTACTAGCCCTGAAAAT
GGATGGCGCTCAAGCGTCT-CACCCATACCTCGCCCTTAGGGTAGAAACGATGCCCTAAGG----------
----
TCTCAACCGTTACACGT------TCGCCTCGA---
CCCTCTCGCATTTGAGGAGAACCAATACGCCTATTGGAAGAGACGGAAAGCTGGCCAAGCCTCGA
CAGCTGCACAACACTCATTGGGGCCTGGTCTGCCCGGCTGAAACCCCTGAAGGCCAAGCTTGTGGC
CTTGTGAAGAATCTGTCGCTGATGTGCTCTATCAGCGTGGGTACTTCGACGGATCCTATTGTCGACT
ATATGATTACTAGGAATATGGAAGTCCTAGAGGAGTACGAACCCATGAGATATCCTAACGCCACCA
AGATCTTCCTGAACGGCTCGTGGATCGGTGTACATCAAGACCCCAAATCTCTCGTCAGAGATGTTC

AACAGCTGCGTCGGGCCAACCAGATCCCATCCGAGGTCTCACTAGTCCGCGATATCCGTGATCGCG
AGTTCAAGATCTTTTCGGATGCGGGCCGTGTCATGCGCCCCTTGTTTGTCGTACAGCAAGAGGAC---
----

GCTGGCGAGACCAAGGGCTGTTTGGCTCTCAACAAAGACATGATACAGAAACTCGAGGCAGACGT
CGACATAGATCCCGAGAACGAAGAGTACTATGGCTGGCAGGGCCTAGTTAACGACGGCGTCATCG
AGTACCTCGACGCAGAAGAGGAAGAGACAGCAATGATATGCATGACGCCTGAAGACCTGGAGAAT
TACCGGTTGACCAAGGCCGGGTTTGATGTTGTTCAAGATAACGGG------
GATGAGATTAACAAACGCCTCAAAACCAAGGTCAACCCCACGACACACATGTATACTCACTGCGA
GATTCATCCTAGCATGCTCCTAGGCATCTGCGCCAGCATTATCCCCTTCCCGGATCACAACCAGGTA
CCCCTGAACGCGTCCCTGGACCCCCTGATTTTGTGCCCCTCACGCACAAACCAC-
ATACATAAACATCATACAGCTATCTATATCAAGCTGTTTTTAATTTACACAACGACAGAT-
GGAGAATTGAAGCTAACTATGTCTCTTTATCATTATAGGTTCACCTTCAGACCGGCCAGTGCGTAA
GTATAACGAC----AAC--------
ACAAAAGATCGCGATGGAACATAGCAGGGCTCACACGATACTAATAGGGTAACCAAATTGGTGCT
GCTTTCTGGCAAACCATCTCTGGCGAGCACGGCCTCGACAGCAATGGCGTGTACGTATCTAATTCG
GCAATTCTAAGAC-
AAGAGTGGAAACTGACCGCCAATCAACAGCTACAACGGAACTTCCGAGCTCCAGCTTGAGCGCAT
GAGCGTCTACTTCAACGAGGTACGCCCTCATCGAAACTGATTAA----
ACGCGAAAGACTTGGCTAATCACTAC----
TATATAGGCTTCTGGCAACAAGTATGTTCCTCGCGCTGTCCTCGTCGATCTCGAGCCTGGTACCATG
GATGCCGTCCGTGCTGGTCCCTTCGGTCAGCTCTTCCGACCTGACAACTTCGTCTTCGGCCAGTCTG
GTGCCGGCAACAACTGGGCCAAGGGTCACTACACCGAGGGTGCCGAGCTCGTTGACCAGGTTCTCG
ATGTCGTCCGTCGTGAAGCTGAAGGCTGCGACTGCCTTCAGGGCTTCCAGATCACCCACTCCCTCG
GTGGTGGTACTGGTGCTGGTATGGGTACCCTGTTGATCTCCAAGATCCGCGAGGAGTTCCCTGACC
GCATGATGGCCACCTTCTCTGTCGTTCCCTCGCCCAAAGTTTCCGACACTGTCGTCGAGCCTTACAA
CGCCACCCTCTCCGTCCACCAGCTGGTCGAGAACTCGGACGAGACCTTCTGCATTGACAACGAGGC
TCTGTACGACATCTGCATGCGTACCCTTAAGCTATCCAACCCCTCGTACGGCGACCTGAACTACCTG
GTATCCGCTGTCATGTCTGGTGTCACCACTTGCCTGCGTTTCCCCGGTCAGCTGAACTCGGACCTGC
GCAAGCTTGCCGTCAACATGGTTCCCTTCCCTCGTCTGCACTTCTTCATGGTCGGCTTCGCTCCCTTG
ACCAGCCGTGGTGCTCACTCGTTCCGTGCTGTCACCGTCCCCGAGTTGACTCAGCAGATGTTCGACC
CCAAGAACATGATGGCTGCCTCTGATTTCCGCAACGGCCGCTACCTGACTTGCTCTGCCATCTTGTA
AGATACATTTCCCTACTGGTTCTGTCACGTTTATTTGCTAACCTGTCTCTTCCAGCCGCGGCAAGGT
CTCGA

>Hypoxylon_papillatum

-CATTAGCGAGTTTACAAAACTCCCAACCCTTTGTGAACCTTACCATTGTTGCCTCGGCGTG-
AGCGCGGCTACCTGGTAGCTACCCTGTAATTG-TTACCCTGTA-
ATGATTACCCGGGAGCTACCCTGT-
AGTACGCGTGAAGGCCCGCCGGTGGACCACTAAACTCTGTTTATCATTGTGGAATTCTGAATACTT
AA-
CTAAATACGTTAAAACTTTCAACAACGGATCTCTTGGTTCTGGCATCGATGAAGAACGCAGCGAAA
TGCGATAAGTAATGTGAATTGCAGAATTCAGTGAATCATCGAATCTTTGAACGCACATTGCGCCCA
TTAGTATTCTAGTGGGCATGCCTATTCGAGCGTCATTACAACCCTTAAGCCCTTGTTGCTTAGTGTT
GGGACTCTACGGCCTAGGGCGTAGTTCCTTAAAGTTAGTGGCGGAGTTAGGGTACACTCTCAGCGT
AGTAGTTTTTCTCGCTTTTGTAGTGGTCCTAACGGCTAGCCGTAAAACCCTT----
ATTCTAGTGGTTGACCTCGGATTAGGTAGGAATACCCGCTGAACTTAAGCATA-
GAAGCGGCAACAGCTCAAA-TTTGAAATCTGGCC----CTC-----
GTGGTCCGAGTTGTAATTTGTAGAGGATGCTTTTGGCGCGGT-
GCCTTCTGAGTTCCCTGGAACGGGACGCCAGAGAGGGTGAGAGCCCCGTACGGTTGGA-
CACCTAGCCTCTGTATAGCTCCTTCGACGAGTCGAGTAGTTTGGGAATGCTGCTCTAAATGGGAGG
TAAATTTCTTCTAAAGCTAAATACCGGCCAGAGACC-
GATAGCGCACAAGTAGAGTGATCGAAAGATGAAAAGCACTTTGAAAAGAGGGTTAAATAGCACGT
GAAATTGTTGAAAGGGAAGCG-TTTGCGACCAGACCTTTTCCTGGCGGATCATCCGGTGTTC--
TCACCGGTGCACTTCGCTTGG--TTTAGGCCAGCATCGGTTTT---
CTTAGGGGGATAAAGGCTTAGGGAACGTAGCTC----CTCCGGGAGTGTT-
ATAGCCCTCTGCGTAATACCCTTCA-GGGGACCGAGGACCGCGCT-CT----
GCAAGGATGCTGGCGTAATGGTCGTCAACGACCCGTCTTGAAACACGGACCAAGGAGTCGAACAT

TTGTGCGAGTGTTTGGGTG--TTAAACCCTCACGCGTAATGAAAGTGAACGGAGGTGAGAGCCCTT--
--AC----GGGTGCATCATCGACCGATCCTGATG-
TCTTCGGATGGATTTGAGTAAGAGCATAACTGTTCGGACCCGAAAGATGGTGAACTATGCGTGGAT
AGGGTGAAGCCAGAGGAAACTCTGGTGGAGGCTCGCAGCGGTTCTGACGTGCAAATCGATCGTCA
AATCTGCGCATGGGGGCGAAAGACTTATCGA-
ACCATCTAGTAGCTGGTTACCGCCGAAGTTTCCCTCAGGATAGCAGTGT--TG-
TCTTCAGTTTTATGAGGTAAAGCGAATGATTAGGGACTCGGGGGCGCTATTTAGCCTTCATCCATTC
TCAAACTTTAAATATGTAAGAAGCCCTTGTTACTTAATTGAACGTGGCATTCGAATGTACCAACA
CTAGTGGGCCATTTTTGGTAAGCAGAACTGGCGATGCGGGATGAACCGAACGCGGGGTTAAGGTG
CCAGAGTGGACGCTCATCAGACACCACAAAAGGTGTTAGTACATCTTGACAGCAGGACGGTGGCC
ATGGAAGTCGGAATCCGCTAAGGACTGTGTAACAACTCACCTGCCGAATGTACTAGCCCTGAAAAT
GGATGGCGCTCAAGCGTCT-CACCCATACCTCGCCCTCAGGGTAGAAACGATGCC-----------------------
----
ATCGATATACTT------TCGCATCGA---
CTCTCTCCCATCTAAGGAGAACGAACACCCCCATCGGACGTGATGGGAAGCTTGCGAAACCCCGGC
AGCTACACAATACCCATTGGGGTCTAGTCTGTCCGGCGGAAACGCCTGAAGGCCAGGCCTGCGGGC
TTGTAAAGAACTTATCGCTAATGTGCTCGATCAGTGTGGGTACGTCGACAGATCCCATCGTCGATT
ATATGATTACCCGAAACATGGAGGTTCTTGAAGAATACGAGCCTATGCGATATCCCAACGCTACCA
AGATCTTCCTCAACGGTTCATGGATCGGTGTGCACCAGGACCCCAAGGCTCTGGTCAGAGATGTCC
AGAACCTTCGCCGGACCAATCAGATCCCCGCTGAGGTGTCCTTAGTTCGGGACATCCGCGATCGTG
AATTCAAGATCTTCTCGGATGCTGGTCGGGTTATGCGTCCCTTATTCGTCGTCGAACAGGAGGGTG
AG---
AGGAAAGGGTCGTTAACCCTCACCAAGGAGATGATTCACAGGCTGGAGGCAGATGTAGACCTGCC
GCAGGATAGTGAGGAGTACTTTGGCTGGCAAGGTCTAGTGAACGAGGGCGTCATCGAATTCCTAG
ATGCCGAAGAAGAGGAGACAGCCATGATTTGTATGACGCCCGAAGACCTGGAAGCTTACCGTCAG
GCCAAAGCCGGTTATGAGCCGGAGGAGAAGGAAGCC---
CCTCAGGAGATTAACAGGCGACTGAAGACCAAGATGAATCCGACGACGCACATGTACACACACTG
CGAAATCCATCCCAGTATGCTCTTGGGTATCTGCGCCAGCATTATCCCGTTCCCCGACCACAATCAG
GTACCCCTGAACGCGTCC----------T----
TGATGCCCCTCACACGCATACACACCAAACATGGCATCCTGGAGTTCTCTACATCCATCCACCGTC
AACCTCTACCACAACACGATGGAAAACTGAAGCTAACCGTGTCTCTTCATCTCTACAGGTCCATCT
CCAGACCGGCCAATGCGTAAGTACC----------
ACAACCACCGAACAGCTATCGCGCTGGGACATTGCGGGGCTCACATGAA-
ATCGCAGGGTAACCAAATTGGTGCTGCCTTCTGGCAAACCATCTCCGGTGAGCATGGTCTCGACAG
CAATGGCGTGTAAGTATCTTTCGACCCCGATTAGCCCATGGGAATGATAATTGATCATCTGTAAAT
AGCTACAACGGAACCTCCGAGCTCCAGCTCGAGCGCATGAGCGTCTACTTCAACGAGGTATGCGGG
CATGGTAAT-GGGAATAGATGCAAGGGATGAGGTACTAAT----
CTATTAATAATAGGCTTCCGGCAACAAGTATGTTCCTCGCGCTGTCCTCGTCGATCTTGAGCCCGGT
ACCATGGATGCCGTCCGTGCTGGTCCCTTCGGCCAGCTCTTCCGACCTGACAACTTCGTCTTCGGCC
AGTCCGGTGCTGGAAACAACTGGGCCAAGGGTCACTACACCGAGGGCGCCGAGCTTGTCGACAAC
GTTCTCGATGTCGTCCGTCGCGAGGCTGAGGGTTGCGATTGCCTCCAGGGTTTCCAGATCACCCACT
CTCTCGGTGGTGGTACCGGTGCCGGTATGGGTACCCTGCTGATCTCTAAGATCCGTGAGGAGTTCC
CCGACCGCATGATGGCTACTTTCTCCGTCGTGCCCTCCCCCAAGGTTTCCGACACCGTCGTCGAGCC
TTACAATGCCACCCTCTCCGTCCACCAGCTGGTCGAGAACTCTGACGAGACTTTCTGCATTGACAAC
GAGGCCCTTTACGACATCTGCATGCGCACTCTGAAGTTGTCCAACCCCTCGTACGGCGATCTCAAC
CACCTGGTCTCCGCTGTCATGTCCGGCGTCACTACTTGCCTACGTTTCCCGGGCCAGCTGAACTCTG
ACCTGCGCAAGCTCGCCGTGAACATGGTGCCTTTCCCCCGTCTACACTTCTTCATGGTCGGATTCGC
TCCTCTGACCAGCCGTGGTGCTCACTCCTTCCGCGCTGTCACTGTTCCCGAGTTGACTCAGCAGATG
TTCGACCCCAAGAACATGATGGCTGCCTCTGATTTCCGCAATGGCCGCTACCTCACATGCTCTGCCA
TCTTGTAAGCTTTCCCCCCCTTTCTTCTATCCTTGACGATCTTGCTGACTTAATTAATCTAGCCGTGG
CAAGGTTTCCA

>Parahypoxylon_ruwenzoriense_MUCL51392

-CATTAGCGAGTTTACAAAACTCCCAACCCTTTGTGAACCTTACCACTGTTGCCTCGGCGTG-
AGCGCGTCTACCTGGTAGTTACCCTGTATTTATTTACCCTGTA-
ATGATTACCCGGGAGCTACCCTGT-
AATTCGCGTGAAGGCCCGCCGGTGGACCACTAAACTCTGTTTATCACTGTGGAATTCTGAATACCT

AAACTAAATACGTTAAAACTTTCAACAACGGATCTCTTGGTTCTGGCATCGATGAAGAACGCAGCG
AAATGCGATAAGTAATGTGAATTGCAGAATTCAGTGAATCATCGAATCTTTGAACGCACATTGCGC
CCATTAGTATTCTAGTGGGCATGCCTATTCGAGCGTCATTACAACCCTTAAGCCCTTGTTGCTTAGC
GTTGGGACTCTACGGCCTAGGGCGTAGTTCCTTAAAGTTAGTGGCGGAGTTAGGGTACACTCTAAG
CGTAGTAGTTCTTCTCGCTTTTGCAGTGGCCTTAGCTGCTAGCCGTAAAACCCCT----
ATTCTAGTGGTTGACCTCGGATTAGGTAGGAATACCCGCTGAACTTAAGCATATGAAGCGGCAACA
GCTCAAA-TTTGAAATCTGGCC----CTC-----
GTGGTCCGAGTTGTAATTTGTAGAGGATGCTTTTGGCGCGGT-
GCCTTCTGAGTTCCCTGGAACGGGACGCCAGAGAGGGTGAGAGCCCCGTACGGTTGGA-
CACCTAGCCTTTGTATAGCTCCTTCGACGAGTCGAGTAGTTTGGGAATGCTGCTCTAAATGGGAGG
TAAATTTCTTCTAAAGCTAAATACCGGCCAGAGACC-
GATAGCGCACAAGTAGAGTGATCGAAAGATGAAAAGCACTTTGAAAAGAGGGTTAAATAGCACGT
GAAATTGTTGAAAGGGAAGCG-TTTGCGACCAGACCTTTTCCAGGCGGATCATCCGGTGTTC--
TCACCGGTGCACTTCGCCTGG--TTTAGGCCAGCATCGGTTTT---
CTTAGGGGGATAAAGGCTTAGGGAACGTAGCTC----CTCAGGGAGTGTT-
ATAGCCCTTTGCGTAATACCCCTCG-GGGGACCGAGGACCGCGCT-CT----
GCAAGGATGCTGGCGTAATGGTCGTCAACGACCCGTCTTGAAACACGGACCAAGGAGTCGAACAT
TTGTGCGAGTGTTTGGGTG--TTAAACCCTCACGCGTAATGAAAGTGAACGGAGGTGAGAGCCCTT--
--AC----GGGTGCATCATCGACCGATCCTGATG-
TCTTCGGATGGATTTGAGTAAGAGCATAACTGTTCGGACCCGAAAGATGGTGAACTATGCGTGGAT
AGGGTGAAGCCAGAGGAAACTCTGGTGGAGGCTCGCAGCGGTTCTGACGTGCAAATCGATCGTCA
AATCTGCGCATGGGGGCGAAAGACTTATCGA-
ACCATCTAGTAGCTGGTTACCGCCGAAGTTTCCCTCAGGATAGCAGTGT--TG-
TCTTCAGTTTTATGAGGTAAAGCGAATGATTAGGGACTCGGGGGCGCTATTTAGCCTTCATCCATTC
TCAAACTTTAAATATGTAAGAAGCCCTTGTTACTTAATTGAACGTGGGCATTCGAATGTACCAACA
CTAGTGGGCCATTTTTGGTAAGCAGAACTGGCGATGCGGGATGAACCGAACGCGGGGTTAAGGTG
CCAGAGTGGACGCTCATCAGACACCACAAAAGGTGTTAGTACATCTTGACAGCAGGACGGTGGCC
ATGGAAGTCGGAATCCGCTAAGGACTGTGTAACAACTCACCTGCCGAATGTACTAGCCCTGAAAAT
GGATGGCGCTCAAGCGTCT-
CACCCATACCTCGCCCTCAGGGTAGAAACGATGCCCTGAGGGCGCTGTATTGATTCGAACAGACGC
TTCCATATCGAGCTTGCTGCCAAGCCCGGCATCATCAGCAATGGTTTGRAATACTCCCTAGCCACG
GGCAATTGGGGCGACCAGAAGAAGGCGATGAGCTCAACCGCCGGTGTGTCGCAGGTCCTGAATCG
ATATACTT------TCGCATCCA---
CGCTTTCCCATCTAAGAAGAACGAACACGCCCATCGGACGCGATGGGAAGCTTGCGAAACCCCGG
CAACTGCACAACACCCATTGGGGCCTAGTCTGTCCGGCAGAAACGCCTGAAGGCCAGGCCTGCGG
GCTTGTCAAGAACTTATCGCTAATGTGTTCGATCAGTGTGGGTACGTCGACAGATCCCATTGTCGAT
TATATGATTACCCGAAACATGGAGGTTCTTGAAGAATACGAGCCCATGCGGTATCCCAACGCTACC
AAGATCTTCCTCAACGGATCATGGATCGGTGTTCACCAGGACCCGAAGGCTCTGGTCAGAGACGTC
CAGAACCTTCGCCGGACCAATCAGATCCCCGCTGAGGTGTCCTTAGTCCGAGACATCCGTGACCGT
GAATTCAAGATCTTTTCGGATGCCGGTCGTGTTATGCGTCCCTTATTTGTCGTCGAACAGGAGGGTG
AG---
AGGAAAGGGTCGTTAATCCTCACCAAGGAGATGATTCACAGGCTGGAGGCAGATGTGGACCTACC
TCAGGATAGTGAGGACTACTTTGGCTGGCAAGGTCTAGTGAACGAAGGCGTCATCGAATTTTTAGA
TGCTGAGGAAGAAGAGACCGCCATGATTTGTATGACACCCGAAGACTTGGAAGCTTACCGTCAGG
CCAAAGCCGGTTATGAGCCGGAGAAAGAAAATACC------
CAGGAGATTAACAGGCGACTCAAGACCAAGATGAATCCGACGACTCACATGTACACGCACTGCGA
GATCCATCCAAGTATGCTCTTGGGTATCTGCGCTAGCATTATCCCATTCCCCGACCACAACCAGGTA

AATTGAAGCTAACCGTGTCTCTTCATCGCTATAGGTTCACCTCCAGACCGGCCAATGCGTAAGTAC
C----------ATAACCACCAAACAACTATCGCGCTGGGACATCGCGGGGCTCACATAAA-
ATCGCAGGGTAACCAAATTGGTGCCGCCTTCTGGCAAACCATCTCTGGCGAGCACGGTCTCGACAG
CAATGGCGTGTATGTATTTTTCGCCTCCTGTCAGCCCATAAGAGCAGCAACTGATCATCCGTAAATA
GCTACAATGGAACCTCCGAGCTCCAGCTCGAGCGCATGAGCGTCTACTTCAACGAGGTATGCGGAC
ACAGGAAT-
GGGAATAGATGCAGGGGATGAGGTGCTAATTCTCCTATTAATAATAGGCTTCCGGCAACAAGTACG
TTCCTCGCGCCGTTCTCGTCGATCTTGAGCCCGGTACCATGGATGCCGTCCGTGCTGGTCCCTTCGG
CCAGCTCTTCCGACCTGACAACTTCGTCTTCGGCCAGTCCGGTGCTGGCAACAACTGGGCCAAGGG

TCACTACACCGAGGGTGCCGAGCTTGTCGATAACGTTCTCGATGTCGTCCGTCGCGAGGCTGAGGG
CTGCGATTGCCTCCAGGGTTTCCAGATCACCCACTCCCTCGGTGGTGGTACCGGTGCCGGTATGGGT
ACTCTGCTGATCTCCAAGATCCGTGAGGAGTTCCCTGACCGCATGATGGCTACTTTCTCCGTCGTCC
CCTCCCCCAAGGTTTCCGACACCGTTGTCGAGCCTTACAATGCCACTCTCTCTGTCCACCAGCTGGT
CGAGAACTCTGACGAGACTTTCTGCATTGACAACGAGGCCCTGTACGACATCTGCATGCGCACTCT
GAAGCTGTCCAACCCCTCATATGGCGATCTCAACCACCTGGTCTCTGCTGTCATGTCCGGTGTCACT
ACTTGTCTGCGTTTCCCGGGCCAGCTGAACTCCGACCTGCGCAAGCTTGCCGTGAACATGGTGCCCT
TCCCCCGTCTGCACTTCTTCATGGTCGGATTCGCTCCTCTGACCAGCCGTGGCGCCCACTCCTTCCG
CGCCGTTACTGTTCCCGAGTTGACTCAGCAGATGTTCGACCCCAAGAACATGATGGCTGCCTCTGA
TTTCCGTAATGGCCGCTACCTCACATGCTCTGCCATCTTGTAAGTTTTTCTCCATTCTCTCTATCCT
CGACAGTCTTACTGACTTAATTAATCTAG----------------

>Hypoxylon_perforatum

-
CATTACTGAGTTCTAAAAACTCCCAACCCTTTGTGAATTATACCTTAGTTGCCTCGGCGTCGAGCAG
ACCTACCCGGGAGCTACCCTGGAGTCACCTACCCTGTAGACGGCTACCCTGGAGCTACCCTGTAGT
TGCAC-TTTCGCGTCGCCGGTGGACTACCAAACTCTTATATGTATAGTGTATCTCTGAATTCTT-
AACAAAATTAGTTAAAACTTTCAACAACGGATCTCTTGGTTCTGGCATCGATGAAGAACGCAGCGA
AATGCGATAAGTAATGTGAATTGCAGAATTCAGTGAATCATCGAATCTTTGAACGCATATTGCGCC
CAGTAGTATTCTACTGGGCATGCCTATTCGAGCGTCATTTCAACCCTTACG-
CCCTGTAGCGTAGTGTTAGGACTCTACTCTTTAGAGAGCAGTTCCCTAAAACCAGTGGCAGTGTTC-
GGTACACTCATAGCGTAGTAAT--
TTCTCGCTTCTGCAGTGGCCCGTACTACTCGCCGTAAAACCCCTAAT-
TTTCTAATGGTTGACCTCGGATTAGGTAGGAATACCCGCTGAACTTAAGCAT-
TGAAGCGGCAACAGCTCAAA-TTTGAAATCTGGCC----CTC-----
GTGGTCCGAGTTGTAATTTGTAGAGGATGCTTTTGGCGCGGT-
GCCTTCTGAGTTCCCTGGAACGGGACGCCAGAGAGGGTGAGAGCCCCGTACGGTTGGA-
CACCTAGCCTCTGTATAGCTCCTTCGACGAGTCGAGTAGTTTGGGAATGCTGCTCTAAATGGGAGG
TAAATTTCTTCTAAAGCTAAATACCGGCCAGAGACC-
GATAGCGCACAAGTAGAGTGATCGAAAGATGAAAAGCACTTTGAAAAGAGGGTTAAATAGCACGT
GAAATTGTTGAAAGGGAAGCG-TTTGCGACCAGACCTTTTCCTGGCGGATCATCCGGTGTTC--
TCACCGGTGCACTTCGCTTGG--TTTAGGCCAGCATCGGTTTT---
CTTAGGGGGATAAAGGCTTAGGGAACGTAGCTC----CTCCGGGAGTGTT-
ATAGCCCTCTGCGTAATACCCTTCA-GGGGACCGAGGACCGCGCT-CT----
GCAAGGATGCTGGCGTAATGGTCGTCAACGACCCGTCTTGAAACACGGACCAAGGAGTCGAACAT
TTGTGCGAGTGTTTGGGTG--TTAAACCCTCACGCGTAATGAAAGTGAACGGAGGTGAGAGCCCTT--
--AC----GGGTGCATCATCGACCGATCCTGATG-
TCTTCGGATGGATTTGAGTAAGAGCATAACTGTTCGGACCCGAAAGATGGTGAACTATGCGTGGAT
AGGGTGAAGCCAGAGGAAACTCTGGTGGAGGCTCGCAGCGGTTCTGACGTGCAAATCGATCGTCA
AATCTGCGCATGGGGGCGAAAGACTTATCGA-
ACCATCTAGTAGCTGGTTACCGCCGAAGTTTCCCTCAGGATAGCAGTGT--TG-
TCTTCAGTTTTATGAGGTAAAGCGAATGATTAGGGACTCGGGGGCGCTATTTAGCCTTCATCCATTC
TCAAACTTTAAATATGTAAGAAGCCCTTGTTACTTAATTGAACGTGGGCATTCGAATGTACCAACA
CTAGTGGGCCATTTTTGGTAAGCAGAACTGGCGATGCGGGATGAACCGAACGCGGGGTTAAGGTG
CCAGAGTGGACGCTCATCAGACACCACAAAAGGTGTTAGTACATCTTGACAGCAGGACGGTGGCC
ATGGAAGTCGGAATCCGCTAAGGACTGTGTAACAACTCACCTGCCGAATGTACTAGCCCTGAAAAT
GGATGGCGCTCAAGCGTCT-CACCCATACCTCGCCCTCAGGGTAGAAACGATGCCCTGAGG-----------
----
----------TACACTT------TCGCCTCGA---
CGCTTTCTCATTTGAGAAGAACTAACACGCCCATCGGGCGAGATGGAAAGCTGGCAAAGCCTCGAC
AGCTTCACAATACCCATTGGGGTCTGGTCTGCCCCGCCGAAACGCCTGAGGGTCAAGCCTGCGGTC
TGGTGAAGAACTTATCACTGATGTGTTCCATCAGCGTCGGCACATCTACGGATCCCATCGTAGATT
ACATGATTACGAGAAATATGGAGGTACTCGAGGAGTACGAACCCTTACGATACCCCGACGCCACG
AAAATCTTCCTCAACGGCTCTTGGATCGGCGTGCACCAGAACCCTAAAGCTCTGGTGAGAGATGTT
CAGAATCTGCGCCGGACCAATCAGATCCCGGCCGAGGTGTCGTTGGTCCGCGACATACGTGATCGT
GAATTCAAGATCTTTTCAGATGCTGGCCGCGTTATGCGCCCCATGTTCGTTGTTAACCAAGAGGAC--
----

ACGGAGGGTGGCGCTAAGAAGGGAACGCTAGCTCTCACCAAGGAGATGATTCAGAGGCTCGAAGC
AGATGTCGAGATAGATCCTAACAGTCAGGAGTACTTTGGCTGGCAAGGCTTGGTCAACGAAGGTGC
CATCGATTATCTTGATGCCGAGGAGGAAGAAACGGCCATGATCTGCATGACACCCGAAGACCTCG
AGACCTACCGACAGACTAAGGCCGGATACCAAGTGTCGCAAGACAACGGG------
GATGAGGTTAACAAACGTCTGAGGACCAAAGTCAATCCGACCACTCACATGTACACTCACTGTGAG
ATCCACCCTAGCATGCTTCTAGGTATCTGTGCCAGCATCATTCCGTTCCCGGATCACAACCAGGTAC
CCCTGAACGCGTCCCCCAATCCCCTGATCTCCTACCCCTCACGCACAAACACCACAAACACAAAAC
GTCTCCATTCCCTGCATCGC---------AGACTTC--AGCA---
TTTGGGGAAATGAAAGCTAACCTTATATCTTCGTCTCAATAGGTTCACCTCCAGACCGGCCAATGC
GTAAGAATTACCTACCCCTACAACTACCGACTA-
TCAACGCGAAAAACGATAGCGGGGCTCACGAATATTATATAGGGTAACCAAATTGGTGCTGCTTTC
TGGCAAACCATCTCTGGCGAGCACGGTCTCGACAGCAATGGCGTGTACGTATTTCATTGGTCAATC
CTCGGATGGGAGTTGGATATTAATGGTTTATCAACAGCTACAACGGAACCTCTGAGCTACAGCTGG
AGCGCATGAGCGTCTACTTCAACGAGGTACGTCGAAATCAAGAACAAGAATACATGCGCAAAGCC
CGTTACTAATTATCTTGACTTGTGCAGGCTTCCGGCAACAAGTATGTTCCCCGTGCCGTCCTCGTCG
ATCTCGAGCCCGGTACCATGGACGCCGTCCGTGCTGGTCCCTTCGGTCAACTCTTCCGACCCGACA
ACTTCGTCTTTGGTCAATCCGGTGCCGGCAACAACTGGGCCAAGGGTCACTACACTGAGGGTGCTG
AGCTGGTCGACCAGGTTTTGGATGTTGTTCGTCGTGAGGCTGAGGGCTGTGACTGCCTCCAGGGTTT
CCAGATTACCCACTCGCTCGGTGGTGGTACCGGTGCCGGTATGGGTACCTTGTTGATCTCCAAGATC
CGCGAGGAGTTCCCCGACCGAATGATGGCCACCTTCTCCGTCGTTCCCTCCCCCAAGGTCTCTGACA
CCGTTGTCGAGCCTTACAACGCTACCCTTTCCGTCCACCAGCTGGTCGAGAACTCGGACGAGACCT
TCTGCATTGATAACGAGGCTCTATACGATATCTGCATGCGTACCCTGAAGCTATCCAACCCCTCGTA
CGGTGACCTGAACCACCTGGTCTCTGCTGTCATGTCGGGTGTCACCACTTGCCTGCGATTCCCCGGT
CAGCTGAACTCTGACCTCCGCAAGTTGGCTGTCAACATGGTGCCCTTCCCCCGTCTGCACTTCTTCA
TGGTCGGCTTCGCTCCTCTGACCAGCCGCGGCGCTTACTCCTTCCGTGCTGTCACCGTTCCCGAGTT
GACGCAGCAGATGTTCGACCCCAAGAACATGATGGCTGCCTCTGACTTCCGCAACGGTCGTTACCT
GACATGCTCTGCCATCTTGTAAGATACTAGCTCCAAATCGTCAATAAATGTTTATTGCTAACCCTT
AATCTCTAGCCGTGGCAAGATCTCCA

>Hypoxylon_sporistriatatunicum_UCH9542

-
CATTACTGAGTTCTCCAAACTCCCAACCCTTTGTGAATCGTATTCCTGTTGCCTCGGCGGCCAGCGT
ACCTATTCGATAGCTACCCTGGAGTCACCTACCCTGGAGGTGGCTACCCTGGAGCTACCCTGTAGTT
GCAC-GAATGCCCCGCCGGTGGACCACCCAACTCTGA-TTTATAGTGTATCTCTGAATTCTTTAA--
AAATTAGTTAAAACTTTCAACAACGGATCTCTTGGTTCTGGCATCGATGAAGAACGCAGCGAAATG
CGATAAGTAATGTGAATTGCAGAATTCAGTGAATCATCGAATCTTTGAACGCATATTGCGCCCAGT
AGTATTCTGCTGGGCATGCCTATTCGAGCGTCATTTCAACCCTTACG-
CCCTGCAGCGTAGCGTTGGGACTCTACTCCTCCGGGGGCAGTTCCCTAAAACCAGTGGCGGTGTTC-
GGTACACTCATAGCGTAGTAAACATCCTCGCTTCTGTAGTATACCGTCCTATCGGCCGTAAAACCCC
CTAT-
ATTCTAATGGTTGACCTCGGATTAGGTAGGAATACCCGCTGAACTTAAGCATATGAAGCGGCAACA
GCTCAAA-TTTGAAATCTGGCC----TTC-----G-
GGTCCGAATTGTAATTTGCAGAGGATGCTTTTGGTGCGGT-
GCCTTCCGAGTTCCCTGGAACGGGACGCCAGAGAGGGTGAGAGCCCCGTACGGTTGGA-
CACCTACCCTATATATAGCTCCTTCGACGAGTCGAGTAGTTTGGGAATGCTGCTCTAAATGGGAGG
TAAATTTCTTCTAAAGCTAAATACCGGCCAGAGACC-
GATAGCGCACAAGTAGAGTGATCGAAAGATGAAAAGCACTTTGAAAAGAGGGTTAAATAGCACGT
GAAATTGTTGAAAGGGAAGCG-TTTGTGACCAGACTTTTTCCAGGCGGATCATCCGGTGTTC--
TCACCGGTGCACTTCGCCTGG--TTTAGGCCAGCATCGGTTTC---
CGTAGGGGGATAAAAACCTGGGGCATGTGGCTC----TCTCGAGAGTGTT-
ATAGCCCCTCGTATAATACCCCTCC-GGGGACCGAGGACCGCGCT-TCG---
GCAAGGATGCTGGCGTAATGGTTATCAACGACCCGTCTTGAAACACGGACCAAGGAGTCGAACAT
TTGTGCGAGTGTTTGGGTG--TTAAACCCTCACGCGTAATGAAAGTGAACGGAGGTGAGAGCCCTT--
---CG---GGGTGCATCATCGACCGATCCTGATG-
TCTTCGGATGGATTTGAGTAAGAGCATAACTGTTCGGACCCGAAAGATGGTGAACTATGCGTGGAT
AGGGTGAAGCCAGAGGAAACTCTGGTGGAGGCTCGCAGCGGTTCTGACGTGCAAATCGATCGTCA
AATCTGCGCATGGGGGCGAAAGACTTATCGA-

ACCATCTAGTAGCTGGTTACCGCCGAAGTTTCCCTCAGGATAGCAGTGT--TG-
TTTTCAGTTTTATGAGGTAAAGCGAATGATTAGGGACTCGGGGGCGCTTTATTGCCTTCATCCATTC
TCAAACTTTAAATATGTAAGAAGCCTTTGTTACTTCATTGAACGTAGGCATTCGAATGTATCAACAC
TAGTGGGCCATTTTTGGTAAGCAGAACTGGCGATGCGGGATGAACCGAACGCGAGGTTAAGGTGC
CAGAGTGGACGCTCATCAGACACCACAAAAGGTGTTAGTACATCTAGACAGTAGGACGGTGGCCA
TGGAAGTCGGAATCCGCTAAGGACTGTGTAACAACTCACCAACCGAATGTACTAGCCCTGAAAATG
GATGGCGCTCAAGCGTCT-
CACCCATACCTCGCCCTTAGGGTAGAAACGATGCCCTAAGGACGCTGCATCGATCTGAATAAGCAT
TTTGAAATTGCCCTAGCTGCTAAGCCTACCATCATCTCAAACGGCCTCAAGTACTCGCTCGCCACAG
GAAACTGGGGCGATCAGAAAAAGGCCATGAGCTCGACGGCCGGTGTGTCGCAAGTCTTGAACCGA
TACACTT------TCGCCTCTA---
CGCTTTCTCATTTGAGAAGAACTAACACGCCCATTGGGCGAGATGGAAAGCTGGCGAAGCCTCGAC
AGCTTCACAATACTCATTGGGGTTTGGTTTGCCCGGCTGAGACGCCTGAGGGTCAGGCTTGTGGTCT
GGTGAAGAACTTGTCGCTGATGTGTTCGATCAGTGTCGGTACGTCCACAGATCCTATCGTAGATTA
CATGATTACGAGAAACATGGAGGTTCTCGAAGAGTACGAACCGCTGCGATACCCCGACGCCACCA
AGATCTTCCTCAACGGCTCTTGGATCGGCGTGCACCAGAATCCCAAGGCTCTAGTGAGAGATGTTC
AGAACCTGCGCCGAACCAATCAAATCCCGGCTGAGGTGTCGCTAGTCCGCGACATACGCGACCGTG
AATTCAAAATCTTCTCAGACGCCGGTCGTGTTATGCGCCCTATGTTTGTTGTACAGCAAGAAGAT----
----
ACCGAGGCTGGCGCTAAGAAGGGAACGTTAGCTCTCACTAAGGAGATGATCCAGAAGCTCGAGGC
AGATGTCGAGATAGATCCTGAGAGCGATGAGTACTATGGCTGGCAAGGCTTGGTCAACGAAGGCG
CCATCGATTATCTCGACGCCGAGGAGGAAGAAACAGCCATGATCTGCATGACGCCTGAGGACCTTG
AGACCTACCGTCAGGCTAAAGCCGGATTCGAGGTGTCCCAGGATAACGGA------
GACGAGGTTAATAAACGACTGAGGACCAAGATTAACCCGACCACGCACATGTACACTCACTGCGA
GATCCATCCCAGCATGCTTCTAGGTATTTGCGCAAGCATTATTCCGTTCCCGGATCATAATCAGGTA
CCCCTGAACGCGTCCCCCAACCCCCTGATCTCCTACCCCTCACCCACAAACACCAAAAAC--------
TCTCCATCCCCTGCAACGC---------AGACATC--AGCA---
TCCAGAAAATTGAAAGCTAACCTTATCTCTTCGTCTCAATAGGTTCACCTCCAGACCGGCCAATGC
GTAAGAACTATACCCACCTATAACCATCGACCA-TCAATGCGAAAAATAATGGCGGGGCTCACGA--
ATTTTATAGGGTAACCAAATTGGTGCTGCTTTCTGGCAAACCATCTCTGGCGAGCACGGTCTCGAC
AGCAATGGCGTGTACGTATTTCATTGGTCAATTGTCGAATTGGAATTGGAAATTAATGATTTATCAT
TAGATACAACGGAACCTCAGAGCTCCAGCTTGAGCGCATGAGCGTCTACTTCAACGAGGTAGGTCA
AAATCAACAAGAAGAATATTTGCGCAGGGTCAGTCACTAAT----
TCAACATGCGCAGGCTTCCGGCAACAAGTATGTTCCCCGTGCCGTCCTCGTCGATCTTGAGCCCGGT
ACCATGGACGCCGTCCGTGCTGGTCCCTTCGGTCAGCTCTTCCGACCCGACAACTTCGTCTTTGGTC
AATCCGGTGCTGGCAACAACTGGGCCAAGGGTCACTACACTGAGGGTGCTGAGCTTGTTGACCAGG
TTTTGGATGTCGTTCGTCGTGAGGCTGAGGGCTGCGACTGCCTTCAGGGTTTCCAGATTACCCACTC
GCTTGGTGGTGGTACCGGTGCCGGTATGGGTACCTTGTTGATCTCCAAGATCCGCGAGGAGTTCCC
CGACCGAATGATGGCCACTTTCTCCGTCGTTCCCTCCCCCAAGGTCTCCGACACCGTTGTCGAGCCT
TACAACGCTACCCTCTCCGTCCACCAGCTGGTCGAGAACTCGGACGAGACCTTCTGCATTGATAAC
GAGGCTCTGTACGATATCTGCATGCGTACCCTGAAGCTATCCAACCCCTCGTACGGTGACCTGAAC
CACCTGGTCTCCGCCGTCATGTCGGGTGTTACCACTTGCTTGCGATTCCCTGGTCAGCTGAACTCTG
ACCTCCGCAAGTTGGCTGTCAACATGGTGCCCTTCCCTCGTCTGCACTTCTTCATGGTCGGCTTCGC
TCCCCTGACCAGCCGTGGCGCTTACTCCTTCCGTGCTGTCACCGTTCCCGAGTTGACGCAACAGATG
TTCGACCCCAAGAACATGATGGCTGCCTCCGATTTCCGCAACGGTCGCTACCTGACGTGCTCTGCC
ATCTTGTAAGATACCCACCTCACGTTATTGATAGGCCCTCATTTGCTAACGTTTAA--
TCCAGCCGTGGCAAGATCTCCA

>Hypoxylon_isabellinum_MUCL53308

TTCTAAAAACTCCCAACCCTTTGTGAACCTTACCTATGTTGCCTCGGCGCCGAGCGCAGCTACCCTG
GAGCTACTTTGAAGCTACCTACCCTGTAGTGAGCTACCCTGGAGCTACCCCGGAGTTGCAT-
TTACGCTCCGCCGATGGACCAGTAAACTCTGTTTTTCCCAGTATATCTCTGAATTCTTTAACAAAAA
TAGTTAAAACTTTCAACAACGGATCTCTTGGTTCTGGCATCGATGAAGAACGCAGCGAAATGCGAT
AAGTAATGTGAATTGCAGAATTCAGTGAATCATCGAATCTTTGAACGCATATTGCGCCCAGTAGTA
TTCTACTGGGCATGCCTATTCGAGCGTCATTTCAACCCTTATG-
CCCTGTAGCATAGTGTTGGGGCTCTACT----GAAAGGTAGTCCCCGAAAACCAGTGGCGGTGTTC-

GGTACACTCATAGCGTAGTAATTTTTCTCGCTTCTGACGTGGCCTGAATCTTCGG------------------------
--TGAAGCGGCAACAGCTCAAA-TTTGAAATCTGGCC----CTC-----G-
GGTCCGAATTGTAATTTGTAGAGGATGCTTTTGGTGCGGT-
GCCTTCTGAGTTCCCTGGAACGGGACGCCAGAGAGGGTGAGAGCCCCGTACGGTTGGC-
CACCTAGCCTCTATATAGCTCCTTCGACGAGTCGAGTAGTTTGGGAATGCTGCTCTAAATGGGAGG
TAAATTTCTTCTAAAGCTAAATACCGGCCAGAGACC-
GATAGCGCACAAGTAGAGTGATCGAAAGATGAAAAGCACTTTGAAAAGAGGGTTAAATAGCACGT
GAAATTGTTGAAAGGGAAGCG-TTTGTGACCAGACTTTCTCCAGGCGGATCATCCGGTGTTC--
TCACCGGTGCACTTCGTCTGG--TGTAGGCCAGCATCGGTTTC---
CGTAGGGGGATAAAAGCTCAGGGAATGTGGCTC----CTCCGGGAGTGTT-
ATAGCCCTCTGTATAATACCCTTCC-GGGGACCGAGGACCGCGCT-TCG---
GCAAGGATGCTGGCGTAATGGTTATCAACGACCCGTCTTGAAACACGGACCAAGGAGTCGAACAT
TTGTGCGAGTGTTTGGGTG--TTAAACCCTCACGCGTAATGAAAGTGAACGGAGGTGAGAGCCCTT--
---CG---GGGTGCATCATCGACCGATCCTGATG-
TCTTCGGATGGATTTGAGTAAGAGCATAACTGTTCGGACCCGAAAGATGGTGAACTATGCGTGGAT
AGGGTGAAGCCAGAGGAAACTCTGGTGGAGGCTCGCAGCGGTTCTGACGTGCAAATCGATCGTCA
AATCTGCGCATGGGGGCGAAAGACTTATCGA-
ACCATCTAGTAGCTGGTTACCGCCGAAGTTTCCCTCAGGATAGCAGTGT--TG-
TTTTCAGTTTTATGAGGTAAAGCGAATGATTAGGGACTCGGGGGCGCTTTATTGCCTTCATCCATTC
TCAAACTTTAAATATGTAAGAAGCCTTTGTTACTTTATTGAACGTAGGCATTCGAATGTATCAACAC
TAGTGGGCCATTTTTGGTAAGCAGAACTGGCGATGCGGGATGAACCGAACGCGGGGTTAAGGTGC
CAGAGTGGACGCTCATCAGACACCACAAAAGGTGTTAGTACATCTAGACAGC-
GGACGGTGGCCATGGAAGTCGGAATCCGCTAAGGACTGTGTAACAACTCACCGGCCGAATGTACT
AGCCCTGAAAATGGATGGCGCTCAAGCGTCT-
CACCCATACCCCGCCCTTAGGGTAGAAACGATGCCCTAAGG--

CCCCTGAACGCGTCCCCCAACCCCCTGATCTCCTGCCCCTCACTCACAAACACCACAAATCCAAAA
CGTTCCAGTCTCCTACATCGC---------AGACTTC--AGCA---
TTTTGAGAAACGAAAGCTAACCTTATCTCGTC--
TCAAATAGGTTCACCTCCAGACCGGCCAATGCGTAAGAATTGCCTCCACCTACAACCATCGAATA-
TCGACGCGAAAAATAATGGCGGGGCTCACGA--
ATATTGTAGGGTAACCAAATCGGTGCTGCTTTCTGGCAAACCATCTCTGGCGAGCACGGTCTCGAC
AGCAATGGCGTGTACGTATTTCATTGGTCAATTCTGGGATGGGAATTGGGAATTAATGGGTTATCT
ACAGCTACAACGGAACCTCTGAGCTCCAGCTGGAGCGCATGAGCGTCTACTTCAACGAGGTACGCC
GAAATCAACAGCAAAAATACATGCGCAAGGACCGTTACTAAT----
TCAACATGCGCAGGCTTCCGGTAACAAGTACGTTCCCCGTGCCGTCCTCGTCGATCTCGAGCCCGG
TACCATGGACGCCGTCCGTGCTGGTCCCTTCGGTCAGCTCTTCCGACCTGACAACTTCGTCTTTGGT
CAATCCGGTGCCGGCAACAACTGGGCCAAGGGTCACTACACTGAGGGTGCTGAGCTTGTCGACAA
CGTTTTGGATGTCGTTCGTCGTGAGGCTGAGGGCTGCGATTGCCTTCAGGGTTTCCAGATTACCCAC
TCGCTCGGTGGTGGTACCGGTGCCGGTATGGGTACCTTGTTGATCTCCAAGATCCGCGAGGAGTTC
CCCGACCGAATGATGGCTACTTTCTCCGTCGTTCCCTCCCCCAAGGTCTCCGACACCGTCGTCGAGC
CCTACAACGCTACTCTTTCCGTCCACCAGCTGGTCGAGAACTCGGACGAGACCTTCTGCATTGATA
ACGAGGCTCTGTACGACATCTGCATGCGTACCCTGAAGCTATCCAACCCCTCGTACGGTGACCTGA
ACCACCTGGTCTCCGCCGTCATGTCGGGTGTTACCACTTGCTTGCGTTTCCCTGGTCAGCTGAACTC
TGATCTCCGCAAGTTGGCTGTCAACATGGTGCCATTCCCTCGTCTGCACTTCTTCATGGTCGGCTTC
GCTCCCCTGACCAGCCGCGGTGCTTACTCTTTCCGTGCCGTCACCGTCCCCGAGTTGACGCAACAGA
TGTTCGACCCCAAGAACATGATGGCCGCTTCCGACTTCCGCAACGGTCGCTACCTGACATGTTCTGC
CATCTTGTAAGATAT--
ACCCTAAATCTCCAATAAGTGCTTATTTGCTAACCCTGAATTTCTAGCCGTGGCAAGATCTCCA

>Hypoxylon_fuscum

-CATTACTGAGTTCTTACAAACTCCAACCCTTTGTGAACCATACCACTGTTGCCTCGGCGTG-
AGCGCGGCTGCGTGGTAGCTACCCGGTAGTCAC---------------
CTACCCGGTACCTACCCTGTACGTCTGCGTATAAGCCCGCCGAAGGACCACTAAACTCTGTTTG--
ACAGTGTAT-TCTGAATGCTTCAACT-
AAATAGTTAAAACTTTCAACAACGGATCTCTTGGTTCTGGCATCGATGAAGAACGCAGCGAAATGC
GATAAGTAATGTGAATTGCAGAATTCAGTGAATCATCGAATCTTTGAACGCACATTGCGCCCATTA
GTATTCTAATGGGCATGCCTATTCGAGCGTCATTTCGACCCTGAAGCCCTGGTTGCTTCGCGTTGGG
ACTCTACTGGCTACCCTGTAGTTCCCTAATGACAGTGGCGGAGTTCAGGTGTACTCTCAGCGTAGTA
ATTCTTCTCGCTTTTGCAGTAGCC-TGGTCACCAGCCGTAAAACCCT-----
TTTCTAGTGGTTGACCTCGGATTAGGTAGGAATACCCGCTGAACTTAAGCATA---------------------------
-----CC----CTC-----GTGGTCCGAGTTGTAATTTGTAGAGGATGCTTTTGGTGCGGT-
GCCTTCCGAGTTCCCTGGAACGGGACGCCAGAGAGGGTGAGAGCCCCGTACGGTTGGA-
CACCTACCCTATATATAGCTCCTTCGACGAGTCGAGTAGTTTGGGAATGCTGCTCTAAATGGGAGG
TAAATTTCTTCTAAAGCTAAATACCGGCCAGAGACC-
GATAGCGCACAAGTAGAGTGATCGAAAGATGAAAAGCACTTTGAAAAGAGGGTTAAATAGCACGT
GAAATTGTTGAAAGGGAAGCG-TTTGCGACCAGACCTTTTCCCGGCGGATCATCTGGTGTTC--
TCACCGGTGCACTTCGCTTGG--TTTAGGCCAGCATCGGTTTC---
TTTAGGGGGATAAAGGCGTTGGGAACGTAGCTC----TTTCGGGAGTGTT-
ATAGCCCTTCACGTAATACCCTTCG-AGGGACCGAGGACCGCGCA-TT----
GCAAGGATGCTGGCGTAATGGTCGTCAACGACCCGTCTTGAAACACGGACCAAGGAGTCGAACAT
TTGTGCGAGTGTTTGGGTG--TTAAACCCTCACGCGTAATGAAAGTGAACGGAGGTGAGAGCCCTT--
--AC----GGGTGCATCATCGACCGATCCTGATG-
TCTTCGGATGGATTTGAGTAAGAGCATAACTGTTCGGACCCGAAAGATGGTGAACTATGCGTGGAT
AGGGTGAAGCCAGAGGAAACTCTGGTGGAGGCTCGCAGCGGTTCTGACGTGCAAATCGATCGTCA
AATCTGCGCATGGGGGCGAAAGACTTATCGA-
ACCATCTAGTAGCTGGTTACCGCCGAAGTTTCCCTCAGGATAGCAGTGT--TG-
TATTCAGTTTTATGAGGTAAAGCGAATGATTAGGGACTCGGGGGCGCTTTATTGCCTTCATCCATTC
TCAAACTTTAAATATGTAAGAAGCCCTTGTTGCTTAATTGAACGTGGGCATTCGAATGTATCAACA
CTAGTGGGCCATTTTTGGTAAGCAGAACTGGCGATGCGGGATGAACCGAACGCGGGGTTAAGGTG
CCAGAGTGGACGCTCATCAGACACCACAAAAGGTGTTAGTACATCTTGACAGCAGGACGGTGGCC
ATGGAAGTCGGAATCCGCTAAGGACTGTGTAACAACTCACCTGCCGAATGTACTAGCCCTGAAAAT
GGATGGCGCTCAAGCGTCT-
CACCCATACCTCGCCCTTAGGGTAGAAACGATGCCCTAAGGGCGCTGCATCGAGAACAACAAGCA
CTTCGAGATTGCTTTAGCAGCCAAGCCAGCCATCGTGACCAACGGTCTCAAGTATTCGCTCGCTAC
AGGCAACTGGGGTGACCAAAAGAAGGCGGCGAGCTCGACAGCCGGTGTGTCGCAAGTGCTCAACA
GATACACAT------TCGCGTCTA---
CTCTATCTCATTTGAGAAGAACGAACACGCCCATCGGCCGAGACGGCAAGCTTGCGAAACCCCGAC
AGCTTCACAACACGCATTGGGGTCTAGTCTGCCCGGCCGAGACGCCCGAGGGACAGGCTTGTGGTT
TGGTCAAGAATCTGTCCTTGATGTGCTCCATCAGTGTGGGCACGTCCACCGATCCTATCGTCGATTA
TATGATCACGAGAAACATGGAGGTACTCGAAGAATATGAACCTTTGCGTTACCCGGATGCCACCAA
GATCTTCTTGAACGGTTCGTGGATCGGTGTACACCAGGATCCCCAAGCTCTAGTTAAGGACGTGCA
GCGTTTGCGTCGCTCCGGCCAGATTCCTCCTGAAGTGTCGCTGGTGAGGGACATCCGTGACCGTGA
GTTCAAGATCTTCTCCGATGCTGGTCGAGTCATGCGCCCCTTGTTCGTTGTACAGCAAGAGGAT------
----
GAGGAAGGGACCGATGACGAACCGGGCAAGGGCAAGGGAACCTTGGCTCTCACCAAGGACATGAT
CCAGAGGCTGGAGGCGGACAACGAAGTCGACCCTAGCAGCGAAGAGTACTTTGGTTGGGAAGGCT
TGGTCGGAGCGGGTGTCATCGATTACCTAGATGCGGAAGAGGAAGAGACCGCTATGATTTGCATG
ACCCCCGAAGACCTGGACATCTACCGTAGGACCAAGGCCGGCGACGAAGTCTATCAGGATAACGG
A------
GAGGAGATTAACAAGCGCCTGAAGACGAAGATCAACCCGACCACTCACATGTACACCCACTGTGA
GATTCATCCCAGCATGCTGCTAGGTATTTGCGCCAGCATCATTCCGTTCCCGGATCACAACCAGGTA
CCCCTGAACGCGTCCCTCGACTCCCCTGACTTCTACCCCTCACGCACAAACAATACGACAACCGCA
TCGTGCCATTCTCTACATCGCAAACTGTTCAATTGATATGAT----
ATTGGAATATTAAAAGCTAACCGTGTCTCTTCCTCTTAATAGGTCCACCTCCAGACCGGCCAATGCG
TAAGATTGATATC-----------

CCCGACGAAACAAGGCGATAGAAGATGGCGGGGCTCACACGAATATCATAGGGTAACCAAATCGG
TGCTGCTTTCTGGCAAACCATCTCTGGCGAGCACGGTCTCGACAGCAATGGCGTGTACGTGTTGTAT
TGGTCAATTCCTAAGAGACAAACACAAATTGACAA-
TCTAAAATAGGTACAACGGAACTTCCGAGCTTCAGCTGGAGCGCATGAGCGTCTACTTCAACGAGG
TACGCTATCTTGAAA---
AAACATAGATGCGCAGACCGTTTTACTAATCACCCCAACATGCACAGGCGTCTGGTAACAAGTACG
TGCCTCGTGCCGTCCTCGTCGATCTCGAGCCCGGTACCATGGATGCTGTCCGTGCTGGTCCCTTCGG
TCAACTCTTCCGACCCGACAACTTCGTCTTTGGTCAATCCGGTGCCGGAAACAACTGGGCAAAGGG
TCATTACACCGAGGGTGCTGAGCTGGTTGACCAGGTTCTCGATGTCGTTCGTCGTGAGGCTGAGGG
CTGCGATTGCCTTCAGGGTTTCCAGATCACCCACTCGCTCGGTGGTGGTACTGGTGCCGGTATGGGT
ACCTTGTTGATTTCCAAGATTCGCGAGGAGTTCCCCGACCGCATGATGGCTACCTTCTCCGTCGTAC
CCTCCCCCAAGGTCTCCGACACCGTCGTCGAGCCTTACAACGCTACCCTCTCCGTCCATCAGCTGGT
CGAGAACTCGGATGAGACCTTCTGCATCGACAACGAGGCTCTGTACGATATCTGTATGCGTACTCT
GAAGCTATCCAACCCCTCGTACGGTGACCTGAACCACCTGGTCTCCGCCGTCATGTCTGGTGTTACT
ACCTGCTTGCGATTCCCCGGTCAGCTGAACTCTGATCTGCGCAAGCTGGCCGTCAACATGGTTCCTT
TCCCGCGTCTGCACTTCTTCATGGTCGGCTTCGCACCCCTGACCAGCCGTGGTGCTTACACCTTCCG
CGCCGTCACCGTTCCCGAGTTGACTCAGCAGATGTTCGACCCCAAGAACATGATGGCTGCCTCTGA
CTTCCGCAACGGTCGTTACCTGACATGCTCTGCCATCTTGTAAGTGACCTATCTTTATTA---
TATCTGCATATTAGTACTAATAACGACATTATAGCCGTGGCAAGGTCTCCA

>Hypoxylon_pseudofuscum

-CATTACTGAGTTCTTACAAACTCCAACCCTTTGTGAACCATACCACTGTTGCCTCGGCGCG-
AGCGCGGCTGCTTGGTAGCTACCCGGTAGTCAC---------------
CTACCCGGGAGCTACCCGGTACGTCTGCGTACAGGCCCGCCGAAGNNCCACCAAACTCTGTTTG--
ACAGTGTAT-TCTGAATGCTTCAACT-
AAATAGTTAAAACTTTCAACAACGGATCTCTTGGTTCTGGCATCGATGAAGAACGCAGCGAAATGC
GATAAGTAATGTGAATTGCAGAATTCAGTGAATCATCGAATCTTTGAACGCACATTGCGCCCATTA
GTATTCTAGTGGGCATGCCTATTCGAGCGTCATTTCGACCCTGAAGCCCTAGTTGCTTCGCGTTGGG
ACTCTACTGGCTACCCTGTAGTTCCCTAATGACAGTGGCGGAGTTCAGGTGTACTCTCAGCGTAGTA
ATTCATCTCGCTTTTGCAGTAGCC-TGGTCGCCGGCCGTAAAACCCCCTAT-TTTCTAGTGG-------------
------------------------------TGAAGCGGCAACAGCTCAAA-TTTGAAATCTGGCC----CTC-----
GTGGTCCGAGTTGTAATTTGTAGAGGATGCTTTTGGTGCGGT-
GCCTTCCGAGTTCCCTGGAACGGGACGCCAGAGAGGGTGAGAGCCCCGTACGGTTGGA-
CACCTACCCTATATATAGCTCCTTCGACGAGTCGAGTAGTTTGGGAATGCTGCTCTAAATGGGAGG
TAAATTTCTTCTAAAGCTAAATACCGGCCAGAGACC-
GATAGCGCACAAGTAGAGTGATCGAAAGATGAAAAGCACTTTGAAAAGAGGGTTAAATAGCACGT
GAAATTGTTGAAAGGGAAGCG-TTTGCGACCAGACCTTTTCCCGGCGGATCATCTGGTGTTC--
TCACCGGTGCACTTCGCTTGG--TTTAGGCCAGCATCGGTTTC---
TTTAGGGGGATAAAGGTGTTGGGAACGTAGCTC----TTTCGGGAGTGTT-
ATAGCCCTTCACGTAATACCCTTCG-AGGGACCGAGGACCGCGCA-TT----
GCAAGGATGCTGGCGTAATGGTCGTCAACGACCCGTCTTGAAACACGGACCAAGGAGTCGAACAT
TTGTGCGAGTGTTTGGGTG--TTAAACCCTCACGCGTAATGAAAGTGAACGGAGGTGAGAGCCCTT--
--AC----GGGTGCATCATCGACCGATCCTGATG-
TCTTCGGATGGATTTGAGTAAGAGCATAACTGTTCGGACCCGAAAGATGGTGAACTATGCGTGGAT
AGGGTGAAGCCAGAGGAAACTCTGGTGGAGGCTCGCAGCGGTTCTGACGTGCAAATCGATCGTCA
AATCTGCGCATGGGGGCGAAAGACTTATCGA-
ACCATCTAGTAGCTGGTTACCGCCGAAGTTTCCCTCAGGATAGCAGTGT--TG-
TATTCAGTTTTATGAGGTAAAGCGAATGATTAGGGACTCGGGGGCGCTTTATTGCCTTCATCCATTC
TCAAACTTTAAATATGTAAGAAGCCCTTGTTGCTTAATTGAACGTGGGCATTCGAATGTATCAACA
CTAGTGGGCCATTTTTGGTAAGCAGAACTGGCGATGCGGGATGAACCGAACGCGGGGTTAAGGTG
CCAGAGTGGACGCTCATCAGACACCACAAAAGGTGTTAGTACATCTTGACAGCAGGACGGTGGCC
ATGGAAGTCGGAATCCGCTAAGGACTGTGTAACAACTCACCTGCCGAATGTACTAGCCCTGAAAAT
GGATGGCGCTCAAGCGTCT-
CACCCATACCTCGCCCTTAGGGTAGAAACGATGCCCTAAGGGCGCTGCATCGAGAACAACAAGCA
CTTCGAGATTGCTCTAGCAGCCAAGCCGGCGATCGTGACCAACGGTCTCAAATATTCGCTCGCTAC
AGGCAACTGGGGCGACCAAAAGAAGGCGGCGAGCTCGACAGCCGGTGTGTCACAAGTACTTAACA
GATACACGT------TTGCGTCTA---

CTCTATCTCATTTGAGAAGAACGAACACGCCCATCGGCCGAGACGGCAAGCTTGCGAAGCCCCGAC
AGCTTCACAACACGCATTGGGGTCTCGTCTGCCCGGCCGAGACGCCCGAGGGACAGGCTTGCGGTT
TGGTCAAGAATCTGTCCTTGATGTGCTCCATCAGTGTGGGCACGTCCACCGACCCTATCGTCGACTA
TATGATCACGAGAAACATGGAGGTGCTCGAAGAATATGAACCGTTGCGTTACCCGGACGCCACCA
AGATCTTCTTGAACGGTTCGTGGATTGGTGTACACCAGGATCCCCAAGCGCTAGTGAAGGACGTGC
AGCGTTTGCGTCGCTCCGGCCAGATTCCTCCTGAAGTGTCACTAGTGAGGGACATCCGTGACCGCG
AGTTCAAGATCTTCTCCGATGCTGGTCGAGTCATGCGCCCCTTGTTTGTTGTTCAGCAAGAGGAC----
----
GAGGAAGGGACCGATGACGAGCCGGGCAAGGTCAAGGGAACCTTGGCTCTCACCAAGGACATGAT
CCAGAGGCTGGAGGCGGACAACGAAGTCGACCCCAGCAGCGAAGAGTACTTTGGTTGGGAAGGCT
TGGTCGGAGCAGGTGTCATCGATTACCTGGATGCGGAAGAAGAAGAGACCGCTATGATTTGCATG
ACCCCCGAAGATCTGGACATCTACCGCAGGACCAAGGCCGGCGACGAAGTCTATCAGGATAACGG
C------
GAGGAGATTAACAAACGCCTGAAGACGAAGATCAACCCGACCACTCACATATACACCCACTGTGA
GATTCATCCCAGCATGCTGCTAGGTATTTGCGCCAGCATCATTCCGTTCCCGGATCATAACCAGGTA

CGCGTCCCTCAACTCCCCTGACTTCTACCCCTCACGCACAAACAATACGACAACCGCATCGTGTCAT
TCTCTACACGGCAAACTGTTCAAGTAATATGAGAATAATGGGAATATCAAAAGCTAACCGTGTCTC
TTCATCTTAATAGGTCCACCTTCAGACCGGCCAATGCGTAAGATTGCTATC----------
CCCCGACGAAACAAGGCGATAGAAGAGGGCGGGGCTCACACGAATATCATAGGGTAACCAAATTG
GTGCTGCTTTCTGGCAAACCATCTCTGGCGAGCACGGTCTCGACAGCAATGGCGTGTACGTCTTCTA
TTGGTCAATTCGTAAGAGACGAATACAAATTGACAA-
TCTGAAATAGGTACAACGGAACTTCGGAGCTTCAGCTGGAGCGCATGAGCGTCTACTTCAACGAGG
TACGCTATCTTGAAA---
AGACATAGATGCGCAAGACGGTTTACTAATCACCCCAACATGCACAGGCGTCTGGTAACAAGTAC
GTGCCTCGTGCCGTCCTCGTCGATCTCGAGCCCGGTACCATGGATGCTGTCCGTGCCGGTCCCTTCG
GTCAACTCTTCCGACCCGACAACTTCGTCTTCGGTCAATCCGGTGCCGGAAACAACTGGGCCAAGG
GTCATTACACCGAGGGTGCTGAGCTGGTCGACCAGGTTCTCGATGTCGTTCGACGTGAGGCCGAGG
GCTGCGATTGCCTTCAGGGCTTCCAGATCACCCACTCGCTCGGTGGTGGTACTGGTGCCGGTATGG
GTACCTTGTTGATCTCCAAGATTCGCGAGGAGTTCCCCGACCGCATGATGGCCACCTTCTCCGTCGT
GCCCTCCCCCAAGGTCTCCGACACCGTCGTCGAGCCTTACAACGCTACCCTCTCAGTCCATCAGCTG
GTCGAGAACTCGGACGAGACCTTCTGCATCGACAACGAGGCTCTGTACGACATCTGCATGCGTACC
CTGAAGCTATCCAACCCCTCGTACGGTGACCTGAACCACCTGGTCTCCGCCGTCATGTCCGGTGTTA
CCACCTGCTTGCGATTCCCCGGTCAGCTGAACTCGGACCTGCGCAAGCTGGCCGTCAACATGGTTC
CCTTCCCGCGTCTGCACTTCTTCATGGTCGGCTTCGCGCCCCTGACCAGCCGTGGTGCTTACACCTT
CCGCGCCGTCACCGTTCCCGAGTTGACTCAGCAGATGTTCGACCCCAAGAACATGATGGCTGCTTC
TGACTTCCGCAACGGTCGTTACCTGACATGCTCTGCCATCTTGTAAGTGACCGATATATTACTTTAT
ATCTACGTATCAGTACTAATAACGATATTATAGCCGTGGCAAGGTCTCCA

>Hypoxylon_fuscoides_MUCL52670

-CATTACTGAGTTCTTACAAACTCCAACCCTTTGTGAACCATACCACTGTTGCCTCGGCGTG-
AGCGCGGCTGCCTGGTAGCTACCCGGTAGTCGC--------------
CTACCCGGTAGCTACCCTGTACGTCTGCGTACAGGCCTGCCGAAGGACCACCAAACTCTGTTTG--
ACAGTGTAT-TCTGAATGCTTCAACT-
AAATAGTTAAAACTTTCAACAACGGATCTCTTGGTTCTGGCATCGATGAAGAACGCAGCGAAATGC
GATAAGTAATGTGAATTGCAGAATTCAGTGAATCATCGAATCTTTGAACGCACATTGCGCCCATTA
GTATTCTAGTGGGCATGCCTATTCGAGCGTCATTTCGACCCTGAAGCCCTAGTTGCTTCGCGTTGGG
ACTCTACTGGCTACCCTGTAGTTCCCTAATGACAGTGGCGGAGTTCAGGTGTACTCTCAGCGTAGTA
ATTCTTCTCGCTTTTGCAGTAGCC-TGGTCGCCGGCCGTAAAACCCCCTAT-
TTTCTAGTGGTTGACCTCGGATTAGGTAGGAATACCCGCTGAACTTAAGCATATGAAGCGGCAACA
GCTCAAA-TTTGAAATCTGGCC----CTC-----
GTGGTCCGAGTTGTAATTTGTAGAGGATGCTTTTGGTGCGGT-
GCCTTCCGAGTTCCCTGGAACGGGACGCCAGAGAGGGTGAGAGCCCCGTACGGTTGGA-
CACCTACCCTATATATAGCTCCTTCGACGAGTCGAGTAGTTTGGGAATGCTGCTCTAAATGGGAGG
TAAATTTCTTCTAAAGCTAAATACCGGCCAGAGACC-
GATAGCGCACAAGTAGAGTGATCGAAAGATGAAAAGCACTTTGAAAAGAGGGTTAAATAGCACGT
GAAATTGTTGAAAGGGAAGCG-TTTGCGACCAGACCTTTTCCCGGCGGATCATCTGGTGTTC--

TCACTGGTGCACTTCGCTTGG--TTTAGGCCAGCATCGGTTTC---
TTTAGGGGGACAAAGGCGTTGGGAACGTAGCTC----TTTCGGGAGTGTT-
ATAGCCCTTCACGTAATACCCTTCG-AGGGACCGAGGACCGCGCA-TT----
GCAAGGATGCTGGCGTAATGGTCGTCAACGACCCGTCTTGAAACACGGACCAAGGAGTCGAACAT
TTGTGCGAGTGTTTGGGTG--TTAAACCCTCACGCGTAATGAAAGTGAACGGAGGTGAGAGCCCTT--
--AC----GGGTGCATCATCGACCGATCCTGATG-
TCTTCGGATGGATTTGAGTAAGAGCATAACTGTTCGGACCCGAAAGATGGTGAACTATGCGTGGAT
AGGGTGAAGCCAGAGGAAACTCTGGTGGAGGCTCGCAGCGGTTCTGACGTGCAAATCGATCGTCA
AATCTGCGCATGGGGGCGAAAGACTTATCGA-
ACCATCTAGTAGCTGGTTACCGCCGAAGTTTCCCTCAGGATAGCAGTGT--TG-
TATTCAGTTTTATGAGGTAAAGCGAATGATTAGGGACTCGGGGGCGCTTTATTGCCTTCATCCATTC
TCAAACTTTAAATATGTAAGAAGCCCTTGTTGCTTAATTGAACGTGGGCATTCGAATGTATCAACA
CTAGTGGGCCATTTTTGGTAAGCAGAACTGGCGATGCGGGATGAACCGAACGCGGGGTTAAGGTG
CCAGAGTGGACGCTCATCAGACACCACAAAAGGTGTTAGTACATCTTGACAGCAGGACGGTGGCC
ATGGAAGTCGGAATCCGCTAAGGACTGTGTAACAACTCACCTGCCGAATGTACTAGCCCTGAAAAT
GGATGGCGCTCAAGCGTCT-
CACCCATACCTCGCCCTTAGGGTAGAAACGATGCCCTAAGGGCGCTGCATCGAGAACAACAAGCA
CTTCGAGATTGCTCTAGCAGCCAAGCCAGCGATCGTGACCAACGGTCTCAAGTATTCGCTCGCTAC
AGGCAACTGGGGTGACCAAAAGAAGGCGGCGAGCTCGACAGCCGGTGTGTCACAAGTGCTTAACA
GATACACGT------TTGCGTCTA---
CTCTATCTCATTTGAGAAGAACGAACACGCCCATCGGCCGAGACGGCAAGCTTGCGAAGCCCCGAC
AGCTTCACAACACGCATTGGGGTCTAGTCTGCCCGGCCGAGACGCCCGAGGGACAGGCTTGCGGTT
TGGTCAAGAATCTGTCGTTGATGTGCTCCATCAGTGTGGGCACGTCCACCGACCCTATCGTCGACTA
TATGATCACGAGAAACATGGAGGTACTCGAAGAATATGAACCGTTGCGTTACCCGGACGCCACCA
AGATCTTCTTGAACGGTTCGTGGATTGGTGTACACCAGGATCCCCAAGCGCTAGTGAAGGACGTGC
AGCGTTTGCGTCGCTCCGGCCAGATTCCTCCTGAAGTGTCGCTAGTGAGGGACATCCGTGACCGTG
AGTTCAAGATCTTCTCCGATGCTGGTCGAGTCATGCGCCCCTTGTTCGTTGTTCAGCAAGAGGAT----
----
GAGGAAGGGACCGATGACGAGCCGGGCAAGGTCAAGGGAACCTTGGCTCTCACCAAGGACATGAT
TCAGAGGCTAGAGGCAGACAACGAAGTCGACCCCAGCAGCGAAGAGTACTTTGGTTGGGAAGGCT
TGGTCGGAGCAGGTGTCATCGATTACCTAGATGCGGAAGAAGAAGAGACCGCTATGATTTGCATG
ACCCCCGAAGATCTGGACATCTACCGCAGGACCAAGGCCGGCGACGAAGTCTACCAGGATAACGG
T------
GAGGAGATTAACAAGCGCCTGAAGACGAAGATCAACCCGACCACTCACATATACACCCACTGTGA
GATTCATCCCAGCATGCTGCTAGGTATTTGCGCCAGCATCATCCCGTTCCCGGATCACAACCAGGT
ACCCCTGAACGCGTCCCTCAACCCCTGTGACTTCTGCCCCTCACGCACAAACAATACGACAACCGC
ATT---------------TTGCA-ACWGTTCAAGTAATATGGAAAT--------
AGTAAAGAGCTAACCGTGTCTCTTCATCGTAATAGGTCCACCTTCAGACCGGCCAATGCGTAAGAT
TGCTATC-----------
CCCGACGAAACAAGGCGATAGAAGAGGGCGGGGCTCACACGAATATCATAGGGTAACCAAATCGG
TGCTGCTTTCTGGCAAACCATCTCTGGCGAGCACGGTCTCGACAGCAATGGCGTGTACGTCTTCTAT
TGGTCAATTCTGAAGAGACGAACACAAATTGACAA-
TCTGAAATAGGTACAACGGAACTTCGGAGCTTCAGCTGGAGCGCATGAGCGTCTACTTCAACGAGG
TACGCTATCTTGAAAACAACACACAGATGCGCAAGACGGTTTACTAATCACCCCAACATGCACAGG
CGTCTGGTAACAAGTACGTGCCTCGTGCCGTCCTCGTCGATCTCGAGCCCGGTACCATGGATGCTGT
CCGTGCCGGTCCCTTCGGTCAACTCTTCCGCCCCGACAACTTCGTCTTCGGTCAATCCGGTGCCGGA
AACAACTGGGCCAAGGGTCATTACACCGAGGGTGCTGAGCTGGTGGACCAGGTTCTCGACGTCGTT
CGTCGTGAGGCCGAGGGTTGCGATTGCCTTCAGGGCTTCCAGATCACCCACTCGCTCGGTGGTGGT
ACTGGTGCCGGTATGGGTACCTTGTTGATCTCCAAGATTCGCGAGGAGTTCCCCGACCGCATGATG
GCCACCTTCTCCGTCGTACCCTCCCCCAAGGTCTCCGACACCGTCGTCGAGCCTTACAATGCTACCC
TCTCGGTCCATCAGCTGGTCGAGAACTCGGACGAGACCTTCTGCATCGACAACGAGGCTCTGTACG
ACATCTGCATGCGTACCCTCAAGCTATCCAACCCCTCGTACGGTGACCTGAACCACCTGGTCTCCGC
CGTCATGTCCGGTGTTACCACCTGCTTGCGATTCCCCGGTCAGCTGAACTCGGACCTGCGCAAGCTG
GCCGTCAACATGGTTCCTTTCCCGCGTCTGCACTTCTTCATGGTCGGCTTCGCGCCCCTGACCAGCC
GTGGTGCTTACACCTTCCGCGCCGTCACCGTTCCCGAGTTGAYTCAGCAGATGTTCGACCCCAAGA
ACATGATGGCTGCTTCTGACTTCCGCAACGGTCGATACCTGACATGCTCTGCCATCTTGTAAGTCAC
CTATCTATTTCGTAATATCTACGCATCAGTACTAATAACGATATAATAGCCGTGGCAAGGTCTCCA

>Hypoxylon_eurasiaticum

-CATTACTGAGTTCTTACAAACTCCAACCCTTTGTGAACCATACCAATGTTGCCTCGGCGCG-
AGCGCGGCTGCTTGGTAGTTACCCCGTAGTCAC--------------
CTACCCGGTACCTACCCTGTACGTCTGCGTACAAGCCCGCCGAAGGACCACTAAACTCTGTTTG--
ACAGTGTAT-TCTGAATGCTTCAACT-
AAATAGTTAAAACTTTCAACAACGGATCTCTTGGTTCTGGCATCGATGAAGAACGCAGCGAAATGC
GATAAGTAATGTGAATTGCAGAATTCAGTGAATCATCGAATCTTTGAACGCACATTGCGCCCATTA
GTATTCTAGTGGGCATGCCTATTCGAGCGTCATTTCGACCCTGAAGCCCTAGTTGCTTCGCGTTGGG
ACTCTACTGGCTACCCTGTAGTTCCCTAAAGACAGTGGCGGAGTTCAGGTGTACTCTCAGCGTAGT
AATTCTTCTCGCTTTTGCAGTAGCC-TGGTCACCAGCCGTTAAACCCCCTAT-
TATCTAGTGGTTGACCTCGGATTAGGTAGGAATACCCGCTGAACTTAAGCATA---------------------------
----

GAAGGCCGCGAGCTCGACAGCCGGTGTATCACAAGTGCTGAACAGATACACAT------TCGCATCTA--
-
CCCTATCTCATTTGAGAAGAACGAACACGCCCATCGGCCGAGACGGCAAGCTTGCGAAGCCCCGA
CAGCTTCACAACACGCACTGGGGTCTAGTCTGCCCGGCCGAGACGCCCGAGGGACAGGCTTGCGGT
TTGGTCAAGAATCTGTCGTTGATGTGCTCCATCAGTGTGGGCACTTCCACCGATCCTATCGTCGATT
ATATGATCACGAGAAATATGGAGGTGCTCGAAGAATATGAACCGCTGCGTTACCCGGATGCCACA
AAGATCTTCTTGAACGGTTCGTGGATTGGTGTACACCAGGACCCCCAAGCGTTAGTTAAGGACGTG
CAGCGTCTGCGCCGCTCCGGCCAGATTCCTCCCGAAGTGTCACTAGTGAGGGACATCCGTGACCGT
GAATTCAAGATCTTCTCCGATGCGGGTCGAGTCATGCGCCCCTTGTTTGTTGTCCAGCAAGAGGAT--
----
GAGGAAGGGACTGACGACGAGCCGGGCAAGGGCAAGGGAACCTTGGCTCTCACCAAGGACATGAT
CCAGAGGCTGGAGGCAGACAACGAAGTCGACCCCAGCAGCGAAGAGTACTTTGGTTGGGAAGGCT
TGGTTGGAGCAGGTGTCATCGATTACCTAGATGCGGAAGAGGAGGAGACCGCCATGATTTGCATG
ACCCCCGAAGATCTGGATATCTATCGCAGAACCAAGGCCGGCGACGAAGTCTATCAGGATAACGG
C------
GAGGAGATTAACAAGCGCCTGAAGACGAAGATCAACCCGACCACTCACATGTACACCCACTGTGA
GATTCATCCCAGCATGCTGCTAGGTATTTGTGCCAGCATCATTCCGTTCCCTGACCACAATCAGGTA
---ATATCGCAAACTATTCAAGTGATA-------
-
ATTGGAATATGAAAAGCTAACCGTGTCTCTTCATCTTCATAGGTTCACCTTCAGACCGGCCAATGCG
TAAGAATGATATC-----------
CCGACGAAAACAAGGCGATAGAAGATTGCGGGGCTCACACGAATATCATAGGGTAACCAAATCGG
TGCTGCTTTCTGGCAAACCATCTCTGGCGAGCACGGTCTCGACAGCAATGGCGTGTATGTGTTTTAT
TGGTCAATTCTTAAATGATGAATACAAATTGACAAATCTAAAATAGGTACAATGGAACCTCGGAAC
TCCAGTTGGAGCGCATGAGCGTCTACTTCAACGAGGTACGCTATCTTGAAAACAACAGA-
AAATGCGCAAGACCCGTTACTAATCACCCCAATATGCACAGGCGTCTGGTAACAAGTACGTGCCTC
GTGCCGTCCTCGTCGATCTCGAGCCCGGTACCATGGATGCTGTCCGTGCTGGTCCCTTTGGTCAACT
CTTCCGACCCGACAACTTCGTCTTTGGTCAATCCGGTGCCGGAAACAACTGGGCGAAGGGTCATTA
CACCGAAGGTGCTGAGCTGGTTGACCAGGTTCTCGACGTCGTTCGTCGTGAGGCTGAGGGCTGCGA
TTGTCTTCAGGGTTTCCAGATCACCCACTCGCTCGGTGGTGGTACTGGTGCCGGTATGGGTACCTTG
TTGATCTCCAAGATTCGCGAGGAGTTCCCCGACCGCATGATGGCTACCTTCTCCGTCGTGCCCTCCC
CTAAGGTCTCCGACACCGTCGTCGAGCCTTACAACGCTACCCTCTCCGTCCATCAGCTGGTCGAGA
ATTCGGACGAGACCTTCTGCATCGACAACGAGGCTCTGTACGACATCTGCATGCGTACCCTTAAGC
TATCCAACCCCTCGTACGGTGACCTGAACCACCTGGTCTCTGCTGTCATGTCCGGTGTTACCACTTG

CTTGCGATTCCCCGGTCAGCTGAACTCTGATCTGCGCAAGCTGGCTGTCAACATGGTTCCTTTCCCG
CGTCTGCACTTCTTCATGGTCGGCTTTGCGCCCCTGACCAGCCGTGGTGCTTACACCTTCCGCGCCG
TCACCGTTCCCGAGTTGACGCAGCAGATGTTCGACCCCAAGAACATGATGGCTGCTTCTGACTTCC
GCAACGGTCGTTACCTGACATGCTCTGCCATCTTGTAAGTGACTATC--

>Hypoxylon_porphyreum

-CATTACTGAGTTCTACAAACTCCCAACCCTTTGTGAACCATACTGTCGTTGCCTCGGCGCG-
AGCGCGGCTACCCTGCAGCTACCCTGTAGTCGT--------------
CTACCCGGTAGCTACCCTGTATCTGCGT-
ACAAGGCCCGCCGGAGGACCATTCAACTCTGTTTTTACGGTGTAT-
TCTGAATGCTTCAACTATAATAGTTAAAACTTTCAACAACGGATCTCTTGGTTCTGGCATCGATGAA
GAACGCAGCGAAATGCGATAAGTAATGTGAATTGCAGAATTCAGTGAATCATCGAATCTTTGAACG
CACATTGCGCCCATTAGTATTCTAGTGGGCATGCCTATTCGAGCGTCATTTCAACCCTGAAGCCCTG
GCTGCTTCGCGTTGGGACCCTACAGGTAACCCTGTAGTTCCCTAAAGGCAGTGGCGGAGTTCGGGT
ATACTCTCAGCGTAGTAGTTCTTCTCGCTGTGGCAGTAGCCCTAGCTATCAGCCGTAAA---------------
--TGAAGCGGCAACAGCTCAAA-TTTGAAATCTGGCC----CTC-----
GTGGTCCGAATTGTAATTTGTAGAGGATGCTTTTGGTGCGGT-
GCCTTCTGAGTACCCTGGAACGGGTCGCCAGAGAGGGTGAGAGCCCCGTACGGTTGGA-
CACCTACCCTATATATAGCTCCTTCGACGAGTCGAGTAGTTTGGGAATGCTGCTCTAAATGGGAGG
TAAATTTCTTCTAAAGCTAAATACCGGCCAGAGACC-
GATAGCGCACAAGTAGAGTGATCGAAAGATGAAAAGCACTTTGAAAAGAGGGTTAAATAGCACGT
GAAATTGTTGAAAGGGAAGCG-TTTGCGACCAGACCTTCTCCTGGCGGATCATCCGGTGTTT--
TCACCGGTGCACTTTGCCAGG--TCTAGGCCAGCATCGGTTTC---
TTTAGGGGGATAAAGGCGTTGGGAACGTAGCTC----TTTCGGGAGTGTT-
ATAGCCCTTCACGTAATACCCTTCG-AGGGACCGAGGACCGCGCT-CT----
GCAAGGATGCTGGCGTAATGGTCGTCAACGACCCGTCTTGAAACACGGACCAAGGAGTCGAACAT
TTGTGCGAGTGTTTGGGTA--TTAAACCCTCACGCGTAATGAAAGTGAACGGAGGTGAGAGCCCTT--
--AC----GGGTGCATCATCGACCGATCCTGATG-
TCTTCGGATGGATTTGAGTAAGAGCATAACTGTTCGGACCCGAAAGATGGTGAACTATGCGTGGAT
AGGGTGAAGCCAGAGGAAACTCTGGTGGAGGCTCGCAGCGGTTCTGACGTGCAAATCGATCGTCA
AATCTGCGCATGGGGGCGAAAGACTTATCGA-
ACCATCTAGTAGCTGGTTACCGCCGAAGTTTCCCTCAGGATAGCAGTGT--TG-
TATTCAGTTTTATGAGGTAAAGCGAATGATTAGGGACTCGGGGGCGCTTTATTGCCTTCATCCATTC
TCAAACTTTAAATATGTAAGAAGCCCTTGTTACTTAATTGAACGTGGGCATTCGAATGTATCAACA
CTAGTGGGCCATTTTTGGTAAGCAGAACTGGCGATGCGGGATGAACCGAACGCGGGGTTAAGGTG
CCAGAGTGGACGCTCATCAGACACCACAAAAGGTGTTAGTACATCTTGACAGCAGGACGGTGGCC
ATGGAAGTCGGAATCCGCTAAGGACTGTGTAACAACTCACCTGCCGAATGTACTAGCCCTGAAAAT
GGATGGCGCTCAAGCGTCT-CACCCATACCTCGCCCTTAGGGTAGAAACGATGCCCTAAGG-----------
----
-------------------------GCATCGA---
CTCTGTCTCATCTGAGAAGAACTAACACGCCTATCGGTAGAGACGGCAAGCTTGCGAAGCCCCGAC
AGCTTCACAACACGCATTGGGGTCTAGTCTGTCCAGCCGAGACGCCCGAAGGACAGGCCTGTGGTC
TGGTCAAGAATCTGTCGTTGATGTGCTCTATCAGTGTGGGCACATCAACCGACCCCATCGTTGATTA
CATGATCACGCGAAACATGGAGGTGCTCGAAGAGTATGAACCACTACGTTACCCCGATGCCACAA
AGATCTTCTTGAACGGCTCGTGGATTGGTGTACACCAGGACCCGCAGGCTTTGGTCAAGGATGTGC
AGCGTTTGCGCCGTACTGGCCAGATTCCTCCTGAAGTGTCACTAGTTAGGGACATCCGTGACCGGG
AGTTCAAGATCTTTTCCGATGCTGGTCGTGTCATGCGCCCCTTGTTTGTTGTTCAGCAAGAGGAC-----
----

GAAGAGGGAACCGACGACGAACCCGGCAAGGGTAAGGGTACCTTGGCTCTCACCAAGGACATGAT
CCAGAGGCTAGAGGCAGACAATGATGTCGATCCCAACAGCGAAGAGTACTTTGGTTGGGAAGGCT
TGGTCGGAGCAGGTGTCATCGATTATCTAGATGCGGAAGAAGAAGAGACCGCCATGATTTGCATG
ACCCCTGAGGATCTGGAGATCTATCGCANGACCAAGGCCGGAGACGAAGTCTATCAGGATAATGG
C------
GAAGAGATTAACAAGCGCCTGAAGACTAAGATCAACCCTACTACTCACATGTATACCCACTGCGAG
ATTCATCCTAGCATGCTGTTAGGTATTTGCGCGTCGATCATTCCGTTCCCGGATCACAACCAGGTAC
CCCTGAACGCGTCCCTCAACCCCCCTGATTTCTGCCCCTCACGCACAAAC-ACACGAC-

ATCGCATCGTGCAATTCCCTACATCGCAAACTACTCGAGCGGT------------
GAAATTTTGAAAGCTAACCGTGTCTCTTCATCTCGATAGGTTCACCTTCAGACCGGCCAATGCGTAA
GAATTCTTA-----------TCCCGACG-
ACCAACGCGATAGAAGATTGCGGGGCTCACACGAATGTCATAGGGTAACCAAATCGGTGCTGCTTT
CTGGCAAACCATCTCTGGCGAGCACGGCCTCGACAGCAATGGCGTGTATGTGTTGCATTGGTCAAT
TC--
GGGAGACGAATAAAAATTGACAGCGTTTGAATAGGTACAACGGAACCTCCGAGCTCCAGCTGGAG
CGCATGAGCGTCTACTTCAACGAGGTACGCTATCTTGAAAA---
CAAATAAATGCGCAAGACCCGTTACTAATCACCCCAACATGCACAGGCGTCTGGTAACAAGTACGT
GCCTCGTGCCGTCCTCGTCGATCTCGAGCCCGGTACCATGGACGCTGTCCGTGCTGGCCCCTTCGGT
CAGCTCTTCCGACCCGACAACTTCGTCTTCGGCCAATCCGGTGCCGGAAACAACTGGGCGAAGGGT
CACTACACCGAGGGTGCTGAGCTGGTTGACCAGGTCCTTGATGTCGTCCGTCGTGAGGCTGAGGGC
TGCGACTGCCTTCAGGGCTTCCAGATCACCCACTCTCTCGGTGGTGGTACTGGTGCCGGTATGGGTA
CCCTGCTGATCTCCAAGATCCGCGAGGAGTTCCCCGACCGCATGATGGCCACCTTCTCCGTCGTCCC
CTCCCCCAAGGTCTCCGACACCGTCGTCGAGCCTTACAACGCCACCCTCTCCGTCCACCAGCTGGTC
GAGAACTCTGACGAGACCTTCTGCATCGACAACGAGGCCCTGTACGACATCTGCATGCGTACCCTG
AAGCTATCCAACCCCTCGTACGGTGACCTGAACCACCTGGTCTCCGCTGTCATGTCTGGTGTTACCA
CCTGCTTGCGATTCCCCGGTCAGCTGAACTCGGACTTGCGCAAGCTGGCCGTCAACATGGTTCCCTT
CCCGCGTCTGCACTTCTTCATGGTCGGCTTCGCTCCCCTGACCAGCCGTGGTGCTCACTCCTTCCGC
GCCGTCACCGTTCCCGAGTTGACTCAGCAGATGTTCGACCCCAAGAACATGATGGCCGCCTCTGAC
TTCCGCAACGGTCGTTACCTGACGTGCTCTGCCATCTTGTAAGTGCCTATC-
TCGATCATGTTTGATGCGTACC--TGCTAACGCCGAT---TCAGCCGCGGCAAGGTCTCCA

>Pyrenopolyporus_laminosus

-CATTACTGAGTTGTAAAAACTCCAAACCCTTTGCGAA-CCTATAATCGTTGCCTCGGCGTG-
AGCGCGCGTGCCCCGCAGCTGCTGC------------------------------GGGG----------
TACGCGCAAGACGGCCCGCCGGAGGACCAATTAACTCTTGTCAAAGCTTTGCTTCTCTGAATTATT
GTTCAAAATCAGTTAAAACTTTCAACAACGGATCTCTTGGTTCTGGCATCGATGAAGAACGCAGCG
AAATGCGATAAGTAATGTGAATTGCAGAATTCAGTGAATCATCGAATCTTTGAACGCACATTGCGC
CCATTAGTATTCTAGTGGGCATGCCTATTCGAGCGTCATTTCGACCCTTAAGCCCCAGTTGCTTAGC
GTTGGGAGTCTGCGTCCTCGGGCGCAGTTCCTCAAAGTCAGTGGCGGAGTTAGGTCACACTCTAAG
CGTAGTAAACGTTCTCGCTTCTGTGGTGTACCTAGCTGCCTGCCGTAAAACCCC-
TATACTTCTAGTGGTTGACCTCGAATTAGGTAGGAATACCCGCTGAACTTAAG----
TGAAGCGGCAACAGCTCAAA-TTTGAAATCTGGCC----CTC-----
GCGGTCCGAGTTGTAATTTGCAGAGGATGCTTTGGGTGCGGC-
GCCTTCCAAGTTCCCTAGAACGGGACGCCTTAGAGGGTGAGAGCCCCGTACGGTTGGA-
CGCCTAGCCTATGTATAGCTCCTTCGACGAGTCGAGTAGTTTGGGAATGCTGCTCTAAATGGGAGG
TAAATTTCTTCTAAAGCTAAATACCGGCCAGAGACC-
GATAGCGCACAAGTAGAGTGATCGAAAGATGAAAAGTACTTTGAAAAGAGGGTTAAATAGCACGT
GAAATTGTTGAAAGGGAAGCG-TTTGCGACCAGACCTTTTCCAGGCGGATCATCCGGCGTTC--
TCGCCGGTGCACTTCGCCTGG--TTTAGGCCAGCATCGGTTTT---
CCCAGGGGGATAAAGGCGGCGGGAACGTGGCTC----TTTCGGGAGTGTT-
ATAGCCCGCCGCGCAATACCCTTGG-GGGGACCGAGGACCGCGCT-TCG---
GCAAGGATGCTGGCGTAATGGTCGTCAACGACCCGTCTTGAAACACGGACCAAGGAGTCGAACAT
TTGTGCGAGTGTTTGGGTG--TCAAACCCTCACGCGTAATGAAAGTGAACGGAGGTGAGAGCCCCT--
---CG--CGGGTGCATCATCGACCGATCCTGATG-
TCTTCGGATGGATTTGAGTAAGAGCATAACTGTTCGGACCCGAAAGATGGTGAACTATGCGTGGAT
AGGGTGAAGCCAGAGGAAACTCTGGTGGAGGCTCGCAGCGGTTCTGACGTGCAAATCGATCGTCA
AATCTGCGCATGGGGGCGAAAGACTTATCGA-
ACCATCTAGTAGCTGGTTACCGCCGAAGTTTCCCTCAGGATAGCAGTGT--TG-
TCTTCAGTTTTATGAGGTAAAGCGAATGATTAGGGACTCGGGGGCGCTATTTTGCCTTCATCCATTC
TCAAACTTTAAATATGTAAGAAGCCCTTGTTACTTAATTGAACGTGGGCATTCGAATGTACCAACA
CTAGTGGGCCATTTTTGGTAAGCAGAACTGGCGATGCGGGATGAACCGAACGCGGGGTTAAGGTG
CCAGAGTGGACGCTCATCAGACACCACAAAAGGTGTTAGTACATCTTGACAGCAGGACGGTGGCC
ATGGAAGTCGGAATCCGCTAAGGACTGTGTAACAACTCACCTGCCGAATGTACTAGCCCTGAAAAT
GGATGGCGCTCAAGCGTCT-CACCCATACCTCGCCCTTAGGGTAGAAACGATGCCCTAAGG-----------
----

CGGGCAACTGGGGCGACCAGAAGAAGGCCATGAGCTCCACGGCCGGCGTGTCGCAGGTCCTAAAC
CGATATACGT------TCGCATCGA---
CTCTTTCTCATTTGCGACGAACGAACACGCCCATCGGAAGGGACGGCAAGCTTGCGAAGCCGCGAC
AGCTACACAACACCCACTGGGGTCTGGTATGTCCGGCCGAGACGCCCGAAGGCCAGGCCTGCGGG
CTGGTAAAGAATTTGTCGCTCATGTGCTCCATCAGCGTGGGTACCTCAACGGACCCGATCGTAGAT
TATATGATTACTAGGAATATGGAGGTCTTGGAGGAATATGAGCCGATGAGATATCCTAACGCCACC
AAGATCTTCCTCAACGGCTCTTGGATCGGCGTGCACCAAGATCCTAAGTCTCTGGTCAGGGACGTT
CAGCAGCTGCGCCGGGCCAACCAGATCCCCTCCGAGGTATCTTTAGTTCGAGACATCAGAGACCGC
GAATTCAAGATTTTTTCGGATGCTGGCCGCGTCATGCGTCCCCTGTTCGTTGTACAGCAGGAGGAT--
----

GACCCCGTCACCGGCGTCCAAAAGGGCCACCTGGCTCTCACGAAGACCCAGATCGCGAAGCTGGA
GGCAAGCATCGACGTAGATGTCGATGCTCCTGGCTACTATGGTTGGCAAGGGTTGGTTAACGACGG
TGTTATCGAGTATCTCGATGCGGAAGAGGAGGAGACAGCTATGATATGCATGACGCCCGAAGACTT
GGAAACCTATCGCATGACCAAGGCCGGCGTTGATTTGCCTCAGGACAACGGG------
GACGAGATTAACAAGCGCCTCAAGACCAAGGTCAACCCTACGACGCACATGTATACGCATTGCGA
GATCCACCCGAGTATGCTTCTAGGTATTTGCGCTAGCATTATTCCCTTCCCAGACCATAATCAGGTA
ACTCCAAAACCCCTG--------------TATCTGCCCCTCACGCATCCAGAACTCAACCA---
CATCCCGTGGTTCTCCAGAACACACTACCACGAAAGAACACGACTATTTGACGAATATTTGGAGCT
AACCATATCTCTTC-TCTTTATAGGTTCACCTTCAGACCGGCCAATGCGTAAGTACC----------
ATAATCGCCGACGAGACATAGCACTGGAATATAGCGGGGCTCATGCGAAGATCGTAGGGTAACCA
AATCGGTGCTGCTTTCTGGCAAACCATCTCGAGCGAGCACGGTCTCGACAGCAATGGCGTGTATGT
ATTTGAGTCGTCAACATGACCGCGAAGAACTCGAACTAATCGCCGATAAACAGCTACAATGGCACC
TCCGAGCTCCAGCTCGAGCGCATGAGCGTCTACTTCAACGAGGTACGGATTCGCGAAAGCAAGATG
GAAATAGATGGAACTAGTTACTGATCGTATCACCGCATGCAGGCCTCCGGTAACAAGTATGTTCCT
CGCGCCGTCCTCGTCGATCTCGAGCCCGGCACCATGGACGCCGTCCGCGCTGGTCCCTTCGGTCAG
CTCTTCCGACCGGATAACTTCGTTTTCGGTCAGTCCGGTGCCGGCAACAACTGGGCCAAGGGTCAC
TACACCGAGGGTGCCGAGCTGGTTGACAACGTCCTCGACGTCGTTCGCCGTGAGGCCGAAGGCTGC
GACTGCCTCCAGGGCTTCCAGATCACCCACTCCCTCGGTGGTGGTACCGGTGCTGGTATGGGTACC
CTGTTGATCTCCAAGATCCGCGAGGAGTTCCCCGACCGCATGATGGCTACCTTCTCCGTCATGCCTT
CCCCCAAGGTCTCCGACACCGTCGTCGAGCCTTACAACGCCACTCTCTCCGTCCACCAGCTGGTCG
AGAACTCTGACGAGACGTTCTGTATCGACAACGAGGCTCTGTACGATATCTGCATGCGTACGCTGA
AGCTGTCCAACCCCTCGTACGGCGATCTGAACCACCTGGTCTCCGCCGTCATGTCCGGCGTCACCA
CTTGTCTGCGTTTCCCCGGCCAGCTGAACTCTGATCTGCGCAAGCTCGCCGTGAACATGGTTCCCTT
CCCCCGTCTCCACTTCTTCATGGTCGGCTTCGCCCCTCTGACCAGCCGCGGCGCTCACTCCTTCCGC
GCCGTCACCGTTCCCGAGTTGACTCAGCAGATGTTCGACCCCAAGAACATGATGGCTGCCTCCGAC
TTCCGCAACGGTCGCTACCTGACGTGCTCGGCCATCTTGTAAGATACCCCCCCAGAATT-
TTTATTGGTATCAGCCTGCTGACCTAAA-------------------------

>Pyrenopolyporus_bambusicola_BCC89355

-CATTACTGAGTTGTAAAAACTCCAAACCCTTTGTGAA-CCTAGAATCGTTGCCTCGGCGTG-
AGCGCGCGTGCCCTGCAGCTACCCTGCA---------------------------GCGG----------
CGCGCGCAACACGGCCCGCCGGAGGACCAATTAACTCTTGTCAAAGCTTTGCTTCTCTGAATTAAT
GTTCAAAATTAGTTAAAACTTTCAACAACGGATCTCTTGGTTCTGGCATCGATGAAGAACGCAGCG
AAATGCGATAAGTAATGTGAATTGCAGAATTCAGTGAATCATCGAATCTTTGAACGCACATTGCGC
CCATTAGTATTCTAGTGGGCATGCCTATTCGAGCGTCATTTCGACCCTTAAGCCCCAGTTGCTTAGC
GTTGGGAGCCTGCGTCCTCGGGCGCAGCTCCTCAAAGTCAGTGGCGGAGTTAGGTCATACTCTAAG
CGTAGTAAACATTCTCGCTTCTGTGGTGTACCTAGCTGCCTGCCGTAAAACCCCCTATATTTCTAGT
GGTTGACCTCGAATTAGGTAGGAATACCCGCTGAACTTAAGCATATGAAGCGGCAACAGCTCAAA-
TTTGAAATCTGGCC----CTC-----GCGGTCCGAGTTGTAATTTGTAGAGGATGCTTTGGGTGCGGC-
GCCTTCCAAGTTCCCTAGAACGGGACGCCTTAGAGGGTGAGAGCCCCGTACGGTTGGA-
CGCCTAGCCCATGTATAGCTCCTTCGACGAGTCGAGTAGTTTGGGAATGCTGCTCTAAATGGGAGG
TAAATTTCTTCTAAAGCTAAATACCGGCCAGAGACC-
GATAGCGCACAAGTAGAGTGATCGAAAGATGAAAAGTACTTTGAAAAGAGGGTTAAATAGCACGT
GAAATTGTTGAAAGGGAAGCG-TTTGCGACCAGACCTTTTCCAGGCGGATCATCCGGCGTTC--
TCGCCGGTGCACTTCGCCTGG--TTTAGGCCAGCATCGGTTTT---
CCCAGGGGGATAAAGGCGGCGGGAACGTGGCTC----CTTCGGGAGTGTT-
ATAGCCCGCCGCGTAATACCCTTGG-GGGGACCGAGGACCGCGCT-TCG---

GCAAGGATGCTGGCGTAATGGTCGTCAACGACCCGTCTTGAAACACGGACCAAGGAGTCGAACAT
TTGTGCGAGTGTTTGGGTG--
TCAAACCCTCACGCGTAATGAAAGTGAACGGAGGTGAGAGCCCCTT-----G--
CGGGTGCATCATCGACCGATCCTGATG-
TCTTCGGATGGATTTGAGTAAGAGCATAACTGTTCGGACCCGAAAGATGGTGAACTATGCGTGGAT
AGGGTGAAGCCAGAGGAAACTCTGGTGGAGGCTCGCAGCGGTTCTGACGTGCAAATCGATCGTCA
AATCTGCGCATGGGGGCGAAAGACTTATCGA-ACCATCTAGTAGCTGGTA----------------------------
----

GGCAACTGGGGCGACCAAGAGAAGGCCATGAGCTCCACGGCCGGTGTGTCGCAGGTCCTAAACCG
ATATACGT------TCGCTTCGA---
CCCTTTCTCATTTGCGACGAACGAACACGCCCATCGGAAGGGACGGCAAGCTTGCGAAGCCGCGAC
AGCTACACAACACCCATTGGGGTCTGGTATGTCCGGCCGAGACGCCCGAAGGCCAAGCCTGCGGG
CTGGTAAAGAATCTGTCGCTCATGTGCTCCATCAGCGTGGGTACCTCGACGGACCCTATCGTAGAC
TACATGATTACTAGGAATATGGAAGTCTTGGAGGAATATGAGCCGATGAGATACCCTAACGCCACC
AAGATCTTCCTCAACGGCTCTTGGATCGGCGTGCACCAAGATCCTAAGTCTCTGGTTAGAGACGTT
CAGCAGCTGCGCCGGGCCAACCAGATCCCCTCCGAGGTATCGTTAGTTCGCGACATCCGAGACCGC
GAGTTTAAGATTTTCTCGGACGCCGGCCGCGTCATGCGTCCCCTGTTCGTTGTACAGCAAGAGGAT-
----

GACCCCGACACCGGCGTCCAAAAGGGTCACCTGGCTCTCACGAAGACCCAGATCGCGAAGCTTGA
GGCAAGCATCGACGTAGATGTCGATGCTCCTGGCTACTATGGTTGGCAAGGGTTGGTTAACGACGG
TGTTATCGAGTATCTCGATGCTGAAGAAGAGGAGACTGCTATGATATGCATGACTCCCGAAGACTT
GGAAACCTATCGCATGACCAAGGCCGGGGTTGATTTGCCCCAGGACAACGGG------
GACGAGATCAACAAGCGTCTCAAGACCAAGGTCAACCCTACGACGCACATGTATACGCATTGCGA
GATCCACCCGAGTATGCTTCTAGGTATTTGCGCTAGCATTATTCCCTTCCCAGACCATAATCAGGTA

GGTTCTCTAGAACACATTACCATGAAAAAACACGACTATTTGACGAATATTTGGAGCTAACCATAT
ATCTTC-TCTTTATAGGTTCACCTTCAGACCGGCCAATGCGTAAGTT-----------
ACCATCGCCAACGAGACATAGCACTGGGACATAGCGGGGCTCATACGAAGATCGTAGGGTAACCA
AATCGGTGCTGCTTTCTGGCAAACCATCTCGAGCGAGCACGGCCTCGACAGCAATGGCGTGTACGT
ATTTGAGTCGTCAACATTACCGCGAAGAACTCCAACTAATCACCAATAAACAGCTACAACGGCACC
TCCGAGCTCCAGCTCGAGCGCATGAGCGTCTACTTCAACGAGGTACGGATTCGCGAAAGCA-----
GAAATAGATGGAACTAGTTACTGATCGTATCACCGCATGCAGGCCTCTGGCAACAAGTATGTTCCT
CGCGCCGTCCTCGTCGATCTCGAGCCCGGCACCATGGACGCCGTCCGTGCCGGTCCCTTCGGTCAG
CTCTTCCGACCGGACAACTTCGTTTTCGGTCAGTCCGGTGCTGGCAACAACTGGGCCAAGGGTCAC
TACACTGAGGGTGCTGAGCTGGTTGACAACGTCCTCGACGTCGTTCGCCGTGAGGCCGAAGGCTGC
GACTGCCTCCAGGGCTTCCAGATCACCCACTCCCTCGGTGGTGGTACCGGTGCCGGTATGGGTACC
CTGTTGATCTCCAAGATCCGCGAGGAGTTCCCCGACCGCATGATGGCTACCTTCTCCGTCATGCCTT
CCCCCAAGGTCTCCGACACCGTCGTCGAGCCCTACAACGCCACTCTCTCCGTCCACCAGCTGGTCG
AGAACTCTGACGAGACGTTCTGTATCGACAATGAGGCTCTGTACGATATCTGCATGCGTACGCTGA
AGTTGTCCAACCCCTCGTACGGCGATCTGAACCACCTGGTCTCCGCCGTCATGTCCGGCGTCACCAC
TTGTCTGCGTTTCCCCGGTCAGTTGAACTCTGACCTGCGCAAGCTCGCCGTGAACATGGTTCCTTTC
CCTCGTCTCCACTTCTTCATGGTCGGCTTCGCCCCTCTGACCAGCCGCGGCGCTCACTCTTTCCGCG
CCGTCACCGTTCCCGAGTTGACTCAGCAGATGTTCGACCCCAAGAACATGATGGCTGCCTCCGATT
TCCGCAACGGTCGCTACCTAACATGCTCGGCCATCTTGTAAGATATCCTCCAGAATTG--
TCGTTCGTATCAGCCTGCTAACCTAAGTTTCGTAGCCGTGGCAAGGTCTCCA

>Hypoxylon_zhaotongensis_GMBCC1168

-CATTACTGAGTTCTACAAAACTCCAACCCTATGTGAATCT--ACTCTGTTGCCTCGGCGCTGAGCGC-
-

CTATTCGGGTGCTACCCTGGAGATTCCTACCCTGTAGGAAGCTACCCTGAAGCTACCCTGTAGTTGC
ATTTTACGCTCCGCCGGTGGACCACTAAACTCTTGTATATACACTGTATCTCTGAGTGACTTAACTA
AATAAGTTAAAACTTTCAACAACGGATCTCTTGGTTCTAGCATCGATGAAGAACGCAGCGAAATGC
GATACGTAATGCGAATTGCAGAATTCAGTGAGTCATCGAATCTTTGAACGCATATTGCGCCCATTA
GTATTCTAGTGGGCATGCCTATTCGAGCGTCATTTCAACCCTTAAG-

CCCTGTAGCTTAGCGTTGGGAGTCTATTCTTTAGTGGATAGTTCCTTAAAGTTAGTGGCGGTGCTA-
GGTACACTCGTAGCGTAGTAATTATTCTCGCTCCTGTGGTGCCCCTAGCTACCTGCCGTAAAACTCT
A--------------AGACCCTAAA-------------------------------TGAAGCGGCAACAGCTCAAA-
TTTGAAATCTGGCC----CTA-----GTGGTCCGAGTTGTAATTTGTAGAGGATGCTTCTGGTTAGGT-
GCCTTCTGAGTTCCCTGGAACGGGACGCCAGAGAGGGTGAGAGCCCCGTACGGTTGGA-
CACCGAGCCTATATGTAGCTCCTTCGACGAGTCGAGTAGTTTGGGAATGCTGCTCTAAATGGGAGG
TAAATTTCTTCTAAAGCTAAATACCGGCTAGAGACC-
GATAGCGCACAAGTAGAGTGATCGAAAGATGAAAAGCACTTTGAAAAGAGGGTTAAATAGCACGT
GAAATTGTTGAAAGGGAAGCG-
TTTGCGACCAGACCTTCTCCAGGGGGATCATCCGGTATTTACTTACCGGTGCACTTCGCCTGG--
TGTAGGCCAGCATCGGTTCT---TGTAGGGGGAGAAAAGCCTGGGGAACGTAGCTC----
TCTAGAGAGTGTT-ATAGCCCCTAGCATAATACCCCTAC-GGGGACCGAGGACCGCGCT-TCG---
GCAAGGATGCTGGCATAATGGTCGTCAACGACCCGTCTTGAAACACGGACCAAGGAGTCGAACAT
TTGTGCGAGTGTTTGGGTA--TAAAACCCTCACGCGTAATGAAAGTGAACGGAGGTGAGAGCCCTT--
---TG---GGGTGCATCATCGACCGATCCTGATG-
TCTTCGGATGGATTTGAGTAAGAGCATAACTGTTCGGACCCGAAAGATGGTGAACTATGCGTGGAT
AGGGTGAAGCCAGAGGAAACTCTGGTGGAGGCTCGCAGCGGTTCTGACGTGCAAATCGATCGTCA
AATCTGCGCATGGGGGCGAAAG--

>Hypoxylon_hypomiltum

-
CATTACAGAGTTACTAAAACTCCCAACCCTTTGTGAACCTTACCGTCGTTGCCTCGGTGGAAGGTGT
GTGCGGTGGGAAGCTACCCTGGAGCTACCTACCCTGTAGATAGCTACCCTGGAGCTACCCTGAAAA
TACGCCCCGCCAGCCGCCGAAGGACCACTAAACTCTTGTTTTTACTGTGTCTCTCTGAATAATGAAA
CAAAATTCGTTAAAACTTTCAACAACGGATCTCTTGGTTCTGGCATCGATGAAGAACGCAGCGAAA
TGCGATAAGTAATGTGAATTGCAGAATTCAGTGAATCATCGAATCTTTGAACGCATATTGCGCCCA
TTAGTATTCTAGTGGGCATGCCTATTCGAGCGTCATTTCAACCCTTAAGCACCTTCTGCTTAGCGTT
GGGAGTCTGCGCCTC--GGCGCAGTTCCCTAAAGGTAGTGGCAGTGTTA-
GGTACACTCGTAGCGTAGTAATTCTTCTCGCGTCTGTAGTGGTCCTGAACGCTAGCCGTTAAACCCC
C----
TTTCTCAAGGTTGACCTCGGATTAGGTAGGAATACCCGCTGAACTTAAGCATATGAAGCGGCAACA
GCTCAAA-TTTGAAATCTGGCC----CTC-----
GTGGTCCGAATTGTAATTTGTAGAGGATGCTTTTGGTGTGGT-
GCCTTCTGAGTTCCCTGGAACGGGACGCCAGAGAGGGTGAGAGCCCCGTACGGTTGGA-
CACCTATCCTATATATAGCTCCTTCGACGAGTCGAGTAGTTTGGGAATGCTGCTCTAAATGGGAGG
TAAATTTCTTCTAAAGCTAAATACCGGCCAGAGACC-

GATAGCGCACAAGTAGAGTGATCGAAAGATGAAAAGCACTTTGAAAAGAGGGTTAAATAGCACGT
GAAATTGTTGAAAGGGAAGCG-TTTGCGACCAGACCTTTTCCTGGCGGATCATCCGGTGTTC--
TCACCGGTGCACTTCGCTTGG--TTTAGGCCAGCATCGGTTTT---
CTTAGGGGGATAAAGGCCTGGGGCACGTAGCTC----TTCCGGGAGTGTT-
ATAGCCCCTAGCGTAATGCCCTTAC-GGGGACCGAGGACCGCGCT-TCG---
GCAAGGATGCTGGCGTAATGGTCGTCAACGACCCGTCTTGAAACACGGACCAAGGAGTCGAACAT
TTGTGCGAGTGTTTGGGTG--TTAAACCCTCACGCGTAATGAAAGTGAACGGAGGTGAGAGCCCTT--
---CG---GGGTGCATCATCGACCGATCCTGATG-
TCTTCGGATGGATTTGAGTAAGAGCATAACTGTTCGGACCCGAAAGATGGTGAACTATGCGTGGAT
AGGGTGAAGCCAGAGGAAACTCTGGTGGAGGCTCGCAGCGGTTCTGACGTGCAAATCGATCGTCA
AATCTGCGCATGGGGGCGAAAGACTTATCGA-
ACCATCTAGTAGCTGGTTACCGCCGAAGTTTCCCTCAGGATAGCAGTGT--TG-
TTTTCAGTTTTATGAGGTAAAGCGAATGATTAGGGACTCGGGGGCTCTTTTTTGCCTTCATCCATTC
TCAAACTTTAAATATGTAAGAAGCCCTTGTTACTTAATTGAACGTGGGCATTCGAATGTATCAACA
CTAGTGGGCCATTTTTGGTAAGCAGAACTGGCGATGCGGGATGAACCGAACGCGGGGTTAAGGTG
CCAGAGTGGACGCTCATCAGACACCACAAAAGGTGTTAGTACATCCAGACAGC-
GGACGGTGGCCATGGAAGTCGGAATCCGCTAAGGACTGTGTAACAACTCACCTGCCGAATGTACTA
GCCCTGAAAATGGATGGCGCTCAAGCGTCT-
CACCCATACCTCGCCCTTAAGGTAGAAACGATGCCTTAAGGGCGCTGTATAGAATCCAATAAACAT
TTCGAGATCGCTCTAGCCGCTAAACCAGCTATCATCACGAACGGTCTCAAATACTCACTCGCTACG
GGAAACTGGGGCGACCAGAAGAAGGCCATGAGCTCGACAGCTGGTGTATCACAGGTCTTGAATCG
ATACACGT------TTGCTTCCA---
CACTTTCTCACTTGAGAAGAACCAACACACCCATTGGGCGTGACGGCAAGCTTGCGAAACCTCGAC
AGCTTCACAATACGCATTGGGGCCTAGTCTGTCCAGCAGAAACGCCTGAAGGCCAGGCCTGCGGTT
TGGTGAAGAACCTGTCTCTGATGTGTTCCGTCAGTGTGGGGACAGCAACGGATCCTATAGTAGACT
ATATGATCACTAGGAATATGGAAGTTCTTGAGGAGTACGAACCCATGCGATACCCTGACGCAACCA
AGATTTTCCTCAATGGTTCCTGGATCGGTGTCCATCAGAACCCCAAGGCTCTAGTAAAGGACGTTC
AGCATCTGCGCCGAACGAATCAGATCCCGGCCGAGGTATCGCTAGTTCGCGACATACGTGATCGCG
AGTTCAAGATCTTTTCAGATGCTGGTCGAGTCATGCGTCCTTTATTTGTTGTAAACCAAGAGGAT----
----
ACAGAAGCCGGCGCCAAAAAGGGTACCTTGGCTCTTACCAAGGATATGATTCAGAGGCTCGAGGC
AGATGTCGATCTTGATCCTGAGAGTGAGGAGTACTTTGGCTGGCAGGGCTTGGTCAGTGAAGGTGT
CATCGACTACCTGGACGCCGAAGAAGAAGAAACAGCCATGATATGCATGACACCCGAGGACCTTG
AGACCTACCGACAAGCTAAACTCGGATACGATGTATCTCAGGACAATGGA------
GACGAGGTTAATAAACGACTGAAGACCAAGGTTAATCCGACAACGCACATGTACACGCACTGCGA
GATCCATCCCAGCATGCTTCTCGGTATTTGTGCCAGTATTATCCCATTTCCAGATCATAACCAAGTA
CCCCTGAACGCGTTCCCCAACCCCCTGATCTTTTGCCCCTCACCCACAAACACAAACATCACGATG
GATCGAGGTGTCGTGTCTCGCA---------GTTTTTA-TCTA---
AATCAGAAAATGGAAGCTAATCATATCTCTTCGTCTTAATAGGTTCACCTTCAGACCGGCCAATGC
GTAAGAACTACCTATATTTCCAACT---GATTA-
TCAACGCGAAGGAATATTGCGGGGCTCACATGGATTTTTTAGGGTAACCAAATTGGTGCTGCTTTC
TGGCAAACCATCTCTGGCGAGCACGGTCTCGACAGCAATGGCGTGTATGTGTTT-
ATTATTCAATTCTATAACGAAAGTTAAAAGCTAATGGTTGGCCAATAGTTACAACGGAACCTCTGA
GCTCCAGCTGGAGCGCATGAGCGTCTACTTCAACGAGGTAAGCTACGATAGAAGCTACAAATACGT
GTCCAAATCTAGGCACTAATCCT----
GCTTGCATAGGCTTCCGGTAACAAGTATGTTCCCCGTGCCGTTCTCGTCGATCTCGAGCCCGGTACC
ATGGATGCCGTCCGTGCTGGTCCCTTCGGTCAGCTCTTCCGACCCGACAACTTCGTCTTTGGTCAGT
CCGGTGCTGGCAACAACTGGGCCAAGGGTCACTACACTGAAGGTGCTGAGCTTGTTGACAATGTTT
TGGATGTCGTTCGTCGTGAGGCTGAGGGCTGTGACTGCCTTCAGGGTTTCCAGATCACCCACTCTCT
CGGTGGTGGTACTGGTGCCGGTATGGGTACCCTGTTGATCTCCAAGATCCGCGAAGAGTTCCCTGA
CCGCATGATGGCTACCTTCTCCGTTGTTCCTTCTCCCAAGGTTTCGGATACCGTTGTTGAGCCTTAC
AACGCCACTCTCTCCGTCCACCAGCTGGTCGAGAACTCAGACGAGACCTTCTGCATTGACAACGAA
GCTCTATACGACATCTGCATGCGTACCCTGAAGCTATCCAACCCCTCGTACGGTGACCTGAACCAC
CTGGTTTCCGCTGTCATGTCTGGTGTCACTACCTGCTTGCGTTTCCCCGGTCAGCTAAACTCTGACCT
CCGCAAGCTGGCTGTCAACATGGTGCCTTTCCCTCGTCTGCACTTCTTCATGGTCGGCTTCGCGCCC
CTGACCAGCCGTGGTGCTTACTCTTTCCGTGCCGTCACCGTTCCTGAGTTGACTCAGCAGATGTTCG
ATCCCAAGAACATGATGGCTGCTTCTGACTTCCGTAACGGTCGTTACCTGACATGCTCTGCTATCTT

GTGAGATAACCCTTCCTAATTATTGACTAATATTTGTTTACTAACGTA--
AATTCTAGCCGTGGCAAGATCTCCA

>Hypoxylon_damuense_FCATAS4207

-CATTACAGAGTTAC-TAAACTCCCAACCCTTTGTGAACCATACCGTCGTTGCCTCGGCGGAAGGC--
GGTTTCGTTGGAGCTACCCTGGAGACACCTACCCTGTAGGTGGCTACCCTGGAGCCGCCCTGAAAA
TATGC-
CCGCCAGCCGCCGGAGGACTATCAAACTCTTGTTTATACAACGTATCTCTGAATTAAAGAACAAAA
TTCGTTAAAACTTTCAACAACGGATCTCTTGGTTCTGGCATCGATGAAGAACGCAGCGAAATGCGA
TAAGTAATGTGAATTGCAGAATTCAGTGAACCATCGAATCTTTGAACGCATATTGCGCCCATTAGT
ATTCTAGTGGGCATGCCTATTCGAGCGTCATTTCAACCCTTAAGCAACTTAAGCTTAGCGTTGGGAG
TCTGCGTCTTACGGCGCAGTTCCCTAAAGGCAGTGGCAGTGTTA-
GGTGCACTCGTAGCGTAGTAATTTTTCTCGCTTCTGCAGTGGCCTGGCGTACTCGCCGTTAAAACCC
CCAT-CTAAAAAAGGTTGACCTCGGATTAG---------------------------TGAAGCGGCAACAGCTCAAA-
TTTGAAATCTGGCC----CTC-----GTGGTCCGAGTTGTAATTTGTAGAGGATGCTTTTGGTGCGGT-
GCCTTCCGAGTTCCCTGGAACGGGACGCCAGAGAGGGTGAGAGCCCCGTACGGTTGGA-
CACCTACCCTATATATAGCTCCTTCGACGAGTCGAGTAGTTTGGGAATGCTGCTCTAAATGGGAGG
TAAATTTCTTCTAAAGCTAAATACCGGCCAGAGACC-
GATAGCGCACAAGTAGAGTGATCGAAAGATGAAAAGCACTTTGAAAAGAGGGTTAAATAGCACGT
GAAATTGTTGAAAGGGAAGCG-TTTGCGACCAGACCTTCTCCTGGCGGATCATCCGGTGTTC--
TCACCGGTGCACTTCGCCTGG--TTTAGGCCAGCATCGGTTTC---
TGTAGGGGGATAAAGGCCTGGGGCACGTATCTC----CTCCGGGAGTGTT-
ATAGCCCCTAGCGTAATGCCCTTAC-GGGGACCGAGGACCGCGCT-TCG---
GCAAGGATGCTGGCGTAATGGTCGTCAACGACCCGTCTTGAAACACGGACCAAGGAGTCGAACAT
TTGTGCGAGTGTTTGGGTG--TTAAACCCTCACGCGTAATGAAAGTGAACGGAGGTGAGAGCCCTT--
---CG---GGGTGCATCATCGACCGATCCTGATG-
TCTTCGGATGGATTTGAGTAAGAGCATAACTGTTCGGACCCGAAAGATGGTGAACTATGCGTGGAT
AGGGTGAAGCCAGAGGAAACTCTGGTGGAGGCTCGCAGCGGTTCTGACGTGCAAATCGATCGTCA
AATCTGCGCATGGGGGCGAAAGACTTATCGA-ACCATCTAGT--

ACGCTGTATCGAATCCAACAAACACTTCGAGATTGCCTTGGCTGCCAAACCCACCATCATCACGAA
CGGTCTCAAGTACTCGCTCGCCACAGGAAACTGGGGCGACCAAAAGAAGGCAATGAGTTCTACAG
CTGGTGTGTCTCAGGTCTTGAACCGATACACGT------TCGCTTCGA---
CGCTTTCTCACTTGAGAAGAACCAACACGCCTATTGGGCGTGACGGTAAGCTTGCGAAGCCCCGAC
AGCTTCACAATACTCATTGGGGTCTGGTCTGCCCAGCCGAAACGCCCGAGGGCCAGGCGTGCGGTT
TGGTCAAAAACTTGTCACTGATGTGTTCAATCAGTGTGGGCACTTCTACAGACCCTATAGTTGATTA
TATGATCACTAGGAATATGGAGGTCCTTGAGGAGTACGAACCCTTGCGGTACCCCGACGCTACTAA
GATCTTTCTTAACGGCTCTTGGATTGGCGTGCACCAGAACCCTAAGGCTTTGGTGAGAGACGTCCA
GAATCTGCGTCGAACGAATCAGATCCCGGCCGAGGTCTCGTTGATCCGCGACATTCGTGATCGTGA
ATTCAAGATCTTTTCGGACGCTGGCCGAGTCATGCGCCCCTTGTTTGTTGTAAACCAAGAGGAC------
----
ACGGAAAGGGGCGCCAAGAAAGGAACGCTAGCCCTCACCAAGGAAATGATCCAGAGGCTCGAGG
CAGATGTCGAACTGGATCCCGAAGGTGATGAGTACTTTGGCTGGCAAGGTTTGGTTAACGAAGGTG
TCATCGACTACCTCGATGCCGAGGAAGAAGAAACCGCGATGATATGCATGACGCCCGAGGACCTT
GACACTTACCGCCAGGCCAAGCTCGGATACGAGGTGTCCCAGGATAATGGG------
GACGAGATTAATAAACGACTGCGGACCAAGGTTAACCCGACAACTCATATGTACACCCACTGTGA
GATCCATCCTAGCATGCTTCTAGGTATTTGCGCGAGCATTATCCCGTTCCCGGATCATAACCA----
CCCCTGAACGCGTCCCCCAACCCCCTGATCTCCTACTCTTCACCCACGATCAGAAACACCACAGCA
G-TCGAGATACCACGTCTCGCA--------G--TTGTC--------
CTACAGAAAATGGAAGCTAATTATATCTCTTCGTCTTAATAGGTTCACCTTCAGACCGGCCAATGC
GTAAGAATTGCCTATATCTCCAACC---AGCTA-
TCCACGCGAAAAAAGATTGCGGGGCTCACATGGATTTTGTAGGGTAACCAAATTGGTGCTGCTTTC
TGGCAGACCATCTCTGGCGAGCACGGCCTCGACAGCAATGGCGTGTACGTGTTCTATTGGTCAATT
CCGTGTCG--
AGTCCAAATCTAATAGTTGATCAATAGCTACAACGGAACCTCTGAGCTCCAGCTGGAGCGCATGAG

CGTTTACTTCAACGAGGTACGCCATAACGAAGAC-
ATAAATACGTGCGCGAGTCCAGTTACTAATTATCCCTACGTGCGCAGGCTTCTGGCAACAAGTACG
TGCCCCGTGCCGTTCTCGTCGATCTCGAGCCCGGTACCATGGACGCCGTCCGTGCTGGTCCTTTCGG
TCAGCTCTTCCGACCCGACAACTTCGTCTTTGGTCAGTCCGGTGCTGGCAACAACTGGGCCAAGGG
TCATTACACTGAAGGTGCTGAGCTCGTCGACAACGTCTTGGATGTCGTTCGTCGTGAGGCTGAGGG
CTGCGACTGCCTTCAGGGTTTCCAGATCACCCACTCTCTCGGTGGTGGTACCGGTGCCGGTATGGGT
ACCTTGTTGATCTCCAAGATCCGCGAGGAGTTCCCTGACCGCATGATGGCTACTTTCTCCGTCGTTC
CTTCCCCCAAGGTCTCGGATACCGTTGTCGAGCCTTACAACGCCACACTCTCCGTCCACCAGCTGGT
CGAGAACTCGGACGAGACTTTCTGCATTGACAACGAGGCTCTGTACGACATCTGCATGCGTACTCT
GAAGCTATCCAACCCATCGTACGGTGACCTAAACCACCTCGTCTCTGCCGTCATGTCTGGCGTCACC
ACCTGCTTGCGATTCCCCGGTCAGCTGAACTCTGATCTCCGCAAGTTGGCTGTCAACATGGTGCCCT
TCCCTCGTCTGCACTTCTTCATGGTCGGCTTCGCCCCCCTGACCAGCCGTGGTGCTTACTCGTTCCGT
GCTGTCACCGTTCCCGAGTTGACTCAGCAGATGTTCGACCCCAAGAACATGATGGCTGCTTCCGAT
TTCCGCAACGGTCGCTACCTGACGTGCTCTGCCATCTTGTAAGAACCCTGCTCTAAGCTGTTGATTT
GAATTTATTTGCTAACCTA--AATTCTAGCCGTGGTAAGATCTCCA

>Hypoxylon_canariense_MUCL47224

-
CATTACTGAGTTCTTAAAAACTCCTACCCTTTGTGAACCTTACCGTCGTTGCCTCGGCGCCGAGCGC
ACCTACCCTGGAGCTACCCTGTAGAACTCTACCCTGGAGAACCCTACCCTGGAGCTACCCTGTAGT
TGCAA-
TCACGCTCCGCCGGTGGACCTCTACACTCTGTTTTGTATAGTGTATCTCGGAAAACCATAACTTAAT
ACGTTAAAACTTTCAACAACGGATCTCTTGGTTCTGGCATCGATGAAGAACGCAGCGAAATGCGAT
AAGTAATGTGAATTGCAGAATTCAGTGAATCATCGAATCTTTGAACGCATATTGCGCCCATTAGTA
TTCTAGTGGGCATGCCATTTCGAGCGTCATTTCGACCCTTAAG-
CCCTGCTGCTTAGCGTTGGGAACCTACGGGGCACGGTGTAGCTCCCTAAAGGTAGTGGCGGTGTTA-
GGTACACTCGTAGCGTAGTAAATTTTCTCGCTCCTGCGGTGTACCTGAGG-
CCTGCCGTGAAAACCCCTATATTTACATGGTTTGACCTCGGATTAGGTAGGAATACCCGCTGAACTT
AAGCATATGAAGCGGCAACAGCTCAAA-TTTGAAATCTGGCC----CTC-----
GTGGTCCGAATTGTAATTTGTAGAGGATGCTTTGGGCGCGGT-
GCCTTCCGAGTTCCCTGGAACGGGACGCCAGAGAGGGTGAGAGCCCCGTACGGTCGGATCACCTA
GCCTATCTATAGCTCCTTCGACGAGTCGGGTAGTTTGGGAATGCTGCTCTAAATGGGAGGTAAATT
TCTTCTAAAGCTAAATACCGGCCAGAGACC-
GATAGCGCACAAGTAGAGTGATCGAAAGATGAAAAGCACTTTGAAAAGAGGGTTAAATAGCACGT
GAAATTGTTGAAAGGGAAGCG-TTTACGACCAGACCTCTTCCAGGCGGATCATCCGGTGTTC--
TCACCGGTGCACTTCGCCTGG--TTCAGGCCAGCATCGGTTTT---
CGTAGGGGGAGAAAGGCCTGGGGAACGTATCTC----CTTAGGGAGTGTT-
ATAGCCCCTTGCGTAATACCCTTAC-GGGGACCGAGGACCGCGCT-CCG---
GCAAGGATGCTGGCGTAATGGTCGTCAACGACCCGTCTTGAAACACGGACCAAGGAGTCGAACAT
TTGTGCGAGTGTTTGGGTA--GTAAACCCTCACGCGTAATGAAAGTGAACGGAGGTGAGAGCCCCT--
---CG---GGGTGCATCATCGACCGATCCTGATG-
TCTTCGGATGGATTTGAGTAAGAGCATAACTGTTCGGACCCGAAAGATGGTGAACTATGCGTGGAT
AGGGTGAAGCCAGAGGAAACTCTGGTGGAGGCTCGCAGCGGTTCTGACGTGCAAATCGATCGTCA
AATCTGCGCATGGGGGCGAAAGACTTATCGA-
ACCATCTAGTAGCTGGTTACCGCCGAAGTTTCCCTCAGGATAGCAGTGT--TG-
TTTTCAGTTTTATGAGGTAAAGCGAATGATTAGGGACTCGGGGGCGCTATATTGCCTTCATCCATTC
TCAAACTTTAAATATGTAAGAAGCCCTTGTTACTTTATTGAACGTGGGCATTCGAATGTATCAACAC
TAGTGGGCCATTTTTGGTAAGCAGAACTGGCGATGCGGGATGAACCGAACGTGGGGTTAAGGTGC
CAGAGTGGACGCTCATCAGACACCACAAAAGGTGTTAGTACATCTAGACAGT-
GGACGGTGGCCATGGAAGTCGGAATCCGCTAAGGACTGTGTAACAACTCACCAACCGAATGTACT
AGCCCTGAAAATGGATGGCGCTCAAGCGTCT-
CACCCATACCTCACCCTTAGGGTAGAAACGATGCCCTAAGGGCGATGCATCGACACCAACAAGCAT
TTCGAAATCGCCCTAGCCGCCAAGCCGGCCATTATTACGAACGGCCTCAAGTACTCGCTCGCTACA
GGAAACTGGGGCGATCAGAAGAAGGCCATGAGTTCGACGGCAGGTGTATCGCAGGTCTTGAACCG
ATATACGT------TCGCCTCGA---
CGCTTTCTCATTTGAGAAGAACCAACACGCCCATCGGCCGAGACGGAAAGCTAGCGAAGCCCCGTC
AGCTTCACAATACCCATTGGGGCTTGGTCTGTCCGGCCGAAACGCCCGAGGGTCAGGCCTGCGGTC

TCGTGAAGAACTTATCATTGATGTGTTCAATCAGTGTCGGTACGTCTACTGACCCTATCGTCGACTA
TATGATCACTAGGAACATGGAAGTTCTTGAGGAGTACGAACCGCTGCGATATCCAGATGCCACCAA
GATCTTCCTCAACGGTTCTTGGATCGGTGTCCATCAGAACCCCAAGGCTCTAGTGAGAGATGTCCA
GAATCTGCGCCGGACTAATCAGATCCCGGCCGAGGTGTCGTTGGTCCGCGACATTCGCGATCGTGA
ATTCAAGATCTTTTCGGATGCCGGTCGGGTCATGCGCCCCTTGTTTGTAGTACAGCAAGAGGAT------
----

AGCGAGGCTGGCGTTAAGAAGGGAACGCTAGCTCTTACCAAGGAGATGATCCAGAGGCTCGAGGC
GGATGTCGATACCGATCCCGATAGCCATGAGTATTTTGGCTGGCAAGGTCTGGTCAACGAAGGTGT
CATCGATTACCTTGACGCCGAGGAAGAAGAGACGGCGATGATATGTATGACGCCCGAGGACCTCG
AGACCTACCGCCAGACCAAGGCCGGTTACGTCGTATCCCAGGATAACGGG------
GACGAGATTAATAAACGGCTAAGGACCAAGGTGAACCCGACCACGCACATGTACACCCACTGCGA
AATTCATCCTAGCATGCTCCTAGGTATTTGCGCCAGTATCATTCCTTTCCCAGATCATAATCAGGTA
CCCCTGAACGCGTCCCCCAACCCCCTGATTTCCTACCCCTCACGCACAAACACCACAAGCACGAAA
CGTTTCGATTCCCTATATCGCGA---ACCGAAATCAC--CATG---
TCTTGGAATATGAAAGCTAACCATATATCTTGATTTGAATAGGTTCACCTTCAGACCGGCCAATGC
GTAAGAACTACCTACTCCAACAACCGCCAATCA-
TCGACGCGAAAGAACATAGCGGGGCTCACATA--
TTTATTAGGGTAACCAAATTGGTGCTGCTTTCTGGCAGACCATCTCTGGCGAGCACGGTCTCGACA
GCAATGGCGTGTACGTATTTGGCTGGGCAATTTTTCAACGAGAGTACAAAATTAATAGTTTGTCAA
CAGCTACAACGGAACCTCTGAGCTCCAGCTGGAGCGCATGAGCGTTTACTTCAACGAGGTAGGTCA
TAATCAGGACCAGGAATGCATGCGCAAGAGTCGTTACTAATTACTTTCATACGCGCAGGCCTCTGG
AAACAAGTATGTGCCCCGTGCCGTTCTCGTCGATCTCGAGCCCGGTACCATGGATGCCGTCCGTGC
TGGTCCCTTTGGTCAGCTCTTCCGACCCGACAACTTCGTCTTCGGTCAGTCCGGTGCTGGCAACAAC
TGGGCCAAGGGTCACTACACCGAGGGTGCCGAGCTCGTTGACCAGGTTTTGGATGTCGTTCGTCGC
GAGGCCGAGGGCTGCGACTGCCTTCAAGGTTTCCAGATCACCCACTCGCTCGGTGGTGGTACCGGT
GCCGGTATGGGTACCTTGTTGATCTCCAAGATCCGCGAGGAGTTTCCCGACCGCATGATGGCCACT
TTCTCCGTCGTGCCCTCCCCCAAGGTCTCCGACACCGTCGTCGAGCCTTACAACGCCACCCTCTCCG
TCCACCAGCTGGTCGAGAACTCGGACGAGACCTTCTGCATTGACAATGAGGCTCTGTACGACATCT
GCATGCGTACCCTCAAGCTGTCCAACCCCTCGTATGGTGACCTGAACCACCTGGTCTCGGCCGTCAT
GTCTGGTGTCACCACCTGCCTGCGATTCCCTGGTCAGCTGAACTCGGATCTCCGCAAGTTAGCCGTC
AACATGGTGCCCTTCCCTCGTCTGCACTTCTTCATGGTCGGCTTTGCTCCCCTGACCAGCCGCGGTG
CTTACTCTTTCCGTGCCGTCACCGTTCCCGAATTGACCCAGCAGATGTTCGACCCCAAGAACATGAT
GGCTGCTTCCGACTTCCGTAACGGTCGTTACCTGACGTGCTCTGCCATCTTGTAAGATACCCGTTCC
GAGTCGTCGATAAGYGTTTATATGCTAACCCT--AATTCTAGCCGTGGCAAGATCTCCA

>Hypoxylon_lenormandii

-CATTACAGAGTTAC-TAAACTCCCAACCCTATGTGAA-CCTACCACTATTGCTTCGGCG---
GGCGCTGCTACCCTGTAGCCACCCTGTAATT-C-----------------AGGGTGGGCTTACCCGGTAGTT-
TGCGTCAAGGCCCGTCAGAGGACCACTAAACTCTTGTACCTGTAC-
GTATTTCTGAATGCTTCAACTTAATAAGTTAAAACTTTCAACAACGGATCTCTTGGTTCTGGCATCG
ATGAAGAACGCAGCGAAATGCGATAAGTAATGTGAATTGCAGAATTCAGTGAATCATCGAATCTTT
GAACGCACATTGCGCCCATTAGTATTCTAGTGGGCATGCCTATTCGAGCGTCATTTCAACCCTTAAG
-CCCTGTTGCTTAGCGTTGGGAA-CTGCGCCATACGGCG-
AGCTCCTTAAAGTTAGTGGCGGAGTTAGTGTATACTCTTAGCGTAGTAAATATCCTCGCTCACGTAG
TACGC---TGAAGCGGCAACAGCTCAAA-
TTTGAAATCTGGCC----CTA-----GCGGTCCGAGTTGTAATTTGTAGAGGATGCTTTTGGTGCGGC-
GCCTTCCGAGTTCCCTGGAACGGGACGCCGGAGAGGGTGAGAGCCCCGTACGGTTGGA-
CGCCTAGCCTCTATATAGCTCCTTCGACGAGTCGAGTAGTTTGGGAATGCTGCTCTAAATGGGAGG
TAAATTTCTTCTAAAGCTAAATACCGGCCAGAGACC-
GATAGCGCACAAGTAGAGTGATCGAAAGATGAAAAGCACTTTGAAAAGAGGGTTAAATAGCACGT
GAAATTGTTGAAAGGGAAGCG-TTTGCGACCAGACTTTTTCCGGGCGGATCATCCGGTGTTT--
AGGCCGGTGCACTCCGTCCGG--TTTAGGCCAGCATCGGTTCT---
CTTAGGGGGATAAAGGCTTAGGGCACGTAGCTC----TTTCGGGAGTGTT-
ATAGCCCTTCGCGTAATACCCTTCG-GGGGACCGAGGTACGCGCT-CT----
GCAAGGATGCTGGCGTAATGGTCGTCAACGACCCGTCTTGAAACACGGACCAAGGAGTCGAACAT
TTGTGCGAGTGTTTGGGTG--TTAAACCCTCACGCGTAATGAAAGTGAACGGAGGTGAGAGCCCTT--
---CG---GGGTGCATCATCGACCGATCCTGATG-

TCTTCGGATGGATTTGAGTAAGAGCATAACTGTTCGGACCCGAAAGATGGTGAACTATGCGTGGAT
AGGGTGAAGCCAGAGGAAACTCTGGTGGAGGCTCGCAGCGGTTCTGACGTGCAAATCGATCGTCA
AATCTGCGCATGGGGGCGAAAGACTTATCGA-
ACCATCTAGTAGCTGGTTACCGCCGAAGTTTCCCTCAGGATAGCAGTGT--TG-
TCTTCAGTTTTATGAGGTAAAGCGAATGATTAGGGACTCGGGGGCGCTATTTTGCCTTCATCCATTC
TCAAACTTTAAATATGTAAGAAGCCCTTGTTACTTAGTTGAACGTGGGCATTCGAATGTACCAACA
CTAGTGGGCCATTTTTGGTAAGCAGAACTGGCGATGCGGGATGAACCGAACGCGGGGTTAAGGTG
CCAGAGTGGACGCTCATCAGACCCCACAAAAGGTGTTAATACATCCTGACAGCAGGACGGTGGCC
ATGGAAGTCGGAATCCGCTAAGGACTGTGTAACAACTCCCCTGCCGAATGTATTAGCCCTGAAAAT
GGATGGCGCTCTAGCGTCT-CACCCATACCTCGCCCTTAGGGTAGAAACCATGCCCTAAGG-----------
----
----------ACACGT------TCGCATCGA---
CTCTTTCACATTTGAGAAGAACCAACACACCTATTGGAAGAGATGGTAAGCTGGCGAAACCTCGCC
AGCTACACAATACCCATTGGGGTCTGGTCTGCCCAGCCGAGACTCCCGAAGGTCAGGCCTGTGGTT
TGGTGAAGAATCTGTCATTGATGTGCTCGATCAGTGTGGGTACCGCGACCGACCCCATTATAGAAT
ACATGATTACTAGAAACATGGATGTTCTTGAGGAGTACGAGCCAATGCGCTATCCCAACGCAACCA
AAATCTTCCTCAACGGCTCCTGGATTGGTGTACACCAGGATCCAAAATCTCTTGTCAAGGATGTTCA
ACAGCTGCGTCGCGCCAACCAGATTCCATCCGAGGTTTCGCTCATCCGCGACATCCGTGATCGCGA
GTTCAAGATCTTTTCAGACGCGGGCCGTGTCATGCGACCATTATTTGTTGTAAACCAAGAAAAT-----
----
AATCCCGACAAGGGTGTGGAAAAGGGAACTCTGGTCCTTGACCAGGATATGCTCGGCAAACTCGA
GCGCGATGTTGAGCTGGATCCAGATAGCGATGAGTACTACGGGTGGCAGGGCCTTGTCAATCAGG
GCGTCATCGAGTATCTGGATGCGGAGGAAGAAGAAACCGCGATGATCTGTATGACACCCGAAGAC
TTGGAAAATTACCGGTTGATCAAGGCTGGATATGATATAGCTCAGGACAACGGT------
GACGAGATTAACAAGCGTCTCAAGACCAAGGTGAATCCTACGACACATATGTACACCCATTGCGA
GATCCATCCCAGTATGCTTCTGGGTATCTGCGCCAGTATCATTCCTTTCCCGGACCACAATCAAGTA
CCCCTGAACGCGTCTCTCAACCCCCTGATTTCCTGCCCCTCACGCACAAAAAAT--
CATCGCAACACCAAACAGCTATGTTTACTAAGCTGTTATCGAGCTGCAATACGATGAATTGAATTA
TTGAAGCTAACCATGTTTTTTCATCATTATAGGTTCATCTCCAGACCGGCCAGTGCGTAAGTAACCA
TGC----AACGATGTCCAACGAAATCTCATAGAAAAACATTGCGGGGCTCACACGAAGCT-
ATAGGGTAACCAAATCGGTGCTGCCTTCTGGCAAACCATCTCTGGCGAGCACGGCCTCGACAGCAA
TGGCGTGTATGTATTTCATGCGGCAATTC------
TCAAACGGCCAACTAACCACCAATAATTAGCTACAATGGAACTTCCGAGCTCCAGCTTGAGCGCAT
GAGCGTTTACTTCAACGAGGTATGCAGCGACGAAATCTTGGAATAGATGATGAAGATTGGTTGCTA
ATTATCACAACATATGTAGGCTTCTGGCAACAAGTATGTTCCTCGCGCCGTCCTCGTCGATCTCGAG
CCCGGTACCATGGATGCCGTCCGTGCTGGTCCCTTCGGTCAGCTTTTCCGACCCGACAACTTCGTCT
TCGGCCAGTCTGGTGCCGGCAACAACTGGGCCAAGGGTCATTACACTGAAGGTGCTGAGCTTGTTG
ACCAGGTCCTTGACGTCGTTCGTCGTGAAGCTGAGGGCTGCGACTGCCTCCAGGGTTTCCAGATCA
CTCACTCTCTCGGTGGTGGTACTGGTGCCGGTATGGGTACCCTGTTGATCTCCAAGATCCGTGAGGA
GTTCCCTGACCGCATGATGGCCACCTTCTCCGTTGTTCCCTCCCCCAAGGTCTCCGACACCGTCGTT
GAGCCTTACAACGCCACCCTCTCCGTCCACCAGCTGGTCGAGAACTCCGACGAGACCTTCTGCATT
GACAACGAGGCTCTGTACGATATCTGCATGCGTACTCTCAAGCTATCCAACCCCTCGTACGGTGAC
CTGAACTACCTGGTTTCCGCCGTCATGTCGGGTGTCACCACTTGCCTGCGTTTCCCCGGTCAGCTAA
ACTCTGACTTGCGCAAGCTTGCCGTCAACATGGTTCCCTTCCCTCGTCTGCACTTCTTCATGGTCGG
CTTCGCTCCCCTGACCAGCCGTGGTGCTTACTCTTTCCGTGCCGTCACTGTTCCTGAGTTGACTCAG
CAGATGTTCGACCCCAAGAACATGATGGCCGCCTCTGACTTCCGCAATGGTCGCTACCTAACATGC
TCTGCCATCTTGTAAGCTTACGTTTAA-----TTCGTCTTATACTCATTCGCTAACTCA--
ATCTATAGCCGTGGTAAGGTTTCCA

>Hypoxylon_mangrovei_MFLU180559

-CATTACAGAGTTACCAAAACTCCCAACCCTTTGTGTA-CCTACTACTATTGCTTCGGCG---GGCGC--
CTACCCTGCGGCTACCCTGTAACT-C----------------GGGGTGGGCTTGCCTGGTAGCT-
TGCGAGAAGGCCCGTCAGAGGACCATCAAACTCTTGTACCTGTACGTAATATCTGAATGCTTCAAC
TTAATAAGTTAAAACTTTCAACAACGGATCTCTTGGTTCTGGCATCGATGAAGAACGCAGCGAAAT
GCGATAAGTAATGTGAATTGCAGAATTCAGTGAATCATCGAATCTTTGAACGCACATTGCGCCCAT
TAGTATTCTAGTGGGCATGCCTATTCGAGCGTCATTTCAACCCTTAAG-
CCCCGTGGCTTAGCGTTGGGACCCTGCGGCTTACGGCGCAGGTCCTTAAATTTAGTGGCGGAGTTA

GTGCATACTCTTAGCGTAGTAAATCTTCTCGCTCACGTAGTACGCCTAGCTACCTGCCGTAAAACCC
CCCCTATTTTTAATGGTTGACCTCGGATTAGGTAGGAATACCCGCTGAACTTAAGCATATGAAGCG
GCAACAGCTCAAA-TTTGAAATCTGGCC----CTA-----
GCGGTCCGAGTTGTAATTTGTAGAGGATGCTTTTGGTGCGGC-
GCCTTCCGAGTTCCCTGGAACGGGACGCCGGAGAGGGTGAGAGCCCCGTACGGTTGGA-
CGCCTAGCCTCTGTATAGCTCCTTCGACGAGTCGAGTAGTTTGGGAATGCTGCTCTAAATGGGAGG
TAAATTTCTTCTAAAGCTAAATACCGGCCAGAGACC-
GATAGCGCACAAGTAGAGTGATCGAAAGATGAAAAGCACTTTGAAAAGAGGGTTAAATAGCACGT
GAAATTGTTGAAAGGGAAGCG-TTTGCGACCAGACTTTTTCCGGGCGGATCATCCGGTGTTT--
AGGCCGGTGCACTCCGTTCGG--TTTAGGCCAGCATCGGTTCT---
CTTAGGGGGATAAAGGCTTAGGGCACGTGGCTC----CTTCTGGAGTGTT-
ATAGCCCTTCGCGTAATACCCCTCG-GGGGACCGAGGTACGCGCT-CT----
GCAAGGATGCTGGCGTAATGGTCGTCAACGACCCGTCTTGAAACACGGACCAAGGAGTCGAACAT
TTGTGCGAGTGTTTGGGTG--TTAAACCCTCACGCGTAATGAAAGTGAACGGAGGTGAGAGCCCTT--
---CG---GGGTGCATCATCGACCGATCCTGATG-
TCTTCGGATGGATTTGAGTAAGAGCATAACTGTTCGGACCCGAAAGATGGTGAACTATGCGTGGAT
AGGGTGAAGCCAGAGGAAACTCTGGTGGAGGCTCGCAGCGGTTCTGACGTGCAAATCGATCGTCA
AATCTGCGCATGGGGGCGAAA--

CAATAAATAGCTACAATGGAACTTCCGAGCTCCAGCTTGAACGCATGAGCGTTTACTTCAACGAGG
TATGCAACGACGAAAATTGGGAACAGATGAGGAAGATTGGTTGCTAATCGTCCCAACACATGTAG
GCTTCTGGCAACAAGTATGTTCCTCGCGCCGTCCTCGTCGATCTCGAGCCCGGTACCATGGATGCCG
TCCGTGCTGGTCCCTTCGGTCAGCTTTTCCGACCCGACAATTTCGTCTTTGGCCAGTCTGGTGCCGG
CAACAACTGGGCCAAGGGTCACTACACTGAAGGTGCTGAGCTTGTTGACAACGTCCTCGACGTCGT
CCGTCGTGAAGCTGAAGGCTGCGACTGCCTCCAGGGTTTCCAGATCACCCACTCTCTCGGTGGTGG
TACTGGTGCCGGTATGGGTACCCTGTTGATCTCCAAGATCCGTGAGGAGTTCCCTGACCGCATGAT
GGCCACCTTCTCCGTCGTTCCCTCCCCCAAGGTCTCCGACACCGTCGTTGAGCCTTACAATGCCACC
CTCTCCGTCCACCAGCTGGTCGAGAACTCCGATGAGACCTTCTGCATTGACAACGAGGCTCTGTAC
GATATCTGCATGCGTACTCTTAAGCTATCCAACCCCTCCTACGGTGACCTGAACTACCTGGTCTCCG
CTGTCATGTCGGGTGTCACCACTTGCCTGCGTTTCCCCGGCCAGCTAAACTCTGACCTGCGCAAGCT
TGCCGTCAACATGGTTCCGTTCCCCCGTCTACACTTCTTCATGGTCGGCTTCGCTCCCTTGACCAGC
CGTGGTGCCTACTCTTTCCGTGCTGTCACTGTTCCTGAGTTGACTCAGCAGATGTTCGACCCCAAGA
ACATGATGGCCGCGTCCGACTTCCGCAACGGTCGTTACCTAACATGCTCTGCCATCTTGTAAGA------
----

>Hypoxylon_teeravasati_PUFD4

-----------------GGGAGGTCCACCCTTTGTGTA-CCTACTACTATTGCTTCGGCG---
GGCGCGGCTACCCTGCAGCTACCCTGTAACTAC-----------------
GGGGTGGGCTTGCCTGGTAGCTATGCGAGAAGGCCCGTCAGAGGACCATCAAACTCTTGTACCTGT
ACGTAATATCTGAATGCTTCAACTTAATAAGTTAAAACTTTCAACAACGGATCTCTTGGTTCTGGCA
TCGATGAAGAACGCAGCGAAATGCGATAAGTAATGTGAATTGCAGAATTCAGTGAATCATCGAAT
CTTTGAACGCACATTGCGCCCATTAGTATTCTAGTGGGCATGCCTATTCGAGCGTCATTTCGACCCT
TAAG-
CCCTATGGCTTAGCGTTGGGACCCTGCGGCTTACGGCGCAGGTCCTTAAATTTAGTGGCGGAGTTA

GTGCATACTCTTAGCGTAGTAAACCTTCTCGCTCACGTAGTACGCCTAGCTACCCGCCGTAAAACCC
CCCTTATTTTTAATGGTTGACCTCGGATTAGGTAGGAATACCCGCTGAACTTAAGCATAGGAAGCG
GCAACAGCTCAAA-TTTGAAATCTGGCC----CTA-----
GCGGTCCGAGTTGTAATTTGTAGAGGATGCTTTTGGTGCGGC-
GCCTTCCGAGTTCCCTGGAACGGGACGCCGGAGAGGGTGAGAGCCCCGTACGGTTGGA-
CGCCTAGCCTCTGTATAGCTCCTTCGACGAGTCGAGTAGTTTGGGAATGCTGCTCTAAATGGGAGG
TAAATTTCTTCTAAAGCTAAATACCGGCCAGAGACC-
GATAGCGCACAAGTAGAGTGATCGAAAGATGAAAAGCACTTTGAAAAGAGGGTTAAATAGCACGT
GAAATTGTTGAAAGGGAAGCG-TTTGCGACCAGACTTTTTCCGGGCGGATCATCCGGTGTTT--
AGGCCGGTGCACTCCGTTCGG--TTTAGGCCAGCATCGGTTCT---
CTTAGGGGGATAAAGGCTTAGGGCACGTGGCTC----CTTCTGGAGTGTT-
ATAGCCCTTCGCGTAATACCCTTCG-GGGGACCGAGGTACGCGCT-CT----
GCAAGGATGCTGGCGTAATGGTCGTCAACGACCCGTCTTGAAACACGGACCAAGGAGTCGAACAT
TTGTGCGAGTGTTTGGGTG--TTAAACCCTCACGCGTAATGAAAGTGAACGGAGGTGAGAGCCCTT--
---CG---GGGTGCATCATCGACCGATCCTGATG-
TCTTCGGATGGATTTGAGTAAGAGCATAACTGTTCGGACCCGAAAGATGGTGAACTATGCGTGGAT
AAGGTGAAGCCAGAGGAAACTCTGGTGGAGGCTCGCAGCGGTTCTGACGTGCAAATCGATCGTCA
AATCTGCACATGGTGGCGAAAGACTTATCGA-ACCAT--

ACGCTGCATCGATCAATCCAGACGTTTCCAGATCGAGCTTGCTGCCAAGCCAGGCATCATCACTAA
CGGTCTAAAGTACTCTCTTGCCACGGGTAACTGGGGTGACCAGAAGAAAGCGATGAGTTCTACTGC
TGGTGTGTCACAAGTGCTTAACCGCTACACAT------TCGCATCGA---
CTCTCTCACATTTGCGAAGAACTAACACACCCATCGGAAGAGATGGTAAGCTGGCGAAACCTCGCC
AACTGCACAATACCCATTGGGGTCTGGTTTGCCCAGCCGAAACGCCCGAAGGTCAGGCCTGTGGCT
TAGTGAAGAATCTGTCACTGATGTGCTCGATCAGTGTGGGCACAGCGACCGATCCCATTATTGAAT
ACATGATCACCAGAAACATGGATGTTCTCGAGGAGTACGAGCCAATGCGCTATCCCAACGCAACC
AAAATCTTCCTCAACGGCTCCTGGATTGGCGTACACCAGGATCCCAAATCTCTGGTTAAAGATGTT
CAGCAGCTACGTCGAGCCAACCAGATTCCGTCGGAGGTTTCGCTCGTTCGCGACATTCGTGATCGC
GAGTTCAAGATCTTTTCAGACGCGGGTCGTGTCATGCGACCCTTATTTGTCGTAAACCAAGAAGAT-
----
GATCCCGCGAATAAGGTGAAAAGGGGAACTTTGGCCCTTGACCAGGATATGCTCGGCAAACTCAA
GCGTGATCTTGATCTGGATCCGGATAGCGATGAGTACTTCGGATGGCAGGGCCTCGTCAATCAGGG
TGTCATTGAGTATCTAGACGCAGAAGAAGAAGAGACCGCAATGATCTGCATGACACCCGAAGACT
TGGAAAATTACCGGTTACTCAAGACTGGATATGACGTAGCCCAGGATAACGGA------
GACGAAATTAACAAGCGTCTCAAGACGAAGGTGAATCCTACGACACATATGTATACACACTGCGA
GATCCACCCCAGTATGCTTCTGGGTATCTGCGCCAGTATTATTCCTTTCCCCGACCACAATCAGGTA

----------------AGGTTCATCTCCAGACCGGCCAGTGCGTAAGTAAC----C----
AACGATAGCCGACCAAATCTCATGGAAAAACATTGCGGGGCTCACACGAAGCT-
ATAGGGTAACCAAATCGGTGCTGCCTTCTGGCAAACCATCTCTGGCGAGCACGGCCTCGACAGCAA
TGGCGTGTATGTATTTCATTCGGCAATTCTCA------
AACGACCAACTGACAACCAATAAATAGCTACAATGGAACTTCCGAGCTCCAGCTTGAACGCATGA
GCGTTTACTTCAACGAGGTATGCAACGACGAAAATTGGGAACAGATGAGGAAGATTGGTTGCTAA
TCGTCACAACACATGTAGGCTTCTGGCAACAAGTATGTACCTCGCGCCGTCCTCGTCGATCTCGAG
CCCGGTACCATGGATGCCGTCCGTGCTGGTCCCTTCGGTCAGCTTTTCCGACCCGACAATTTCGTCT
TCGGCCAGTCTGGTGCCGGCAACAACTGGGCCAAGGGTCACTACACTGAAGGTGCTGAGCTTGTTG
ACAACGTTCTCGACGTCGTCCGTCGTGAAGCTGAAGGCTGCGACTGCCTCCAGGGTTTCCAGATCA
CCCACTCTCTCGGTGGTGGTACTGGTGCCGGTATGGGTACCCTGTTGATCTCCAAGATCCGTGAGG
AGTTCCCTGACCGCATGATGGCCACCTTCTCCGTCGTTCCCTCCCCCAAGGTCTCCGACACCGTCGT
TGAGCCTTACAACGCCACCCTCTCCGTCCACCAGCTGGTCGAGAACTCCGATGAGACCTTCTGCATT
GACAACGAGGCTCTGTACGATATCTGCATGCGTACTCTTAAGCTATCCAACCCCTCGTACGGTGAC
CTGAACTACCTGGTCTCCGCTGTCATGTCGGGTGTCACCACTTGCCTGCGTTTCCCCGGCCAGCTAA
ACTTTGACCTGCGCAAGCTTGCCGTCAACATGGTTCCGTTCCCCCGTCTACACTTCTTCATGGTCGG
CTTCGCTCCCTTGACCAGCCGTGGTGCCTACTCTTTCCGTGCTGTCACTGTTCCTGAGT-------------------

----

>Hypoxylon_aurantium_MFLU161202

-CATTATAGAGTTGCAAAAATCCCAAACCCTTTGTGAA-CCTACCACTGTTGCCTTGGCGCG-
AGCGC--CTACCTTATAGCTGC-----------CTACCTGGCCCGTA---GC---GTAGCTATAGCGT-
AGTCCGCTGTACGGCCCGTCAGAGGACCTCTAAACTCTATTTTTTACTGTTATTATCTGAATGGATT
AACTAATAAATTAAAAACTTTCAACAACGGATCTCTTGGTTCTGGCATCGATGAAGAACGCAGCGA
AATGCGATAAGTAATGTGAATTGCAGAATTCAGTGAATCATCGAATCTTTGAACGCACATTGCGCC
CATTAGTATTCTAGTGGGCATGCCTATTCGAGCGTCATTCAACCCCTTAAGCCTTCGTTGCTTAGCG
TTGGGAATCTACGTCTTACGGCGTAGCTCCTTAAAATTAGTGGCGGAGTCGCAGCACACTCTGAGC
GTAGTAGTATTTCTCGCTTTTGCGGTGGCCGCGGTTTCTTGCCGTAAAAACCCCTAT-
TTTCTAGTGGTTGACCTCGGATTAGGTAGGAATACCCGCTGAACTTAAGCATATGAAGCGGCAACA
GCTCAAA-TTTGAAATCTGGCC----CTA-----
GCGGTCCGAGTTGTAATTTGTAGAGGATGCTTCTGGCGAGGC-
GCCTTCCGAGTTCCCTGGAACGGGACGCCGTAGAGGGTGAGAGCCCCGTACGGTCGGT-
TGCTAAGCCTATATGTAGCTCCTTCGACGAGTCGAGTAGTTTGGGAATGCTGCTCTAAATGGGAGG
TAAATTTCTTCTAAAGCTAAATACCGGCCAGAGACC-
GATAGCGCACAAGTAGAGTGATCGAAAGATGAAAAGCACTTTGAAAAGAGGGTTAAATAGCACGT
GAAATTGTTGAAAGGGAAGCG-TTTGCGACCAGACCTTTTCGTAGCGGATCATCCGGTGTTC-
TTCGCCGGTGCACTTCGCTACG--ATCAGGCCAGCATCGGTTTT---
CTCAGGGGGATAAAGGCCTGGGGAACGTAGCTC----TCTCGGGAGTGTT-
ATAGCCCCTCGCGTAATACCTTTTA-GGGGACCGAGGACCGCGCT-TCG---
GCAAGGATGCTGGCGTAATGGTCGTCAACGACCCGTCTTGAAACACGGACCAAGGAGTCGAACAT
TTATGCGAGTGTTTGGGTG--CTAAACCCTCACGCGTAATGAAAGTGAACGGAGGTGAGAGCCTT----
ATT----AGGTGCATCATCGACCGATCCTGATG-
TCTTCGGATGGATTTGAGTAAGAGCATAGCTGTTCGGACCCGAAAGATGGTGAACTATGCGTGGAT
AGGGTGAAGCCAGAGGAAACTCTGGTGGAGGCTCGCAGCGGTTCTGACGTGCAAATCGATCGYCA
AATCTGCGCATGGGGGCGAAA--

>Hypoxylon_lividipigmentum_STMA14045

GCATTACCGAGTTCT-AAACTCCCAAACCCTATGTGAA-CGTACCGCAGTTGCCTCGGCGTG-
AGCGCACTGCCCTGGTGGCCGCCCTC-----------------------GC---GCGGCCGCCTGGC-GGCGCAC-
TCAAGGCCCGCCGAAGGACCACTAAACTCTGTTTAATATAACGTTTCTCTGAATGCTTCAACTAAAT
AAGTTAAAACTTTCAACAACGGATCTCTTGGTTCTGGCATCGATGAAGAACGCAGCGAAATGCGAT
AAGTAATGTGAATTGCAGAATTCAGTGAATCATCGAATCTTTGAACGCACATTGCGCCCATTAGTA

TTCTAGTGGGCATGCCTATTCGAGCGTCATTTCAACCCTTAAG-
CCCTGTTGCTTAGCGTTGGGAATCTACCGGGTCCCGCGTAGTTCCCTAAAACCAGTGGCGGAGCCC-
AGGGTGCCGCGAGCGTAGTAATTTATCTCGCTTTTGCAGTTCCCTGTGTTGCCGGCCGTAAAACCCC
CCATA--
TTTTCTGGTTGACCTCGGATTAGGTAGGAATACCCGCTGAACTTAAGCATATGAAGCGGCAACAGC
TCAAA-TTTGAAATCTGGCC----CTC-----
GCGGTCCGAGTTGTAATTTGTAGAGGATGCTTTTGGTGCGGC-
GCCTTCCGAGTTCCCTGGAACGGGACGCCTTAGAGGGTGAGAGCCCCGTACGGTTGGA-
CGCCAATCCTATATATAGCTCCTTCAACGAGTCGAGTAGTTTGGGAATGCTGCTCTAAATGGGAGG
TAAATTTCTTCTAAAGCTAAATACCGGCCAGAGACC-
GATAGCGCACAAGTAGAGTGATCGAAAGATGAAAAGCACTTTGAAAAGAGGGTTAAATAGCACGT
GAAATTGTTGAAAGGGAAGCG-TTTGCGACCAGACCTTCTCCAGGCGGATCAACCAACGTTC--
TCGATGGTGCACTTCGCCTGG--TTTAGGCCAGCATCGGTTTT---
CCTAGGGGGATAAAGGCTTGGGGAACGTGGCTC----CCTCGGGAGTGTT-
ATAGCCCCTCGCGTAATACCCTTAG-GGGGACCGAGGACCGCGCT-TCG---
GCAAGGATGCTGGCGTAATGGTCGTCAACGACCCGTCTTGAAACACGGACCAAGGAGTCGAACAT
TTGTGCGAGTGTTTGGGTG--TCAAACCCTCACGCGTAATGAAAGTGAACGGAGGTGAGAGCCCTT--
--AC----GGGTGCATCATCGACCGATCCTGATG-
TCTTCGGATGGATTTGAGTAAGAGCATAACTGTTCGGACCCGAAAGATGGTGAACTATGCGTGGAT
AGGGTGAAGCCAGAGGAAACTCTGGTGGAGGCTCGCAGCGGTTCTGACGTGCAAATCGATCGTCA
AATCTGCGCATGGGGGCGAAAGACTTATCGA-
ACCATCTAGTAGCTGGTTACCGCCGAAGTTTCCCTCAGGATAGCAGTGT--TG-
TCTTCAGTTTTATGAGGTAAAGCGAATGATTAGGGACTCGGGGGCGCTATTTTGCCTTCATCCATTC
TCAAACTTTAAATATGTAAGAAGCCCTTGTTACTTAGTTGAACGTGGGCATTCGAATGTACCAACA
CTAGTGGGCCATTTTTGGTAAGCAGAACTGGCGATGCGGGATGAACCGAACGCGGGGTTAAGGTG
CCAGAGTGGACGCTCATCAGACACCACAAAAGGTGTTAGTACATCTTGACAGCAGGACGGTGGCC
ATGGAAGTCGGAATCCGCTAAGGACTGTGTAACAACTCACCTGCCGAATGTACTAGCCCTGAAAAT
GGATGGCGCTCAAGCGTCT-CACCCATACCTCGCCCTTAGGGTAGAAACGATGCCCTAAGG----------
----

CCCCTGAACGCGTCTTGCAAAATCCTTGATATCTGCCCCTCATGCATCCAATGTGCAACCACGCCAT
-
CCATCACTTCCCATGTCATTCTCTATTCAGCCTGCGTGCCATCAGATCAAGACATCAAAGCTAACCG
CGTCTCTTT-TCTCTATAGGTTCATCTTCAGACCGGCCAATGCGTAAGTGCC----------
ACGAATACCGACGAAACGCGGTACTGGGAGGCAGCGGGGCTCACATG--
GGTTGCAGGGTAACCAAATCGGTGCCGCTTTCTGGCAGACCATCTCCAGCGAGCACGGTCTCGACA
GCAATGGCGTGTATGT-
TCTGAGAAGTCAATCCCGGTACCAGGGATGTCGGCTAACGACCAATAAACAGCTACAACGGAACC
TCGGAGCTCCAACTCGAGCGCATGAGCGTCTACTTCAACGAGGTATGGATCTACRAAGCTGGAGTG
TGGACATGTAGAATGATTTGCTGATCGCCACGACCTACGCAGGCTTCCGGCAACAAGTATGTTCCT
CGCGCCGTTCTCGTCGATCTCGAGCCCGGCACCATGGATGCCGTCCGTGCTGGCCCCTTCGGCCAG
CTTTTCCGACCGGACAACTTCGTTTTCGGCCAGTCGGGTGCCGGCAACAACTGGGCCAAGGGTCAC
TACACTGAGGGTGCTGAGCTCGTCGACAATGTTCTCGACGTTGTCCGCCGCGAGGCCGAGGGCTGC
GACTGCCTCCAGGGCTTCCAGATCACCCACTCGCTCGGTGGTGGTACCGGTGCCGGTATGGGTACC
CTGCTGATCTCCAAGATCCGCGAAGAGTTCCCCGACCGCATGATGGCCACCTTCTCCGTCATGCCCT
CCCCCAAGGTCTCCGACACCGTCGTCGAGCCTTACAACGCCACCCTCTCTGTCCACCAGCTGGTCG
AGAACTCGGACGAGACCTTCTGCATTGACAACGAGGCTCTGTACGACATCTGCATGCGCACCCTGA
AGTTGTCCAACCCCTCGTACGGTGACCTGAACCACCTGGTCTCTGCCGTCATGTCCGGCGTCACAAC
CTGCCTGCGTTTCCCCGGTCAGCTGAACTCYGACCTGCGCAAGCTCGCCGTGAACATGGTTCCCTTC
CCTCGTCTCCACTTCTTCATGGTCGGCTTCGCTCCCCTGACCAGCCGTGGCGCCTACACCTTCCGAG
CCGTCACCGTTCCCGAGCTGACCCAGCAGATGTTCGACCCCAAGAACATGATGGCTGCCTCTGACT

TCCGCAACGGTCGCTACCTGACGTGCTCCGCCATCTTGTATGATACCCTCCTCTACTTTTCCGCTAT
GAATATGCTGCTGACCTAAAGTAATTAGCCGTGGCAAGGTCTCCG

>Hypoxylon_rickii

-CATTAAAGGGTCGCCTTACTGCCAAACCCTATGTGAA-CTTACCACTGTTGCCTCGGCGG--
CGCGCGGCTACCCTGGAGCTACCCTGTAGCCATTTACCCTGTAGATGGCTACCCTGTAGCTACCCTG
TACTCGCACGTAAAGGCCCGTCAAAGGACC-CTAAACTCTATTTT--
ACTTGGTATCTCTGAGTAAATCAACTTAATAAGTTAAAACTTTCAACAACGGATCTCTTGGTTCTGG
CATCGATGAAGAACGCAGCGAAATGCGATAAGTAATGTGAATTGCAGAATTCAGTGAATCATCGA
ATCTTTGAACGCACATTGCGCCCATTAGTATTCTAGTGGGCATGCCTATTCGAGCGTCATTTCAACC
CTTAAG-
CCCTGTTGCTTAGCGTTGGGGGCCTGCGCCTGGCGGCGTAGCCCCTGAAAGATAGTGGCGGAGTCA
GTGAGCACTCTGAGCGTAGTAACTTTTCTCGCTCCGGTAGTGCCCCTGGCTGCTGGCCGTTAAACCC
CCCATACTTCTAGTGGTTGACCTCGGATTAGGTAGGAATACCCGCTGAACTTAAGCATATGAAGCG
GCAACAGCTCAAA-TTTGAAATCTGGCC----CTA-----G-
GGCCCGAATTGTAATTTGTAGAGGATGCTTTGGGCGCGGT-
GCCTTCTGAGTTCCCTGGAACGGGACGCCAAAGAGGGTGAGAGCCCCGTACGGTTGGC-
CACCAAGCCTGTATATAGCTCCTTCGACGAGTCGAGTAGTTTGGGAATGCTGCTCTAAATGGGAGG
TAAATTTCTTCTAAAGCTAAATACCGGCCAGAGACC-
GATAGCGCACAAGTAGAGTGATCGAAAGATGAAAAGCACTTTGAAAAGAGGGTTAAATAGCACGT
GAAATTGTTGAAAGGGAAGCG-TTTGCGACCAGACCTTTTCCAGGCGGATCATCCGGGGTTT--
TCTCCGGTGCACTTCGCCTGG--TTTAGGCCAGCATCGGTTCT---
CTTAGGGGGATAAAGGCCTGGGGAACGTAGCTC----CCTCGGGAGTGTT-
ATAGCCCCTTGCGTAATACCCTTCG-GGGGACCGAGGAACGCGCT-CT----
GCAAGGATGCTGGCGTAATGGTCGTCAACGACCCGTCTTGAAACACGGACCAAGGAGTCGAACAT
TTGTGCGAGTGTTTGGGTG--TCAAACCCTCACGCGTAATGAAAGTGAACGGAGGTGAGAGCCCTT--
---CG---GGGTGCATCATCGACCGATCCTGATG-
TCTTCGGATGGATTTGAGTAAGAGCATAACTGTTCGGACCCGAAAGATGGTGAACTATGCGTGGAT
AGGGTGAAGCCAGAGGAAACTCTGGTGGAGGCTCGCAGCGGTTCTGACGTGCAAATCGATCGTCA
AATCTGCGCATGGGGGCGAAAGACTTATCGA-
ACCATCTAGTAGCTGGTTACCGCCGAAGTTTCCCTCAGGATAGCAGTGT--TG-
TCTTCAGTTTTATGAGGTAAAGCGAATGATTAGGGACTCGGGGGCGCTATATTGCCTTCATCCATTC
TCAAACTTTAAATATGTAAGAAGCCCTTGTTACTTAGTTGAACGTGGGCATTCGAATGTACCAACA
CTAGTGGGCCATTTTTGGTAAGCAGAACTGGCGATGCGGGATGAACCGAACGCGGGGTTAAGGTG
CCAGAGTGGACGCTCATCAGACACCACAAAAGGTGTTAATACATTATGACAGCCGGACTGTGGCC
ATGGAAGTCGGAATCAGCTAAGGACTGTGTAACAACTCACCGGCCGAATGTATTAGCCCTGAAAAT
GGATGGCGCTCAAGCGTCT-
CACCCATACCTCGCCCTTAGGGTAGAAACGATGCCCTAAGGACGTTGCATTGACCAGAATAGGCGG
TTCCAGATCGAGCTTGCCGCGAAGCCGGCCATCATCACCAACGGCCTCAAGTACTCGCTCGCCACA
GGCAACTGGGGCGATCAGAAGAAGGCCATGAGTTCGACCGCCGGTGTGTCCCAGGTGCTGAACCG
ATACACGT------TCGCATCGA---
CACTCTCGCATTTGCGACGAACCAACACCCCTATCGGACGTGACGGAAAGCTGGCGAAACCGCGA
CAGCTCCACAACACGCACTGGGGATTGGTGTGCCCGGCCGAGACGCCCGAAGGTCAGGCTTGTGG
GTTGGTGAAGAATCTGTCCTTGATGTGTTCTATCAGCGTTGGGACATCGACCGACCCCATCGTAGA
CTACATGATCACTAGGAGCATGGAAGTCTTGGAGGAGTACGAGCCGATGCGATACCCACACGCCA
CCAAGATCTTCCTGAACGGTTCCTGGATCGGGGTGCATCAAGACCCGAAATCGCTGGTCCGAGATG
TGCAGCAGCTCCGACGAGCTAACCAGATCCCATCCGAGGTGTCATTGCTCCACGATATACGGGATC
GTGAGTTCAAGATCTTCTCGGATGCTGGTCGGGTCATGCGACCCTTGTTCGTCGTGCAGCAAGACG
AA---GACCCCGAACGTCG---------
AGGAACGTTAGCACTTACCAAAGAGCATATTCAGCGATTGGAAACCGACAACGACTTGGATCCGG
ACAGTGAAGAATACTTTGGTTGGCAAGGTTTGGTGAACGAAGGTGTTATTGAATACCTAGATGCGG
AGGAAGAAGAAACGGCGATGATCTGCATGACTCCGGAAGACCTCGAGACGTTCCGCTTAACGAAG
GGCGGTTATGAAGTGTCCCAAGACAACGGT------
GATGAAATCAACAAGAGGCTTAAGACGAAGATCAACCCTACGACACACACGTACACGCATTGCGA
GATTCATCCCAGCATGTTGTTGGGTATTTGCGCCAGTATTATTCCTTTCCCAGACCACAACCAGGTA
--------------------------------------T----------------------------------
CAATAAGATGGGCTATCGCAAATCAAGGCAATAAC-

AACATAGGAATTGGAGCTAATCATGTCTGTTTACATCTCTAGGTTCACCTTCAGACCGGCCAGTGC
GTAAGTAAA---------CACTACGAACGATGGACGAATTCGAAGGAGTATGGCGGGGCTCACT--
AAGATGATAGGGTAACCAAATCGGTGCTGCTTTCTGGCAGACCATCTCCAGCGAGCACGGTCTCGA
CAGCAATGGCGTGTAGGTGCTCCATTCGTCAATTCTTGCTATAACATTGTGGGCTAACAGCCAATG
AACAGCTACAACGGGACCTCGGAGCTCCAGCTCGAGCGCATGAGCGTCTACTTTAACGAGGTACAC
AGTCAAAGAAACCAACCATGTTTGTTTAGGAGTGGTTACTAATCACCCCAACATGCACAGGCATCG
GGTAACAAGTACGTCCCCCGCGCCGTCCTCGTCGATCTCGAACCCGGTACCATGGACGCCGTCCGC
GCTGGCCCCTTCGGTCAGCTCTTCCGACCCGACAACTTCGTCTTCGGCCAATCCGGTGCCGGCAACA
ACTGGGCCAAGGGTCATTACACTGAGGGCGCTGAGCTCGTTGACAACGTTCTTGACGTCGTCCGCC
GCGAGGCTGAGGGCTGCGACTGCCTCCAGGGCTTCCAGATTACCCACTCTCTCGGTGGTGGTACCG
GTGCCGGTATGGGTACCCTGCTGATCTCCAAGATCCGCGAGGAGTTCCCTGACCGCATGATGTCTA
CCTACTCCGTCGTTCCCTCCCCCAAGGTCTCTGACACCGTCGTTGAGCCCTACAATGCCACCCTCTC
CGTCCACCAGCTGGTCGAGAACTCGGACGAGACCTTCTGCATTGACAACGAGGCCTTGTACGACAT
CTGCATGCGTACCCTCAAGCTATCCAACCCCTCGTACGGTGACCTGAACCACCTGGTCTCCGCCGTC
ATGTCCGGTGTTACCACCTGCTTGCGTTTCCCTGGCCAGCTGAACTCTGATCTGCGCAAGCTTGCCG
TCAACATGGTTCCTTTCCCTCGTCTGCACTTCTTCATGGTTGGATTTGCTCCTCTGACCAGCCGTGGT
GCCTACACCTTCCGTGCTGTCACCGTCCCCGAGTTGACCCAGCAGATGTTCGATCCCAAGAACATG
ATGGCCGCTTCTGACTTCCGTAACGGTCGCTACTTGACGTGCTCTGCCATCTTGTAAGATATTATCT
CCGTGATGTTTTTTCTGGTGGGCTTGCTAACTGTATATTCCTAGCCGTGGCAAGGTCTCGA

>Hypoxylon_baruense_UCH9545

-CATTACAGAGTATT-ACAACTCATAACCCTATGTGAACCTTACCACCGTTTCCTCGGCGCG-
TGCGCGGCTGCGTTGCGGCCGCTCC---------------------GTCGCCCT----
CCGCCCCGGACTCGCGCCTCAAGGCCCGCCGGCGGACCCCCAAACTCTTG-
TCATGCAGTGGAATTCTGAATGCTTAACTAAAATAAGTTAAAACTTTCAACAACGGATCTCTTGGTT
CTGGCATCGATGAAGAACGCAGCGAAATGCGATAAGTAATGTGAATTGCAGAATTCAGTGAATCA
TCGAATCTTTGAACGCACATTGCGCCCATTAGCATTCTAGTGGGCATGCCTATTCGAGCGTCATTTC
AACCCTTAAG-
CCTCGTCGCTTAGCGTTGGGGGCCTGCCGCGGAGCGCGCAGCCCCTGAAAACCAGTGGCGGAGTCG
GTGAGCACTCTGAGCGTAGTAAC--
CTCTCGTTCCTGTAGTGCTCCCGGCGGCCTGCCGTGAAACCCCCTATACTTCTAATGGTTGACCTCG
GATTAGGTAGGAATACCCGCTGAACTTAAGCATATGAAGCGGCAACAGCTCAAA-
TTTGAAATCTGGCC----CCC-----GCGGTCCGAGTTGTAATTTGTAGAGGAAGCTTTTGGCGCGGT-
GCCTTCTGAGTTCCCTGGAACGGGACGCCAGAGAGGGTGAGAGCCCCGTACGGTTGGC-
CACCAAGCCTCTATAAAGCTCCTTCGACGAGTCGAGTAGTTTGGGAATGCTGCTCTAAATGGGAGG
TAAATTTCTTCTAAAGCTAAATACCGGCCAGAGACC-
GATAGCGCACAAGTAGAGTGATCGAAAGATGAAAAGCACTTTGAAAAGAGGGTTAAATAGCACGT
GAAATTGTTGAAAGGGAAGCG-TTTGCGACCAGACCTTCTCCAGGCGGATCATCCGGCGTTC--
TCGCCGGTGCACTTCGTCTGG--TCTAGGCCAGCATCGGTTTT---
CCCAGGGGGATAAAGGCTTGGGGAACGTAGCTC----CCTCGGGAGTGTT-
ATAGCCCCTCGCGTAATGCCCTTGG-GGGGACCGAGGACCGCGCA-TT----
GCAAGGATGCTGGCGTAATGGTCGTCAACGACCCGTCTTGAAACACGGACCAAGGAGTCGAACAT
TTGTGCGAGTGTTTGGGTG--TTAAACCCTCACGCGTAATGAAAGTGAACGGAGGTGAGAGCCCTT--
---CG---GGGTGCATCATCGACCGATCCTGATG-
TCTTCGGATGGATTTGAGTAAGAGCATAACTGTTCGGACCCGAAAGATGGTGAACTATGCGTGGAT
AGGGTGAAGCCAGAGGAAACTCTGGTGGAGGCTCGCAGCGGTTCTGACGTGCAAATCGATCGTCA
AATCTGCGCATGGGGGCGAAAGACTTATCGA-
ACCATCTAGTAGCTGGTTACCGCCGAAGTTTCCCTCAGGATAGCAGTGT--TG-
TACTCAGTTTTATGAGGTAAAGCGAATGATTAGGGACTCGGGGGCGCTATTTAGCCTTCATCCATTC
TCAAACTTTAAATATGTAAGAAGCCCTTGTTACTTAGTTGAACGTGGGCATTCGAATGTATCAACA
CTAGTGGGCCATTTTTGGTAAGCAGAACTGGCGATGCGGGATGAACCGAACGCGGGGTTAAGGTG
CCAGAGTGGACGCTCATCAGACACCACAAAAGGTGTTAGTACATCTAGACAGCAGGACGGTGGCC
ATGGAAGTCGGAATCCGCTAAGGACTGTGTAACAACTCACCTGCCGAATGTACTAGCCCTGAAAAT
GGATGGCGCTCAAGCGTCT-CACCCATACCTCGCCCTTAGGGTAGAAACGATGCCCTAAGG----------
----

CCCCTGAACGCGAATTACAATCCCCCTGATTTTCACCCCTCACGCACAAAAACCGCAAGCACAGCA
TCTCATAGTCATTGCTATGAGACTATCGTCAAATCCCTACACAACATATGGAGAAGTGGAAGCTAA
TATCATTTTTTTATCTTAATAGGTTCATCTTCAGACCGGCCAATGCGTAAGTAGTAGCCAGCTACAC
CATATCCAACAA-ACAACGAAAGAGAATATGGT--
GGTACTAACTAAGATCGTAGGGTAACCAAATTGGTGCTGCTTTCTGGCAAACCATCTCTAGCGAGC
ACGGTCTTGACAGCAATGGAGTGTATGTATTTCTATTCGCCATCCTTTTGATGAATTCGGTAACTAA
CAGCCAATCAATAGTTACAATGGAACTTCTGAGCTCCAGCTCGAGCGTATGAGCGTCTACTTTAAC
GAGGTACACAGTCTTAGAAACCCCAATTAGAAGAGCAAGAATCGTTACTAATCACCCTAACATGCA
CAGGCATCTGGTAACAAGTATGTCCCCCGCGCCGTCCTCGTCGATCTCGAACCCGGTACCATGGAT
GCCGTCCGCGCTGGTCCTTTCGGTCAACTCTTCCGTCCCGACAACTTCGTCTTCGGCCAATCCGGTG
CCGGAAACAACTGGGCCAAGGGTCATTACACTGAAGGTGCTGAGCTTGTCGACAACGTTCTCGATG
TCGTTCGTCGTGAGGCTGAGGGCTGTGACTGCCTCCAGGGTTTCCAAATTACCCACTCTCTTGGTGG
TGGTACCGGTGCTGGTATGGGTACCTTGTTGATCTCCAAGATCCGTGAAGAGTTCCCTGACCGTATG
ATGTCCACTTACTCCGTCGTTCCTTCTCCCAAGGTCTCCGATACTGTTGTTGAGCCTTACAATGCTAC
TCTCTCCGTTCACCAGCTGGTCGAGAACTCCGACGAGACCTTCTGTATTGATAACGAGGCCTTGTAC
GATATTTGCATGCGCACTCTCAAGCTATCTAACCCTTCTTATGGTGACCTGAACCACCTTGTCTCCG
CCGTCATGTCTGGCGTTACCACTTGCTTGCGTTTCCCTGGTCAGCTGAACTCTGATCTACGCAAGCT
TGCCGTCAACATGGTTCCTTTCCCTCGTCTCCATTTCTTCATGGTTGGCTTCGCTCCTCTAACCAGCC
GTGGTGCCTATACCTTCCGTGCTGTCACCGTTCCCGAGTTGACTCAGCAAATGTTCGACCCCAAGAA
CATGATGGCTGCTTCTGACTTCCGTAATGGTCGTTACCTGACGTGCTCTGCCATCTTGTAAGATATC
CCTTTCCAACTACTTTTTTGTATATATCTGCTAACTCCCTTTTTTTAGCCGTGGTAAGGTCTCCA

>Hypoxylon_ferrugineum_CBS141259

-CATTACAGAGTTACTAAACTCCAAAACCCTATGTGAACCTTACTATAGTTTCCTCGGCGTC--
GCGCGGCTACCCTGTAGCTACCCTGTAGAGACCTCC----------
TCTACCCGGCAGCTACCCTGTACCCGCGC-
TGAAGGCCCGCCGCTGGACCACTAAACTCTTGTTTTTACAGTGTATCTCTGAATGCTTCAACAAAAT
AAATTAAAACTTTCAACAACGGATCTCTTGGTTCTGGCATCGATGAAGAACGCAGCGAAATGCGAT
AAGTAATGTGAATTGCAGAATTCAGTGAATCATCGAATCTTTGAACGCACATTGCGCCCACTAGTA
TTCTGGTGGGCATGCCTATTCGAGCGTCATTTCAACCCTTAAGCCCCTGTTGCTTAGCGTTGGGAAT
CTACGTCCTAGGGCGTAGTTCCTTAAAATTAGTGGCGGAGTCGGTGCATACTCTAGGCGTAGTAAC
TTTACCAGCCTCTGTAGTGTACCTGGCTTCCGGCCGTTAAAGCCCCTATATTTTCAATGGTTGACCT
CGGATTAGGTAGGAATACCCGCTGAACTTAAGCATA--

---------------CCCCTGAACGCGTTCCTCAACCCCCTGATTTCGTGCCCCTCACGCACAAAACAT---
ACAGCAAAATGCACAAGCTGCTCACGTCGAACTGGGATGGAATTACACAACGAGA-

TGATGGAAATGTATGCTAATCATATCTTTTT--
TCATATAGGTTCACCTCCAGACCGGCCAGTGCGTAAGTACA-CATC----
CACGACCACCGATATAATACGGCGATAAGACATGGCGGGGCTCACACAATATTCGTAGGGTAACC
AAATCGGTGCTGCTTTCTGGCAGACCATCTCTGGCGAGCACGGCCTCGACAGCAATGGCGTGTACG
TATATTATTCGCCAATGCTCGATTTAAATCTGTCATCTAACGGCGAAATAACAGTTACAACGGCACT
TCCGAGCTCCAGCTCGAGCGCATGAGCGTCTACTTCAACGAGGTATGCACTTACAAGATAAAGAAA
AAAATAAGTCGCGATGGTTGCTAATCATCACAAAACATGCAGGCTTCCGGCAACAAGTATGTTCCC
CGCGCTGTCCTCGTCGATCTCGAACCCGGCACCATGGATGCTGTCCGTTCTGGTCCCTTCGGTCAGC
TCTTCCGACCCGACAACTTCGTCTTCGGCCAGTCTGGTGCCGGAAACAACTGGGCCAAGGGTCACT
ACACTGAGGGTGCTGAGCTCGTCGACCAAGTTCTCGATGTCGTTCGTCGCGAGGCTGAGGGCTGCG
ACTGCCTTCAGGGCTTCCAGATCACCCACTCCCTCGGTGGTGGTACCGGTGCCGGTATGGGTACTCT
CCTGATCTCCAAGATCCGCGAGGAGTTCCCCGACCGCATGATGGCTACCTTCTCCGTCGTTCCCTCT
CCCAAGGTCTCCGACACCGTCGTTGAGCCTTACAACGCCACCCTCTCCGTCCACCAGCTGGTCGAG
AACTCCGACGAGACCTTCTGCATTGACAACGAGGCTCTGTACGACATCTGCATGCGTACCCTCAAG
CTATCCAACCCCTCGTACGGTGACCTGAACCACCTTGTCTCTGCCGTCATGTCCGGTGTTACCACCT
GCCTGCGCTTCCCCGGTCAGCTCAACTCTGACCTGCGCAAGCTTGCCGTCAACATGGTTCCCTTCCC
TCGTCTGCACTTCTTCATGGTCGGATTCGCTCCCCTGACCAGCCGTGGTGCTCACTCCTTCCGCGCT
GTCACTGTTCCCGAGTTGACTCAGCAGATGTTCGACCCCAAGAACATGATGGCTGCTTCTGACTTCC
GCAACGGTCGCTACCTGACCTGCTCTGCCATCTTGTAAGATACCTTTATACGTCGCTTTGCTCGTCT
TCGTTTACTAACTCATG-TTTCTAGCCGTGGCAAGGTCTCCA

>Hypoxylon_cyclobalanopsidis_FCATAS2714

-CATTACTGAGTTATCATAAACTCCAACCCTATGTGAACCATACTCTTGTTGCCTTGGCGTG-
CGCGGGGCTGTCGGGTAGCTATCCTGGAGGT--CTACCCGGTAGGA-
CCTACCCGCTAGCTACCCGGG-AGCCTGCCATATGGCCCGCCAGAGGACCACCAAACTCTGC-
TTCTACCGTGGGACTCTGAATTAGCA-
ACTGAAATCGTTAAAACTTTCAACAACGGATCTCTTGGTTCTGGCATCGATGAAGAACGCAGCGAA
ATGCGATAAGTAATGTGAATTGCAGAATTCAGTGAATCATCGAATCTTTGAACGCACATTGCGCCC
ATTAGTATTCTAGTGGGCATGCCTATTCGAGCGTCATTTCAACCCTGAAGCCCTCGTCGCTTCGCGT
TGGGGATCTACAGGACACCCTGTAGGCCCCGAAAAGGAGTGGCAGAGTTCGGGCATACTCTCAGC
GTAGTAAACTTCCTCGCTCTTGCAGTA-
TCCCGTCCCCTCGCCGTAAAACCCCCCCTATTCTTCTAAGGTGACCTCGGATTAGGTAGAATGCCCC
AAT-------------TGAAGCGGCAACAGCTCAAA-TTTGAAATCTGGCC----CTC-----G-
GGTCCGAGTTGTAATTTGTAGAGGATGCTTTGGGTGCGGT-
GCCTTCCGAGTTCCCTGGAACGGGACGCCAGAGAGGGTGAGAGCCCCGTACGGTTGGA-
CACCTACCCTGTAGATAGCTCCTTCGACGAGTCGAGTAGTTTGGGAATGCTGCTCTAAATGGGAGG
TAAATTTCTTCTAAAGCTAAATACCGGCCAGAGACC-
GATAGCGCACAAGTAGAGTGATCGAAAGATGAAAAGCACTTTGAAAAGAGGGTTAAATAGCACGT
GAAATTGTTGAAAGGGAAGCG-TTTGCGACCAGACCTTCTCCAGGCGGATCATCTGGTGTTC--
TCACCGGTGCACTTCGCCTGG--TCTAGGCCAGCATCGGTTTC---
TCTAGGGGGGTAAAGGCGTGGGGAACGTAGCTC----TTTCGGGAGTGTT-
ATAGCCCTTCGCGTAATACCTTTCG-AGGGACCGAGGACCGCGCT-CT----
GCAAGGATGCTGGCGTAATGGTCGTCAACGACCCGTCTTGAAACACGGACCAAGGAGTCGAACAT
TTGTGCGAGTGTTTGGGTG--TCAAACCCTCACGCGTAATGAAAGTGAACGGAGGTGAGAGCCCTT--
--AC----GGGTGCATCATCGACCGATCCTGATG-
TCTTCGGATGGATTTGAGTAAGAGCATAACTGTTCGGACCCGAAAGATGGTGAACTATGCGTGGAT
AGGGTGAAGCCAGAGGAAACTCTGGTGGAGGCTCGCAGCGGTTCTGACGTGCAAATCGATCGTCA
AATCTGCGCATGGGGGCGAAAG----------ACCAT---

ACGCTGCATCGAAAATAATAAGCACTTCGAGATCGCTCTAGCAGCCAAACCAGCGATTGTGACCAA
CGGTCTCAAGTACTCACTCGCCACAGGCAACTGGGGTGATCAGAAGAAGGCGGCGAGTTCGACGG
CCGGTGTGTCACAAGTCCTGAACCGATACACGT------TCGCTTCCA---
CTCTTTCTCATTTGAGACGAACGAACACTCCTATCGGAAGAGATGGAAAGCTCGCGAAACCCCGCC
AGCTTCACAACACGCACTGGGGTCTAGTCTGCCCGGCCGAGACGCCCGAGGGACAGGCCTGTGGTT
TGGTCAAGAATCTGTCGCTGATGTGCTCTATCAGTGTGGGCACGTCCACTGATCCAATCGTCGATTA

CATGATCACGAGAAACATGGAAGTGCTCGAAGAGTATGAACCTTTGCGCTACCCCGATGCTACCAA
GATCTTCTTAAACGGCTCCTGGATTGGCGTACACCAGGACCCCCAGGCGCTTGTGAAAGATGTGCA
GCGTTTACGCCGCTCCGGCCAGATTCCTCCTGAAGTGTCGCTAGTTCGAGACATTCGCGATCGCGA
GTTCAAGATCTTCTCCGATGCTGGCCGTGTTATGCGTCCCTTGTTTGTTGTTCAGCAAGAGGGTGAT
AAGCCGTCGTCGTCGTCATCTGACGACAGTGAGGATGAAGAAGAGAAGGAAGAGGATGAAGCTGA
CAAAGTCAAGGGAACTTTGGCGCTCACCAAGGAGATGATCCAGAGGCTGGAAGCAGATAACGACC
TCGATCCCGATAGCGAAGAGTACTTCGGTTGGCAAGGTTTGGTCGGCGCTGGTGTCATCGACTACC
TAGACGCTGAGGAAGAGGAGACGGCCATGATCTGCATGACACCCGAAGATCTGGAGATCTACCGT
CGAACCAAGGCCGGTGACGAAGTCGACCAGGATAACGGT------
GACGAGATTAACAAGCGCCTGAAGACGAAGATCAACCCGACCACCCACATGTACACGCACTGTGA
GATCCATCCTAGCATGCTGCTAGGTATCTGCGCGAGCATCATTCCG---------------------
CCCCTGAACGCGTTCCTCAACCCCCTGATGCTATGCCCCTCACGCACAAACAATTCGAC-
ACATCGC----AAACTACTCAAGTTGTTATAT-------TTGGATATTA---
ATTTTGAATATTAAAACTAACCG------
TTTTTCTCGATAGGTTCACCTTCAGACCGGCCAATGCGTAAGAATGATAT------------CCCGACGA-
CCAGCGCGGAGAAAGATGGCGGGGCTCACG--
AAGATCATAGGGTAACCAAATCGGTGCTGCTTTCTGGCAAACCATCTCTGGCGAGCACGGCCTCGA
CAGCAATGGCGTGTATGTTTTTCATTGGCCAATCCCAAGAC-
AAATATAGAAAATAACTGACAAACAACAGCTACAATGGCACCTCGGAACTCCAGCTGGAGCGCAT
GAGCGTCTACTTCAACGAGGTACGCTATCTTGAAAATGCTTCCCTCGCGCAA-
AGAACCGTTACTAATCACCCCAACATGCACAGGCGTCTGGTAACAAGTACGTGCCTCGTGCCGTCC
TCGTCGACCTCGAGCCCGGCACCATGGACGCCGTCCGTGCTGGCCCCTTTGGTCAGCTCTTCCGACC
CGACAACTTCGTCTTTGGTCAGTCCGGTGCCGGAAACAACTGGGCCAAGGGTCACTACACCGAGGG
TGCCGAGCTGGTCGACCAGGTTCTCGACGTCGTCCGTCGTGAGGCCGAGGGCTGCGACTGTCTCCA
GGGCTTCCAGATCACCCACTCCCTCGGTGGTGGTACCGGTGCCGGTATGGGAACCTTGTTGATCTCC
AAGATCCGCGAGGAGTTCCCCGACCGCATGATGGCTACCTTCTCCGTTGTGCCCTCTCCCAAGGTCT
CTGACACCGTTGTCGAGCCTTACAACGCCACCCTCTCGGTCCACCAGCTGGTCGAGAACTCGGACG
AGACCTTCTGCATCGACAACGAGGCTCTCTACGACATCTGCATGCGTACCCTCAAGCTGTCCAACC
CCTCGTACGGCGACCTGAACCACCTGGTCTCCGCCGTCATGTCGGGCGTCACCACCTGCCTGCGATT
CCCCGGCCAGCTGAACTCGGACCTGCGCAAGCTCGCCGTCAACATGGTTCCCTTCCCGCGTCTGCA
CTTCTTCATGGTCGGCTTCGCCCCCCTGACCAGCCGCGGTGCTCACTCCTTCCGCGCCGTCACCGTG
CCCGAGTTGACCCAGCAGATGTTCGACCCCAAGAACATGATGGCTGCCTCTGACTTCCGCAACGGC
CGCTACCTGACGTGCTCTGCCATCTTGTGAGTGCTCATATATCACTATAACCTCTACGTAGCATTGC
TAATAAAGACATCACAGCCGTGGCAAGGTCTCCA

>Hypoxylon_hainanense_FCATAS2712

-CATTACCGAGTTGTCGAAACTCCAAACCCATTGTGAACCTTACCG-
CGTTGCCTCGGCGGTGAGCGCGGGCACCTGGGAGCTACCCTGTAGAA--
CTACCCTGAAGCTAGCTACCCTAGAGCTACCCTGGAGCTG----
CGCAAGCCCGCCGGAGGACCACTAAACTCTTG-
TTCGCTGCAGAATTTCTGAATCATAAAACCAAATACGTTAAAACTTTCAACAACGGATCTCTTGGTT
CTGGCATCGATGAAGAACGCAGCGAAATGCGATAAGTAATGTGAATTGCAGAATTCAGTGAATCA
TCGAATCTTTGAACGCACATTGCGCCCATTAGTATTCTAGTGGGCATGCCTATTCGAGCGTCATTTC
GACCCTTAAGCCCCTGTTGCTTAGCGTTGGGAGTCTACGGCT-
ACGGCGTAGTTCCCGAAAATGAGTGGCGGAGTTAGGGCGTACTCTAAGCGTAGTAAAACCTCTCGC
TTCTGCAGTATCCCTAGCTACCTGCCGTAAAACCCCT----TCTTTAGTGG-
TGACCTCGGATAGAAGGAAAAACCCCAT--

---------------------------------TCCGGCGTTC--TAGCCGGTGCACTTGACCCGG--
GGTAGCCCAGCATCGGTTCC----TTAGGGGGATAAAGGATCTGGGAACGTGGCTC----
CTTCGGGAGTGTT-ATA-CCCTCAGCGTAAT-CCCTTAG-GGGGACCGAGGACCGCGCT-TCG---
GCAAGGATGCTGGCATAATGGTCGTCAACGACCCGTCTTGAAACACGGACCAAGGAGTCGAACAT
TTGTGCGAGTGTTTGGGTG--TCAAACCCTCACGCGTAATGAAAGTGAACGGAGGCGAGAGCCCT----
-A-----GGGCGCATCGTCGACCGATCCTGATG-
TCTTCGGATGGATTTGAGTAAGAGCATAACTGTTCGGACCCGAAAGATGGTGAACTATGCGTGGAT
AGGGTGAAGCCAGAGGAAACTCTGGTGGAGGCTCGCAGCGGTTCTGACGTGCAAATCGATCGTCA

AATCTGCGCATGGGGGCGAAAGACTTATCGA-A---

ACGCTGCATCGACCAGAATCGACGTTTCCAGATCGAGTTGGCTGCCAAGCCTGCCATCATTACCAA
CGGCCTCAAGTACTCTCTCGCCACGGGCAACTGGGGCGACCAGAAGAAGGCGATGAGCTCGACCG
CCGGCGTGTCGCAGGTCCTGAACCGATACACGT------TTGCGTCGA---
CTCTCTCCCATCTGAGACGGACGAACACGCCCATCGGAAGGGACGGAAAACTTGCGAAGCCCCGG
CAGCTGCACAATACCCATTGGGGCCTGGTCTGTCCGGCCGAGACGCCCGAAGGTCAGGCTTGTGGG
CTGGTGAAGAATTTGTCGCTGATGTGTTCCATCAGCGTGGGTACGTCGACGGATCCCATCGTAGAC
TACATGATCACTAGGAATATGGAAGTCTTGGAGGAATACGAACCCATGCGGTACCCCAACGCCACC
AAGATCTTTCTCAACGGTTCTTGGATCGGTGTGCACCAAGATCCCAAATCCCTGGTTAGAGATGTTC
AGCAGCTCCGTCGGGCTAACCAGATCCCCTCTGAGGTGTCCCTGGTTCGCGACATCCGCGATCGCG
AGTTCAAGATCTTCTCGGACGCCGGCCGTGTTATGCGGCCCTTATTTGTCGTGCATCAGGAGGAT----
----
AACCCGGACGCCGGCACTACGAAGGGCTCGCTGACACTCAACAAGGAGATGATCCAGAGGTTGGA
GGCGGACGTCGACCTGGATCCCGAAAGCGACGAATACTTTGGCTGGCAAGGCCTGGTTAACGAGG
GCGCTATCGAATACCTCGACGCAGAAGAGGAGGAGACGGCCATGATCTGCATGACCCCCGAAGAC
CTAGAGAACTACCGCTTAACCAAGCTTGGACAAGATATGCCCCAGGACAACGGA------
GACGAGATTAACAAGCGTCTCAAAACGAAGGTCAACCCCACGACGCATATGTACACGCATTGCGA
GATCCATCCCAGCATGCTCTTAGGCATCTGCGCGAGCATCATCCCCTTCCCGGATCACAATCAGGT
ACCCCTGAACGCGTTCTCCAACCCCCCTGATTCCTGCCCCTCATGCACACAATGTGCCACC---ACAT-
TCATCACATTCTTTAATTCGTTGTTATCAGACTGCATAGCAGCACGAACAATACCAGAAGCTAACA
TTGCCTTTTTCTCTCGATAGGTTCACCTCCAGACCGGCCAATGCGTAAGTGCCACGAA----------
ACCAATTCAACATGGTACTGGAACATAGCGGGGCTTACACGAGGATTATAGGGTAACCAAATTGGT
GCTGCCTTCTGGCAAACCATCTCCAGCGAGCACGGCCTCGACAGCAACGGCGTGTACGTATCTGAG
TCGTCGATTACAACATTGAGAATCCTGACTGACAATCAATAAATAGCTACAATGGAACCTCGGAGC
TCCAGCTCGAGCGCATGAGCGTTTACTTCAACGAGGTAGGGCTACGTAGAAATGAAGAACGATAG
TTTAGGATCTGTTGCTAATCAACCCGACCCACGCAGGCCTCTGGTAACAAGTATGTTCCTCGCGCTG
TCCTCGTCGATCTCGAGCCCGGCACCATGGACGCCGTCCGTGCTGGTCCCTTCGGCCAGCTCTTCCG
ACCCGACAACTTCGTTTTTGGCCAGTCTGGCGCCGGAAACAACTGGGCCAAGGGTCACTACACCGA
GGGTGCTGAGCTCGTCGACAACGTCCTTGATGTTGTTCGTCGCGAAGCTGAGGGCTGCGACTGCCT
TCAGGGTTTCCAGATCACTCACTCTCTCGGTGGTGGTACCGGTGCCGGTATGGGTACTCTTCTGATC
TCCAAGATCCGCGAAGAGTTCCCCGACCGCATGATGGCTACTTTCTCCGTCATGCCCTCCCCTAAGG
TCTCTGACACCGTCGTCGAGCCCTACAACGCCACCCTCTCCGTCCACCAGCTGGTCGAGAACTCGG
ACGAGACCTTCTGCATTGACAACGAGGCTCTCTACGACATCTGCATGCGCACCCTGAAGTTGTCCA
ACCCTTCGTACGGTGACCTGAACCACTTGGTCTCTGCCGTCATGTCCGGCGTCACCACTTGCCTGCG
TTTCCCCGGTCAGCTAAACTCTGATCTGCGCAAGCTCGCCGTGAACATGGTTCCCTTCCCTCGTCTT
CACTTTTTCATGGTTGGCTTCGCTCCCCTGACGAGCCGTGGCGCCTACACCTTCCGTGCCGTCACCG
TCCCTGAGTTGACTCAGCAGATGTTCGACCCCAAGAACATGATGGCTGCTTCCGATTTCCGCAACG
GTCGCTACCTGACGTGCTCTGCCATCTTGTATGATACCCCTTATATTATCATTTCTGTTACAGATTTG
CTAACTTGAGTTTCCTAGCCGTGGCAAGGTCTCCG

>Hypoxylon_carneum

-CATTACCGAGTTCTCGAGACTCCCAACCCTGTGTGAACGTTACCGCCGTTGCCTCGGCG---------------
-------------G---CGCCCCGCGCGCCGCCGGAGGACC--
CAAACTCTCTTCTGCGGCGTGCATCTCTGAACGCGTA-
ACCGAATCAGTTAAAACTTTCAACAACGGATCTCTTGGTTCTGGCATCGATGAAGAACGCAGCGAA
ATGCGATACGTAATGCGAATTGCAGAATTTAGTGAGCCATCGAATCTTTGAACGCACATTGCGCCC
GCCAGCATTCTGGCGGGCATGCCTATCCGAGCGTCATTTCAACCCTTAAGCCCCTGCCGCTTAGCGT
TGGGACCCTGCGGCGCGCGCCGCAGCTCCCCAAAGGTAGTGGCGGTGCCG-
GGTGCACTCGTAGCGCAGTAAGCACTCTCGCTCACGAGGCGCCCCCGGCCACCCGCCGTAAAACGC
CCCGTGCCTCTCGCGGTTGACCTCGGATTAGGTAGGGATACCCGCTGAACTTAAGCATA---------------
------TTTGAAATCTGGCC----CTG-----
GCGGCCCGAGTTGTAATTTGCAGAGGATGCTTTGGGCGCGGC-
GCCTTCCGAGTTCCCTGGAACGGGACGCCACAGAGGGTGAGAGCCCCGTACGGTGGGA-
CGCCTACCCCGCGTATAGCTCCTTCGACGAGTCGAGTAGTTTGGGAATGCTGCTCTAAACGGGAGG

TAAATTTCTTCTAAGGCTAAATACCGGCCAGAGACC-
GATAGCGCACAAGTAGAGTGATCGAAAGATGAAAAGCACTTTGAAAAGAGGGTTAAACAGCACGT
GAAATTGTTGAAAGGGAAGCG-TCCGCGACCAGACCTTCTCCAGGCGGATCCTACGGCGTTC--
TCGACGTGGCCCTCCGCCTGG--TCTAGGCCAGCATCGGTCCC---
CGCGGGGGGAGAAAGGCCCGGGGAACGTGGCTC----CCCCGGGAGTGTT-
ATAGCCCCGGGCGCAATGCCCCCGCGGGGGACCGAGGACCGCGCT-CT----
GCAAGGATGCTGGCGTAATGGTCGTCGACGACCCGTCTTGAAACACGGACCAAGGAGTCGAACAT
CTGTGCGAGTGTCTGGGTG--CCAAACCCTCGCGCGTAATGAAAGTGAACGTAGGTGAGAGCCCCC--
---CG---GGGCGCATCATCGACCGATCCGGATG-
TCTTCGGATGGATTTGAGTAGGAGCACAGCTGTTCGGACCCGAAAGATGGTGAACTATGCGTGGAT
AGGGTGAAGCCAGAGGAAACTCTGGTGGAGGCTCGCAGCGGTTCTGACGTGCAAATCGATCGTCG
AATCTGCGCATGGGGGCGAAAGACTTATCGA-
ACCATCTAGTAGCTGGTTACCGCCGAAGTTTCCCTCAGGATAGCAGTGTTGTG-
TCTTCAGTTTTATGAGGTAAAGCGAATGATTAGGGACTCGGGGGCGCTCTTTAGCCTTCATCCATTC
TCAAACTTTAAATATGTAAGAAGCCCTTGTTACTTCGCTGAACGTGGGCATTCGAATGTATCAACA
CTAGTGGGCCATTTTTGGTAAGCAGAACTGGCGATGCGGGATGAACCGAACGCGGGGTTAAGGTG
CCGGAGTGGACGCTCATCAGACACCACAAAAGGTGTTAGTACATCTAGACAGTTGGACGGTGGCC
ATGGAAGTCGGAATCCGCTAAGGACTGTGTAACAACTCACCA---

CTCTCTCATCTGAGAAGAACCAACACGCCTATTGGGCGAGACGGAAAGCTAGCAAAGCCCCGACA
GCTTCACAATACCCACTGGGGTCTAGTCTGCCCGGCCGAGACGCCCGAGGGTCAGGCCTGCGGTTT
AGTGAAAAACCTATCATTGATGTGTTCAATCAGTGTCGGTACATCCACGGATCCCATCGTGGATTA
TATGATCACCAGAAATATGGAAGTCCTCGAAGAGTATGAACCGCTACGATATCCCGACGCCACCAA
GATCTTCCTCAACGGCTCTTGGATCGGGGTACACCAGAACCCCAAGGCTCTTGTGAGAGACGTGCA
GAACCTGCGCCGAACTAATCAGATTCCGGCCGAGGTGTCGTTGGTTCGTGACATACGCGATCGCGA
ATTCAAGATCTTTTCGGATGCCGGTCGGGTCATGCGTCCTTTGTTTGTCGTACAACAGGAAGAC------
----
CTCGAGGCCGGCGCTAAGAAGGGAACGTTGGCCCTCAATAAGGAAATGATCCAGAGGCTCGAGGC
GGATGTCGAGGTAGACCCCGACAGCGAGGAGTACTTTGGCTGGCAAGGCTTGGTCAACGAAGGTG
TCATCGATTACCTCGACGCCGAGGAGGAAGAGACGGCCATGATCTGCATGACGCCCGAGGACCTT
GAGACATACCGCCAGACCAAGGCCGGATACGCAGTGTCCCAGGATAACGGC------
GATGAGATCAACAAGCGACTGAGGACCAAGGTCAACCCCACCACACACATGTACACCCACTGCGA
AATCCACCCTAGCATGCTCCTAGGTATCTGTGCGAGCATTATTCCGTTCCCGGACCATAATCAGGTA
CCCCTGAACGCGTCCCCCAATCCCCTAATTCTTTGCCCCTCATGTACAAACACCATAAACACAACAC
CCCACAACCCACAACACCACAACACTATCAACTTGCATGGCAATGTTCCCGAAAATGAAAGCTGAC
CGTATCTC-----
ATCAATAGGTTCACCTCCAGACCGGCCAATGTGTAAGAATTACCTACACCCATAACCATCTACCTA
CGAACGCGAAAGAATATGGCGGGGCTCACATGAGTTTATTAGGGTAACCAAATTGGTGCCGCTTTC
TGGCAAACCATTTCTGGCGAGCACGGTCTCGACAGCAATGGCGTGTACGTATTTTGTTTATCAATTC
TTACGACGAAGGTAGAAACTAATGATCTGTCAACAGCTACAACGGAACCTCCGAGCTCCAACTGG
AGCGCATGAGCGTCTACTTCAACGAGGTATGCCTTGATCAAGAGCAGGAATATTTGCGCAAGACTC
CTTACTGATTATTATGACATGCGCAGGCTTCCGGCAACAAGTACGTGCCTCGTGCCGTCTTGGTCGA
TCTCGAGCCCGGTACCATGGACGCTGTTCGTGCTGGTCCTTTTGGTCAGCTCTTCCGACCCGACAAC
TTCGTTTTCGGTCAGTCCGGTGCCGGCAACAACTGGGCCAAGGGTCATTACACGGAGGGTGCCGAG
CTGGTCGACGCCGTCTTGGATGTCGTTCGTCGCGAGGCTGAGGGATGCGACTGCCTTCAGGGCTTC
CAGATTACCCATTCGCTCGGTGGTGGTACCGGTGCCGGTATGGGTACCTTGCTGATCTCCAAGATTC
GCGAGGAGTTCCCCGACCGCATGATGGCCACCTTCTCCGTCGTTCCCTCTCCCAAGGTCTCCGACAC
CGTCGTCGAGCCTTACAACGCCACCCTCTCCGTCCATCAGCTGGTCGAGAACTCGGACGAGACTTT
CTGCATTGACAACGAGGCTCTATACGACATCTGCATGCGTACCCTGAAGCTATCCAACCCTTCCTAT
GGTGACCTGAACCACTTGGTCTCGGCCGTCATGTCGGGTGTCACCACCTGTCTGCGATTCCCTGGCC
AGCTGAACTCTGACCTCCGCAAGCTGGCCGTCAACATGGTGCCCTTCCCTCGTCTGCACTTCTTCAT
GGTTGGCTTCGCTCCTCTGACCAGCCGCGGTGCTTACTCTTTCCGTGCCGTCACCGTTCCCGAGTTG
ACGCAGCAGATGTTTGACCCGAAGAACATGATGGCCGCCTCGGACTTCCGCAACGGTCGCTACCTG
ACGTGCTCTGCCATCTTGTAAGATACCTAAAACCCCCAGTCCACGAGTGCTTGTTTGCTGACCCTAA
TACTCTAGCCGTGGCAAGATCTCGA

>Hypoxylon_fragiforme

-CATTAGAGGAATACCAAAACCTCCCAACCCCTGTGAA-CTTACCACTGTTTCCTCGGCGTG-CGC----
-------------ACCTGGGGGATCCCTACCCTGTA----
GCTACCCTGTAGCTACCCTACAGGCGCGCTCAAGGTCCCGCCGAAGTACC-CTGAACTCTGTT----
AACGTGGAATTCTGAATGCTTCAACTAAATAAGTTAAAACTTTCAACAACGGATCTCTTGGTTCTG
GCATCGATGAAGAACGCAGCGAAATGCGATAAGTAATGTGAATTGCAGAATTCAGTGAATCATCG
AATCTTTGAACGCACATTGCGCCCATTAGTATTCTAGTGGGCATGCCTATTCGAGCGTCATTTCAAC
CCTTAAGCCTCTGTTGCTTAGCGTTGGGAGTCTACGGGTTACCCTGTAGTTCCTGAAAACCATTGGC
GGAGTCAGGGAGCACTCTAAGCGTATTACATTGTCTCGCTTTGGATATT-
CCCCGCCTCCACGCCGTAAAACCCCCCATA----CAAATGTTGACCTCG---
--------------------AATCTGG-C----CTC-----
GTGGTCCGAGTTGTAATTTGTAGAGGATGCTTTTGGTGCGGT-
GCCTTCCGAGTTCCCTGGAACGGGACGCCAGAGAGGGTGAGAGCCCCGTACGGTTGGA-
CACCTACCCTATATATAGCTCCTTCGACGAGTCGAGTAGTTTGGGAATGCTGCTCTAAATGGGAGG
TAAATTTCTTCTAAAGCTAAATACCGGCCAGAGACC-
GATAGCGCACAAGTAGAGTGATCGAAAGATGAAAAGCACTTTGAAAAGAGGGTTAAATAGCACGT
GAAATTGTTGAAAGGGAAGCG-TTTGCGACCAGACTTTTTCCAGGGGGATCATCCGGTGTTC--
TCACCGGTGCACTCCGCCTGG--TTTAGGCCAGCATCGGTTCT---
CTTAGGGGGATAAAGGCTTGGGGAACGTAGCTC----CTTCGGGAGTGTT-
ATAGCCCCTTGCGTAATACC-TTCG-GGGGACCGAGGATCGCGCT-CT----
GCAAGGATGCTGGCGTAATGGTCGTCAACGACCCGTCTTGAAACACGGACCAAGGAGTCGAACAT
TTGTGCGAGTGTTTGGGTG--TTAAACCCTCACGCGTAATGAAAGTGAACGGAGGTGAGAGCCCTT--
--AC----GGGTGCATCATCGACCGATCCTGATG-
TCTTCGGATGGATTTGAGTAAGAGCATAACTGTTCGGACCCGAAAGATGGTGAACTATGCGTGGAT
AGGGTGAAGCCAGAGGAAACTCTGGTGGAGGCTCGCAGCGGTTCTGACGTGCAAATCGATCGTCA
AATCTGCGC--

GCGTTGTATCGATTCGAATCGACGTTTCCAAATCGAGCTTGCCGCCAAACCTGCCATCATCACCAA
CGGTCTGAAGTACTCCCTCGCCACAGGTAACTGGGGCGACCAAAAGAAGGCGATGAGCTCGACTG
CCGGTGTCTCCCAGGTCCTGAACCGATATACTT------TCGCCTCGA---
CCCTCTCTCACTTGAGACGGACGAACACTCCCATCGGAAGAGACGGGAAGCTTGCGAAGCCTCGAC
AGCTTCACAATACTCATTGGGGTCTTGTCTGTCCAGCTGAGACGCCCGAAGGCCAGGCCTGTGGAC
TGGTGAAGAACTTGTCGCTGATGTGCTCCATCAGCGTGGGTACATCGACGGATCCTATCGTGGAGT
ATATGATTACGAGAAATATGGAAGTCCTGGAGGAATACGAACCGATGCGATACCCTAATGCCACC
AAGATCTTCCTTAACGGTTCTTGGATTGGTGTACACCAGGATCCCAAGACTCTTGTCAAGGATATCC
AGGCGCTTCGTCGGGCCAACCAGATTCCCTCCGAGGTTTCCTTGATCCGCGATATCCGTGATCGTGA
GTTCAAGATATTCTCAGACGCAGGTCGTGTCATGCGCCCCTTGTTTGTCGTGCAACAAGAAGAT-----
----
AATCCCGATCAAGGCATTGCCAAGGGTACATTGGCCCTTACCAAAGAGATGATCCAGCGACTAGA
AGCGGATGTTGATCTTGATCCTGAAAGCGAGGAGTACTTTGGCTGGCAAGGTCTCGTTAACGAGGG
TGTAATCGAGTTTCTCGACGCGGAGGAAGAGGAAACGGCTATGATTTGCATGACACCCGAAGACCT
GGAAAACTACCGGTTGACCAAGCTCGGATTT---
--CCCCTGAACGCGTCC---
AACTCCCCTGATTTTCGCCCCTCACGCACAAAATAA---
ACCACCTCATTCTACAATCGCCGATGTTGAACTGTTGTCAAAACACGCAACAACATTATGCAGAGT
TGAAGCTAATCGCGTCTTTTTATCTCAATAGGTTCATCTTCAGACCGGCCAGTGCGTAAGTAAC------
---
CACCACTACCGATCAAATATCGAATAGGAACTTGGCGGGGCTCACAACAGTATTGTAGGGTAACCA
AATCGGTGCTGCTTTCTGGCAAACTATCTCCAGCGAGCACGGTCTTGACAGCAATGGCGTGTACGT
ATTTAATTCGCCAATTCCCAAAAACGCGATATGTACTGACCGCCAATAAATAGCTACAATGGCACT
TCAGAGCTCCAGCTCGAGCGCATGAGCGTCTACTTTAATGAGGTACACAGCCGAAGAGCCCCCAAT
GATGTGGTTAGGAATAGTTACTAATCACCCCAACATGCACAGGCATCTGGTAACAAGTATGTCCCC
CGCGCCGTCCTCGTCGATCTCGAACCCGGTACCATGGACGCCGTCCGTGCTGGTCCCTTCGGCCAA
CTCTTCCGACCCGACAACTTCGTTTTCGGCCAATCCGGTGCCGGAAACAACTGGGCCAAGGTCAT

TACACTGAGGGTGCTGAGCTAGTCGACCAGGTTCTCGATGTCGTTCGTCGTGAGGCTGAGGGCTGT
GACTGCCTTCAGGGTTTCCAGATCACCCACTCCCTCGGTGGTGGTACCGGTGCCGGTATGGGTACTT
TGTTGATCTCCAAGATCCGCGAGGAGTTCCCCGACCGCATGATGTCCACCTACTCCGTTGTTCCCTC
TCCCAAGGTCTCTGACACCGTCGTTGAACCTTACAACGCCACTCTCTCCGTCCATCAGCTGGTCGAG
AACTCAGACGAGACCTTCTGCATTGATAACGAGGCCTTGTACGACATTTGCATGCGCACTCTCAAG
TTGTCCAACCCTTCGTATGGCGACCTGAACCACCTGGTCTCTGCCGTCATGTCCGGTGTTACTACTT
GCCTGCGTTTCCCCGGCCAGTTGAACTCTGACCTACGCAAGCTAGCCGTCAACATGGTTCCCTTCCC
TCGTCTGCATTTCTTCATGGTCGGCTTCGCTCCTCTTACCAGCCGTGGTGCCTACACTTTCCGTGCTG
TCACCGTTCCCGAGTTGACTCAGCAGATGTTCGACCCAAAGAACATGATGGCCGCCTCTGACTTCC
GCAACGGTCGTTACTTGACGTGCTCTGCCATCTTGTACGTTTACCCCCCAAACCC------------------------
----

>Hypoxylon_howeanum

--ATTAGAGGAATACCAAAACCTCCCAACCCCTGTGAA-CTTACCACTGTTTCCTCGGCGTG-CGC-----
------------ACCTGGGAGATGCTTACCCTGTA----
GCTACCCTGTAGCTACCCTACAGGCGCGCTCAAGGTCCCGCCGAAGTACC-CTGAACTCTGTT----
TACGTGGAATTCTGAATGCTTCAACTAAATAAGTTAAAACTTTCAACAACGGATCTCTTGGTTCTGG
CATCGATGAAGAACGCAGCGAAATGCGATAAGTAATGTGAATTGCAGAATTCAGTGAATCATCGA
ATCTTTGAACGCACATTGCGCCCATTAGTATTCTAGTGGGCATGCCTATTCGAGCGTCATTTCAACC
CTTAAGCCCCTGTTGCTTAGCGTTGGGAGTCCACAGGTTACCCTGTAGCTCCTGAAAACCATTGGCG
GAGTCTGGGAGCACTCTAAGCGTATTATATTGTCTCGCTTTGGATCTTGCCCCGCCTCCACGCCGTA
AAACACCCCATA----CAAATGTTGACCTCG-ATTAGGTAGGAATA--

TGGAACGGGACGCCAGAGAGGGTGAGAGCCCCGTACGGTTGGA-
CACCTACCCTATATATAGCTCCTTCGACGAGTCGAGTAGTTTGGGAATGCTGCTCTAAATGGGAGG
TAAATATCTTCTAAAGCTAAATACCGGCCAGAGACC-
GATAGCGCACAAGTAGAGTGATCGAAAGATGAAAAGCACTTTGAAAAGAGGGTTAAATAGCACGT
GAAATTGTTGAAAGGGAAGCG-TTTGCGACCAGACTTTTTCCAGGCGGATCATCCGGTGTTC--
TCACCGGTGCACTTCGCCTGG--TTTAGGCCAGCATCGGTTCT---
CTTAGGGGGATAAAGGCTTGGGGAACGTAGCTC----CTTCGGGAGTGTT-
ATAGCCCCTTGCGTAATACCCTTCG-GGGGACCGAGGATCGCGCT-CT----
GCAAGGATGCTGGCGTAATGGTCGTCAACGACCCGTCTTGAAACACGGACCAAGGAGTCGAACAT
TTGTGCGAGTGTTTGGGTG--TTAAACCCTCACGCGTAATGAAAGTGAACGGAGGTGAGAGCCCTT--
--AC----GGGTGCATCATCGACCGATCCTGATG-
TCTTCGGATGGATTTGAGTAAGAGCATAACTGTTCGGACCCGAAAGATGGTGAACTATGCGTGGAT
AGGGTGAAGCCAGAGGAAACTCTGGTGGAGGCTCGCAGCGGTTCTGACGTGCAAATCGATCGTCA
AATCTGCGCATGGGGGCGAAAGACTTATCGA-
ACCATCTAGTAGCTGGTTACCGCCGAAGTTTCCCTCAGGATAGCAGTGT--TG-
TTTTCAGTTTTATGAGGTAAAGCGAATGATTAGGGACTCGGGGGCGCTATTTAGCCTTCATCCATTC
TCAAACTTTAAATATGTAAGAAGCCCTTGTTACTTAATTGAACGTGGGCATTCGAATGTATCAACA
CTAGTGGGCCATTTTTGGTAAGCAGAACTGGCGATGCGGGATGAACCGAACGCGGGGTTAAGGTG
CCAGAGTGGACGCTCATCAGACACCACAAAAGGTGTTAGTACATCTTGACAGCAGGACGGTGGCC
ATGGAAGTCGGAATCCGCTAAGGACTGTGTAACAACTCACCTGCCGAATGTACTAGCCCTGAAAAT
GGATGGCGCTCAAGCGTCT-CACCCATACCTCGCCCTTAGGGTAGAAACGATGCCCTAAGG-----------
----

CATTTGAGAAGAACGAACACTCCTATTGGAAGAGACGGAAAGTTGGCGAAGCCCCGACAGCTTCA
CAACACGCACTGGGGTCTTGTCTGTCCAGCCGAGACGCCCGAAGGTCAGGCCTGTGGATTGGTCAA
AAATCTGTCGTTGATGTGCTCTATCAGTGTGGGTACATCGACCGACCCGATTGTAGACTATATGATC
ACTCGAAGCATGGATGTTTTAGAGGAATATGAACCAAAGACCAATCCTAATGCTACGAAAATCTTC
TTGAACGGCTCTTGGATTGGCACTCACACAGACCCCAAGGCTCTCGTCAGGGATATTCAGGAATTA
CGACGAGCTAACCAGATTCCATCTGAGGTGTCATTGGTGCGCGACATTCGCGATCGCGAGTTCAAG
ATATTCTCTGATGCTGGTCGAGTTATGCGCCCACTATTTGTCGTTCAACGAGAGGAT--------------------
----

GACCAGGAGAAGAGTATCGTCAAGGGGTCGTTGGCTCTCACAAAGGACATGATACAGAGGCTGGA
GGCCGACAATGATCAAGATCCCGACAGTGAAGATTATTTGGTTGGCAAGGACTAGTCAACGACG
GTGCTATTGAATACCTCGATGCTGAGGAAGAGGAGACGGCGATGATTTGCATGACGCCGGAGGAT

CTCGATACGTTTCGTTTGGCCAAGGCTGGTTATGATACGAACCAGGACAACGGC------
GACGAAATTAACAAACGGTTGAAAACCAAAGTCAACCCCACAACTCACATGTACACACATTGTGA
GATCCATCCTAGCATGCTCTTGGGAATCTGTGCCAGTATTATTCCCTTCCCAGATCACAATCAGGTA
CCCCTGAACGCGTCC---AACTCCCCTGATTTTCGCCCCTCACGCACAAAATAA---
ACCACATCATTCTACAATCCCCTATGTTGAACTGTTGTCAAATCACGCAACAACATTATGGAGAGTT
GAAGCTAATCGCGTCT-TTTATCTAAACAGGTTCATCTTCAGACCGGCCAGTGCGTAAGTAAC--------
-

CACCACTACCGATCAAATACCGAGCAGGAATATAGCGGGGCTCACAACAGTATTGTAGGGTAACC
AAATCGGTGCTGCTTTCTGGCAAACTATCTCCAGCGAGCACGGTCTCGACAGCAATGGCGTGTACG
TATTTAATTCGCCAATTCAGAAGAGGAAAAAATGTGCTGACCGCCAATAAATAGCTACAATGGCAC
TTCAGAGCTCCAGCTCGAGCGCATGAGCGTCTACTTTAACGAGGTACACAGCCGAAGAGCCCCCAA
TGATATGGTTAGGAATAGTTACTAATCACCCCAACATGCACAGGCATCTGGTAACAAGTATGTCCC
CCGCGCCGTCCTCGTCGATCTCGAACCCGGTACCATGGACGCCGTCCGTGCTGGTCCCTTCGGCCA
ACTCTTCCGACCCGACAACTTCGTTTTCGGCCAATCCGGTGCCGGCAACAACTGGGCCAAGGGTCA
TTACACTGAGGGTGCTGAGCTTGTCGACCAGGTTCTCGATGTCGTTCGTCGTGAGGCTGAGGGCTG
TGACTGCCTTCAGGGTTTCCAGATCACCCACTCCCTCGGTGGTGGTACCGGTGCTGGTATGGGTACC
TTGTTGATCTCCAAGATCCGCGAGGAGTTCCCTGACCGCATGATGTCCACCTACTCCGTTGTTCCCT
CCCCCAAGGTCTCCGACACCGTCGTTGAACCTTACAACGCCACTCTCTCCGTCCACCAGCTGGTCGA
GAACTCAGATGAGACCTTCTGCATTGACAACGAGGCCTTGTACGACATTTGCATGCGCACTCTAAA
GTTGTCTAACCCTTCGTACGGTGACCTGAACCACCTGGTCTCTGCCGTCATGTCTGGTGTTACTACT
TGCCTACGTTTCCCCGGCCAGTTGAACTCTGACCTACGCAAGCTAGCCGTCAACATGGTTCCCTTCC
CTCGTCTCCATTTCTTCATGGTCGGCTTCGCTCCTCTTACCAGCCGTGGTGCCTACACCTTCCGTGCT
GTCACCGTTCCCGAGTTGACTCAGCAGATGTTCGACCCCAAGAACATGATGGCCGCCTCTGACTTC
CGCAACGGTCGTTACTTGACGTGCTCTGCCATCTTGTACGTTTATTCCCCAAACCCATTGGTCCCAA
TTCGGTTGCTAACTATTAGCTTCTAGCCGTGGCAAGGTCTCCA

>Hypoxylon_ticinense

-CATTAGAGGAATATCAAAACCTCCTAACCTGTGTGAACCTTATC--CTGTGTTTCGGCGTG-TGCGC--
CTACCCTGGAGCTACCCTGGAGAAATCTACCCGGGAGGAAGATACCCTGTAGCTGCCCTGGGGCCG
CGCCCACGGGCCCGCCGAAAGAACCC-AAATTCTATT----
TGTGTGGAATTCGAAAAGCTTCAACTAAATAAGTTAAAACTTTCAACAACGGATCTCTTGGTTCTG
GCATCGATGAAGAACGCAGCGAAATGCGATAAGTAATGTGAATTGCAGAATTCAGTGAATCATCG
AATCTTTGAACGCACATTGCGCCCATTAGTATTCTAGCGGGCATGCCTATTCGAGCGTCATTTCAAC
CCTTAAGCCTCTGTTGCTTAGTGTTGGGAGCCTGCGTGTTACAGCGCAGTTCCTGAAAATTATCGGC
GGAGTTAGGGAGCATTCCAAGCAAAATACGTGTTTTTACACCTGCATCAGCCCCGCTTCCTTGCCGT
AAACCCCCCTAAT----
TTCATGTTGACCTCGGATTAGGTAGGAATACCCGCTGAACTTAAGCATATGAAGCGGCAACAGCTC
AAA-TTTGAAATCTGGCC----CTC-----
GCGGTCCGAGTTGTAATTTGTAGAGGATGCTTTTGGTGCGGT-
GCCTTCCGAGTTCCCTGGAACGGGACGCCAGAGAGGGTGAGAGCCCCGTACGGTTGGA-
CACCTACCCTATACATAGCTCCTTCGACGAGTCGAGTAGTTTGGGAATGCTGCTCTAAATGGGAGG
TAAATTTCTTCTAAAGCTAAATACCGGCCAGAGACC-
GATAGCGCACAAGTAGAGTGATCGAAAGATGAAAAGCACTTTGAAAAGAGGGTTAAATAGCACGT
GAAATTGTTGAAAGGGAAGCG-TTTGCGACCAGACTTTTTCCAGGCGGATCATCCGGTGTTC--
TCACCGGTGCACTTCGCCTGG--TTTAGGCCAGCATCGGTTCT---
CTTAGGGGGATAAAGGCTCGGGGAACGTAGCTC----CTTCGGGAGTGTT-
ATAGCCCCTTGCGTAATACCCTTCG-GGGGACCGAGGATCGCGCT-CT----
GCAAGGATGCTGGCGTAATGGTCGTCAACGACCCGTCTTGAAACACGGACCAAGGAGTCGAACAT
TTGTGCGAGTGTCTGGGTG--TTAAACCCTCACGCGTAATGAAAGTGAACGGAGGTGAGAGCCCTT--
--GC----GGGTGCATCATCGACCGATCCTGATG-
TCTTCGGATGGATTTGAGTAAGAGCATAACTGTTCGGACCCGAAAGATGGTGAACTATGCGTGGAT
AGGGTGAAGCCAGAGGAAACTCTGGTGGAGGCTCGCAGCGGTTCTGACGTGCAAATCGATCGTCA
AATCTGCGCATGGGGGCGAAAGACTTATCGA-
ACCATCTAGTAGCTGGTTACCGCCGAAGTTTCCCTCAGGATAGCAGTGT--TG-
TTTTCAGTTTTATGAGGTAAAGCGAATGATTAGGGACTCGGGGGCGCTATTTAGCCTTCATCCATTC
TCAAACTTTAAATATGTAAGAAGCCCTTGTTGCTTAATTGAACGTGGGCATTCGAATGTATCAACA
CTAGTGGGCCATTTTTGGTAAGCAGAACTGGCGATGCGGGATGAACCGAACGCGGGGTTAAGGTG

CCAGAGTGGACGCTCATCAGACACCACAAAAGGTGTTAGTACATCTTGACAGCAGGACGGTGGCC
ATGGAAGTCGGAATCCGCTAAGGACTGTGTAACAACTCACCTGCCGAATGTACTAGCCCTGAAAAT
GGATGGCGCTCAAGCGTCT-
CACCCATACCTCGCCCTTAGGGTAGAAACGATGCCCTAAGGACGATGCATCGACCAGAACAGACG
TTTCCAGATTGAGCTTGCAGCGAAGCCTGCCATCATCACGAATGGACTAAAGTATTCGCTCGCCAC
AGGAAATTGGGGTGATCAAAAGAAGGCGATGAGCTCGACTGCTGGTGTGTCTCAAGTTCTAAATCG
ATACACGT------TCGCTTCGA---
CCCTTTCTCATTTACGAAGAACGAACACCCCCATCGGAAGAGACGGAAAGTTGGCGAAGCCTCGCC
AGCTTCACAACACGCATTGGGGTCTGGTCTGTCCAGCCGAGACGCCCGAGGGTCAGGCCTGTGGCC
TGGTGAAGAATCTCTCGCTAATGTGCTCCATCAGCGTGGGTACTTCAACGGATCCTATCGTAGACT
ATATGATTACACGTAGCATGGACGTCTTGGAGGAATATGATCCAAAAACACGACCCAATGATACG
AAAATCTTTCTGAATGGCTCCTGGATTGGTACACACCAGGACCCTAAGGCCCTTGTTAGGGATATC
CAGGAACTGCGACGATCCAACCAGATTCCGTCCGAGGTATCCTTGGTCCGCGACATTCGTGACCGC
GAATTCAAAATCTTCTCCGACGCTGGCCGCGTTATGCGCCCACTATTTGTCGTTCAACGAGAGGAT--
----

GACCCAGAGAAGCATATTGTCAAGGGATCATTAGCTCTTACAAAGGACATGATACAGAGGCTGGA
AGCCGACGTTGATCAAGATCCCGAGAGTGAAGATTACTATGGCTGGCAAGGTCTAGTCAACGAAG
GTGCCATTGAGTATCTTGACGCTGAGGAGGAGGAAACGGCGATGATTTGCATGACACCGGAGGAT
CTGGAGACTTTCCGTTTGACCAAGGCCGGCTATGAGGTGAGCCAGGACAACGGC------
GACGAGATCAACAAGCGGCTCAAGACCAAAGTCAATCCCACGACTCACATGTACACACACTGCGA
AATTCATCCTAGCATGCTCCTAGGAATCTGTGCCAGTATTATTCCATTCCCAGATCATAATCAGGTA
ACCCTGAACGCGTCC---AACTCCCCTGATTTTCGCCCCTCACGCACAAAATCA---ACC-------------TT--
-----
TTGTGCTATCGTCAAGTCACGCAACAACGTTATAGAAAGATGAAGCTAATCGCGTCTTTTTTATCGT
ATAGGTTCACCTTCAGACCGGCCAGTGCGTAAGTAAT---------
CACCGCTACCGATCAAACATCGAATTGGAACATGGCGGGGCTCACAGCAATATTGTAGGGTAACC
AAATCGGTGCTGCTTTCTGGCAAACCATCTCCAGCGAGCACGGTCTCGACAGCAACGGCGTGTATG
TATTTTATTCGTCAATTTGCGCAGTGATTTCCCCTGCTAACCGCCAATAAACAGCTACAATGGCACT
TCGGAGCTCCAGCTCGAGCGCATGAGCGTCTACTTTAACGAGGTACACAGCCGAAGAGCCCCTGAT
GATATCGTTAGGAATAGTTACTAATCACCCCAACATGCACAGGCATCTGGTAACAAGTATGTCCCC
CGCGCCGTCCTCGTCGATCTCGAACCCGGAACCATGGACGCCGTCCGTGCTGGTCCCTTTGGCCAG
CTCTTCCGACCCGACAACTTCGTTTCGGCCAATCCGGTGCCGGCAACAACTGGGCCAAGGGTCAC
TATACGGAGGGTGCTGAGCTTGTCGACCAAGTTTTGGATGTTGTTCGTCGTGAGGCAGAAGGCTGT
GACTGCCTCCAGGGTTTCCAGATCACCCACTCCCTTGGTGGTGGTACCGGTGCCGGTATGGGTACTC
TGTTGATCTCCAAGATCCGTGAGGAGTTCCCCGACCGCATGATGTCCACATACTCCGTCGTTCCCTC
TCCTAAGGTCTCCGACACCGTCGTTGAACCTTACAATGCCACTCTCTCCGTCCACCAGCTGGTCGAG
AATTCGGACGAGACCTTCTGCATTGACAACGAGGCCCTATACGACATCTGCATGCGTACTCTTAAG
CTGTCGAACCCTTCATACGGTGACCTGAACCACCTGGTCTCTGCCGTCATGTCTGGCGTCACCACTT
GTCTGCGTTTCCCCGGCCAACTGAACTCTGACCTGCGCAAGCTTGCCGTCAACATGGTTCCTTTCCC
TCGTCTTCACTTCTTCATGGTCGGCTTCGCTCCCCTGACCAGCCGTGGTGCCTACACCTTCCGTGCTG
TTACTGTCCCCGAGTTGACTCAGCAGATGTTCGACCCCAAGAACATGATGGCCGCCTCTGACTTCC
GTAACGGCCGTTACCTGACATGCTCTGCCATCTTGTAAGATTACCTCTCGTCCGTTTTTTCGTGCCT
TGTTCGCTAATTCTTTGCTTTTAGCCGCGGCAAGGTCTCCA

>Annulohypoxylon_nitens

------CTGAGTTTCAACAACTCC-
AACCCTTTGTCGAACCTACCACTGTTTCCTCGGCGTACTGCGCGGCCTCTGGGCCGCT------------------
----------------GCAG--------------CGCC------------
GAAGGACCGTCAAACTCTTTTTTCTGTAAAACTCATATAAAATTTATTACAAAATAAGTTAAAACTT
TCAACAACGGATCTCTTGGTTCTGGCATCGATGAAGAACGCAGCGAAATGCGATAAGTAATGTGAA
TTGCAGAATTCAGTGAATCATCGAATCTTTGAACGCACATTGCGCCCATTAGTATTCTAGTGGGCAT
GCCTATTCGAGCGTCATTTCGACCCTTAAGCCCCAGTTGCTTAGCGTTGGGAGTCTGCGGCCCAGGC
CGCAGTTCCTCAAAGTCAGTGGCGGAGTTGTAGCACACTCTAAGCGTAGTAGTTTGCCTCGCAAAC
AGAGCGGCCTCAGCTGCCAGCCGTAAAGCCCTATA--TTCTTAG---
--CGGTCCGAGTTGTAATTTGCAGAGGATGCTTTTGGTGCGGT-
GCCTTCCGAGTTCCCTGGAACGGGACGCCATAGAGGGTGAGAGCCCCGTACGGTTGGA-
CACCTAGCCTCTATATAGCTCCTTCGACGAGTCGAGTAGTTTGGGAATGCTGCTCTAAATGGGAGG

TAAATTTCTTCTAAAGCTAAATACCGGCCAGAGACC-
GATAGCGCACAAGTAGAGTGATCGAAAGATGAAAAGCACTTTGAAAAGAGGGTTAAATAGCACGT
GAAATTGTTGAAAGGGAAGCG-TTTGCGACCAGACCTTCTCCAGGGGGATCAACCGTTATTT--
TTAGCGGTGCACTCCCCCTGG--TTTAGGCCAGCATCGGTTCT---
CTTAGGGGGATAAAGGCCTGGGGAACGTAGCTC----TTTAGGGAGTGTT-
ATAGCCCCTAGCGTAATACCCTTCA-GGGGACCGAGGACCGCGCT-TCG---
GCAAGGATGCTGGCGTAATGGTCGTCAACGACCCGTCTTGAAACACGGACCAAGGAGTCGAACAT
TTGTGCGAGTGTTTGGGTG--TTAAACCCTCACGCGTAATGAAAGTGAACGGAGGTGAGAGCCTT----
-A-----GGGTGCATCATCGACCGATCCTGATG-
TCTTCGGATGGATTTGAGTAAGAGCATAACTGTTCGGACCCGAAAGATGGTGAACTATGCGTGGAT
AGGGTGAAGCCAGAGGAAACTCTGGTGGAGGCTCGCAGCGGTTCTGACGTGCAAATCGATCGTCA
AATCTGCGCATGGGGGCGAAAGACTTATCGA---

GATCAAAAGAAGGCAATGAGCTCGACCGCAGGCGTGTCACAGGTCTTGAACCGATACACAT------
TCTCGTCCA---
CACTTTCGCATTTGCGACGAACGAACACGCCCATCGGGAGAGATGGAAAACTAGCCAAGCCGCGG
CAGCTTCACAACACCCATTGGGGTCTCGTCTGTCCGGCGGAGACTCCCGAGGGCCAAGCTTGTGGC
TTGGTGAAAAACTTATCTTTGATGTGTTCTATCAGCGTCGGGACGTCAACGGATCCTATCGTGGACT
ATATGATTACTAGGAACATGGAAGTCCTGGAGGAATATGAGCCGATGAGATACCCGAATGCCACT
AAGATCTTCCTCAATGGCTCTTGGATCGGTGTGCATCAGGATCCCAAGACCCTCGTCAGAGATGTC
CAAGCGCTCCGTCGAGCCAACCAGATTCCCGCCGAGGTATCGTTGGTTCGTGATATTCGAGATCGT
GAATTCAAGATCTTTTCAGATGCCGGACGTGTGATGCGTCCTTTGTTCCGTGTACAGCAGGAAGAT-
----
ATCGAAGAGCAGGGTATCGAAAAGGGCACATTAGCTCTTACTAAGCAGATGATAAAGCGCCTGGA
AGCAGATGTTGACTTAGATCCGGAAAGCGAAGCGTACTTTGGCTGGCAGGGTCTAGTTAATGAGGG
TGTTGTTGAGCTCCTTGATGCGGAAGAGGAGGAGACTGCTATGATTTGCATGACGCCCGAAGACTT
GGATA---

ATGGACGCCGTCCGTGCTGGCCCTTTCGGCCAACTTTTCCGTCCCGACAACTTCGTCTTCGGCCAGT
CTGGTGCCGGAAACAACTGGGCGAAGGGTCACTACACTGAGGGTGCTGAGCTAGTTGACAACGTT
CTTGATGTTGTCCGTCGTGAGGCTGAGGGCTGTGACTGCCTCCAGGGTTTCCAGATCACCCACTCTC
TCGGTGGTGGTACCGGTGCCGGTATGGGTACCCTGCTGATCTCTAAGATCCGCGAAGAGTTCCCCG
ACCGTATGATGGCTACCTTCTCCGTCGTCCCCTCCCCCAAGGTTTCCGACACCGTCGTCGAGCCTTA
CAACGCCACTCTCTCAGTTCACCAGCTGGTTGAGAACTCCGACGAGACCTTCTGTATCGACAACGA
AGCTCTGTACGATATCTGCATGCGTACTCTCAAGCTGTCCAACCCCTCTTACGGCGACCTTAATCAC
CTGGTCTCTGCCGTCATGTCTGGTGTCACCACTTGCTTGCGTTTCCCTGGTCAGCTTAACTCTGACTT
GCGCAAGCTTGCCGTCAACATGGTTCCTTTCCCTCGTCTTCACTTCTTCATGGTCGGCTTCGCTCCCT
TGACCAGCCGTGGCGCCTACTCCTTCCGCGCTGTTACCGTTCCCGAGTTGACCCAGCAGATGTTCGA
CCCCAAGAACATGATGGCTGCTTCTGACTTCCGCAACGGTCGTTACCTAACGTGCTCTGCCATCTTG

>Hypoxylon_ochraceum

-CATTACCGAGTTATAAAAAACTCCCACCCTTTGTGAA-
TTTACTCTTGTTGCCTCGGCGTTGGGAGCACCTACCCTGGAGCTACCCTGAAGAGACCTACCCTGGA
GAAGGCTACCCTGCACCTACCCTGGAGATGCACTTTACACCACGCCGGTGGACTTTATAACTCTGTT
TTTTCCACTGAATCTCTGAAAAAATA-
ACTAAATTAGTTAAAACTTTCAACAACGGATCTCTTGGTTCTGGCATCGATGAAGAACGCAGCGAA
ATGCGATAAGTAATGTGAATTGCAGAATTCAGTGAATCATCGAATCTTTGAACGCATATTGCGCCC
ATTAGTATTCTAGTGGGCATGCCTATTCGAGCGTCATTTCAACCCTTAAG-
CCCTGTTGCTTAGTGTTGGGTATCTACCCTGTAAAGAGTAGTTCCCTAAATGTAGTGGCGGTGTTT-

AGGGCACTGGTAGCGTAGTAAAATTTCTCGCCTCTAAAGT-
GCCCTAGAAGCCTGCCGTAAAATTATATATATATATCAAGGTTGACCTCGGATTAGGTAGGAATAC
CCGCTGAACTTAAGCATA--

ACGCTGCATCGATACAAACAAACATTTTGAAATCGCCCTAGCTGCCAAGCCGGCTATCATTACTAA
CGGTCTTAAATACTCGCTTGCTACAGGAAATTGGGGTGACCAGAAGAAGGCCATGAGCTCGACRGC
YGGCGTCTCGCAGGTCTTGAACCGATACACGT------TCGCTTCGA---
CACTTTCTCATCTGAGACGAACCAACACGCCTATTGGGCGGACGGAAAACTGGCGAAGCCCCGG
CAACTTCACAATACCCATTGGGGTTTAGTCTGCCCGGCCGAAACACCCGAAGGTCAGGCCTGCGGT
TTGGTCAAGAACTTATCACTGATGTGTTCAATCAGTGTTGGCACTTCGACGGACCCTATCGTTGATT
ATATGATTACTAGGAATATGGAGGTCCTTGAAGAGTACGAACCACTGCGATACCCCGACGCTACCA
AGATCTTCCTGAACGGTTCCTGGATCGGCGTGCATCAGAACCCCAAGTCTCTCGTGAGAGATGTTC
AGAATTTGCGGCGGACAAACCAGATTCCGGCCGAGGTGTCATTGGTCCGCGATATACGTGATCGTG
AATTCAAGATCTTTTCGGACGCTGGTCGCGTCATGCGCCCCTTGTTCGTCGTACAGCAAGAAGAC---
----
ACCGAGGCTGGTACTAAGAAGGGGACGCTGGCCCTTACAAAAGAAATGATTCAGAGGCTCGAGGC
GGATGTCGACGTAGATCCCAATAGTGAGGAGTACTTTGGCTGGCAAGGCTTGGTCAACGAAGGTGT
CATCGATTATCTCGACGCCGAGGAGGAAGAGACGGCCATGATCTGCATGACACCCGAGGATCTTG
AGACATACCGCCAAACCAAGGCCGGATACGAAGTGTCCCAGGATAACGGG------
GATGAGATTAATAAGCGACTGAGGACCAAGGTTAACCCGACCACGCATATGTACACTCATTGCGA
AATCCATCCTAGTATGCTTCTAGGTATYTGCGCGAGCATTATTCCGTTCCCCGATCATAATCAGGTA

CGTCCCCCATCCCCCAACCCCCTGATTTCCTACCCCTCACGCACAAACATCACAAACACGAAACGA
TTCGATTCCCTACATTGC---------AGAACGC--TGGA---
TTCTAAAAATCAAATGCTAACCATATATTTTCATCTCGATAGGTTCACCTCCAGACCGGCCAATGCG
TAAGATCTACCTATCCCTACAACTACCGACCA-
TCGACGCGAAAGAATATAGCGGGGCTCACATGAATTTTACAGGGTAACCAAATTGGTGCCGCTTTC
TGGCAGACCATCTCTGGCGAGCACGGCCTCGACAGCAATGGCGTGTACGTGTTTAATTGGGCAATT
CCAACAAGGGAATTAAGAACTAATAATT---
CGATAGCTACAACGGAACCTCTGAGCTCCAACTGGAGCGCATGAGCGTCTACTTCAACGAGGTACG
TAATAAACACAACAAGAAGTGCATGCGAAAGACTCGTTATTAATTACTTTGACGTGCACAGGCTTC
CGGCAACAAGTATGTTCCCCGTGCTGTCCTTGTCGATCTTGAGCCTGGTACCATGGACGCCGTCCGT
GCTGGTCCCTTCGGTCAGCTCTTCCGACCCGACAACTTCGTCTTCGGTCAATCCGGTGCTGGCAACA
ACTGGGCCAAAGGTCACTACACTGAAGGTGCTGAGCTGGTTGACAACGTCTTGGATGTTGTCCGTC
GCGAGGCTGAGGGCTGCGACTGCCTCCAGGGTTTCCAAATCACCCACTCGCTCGGTGGTGGTACTG
GTGCCGGTATGGGTACCTTGTTGATCTCCAAGATCCGCGAGGAGTTCCCTGACCGTATGATGGCCA
CTTTCTCCGTCGTTCCCTCCCCTAAGGTCTCCGACACCGTTGTCGAGCCTTACAACGCCACTCTCTCC
GTCCACCAGCTGGTCGAGAACTCGGACGAGACCTTCTGCATTGATAACGAGGCTCTCTACGATATC
TGCATGCGTACCCTAAAGCTATCCAACCCCTCGTATGGTGACCTGAACCACCTGGTCTCCGCCGTCA
TGTCGGGTGTTACCACTTGCTTGCGATTCCCTGGCCAGCTGAACTCTGACCTCCGCAAGTTGGCTGT
CAACATGGTGCCCTTCCCTCGTCTGCACTTCTTCATGGTCGGCTTCGCCCCCCTGACCAGCCGTGGT
GCCTACTCCTTCCGTGCCGTCACCGTTCCCGAGTTGACGCAGCAGATGTTCGACCCCAAGAACATG
ATGGCTGCTTCTGACTTCCGAAATGGTCGCTACCTGACGTGCTCTGCCATCTTGTAAGTGGCTTGGT
CCAAATTGTCGATTAGTACTTGTTTACTAACACTAAATTAATAGCCGTGGCAAGATCTCCA

>Rhopalostroma_angolense

A-------------------CTCCCACCCTTTGCGAACCTTACCACTGTTGCCTCGGCGGGACGCGC-----------------
-------------------------------GGAG----------AACGCGC-TACAGGCCCGCCGGCGGACTGCTATATTCTGT-
CAGTGTAACGTAACTCTGAATGCTTCAAACAAATAAGTTAAAACTTTCAACAACGGATCTCTTGGT

TCTGGCATCGATGAAGAACGCAGCGAAATGCGATAAGTAATGTGAATTGCAGAATTCAGTGAATC
ATCGAATCTTTGAACGCACATTGCGCCCGTTAGTATTCTAGCGGGCATGCCTGTTCGAGCGTCATTA
CGACCCTTAAG-
CCCTGTAGCTTAGCGTTGGGAACCTAGGTTTCTGTGCCCAGCTCCCTAAAGGTAGTGGCGGAGTCG
GGGCCTGCTCTGAGCGTAGTAGTTCTTCTCGCTTCGGTAGTAGTCCCGGCGGCCTGCCGTAAAACA
ACCCCTA---GAAGCGGCAACAGCTCAAA-
TTTGAAATCTGGCC----CTA-----GCGGTCCGAGTTGTACTTTGTAGAGGATGCTTTTGGCGAGGT-
GCCTTCCGAGTTCCCTGGAACGGGACGCCGGAGAGGGTGAGAGCCCCGTACGGTTGGA-
CGCCGAGCCTCTGTATAGCTCCTTCGACGAGTCGAGTAGTTTGGGAATGCTGCTCTAAATGGGAGG
TAAATTTCTTCTAAAGCTAAATACCGGCCAGAGACC-
GATAGCGCACAAGTAGAGTGATCGAAAGATGAAAAGCACTTTGAAAAGAGGGTTAAATAGCACGT
GAAATTGTTGAAAGGGAAGCG-TTTGCGACCAGACTTTTTCCGGGGGGATCATCCGGCGTTC--
TCGCCGGTGCACTTCCCCCGG--TCGAGGCCAGCATCGGTTTC---
CTTAGGGGGATAAAGGCCCGGGGAACGTGGCTC----CTTCGGGAGTGTT-
ATAGCCCCGGGCGTAATACCCCTCG-GGGGACCGAGGAACGCGCT-CT----
GCAAGGATGCTGGCGTAATGGTCGTCAACGACCCGTCTTGAAACACGGACCAAGGAGTCGAACAT
TTGTGCGAGTGTTTGGGTG--TCAAACCCTCACGCGTAATGAAAGTGAACGGAGGTGAGAGCCCTC--
--GC----GGGTGCATCATCGACCGATCCTGATG-
TCTTCGGATGGATTTGAGTAAGAGCATAACTGTTCGGACCCGAAAGATGGTGAACTATGCGTGGAT
AGGGTGAAGCCAGAGGAAACTCTGGTGGAGGCTCGCAGCGGTTCTGACGTGCAAATCGATCGTCA
AATCTGCGCATGGGGGCGAAAGACTTATCGA-
ACCATCTAGTAGCTGGTTACCGCCGAAGTTTCCCTCAGGATAGCAGTGT--
TGTTCTTCAGTTTTATGAGGTAAAGCGAATGATTAGGGACTCGGGGGCGCTATATTGCCTTCATCCA
TTCTCAAACTTTAAATATGTAAGAAGCCCTTGTTACTTAGTTGAACGTGGGCATTCGAATGTACCAA
CACTAGTGGGCCATTTTTGGTAAGCAGAACTGGCGATGCGGGATGAACCGAACGCGGGGTTAAGG
TGCCAGAGTGGACGCTCATCAGACACCACAAAAGGTGTTAGTACATCTTGACAGCAGGACGGTGG
CCATGGAAGTCGGAATCCGCTAAGGACTGTGTAACAACTCACCTGCCGAATGTACTAGCCCTGAAA
ATGGATGGCGCTCAAGCGTCT-CACCCATACCTCGCCCTTAGGGTAGAAACGATGCCCTAAGG-------
----
----TTTTGACCGATACACGT------TCGCGTCAA---
CTCTCTCCCATTTAAGGCGAACGAACACGCCTATTGGAAGAGACGGGAAGCTCGCGAAACCTCGAC
AGCTGCACAATACCCACTGGGGTCTGGTCTGTCCGGCCGAAACGCCCGAAGGCCAGGCTTGCGGTC
TGGTGAAGAATCTGTCGCTGATGTGCTCCATCAGCGTGGGTACCTCAACGGATCCCATCGTAGACT
ATATGATTACTAGGAATATGGAAGTCTTGGAGGAATATGAGCCGATGCGATACCCTAACGCTACCA
AGATCTTCCTCAACGGATCGTGGATCGGCGTGCACCAGGATCCCAAGTCTCTCGTCCGAGACGTCC
AGCAGCTTCGTCGGGCTAACCAAATCCCCTCCGAAGTGTCCCTCGTTCGCGATATTCGTGATCGCG
AGTTCAAGATCTTTTCGGACGCAGGCCGTGTCATGCGGCCCTTGTTCGTGGTGCAGCAAGAGGAC---
----
GATCCCGATGCCGGTATCACGAAAGGGTCGCTGGCCCTTACCAAGGAGATGATCCAGAGGCTGGA
GGCGAGTGTCGATCTCGACCCGGAGAGCGAGGAGTACTTTGGTTGGCAAAGTCTTGTTAACGAGGG
CGTTATCGAGTACCTCGACGCGGAGGAGGAAGAAACGGCCATGATTTGTATGACACCCGAAGATTT
GGAAACCTATCGGATGTCCAAGCTTGGGTACGACGTGTCTCAGGACAACGGA------
GATGAGATCAACAAGCGGCTCAAGACCAAGTTGAATCCCACGACGCACATGTACACGCATTGCGA
GATCCATCCTAGCATGCTCCTGGGTATCTGCGCGAGCATCATCCCCTTCCCCGACCACAACCAGGT
A---------------------CCCCCTGAGTTCTGCCCCTCATGCACGAACGCC---ATCACA--------
CGACATTTTACGCTGCAACCGAATCAGATGTCGTGACAATAAACGTGAATTTTTAAGCTAACCGCG
TTTT-----TCCAATAGGTTCACCTTCAGACCGGCCAATGTGTAAGTAGC---------
AGCGACGACGGGAAGG--
ACCGCGATAGTGAATGGCGGGGCTCATACCAAGGTGATAGGGTAACCAAATCGGTGCTGCTTTCTG
GCAAACCATCTCCAGCGAGCACGGCCTCGACAGCAATGGCGTGTACGTATCCGAGCGGCCAGTCCT
ATCGTCGAGGACGTCAACTGACGACAAATCAATAGCTACAACGGTACTTCCGAGCTTCAGCTCGAG
CGCATGAGCGTCTACTTCAACGAGGTACGGACTTACAGATACCAAAAATAGGCAAATAGAACTAG
CTACTAATCGCCTCGACGCGTACAGGCTTCCGGCAACAAGTATGTTCCCCGTGCCGTCCTCGTCGAT
CTCGAGCCCGGTACCATGGACGCCGTCCGTGCTGGTCCCTTCGGCCAGCTCTTCCGACCCGACAAC
TTCGTTTTTGGTCAGTCCGGTGCTGGCAACAACTGGGCCAAGGGTCACTACACCGAGGGTGCCGAG
CTGGTTGACAACGTTCTTGACGTCGTTCGTCGCGAGGCTGAAGGCTGCGACTGCCTCCAGGGTTTCC
AGATCACCCACTCCCTCGGTGGTGGTACCGGTGCCGGTATGGGTACCCTGCTCATCTCCAAGATCC

GCGAGGAGTTCCCCGACCGTATGATGGCCACCTTCTCCGTCATGCCCTCCCCCAAGGTCTCCGACA
CGGTCGTTGAGCCTTACAATGCAACCCTCTCCGTCCACCAGCTGGTCGAGAACTCGGATGAGACCT
TCTGTATCGACAACGAGGCGCTGTACGACATCTGCATGCGTACCCTGAAGCTGTCTAACCCCTCCT
ACGGAGACCTGAACCACCTGGTCTCCGCCGTCATGTCCGGTGTTACCACTTGCTTGCGTTTCCCTGG
TCAGCTAAACTCTGACCTGCGCAAGCTCGCCGTGAACATGGTTCCTTTCCCTCGTCTCCACTTCTTC
ATGGTCGGCTTCGCTCCCCTGACCAGCCGCGGCGCTTACACTTTCCGTGCCGTCACCGTTCCCGAAT
TGACGCAACAGATGTTCGACCCCAAGAACATGATGGCTGCTTCCGACTTCCGTAACGGTCGTTACC
TGACGTGCTCTGCCATCTTGTATGATATCCTCCCCCCTTCTTTATGCATGACTAGCTTGCTAACCTGG
ACTGTGTAGCCGTGGCAAGGT-----

>Hypoxylon_lechatii_MUCL54609

-CATTACCGAGTTCTTAACTTTGTAAACCCTATGTGAAATGTATTATGTATGCCTCGGCAGG-
AGCGCGCTTTCTCGGTGGCTACCCTGTAGCAACCCCCCTTTCCCGGG-----
CGTGGCGCGGCCCTGTAATTG----CCCGTTCCTGCCAGCAGGCTTCTAAATTCTATTTA--
CCACTATATATTCAGATTATTATGTATAATAAGTTAAAACTTTCAACAACGGATCTCTTGGTTCTGG
CATCGATGAAGAACGCAGCGAAATGCGATAAGTAATGTGAATTGCAGAATTCAGTGAATCATCGA
ATCTTTGAACGCACATTGCGCCCATTAGTATTCTAGTGGGCATGCCTATTCGAGCGTCATTTCAACC
CTTAAGCCTCAGTTGCTTAGCGTTGGGAGACTGAGCCCCGGGGCCCAGCTCCTCAAAGTCAGCGGC
GGAATTAGGGCGTATCCTAAGCGTAGTAGTATATCTCGCTTCTGCGGTACCCCTGATCTCTAGCCGT
TAAACCCCCTATA-
ATCTAGTGGTTGACCTCGGATTAGGTAGGAATACCCGCTGAACTTAAGCATATGAAGCGGCAACAG
CTCAAA-TTTGAAATCTGGCC----CTC-----
GCGGTCCGAGTTGTAATTTGTAGAGGATGCTTTTGGCGCGGT-
GCCTTCCGAGTTCCCTGGAACGGGACGCCATAGAGGGTGAGAGCCCCGTACGGTTGGA-
CGCCTAGCCTCTGTATAGCTCCTTCGACGAGTCGAGTAGTTTGGGAATGCTGCTCTAAATGGGAGG
TAAATTTCTTCTAAAGCTAAATACCGGCCAGAGACC-
GATAGCGCACAAGTAGAGTGATCGAAAGATGAAAAGCACTTTGAAAAGAGGGTTAAATAGCACGT
GAAATTGTTGAAAGGGAAGCG-TTTGCGACCAGACCTTTTCCAGGCGGATCATCCGGCGTTC--
TCGCCGGTGCACTTCGCCTGG--TTTAGGCCAGCATCGGTTTC---
CTTAGGGGGATAAAGGCTTAGGGAACGTATCTC----TCTCGGGAGTGTT-
ATATCCCTTTGCGTAATACCCTTCG-GGGGACCGAGGACCGCGCT-CT----
GCAAGGATGCTGGCGTAATGGTCGTCAACGACCCGTCTTGAAACACGGACCAAGGAGTCGAACAT
TTGTGCGAGTGTTTGGGTG--TTAAACCCTCACGCGTAATGAAAGTGAACGGAGGTGAGAGCCTTT---
CG-----GGGCGCATCATCGACCGATCCTGATG-
TCTTCGGATGGATTTGAGTAAGAGCATAACTGTTCGGACCCGAAAGATGGTGAACTATGCGTGGAT
AGGGTGAAGCCAGAGGAAACTCTGGTGGAGGCTCGCAGCGGTTCTGACGTGCAAATCGATCGTCA
AATCTGCGCATGGGGGCGAAAGACTTATCGA-
ACCATCTAGTAGCTGGTTACCGCCGAAGTTTCCCTCAGGATAGCAGTGT--TG-
TCTTCAGTTTTATGAGGTAAAGCGAATGATTAGGGACTCGGGGGCGCTATTTTGCCTTCATCCATTC
TCAAACTTTAAATATGTAAGAAGCCCTTGTTACTTAGTTGAACGTGGGCATTCGAATGTACCAACA
CTAGTGGGCCATTTTTGGTAAGCAGAACTGGCGATGCGGGATGAACCGAACGCGGGGTTAAGGTG
CCAGAGTGGACGCTCATCAGACACCACAAAAGGTGTTAGTACATCTTGACAGCAGGACGGTGGCC
ATGGAAGTCGGAATCCGCTAAGGACTGTGTAACAACTCACCTGCCGAATGTACTAGCCCTGAAAAT
GGATGGCGCTCAAGCGTCT-
CACCCATACCTCGCCCTTAAGGTAGAAACGATGCCTTAAGGGCGATGTATCGATCAGAACAAGCGC
TTCCAAATCGAGCTGGCTGCTAAGCCTAGTATAATCACGAATGGTCTAAAGTACTCTCTTGCTACG
GGTAACTGGGGTGACCAGAAGAAGGCAATGAGTTCGACGGCTGGCGTGTCGCAGGTCCTGAATCG
ATACACGT------TTGCGTCTA---
CTCTCTCTCACTTGAGACGGACAAATACACCTATTGGCAGAGATGGGAAGCTTGCGAAGCCCCGGC
AGCTGCATAATACCCACTGGGGTCTGGTATGTCCTGCTGAGACGCCTGAAGGCCAAGCCTGCGGGT
TGGTAAAGAATTTGTCTCTCATGTGCTCTATTAGCGTGGGTACATCGACGGATCCCATCGTAGACTA
CATGATTACTAGAAATATGGAAGTCTTGGAGGAATACGAACCGATGCGCTACCCCAACGCTACTAA
GATCTTTCTCAATGGTTCTTGGATTGGTGTACACCAGGACCCCAAGGCTCTGGTTCGAGACGTGCA
GCAACTTCGCCGGGCTAACCAGATCCCCTTCGAAGTATCGTTAGTTCGCGACATTCGTGATCGTGA
ATTCAAGATCTTCTCGGATGCTGGCCGCGTTATGCGGCCCTTGTTCGTTGTACAGCAAGAGGAT------
----

AATCCCACGACCGGTGCTCCTAAAGGCTCACTAGCCCTTAACAAGGACATGATCCAGAGACTGGAG

GCCGATGTCGATCTGGACCCCGAGAGTGAGGAGTACTTCGGCTGGCAGGGCCTAGTTAACGAAGG
TGCCATCGAATATCTCGATGCGGAAGAAGAGGAAACAGCTATGATTTGCATGACTCCTGAGGACTT
GGAAAACTACCGGATGACCAAACTTGGAATTGATATGTCTCAAGATAATGGA------
GACGAGATTAACAAACGGCTCAAAACCAAGGTTAATCCTACGACGCACATGTATACACATTGCGA
GATTCATCCCAGTATGCTCCTGGGTATCTGCGCGAGTATTATTCCATTTCCCGACCATAATCAGGTA
CCCCTGAACGCGTCCCTCAAACCCCTGATCTTCTGCCCCTCATGTACACAAACCGCCACTACAACGT

CATCTCGTGTTATATCATCACCAGATTATACAGCAATAAAAAAAAACATCTAAACTAACCGCGTT---
--TTCTCAATAGGTTCATCTTCAGACTGGCCAATGTGTAAGTGAA---------AAGGACGATCGATAAA----
-
GTGAACAAGTATAGCGGGGCTCACGAACAACGTATAGGGTAACCAAATTGGTGCCGCCTTCTGGC
AAACCATCTCCAGCGAGCACGGCCTCGACAGCAATGGCGTGTATGTATTTGAGTCGTCAATTGTAC
TACCAAAGAAGTCAACTAACCGCCAATAAATAGCTACAATGGAACCTCCGAGCTCCAGCTTGAGC
GCATGAGCGTCTACTTCAACGAGGTACGAGTTTATAAGAGCTGGTTGCGTGTAAATATAACCAAAA
GCTAATCGCCTCTACGTATATAGGCTTCTGGCAACAAGTATGTCCCTCGCGCCGTTCTCGTCGATCT
CGAGCCCGGTACCATGGATGCCGTCCGTGCTGGTCCCTTTGGTCAGCTTTTCCGACCCGACAACTTT
GTCTTCGGTCAGTCCGGTGCTGGCAACAACTGGGCCAAGGGTCACTACACTGAGGGTGCCGAGTTA
GTCGACAACGTCCTCGATGTCGTTCGTCGTGAGGCTGAAGGCTGTGACTGCCTCCAGGGTTTCCAG
ATCACCCACTCTCTCGGTGGTGGTACTGGTGCTGGTATGGGTACTCTGTTGATCTCCAAGATCCGCG
AAGAGTTCCCCGACCGCATGATGGCTACCTTCTCCGTCATGCCCTCGCCTAAGGTCTCTGATACCGT
CGTCGAGCCCTATAACGCCACCCTCTCCGTCCACCAGCTCGTCGAGAACTCCGACGAGACCTTCTG
TATCGACAACGAGGCCCTTTACGATATCTGCATGCGTACTCTCAAGTTATCTAACCCTTCGTATGGC
GATCTTAACCACCTCGTCTCCGCCGTCATGTCCGGCGTTACCACTTGCTTGCGTTTCCCCGGTCAGC
TAAACTCTGACCTTCGCAAGCTCGCCGTGAACATGGTTCCTTTCCCTCGTCTACACTTCTTCATGGTT
GGCTTCGCACCTCTTACCAGCCGTGGCGCTCACTCTTTCCGCGCTGTTACCGTTCCCGAGTTGACCC
AGCAGATGTTCGACCCCAAGAACATGATGGCTGCTTCCGACTTCCGTAACGGTCGTTACCTGACTT
GCTCTGCCATCTTGTAAGATGATACCGCTCCTAAATTGCTCGTCGTCAGTTTGCTAACCTCGTTTTCC
CAGCCGTGGCAAGGTCTCGA

>Hypoxylon_samuelsii

-
CATTACAGAGTTACTAAAACTCCCAACCCTTTGTGAACCTTACCGTCGTTTCCTCGGCGGCGAGCGC
--
GAATTCAGAAGCTACCCTGGAGCCACCTACCCTGTAGGTGGCTACCCTGGAGCTACCGTATGAGTG
CGC-
GCCGGCCCCGTCGATGGACCACCAAACTCTTTTTAATACTACGTGTCTCTGAACAACCGAAACAAA
TTCGTTAAAACTTTCAACAACGGATCTCTTGGTTCTGGCATCGATGAAGAACGCAGCGAAATGCGA
TAAGTAATGTGAATTGCAGAATTCAGTGAATCATCGAATCTTTGAACGCATATTGCGCCCATTAGT
ATTCTAGTGGGCATGCCTATTCGAGCGTCATTTCAACCCTTAAGCCTCTGTTGCTTAGCGTTAGGAA
TCTGCGTCGAGAGACGCAGCTCCCTAAAGGTAGTGGCGGTGTCGGGTACACTTCGTAGCGCAGTAA
CTTTTCTCGCTTCTGCAGTGGCTGGAAAGACTTACCGTAAAA-----AAAA----TAGTGACTATTT---------
---------------------------TGAAGCGGCAACAGCTCAAA-TTTGAAATCTGGCC----CTC-----
GCGGTCCGAGTTGTAATTTGTAGAGGATGCTTTTGGTGCGGT-
GCCTTCCGAGTTCCCTGGAACGGGACGCCGGAGAGGGTGAGAGCCCCGTACGGTTGGA-
CACCTATCCTCTATATAGCTCCTTCAACGAGTCGAGTAGTTTGGGAATGCTGCTCTAAATGGGAGGT
AAATTTCTTCTAAAGCTAAATACCGGCCAGAGACC-
GATAGCGCACAAGTAGAGTGATCGAAAGATGAAAAGCACTTTGAAAAGAGGGTTAAATAGCACGT
GAAATTGTTGAAAGGGAAGCG-TTTGCGACCAGACCTTCTCCAGGGGGATCATCCGGCGTTC--
TCGCCGGTGCACTTCGCCTGG--TTTAGGCCAGCATCGGTTTT---
CGTAGGGGGAAAAAGTCTTGGGGCACGTAGCTC----TTCCGGGAGTGTT-
ATAGCCCCTCGTGTAATACCCTTCC-GTGGACCGAGGACCGCGCC-
TTCTCGGCAAGGATGCTGGCGTAATGGTCGTCAACGACCCGTCTTGAAACACGGACCAAGGAGTCG
AACATTTGTGCGAGTGTTTGGGTG--
TTAAACCCTCACGCGTAATGAAAGTGAACGGAGGTGAGAGCCTTT-----CG---
GGGCGCATCATCGACCGATCCTGATG-
TCTTCGGATGGATTTGAGTAAGAGCATAACTGTTCGGACCCGAAAGATGGTGAACTATGCGTGGAT
AGGGTGAAGCCAGAGGAAACTCTGGTGGAGGCTCGCAGCGGTTCTGACGTGCAAATCGATCGTCA

AATCTGCGCATGGGGGCGAAAGACTTATCGA-
ACCATCTAGTAGCTGGTTACCGCCGAAGTTTCCCTCAGGATAGCAGTGT--TG-
TTTTCAGTTTTATGAGGTAAAGCGAATGATTAGGGACTCGGGGGCGCTTTTTGCCTTCATCCATTC
TCAAACTTTAAATATGTAAGAAGCCCTTGTTACTTAGCTGAACGTGGGCATTCGAATGTATCAACA
CTAGTGGGCCATTTTTGGTAAGCAGAACTGGCGATGCGGGATGAACCGAACGCGGGGTTAAGGTG
CCAGAGTGGACGCTCATCAGACACCACAAAAGGTGTTAGTACATCCAGACAGC-
GGACGGTGGCCATGGAAGTCGGAATCCGCTAAGGACTGTGTAACAACTCACCTGCCGAATGTACTA
GCCCTGAAAATGGATGGCGCTCAAGCGTCT-CACCCATACCTCGCC-------------------------
GCGCTGCATCGAGTCGAACAAACATTTCGAAATCGCCCTGGCTGCCAAACCAGCGATTATCACGAA
TGGTCTGAAATACTCGCTCGCCACCGGAAATTGGGGCGACCAGAAGAAGGCGATGAGTTCGACAG
CTGGCGTGTCTCAGGTCCTGAACCGATACACGT------TCGCTTCTA---
CGCTTTCCCACTTGAGAAGAACCAATACGCCTATCGGCCGAGACGGGAAGCTCGCGAAACCCCGG
CAGCTTCACAATACCCATTGGGGTCTCGTCTGCCCGGCCGAAACACCTGAGGGTCAGGCCTGTGGT
CTAGTGAAGAACTTATCGCTGATGTGTTCGATCAGCGTGGGCACGTCTACGGATCCTATAGTAGAT
TATATGATCACTAGGAATATGGAGGTTCTGGAGGAATACGAACCCTTACGATACCCTGATGCTACC
AAGATCTTCCTCAACGGCTCTTGGATCGGTGTACACCAGAACCCCAAGGCTCTAGTAAGAGACGTC
CAGAATCTGCGCCGGACAAATCAGATTCCGGCCGAGGTGTCGCTGATCCGTGACATACGCGATCGC
GAATTCAAGATCTTTTCGGATGCTGGCCGCGTCATGCGCCCCTTGTTTGTTGTGAATCAAGAGGAT--
----
ACCGAGGCAGGCATTAAAAAGGGAACCTTAGCTCTCACTAAGGAGATGATTCAGAGACTTGAAGC
AGATGTCGACCTGGATCCTGAAAGCGAGGAATACTTTGGCTGGCAAGGCCTGGTCAATGAAGGTGT
CATTGATTACCTCGATGCAGAAGAAGAAGAGACAGCCATGATCTGCATGACACCCGAGGACCTAG
AGACCTATCGCCAGACCAAGCTCGGCTACAATGTGTCTCAGGATAACGGG------
GACGAGATTAATAAGCGACTGAGGACTAAGGTTAACCCGACTACCCATATGTATACCCACTGCGAG
ATCCATCCTAGCATGCTCTTAGGTATATGCGCGAGCATTATCCCGTTCCCGGATCATAATCAGGTAC
CCCTGAACGCGTCCCCCAACCCCTTGATCTACTGCCCCTCACCTACAAACACCACAAACACAATAC
CTCGAGATATTACATCTCGCC-----------ACTC--TCCG---
CTATAGAAAATGAAAGCTAATCGTATCTCTTCATCTCGATAGGTTCACCTTCAGACCGGCCAATGC
GTAAGAAATACCCTTACATCTAACTATCGACTA-
TTGACGCGAAGGAATATTGCGGGGCTTACAATACTTTTGTAGGGTAACCAAATTGGTGCCGCTTTC
TGGCAGACCATCTCTGGCGAGCACGGTCTCGACAGCAATGGCGTGTACGTGCTTCATCGGTCAATT
CCAAGAT-
GAGTACAAAAACTGACAGCTCGTTACTAGCTACAACGGAACCTCTGAGCTCCAGCTGGAACGCATG
AGCGTCTACTTCAACGAGGTACGCCACAGTAGAGTCCAGGAATACATGCGCGAGTCCTGTCACTAA
TTATTTCTTCGTGCGTAGGCTTCCGGCAACAAGTATGTTCCCCGTGCCGTCCTCGTCGATCTCGAGC
CTGGTACCATGGATGCCGTCCGTGCTGGCCCCTTCGGACAGCTCTTCCGACCGGACAACTTCGTCTT
TGGTCAGTCCGGTGCTGGCAACAACTGGGCCAAGGGTCACTACACTGAGGGTGCCGAGCTCGTTGA
CAATGTCTTGGATGTTGTTCGTCGCGAGGCTGAAGGCTGCGACTGCCTTCAGGGTTTTCAGATCACC
CACTCGCTCGGTGGCGGTACTGGTGCCGGTATGGGTACCTTGCTGATCTCCAAGATCCGCGAGGAG
TTCCCTGACCGCATGATGGCCACCTTCTCCGTTGTTCCTTCCCCCAAGGTCTCGGATACCGTTGTAG
AGCCTTACAACGCCACCCTCTCCGTCCACCAGCTGGTCGAGAACTCGGACGAGACTTTCTGCATTG
ACAACGAGGCTTTGTACGACATCTGCATGCGTACCTTGAAGCTATCCAACCCCTCGTATGGTGATCT
GAACCACCTAGTCTCCGCCGTCATGTCTGGCGTCACCACCTGCTTGCGATTCCCTGGTCAGCTGAAC
TCTGACCTCCGTAAGCTGGCTGTCAACATGGTGCCCTTCCCTCGTCTGCACTTCTTCATGGTTGGCTT
CGCGCCCCTGACCAGCCGTGGTGCTTACTCGTTCCGTGCCGTCACCGTTCCCGAGTTGACTCAGCAG
ATGTTCGACCCCAAGAACATGATGGCTGCCTCTGACTTCCGCAATGGTCGCTACCTGACGTGCTCC
GCCATCTTGTAAGACTGCCGCCCTAAGTAGTCGATTCGTGCTTGCTTGCTGACCTTAA--
TTCTAGCCGTGGTAAGATCTCCA

>Hypoxylon_munkii_MUCL53315

-TGTTACCGCG-------------TAACCCTTTGTGAACCTTACCGTCGTTGCCTCGGCGGCGAGCTC--
AAATCCGAAGGCTACCCTGGAGCCACTTACCCTGTAGGTGGCTACCCTGGAGCTACCCCGGAGCTA
CGTCATCGGCCCCGTCGAAGGACCGTTAAATTCTT-T---------
TCACGTCTGAACTATTAAAAAAAAATCGTTAAAACTTTCAGCAACGGATCTCTTGGTTCTGGCATC
GATGAAGAACGCAGCGAAATGCGATAAGTAATGTGAATTGCAGAATTCAGTGAATCATCGAATCT
TTGAACGCATATTGCGCCCATTAGTATTCTAGTGGGCATGCCTATTCGAGCGTCATTTCAACCCTTA
AGCCCCTGTTGCTTAGCGTTAGGAATCTGCGTC--

GCGGCGCAGTTCCCCAAAGGTAGTGGCAGTGTAG-
GGCACACTCGTAGCGTAGTAGATTATCTCGCTTCCGACGTGGTCCGAACGACTCGC--------------------
--TGAAGCGGCAACAGCTCAAA-TTTGAAATCTGGCC----CTC-----
GCGGTCCGAGTTGTAATTTGTAGAGGATGCTTTTGGTGCGGT-
GCCTTCCGAGTTCCCTGGAACGGGACGCCGGAGAGGGTGAGAGCCCCGTACGGTTGGA-
CACCTATCCTATATGTAGCTCCTTCGACGAGTCGAGTAGTTTGGGAATGCTGCTCTAAATGGGAGG
TAAATTTCTTCTAAAGCTAAATACCGGCCAGAGACC-
GATAGCGCACAAGTAGAGTGATCGAAAGATGAAAAGCACTTTGAAAAGAGGGTTAAATAGCACGT
GAAATTGCTGAAAGGGAAGCG-TTTGCGACCAGACCTCCTCCGGGCGGATCATCCGGTGTTT--
TCACCGGTGCACTTCGCCCGG--TCTAGGCCAGCATCGGTTTT---
CGTAGGGGGATAAAGGCCCGGGGCACGTAGCTC----TTCCGGGAGTGTT-
ATAGCCCCCCGCGTAATGCCCTTAC-GGGGACCGAGGACCGCGCT-CTT---
GCAAGGATGCTGGCGTAATGGTCGTCAACGACCCGTCTTGAAACACGGACCAAGGAGTCGAACAT
TTGTGCGAGTGTTTGGGTG--TTAAACCCTCACGCGTAATGAAAGTGAACGGAGGTGAGAGCCCTT--
---CG---GGGTGCATCATCGACCGATCCTGATG-
TCTTCGGATGGATTTGAGTAAGAGCATAACTGTTCGGACCCGAAAGATGGTGAACTATGCGTGGAT
AGGGTGAAGCCAGAGGAAACTCTGGTGGAGGCTCGCAGCGGTTCTGACGTGCAAATCGATCGTCA
AATCTGCGCATGGGGGCGAAAGACTTATCGA-
ACCATCTAGTAGCTGGTTACCGCCGAAGTTTCCCTCAGGATAGCAGTGT--TG-
TTTTCAGTTTTATGAGGTAAAGCGAATGATTAGGGACTCGGGGGCGCTTTTTAGCCTTCATCCATTC
TCAAACTTTAAATATGTAAGAAGCCCTTGTTACTTCGTTGAACGTGGGCATTCGAATGTATCAACAC
TAGTGGGCCATTTTTGGTAAGCAGAACTGGCGATGCGGGATGAACCGAACGCGGGGTTAAGGTGC
CAGAGTGGACGCTCATCAGACACCACAAAAGGTGTTAGTACATCCAGACAGC-
GGACGGTGGCCATGGAAGTCGGAATCCGCTAAGGACTGTGTAACAACTCACCTGCCGAATGTACTA
GCCCTGAAAATGGATGGCGCTCAAGCGTCT-
CACCCATACCTCGCCCTTAGGGTAGAAACGATGCCCTAAGGGCGATGCATCGAGTCGAACAAGCAT
TTCGAAATCGCCCTAGCTGCTAAACCGGCCATTATCACGAACGGCCTCAAGTACTCGCTCGCCACG
GGAAATTGGGGCGATCAGAAGAAGGCCATGAGTTCGACCGCTGGTGTGTCTCAGGTCCTGAATCG
ATACACGT------TCGCTTCTA---
CGCTTTCTCACTTGAGAAGAACCAACACGCCTATTGGTCGTGACGGTAAGCTCGCGAAACCCCGGC
AGCTTCACAATACCCATTGGGGCCTGGTCTGCCCGGCCGAAACGCCCGAGGGTCAGGCCTGTGGTC
TGGTGAAGAATTTGTCGTTGATGTGTTCGATCAGCGTGGGGACACCTACGGATCCTATAGTGGATT
ATATGATCACCAGGAATATGGAGGTTCTCGAGGAGTACGAACCCTTACGATACCCAGACGCTACTA
AGATCTTCCTCAACGGCTCTTGGATCGGTGTACACCAGAACCCCAAGGCTCTGGTAAGAGATGTCC
AGAATTTGCGCCGGACAAATCAGATCCCGGCCGAGGTATCGCTGATCCGCGACATACGCGATCGCG
AATTCAAGATCTTTTCAGATGCGGGCCGGGTCATGCGACCCTTGTTTGTCGTGAATCAGGAGGAC---
----
ACCGAGGCTGGTATCAAGAAGGGTACCTTAGCTCTAACTAAGGAGATGATCCAGAGGCTTGAATCC
GATGTCGATCTGGATCCCGATAGCGAGGAGTACTTCGGCTGGCAGGGCTTGGTCAACGAAGGTGTC
ATCGATTATCTCGACGCAGAGGAAGAAGAAACCGCTATGATCTGCATGACGCCCGAGGACCTAGA
AACCTACCGCCAGAACAAACTCGGATACAACGTGTCCCAGGATAATGGG------
GATGAGATTAATAAGCGCCTGAGGACCAAGGTTAACCCGACTACGCACATGTACACCCACTGCGA
GATCCATCCCAGCATGCTGCTGGGTATATGCGCGAGCATTATCCCGTTTCCGGATCACAATCAGGT
A---------GCGGCACCCAACCCCCTGATCTTCTACCCCTCACACATAAACACCACGAACACCAGAC-
TCGAGATACCACATCTCAT---------AGTTGTT--CGTC---
ATACAGAGAATGAAAGCTAATCATATCTATTCATCTTAATAGGTTCACCTTCANACCGGCCAATGC
GTAAGAACTACCTAGAACTCCAACCGTCGACTA-
TCGACGCGAAGGAATATTGCGGGGCTCACATGGATTTTGTAGGGTAACCAAATTGGTGCTGCTTTC
TGGCAGACCATCTCTGGCGAGCACGGTCTCGACAGCAATGGCGTGTACGTGTTACATTGGTCAATT
CCGTGACGAG---
TAAAAACTAATAGCTCATTAATAGTTACAACGGAACCTCTGAGCTCCAGCTAGAGCGCATGAGCGT
CTACTTCAACGAGGTATGCCACAACGAAGCCCAGAAATGTATGCGCAAGTCCCGTCACTAATTATC
CCTACGTGCGCAGGCTTCCGGCAACAAGTATGTTCCCCGTGCCGTCCTTGTCGATCTTGAGCCCGGT
ACCATGGATGCCGTCCGTGCTGGTCCCTTCGGTCAGCTCTTCCGACCCGACAACTTCGTCTTTGGTC
AGTCCGGTGCTGGCAACAACTGGGCCAAGGGTCACTACACGGAAGGTGCCGAACTCGTTGACAAT
GTCTTGGATGTCGTTCGTCGTGAGGCTGAGGGCTGCGACTGCCTTCAGGGCTTCCAGATCACTCACT
CGCTCGGTGGTGGTACCGGTGCCGGTATGGGTACTTTGTTGATCTCCAAGATCCGCGAGGAGTTCC

CTGACCGCATGATGGCCACCTTCTCCGTTGTCCCTTCCCCCAAGGTCTCGGATACCGTTGTCGAGCC
TTACAACGCCACCCTCTCCGTGCACCAGCTGGTTGAGAACTCGGATGAGACTTTCTGCATTGACAA
CGAGGCTCTGTACGACATCTGCATGCGTACTCTGAAGCTATCTAACCCCTCGTATGGTGACCTGAA
CCACTTGGTCTCTGCCGTCATGTCTGGCGTTACCACCTGCTTGCGATTCCCTGGTCAGCTGAACTCT
GACCTCCGTAAGCTCGCTGTCAACATGGTGCCCTTCCCCCGTCTGCACTTCTTCATGGTCGGTTTCG
CGCCCCTGACCAGCCGTGGCGCTTACTCGTTCCGTGCTGTCACCGTTCCCGAGTTGACTCAGCAAAT
GTTCGACCCCAAGAACATGATGGCTGCCTCTGACTTCCGCAACGGTCGTTACCTGACGTGCTCGGC
CATCTTGTAAGACTTTCCCTCTAAATAGTCGATTCGTACTTATTTGCTAACCTAGA---
TCTAGCCGTGGCAAGATCT---

>Hypoxylon_pulicicidum

-CATTAATGAGTTAC--AAACTCCAAACCCTTTGTGAACCTTACCGTCGTTTCCTCGGCGCG-
TGCGCACCTACCCTGTAGCTACCCTGCAGGCACCTACCCTGTAGTAGTCTACCCTGGAGCTACCCTG
TAGCCGCG--
TGAAGGCCCGCCGAAGGACCGTTAAACTCTTGCTTTACAACTGTATCTCTGAACACGTAACTGAAA
TAAGTTAAAACTTTCAACAACGGATCTCTTGGTTCTGGCATCGATGAAGAACGCAGCGAAATGCGA
TAAGTAATGTGAATTGCAGAATTCAGTGAATCATCGAATCTTTGAACGCACATTGCGCCCATTAGT
ATTCTAGTGGGCATGCCTATTCGAGCGTCATTTCAACCCTTAAG-
CCCTGTTGCTTAGTGTTGGGAGTCTACGGCTT-
CGGCGTAGTTCCTGAAAGTCAGTGGCGGAGTTAGGGTACACTCTCAGCGTAGTAAT--
CTCTCGCTCGTGTGGTGGCCCTGGCTGCTGGCCGTAAAACCCCCTAT-TTTCTAGT-----------------------
---------------------TGAAGCGGCAACAGCTCAAA-TTTGAAATCTGGCC----CTA-----
GTGGTCCGAATTGTAATTTGTAGAGGATGCTTTTGGTGCGGT-
GCCTTCCGAGTTCCCTGGAACGGGACGCCGGAGAGGGTGAGAGCCCCGTACGGTTGGA-
CACCTAGCCTCTGTATAGCTCCTTCGACGAGTCGAGTAGTTTGGGAATGCTGCTCTAAATGGGAGG
TAAATTTCTTCTAAAGCTAAATACCGGCCAGAGACC-
GATAGCGCACAAGTAGAGTGATCGAAAGATGAAAAGCACTTTGAAAAGAGGGTTAAATAGCACGT
GAAATTGTTGAAAGGGAAGCG-TTTGCGACCAGACCTTTTCCGGGCGGATCATCCGGCGTTC--
TCGCCGGTGCACTTCGTCCGG--TCTAGGCCAGCATCGGTTTC---
CTTAGGGGGATAAAGGCTTGGGGAACGTAGCTC----TTTAGGGAGTGTT-
ATAGCCCCTTGCGTAATACCCTTCG-GGGGACCGAGGACCGCGCT-CT----
GCAAGGATGCTGGCGTAATGGTCGTCAACGACCCGTCTTGAAACACGGACCAAGGAGTCGAACAT
TTGTGCGAGTGTTTGGGTG--TTAAACCCTTACGCGTAATGAAAGTGAACGGAGGTGAGAGCCCTT---
-AC----GGGTGCATCATCGACCGATCCTGATG-
TCTTCGGATGGATTTGAGTAAGAGCATAACTGTTCGGACCCGAAAGATGGTGAACTATGCGTGGAT
AGGGTGAAGCCAGAGGAAACTCTGGTGGAGGCTCGCAGCGGTTCTGACGTGCAAATCGATCGTCA
AATCTGCGCATGGGGGCGAAAGACTTATCGA-
ACCATCTAGTAGCTGGTTACCGCCGAAGTTTCCCTCAGGATAGCAGTGT--TG-
TCTTCAGTTTTATGAGGTAAAGCGAATGATTAGGGACTCGGGGGCGCTATATTGCCTTCATCCATTC
TCAAACTTTAAATATGTAAGAAGCCCTTGTTACTTAAGTGAACGTGGGCATTCGAATGTACCAACA
CTAGTGGGCCATTTTTGGTAAGCAGAACTGGCGATGCGGGATGAACCGAACGCGGGGTTAAGGTG
CCAGAGTGGACGCTCATCAGACACCACAAAAGGTGTTAGTACATCTNGACAGCAGGACTGTGGCC
ATGGAAGTCGGAATCAGCTAAGGACTGTGTAACAACTCACCTGCCGAATGTACTAGCCCTGAAAAT
GGATGGCGCTCAAGCGTCT-CACCCATACCTCGCCCTTAGGGTAGAAACGATGCCCTAAGG-----------
----
CCGACGTTTCCAAATCGAGCTTGCCGCCAAGCCCGCCATCATCACCAACGGTCTGAAGTACTCCCT
TGCCACAGGTAACTGGGGTGACCAGAAGAAGGCGATGAGCTCTACTGCTGGCGTGTCGCAGGTCTT
GAACAGATATACCT------TCGCGTCAA---
CTCTGTCTCACTTGAGGCGAACCAACACACCCATTGGAAGAGACGGGAAGCTTGCGAAGCCTCGAC
AGCTTCACAATACCCATTGGGGTCTCGTCTGTCCGGCTGAGACACCCGAAGGACAGGCCTGCGGGC
TGGTGAAGAATTTAGCGCTGATGTGCTCTATCAGTGTGGGTACATCGACGGATCCTATTGTAGACT
ATATGATTACGAGGAATATGGAAGTCTTGGAGGAATACGAACCGATGCGATACCCCAACGCCACC
AAGATCTTCCTCAATGGCTCTTGGATCGGTGTACATCAAGATCCCAAGACTCTTGTTAGAGATATCC
AGGCCCTTCGTCGAGCCAATCAGATCCCCTCTGAAGTTTCTCTGGTCCGCGATATCCGTGATCGTGA
GTTCAAGATCTTCTCAGATGCGGGTCGTGTCATGCGCCCCTTGTTTGTCGTACAACAAGAGGAT------
----
ATACCCGACCAGAATGTTACTAAGGGTACATTGGCTCTTACCAAAGAGATGATCCAGCGGCTGGAG

GCGGATGTTGATCTGGATCCTGAGAGCGAGGAGTACTTCGGCTGGCAAGGTCTGGTTAACGAGGGT
GTTATCGAGTTTCTCGACGCGGAAGAAGAGGAAACGGCTATGATTTGCATGACGCCGGAAGATTTG
GAAACTTATCGAATGACCAAGGCCGGTCTTGAAGTGGACCAGGACAACGGA------
GATGAAGTTAACAAACGGCTCAAGACTAAGGTGAACCCGTCGACACACATGTACACGCATTGCGA
AATCCACCCCAGTATGCTCCTAGGTATCTGCGCCAGC------------------------------
CCCTGAACGCGTCCACTCAACCCCTGATTTCTGCCCCTCACGCACACAAACC---
ACTGCAGCATCATGCGGTGGACAGGACTGCGATGTCATCAAACTGGACAACGACG-
AGGAAAGAATCACAGCTAAC----
GATGTTTGTTTCAATAGGTTCACCTCCAGACCGGCCAATGCGTAAGTAAAACGGT----
ACGAACCACGGGCGAAGCTACGCGCTGGAACATAGAGGGGCTTACACA--
AACTGCAGGGTAACCAAATCGGTGCTGCTTTCTGGCAAACCATCTCTGGCGAGCACGGCCTCGACA
GCAATGGCGTGTAAGTACATGAGCTGTCAATTCTGATGCCAAGAGTAGGAACTAACCACCGATAAT
TAGCTACAACGGAACCTCTGAGCTCCAGCTCGAGCGCATGAGCGTTTACTTCAACGAGGTATGCAG
GAGTCGAAGCCTCGAAGAAAGCCTCAAGGACAGCTACTAACTACTCTAACCTCTGCAGGCTTCCGG
CAACAAGTACGTGCCTCGCGCCGTCCTCGTCGATCTCGAGCCCGGTACCATGGATGCCGTCCGTGC
TGGTCCCTTCGGCCAGCTCTTCCGACCTGACAACTTCGTCTTCGGCCAGTCTGGTGCCGGAAACAAC
TGGGCCAAGGGTCACTACACTGAGGGTGCTGAGCTTGTTGACAACGTCCTTGATGTTGTCCGTCGT
GAGGCTGAGGGCTGCGACTGCCTCCAGGGTTTCCAGATCACCCACTCCCTCGGTGGTGGTACCGGT
GCCGGTATGGGTACTCTCCTGATCTCCAAGATCCGTGAGGAGTTCCCCGACCGCATGATGGCCACC
TTCTCCGTCGTGCCTTCTCCTAAGGTCTCTGACACCGTCGTCGAGCCCTACAACGCCACCCTCTCGG
TCCACCAGCTGGTCGAGAACTCCGACGAGACCTTCTGCATTGACAACGAGGCTCTGTACGACATCT
GCATGCGTACCCTGAAGCTATCCAACCCCTCGTACGGTGACCTGAACCACCTGGTCTCCGCTGTCAT
GTCCGGTGTCACCACCTGCTTGCGCTTCCCTGGCCAGCTGAACTCTGACCTCCGCAAGCTTGCCGTG
AACATGGTTCCTTTCCCTCGTCTCCACTTCTTCATGGTCGGCTTCGCCCCTCTGACCAGCCGTGGCG
CTCACTCCTTCCGCGCTGTCACCGTTCCCGAGTTGACTCAGCAGATGTTCGACCCCAAGAACATGAT
GGCTGCTTCCGACTTCCGTAACGGCCGCTACCTGACTTGCTCTGCCATCTTGTAAGATATCCTTCGC
GAATTGTCCGCTATTGCTAGTTTGCTAACTTGTGTTTTCCAGCCGTGGCAAGGTCTCCA

>Hypoxylon_olivaceopigmentum

-CATTAACGAGTTAACAAAACTCCCAACCCTTTGTGAACCTTACCACAGTTTCCTCGGCGCA-
AGCCTGGCTACCCCGTAGCTACCCTGAATCTATCTACCCTGTAGGAAGCTACCCTGTAGCTACCCTG
TATCTACGCTCCAGCGCCCGCCGGTGGACCGCTAAACTCTTGTATACTGATGTGGAATCTGAATTAT
CAACTGAAATAAGTTAAAACTTTCAACAACGGATCTCTTGGTTCTGGCATCGATGAAGAACGCAGC
GAAATGCGATAAGTAATGTGAATTGCAGAATTCAGTGAATCATCGAATCTTTGAACGCATATTGCG
CCCACTAGCATTCTGGTGGGCATGCCTATTCGAGCGTCATTTCGACCCCTAAGCCCCTGTTGCTTAG
CGTTGAGGATCTACGGCTTCTGGCGTAGTCCTCTAAAGTTAGTGGCGGAGCCGCGGCGTACTCTGA
GCGTAGTAATTCTCCTCGCTTTTGCTGTACCCGTAGCTGTCGGCCGTAAAACCCCTTAAATTTCAAG
TGGTTGACCTCGGATTAGGTAGGAATACCCGCTGAACTTAAGC--
--------CGAGTTGTAATTTGTAGAGGATGCTTTTGGTGCGGC-
GCCTTCCGAGTTCCCTGGAACGGGACGCCGGAGAGGGTGAGAGCCCCGTACGGTTGGA-
CGCCTAGCCTATATATAGCTCCTTCGACGAGTCGAGTAGTTTGGGAATGCTGCTCTAAATGGGAGG
TAAATTTCTTCTAAAGCTAAATACCGGCCAGAGACC-
GATAGCGCACAAGTAGAGTGATCGAAAGATGAAAAGCACTTTGAAAAGAGGGTTAAATAGCACGT
GAAATTGTTGAAAGGGAAGCG-TTTGCGACCAGACCTTTTCCAGGCGGATCATCCGGTATTC--
TTACCGGTGCACTTCGCCTGG--TCTAGGCCAGCATCGGTTTC---
CTTAGGGGGATAAAGGCCGGGGGAACGTAGCTC-----TTCTGGAGTGTT-
ATAGCCCCCGGTGTAATACCCCTCG-GGGGACCGAGGACCGCGCA-TCT---
GCAAGGATGCTGGCGTAATGGTCGTCAACGACCCGTCTTGAAACACGGACCAAGGAGTCGAACAT
TTGTGCGAGTGTTTGGGTG--TTAAACCCTCACGCGTAATGAAAGTGAACGGAGGTGAGAGCCCTT--
--AC----GGGCGCATCATCGACCGATCCTGATG-
TCTTCGGATGGATTTGAGTAAGAGCATAACTGTTCGGACCCGAAAGATGGTGAACTATGCGTGGAT
AGGGTGAAGCCAGAGGAAACTCTGGTGGAGGCTCGCAGCGGTTCTGACGTGCAAATCGATCGTCA
AATCTGCGCATGGGGGCGAAAGACTAATCGA-
ACCATCTAGTAGCTGGTTACCGCCGAAGTTTCCCTCAGGATAGCAGTGT--TG-
TCTTCAGTTTTATGAGGTAAAGCGAATGATTAGGGACTC--

ACGTTGTATTGAGACGAATAGACGCTTCCAGATTGAGCTTGCCGCCAAGCCTGCCATCATCACTAA
CGGCTTGAAATACTCCCTTGCCACCGGTAACTGGGGCGACCAGAAGAAGGCAATGAGCTCGACTG
CCGGTGTGTCACAGGTTCTGAACCGATACACCT------TCTCGTCGA---
CTCTCTCTCACTTGCGACGGACAAACACCCCCATTGGAAGGGATGGAAAGCTGGCGAAGCCCCGAC
AGCTTCATAATACTCATTGGGGTCTGGTCTGTCCGGCTGAGACGCCTGAAGGCCAGGCCTGCGGGT
TGGTGAAGAACTTGTCGCTGATGTGCTCCATCAGCGTGGGTACATCGACGGATCCTATCGTGGACT
ATATGATTACTAGGAATATGGAAGTCCTGGAGGAATACGAACCGATGCGATACCCTAACGCTACCA
AGATCTTCCTCAATGGCTCTTGGATTGGTGTGCATCAAGATCCTAAGACTCTCGTCAGAGATATCCA
AGCACTTCGACGCGCCAACCAGATCCCTTCGGAGGTGTCGTTGATTCGAGATATTCGTGACCGAGA
GTTTAAGATCTTCTCGGATGCGGGTCGTGTGATGCGGCCTTTGTTTGTCGTACAGCAGGAGGAT------
----
AACCCGGAGAAAGGCGTTACTAAGGGCACATTGGCTTTGACCAAAGAGATGATACAGCGACTAGA
GGCTGATGTTGATCTGGACCCGGAGAGCGAGGAGTACTTCGGCTGGCAAGGCCTCGTCAACGAGG
GAGTTATCGAATTCCTTGATGCCGAGGAAGAGGAAACGGCCATGATTTGCATGACCCCCGAAGACT
TGGAGACCTTTCGTGCGACCAAGGCCGGGGAGCCAGTTGCTCAGGACAACGGG------
GATGAAGTCAACAAGCGACTTAAGACCAAGGTGAACCCATCGACGCACATGTATACGCATTGCGA
AATTCATCCGAGTATGCTCCTCGGCATCTGCGCCAGTATC----------------------------
CTGAACGCGTCCCTCAAACCCCTGATTTTCTGCCCCTCACGCACACACAAC---
GGCACAGCATCCCTCGATTCTCCCTGACAGCCCATCATCAACTCATGCAGGAATG-
AGATGGTAAATCAAGCTAACCGCGTTATTACTTGCCTATAGGTTCACCTTCAGACCGGCCAGTGCG
TAAGTACTACGAT----
GACAACCACCGACGAAACGTCGCGATAGAACTTGGAGGGGCTCACATGAATATCATAGGGTAACC
AAATTGGTGCTGCTTTCTGGCAGACCATCTCTGGCGAGCACGGTCTCGACAGCAATGGCGTGTATG
TATATGAGTTGTCAATTTTGATGCCAAGAATCCCAACTGACCACTGATACATAGCTACAACGGAAC
CTCTGAGCTCCAGCTCGAGCGCATGAGCGTCTACTTCAACGAGGTACGCAGCAATCGAAGCCAGGA
AGAGTCATCTAGGAGTAGCCACTAATCA---
TAATATGCATAGGCTTCCGGCAACAAGTATGTTCCTCGTGCCGTTCTCGTCGATCTCGAGCCCGGTA
CCATGGATGCCGTCCGCGCTGGTCCTTTCGGCCAGCTTTTCCGACCTGACAACTTCGTCTTCGGCCA
GTCGGGTGCCGGCAACAACTGGGCCAAGGGTCACTACACTGAGGGTGCTGAGCTTGTTGACCAGGT
CCTTGACGTTGTTCGTCGTGAGGCTGAGGGCTGCGACTGCCTCCAGGGTTTCCAGATCACCCACTCT
CTCGGTGGTGGTACCGGTGCTGGTATGGGTACTCTTCTGATCTCCAAGATCCGTGAGGAGTTCCCCG
ACCGCATGATGGCTACCTTCTCCGTCGTGCCCTCCCCCAAGGTCTCCGACACCGTCGTCGAGCCTTA
CAACGCCACCCTCTCCGTCCACCAGCTGGTCGAGAACTCGGATGAGACCTTCTGCATTGACAACGA
GGCTCTGTACGACATCTGCATGCGCACCCTGAAGCTATCCAACCCCTCGTACGGTGACCTGAACCA
CCTGGTCTCGGCTGTCATGTCTGGTGTCACCACTTGCTTGCGTTTCCCCGGCCAGCTGAACTCTGAC
CTTCGCAAGCTCGCCGTGAACATGGTTCCCTTCCCTCGTCTCCACTTCTTCATGGTCGGCTTCGCCCC
TCTGACCAGCCGTGGCGCGCACTCCTTCCGCGCCGTCACCGTTCCCGAGCTCACTCAGCAGATGTTC
GACCCCAAGAACATGATGGCTGCCTCTGACTTCCGCAACGGTCGCTACCTGACCTGCTCTGCCATCT
TGTAAGATGATCCTCTTGACT-
ATTTATGAATGCTATGTTACTAACTTGGTTCCTCTAGCCGTGGTAAGGTCTCCA

>Pyrenopolyporus_hunteri

------------------------AACCCTTTGTGAACCTTACCGTCGTTGCCTCGGCGTG-
AGCGCAGCTACCCTGCGGCTACCCCGGGGCAGCTTGCTCCAAAAAAAGCTAACCTAAAGCTGCTCC
G-AAATATGCTCCAAAGCTCGAAATGAAGCTCC-AAACTTTTTT---------
TTTTTTCTGAATTACTTAATTAAATCAGTTAAAACTTTCAACAACGGATCTCTTGGTTCTGGCATCG
ATGAAGAACGCAGCGAAATGCGATAAGTAATGTGAATTGCAGAATTCAGTGAATCATCGAATCTTT
GAACGCACATTGCGCCCATTAGTATTCTAGTGGGCATGCCTATTCGAGCGTCATTTCGACCCTTAAG
CCCTCGTTGCTTAGCGTTGGGAGCCTGCGTCCCGGGGCGCAGTTCCTCAAAGTTAGTGGCGGAGCT
AGGGCACACTCTAAGCGTAGTAAGTATTCTCGCTTCTGTGGTGTACCTGGCTTCCTGCCGTAAAACC
CC--
-GCTTTGGGCGCGGC-
GCCTTCCAAGTTCCCTAGAACGGGACGCCTTAGAGGGTGAGAGCCCCGTACGGTTGGA-
CGCCTAGCCTATGTATAGCTCCTTCGACGAGTCGAGTAGTTTGGGAATGCTGCTCTAAATGGGAGG
TAAATTTCTTCTAAAGCTAAATACCGGCCAGAGACC-
GATAGCGCACAAGTAGAGTGATCGAAAGATGAAAAGTACTTTGAAAAGAGGGTTAAATAGCACGT

GAAATTGTTGAAAGGGAAGCG-TTTGCGACCAGACCTTTTCCAGGCGGATCATCCGGCGTTC--
TCGCCGGTGCACTTCGCCTGG--TTTAGGCCAGCATCGGTTTT---
CCCAGGGGGATAAAGGCGGTGGGAACGTAGCTC----TTTCGGGAGTGTT-
ATAGCCCGCCGCGTAATACCCTTGG-GGGGACCGAGGACCGCGCT-TCG---
GCAAGGATGCTGGCGTAATGGTCGTCAACGACCCGTCTTGAAACACGGACCAAGGAGTCGAACAT
TTGTGCGAGTGTTTGGGTG--TCAAACCCTCACGCGTAATGAAAGTGAACGGAGGTGAGAGCCCCT--
---CG--CGGGTGCATCATCGACCGATCCTGATG-
TCTTCGGATGGATTTGAGTAAGAGCATAACTGTTCGGACCCGAAAGATGGTGAACTATGCGTGGAT
AGGGTGAAGCCAGAGGAAACTCTGGTGGAGGCTCGCAGCGGTTCTGACGTGCAAATCGATCGTCA
AATCTGCGCATGGGGGCGAAAGACTTATCGA-
ACCATCTAGTAGCTGGTTACCGCCGAAGTTTCCCTCAGGATAGCAGTGT--TG-
TCTTCAGTTTTATGAGGTAAAGCGAATGATTAGGGACTCGGGGGCGCTATTTTGCCTTCATCCATTC
TCAAACTTTAAATATGTAAGAAGCCCTTGTTACTTAGTTGAACGTGGGCATTCGAATGTACCAACA
CTAGTGGGCCATTTTTGGTAAGCAGAACTGGCGATGCGGGATGAACCGAACGCGGGGTTAAGGTG
CCAGAGTGGACGCTCATCAGACACCACAAAAGGTGTTAGTACATCTTGACAGCAGGACGGTGGCC
ATGGAAGTCGGAATCCGCTAAGGACTGTGTAACAACTCACCTGCCGAATGTACTAGCCCTGAAAAT
GGATGGCGCTCAAGCGTCT-CACCCATACCTCGCC---
AACAGACGCTTCCAAATCGAGCTTGCTGCCAAGCCGGCCATAATCACCAATGGTTTGAAATATTCT
CTAGCCACAGGCAACTGGGGCGACCAGAAGAAAGCCATGAGCTCCACGGCCGGCGTGTCGCAGGT
CCTAAACAGATATACGT------TCGCCTCGA---
CCCTTTCCCATTTACGACGAACGAACACGCCCATCGGAAGGGACGGCAAGCTCGCGAAGCCGCGA
CAGCTACACAACACTCATTGGGGTCTGGTATGTCCGGCCGAGACGCCCGAAGGCCAAGCCTGCGG
GCTGGTCAAGAATCTGTCGCTTATGTGCTCCATCAGCGTGGGTACCTCAACGGATCCTATCGTAGAT
TATATGATCACCAGAAACATGGAGGTCTTGGAGGAATATGAGCCCATGAGATATCCTAACGCAACC
AAGATCTTCCTCAACGGCTCCTGGATCGGTGTGCACCAAGATCCCAAGTCTCTAGTTAGAGACGTT
CAGCAGCTGCGCCGGGCCAACCAGATTCCCTCCGAGGTATCTTTAGTTCGCGACATCCGAGACCGC
GAGTTCAAGATTTTCTCAGACGCCGGCCGCGTCATGCGTCCCTTGTTTGTTGTACAGCAAGAGGAT--
----
GACCCGGACACCGGTGTCCCCAAGGGCCACCTGGCTCTCACGAAGACCCAGATTGCGAAGCTGGA
GGCAAGCATCGACGTAGAGGTCGACGCTCCCGGCTACTATGGCTGGCAAGGGTTAGTTAACGACG
GTGTTATCGAGTATCTCGATGCGGAGGAGGAGGAGACGGCTATGATATGCATGACGCCCGAAGAC
TTGGAAACATATCGCATGGCCAAGGCCGGCATTGATATGCCTCAGGACAACGGG------
GACGAGATCAACAAGCGCCTCAAGACCAAGGTTAACCCCACGACGCACATGTACACGCACTGCGA
GATCCACCCGAGTATGCTTCTAGGTATTTGCGCTAGCATTATTCCCTTCCCAGACCATAATCAGGTA
CCCCTGAACGCGTCG--------------
TGAATGCCCCTCACGCATCCAGAGCTGAACCACATCATCCTATGGTTCTCTGGAACACACTACCAC
GAAAAAACACGACTATTTGACGAATATTTGGAGCTAACCATATCTCTTG-
TCTTTATAGGTTCACCTTCAGACCGGCCAATGCGTAAGTACC----------
ATAATCGCCAACGAGACATAGCACTGGGACGTAGCGGGGCTCATACGAAGATCGTAGGGTAACCA
AATCGGTGCTGCTTTCTGGCAAACCATCTCGAGCGAGCACGGCCTCGACAGCAATGGCGTGTACGT
ATTTGAGTCGTCAACACTACCGCGAAGAGGTCCAACTAATCACCAATAAACAGCTACAACGGCACC
TCCGAGCTCCAGCTCGAGCGCATGAGCGTCTACTTCAACGAGGTACGGATTCGCGAAAGCA-----
GAAATGGATGGAGCTAGTTACTGATCGTATCACCGCATGCAGGCCTCCGGCAACAAGTATGTTCCT
CGCGCTGTCCTCGTCGATCTCGAGCCCGGCACCATGGACGCCGTCCGTGCCGGTCCCTTCGGTCAG
CTCTTCCGACCGGACAACTTCGTCTTCGGTCAGTCCGGTGCTGGCAACAACTGGGCCAAGGGTCAC
TACACTGAGGGTGCTGAGCTGGTTGACAACGTCCTCGACGTCGTCCGCCGTGAGGCCGAAGGCTGC
GACTGCCTCCAGGGCTTCCAGATCACCCACTCCCTCGGTGGTGGTACCGGTGCCGGTATGGGTACC
CTGTTGATCTCCAAGATCCGCGAGGAGTTCCCCGACCGCATGATGGCCACCTTCTCCGTCATGCCTT
CGCCCAAGGTCTCCGACACGGTCGTCGAGCCCTACAACGCCACTCTCTCCGTCCACCAGCTGGTCG
AGAACTCTGACGAGACCTTCTGTATCGACAACGAGGCTCTGTACGATATCTGCATGCGTACGCTGA
AGTTGTCCAACCCCTCGTACGGCGATCTGAACCACCTGGTCTCCGCCGTCATGTCTGGCGTCACCAC
TTGTCTGCGTTTCCCCGGCCAGCTGAACTCTGACCTGCGCAAGCTCGCCGTGAACATGGTTCCTTTC
CCCCGTCTCCACTTCTTCATGGTCGGCTTCGCCCCTCTGACCAGCCGCGGCGCTCACTCCTTCCGCG
CCGTCACCGTTCCCGAGTTGACTCAGCAGATGTTCGACCCCAAGAACATGATGGCTGCCTCCGACT
TCCGCAACGGTCGCTACCTGACGTGCTCTGCCATCTTGTAAGATATCCCCCTAAATCA-
TTTGTTAGTATCGGCCTGCTGACCTAAATCCCGTAGCCGTGGCAAGGTCTCCA

>Pyrenopolyporus_nicaraguensis

--ATTACTGAGTTGTCAAAACTCC-AACCCTTTGTGAACCTTACCGTCGTTGCCTCGGCGTG-
ANNGCAGCTACCCTGCGGCTACCCCGGGGCAGCTTGCTCCAAAAAAAGCTAACCTAAAGCTGCTCC
G-AAATATGCTCCAAA-CTCGAAATGAAGCTCC-AAACTTTT-T---------
TTTTTTCTGAATTACTTAATNAAATCAGTTAANACTTTCAACAACGGATCTCTTGGTTCTGGCATCG
ATGAAGAACGCAGCGAAATGCGATAAGTAATGTGAATTGCAGAATTCAGTGAATCATCGAATCTTT
GAACGCACATTGCGCCCATTAGTATTCTAGTGGGCATGCCTATTCGAGCGTCATTTCGACCCTTAAG
CCCTCGTTGCTTAGCGTTGGGAGCCTGCGTCCCGGGGCGCAGTTCCTCAAAGTTAGTGGCGGAGCT
AGGGCACACTCTAAGCGTAGTAAGTATTCTCGCTTCTGTGGTGTACCTGGCTTCCTGCCGTAAAACC
CCT----CATCTAGTGGTTGACCTCGAATTAGGTAGGAATA-------------------------------------A-
TTTGAAATCTGGCC----CTC-----GCGGTCCGAGTTGTAATTTGTAGAGGATGCTTTGGGCGCGGC-
GCCTTCCAAGTTCCCTAGAACGGGACGCCTTAGAGGGTGAGAGCCCCGTACGGTTGGA-
CGCCTAGCCTATGTATAGCTCCTTCGACGAGTCGAGTAGTTTGGGAATGCTGCTCTAAATGGGAGG
TAAATTTCTTCTAAAGCTAAATACCGGCCAGAGACC-
GATAGCGCACAAGTAGAGTGATCGAAAGATGAAAAGTACTTTGAAAAGAGGGTTAAATAGCACGT
GAAATTGTTGAAAGGGAAGCG-TTTGCGACCAGACCTTTTCCAGGCGGATCATCCGGCGTTC--
TCGCCGGTGCACTTCGCCTGG--TTTAGGCCAGCATCGGTTTT---
CCCAGGGGGATAAAGGCGGTGGGAACGTAGCTC----TTTCGGGAGTGTT-
ATAGCCCGCCGCGTAATACCCTTGG-GGGGACCGAGGACCGCGCT-TCG---
GCAAGGATGCTGGCGTAATGGTCGTCAACGACCCGTCTTGAAACACGGACCAAGGAGTCGAACAT
TTGTGCGAGTGTTTGGGTG--TCAAACCCTCACGCGTAATGAAAGTGAACGGAGGTGAGAGCCCCT--
---CG--CGGGTGCATCATCGACCGATCCTGATG-
TCTTCGGATGGATTTGAGTAAGAGCATAACTGTTCGGACCCGAAAGATGGTGAACTATGCGTGGAT
AGGGTGAAGCCAGAGGAAACTCTGGTGGAGGCTCGCAGCGGTTCTGACGTGCAAATCGATCGTCA
AATCTGCGCATGGGGGCGAAAGACTTATCGA-
ACCATCTAGTAGCTGGTTACCGCCGAAGTTTCCCTCAGGATAGCAGTGT--TG-
TCTTCAGTTTTATGAGGTAAAGCGAATGATTAGGGACTCGGGGGCGCTATTTTGCCTTCATCCATTC
TCAAACTTTAAATATGTAAGAAGCCCTTGTTACTTAGTTGAACGTGGGCATTCGAATGTACCAACA
CTAGTGGGCCATTTTTGGTAAGCAGAACTGGCGATGCGGGATGAACCGAACGCGGGGTTAAGGTG
CCAGAGTGGACGCTCATCAGACACCACAAAAGGTGTTAGTACATCTTGACAGCAGGACGGTGGCC
ATGGAAGTCGGAATCCGCTAAGGACTGTGTAACAACTCACCTGCCGAATGTACTAGCCCTGAAAAT
GGATGGCGCTCAAGCGTCT-CACCCATACCTCGCCCTTAGGGTAGAAACGATGCCCTAAGG----------
----

AAGGCCAAGCCTGCGGGCTGGTCAAGAATCTGTCGCTTATGTGCTCCATCAGCGTGGGTACCTCAA
CGGATCCTATCGTAGATTATATGATCACCAGAAACATGGAGGTCTTGGAGGAATATGAGCCCATGA
GATATCCTAACGCAACCAAGATCTTCCTCAACGGCTCCTGGATCGGTGTGCACCAAGATCCCAAGT
CTCTAGTTAGAGACGTTCAGCAGCTGCGCCGGGCCAACCAGATTCCCTCCGAGGTATCTTTAGTTC
GCGACATCCGAGACCGCGAGTTCAAGATTTTCTCAGACGCCGGCCGCGTCATGCGTCCCTTGTTTGT
TGTACAGCAAGAGGAT--
GACCCGGACACCGGTGTCCCCAAGGGCCACCTGGCTCTCACGAAGACCCAGATTGCGAAGCTGGA
GGCAAGCATCGACGTAGAGGTCGACGCTCCCGGCTACTATGGCTGGCAAGGGTTAGTTAACGACG
GTGTTATCGAGTATCTCGATGCGGAGGAGGAGGAGACGGCTATGATATGCATGACGCCCGAAGAC
TTGGAAACATATCGCATGGCCAAGGCCGGCATTGATATGCCTCAGGACAACGGG------
GACGAGATCAACAAGCGCCTCAAGACCAAGGTTAACCCCACGACGCACATGTACACGCACTGCGA
GATCCACCCGAGTATGCTTCTAGGTATTTGCGCTAGCATTATTCCCTTCCCGGACCATAATCAGGTA
CCCCTGAACGCGTCG--------------
TGAATGCCCCTCACGCATCCAGAACTGAACCACATCATCCTATGGTTCTCTGGAACACACTACCAC
GAAAAAACACGACTATTTGACGAATATTTGGAGCTAACCATCTCTCTTG-
TCTTTATAGGTTCACCTTCAGACCGGCCAATGCGTAAGTACC----------
ATAATCGCCAACGAGACATAGCACTGGGACGTAGCGGGGCTCATACGAAGATCGTAGGGTAACCA
AATCGGTGCTGCTTTCTGGCAAACCATCTCGAGCGAGCACGGCCTCGACAGCAATGGCGTGTACGT
ATTTGAGTCGTCAACCTTACCGCGAAGAAGTCCAACTAATCACCAATAAACAGCTACAACGGCACC
TCCGAGCTCCAGCTCGAGCGCATGAGCGTCTACTTCAACGAGGTACGGATTCGCGAAAGCA-----
GAAATAGATGGAGCTAGTTACTGACCGTATCGCCGCATGCAGGCCTCTGGCAACAAGTATGTTCCT

CGCGCTGTCCTCGTCGATCTCGAGCCCGGCACCATGGACGCCGTCCGTGCCGGTCCCTTCGGCCAG
CTCTTCCGACCGGACAACTTCGTCTTCGGTCAGTCCGGTGCTGGCAACAACTGGGCCAAGGGTCAC
TACACTGAGGGTGCTGAGCTGGTTGACAACGTCCTCGACGTCGTCCGCCGTGAGGCCGAAGGCTGC
GACTGCCTCCAGGGCTTCCAGATCACCCACTCCCTCGGTGGTGGTACCGGTGCCGGTATGGGTACC
CTGTTGATCTCCAAGATCCGCGAGGAGTTCCCCGACCGCATGATGGCCACCTTCTCCGTCATGCCTT
CGCCCAAGGTCTCCGACACGGTCGTCGAGCCCTACAACGCCACTCTCTCCGTCCACCAGCTGGTCG
AGAACTCTGACGAGACCTTCTGTATCGACAACGAGGCTCTGTACGATATCTGCATGCGTACGCTGA
AGTTGTCCAACCCCTCGTACGGCGATCTGAACCACCTGGTCTCCGCCGTCATGTCCGGCGTCACCAC
TTGTCTGCGTTTCCCCGGCCAGCTGAACTCTGACCTGCGCAAGCTCGCCGTGAACATGGTTCCTTTC
CCCCGTCTCCACTTCTTCATGGTCGGCTTCGCCCCTCTGACCAGCCGCGGCGCTCACTCCTTCCGCG
CCGTCACCGTTCCCGAGTTGACTCAGCAGATGTTCGACCCCAAGAACATGATGGCTGCCTCTGACT
TCCGCAACGGTCGCTACCTGACGTGCTCTGCCATCTTGTAAGATAGCCCCCTAAATCA-
TTTGTTAGTATCGGCCTGCTGACCTAAATCCCGTAGCCGTGGCAAGGTCTCCA

>Pyrenopolyporus_cinereopigmentosus_BCC89382

-----------------------------------ACCTTACCCTCGTTGCCTCGGCGTG-
AGCGCAGCTACCCTGCGCCTACCCCGGGGCAGCTTGCTCCAAAAAAAGCT-----
AAAACTGCTCCGAAAAAATGCTCCAAAGCTCGAAATGAAGCTCC-AAACTT-------------
GTTTTTCTGAATTACCTAATTAAATCAGTTAAAACTTTCAACAACGGATCTCTTGGTTCTGGCATCG
ATGAAGAACGCAGCGAAATGCGATAAGTAATGTGAATTGCAGAATTCAGTGAATCATCGAATCTTT
GAACGCACATTGCGCCCATTAGTATTCTAGTGGGCATGCCTATTCGAGCGTCATTTCGACCCTTAAG
CCCTCGTTGCTTAGCGTTGGGAGCCTGCGTCCCGGGGCGCAGCTCCTCAAAGTTAGTGGCGGAGCT
AGGGCACACTCTAAGCGTAGTAAGTATTCTCGCTTCTGTGGTGTACCTAGCTGCCTGCCGTAAAAC
CCCT----CTCTTAGTGGTTGACCTCGAATTAGGTAGGAATACCCGCTGAACTTAAGC---
TGAAGCGGCAACAGCTCAAA-TTTGAAATCTGGCC----CTC-----
GCGGTCCGAGTTGTAATTTGTAGAGGATGCTTTGGGCGCGGC-
GCCTTCCAAGTTCCCTAGAACGGGACGCCTTAGAGGGTGAGAGCCCCGTACGGTTGGA-
CGCCTAGCCTATGTATAGCTCCTTCGACGAGTCGAGTAGTTTGGGAATGCTGCTCTAAATGGGAGG
TAAATTTCTTCTAAAGCTAAATACCGGCCAGAGACC-
GATAGCGCACAAGTAGAGTGATCGAAAGATGAAAAGTACTTTGAAAAGAGGGTTAAATAGCACGT
GAAATTGTTGAAAGGGAAGCG-TTTGCGACCAGACCTTTTCCAGGCGGATCATCCGGCGTTC--
TCGCCGGTGCACTTCGCCTGG--TTTAGGCCAGCATCGGTTTT---
CCCAGGGGGATAAAGGCGGCGGGAACGTAGCTC----TCTCGGGAGTGTT-
ATAGCCCGCCGCGTAATACCCTTGG-GGGGACCGAGGACCGCGCT-TCG---
GCAAGGATGCTGGCGTAATGGTCGTCAACGACCCGTCTTGAAACACGGACCAAGGAGTCGAACAT
TTGTGCGAGTGTTTGGGTG--TCAAACCCTCACGCGTAATGAAAGTGAACGGAGGTGAGAGCCCCT--
---CG--CGGGTGCATCATCGACCGATCCTGATG-
TCTTCGGATGGATTTGAGTAAGAGCATAACTGTTCGGACCCGAAAGATGGTGAACTATGCGTGGAT
AGGGTGAAGCCAGAGGAAACTCTGGTGGAGGCTCGCAGCGGTTCTGACGTGCAAATCGATCGTCA
AATC--

GGCAACTGGGGCGACCAGAAGAAAGCCATGAGCTCCACGGCGGGCGTGTCGCAGGTCCTAAACCG
ATATACGT------TCGCATCGA---
CTCTTTCCCATTTGCGACGAACGAACACGCCCATCGGAAGGGACGGCAAGCTTGCGAAGCCGCGAC
AGCTACACAACACCCATTGGGGTCTGGTATGTCCGGCCGAGACGCCCGAAGGCCAAGCCTGCGGG
CTGGTAAAGAATCTGTCGCTTATGTGCTCCATCAGCGTGGGTACCTCAACGGATCCTATCGTAGATT
ATATGATCACTAGGAATATGGAGGTCTTGGAGGAATATGAGCCCATGAGATATCCTAACGCCACCA
AGATCTTCCTCAACGGCTCTTGGATCGGTGTGCACCAAGATCCCAAGTCTCTAGTTAGAGACGTTC
AGCAGCTGCGCCGGGCCAACCAGATTCCCTCCGAGGTATCTTTGGTTCGCGACATCCGAGACCGCG
AGTTCAAGATTTTTTCGGACGCCGGCCGCGTCATGCGTCCCCTGTTTGTTGTACAGCAAGAGGAT----
----

GACCCCGACACCGGCGTCCCCAAGGGCCACCTAGCTCTCACGAAGACCCAGATTGCGAAGCTGGA
GGCAAGCATCGACGTAGAGGTCGACGCTCCCGGCTACTATGGTTGGCAAGGGTTGGTTAACGACG
GTGTTATCGAGTATCTCGATGCGGAAGAGGAGGAGACGGCTATGATATGCATGACGCCCGAAGAC

TTGGAGACTTATCGCATGGCCAAGGCCGGCATTGACATGCCTCAGGACAACGGG------
GACGAGATTAACAAGCGCCTCAAGACCAAGGTCAACCCCACGACGCACATGTACACGCATTGCGA
GATCCATCCGAGTATGCTTCTAGGTATTTGCGCTAGCATTATTCCCTTCCCAGACCATAACCAGGTA

GGTTCTCTAGAACACATTACCATGAAAAAACACGACTATTTGACGAATATTTGGAGCTAACCATAT
ATCTTC-TCTTTATAGGTTCACCTTCAGACCGGCCAATGCGTAAGTT------------
ACCATCGCCAACGAGACATAGCACTGGGACATAGCGGGGCTCATACGAAGATCGTAGGGTAACCA
AATCGGTGCTGCTTTCTGGCAAACCATCTCGAGCGAGCACGGCCTCGACAGCAATGGCGTGTACGT
ATTTGAGTCGTCAACATTACCGCGAAGAACTCCAACTAATCACCAATAAACAGCTACAACGGCACC
TCCGAGCTCCAGCTCGAGCGCATGAGCGTCTACTTCAACGAGGTACGGATTCGCGAAAGCA-----
GAAATAGATGGAACTAGTTACTGATCGTATCACCGCATGCAGGCCTCTGGCAACAAGTATGTTCCT
CGCGCCGTCCTCGTCGATCTCGAGCCCGGCACCATGGACGCCGTCCGTGCCGGTCCCTTCGGTCAG
CTCTTCCGACCGGACAACTTCGTTTTCGGTCAGTCCGGTGCTGGCAACAACTGGGCCAAGGGTCAC
TACACTGAGGGTGCTGAGCTGGTTGACAACGTCCTCGACGTCGTTCGCCGTGAGGCCGAAGGCTGC
GACTGCCTCCAGGGCTTCCAGATCACCCACTCCCTCGGTGGTGGTACCGGTGCCGGTATGGGTACC
CTGTTGATCTCCAAGATCCGCGAGGAGTTCCCCGACCGCATGATGGCTACCTTCTCCGTCATGCCTT
CCCCCAAGGTCTCCGACACCGTCGTCGAGCCCTACAACGCCACTCTCTCCGTCCACCAGCTGGTCG
AGAACTCTGACGAGACGTTCTGTATCGACAACGAGGCTCTGTACGATATCTGCATGCGTACGCTGA
AGTTGTCCAACCCCTCGTACGGCGATCTGAACCACCTGGTCTCCGCCGTCATGTCCGGCGTCACGA
CTTGTCTGCGTTTCCCCGGCCAGCTGAACTCTGACCTGCGCAAGCTCGCCGTGAACATGGTTCCTTT
CCCTCGTCTCCACTTCTTCATGGTCGGCTTCGCCCCTCTGACCAGCCGCGGCGCTCACTCTTTCCGC
GCCGTCACCGTTCCCGAGTTGACTCAGCAGATGTTCGACCCCAAGAACATGATGGCTGCCTCCGAT
TTCCGCAACGGTCGCTACCTGACGTGCTCGGCCATCTTGTAAGATATCCTCCAGAATTG--
TCGTTCGTATCAGCCTGCTAACCTAAGTTTCGTAGCCGTGGCAAGGTCTCCA

>Pyrenopolyporus_macrosporus_BCC89373

---GCGTG-
AGCGCAGCTACCCCGCGGCTACCCCGGGGCGGCTTGCTCCAAGAAAAGCT-----AAAACTGCTCGG-
AAAAATGCTCCAAAGCTCGAAATGAAGCTCC-AAACTT-------------
GTTTTTCTGAATTATTTAATTAAATCAGTTAAAACTTTCAACAACGGATCTCTTGGTTCTGGCATCG
ATGAAGAACGCAGCGAAATGCGATAAGTAATGTGAATTGCAGAATTCAGTGAATCATCGAATCTTT
GAACGCACATTGCGCCCATTAGTATTCTAGTGGGCATGCCTATTCGAGCGTCATTTCGACCCTTAAG
CCCTCGTTGCTTAGCGTTGGGAGCCTGCGTCCCGGGGCGCAGTTCCTCAAAGTTAGTGGCGGAGCT
AGGGCACACTCTAAGCGTAGTAAGTATTCTCGCTTC---
----------------------------TGAAGCGGCAACAGCTCAAA-TTTGAAATCTGGCC----CTC-----
GCGGTCCGAGTTGTAATTTGTAGAGGATGCTTTGGGCGCGGC-
GCCTTCCAAGTTCCCTAGAACGGGACGCCTTAGAGGGTGAGAGCCCCGTACGGTTGGA-
CGCCTAGCCTATGTATAGCTCCTTCGACGAGTCGAGTAGTTTGGGAATGCTGCTCTAAATGGGAGG
TAAATTTCTTCTAAAGCTAAATACCGGCCAGAGACC-
GATAGCGCACAAGTAGAGTGATCGAAAGATGAAAAGTACTTTGAAAAGAGGGTTAAATAGCACGT
GAAATTGTTGAAAGGGAAGCG-TTTGCGACCAGACCTTTTCCAGGCGGATCATCCGGCGTTC--
TCGCCGGTGCACTTCGCCTGG--TTTAGGCCAGCATCGGTTTT---
CCCAGGGGGATAAAGGCGGCGGGAACGTGGCTC----TCTCGGGAGTGTT-
ATAGCCCGCCGCGCAATACCCTTGG-GGGGACCGAGGACCGCGCT-TCG---
GCAAGGATGCTGGCGTAATGGTCGTCAACGACCCGTCTTGAAACACGGACCAAGGAGTCGAACAT
TTGTGCGAGTGTTTGGGTG--TTAAACCCTCACGCGTAATGAAAGTGAACGGAGGTGAGAGCCCCT--
---CG--CGGGTGCATCATCGACCGATCCTGATG-
TCTTCGGATGGATTTGAGTAAGAGCATAACTGTTCGGACCCGAAAGATGGTGAACTATGCGTGGAT
AGGGTGAAGCCAGAGGAAACTCTGGTGGAGGCTCGCAGCGGTTCTGACGTGCAAATCGATCGTCA
AATCTGCGCATGGGGGCGAAAGACTTATCGA-ACCATCTAGTAGCT---

GGCAACTGGGGCGACCAGAAGAAAGCCATGAGCTCCACGGCGGGCGTGTCGCAGGTCCTAAACCG
ATATACGT------TCGCATCGA---
CTCTTTCTCATTTGCGACGAACGAACACGCCCATCGGAAGGGACGGCAAGCTTGCGAAGCCGCGAC

AGCTACACAACACCCATTGGGGTCTGGTATGTCCGGCCGAGACGCCCGAAGGCCAAGCCTGCGGG
CTGGTAAAGAATCTGTCGCTTATGTGCTCCATCAGCGTGGGTACCTCAACGGATCCTATCGTAGATT
ATATGATCACTAGGAATATGGAGGTCTTGGAGGAATATGAGCCCATGAGATATCCTAACGCCACCA
AGATCTTCCTCAACGGCTCTTGGATCGGCGTGCACCAAGATCCCAAGTCTCTAGTTAGAGACGTTC
AGCAGCTGCGCCGGGCCAACCAGATTCCCTCCGAGGTATCTTTAGTTCGCGACATCCGAGACCGCG
AGTTCAAGATTTTTTCGGACGCCGGCCGCGTCATGCGTCCCTTGTTTGTTGTACAGCAAGAGGAC----
----

GACCCCGACACCGGCGTCCCCAAGGGCCACCTAGCTCTCACGAAGACCCAGATTGCGAAGCTGGA
GGCAAGCATCGACGTAGAGGTCGACGCCCCCGGCTACTATGGTTGGCAAGGGTTGGTTAACGACG
GTGTTATCGAGTATCTCGATGCGGAAGAGGAGGAGACGGCTATGATATGCATGACGCCCGAAGAC
TTGGAGACTTATCGCATGACCAAGGCCGGCATTGATATGCCTCAGGACAACGGG------
GACGAGATTAACAAGCGCCTCAAGACCAAGATTAACCCCACGACGCACATGTACACGCATTGCGA
GATCCATCCGAGTATGCTTCTAGGTATTTGCGCTAGCATTATTCCCTTCCCAGACCATAATCAGGTA
ACTCCAAACCCCCTG--------------
TTTCTGCCCCTCACGCATCCAGAACTGAACCACATCATCCCGTGGTTCTCTAGAGCACACTACCGTG
AAAAAACACGACTATTTGACGAATATTTGGAGCTAACCATATCTCTTC-
TCTTTATAGGTTCACCTTCAGACCGGCCAATGCGTAAGTACC----------
ATAATCGCCAACGAGACATAGCACTGGGACATAGCGGGGCTCATACGAAGATCGTAGGGTAACCA
AATCGGTGCTGCTTTCTGGCAAACCATCTCGAGCGAGCACGGCCTCGACAGCAATGGCGTGTACGT
ATTTGAGTCGTCAACGTTACCGCGAAGAACTCCAACTAACCGTCGGTAAACAGCTACAACGGCACC
TCCGAGCTCCAGCTCGAGCGCATGAGCGTTTACTTCAACGAGGTACGGATTCGCGAAAGCA-----
GAAATAGATGGAACTAGTTACTGATCGTATCATCGCATGCAGGCCTCTGGCAACAAGTATGTTCCT
CGCGCCGTCCTCGTCGATCTCGAGCCCGGCACCATGGACGCCGTCCGTGCCGGTCCCTTCGGTCAG
CTCTTCCGACCGGACAACTTCGTTTTCGGTCAGTCCGGTGCTGGCAACAACTGGGCCAAGGGTCAC
TACACTGAGGGTGCTGAGCTGGTTGACAACGTCCTCGACGTCGTTCGCCGTGAGGCCGAAGGCTGC
GACTGCCTCCAGGGCTTCCAGATCACCCACTCCCTCGGTGGTGGCACCGGTGCCGGTATGGGTACC
CTGTTGATCTCCAAGATCCGCGAGGAGTTCCCCGACCGCATGATGGCTACCTTCTCCGTCATGCCTT
CCCCCAAGGTCTCCGACACCGTCGTCGAGCCCTACAACGCCACTCTCTCCGTCCACCAGCTGGTCG
AGAACTCTGACGAGACGTTCTGTATCGACAACGAGGCTCTGTACGATATCTGCATGCGTACGCTGA
AGTTGTCCAACCCCTCGTACGGCGATCTGAACCACCTGGTCTCCGCCGTCATGTCCGGCGTCACCAC
TTGCCTGCGTTTCCCCGGCCAGCTGAACTCTGACCTACGCAAGCTCGCCGTGAACATGGTTCCTTTC
CCTCGTCTCCACTTCTTCATGGTCGGCTTCGCCCCCCTGACCAGCCGCGGCGCTCACTCCTTCCGCG
CCGTCACCGTTCCCGAGTTGACTCAGCAGATGTTCGACCCCAAGAACATGATGGCTGCCTCCGATT
TCCGCAACGGTCGCTACCTGACGTGCTCGGCCATCTTGTAAGATATCCCCCGGAATTG--
TCGTTCGTATCAGCCTGCTAACCTAAATTTCGTAGCCGTGGCAAGGTCTCCA

>Annulohypoxylon_moriforme

ACCCTTTGTGAACCTTACCGTCGTTTCCTCGGCGTACTACACGGCTACCCTGTGGCTACCCTGAGGT
TACCTACCCTGGAGAAGGCTACTCTGTAGCTATTTTATAAGCGCGTTGGTGGGTGTGCCGGTAGGT
GGCTAAATTCTGTTTTTTACCCTGGAGAACTGAAAGATTTAAAACAAAAGTTTAAAACTTTCAACA
ACGGATCTCTTGGTTCTGGCATCGATGAAGAACGCAGCGAAATGCGATAAGTAATGTGAATTGCAG
AATTCAGTGAATCATCGAATCTTTGAACGCACATTGCGCCCATTAGTATTCTAGTGGGCATGCCTAT
CCGAGCGTCATTTCAACCCTTAAGCCCCTGTTGCTTAGCGTTGGGAATCTGCGTCTTAGGGCGCAGT
TCCTTAAAGTTAGTGGCGGAGTTATAGCACACCTTAAGCGTAGTAGTATGCCTCGCTTCTGGAGTA
GCTGTAGCTGCCGGCCGTAAAACCCC--
TGAAGCGGCAACAGCTCAAA-TTTGAAATCTGGCC----CTC-----
GCGGTCCGAGTTGTAATTTGCAGAGGATGCTTTTGGTGCGGT-
GCCTTCCGAGTTCCCTGGAACGGGACGCCAGAGAGGGTGAGAGCCCCGTACGGTTGGA-
CACCTACCCTATATATAGCTCCTTCGACGAGTCGAGTAGTTTGGGAATGCTGCTCTAAATGGGAGG
TAAATTTCTTCTAAAGCTAAATACCGGCCAGAGACC-
GATAGCGCACAAGTAGAGTGATCGAAAGATGAAAAGCACTTTGAAAAGAGGGTTAAATAGCACGT
GAAATTGTTGAAAGGGAAGCG-TTTGCGACCAGACCTTCTCCAGGCGGATCATCCGGCTCTC--
TCGCCGGTGCACTTCGCCTGG--TTTAGGCCAGCATCGGTTTC---
CTTAGGGGGATAAAGGCCTGGGGAACGTAGCTC----TTTAGGGAGTGTT-
ATAGCCCCTGGCGTAATACCCCTCG-GGGGACCGAGGACCGCGCT-TCG---
GCAAGGATGCTGGCGTAATGGTCGTCAACGACCCGTCTTGAAACACGGACCAAGGAGTCGAACAT

TTGTGCGAGTGTTTGGGTG--TCAAACCCTCACGCGTAATGAAAGTGAACGGAGGTGAGAGCCCCT--
--A-G---GGGTGCATCATCGACCGATCCTGATG-
TCTTCGGATGGATTTGAGTAAGAGCATAACTGTTCGGACCCGAAAGATGGTGAACTATGCGTGGAT
AGGGTGAAGCCAGAGGAAACTCTGGTGGAGGCTCGCAGCGGTTCTGACGTGCAAATCGATCGTCA
AATCTGCGCATGGGGGCGAAAGACTTATCGA-
ACCATCTAGTAGCTGGTTACCGCCGAAGTTTCCCTCAGGATAGCAGTGT--TG-
TCTTCAGTTTTATGAGGTAAAGCGAATGATTAGGGACTCGGGGGCGCTATATTGCCTTCATCCATTC
TCAAACTTTAAATATGTAAGAAGCCCTTGTTACTTAGTTGAACGTGGGCATTCGAATGTACCAACA
CTAGTGGGCCATTTTTGGTAAGCAGAACTGGCGATGCGGGATGAACCGAACGCGGGGTTAAGGTG
CCAGAGTGGACGCTCATCAGACACCACAAAAGGTGTTAGTACATCTTGACAGCAGGACGGTGGCC
ATGGAAGTCGGAATCCGCTAAGGACTGTGTAACAACTCACCTGCCGAATGTACTAGCCCTGAAAAT
GGATGGCGCTCAAGCGTCT-
CACCCATACCTCGCCCTTAGGGTAGAAACGATGCCCTAAGGACGCTGTATCGACGGAAACAAACGT
TTCCAAATTGAGCTTGCCGCCAAGCCCGCAATTATCACCAACGGCTTGAAGTATTCACTCGCCACG
GGCAATTGGGGCGATCAGAAGAAGGCAATGAGCTCAACTGCCGGTGTATCGCAGGTATTGAATCG
ATATACCT------TCTCGTCAA---
CCCTTTCTCATTTACGACGAACAAACACCCCTATCGGAAGAGATGGAAAGCTAGCCAAACCACGAC
AGCTTCACAACACCCATTGGGGACTCGTTTGTCCGGCAGAAACTCCCGAAGGTCAAGCTTGTGGTT
TGGTGAAGAATTTGTCCTTGATGTGCTCTATCAGCGTTGGAACATCGACAGATCCTATCGTGGACTA
CATGATCACCAGGAATATGGAAGTCTTGGAGGAGTATGAACCGATGAGATACCCCAACGCTACCA
AGATTTTCCTCAACGGCTCTTGGATTGGTGTGCACCAGGACCCCAAGACCCTCGTCAGAGATGTCC
AGGCACTTCGTCGAGCCAACCAGATTCCTGCCGAGGTATCGTTAGTTCGTGACATCCGGGACCGTG
AATTCAAGATCTTTTCGGACGCAGGTCGGGTGATGCGCCCTTTGTTCCGTGTGCAACAGGAAGAC---
----
ATCCATGAACAGGGCATCGAGAAAGGCACGTTAGCTCTTACTAAGCCGATGATAAAGCGCCTGGA
AGCAGATGTCGATCTGGATCCGGAAAGCGAGGCATACTTTGGCTGGCAAGGTTTGGTCAATGAGG
GTGTTATCGAGTTTCTCGATGCAGAGGAAGAAGAGACTGCCATGATTTGCATGACTCCGGAAGATT
TGGACACTTATCGCATGACCAAGCTTGGATATGAAGTATCCCAGGACAACGGA------
GACGAAGTGAACAAGCGACTCAAAACCAAAGTGAATCCATCGACGCACATGTATACCCATTGCGA
GATTCACCCCAGTATGCTCTTGGGTATCTGCGCAAGCATCATTCCGTTCCCAGATCACAACCAGGTA
CCCCTGAACGCGTCCCTCAAACCCCTTGATTCCTGCCCCTCACGCACAAAAAAC---
ACCACAACATCGTGTTCTTTTCTGTGTAACATCGGCATCAATTTGAGCAATAATTACTTCGGATCAG
ACAGCTAACCGTGTTTTTTTATCTCAATAGGTTCACCTCCAGACCGGCCAGTGCGTAAGTACTATAG
C----
TACAACCACCGACGAAGAATCGCGACGGAATATAGCGGGGCTCACACGAAGATTATAGGGTAACC
AAATTGGTGCTGCTTTCTGGCAAACCATCTCTGGCGAGCACGGCCTCGACAGCAATGGCGTGTACG
TATATCAATTGTCAATTCTGACGCCAAGAAGCGCAACTAACGACCAATAAACAGCTACAATGGAAC
CTCTGAGCTCCAGCTCGAACGCATGAGCGTTTACTTTAACGAGGTACGCAACCGGGGCAACACCTT
ACATTGGTTTAAGAGTAGTTACTAATCACCCCAACATGCACAGGCATCTGGTAACAAGTATGTTCC
CCGAGCCGTCCTCGTCGATCTCGAGCCGGGTACCATGGACGCCGTCCGTGCCGGCCCCTTCGGCCA
GCTTTTCCGTCCCGACAACTTCGTCTTCGGCCAGTCTGGTGCCGGAAACAACTGGGCGAAGGGTCA
CTACACGGAGGGTGCTGAGCTTGTTGACAACGTTCTTGATGTTGTTCGTCGTGAGGCTGAGGGCTG
TGACTGCCTTCAGGGTTTCCAGATCACCCACTCTCTCGGTGGTGGTACCGGTGCCGGTATGGGTACT
CTGCTGATCTCCAAGATCCGCGAGGAGTTCCCCGACCGTATGATGGCTACCTTCTCCGTCGTTCCCT
CTCCTAAGGTTTCCGACACCGTCGTCGAGCCTTACAACGCCACTCTCTCAGTCCACCAGCTGGTTGA
GAACTCCGACGAGACGTTCTGTATCGACAACGAAGCTCTTTACGACATTTGCATGCGTACTCTCAA
GCTATCTAACCCCTCGTACGGTGACTTGAACCACTTGGTCTCTGCCGTCATGTCTGGTGTTACCACT
TGCTTGCGCTTCCCTGGTCAGCTGAACTCTGACCTTCGCAAGCTCGCTGTGAACATGGTTCCTTTCC
CCCGTCTCCACTTCTTCATGGTTGGCTTCGCTCCCCTGACCAGCCGTGGCGCTTACTCCTTCCGCGCC
GTTACCGTTCCCGAGTTGACCCAGCAGATGTTCGACCCCAAGAACATGATGGCTGCTTCTGACTTCC
GCAACGGCCGTTACCTAACGTGCTCTGCCATCTTGTAAGATTGCATCTTTCTATTATAACTCACAAG
CAATGTACTAATTCGACCCTTCTAGCCGTGGCAAGATCTCCA

>Annulohypoxylon_stygium

----TACTGAGTTATAAAAACTCC-AACCCTTTGTGAA-CCTACCATGTTTCCTCCGGCGTACCGCGC--
CTACCCGGGGGCTGCCT--
GTGCTACCTATCCTTCGGGGTGCTAGCCTGTAGCCGCTCCCTAAACGTGCATCAAAGC--

TCTGAGGGGTC--TGAATTCTA-
TTTTTGTGATCAAACCAAGGTTTTAAAAACCAAATACGTTAAAACTTTCAACAACGGATCTCTTGGT
TCTGGCATCGATGAAGAACGCAGCGAAATGCGATAAGTAATGTGAATTGCAGAATTCAGTGAATC
ATCGAATCTTTGAACGCACATTGCGCCCATTAGTATTCTAGTGGGCATGCCTATTCGAGCGTCATTA
CAACCCTTAAG-
CCTTGTAGCTTAGCGTTGGGAATCTACCCCTCGAGGGGTAGTTCCTTAAATTTAGTGGCGGGGTTAT
AGCACACTCTAAGCGTAGTAGT--
AACTCGCTTTCAGGGAGGCTGTAGCTGCTTGCCGTAAAACCCCCTATACTTATAG----------------------
--------------------------------GCTCAAA-TTTGAAATCTGGCC----CTA-----
GCGGTCCGAGTTGTAATTTGCAGAGGATGCTTTTGGTGCGGT-
GCCTTCCGAGTTCCCTGGAACGGGACGCCATAGAGGGTGAGAGCCCCGTACGGTTGGA-
TACCTAGCCTCTATATAGCTCCTTCGACGAGTCGAGTAGTTTGGGAATGCTGCTCTAAATGGGAGG
TAAATTTCTTCTAAAGCTAAATACCGGCCAGAGACC-
GATAGCGCACAAGTAGAGTGATCGAAAGATGAAAAGCACTTTGAAAAGAGGGTTAAATAGCACGT
GAAATTGTTGAAAGGGAAGCG-TTTGCGACCAGACCTTCTCCAGGGGGATCAACCGCTGTTC--
TCAGCGGTGCACTCCCCCTGG--TTTAGGCCAGCATCGGTTCT---
CTTAGGGGGATAAAGGCCTGGGGAACGTAGCTC----TTTAGGGAGTGTT-
ATAGCCCCTAGCGTAATACCCTTCA-GGGGACCGAGGACCGCGCT-TCG---
GCAAGGATGCTGGCGTAATGGTCGTCAACGACCCGTCTTGAAACACGGACCAAGGAGTCGAACAT
TTGTGCGAGTGTTTGGGTG--TTAAACCCTCACGCGTAATGAAAGTGAACGGAGGTGAGAGCCTT----
-A-----GGGTGCATCATCGACCGATCCTGATG-
TCTTCGGATGGATTTGAGTAAGAGCATAACTGTTCGGACCCGAAAGATGGTGAACTATGCGTGGAT
AGGGTGAAGCCAGAGGAAACTCTGGTGGAGGCTCGCAGCGGTTCTGACGTGCAAATCGATCGTCA
AATCTGCGCATGGGGGCGAAAGACTTATCGA-
ACCATCTAGTAGCTGGTTACCGCCGAAGTTTCCCTCAGGATAGCAGTGT--TG-
TCTTCAGTTTTATGAGGTAAAGCGAATGATTAGGGACTCGGGGGCGCTATATTGCCTTCATCCATTC
TCAAACTTTAAATATGTAAGAAGCCCTTGTTACTTAGTTGAACGTGGGCATTCGAATGTACCAACA
CTAGTGGGCCATTTTTGGTAAGCAGAACTGGCGATGCGGGATGAACCGAACGCGGGGTTAAGGTG
CCAGAGTGGACGCTCATCAGACACCACAAAAGGTGTTAGTACATCTTGACAGCAGGACGGTGGCC
ATGGAAGTCGGAATCCGCTAAGGACTGTGTAACAACTCACCTGCCGAATGTACTAGCCCTGAAAAT
GGATGGCGCTCAAGCGTCT-
CACCCATACCTCGCCCTTAGGGTAGAAACGATGCCCTAAGGGCGCTGCATCGAAGGCGGCAGGAG
ATTCCAGATTGAGCTTGCCGCCAAGCCTGCTATCATCACCAATGGTCTGAAATACTCGCTCGCCAC
GGGTAACTGGGGCGACCAGAAAAAGGCCATGAGCTCAACCGCAGGCGTATCTCAGGTCTTGAATC
GGTACACCT------TCTCGTCGA---
CCCTTTCTCACTTGCGGCGAACGAACACGCCTATTGGGAGGGACGGAAAGCTGGCCAAGCCGCGG
CAGCTTCACAACACTCATTGGGGCCTCGTCTGTCCTGCGGAGACCCCCGAAGGCCAGGCTTGCGGC
CTAGTTAAGAATCTGTCCCTCATGTGCTCTATCAGCGTGGGAACGTCGACGGATCCTATAGTGGAC
TACATGATTACTAGGAATATGGAAGTCCTGGAGGAGTATGAGCCGATGAGATACCCCAATGCTACC
AAGATCTTCCTCAACGGATCCTGGATCGGCGTGCACCACGACCCCAAGAACCTAGTCAGGGATGTT
CAGGCACTTCGTCGGACCAACCAGATCCCCGCCGAGGTATCGCTGGTTCGCGATATCCGAGATCGC
GAATTCAAGATCTTCTCGGATGCCGGTCGCGTGATGCGGCCTTTATTCCGCGTGCAGCAGGAAGAT-
----
ATCGAAGAGCAGGGCATCGAGAAGGGCACGTTGGCCCTTACCAAGTCGATGATTAAGCGTCTCGA
GGCGGACGTCGACATAGATCCGGAGAGCGATGCGTACTTTGGTTGGCAGGGCCTGGTGAATGAAG
GTGTCATCGAGTTCCTCGACGCGGAGGAGGAGGAGACTGCCATGATCTGCATGACGCCCGAAGATT
TGGACAACTTTCGCATGACCAAGCTTGGGTTCGACGTGTCCCAGGACAACGGC------
GACGAGGTAAACAAGCGGCTGAAGACCAAAGTGAACCCCTCGACGTACATGTACACCCACTGCGA
GATCCACCCCAGCATGCTCCTGGGAATCTGCGCGAGCATCATCCCTTTCCCGGACCACAA------------
---------TCCAAACCCCTTGAATCTTGCCCCTCACGCACAAAAAAC---
ACCACAACATCGCATTCTTTCCTGTGCAACAGCAGCATCGAATCGAATCCCGATGACCGTGA--
ATCATAGCTAACCATATTTTTTCATCTCAACAGGTTCACCTCCAGACCGGCCAGTGCGTAAGTACTA
TAGC----
TACAATCTCCGACGAAGAATCGCGACGGAATATAGCGGGGCTCACACGATGATAATAGGGTAACC
AAATTGGTGCTGCTTTCTGGCAAACCATCTCTGGCGAGCACGGTCTCGACAGCAATGGCGTGTAAG
TATCTGAATTGTCAATTCTCGCGACGTAAAAAATAACTGACCACCAATCAATAGCTACAACGGAAC
CTCGGAGCTCCAGCTCGAGCGCATGAGCGTCTACTTCAACGAGGTACGCAACCAGGGCAACACAT

AACAAATATTTAAAAGTAGTTACTAATCACCCCAACATGCACAGGCATCTGGTAACAAGTATGTTC
CCCGCGCCGTCCTCGTCGATCTCGAGCCGGGTACTATGGACGCCGTCCGTGCTGGCCCCTTCGGCC
AACTTTTCCGTCCCGACAACTTCGTCTTCGGCCAGTCTGGTGCCGGAAACAACTGGGCGAAGGGTC
ACTACACTGAGGGTGCTGAGCTAGTTGACAACGTTCTGGATGTTGTCCGTCGTGAGGCTGAGGGCT
GTGACTGCCTCCAGGGTTTCCAGATTACCCACTCTCTCGGTGGTGGTACCGGTGCCGGTATGGGTAC
CCTGCTGATCTCTAAGATCCGCGAAGAGTTCCCCGACCGTATGATGGCTACCTTCTCCGTCGTCCCC
TCCCCCAAGGTTTCCGACACCGTCGTCGAGCCTTACAACGCCACTCTCTCAGTTCACCAGTTGGTTG
AGAACTCCGACGAGACCTTCTGTATCGACAACGAAGCTCTGTACGATATCTGCATGCGTACTCTCA
AGCTGTCCAACCCCTCTTACGGTGACCTTAACCACCTGGTCTCTGCCGTCATGTCTGGTGTCACCAC
TTGCTTGCGTTTCCCTGGTCAGCTTAACTCTGACTTGCGCAAGCTTGCCGTCAACATGGTTCCTTTCC
CCCGTCTTCACTTCTTCATGGTCGGCTTCGCTCCCTTGACCAGCCGTGGCGCCTACTCCTTCCGCGCC
GTTACCGTTCCCGAGTTGACCCAGCAGATGTTCGACCCCAAGAACATGATGGCTGCTTCTGACTTCC
GCAACGGTCGTTACCTAACGTGCTCTGCCAT--

>Annulohypoxylon_substygium_MUCL51708

-CATTACTGAGT--ACAAAAACTCCAACCCTATGTGAA-
CCTACCCTGTTTCCTCCGGCGTACCGCGCGCCTATCCGGGGGCTACCT--
GTGCCGCCTACCCTTCGGGGTACCAGCCTGTAGCCTTCTCGCAAGTGTGCACCAACCCTTTTTAGG
GGTC--TGAACTCTA-TCCTCGTGGT--
AACTAAAGTTTTTTAACTAAATACTTTAAAACTTTCAACAACGGATCTCTTGGTTCTGGCATCGAT
GAAGAACGCAGCGAAATGCGATAAGTAATGTGAATTGCAGAATTCAGTGAATCATCGAATCTTTG
AACGCACATTGCGCCCATTAGTATTCTAGTGGGCATGCCTATTCGAGCGTCATTACAACCCTTAAG-
CCCTGTAGCTTAGCGTTGGGAATCTACGCCCCGGGGCGCAGTTCCTTAAATTTAGTGGCGGAGTTA
TAGCATACCCCAAGCGCAGTAGTTTAGCTCGCTTTCAGGGAGGCTGTAG-
TGCCTGCCGTAAAACCCCCTATACTT--

CCCCTGAACGCGTCCTCCAAACCCCTTGATTCTTGCCCCTCACGCACAAAAAAC---
ACCACAACATCGCACTCTTTCCTGTGCAACGCTAGCATCAAATTAGCCCACGATGATGTTATGAATT
ACAGCTAACCATGTTTTTTCATCCCAACAGGTTCACCTCCAGACCGGCCAGTGCGTAAGTACTATA
GC----TACAATCACCGACGAAGAATCGCAACAGAATATAGCGGGGCTCACACGATGAT-
ATAGGGTAACCAAATTGGTGCTGCTTTCTGGCAAACCATCTCTGGCGAGCACGGTCTCGACAGCAA
TGGCGTGTAAGTATCTGGATTGCCAATTCCGACGCCAGCAAAAATAACTGACCACCAATAAATAGC
TACAACGGAACCTCGGAGCTCCAGCTCGAACGCATGAGCGTTTACTTCAACGAGGTACGCAACCAG
GGCAACAGGTAGCAAACATTCAAAAATAGTTACTAATCACCCCAACATGCACAGGCATCTGGCAA
CAAGTATGTTCCCCGCGCCGTCCTCGTCGATCTCGAGCCGGGTACTATGGACGCCGTCCGTGCTGG
CCCCTTCGGCCAACTTTTCCGTCCCGACAACTTCGTCTTCGGCCAGTCTGGTGCCGGAAACAACTGG
GCGAAGGGTCATTACACTGAGGGTGCCGAGCTGGTTGACAACGTTCTTGATGTTGTTCGTCGTGAG
GCTGAGGGCTGCGATTGCCTCCAGGGTTTCCAGATCACCCACTCTCTCGGTGGTGGTACCGGTGCC
GGTATGGGTACCTTGTTGATCTCTAAGATCCGCGAAGAGTTCCCCGACCGAATGATGGCCACCTTC

TCCGTCGTTCCCTCCCCTAAGGTTTCCGACACCGTCGTCGAGCCTTACAACGCCACTCTCTCAGTTC
ACCAGCTGGTTGAGAACTCCGATGAGACCTTCTGTATCGACAACGAAGCTCTGTACGACATCTGCA
TGCGTACACTCAAGCTGTCCAACCCCTCTTACGGCGACCTGAACCACCTCGTCTCTGCCGTCATGTC
TGGTGTCACCACTTGCTTGCGTTTCCCTGGTCAGCTTAACTCTGACTTGCGAAAGCTTGCCGTCAAC
ATGGTTCCTTTCCCTCGTCTTCATTTCTTCATGGTCGGCTTCGCTCCCTTGACCAGCCGTGGCGCCTA
CTCCTTCCGCGCCGTTACCGTCCCCGAGTTGACCCAGCAGATGTTCGACCCTAAGAACATGATGGC
CGCTTCTGACTTCCGTAACGGTCGTTACCTGACGTGCTCTGCCATCTTGTAAGATAACATCCTACTG
ATCCTTCTAGATAT---TGTGCTAATTTATATCTTTCAGCCGTGGCAAGATCTCCA

>Annulohypoxylon_substygium_STMA14066

-CATTACTGAGTTATAAAAAACTCCAACCCTATGTGAA-
CCTACCCTGTTTCCTCCGGCGTACCGCGCCTATCCGGGGGCTACCT--
GTGCCGCCTACCCTTCGGGGTACCAGCCTGTAGCCTTCTCGCAAGTGTGCACCAACCCTTTTTAGG
GGTC--TGAACTCTA-
TCCTAGTGGTTAAACTAAAGTTTTTTAACTAAATACTTTAAAACTTTCAACAACGGATCTCTTGGT
TCTGGCATCGATGAAGAACGCAGCGAAATGCGATAAGTAATGTGAATTGCAGAATTCAGTGAATC
ATCGAATCTTTGAACGCACATTGCGCCCATTAGTATTCTAGTGGGCATGCCTATTCGAGCGTCATTA
CAACCCTTAAG-
CCCTGTAGCTTAGCGTTGGGAATCTACGCCCCGGGGCGCAGTTCCTTAAATTTAGTGGCGGAGTTA
TAGCATACCCCAAGCGCAGTAGTTTAGCTCGCTTTCAGGGAGGCTGTAGCTGCTTGCCGTAAAACC
CCCTATACTTATAG--

------------------------------TCCAAACCCCTTGATTCTTGCCCCTCACGCACAAAAAAC---
ACCACAACATCGCACTCTTTCCTGTGCAACGCTAGCATCAAATTAGCTCACGATGATGTTATGAATT
ACAGCTAACCATGTTTTTTCATCCCAACAGGTTCACCTCCAGACCGGCCAGTGCGTAAGTACTATA
GC----TACAATCACCGACGAAGAATCGCGACAGAATATAGCGGGGCTCACACGATGAT-
ATAGGGTAACCAAATTGGTGCTGCTTTCTGGCAAACCATCTCTGGCGAGCACGGTCTCGACAGCAA
TGGCGTGTAAGTATCTGGATTGCCAATTCCGACGCCAGCAAAAATAACTGACCACCAATAAATAGC
TACAACGGAACCTCGGAGCTCCAGCTCGAACGCATGAGCGTTTACTTCAACGAGGTACGCAACCAG
GGCAACAGGTAGCAAACATTCAAAAATAGTTACTAATCACCCCAACATGCACAGGCATCTGGCAA
CAAGTATGTTCCCCGCGCCGTCCTCGTCGATCTCGAGCCGGGTACTATGGACGCCGTCCGTGCTGG
CCCCTTCGGCCAACTTTTCCGTCCCGACAACTTCGTCTTCGGCCAGTCTGGTGCCGGAAACAACTGG
GCGAAGGGTCATTACACTGAGGGTGCCGAGCTGGTTGACAACGTTCTTGATGTTGTTCGTCGTGAG
GCTGAGGGCTGCGATTGCCTCCAGGGTTTCCAGATCACCCACTCTCTCGGTGGTGGTACCGGTGCC
GGTATGGGTACCTTGTTGATCTCTAAGATCCGCGAGGAGTTCCCCGACCGAATGATGGCCACCTTC
TCCGTCGTTCCCTCCCCTAAGGTTTCCGACACCGTCGTCGAGCCTTACAACGCCACTCTCTCAGTTC
ACCANTNGGTTGAGAACTCCGATGAGACCTTCTGTATCGACAACGAAGCTCTGTACGACATCTGCA
TGCGTACTCTCAAGCTGTCCAACCCCTCTTACGGCGACATGAACCACCTCGTTTNTGCCGTCATGTC
TGGTGTCACCACTTGCTTGCGTTTCCCTGGTCAGCTTAACTCTGACTTGCGAAAGYTTGCCGTCAAC
ATGGTTCCTTTCCCTCGTCTTCATTTCTTCATGGTCGGCTTCGCTCCCTTGACCAGCCGTGGCGCCTA
CTCCTTCCGCGCCGTTACCGTCCCCGAGTTGACCCAGCAGATGTTCGACCCTAAGAACATGATGGC

CGCTTCTGACTTCCGTAACGGTCGTTACCTGACGTGCTCTGCCATCTTGTAAGATAACATCCTATTG
ATCCTTCTAGATAT---TGTG----------------------------------

>280

-CATTACTGAGTTATAAAAAACTCCAACCCTATGTGAA-
CCTACCCTGTTTCCTCCGGCGTACCGCGCGCCTATCCGGGGGCTACCT--
GTGCCGCCTACCCTTCGGGGTACCAGCCTGTAGCCTTCTCGCAAGTGTGCACCAACCCTTTTTTAGG
GGTC--TGAACTCTA-TCCTCGTGGT--
AACTAAAGTTTTTTAACTAAATACTTTAAAACTTTCAACAACGGATCTCTTGGTTCTGGCATCGAT
GAAGAACGCAGCGAAATGCGATAAGTAATGTGAATTGCAGAATTCAGTGAATCATCGAATCTTTG
AACGCACATTGCGCCCATTAGTATTCTAGTGGGCATGCCTATTCGAGCGTCATTACAACCCTTAAG-
CCCTGTAGCTTAGCGTTGGGAATCTACGCCCCGGGGCGCAGTTCCTTAAATTTAGTGGCGGAGTTA
TAGCATACCCCAAGCGCAGTAGTTTAGCTCGCTTTCAGGGAGGCTGTAGCTGCCTGCCGTAAAACC
CCCTATACTTATAGTGGTTGACCTCGGATTAGGTAGGAATACCCGCTGAACTTAAGCATATGAAGC
GGCAAGAGCTCAAA-TTTGAAATCTGGCC----CTA-----
GCGGTCCGAGTTGTAATTTGCAGAGGATGCTTTTGGTGCGGT-
GCCTTCCGAGTTCCCTGGAACGGGACGCCATAGAGGGTGAGAGCCCCGTACGGTTGGA-
CACCTAGCCTCTATAAAGCTCCTTCGACGAGTCGAGTAGTTTGGGAATGCTGCTCTAAATGGGAGG
TAAATTTCTTCTAAAGCTAAATACCGGCCAGAGACC-
GATAGCGCACAAGTAGAGTGATCGAAAGATGAAAAGCACTTTGAAAAGAGGGTTAAATAGCACGT
GAAATTGTTGAAAGGGAAGCG-TTTGCGACCAGACCTTCTCCGGGGGGATCAACCGCTGTTC--
TCAGCGGTGCACTCCCCCCGG--TTTAGGCCAGCATCGGTTCT---
CTCAGGGGGATAAAGGCCTGGGGAACGTAGCTC----TTTAGGGAGTGTT-
ATAGCCCCTGGCGCAATACCCTTCG-GGGGACCGAGGACCGCGCT-CCG---
GCAAGGATGCTGGCGTAATGGTCGTCAACGACCCGTCTTGAAACACGGACCAAGGAGTCGAACAT
TTGTGCGAGTGTTTGGGTG--TTAAACCCTCACGCGTAATGAAAGTGAACGGAGGTGAGAGCCTTT---
-A-G---GGGTGCATCATCGACCGATCCTGATG-
TCTTCGGATGGATTTGAGTAAGAGCATAACTGTTCGGACCCGAAAGATGGTGAACTATGCGTGGAT
AGGGTGAAGCCAGAGGAAACTCTGGTGGAGGCTCGCAGCGGTTCTGACGTGCAAATCGATCGTCA
AATCTGCGCATGGGGGCGAAAGACTTATCGA-
ACCATCTAGTAGCTGGTTACCGCCGAAGTTTCCCTCAGGATAGCAGTGT--TG-
TCTTCAGTTTTATGAGGTAAAGCGAATGATTAGGGACTCGGGGGCGCTATATTGCCTTCATCCATTC
TCAAACTTTAAATATGTAAGAAGCCCTTGTTACTTAATTGAACGTGGGCATTCGAATGTACCAACA
CTAGTGGGCCATTTTTGGTAAGCAGAACTGGCGATGCGGGATGAACCGAACGCGGGGTTAAGGTG
CCAGAGTGGACGCTCATCAGACACCACAAAAGGTGTTAGTACATCTTGACAGCAGGACGGTGGCC
ATGGAAGTCGGAATCCGCTAAGGACTGTGTAACAACTCACCTGCCGAATGTACTAGCCCTGAAAAT
GGATGGCGCTCAAGCGTCT-
CACCCATACCTCGCCCTTAGGGTAGAAACGATGCCCTAAGGGCGCTGCATCGAAGGTGGCAGGCG
GTTCCAGATCGAGCTTGCCGCCAAGCCCGCCATCATCACCAACGGTCTAAAATACTCGCTCGCCAC
GGGAAATTGGGGCGACCAGAAGAAGGCCATGAGCTCGACCGCGGGCGTGTCCCAGGTCCTGAACC
GATACACCT------TCTCGTCGA---
CTCTCTCTCACTTGCGACGGACGAACACGCCCATCGGGAGGGACGGCAAGCTAGCCAAGCCGCGG
CAGCTTCACAACACCCACTGGGGTCTCGTCTGCCCGGCGGAGACTCCCGAAGGCCAGGCTTGCGGC
CTGGTCAAGAACCTGTCCCTGATGTGCTCTATCAGCGTGGGAACGTCGACGGACCCTATAGTGGAC
TACATGATTACTAGGAACATGGAGGTCCTGGAGGAGTACGAGCCGATGAGGTACCCCAACGCCAC
CAAGATCTTCCTCAACGGTTCCTGGATCGGCGTGCACCATGACCCCAAGAACCTCGTCAGGGACGT
TCAGGCGCTTCGTCGGACCAATCAGATTCCCGCCGAGGTATCGCTGGTCCGCGACATCCGAGATCG
CGAATTCAAGATCTTCTCTGACGCCGGTCGCGTGATGCGTCCCCTGTTCCGCGTGCAGCAGGAAGA
T---
ATCCAGGAGCAGGGGATCGAGAAGGGCACGCTGGCCCTCACCAAGCAGATGATCAAGCGTCTGGA
GGCGGACGTCGACATGGACCCGGAGAGCGAGGCGTACTTTGGCTGGCA---------------------------------------
----

CCCCTGAACGCGTCCTCCAAACCCCTTGATTCTTGCCCCTCACGCACAAAAAAC---
ACCACAACATCGCACTCTTTCCTGTGCAACGCTAGCATCAAATTAGCCCACGATGATGTTATGAATT
ACAGCTAACCATGTTTTTTCATCCCAACAGGTTCACCTCCAGACCGGCCAGTGCGTAAGTACTATA
GC----TACAATCACCGACGAAGAATCGCAACAGAATATAGCGGGGCTCACACGATGAT-

ATAGGGTAACCAAATTGGTGCTGCTTTCTGGCAAACCATCTCTGGCGAGCACGGTCTCGACAGCAA
TGGCGTGTAAGTATCTGGATTGCCAATTCCGACGCCAGCAAAAATAACTGACCACCAATAAATAGC
TACAACGGAACCTCGGAGCTCCAGCTCGAACGCATGAGCGTTTACTTCAACGAGGTACGCAACCAG
GGCAACAGGTAGCAAACATTCAAAAATAGTTACTAATCACCCCAACATGCACAGGCATCTGGCAA
CAAGTATGTTCCCCGCGCCGTCCTCGTCGATCTCGAGCCGGGTACTATGGACGCCGTCCGTGCTGG
CCCCTTCGGCCAACTTTTCCGTCCCGACAACTTCGTCTTCGGCCAGTCTGGTGCCGGAAACAACTGG
GCGAAGGGTCATTACACTGAGGGTGCCGAGCTGGTTGACAACGTTCTTGATGTTGTTCGTCGTGAG
GCTGA--

>485

-CATTACTGAGTTATAAAAAACTCCAACCCTATGTGAA-
CCTACCCTGTTTCCTCCGGCGTACCGCGCGCCTATCCGGGGGCTACCT--
GTGCCGCCTACCCTTCGGGGTACCAGCCTGTAGCCTTCTCGCAAGTGTGCACCAACCCTTTTTAGG
GGTC--TGAACTCTA-TCCTCGTGGT--
AACTAAAGTTTTTTAACTAAATACTTTAAAACTTTCAACAACGGATCTCTTGGTTCTGGCATCGAT
GAAGAACGCAGCGAAATGCGATAAGTAATGTGAATTGCAGAATTCAGTGAATCATCGAATCTTTG
AACGCACATTGCGCCCATTAGTATTCTAGTGGGCATGCCTATTCGAGCGTCATTACAACCCTTAAG-
CCCTGTAGCTTAGCGTTGGGAATCTACGCCCCGGGGCGCAGTTCCTTAAATTTAGTGGCGGAGTTA
TAGCATACCCCAAGCGCAGTAGTTTAGCTCGCTTTCAGGGAGGCTGTAGCTGCCTGCCGTAAAACC
CCCTATACTTATAGTGGTTGACCTCGGATTAGGTAGGAATACCCGCTGAACTTAAGCATATGAAGC
GGCAAGAGCTCAAA-TTTGAAATCTGGCC----CTA-----
GCGGTCCGAGTTGTAATTTGCAGAGGATGCTTTTGGTGCGGT-
GCCTTCCGAGTTCCCTGGAACGGGACGCCATAGAGGGTGAGAGCCCCGTACGGTTGGA-
CACCTAGCCTCTATAAAGCTCCTTCGACGAGTCGAGTAGTTTGGGAATGCTGCTCTAAATGGGAGG
TAAATTTCTTCTAAAGCTAAATACCGGCCAGAGACC-
GATAGCGCACAAGTAGAGTGATCGAAAGATGAAAAGCACTTTGAAAAGAGGGTTAAATAGCACGT
GAAATTGTTGAAAGGGAAGCG-TTTGCGACCAGACCTTCTCCGGGGGGATCAACCGCTGTTC--
TCAGCGGTGCACTCCCCCCGG--TTTAGGCCAGCATCGGTTCT---
CTCAGGGGGATAAAGGCCTGGGGAACGTAGCTC----TTTAGGGAGTGTT-
ATAGCCCCTGGCGCAATACCCTTCG-GGGGACCGAGGACCGCGCT-CCG---
GCAAGGATGCTGGCGTAATGGTCGTCAACGACCCGTCTTGAAACACGGACCAAGGAGTCGAACAT
TTGTGCGAGTGTTTGGGTG--TTAAACCCTCACGCGTAATGAAAGTGAACGGAGGTGAGAGCCTTT---
-A-G---GGGTGCATCATCGACCGATCCTGATG-
TCTTCGGATGGATTTGAGTAAGAGCATAACTGTTCGGACCCGAAAGATGGTGAACTATGCGTGGAT
AGGGTGAAGCCAGAGGAAACTCTGGTGGAGGCTCGCAGCGGTTCTGACGTGCAAATCGATCGTCA
AATCTGCGCATGGGGGCGAAAGACTTATCGA-
ACCATCTAGTAGCTGGTTACCGCCGAAGTTTCCCTCAGGATAGCAGTGT--TG-
TCTTCAGTTTTATGAGGTAAAGCGAATGATTAGGGACTCGGGGGCGCTATATTGCCTTCATCCATTC
TCAAACTTTAAATATGTAAGAAGCCCTTGTTACTTAATTGAACGTGGGCATTCGAATGTACCAACA
CTAGTGGGCCATTTTTGGTAAGCAGAACTGGCGATGCGGGATGAACCGAACGCGGGGTTAAGGTG
CCAGAGTGGACGCTCATCAGACACCACAAAAGGTGTTAGTACATCTTGACAGCAGGACGGTGGCC
ATGGAAGTCGGAATCCGCTAAGGACTGTGTAACAACTCACCTGCCGAATGTACTAGCCCTGAAAAT
GGATGGCGCTCAAGCGTCT-
CACCCATACCTCGCCCTTAGGGTAGAAACGATGCCCTAAGGGCGCTGCATCGAAGGTGGCAGGCG
GTTCCAGATCGAGCTTGCCGCCAAGCCCGCCATCATCACCAACGGTCTAAAATACTCGCTCGCCAC
GGGAAATTGGGGCGACCAGAAGAAGGCCATGAGCTCGACCGCGGGCGTGTCCCAGGTCCTGAACC
GATACACCT------TCTCGTCGA---
CTCTCTCTCACTTGCGACGGACGAACACGCCCATCGGGAGGGACGGCAAGCTAGCCAAGCCGCGG
CAGCTTCACAACACCCACTGGGGTCTCGTCTGCCCGGCGGAGACTCCCGAAGGCCAGGCTTGCGGC
CTGGTCAAGAACCTGTCCCTGATGTGCTCTATCAGCGTGGGAACGTCGACGGACCCTATAGTGGAC
TACATGATTACTAGGAACATGGAGGTCCTGGAGGAGTACGAGCCGATGAGGTACCCCAACGCCAC
CAAGATCTTCCTCAACGGTTCCTGGATCGGCGTGCACCATGACCCCAAGAACCTCGTCAGGGACGT
TCAGGCGCTTCGTCGGACCAATCAGATTCCCGCCGAGGTATCGCTGGTCCGCGACATCCGAGATCG

CGAATTCAAGATCTTCTCTGACGCCGGTCGCGTGATGCGTCCCCTGTTCCGCGTGCAGCAGGAAGA
T--
ATCCAGGAGCAGGGGATCGAGAAGGGCACGCTGGCCCTCACCAAGCAGATGATCAAGCGTCTGGA
GGCGGACGTCGACATGGACCCGGAGAGCGAGGCGTACTTTGGCTGGCAGGGGCTGGTGAACGAAG
GCGTCATCGAGTTCCTCGACGCGGAGGAGGAGGAGACAGCCATGATCTGCATGACGCCCGAAGAC
TTGGACAATTATCGCATGGCTAAGCTCGGGTACGACGTGTCGCAGGACAACGGC------
GACGAGGTGAACAAGCGGCTGAAGACGAAGGTGAATCCGTCGACGCACATGTACACCCACTGCGA
GATCCACCCCAGCATGCTCCTGGGAATCTGCGCGAGCATCATCCCTTTCCCGGACCACAATCAGGT
A--

AATATAGCGGGGCTCACACGATGAT-
ATAGGGTAACCAAATTGGTGCTGCTTTCTGGCAAACCATCTCTGGCGAGCACGGTCTCGACAGCAA
TGGCGTGTAAGTATCTGGATTGCCAATTCCGACGCCAGCAAAAATAACTGACCACCAATAAATAGC
TACAACGGAACCTCGGAGCTCCAGCTCGAACGCATGAGCGTTTACTTCAACGAGGTACGCAACCAG
GGCAACAGGTAGCAAACATTCAAAAATAGTTACTAATCACCCCAACATGCACAGGCATCTGGCAA
CAAGTATGTTCCCCGCGCCGTCCTCGTCGATCTCGAGCCGGGTACTATGGACGCCGTCCGTGCTGG
CCCCTTCGGCCAACTTTTCCGTCCCGACAACTTCGTCTTCGGCCAGTCTGGTGCCGGAAACAACTGG
GCGAAGGGTCATTACACTGAGGGTGCCGAGCTGGTTGACAACGTTCTTGATGTTGTTCGTCGTGAG
GCTGANGGCTGCGATTGCCTCCAGGGTTTCCAGATCACCCACTCTCTCGGTGGTGGTACCGGTGCC
GGTATGGGTACCTTGTTGATCTCTAAGATCCGCGAAGAGTTCCCCGACCGAATGATGGCCACCTTC
TCCGTCGTTCCCTCCCCTAAGGTTTCCGACACCGTCGTCGAGCCTTACAACGCCACTCTCTCAGTTC
ACCAGCTGGTTGAGAACTCCGATGAGACCTTCTGTATCGACAACGAAGCTCTGTACGACATCTGCA
TGCGTACACTCAAGCTGTCCAACCCCTCTTACGGCGACCTGAACCACCTCGTCTCTGCCGTCATGTC
TGGTGTCACCACTNGCTTGCGTTTCCCTGGTCAGCTTAACTCTGACTTGCGAAAGCTTGCCGTCAAC
ATGGTTCCTTTCCCTCGTCTTCATTTCTTCATGGTCGGCTTCGCTCCCTTGACCAGCCGTGGCGCCTA
CTCCTTCCGCGCCGTTACCGTCCCCGAGTTG---

>318

-CATTACTGAGTTATAAAAAACTCCAACCCTATGTGAA-
CCTACCCTGTTTCCTCCGGCGTACCGCGCGCCTATCCGGGGGCTACCT--
GTGCCGCCTACCCTTCGGGGTACCAGCCTGTAGCCTTCTCGCAAGTGTGCACCAACCCTTTTTTAGG
GGTC--TGAACTCTA-TCCTCGTGGT--
AACTAAAGTTTTTTTAACTAAATACTTTAAAACTTTCAACAACGGATCTCTTGGTTCTGGCATCGAT
GAAGAACGCAGCGAAATGCGATAAGTAATGTGAATTGCAGAATTCAGTGAATCATCGAATCTTTG
AACGCACATTGCGCCCATTAGTATTCTAGTGGGCATGCCTATTCGAGCGTCATTACAACCCTTAAG-
CCCTGTAGCTTAGCGTTGGGAATCTACGCCCCGGGGCGCAGTTCCTTAAATTTAGTGGCGGAGTTA
TAGCATACCCCAAGCGCAGTAGTTTAGCTCGCTTTCAGGGAGGCTGTAGCTGCCTGCCGTAAAACC
CCCTATACTTATAGTGGTTGACCTCGGATTAGGTAGGAATACCCGCTGAACTTAAGCATATGAAGC
GGCAAGAGCTCAAA-TTTGAAATCTGGCC----CTA-----
GCGGTCCGAGTTGTAATTTGCAGAGGATGCTTTTGGTGCGGT-
GCCTTCCGAGTTCCCTGGAACGGGACGCCATAGAGGGTGAGAGCCCCGTACGGTTGGA-
CACCTAGCCTCTATAAAGCTCCTTCGACGAGTCGAGTAGTTTGGGAATGCTGCTCTAAATGGGAGG
TAAATTTCTTCTAAAGCTAAATACCGGCCAGAGACC-
GATAGCGCACAAGTAGAGTGATCGAAAGATGAAAAGCACTTTGAAAAGAGGGTTAAATAGCACGT
GAAATTGTTGAAAGGGAAGCG-TTTGCGACCAGACCTTCTCCGGGGGGATCAACCGCTGTTC--
TCAGCGGTGCACTCCCCCCGG--TTTAGGCCAGCATCGGTTCT---
CTCAGGGGGATAAAGGCCTGGGGAACGTAGCTC----TTTAGGGAGTGTT-
ATAGCCCCTGGCGCAATACCCTTCG-GGGGACCGAGGACCGCGCT-CCG---
GCAAGGATGCTGGCGTAATGGTCGTCAACGACCCGTCTTGAAACACGGACCAAGGAGTCGAACAT
TTGTGCGAGTGTTTGGGTG--TTAAACCCTCACGCGTAATGAAAGTGAACGGAGGTGAGAGCCTT----
-A-----GGGTGCATCATCGACCGATCCTGATG-
TCTTCGGATGGATTTGAGTAAGAGCATAACTGTTCGGACCCGAAAGATGGTGAACTATGCGTGGAT
AGGGTGAAGCCAGAGGAAACTCTGGTGGAGGCTCGCAGCGGTTCTGACGTGCAAATCGATCGTCA
AATCTGCGCATGGGGGCGAAAGACTTATCGA-
ACCATCTAGTAGCTGGTTACCGCCGAAGTTTCCCTCAGGATAGCAGTGT--TG-
TCTTCAGTTTTATGAGGTAAAGCGAATGATTAGGGACTCGGGGGCGCTATATTGCCTTCATCCATTC

TCAAACTTTAAATATGTAAGAAGCCCTTGTTACTTAATTGAACGTGGGCATTCGAATGTACCAACA
CTAGTGGGCCATTTTTGGTAAGCAGAACTGGCGATGCGGGATGAACCGAACGCGGGGTTAAGGTG
CCAGAGTGGACGCTCATCAGACACCACAAAAGGTGTTAGTACATCTTGACAGCAGGACGGTGGCC
ATGGAAGTCGGAATCCGCTAAGGACTGTGTAACAACTCACCTGCCGAATGTACTAGCCCTGAAAAT
GGATGGCGCTCAAGCGTCT-
CACCCATACCTCGCCCTTAGGGTAGAAACGATGCCCTAAGGGCGCTGCATCGAAGGTGGCAGGCG
GTTCCAGATCGAGCTTGCCGCCAAGCCCGCCATCATCACCAACGGTCTAAAATACTCGCTCGCCAC
GGGAAATTGGGGCGACCAGAAGAAGGCCATGAGCTCGACCGCGGGCGTGTCCCAGGTCCTGAACC
GATACACCT------TCTCGTCGA---
CTCTCTCTCACTTGCGACGGACGAACACGCCCATCGGGAGGGACGGCAAGCTAGCCAAGCCGCGG
CAGCTTCACAACACCCACTGGGGTCTCGTCTGCCCGGCGGAGACTCCCGAAGGCCAGGCTTGCGGC
CTGGTCAAGAACCTGTCCCTGATGTGCTCTATCAGCGTGGGAACGTCGACGGACCCTATAGTGGAC
TACATGATTACTAGGAACATGGAGGTCCTGGAGGAGTACGAGCCGATGAGGTACCCCAACGCCAC
CAAGATCTTCCTCAACGGTTCCTGGATCGGCGTGCACCATGACCCCAAGAACCTCGTCAGGGACGT
TCAGGCGCTTCGTCGGACCAATCAGATTCCCGCCGAGGTATCGCTGGTCCGCGACATCCGAGATCG
CGAATTCAAGATCTTCTCTGACGCCGGTCGCGTGATGCGTCCCCTGTTCCGCGTGCAGCAGGAAGA
T---
ATCCAGGAGCAGGGGATCGAGAAGGGCACGCTGGCCCTCACCAAGCAGATGATCAAGCGTCTGGA
GGCGGACGTCGACATGGACCCGGAGAGCGAGGCGTACTTTGGCTGGCAGGGGCTGGTGAACGAAG
GCGTCATCGAGTTCCTCGACGCGGAGGAGGAGGAGACAGCCATGATCTGCATGACGCCCGAAGAC
TTGGACAATTATCGCATGGCTAAGCTCGGGTACGACGTGTCGCAGGACAACGGC------
GACGAGGTGAACAAGCGGCTGAAGACGAAGGTGAATCCGTCGACGCACATGTACACCCACTGCGA
GATCCACCCCAGCATGCTCCTGGGAATCTGCGCGAGCATCATCCCTTTCCCGGACCACAATCAGGT
ACCCCTGAACGCGTCCTCCAAACCCCTTGATTCTTGCCCCTCACGCACAAAAAAC---
ACCACAACATCGCACTCTTTCCTGTGCAACGCTAGCATCAAATTAGCCCACGATGATGTTATGAATT
ACAGCTAACCATGTTTTTTCATCCCAACAGGTTCACCTCCAGACCGGCCAGTGCGTAAGTACTATA
GC----TACAATCACCGACGAAGAATCGCAACAGAATATAGCGGGGCTCACACGATGAT-
ATAGGGTAACCAAATTGGTGCTGCTTTCTGGCAAACCATCTCTGGCGAGCACGGTCTCGACAGCAA
TGGCGTGTAAGTATCTGGATTGCCAATTCCGACGCCAGCAAAAATAACTGACCACCAATAAATAGC
TACAACGGAACCTCGGAGCTCCAGCTCGAACGCATGAGCGTTTACTTCAACGAGGTACGCAACCAG
GGCAACAGGTAGCAAACATTCAAAAATAGTTACTAATCACCCCAACATGCACAGGCATCTGGCAA
CAAGTATGTTCCCCGCGCCGTCCTCGTCGATCTCGAGCCGGGTACTATGGACGCCGTCCGTGCTGG
CCCCTTCGGCCAACTTTTCCGTCCCGACAACTTCGTCTTCGGCCAGTCTGGTGCCGGAAACAACTGG
GCGAAGGGTCATTACACTGAGGGTGCCGAGCTGGTTGACAACGTTCTTGATGTTGTTCGTCGTGAG
GCTGAGGGCTGCGATTGCCTCCAGGGTTTCCAGATCACCCACTCTCTCGGTGGTGGTACCGGTGCC
GGTATGGGTACCTTGTTGATCTCTAAGATCCGCGAAGAGTTCCCCGACCGAATGATGGCCACCTTC
TCCGTCGTTCCCTCCCCTAAGGTTTCCGACACCGTCGTCGAGCCTTACAACGCCACTCTCTCAGTTC
ACCAGCTGGTTGAGAACTCCGATGAGACCTTCTGTATCGACAACGAAGCTCTGTACGACATCTGCA
TGCGTACACTCAAGCTGTCCAACCCCTCTTACGGCGACCTGAACCACCTCGTCTCNGCCGTCATGTC
TGGTGTCACCACTTGCTTGCGTTTCCCTGGTCAGCTTAACTCTGACTTGCGAAAGCTTGCCGTCAAC
ATGGTTCCTTTCCCTCGTCTTCATTTCTTCATGGTCGGCTTCGCTCCCTTGACCAGCCGTGGCGCCTA
CTCCTTCCGCGCCGTTACCGTCCCCGAGTTGACCCAGCAGATGTTCGACCCTAAGAACATGATGGC
CGCTTCTGACTTCCGTAACGGTCGTTACCTGACGTGCTCTGCCATCTTGTAAGATAACATCCTACTG
ATCCTTCT---

>396

-CATTACTGAGTTATAAAAAACTCCAACCCTATGTGAA-
CCTACCCTGTTTCCTCCGGCGTACCGCGCGCCTATCCGGGGGCTACCT--
GTGCCGCCTACCCTTCGGGGTACCAGCCTGTAGCCTTCTCGCAAGTGTGCACCAACCCTTTTTAGG
GGTC--TGAACTCTA-TCCTCGTGGT--
AACTAAAGTTTTTTTAACTAAATACTTTAAAACTTTCAACAACGGATCTCTTGGTTCTGGCATCGAT
GAAGAACGCAGCGAAATGCGATAAGTAATGTGAATTGCAGAATTCAGTGAATCATCGAATCTTTG
AACGCACATTGCGCCCATTAGTATTCTAGTGGGCATGCCTATTCGAGCGTCATTACAACCCTTAAG-
CCCTGTAGCTTAGCGTTGGGAATCTACGCCCCGGGGCGCAGTTCCTTAAATTTAGTGGCGGAGTTA
TAGCATACCCCAAGCGCAGTAGTTTAGCTCGCTTTCAGGGAGGCTGTAGCTGCCTGCCGTAAAACC
CCCTATACTTATAGTGGTTGACCTCGGATTAGGTAGGAATACCCGCTGAACTTAA-----
TGAAGCGGCAAGAGCTCAAA-TTTGAAATCTGGCC----CTA-----

GCGGTCCGAGTTGTAATTTGCAGAGGATGCTTTTGGTGCGGT-
GCCTTCCGAGTTCCCTGGAACGGGACGCCATAGAGGGTGAGAGCCCCGTACGGTTGGA-
CACCTAGCCTCTATAAAGCTCCTTCGACGAGTCGAGTAGTTTGGGAATGCTGCTCTAAATGGGAGG
TAAATTTCTTCTAAAGCTAAATACCGGCCAGAGACC-
GATAGCGCACAAGTAGAGTGATCGAAAGATGAAAAGCACTTTGAAAAGAGGGTTAAATAGCACGT
GAAATTGTTGAAAGGGAAGCG-TTTGCGACCAGACCTTCTCCGGGGGGATCAACCGCTGTTC--
TCAGCGGTGCACTCCCCCCGG--TTTAGGCCAGCATCGGTTCT---
CTCAGGGGGATAAAGGCCTGGGGAACGTAGCTC----TTTAGGGAGTGTT-
ATAGCCCCTGGCGCAATACCCTTCG-GGGGACCGAGGACCGCGCT-CCG---
GCAAGGATGCTGGCGTAATGGTCGTCAACGACCCGTCTTGAAACACGGACCAAGGAGTCGAACAT
TTGTGCGAGTGTTTGGGTG--TTAAACCCTCACGCGTAATGAAAGTGAACGGAGGTGAGAGCCTT----
-A-----NGGTGCATCATCGACCGATCCTGATG-
TCTTCGGATGGATTTGAGTAAGAGCATAACTGTTCGGACCCGAAAGATGGTGAACTATGCGTGGAT
AGGGTGAAGCCAGAGGAAACTCTGGTGGAGGCTCGCAGCGGTTCTGACGTGCAAATCGATCGTCA
AATCTGCGCATGGGGGCGAAAGACTTATCGA-
ACCATCTAGTAGCTGGTTACCGCCGAAGTTTCCCTCAGGATAGCAGTGT--TG-
TCTTCAGTTTTATGAGGTAAAGCGAATGATTAGGGACTCGGGGGCGCTATATTGCCTTCATCCATTC
TCAAACTTTAAATATGTAAGAAGCCCTTGTTACTTAATTGAACGTGGGCATTCGAATGTACCAACA
CTAGTGGGCCATTTTTGGTAAGCAGAACTGGCGATGCGGGATGAACCGAACGCGGGGTTAAGGTG
CCAGAGTGGACGCTCATCAGACACCACAAAAGGTGTTAGTACATCTTGACAGCAGGACGGTGGCC
ATGGAAGTCGGAATCCGCTAAGGACTGTGTAACAACTCACCTGCCGAATGTACTAGCCCTGAAAAT
GGATGGCGCTCAAGCGTCT-
CACCCATACCTCGCCCTTAGGGTAGAAACGATGCCCTAAGGGCGCTGCATCGAAGGTGGCAGGCG
GTTCCAGATCGAGCTTGCCGCCAAGCCCGCCATCATCACCAACGGTCTAAAATACTCGCTCGCCAC
GGGAAATTGGGGCGACCAGAAGAAGGCCATGAGCTCGACCGCGGGCGTGTCCCAGGTCCTGAACC
GATACACCT------TCTCGTCGA---
CTCTCTCTCACTTGCGACGGACGAACACGCCCATCGGGAGGGACGGCAAGCTAGCCAAGCCGCGG
CAGCTTCACAACACCCACTGGGGTCTCGTCTGCCCGGCGGAGACTCCCGAAGGCCAGGCTTGCGGC
CTGGTCAAGAACCTGTCCCTGATGTGCTCTATCAGCGTGGGAACGTCGACGGACCCTATAGTGGAC
TACATGATTACTAGGAACATGGAGGTCCTGGAGGAGTACGAGCCGATGAGGTACCCCAACGCCAC
CAAGATCTTCCTCAACGGTTCCTGGATCGGCGTGCACCATGACCCCAAGAACCTCGTCAGGGACGT
TCAGGCGCTTCGTCGGACCAATCAGATTCCCGCCGAGGTATCGCTGGTCCGCGACATCCGAGATCG
CGAATTCAAGATCTTCTCTGACGCCGGTCGCGTGATGCGTCCCCTGTTCCGCGTGCAGCAGGAAGA
T---
ATCCAGGAGCAGGGGATCGAGAAGGGCACGCTGGCCCTCACCAAGCAGATGATCAAGCGTCTGGA
GGCGGACGTCGACATGGACCCGGAGAGCGAGGCGTACTTTGGCTGGCAGGGGCTGGTGAACGAAG
GCGTCATCGAGTTCCTCGACGCGGAGGAGGAGGAGACAGCCATGATCTGCATGACGCCCGAAGAC
TTGGACAATTATCGCATGGCTAAGCTCGGGTACGACGTGTCGCAGGACAACGGC------
GACGAGGTGAACAAGCGGCTGAAGACGAAGGTGAATCCGTCGACGCACATGTACACCCACTGCGA
GATCCACCCCAGCATGCTCCTGGGAATCTGCGCGAGCATCATCCCTTTCCCGGACCACAATCAGGT
A---
CCTGTGCAACGCTAGCATCAAATTAGCCCACGATGATGTTATGAATTACAGCTAACCATGTTTTTC
ATCCCAACAGGTTCACCTCCAGACCGGCCAGTGCGTAAGTACTATAGC----
TACAATCACCGACGAAGAATCGCAACAGAATATAGCGGGGCTCACACGATGAT-
ATAGGGTAACCAAATTGGTGCTGCTTTCTGGCAAACCATCTCTGGCGAGCACGGTCTCGACAGCAA
TGGCGTGTAAGTATCTGGATTGCCAATTCCGACGCCAGCAAAAATAACTGACCACCAATAAATAGC
TACAACGGAACCTCGGAGCTCCAGCTCGAACGCATGAGCGTTTACTTCAACGAGGTACGCAACCAG
GGCAACAGGTAGCAAACATTCAAAAATAGTTACTAATCACCCCAACATGCACAGGCATCTGGCAA
CAAGTATGTTCCCCGCGCCGTCCTCGTCGATCTCGAGCCGGGTACTATGGACGCCGTCCGTGCTGG
CCCCTTCGGCCAACTTTTCCGTCCCGACAACTTCGTCTTCGGCCAGTCTGGTGCCGGAAACAACTGG
GCGAAGGGTCATTACACTGAGGGTGCCGAGCTGGTTGACAACGTTCTTGATGTTGTTCGTCGTGAG
GCTGAGGGCTGCGATTGCCTCCAGGGTTTCCAGATCACCCACTCTCTCGGTGGTGGTACCGGTGCC
GGTATGGGTACCTTGTTGATCTCTAAGATCCGCGAAGAGTTCCCCGACCGAATGATGGCCACCTTC
TCCGTCGTTCCCTCCCCTAAGGTTTCCGACACCGTCGTCGAGCCTTACAACGCCACTCTCTCAGTTC
ACCAGCTGGTTGAGAACTCCGATGAGACCTTCTGTATCGACAACGAAGCTCTGTACGACATCTGCA
TGCGTACACTCAAGCTGTCCAACCCCTCTTACGGCGACCTGAACCACCTCGTCTCTGCCGTCATGTC
TGGTGTCACCACTTGCTTGCGTTTCCCTGGTCAGCTTAACTCTGACTTGCGAAAGCTTGCCGTCAAC

ATGGTTCCTTTCCCTCGTCTTCATTTCTTCATGGTCGGCTTCGCTCCCTTGACCAGCCGTGGCGCCTA
CTCCTTCCGCGCCGTTACCGTCCCCGAGTTG--

>465

-CATTACTGAGTTATAAAAAACTCCAACCCTATGTGAA-
CCTACCCTGTTTCCTCCGGCGTACCGCGCGCCTATCCGGGGGCTACCT--
GTGCCGCCTACCCTTCGGGGTACCAGCCTGTAGCCTTCTCGCAAGTGTGCACCAACCCTTTTTAGG
GGTC--TGAACTCTA-TCCTCGTGGT--
AACTAAAGTTTTTTAACTAAATACTTTAAAACTTTCAACAACGGATCTCTTGGTTCTGGCATCGAT
GAAGAACGCAGCGAAATGCGATAAGTAATGTGAATTGCAGAATTCAGTGAATCATCGAATCTTTG
AACGCACATTGCGCCCATTAGTATTCTAGTGGGCATGCCTATTCGAGCGTCATTACAACCCTTAAG-
CCCTGTAGCTTAGCGTTGGGAATCTACGCCCCGGGGCGCAGTTCCTTAAATTTAGTGGCGGAGTTA
TAGCATACCCCAAGCGCAGTAGTTTAGCTCGCTTTCAGGGAGGCTGTAGCTGCCTGCCGTAAAACC
CCCTATACTTATAGTGGTTGACCTCGGATTAGGTAGGAATACCCGCTGAACTTAAGCATATGAAGC
GGCAAGAGCTCAAA-TTTGAAATCTGGCC----CTA-----
GCGGTCCGAGTTGTAATTTGCAGAGGATGCTTTTGGTGCGGT-
GCCTTCCGAGTTCCCTGGAACGGGACGCCATAGAGGGTGAGAGCCCCGTACGGTTGGA-
CACCTAGCCTCTATAAAGCTCCTTCGACGAGTCGAGTAGTTTGGGAATGCTGCTCTAAATGGGAGG
TAAATTTCTTCTAAAGCTAAATACCGGCCAGAGACC-
GATAGCGCACAAGTAGAGTGATCGAAAGATGAAAAGCACTTTGAAAAGAGGGTTAAATAGCACGT
GAAATTGTTGAAAGGGAAGCG-TTTGCGACCAGACCTTCTCCGGGGGGATCAACCGCTGTTC--
TCAGCGGTGCACTCCCCCCGG--TTTAGGCCAGCATCGGTTCT---
CTCAGGGGGATAAAGGCCTGGGGAACGTAGCTC----TTTAGGGAGTGTT-
ATAGCCCCTGGCGCAATACCCTTCG-GGGGACCGAGGACCGCGCT-CCG---
GCAAGGATGCTGGCGTAATGGTCGTCAACGACCCGTCTTGAAACACGGACCAAGGAGTCGAACAT
TTGTGCGAGTGTTTGGGTG--TTAAACCCTCACGCGTAATGAAAGTGAACGGAGGTGAGAGCCTTT---
-A-G---GGGTGCATCATCGACCGATCCTGATG-
TCTTCGGATGGATTTGAGTAAGAGCATAACTGTTCGGACCCGAAAGATGGTGAACTATGCGTGGAT
AGGGTGAAGCCAGAGGAAACTCTGGTGGAGGCTCGCAGCGGTTCTGACGTGCAAATCGATCGTCA
AATCTGCGCATGGGGGCGAAAGACTTATCGA-
ACCATCTAGTAGCTGGTTACCGCCGAAGTTTCCCTCAGGATAGCAGTGT--TG-
TCTTCAGTTTTATGAGGTAAAGCGAATGATTAGGGACTCGGGGGCGCTATATTGCCTTCATCCATTC
TCAAACTTTAAATATGTAAGAAGCCCTTGTTACTTAATTGAACGTGGGCATTCGAATGTACCAACA
CTAGTGGGCCATTTTTGGTAAGCAGAACTGGCGATGCGGGATGAACCGAACGCGGGGTTAAGGTG
CCAGAGTGGACGCTCATCAGACACCACAAAAGGTGTTAGTACATCTTGACAGCAGGACGGTGGCC
ATGGAAGTCGGAATCCGCTAAGGACTGTGTAACAACTCACCTGCCGAATGTACTAGCCCTGAAAAT
GGATGGCGCTCAAGCGTCT-
CACCCATACCTCGCCCTTAGGGTAGAAACGATGCCCTAAGGGCGCTGCATCGAAGGTGGCAGGCG
GTTCCAGATCGAGCTTGCCGCCAAGCCCGCCATCATCACCAACGGTCTAAAATACTCGCTCGCCAC
GGGAAATTGGGGCGACCAGAAGAAGGCCATGAGCTCGACCGCGGGCGTGTCCCAGGTCCTGAACC
GATACACCT------TCTCGTCGA---
CTCTCTCTCACTTGCGACGGACGAACACGCCCATCGGGAGGGACGGCAAGCTAGCCAAGCCGCGG
CAGCTTCACAACACCCACTGGGGTCTCGTCTGCCCGGCGGAGACTCCCGAAGGCCAGGCTTGCGGC
CTGGTCAAGAACCTGTCCCTGATGTGCTCTATCAGCGTGGGAACGTCGACGGACCCTATAGTGGAC
TACATGATTACTAGGAACATGGAGGTCCTGGAGGAGTACGAGCCGATGAGGTACCCCAACGCCAC
CAAGATCTTCCTCAACGGTTCCTGGATCGGCGTGCACCATGACCCCAAGAACCTCGTCAGGGACGT
TCAGGCGCTTCGTCGGACCAATCAGATTCCCGCCGAGGTATCGCTGGTCCGCGACATCCGAGATCG
CGAATTCAAGATCTTCTCTGACGCCGGTCGCGTGATGCGTCCCCTGTTCCGCGTGCAGCAGGAAGA
T--
ATCCAGGAGCAGGGGATCGAGAAGGGCACGCTGGCCCTCACCAAGCAGATGATCAAGCGTCTGGA
GGCGGACGTCGACATGGACCCGGAGAGCGAGGCGTACTTTGGCTGGCAGGGGCTGGTGAACGAAG
GCGTCATCGAGTTCCTCGACGCGGAGGAGGAGGAGACAGCCATGATCTGCATGACGCCCGAAGAC
TTGGACAATTATCGCATGGCTAAGCTCGGGTACGACGTGTCGCAGGACAACGGC------
GACGAGGTGAACAAGCGGCTGAAGACGAAGGTGAATCCGTCGACGCACATGTACACCCACTGCGA
GATCCACCCCAGCATGCTCCTGGGAATCTGCGCGAGCATCATCCCTTTCCCGGACCACAATCAGGT
ACCCCTGAACGCGTCCTCCAAACCCCTTGATTCTTGCCCCTCACGCACAAAAAAC---

ACCACAACATCGCACTCTTTCCTGTGCAACGCTAGCATCAAATTAGCCCACGATGATGTTATGAATT
ACAGCTAACCATGTTTTTTCATCCCAACAGGTTCACCTCCAGACCGGCCAGTGCGTAAGTACTATA
GC----TACAATCACCGACGAAGAATCGCAACAGAATATAGCGGGGCTCACACGATGAT-
ATAGGGTAACCAAATTGGTGCTGCTTTCTGGCAAACCATCTCTGGCGAGCACGGTCTCGACAGCAA
TGGCGTGTAAGTATCTGGATTGCCAATTCCGACGCCAGCAAAAATAACTGACCACCAATAAATAGC
TACAACGGAACCTCGGAGCTCCAGCTCGAACGCATGAGCGTTTACTTCAACGAGGTACGCAACCAG
GGCAACAGGTAGCAAACATTCAAAAATAGTTACTAATCACCCCAACATGCACAGGCATCTGGCAA
CAAGTATGTTCCCCGCGCCGTCCTCGTCGATCTCGAGCCGGGTACTATGGACGCCGTCCGTGCTGG
CCCCTTCGGCCAACTTTTCCGTCCCGACAACTTCGTCTTCGGCCAGTCTGGTGCCGGAAACAACTGG
GCGAAGGGTCATTACACTGAGGGTGCCGAGCTGGTTGACAACGTTCTTGATGTTGTTCGTCGTGAG
GCTGAgGGCTGCGATTGCCTCCAGGGTTTCCAGATCACCCACTCTCTCGGTGGTGGTACCGGTGCCG
GTATGGGTACCTTGTTGATCTCTAAGATCCGCGAAGAGTTCCCCGACCGAATGATGGCCACCTTCTC
CGTCGTTCCCTCCCCTAAGGTTTCCGACACCGTCGTCGAGCCTTACAACGCCACTCTCTCAGTTCAC
CAGCTGGTTGAGAACTCCGATGAGACCTTCTGTATCGACAACGAAGCTCTGTACGACATCTGCATG
CGTACACTCAAGCTGTCCAACCCCTCTTACGGCGACCTGAACCACCTCGTCTC----------------------------
----

>554

-CATTACTGAGTTATAAAAAACTCCAACCCTATGTGAA-
CCTACCCTGTTTCCTCCGGCGTACCGCGCGCCTATCCGGGGGCTACCT--
GTGCCGCCTACCCTTCGGGGTACCAGCCTGTAGCCTTCTCGCAAGTGTGCACCAACCCTTTTTAGG
GGTC--TGAACTCTA-TCCTCGTGGT--
AACTAAAGTTTTTTAACTAAATACTTTAAAACTTTCAACAACGGATCTCTTGGTTCTGGCATCGAT
GAAGAACGCAGCGAAATGCGATAAGTAATGTGAATTGCAGAATTCAGTGAATCATCGAATCTTTG
AACGCACATTGCGCCCATTAGTATTCTAGTGGGCATGCCTATTCGAGCGTCATTACAACCCTTAAG-
CCCTGTAGCTTAGCGTTGGGAATCTGCGCCCCGGGGCGCAGTTCCTTAAATTTAGTGGCGGAGTTA
TAGCATACCCCAAGCGCAGTAGTTTAGCTCGCTTTCAGGGAGGCTGTAGCTGCCTGCCGTAAAACC
CCCTATACTTATAGTGGTTGACCTCGGATTAGGTAGGAATACCCGCTGAACTTAAGCATATGAAGC
GGCAAGAGCTCAAA-TTTGAAATCTGGCC----CTA-----
GCGGTCCGAGTTGTAATTTGCAGAGGATGCTTTTGGTGCGGT-
GCCTTCCGAGTTCCCTGGAACGGGACGCCATAGAGGGTGAGAGCCCCGTACGGTTGGA-
CACCTAGCCTCTATAAAGCTCCTTCGACGAGTCGAGTAGTTTGGGAATGCTGCTCTAAATGGGAGG
TAAATTTCTTCTAAAGCTAAATACCGGCCAGAGACC-
GATAGCGCACAAGTAGAGTGATCGAAAGATGAAAAGCACTTTGAAAAGAGGGTTAAATAGCACGT
GAAATTGTTGAAAGGGAAGCG-TTTGCGACCAGACCTTCTCCGGGGGGATCAACCGCTGTTC--
TCAGCGGTGCACTCCCCCCGG--TTTAGGCCAGCATCGGTTCT---
CTCAGGGGGATAAAGGCCTGGGGAACGTAGCTC----TTTAGGGAGTGTT-
ATAGCCCCTGGCGCAATACCCTTCG-GGGGACCGAGGACCGCGCT-CCG---
GCAAGGATGCTGGCGTAATGGTCGTCAACGACCCGTCTTGAAACACGGACCAAGGAGTCGAACAT
TTGTGCGAGTGTTTGGGTG--TTAAACCCTCACGCGTAATGAAAGTGAACGGAGGTGAGAGCCTT----
-A-----GGGTGCATCATCGACCGATCCTGATG-
TCTTCGGATGGATTTGAGTAAGAGCATAACTGTTCGGACCCGAAAGATGGTGAACTATGCGTGGAT
AGGGTGAAGCCAGAGGAAACTCTGGTGGAGGCTCGCAGCGGTTCTGACGTGCAAATCGATCGTCA
AATCTGCGCATGGGGGCGAAAGACTTATCGA-
ACCATCTAGTAGCTGGTTACCGCCGAAGTTTCCCTCAGGATAGCAGTGT--TG-
TCTTCAGTTTTATGAGGTAAAGCGAATGATTAGGGACTCGGGGGCGCTATATTGCCTTCATCCATTC
TCAAACTTTAAATATGTAAGAAGCCCTTGTTACTTAATTGAACGTGGGCATTCGAATGTACCAACA
CTAGTGGGCCATTTTTGGTAAGCAGAACTGGCGATGCGGGATGAACCGAACGCGGGGTTAAGGTG
CCAGAGTGGACGCTCATCAGACACCACAAAAGGTGTTAGTACATCTTGACAGCAGGACGGTGGCC
ATGGAAGTCGGAATCCGCTAAGGACTGTGTAACAACTCACCTGCCGAATGTACTAGCCCTGAAAAT
GGATGGCGCTCAAGCGTCT-CACCCATACCTCGCCCTTAGGGTAGAAACGATGCCCTAAGG-----------
----

GATCGAGCTTGCCGCCAAGCCCGCCATCATCACCAACGGTCTAAAATACTCGCTCGCCACGGGAAA
TTGGGGCGACCAGAAGAAGGCCATGAGCTCGACCGCGGGCGTGTCCCAGGTCCTGAACCGATACA
CCT------TCTCGTCGA---

CTCTCTCTCACTTGCGACGGACGAACACGCCCATCGGGAGGGACGGCAAGCTAGCCAAGCCGCGG
CAGCTTCACAACACCCACTGGGGTCTCGTCTGCCCGGCGGAGACTCCCGAAGGCCAGGCTTGCGGC
CTGGTCAAGAACCTGTCCCTGATGTGCTCTATCAGCGTGGGAACGTCGACGGACCCTATAGTGGAC
TACATGATTACTAGGAACATGGAGGTCCTGGAGGAGTACGAGCCGATGAGGTACCCCAACGCCAC
CAAGATCTTCCTCAACGGTTCCTGGATCGGCGTGCACCATGACCCCAAGAACCTCGTCAGGGACGT
TCAGGCGCTTCGTCGGACCAATCAGATTCCCGCCGAGGTATCGCTGGTCCGCGACATCCGAGATCG
CGAATTCAAGATCTTCTCTGACGCCGGTCGCGTGATGCGTCCCCTGTTCCGCGTGCAGCAGGAAGA
T--
ATCCAGGAGCAGGGGATCGAGAAGGGCACGCTGGCCCTCACCAAGCAGATGATCAAGCGTCTGGA
GGCGGACGTCGACATGGACCCGGAGAGCGAGGCGTACTTTGGCTGGCAGGGGCTGGTGAACGAAG
GCGTCATCGAGTTCCTCGACGCGGAGGAGGAGGAGACAGCCATGATCTGCATGACGCCCGAAGAC
TTGGACAATTATCGCATGGCTAAGCTCGGGTACGACGTGTCGCAGGACAACGGC------
GACGAGGTGAACAAGCGGCTGAAGACGAAGGTGAATCCGTCGACGCACATGTACACCCACTGCGA
GATCCACCCCAGCATGCTCCTGGGAATCTGCGCGAGCATCATCCCTTTCCCGGACCACAATCAGGT
ACCCCTGAACGCGTCCTCCAAACCCCTTGATTCTTGCCCCTCACGCACAAAAAAC---
ACCACAACATCGCACTCTTTCCTGTGCAACGCTAGCATCAAATTAGCCCACGATGATGTTATGAATT
ACAGCTAACCATGTTTTTCATCCCAACAGGTTCACCTCCAGACCGGCCAGTGCGTAAGTACTATA
GC----TACAATCACCGACGAAGAATCGCAACAGAATATAGCGGGGCTCACACGATGAT-
ATAGGGTAACCAAATTGGTGCTGCTTTCTGGCAAACCATCTCTGGCGAGCACGGTCTCGACAGCAA
TGGCGTGTAAGTATCTGGATTGCCAATTCCGACGCCAGCAAAAATAACTGACCACCAATAAATAGC
TACAACGGAACCTCGGAGCTCCAGCTCGAACGCATGAGCGTTTACTTCAACGAGGTACGCAACCAG
GGCAACAGGTAGCAAACATTCAAAAATAGTTACTAATCACCCCAACATGCACAGGCATCTGGCAA
CAAGTATGTTCCCCGCGCCGTCCTCGTCGATCTCGAGCCGGGTACTATGGACGCCGTCCGTGCTGG
CCCCTTCGGCCAACTTTTCCGTCCCGACAACTTCGTCTTCGGCCAGTCTGGTGCCGGAAACAACTGG
GCGAAGGGTCATTACACTGAGGGTGCCGAGCTGGTTGACAACGTTCTTGATGTTGTTCGTCGTGAG
GCTGAgGGCTGCGATTGCCTCCAGGGTTTCCAGATCACCCACTCTCTCGGTGGTGGTACCGGTGCCG
GTATGGGTACCTTGTTGATCTCTAAGATCCGCGAAGAGTTCCCCGACCGAATGATGGCCACCTTCTC
CGTCGTTCCCTCCCCTAAGGTTTCCGACACCGTCGTCGAGCCTTACAACGCCACTCTCTCAGTTCAC
CAGCTGGTTGAGAACTCCGATGAGACCTTCTGTATCGACAACGAAGCTCTGTACGACATCTGCATG
CGTACACTCAAGCTGTCCAACCCCTCTTACGGCGACCTGAACCACCTCGTCTC---------------------------
----

>Hypoxylon_vogesiacum

-CATTACTGAGTTCTACAAACTCCCAACCCTTTGTCAACTGTACC--TGTTGCTTCGGCGCG-
AGCGTGGCTACCCTGTAGCTACCCTGGAGCTACCTACCTGCTCGATGGCTACCCTGCAGCTACCCTG
TAGCCCCGCTCTGGAGCCCGCCGAAAGACCACCAAACTCTT-
TGATATTATTGAATCTCTGAATCCTTCAACTAAATTAGTTAAAACTTTCAACAACGGATCTCTTGGT
TCTGGCATCGATGAAGAACGCAGCGAAATGCGATAAGTAATGTGAATTGCAGAATTCAGTGAATC
ATCGAATCTTTGAACGCACATTGCGCCCATTAGTATTCTAGTGGGCATGCCTATTCGAGCGTCATTT
CGACCCTGAAGCCCTGGCTGCTTCGCGTTGGGACTCTACATCTCATAGAGTAGTTCCTTAAAGTAAT
TGGCAGAGTTGGGGTATGCCCTAAGCGTAGTATTATTTCTCGCTTGAGGGTTGTCCCTGGCTACCAG
CCGTAAAGCTGTT----TTTATAGTGGTTGACCTCGGATTAGGTAGGAATACCCGCTGAACT--------
TGAAGCGGCAACAGCTCAAA-TTTGAAATCTGGCC----CTC-----
GTGGTCCGAGTTGTACTTTGCAGAGGATGCTTTTGGTGCGGT-
GCCTTCCGAGTTCCCTGGAACGGGACGCCAGAGAGGGTGAGAGCCCCGTACGGTCGGA-
CACCTACCCTATATATAGCTCCTTCGACGAGTCGAGTAGTTTGGGAATGCTGCTCTAAATGGGAGG
TAAATTTCTTCTAAAGCTAAATACCGGCCAGAGACC-
GATAGCGCACAAGTAGAGTGATCGAAAGATGAAAAGCACTTTGAAAAGAGGGTTAAATAGCACGT
GAAATTGTTGAAAGGGAAGCG-TTTGCGACCAGACCTTTTCCTGGCGGATCATCCGGTGTTC--
TCACCGGTGCACTTCGCCTGG--CTTAGGCCAGCATCGGTTTC---
TTTAGGGGGATAAAGGCTTGGGGAACGTAGCTC----TTTCGGGAGTGTT-
ATAACCCCTTGTGTAATACCTTTCG-AGGGACCGAGGACCGCGCT-TCG---
GCAAGGATGCTGGCATAATGGTCGTCAACGACCCGTCTTGAAACACGGACCAAGGAGTCGAACAT
TTGTGCGAGTGTTTGGGTG--TTAAACCCTCACGCGTAATGAAAGTGAACGGAGGTGAGAGCCCTT--
--AC----GGGTGCATCATCGACCGATCCTGATG-

TCTTCGGATGGATTTGAGTAAGAGCATAACTGTTCGGACCCGAAAGATGGTGAACTATGCGTGGAT
AGGGTGAAGCCAGAGGAAACTCTGGTGGAGGCTCGCAGCGGTTCTGACGTGCAAATCGATCGTCA
AATCTGCGCATGGGGGCGAAAGACTTATCGA-ACCAT---

ACGCTGCATCGAAACCAACAAGCATTTCGAAATCGCTCTGGCCGCCAAACCCGCGATTGTCACAAA
CGGTCTCAAGTATTCCCTCGCCACCGGAAACTGGGGCGACCAGAAGAAAGCCATGAGCTCGACGG
CCGGCGTGTCGCAGGTCCTGAACCGATACACGT------TCTCGTCGA---
CTCTTTCTCACTTGAGAAGAACGAACACGCCTATCGGCAGAGACGGCAAGCTTGCGAAGCCCCGAC
AACTGCACAACACGCACTGGGGTCTGGTCTGCCCGGCCGAGACGCCCGAGGGACAGGCCTGCGGT
CTGGTAAAGAATCTGTCGTTGATGTGCTCCATCAGCGTGGGCACGTCGACCAATCCTATCATCGATT
ACATGATTACTAGAAATATGGAGGTTCTCGAGGAGTATGAGCCGACGCGCCACCCCGACGCTACCA
AGATCTTCCTCAACGGCTCTTGGATTGGTGTGCATCACAAACCCCAGAACCTAGTGCGAGATGTCC
AGGCTTTGCGCCGCACCGGCCAGATCCCCGCCGAGGTGTCGTTGGTTCGCGACATCCGTGATCGCG
AATTCAAGATCTTTTCCGACGCCGGTCGAGTAATGCGCCCCTTGTTCGTCGTCGAGCAAGAGGTT----
----

GACGACAGCGGCATCAAGGACAAGGGAGTATTGGCTCTCACAAAGGACATGATCCAGAGATTGGA
AGCCGATGTCGACATAGATCCCGAGAGCGAAGAGTACTTCGGCTGGCAAGGCCTGATCGACGCAG
GTGTCGTCGATTATCTCGACGCGGAGGAGGAGGAGACGGCCATGATTTGCATGTCGCCGGAAGATC
TCGACATCTACCGCCGGACCAAGGCCGGCGAGGAGGTGTACCAGGACAATGGG------
GACGAGATTAACAAGCGCCTGAAGACCAAGATCAACCCCACGACGCATAGATATACCCACTGCGA
GATTCACCCTAGCATGCTCTTGGGCATCTGCGCGAGCATCATTCCGTTCCCGGACCACAATCAGGT
A----------------CCAACCCCCTTGATTCCTGCCCCTCACGCACAAACACCACAAACAT-AGAT-
TCTAAATTCTCTGCATCGCGA----------CCACAACATA---
TATTGGGAAATTGAAGCTAATCATGTCTCTTCATCTGAATAGGTTCACCTTCAGACCGGCCAATGCG
TAAGAACCACA-------------ACCGACGA-
TCAGCACGATGGAGGATGGCTTAACTCACACGAATCTTATAGGGCAACCAAATTGGTGCTGCTTTC
TGGCAAACCATCTCTGGCGAGCACGGTCTCGACAGCAATGGCGTGTACGTGTCTTATTGGTCAATT
CGCAGAGAGGCATCTCGAACTGACACCCTATCAACAGGTACAACGGAACCTCTGAGCTCCAGCTCG
AGCGCATGAGCGTCTACTTCAACGAGGTACGATATCTTGAAAAATCAACA-
AGATGCGCAAGACCCGTTACTAATCA-
CCTAACATGCACAGGCATCTGGTAACAAGTACGTGCCCCGTGCCGTCCTCGTCGATCTCGAGCCCG
GTACCATGGACGCCGTCCGTGCTGGTCCCTTCGGCCAGCTCTTCCGACCCGACAACTTCGTCTTCGG
CCAGTCCGGTGCCGGAAACAACTGGGCCAAGGGTCACTACACCGAGGGTGCTGAGCTGGTCGACC
AGGTTCTCGACGTCGTCCGTCGCGAGGCCGAGGGCTGCGACTGCCTCCAGGGTTTCCAGATCACCC
ACTCTCTCGGCGGTGGCACTGGTGCCGGTATGGGCACCCTGCTGATCTCCAAGATCCGCGAGGAGT
TCCCCGACCGCATGATGGCCACCTTCTCCGTCGTCCCCTCCCCCAAGGTCTCCGACACCGTCGTCGA
GCCTTACAACGCCACCCTCTCCGTCCACCAGCTGGTCGAGAACTCGGACGAGACCTTCTGCATCGA
CAACGAGGCTCTCTACGACATCTGCATGCGCACTCTCAAGCTGTCCAACCCCTCGTACGGCGACCT
GAACCACCTGGTCTCCGCCGTCATGTCCGGCGTCACCACCTGCCTGCGATTCCCCGGTCAGCTGAA
CTCTGACCTGCGCAAGCTCGCCGTCAACATGGTGCCCTTCCCGCGTCTGCACTTCTTCATGGTCGGC
TTCGCGCCCCTGACCAGCCGCGGTGCCTACACCTTCCGCGCCGTCACCGTTCCCGAGTTGACTCAGC
AGATGTTCGACCCCAAGAACATGATGGCCGCCTCTGACTTCCGCAACGGTCGTTACCTGACGTGCT
CGGCCATCTTGTAAGACACCCACCCTACTCCGTATGATGCCGCTCATCTGCTAACCTGACTCTTCCA
GCCG--------------

>Hypoxylon_chrysalidosporum_FCATAS2710

-CATTACTGAGTTCTCCAAACCCCAAACCCTATGTGAACATTACCATCGTTGCCTCGGCG---GGCGC--
CCCTTC--AAAA-------------------
ACGGCTCCGCCGGTGGATCCCTAAACTCTTA-
GTATACTGTGAATATCTGAATGCTTCAACTTAAATAGTTAAAACTTTCAACAACGGATCTCTTGGTT
CTGGCATCGATGAAGAACGCAGCGAAATGCGATAAGTAATGTGAATTGCAGAATTCAGTGAATCA
TCGAATCTTTGAACGCATATTGCGCCCATTAGTATTCTAGTGGGCATGCCTATTCGAGCGTCATTTC
AACCCTTACGCCCTCGTCGCGTAGCGTTGGGACTCTACCGCAGCTGCGGTAGTTCCTGAAATGTAG
CGGGCGGACCTGAGCGTACCCCAAGCGTAGTAACTTCTCTCGCTTCTGCGGTACCTGGACTTTCTAG
CCCTAAAACCCCCTAATTAATCAAGTGGTGACCTCGAA-TAGGTAGAATCCCCAGTT-------------

TGAAGCGGCAACAGCTCAAA-TTTGAAATCTGGCC----CTC-----
GCGGTCCGAGTTGTAATTTGTAGAGGATGCTTTGGGCGCGGT-
ACCTACCGAGTTCCCTGGAACGGGACGCCATAGAGGGTGAGAGCCCCGTACGGCTGGA-
TACCTAGCCTCTGTATAGCTCCTTCGACGAGTCGAGTAGTTTGGGAATGCTGCTCTAAACGGGAGG
TAAATTTCTTCTAAAGCTAAATACCGGCCAGAGACC-
GATAGCGCACAAGTAGAGTGATCGAAAGATGAAAAGCACTTTGAAAAGAGGGTTAAATAGCACGT
GAAATTGTTGAAAGGGAAGCG-TTTGCGACCAGACCTGTACCTGGCGGATCATCCGGTGCTC--
GCACCGGTGCACTTCGCCTGG--CATAGGCCAGCATCGGCTTC---
GCCAGGGGGATAAAGGCTTAGGGAAAGTAGCTCCC--TCGTTGGAGTGTT-
ATAGCCCTTCGCGTAATGCCCTTGG-CGGGACCGAGGACCGCGCT-TCGT--
GCAAGGATGCTGGCATAATGGTCGTCAACGACCCGTCTTGAAACACGGACCAAGGAGTCGAACAT
TTGTGCGAGTGTTAGGGCG--TCAAACCCTTACGCGTAATGAAAGTGAACGGAGGTGAGAGCCTT----
-A-----GGGCGCATCATCGACCGATCCTGATG-
TCTTCGGATGGATTTGAGTAAGAGCATAACTGTTCGGACCCGAAAGATGGTGAACTATGCGTGGAT
AGGGTGAAGCCAGAGGAAACTCTGGTGGAGGCTCGCAGCGGTTCTGACGTGCAAATCGATCGTCA
AATCTGCGCATGGGGGCGAAA-GACTATCGA-ACCAT--

GCGCTGCATCGACTTGAATAAGCATTTCGAAATCGCACTTGCTGTCAAGCCGGCAATTATCACAAA
CGGACTGAAGTATTCGCTCGCGACCGGAAATTGGGGTGATCAGAAGAAGGCGATGAGCTCGACTG
CTGGTGTCTCTCAAGTCTTGAACCGATACACGT------TCGCTTCGA---
CTCTCTCTCACTTGAGAAGAACCAACACGCCTATTGGAAGAGACGGCAAGCTTGCGAAGCCTCGAC
AGCTTCACAACACCCATTGGGGTCTAGTCTGTCCGGCTGAGACGCCGGAAGGACAGGCTTGTGGCC
TAGTCAAGAATTTATCACTGATGTGCTCTGTCAGTGTGGGCACGTCGACCGAGCCTATCATTGATTA
TATGATAACGAGAAATATGGAAGTTTTGGAAGAGTACGAACCTTTGCGTTACCCGGACGCTACTAA
GATCTTTCTGAATGGTTCTTGGATCGGTATCCACCAAGATCCCAAGGCTCTCGTCGAGGATGTCCAA
AACCTACGTCGCACGAACCAGATTCCCGCCGAAGTATCCTTGTTCCGCGATATTCGAGATCGTGAA
TTCAAGATTTTCTCCGATGCCGGTCGAGTCATGCGTCCCTTGTTCGTTGTGCAACAAGGAGAG--------
----
AGCCCCGAGGATGGCATCAAAAAAGGAAAACTGGCCCTCACTAAAGAGATGATCCAGAGGCTGGA
GGCAGACAACGACTTAGACCCCGATAGCGAAGAATACTTTGGATGGCAGGGCCTCGTCAATGAAG
GAGCTATTGATTATTTGGATGCGGAAGAAGAGGAGACAGCCATGATCTGCATGACTCCAGAAGAC
CTGGATATCTATCGTCAAGCCAAAGCCGGGCAAGACGTGTATCAGGATAACGGT------
GAAGAGGTCAACAAACGACTAAAGACCAAGATCAACCCTACAACCCACATGTACACCCACTGCGA
AATCCACCCCAGCATGCTGCTAGGTATTTGCGCGAGTATCATTCCATTCCCCGATC-----------------------
----
AAAGCTAACAGCATCTCTTCGTCTCCATAGGTTCACCTCCAGACCGGCCAATGCGTAAGAAATATC
C-----
CCCGACGATCAATGCGATCCCAGAAGGGGGGGCTTTGAGGCTCACGAAGATGGTATAGGGTAACC
AAATCGGTGCTGCTTTCTGGCAAACCATCTCTGGCGAGCACGGTCTCGACAGCAATGGCGTGTATG
TTTTTCATTCTCTTATTCCCATACCCAGGTTACTAACTGAC--
CGATGAAACAGCTACAACGGAACGTCTGAGCTCCAGCTGGAGCGCATGAGCGTTTACTTCAACGA
GGTACGCTATCTTAGAAAAAGAATATCCATGCGAAAGTCCCGTTACTAATCACCCCAACATGCACA
GGCGTCTGGTAACAAGTACGTGCCCCGAGCCGTCCTCGTCGATCTCGAGCCCGGTACCATGGACGC
CGTCCGCGCTGGTCCCTTCGGTCAGCTCTTCCGCCCCGACAACTTCGTCTTCGGCCAGTCTGGTGCC
GGAAACAACTGGGCCAAGGGTCACTACACCGAAGGTGCTGAGCTGGTCGACCACGTCCTCGACGT
CGTCCGTCGTGAGGCTGAGGGCTGTGACTGCCTTCAGGGCTTCCAGATCACCCACTCCCTCGGTGG
TGGTACCGGTGCCGGTATGGGTACTTTGTTGATCTCCAAGATCCGCGAGGAGTTCCCCGACCGCAT
GATGGCTACTTTCTCCGTCGTCCCCTCCCCCAAGGTCTCCGACACCGTTGTCGAGCCTTACAACGCT
ACCCTCTCCGTCCACCAGCTGGTCGAGAACTCGGACGAGACCTTCTGCATTGACAACGAGGCTCTG
TACGACATCTGCATGCGTACCCTTAAGCTGTCTAACCCCTCCTACGGTGACCTGAACCACCTCGTCT
CCGCCGTCATGTCTGGCGTCACCACCTGCTTGCGATTCCCCGGTCAGCTGAACTCTGACCTGCGCAA
GCTCGCCGTCAACATGGTTCCCTTCCCCCGTCTGCACTTCTTCATGGTCGGCTTCGCTCCCCTGACC
AGCCGCGGCGCCCACTCTTTCCGCGCCGTCACCGTTCCCGAGTTGACCCAGCAGATGTTCGACCCC
AAGAACATGATGGCTGCTTCTGACTTCCGCAACGGTCGTTACCTAACGTGCTCTGCCATCTTGTAAG

TTATTTATATACTTTGCCTTTGCTACACAAGTTTGCTAACTCGATTTTTCTAGCCGTGGCAAGGTCTC
CA

>Hypoxylon_lienhwacheense

-
CATTATCGAGTTGTAAAAACTCCCACCCTTTTGTGAACCTTACCGTTGTTGCCTCGGCGCGTAAGGC
--ATATCCAGCAGCTATCC--
TAGCTGCCCCTCCTCTTGGTGGGTACTCCGGGGCTACCTGGCACGCGTGCGCCGCCCCGCGTCAAT
GGAGCGCCAAACTCTTGTTTTTACAGGGAGCCTCTGAAATATATATATAATCATATAAAAACTTTC
AACAACGGATCTCTTGGTTCTGGCATCGATGAAGAACGCAGCGAAATGCGATAAGTAATGTGAATT
GCAGAATTCAGTGAATCATCGAATCTTTGAACGCATATTGCGCCCATTAGTATTCTAGTGGGCATG
CCTATCCGAGCGTCATTTCGACCCTTAAGCCCCTGTTGCTTAGCGTTGGGAGCCTGCGGTCCGGGGC
GCATCTCCTCAAAATGAGTGGCGGAGTCGCGGCGTGCTCTGAGCGTAGTAGATCTTCTCGCTTTGG
TAGCGCCCGCGGCAGCTAGCCGTTAAACCCCCTA--
CTCTTAGTGGTTGACCTCGGATTAGGTAGGAATACCCGCTGAACTTAA-----
TGAAGCGGCAACAGCTCAAA-TTTGAAATCTGGCC----CTC-----
GCGGTCCGAGTTGTAATTTGTAGAGGATGCTTTCGGCGCGGC-
GCCTTCCGAGTTCCCTGGAACGGGACGCCAGAGAGGGTGAGAGCCCCGTACGGTTGGA-
CGCCTAGCCTACACATAGCTCCTTCGACGAGTCGAGTAGTTTGGGAATGCTGCTCTAAATGGGAGG
TAAATTTCTTCTAAAGCTAAATACCGGCCAGAGACC-
GATAGCGCACAAGTAGAGTGATCGAAAGATGAAAAGCACTTTGAAAAGAGGGTTAAATAGCACGT
GAAATTGTTGAAAGGGAAGCG-TTTGCGACCAGACCTATGCCCGGCGGATCATCCGGCGTTT--
TCGCCGGTGCACTTCGCCGGGTCTCTAGGCCAGCATCGGTTCC---
CTTAGGGGGAGAAAGGCTCAGGGAACGTAGCTCCCCGTTTGGGGAGTGTT-
ATAGCCCTCTGCGTAATACCCCTCG-GGGGACCGAGGAACGCGCTGTAATG-
GCAAGGATGCTGGCGTAATGGTCGTTAACGACCCGTCTTGAAACACGGACCAAGGAGTCGAACAT
TTGTGCGAGTGTTTGGGTG--
TCAAACCCTCACGCGTAATGAAAGTGAACGGAGGTGAGAGCCCCTC---GCG---
GGGCGCATCATCGACCGATCCTGATG-
TCTTCGGATGGATTTGAGTAAGAGCATAACTGTTCGGACCCGAAAGATGGTGAACTATGCGTGGAT
AGGGTGAAGCCAGAGGAAACTCTGGTGGAGGCTCGCAGCGGTTCTGACGTGCAAATCGATCGTCA
AATCTGCGCATGGGGGCGAAAGACTTATCGA-
ACCATCTAGTAGCTGGTTACCGCCGAAGTTTCCCTCAGGATAGCAGTGT--
TGTTCTTCAGTTTTATGAGGTAAAGCGAATGATTAGGGACTCGGGGGCGCTATTTGCCTTCATCCA
TTCTCAAACTTTAAATATGTAAGAAGCCCTTGTTGCTTAGTTGAACGTGGGCATTCGAATGTACCAA
CACTAGTGGGCCATTTTTGGTAAGCAGAACTGGCGATGCGGGATGAACCGAACGCGGGGTTAAGG
TGCCAGAGTGGACGCTCATCAGACACCACAAAAGGTGTTAGTACATCTTGACAGCAGGACGGTGG
CCATGGAAGTCGGAATCCGCTAAGGACTGTGTAACAACTCACCTGCCGAATGTACTAGCCCTGAAA
ATGGATGGCGCTCAAGCGTCT-
CACCCATACCTCGCCCTTAGGGTAGAACAGAGGCCCTAAGGGCGGTGTATTGAATCGAACAAGCG
CTTCCAGATCGAGCTTGCTGCGAAGCCGGCTATCATTACCAATGGGTTGAAGTACTCTCTCGCTACA
GGAAACTGGGGCGACCAGAAGAAGGCCATGAGTTCAACTGCGGGTGTGTCACAGGTACTGAATCG
ATACACGT------TCGCGTCGA---
CCCTTTCTCACTTGAGACGGACGAATACGCCTATTGGAAGAGATGGGAAGCTGGCGAAGCCCCGCC
AATTGCACAATACACATTGGGGTCTCGTCTGTCCAGCCGAGACGCCCGAAGGTCAGGCTTGCGGTC
TGGTGAAGAATTTGGCACTGATGTGCTCCGTAAGCGTGGGCACATCAACAGATCCCATCGTAGACT
ATATGATTACCAGGAATATGGAGGTCCTAGAAGAATACGAGCCGATGAGATACCCTAATGCGACT
AAAATATTCCTCAACGGTTCCTGGATCGGAGTCCATCAGGATCCCCAGTCCCTGGTCAGGGATATC
CAAAATCTGCGGCGGTCTGGCCAGATTCCGGCTGAGGTGTCATTGGTTCGTGATATACGTGACCGC
GAATTCAAGATTTTCTCAGATGCCGGTCGCGTCATGCGTCCCCTGTTCGTTGTACAACAAGAGGAC-
----
GGGCCAAACTCGACTAAGGGTACCCTAGCGCTCAATAAAGAAATGATACAAAGGTTAGAAGCAAG
CGCAGATCTTGACCCAAATCATGAGGACTACTTCGGCTGGCAAGGTCTGGTCAACGAAGGTGTCAT
CGAATATCTCGATGCGGAGGAAGAGGAGACGGCTATGATATGCATGACGCCCGAGGATCTGGAAA
CGTTCAGGATGGCCAAAGCGGGACATAATATGTCTCAGGACATCGGG------
GATGAAATCAATAAGCGGTTAAAAACCAAGGTGAACCCTACGACGCACATGTATACACATTGCGA
GATCCACCCAAGCATGCTCTTAGGCGTCTGCGCGAGCATTATACCCTTCCCAGACCATAATCAAGT

ACCCTTGAACGCGTCGTCCAACCCTATTCATTACTACCCCTCATATACACGTAAAATCATCAT-
TCAT-TCGCGATTTTATTTACCTCGTCATCATTAAATCGCT-----
ATACACTTGGAATCTCAAGCTAATCGCGTGTTTTATCCATATAGGTTCACCTTCAGACCGGCCAAT
GCGTAAGTTAT---------
CTCCACCACGAAGCAAACATGGTTATGATCTATGGCGGAGCTCACATCAACATGATAGGGTAACCA
AATTGGTGCCGCCTTCTGGCAAACCATCTCCAGCGAGCATGGTCTCGACAGCAATGGAGTGTATGT
ATCTAATTCGACAATCACGTCGCCAAGGATAAGAACTGACCACTGATAAACAGGTACAATGGAAC
TTCCGAGCTCCAGCTTGAGCGCATGAGCGTCTACTTCAACGAGGTACGAATCCGAAGAAATTATGC
ATGTACGCGTAGGATCGGTTACTAACCTCTTCCACCTTCGCAGGCCTCTGGCAACAAGTATGTCCCT
CGCGCCGTTCTCGTCGATCTCGAGCCCGGTACTATGGACGCTGTCCGTGCTGGTCCCTTCGGTCAGC
TCTTCCGACCCGACAACTTCGTCTTTGGTCAGTCCGGTGCCGGAAACAACTGGGCCAAGGGTCACT
ACACCGAGGGTGCTGAGCTCGTCGATCAGGTCCTTGACGTCGTCCGCCGCGAGGCTGAAGGCTGTG
ACTGCCTCCAGGGCTTCCAGATCACCCACTCTCTCGGTGGTGGTACCGGTGCCGGTATGGGTACCCT
GCTTATCTCCAAGATTCGCGAGGAGTTCCCCGACCGCATGATGGCTACTTTCTCCGTCATGCCCTCC
CCCAAGGTCTCTGACACTGTCGTCGAGCCCTACAACGCTACCCTCTCCGTTCACCAGCTGGTCGAG
AACTCGGACGAGACCTTCTGCATTGACAACGAGGCTCTCTACGACATCTGCATGCGTACCCTGAAG
TTGTCCAACCCCTCGTATGGCGACCTGAACCACTTGGTCTCCGCTGTCATGTCCGGTGTCACCACCT
GTCTGCGTTTCCCTGGTCAGCTGAACTCCGACCTCCGCAAGCTCGCCGTGAACATGGTTCCCTTCCC
TCGTCTCCACTTCTTCATGGTCGGCTTTGCTCCCTTGACTAGCCGTGGTGCTCACTCTTTCCGCGCCG
TGACCGTGCCCGAGTTGACCCAGCAGATGTTCGACCCGAAGAACATGATGGCTGCCTCCGACTTCC
GCAACGGTCGATACCTGACGTGTTCTGCGATCTTGTATGATACCTTGCCCCCCCCCTATCTCGCTAC
TGATCTGCTAACATGATGTCTCTAGCCGCGGCAAGGTCTCCA

>Thamnomyces_dendroidea

-CATTAGCGAGTGGTAATAACTCGTAGCCTCGTGCGAA-CCTACC--CGTAGCCTCGGCGGGTCGT----
CCGCCC--GTGTTTAGCCG---
GGTGCGCGCTATGGCCCGTCGGTGGACGTTTTAACCTTGCT---------CGTATTCTGAATGGACTAG--
TAAACTATTACAACTTTCAACGACGGATCTCTTGGTTCTGGCATCGATGAAGAACGCAGCGAAATG
CGATACGTAATGCGAATTGCAGAATTCAGTGAGTCATCGAATCTTTGAACGCACATTGCGCCCGCT
AGCATTCTAGCGGGCATGCCTGCTCGAGCGTCATTACA-CCCCTAAGCCT-----
AGCTTGCGTTGGGAATCTAGTCAGCGCCGGCTAGTTCCTCAAAATCAGTGGCGGAGTCAGGATAGA
CCGTACGCGTAGTAAT--ATCTCGCCTGCGTAGTC---CTGGCGGCTTGCCGTAAAACATCCTCCA----
CAGT--TGAAGCGGCAACAGCTCAAA-TTTGAAATCTGGCC----CTA-
----GCGGTCCGAGTTGTACTTTGCAGAGGATGCTATGGGCGAGGC-
GCCTTCCGAGTTCCCTGGAACGGGACGCCAGAGAGGGTGAGAGCCCCGTACGGTGGGA-
CGCGTCGCCTGCGTATAGCTCCTTCGACGAGTCGAGTAGTTTGGGAATGCTGCTCTAAACGGGAGG
TAAATTTCTTCTAAAGCTAAATACCGGCCAGAGACC-
GATAGCGCACAAGTAGAGTGATCGAAAGATGAAAAGCACTTTGAAAAGAGGGTTAAACAGCACGT
GAAATCGTTGAAAGGGAAGCG-TTTGCGACCAGACGTCGCCTAGGGGGATCATCCGGCAGCC-
TCTGCCGGTGCACTTCCGCTAG--GCGAGGCCAGCGTCGGCTTC---
CTCAGGGGGATAAAGGCCCGGGACACGTATCTC----CTTCGGGAGTGTT-
ATAGCCCCGGGCGTACTACCCCTGA-GGGGACCGAGGAACGCGTT-CT----
GCAAGGACGCTGGCGTAATGGTCGTCAATGACCCGTCTTGAAACACGGACCAAGGAGTCGAACGT
TTATGCGAGTGTACGGGTG--TCAAACCCTGGCGCGTAATGAAGGTGAACGGAGGTGAGAGCCCTT--
---CG---GGGTGCATCATCGACCGATCCTGATG-
TCTTCGGATGGATTTGAGTAAGAGCATATGCGTTCGGACCCGAAAGATGGTGAACTATGCGCGGAT
AGGGTGAAGCCAGAGGAAACTCTGGTGGAGGCTCGCAGCGGTTCTGACGTGCAAATCGATCGTCA
AATCTGCGCATGGGGGCGAAAGACTTATCGA-
ACCATCTAGTAGCTGGTTACCGCCGAAGTTTCCCTCAGGATAGCAGTGT--TG-
TCTTCAGTTTTATGAGGTAAAGCGAATGATTAGGGACTCGGGGGCGCTATACTGCCTTCATCCATTC
TCAAACTTTCAATATGTAAGAAGCCCCCGTTGCTTAGCTGAACGGGGGCCTTCGAATGTTGCAACA
CTAGTGGGCCATTTTTGGTAAGCAGAACTGGCGATGCGGGATGAACCGAACGTGGGGTTAAGGTG
CCAGAGTAGACGCTCATCAGATACCACAAAAGGTGTTAGTACATCTTGACAGCAGGACGGTGGCC
ATGGAAGTCGGAATCCGCTAAGGACTGTGTAACAACTCACCTGCCGAATGTACTAGCCCTGAAAAT
GGATGGCGCTCAAGCGTCT-CACCTATACCCCGCCCTTAGGGTAGAAACGAGGCCCTAAGG------------
----
-------------ACTT------TCTCGTCGA---

CTCTTTCCCATCTAAGGCGAACCAACACGCCTATCGGAAGAGACGGGAAGCTCGCGAAACCTCGAC
AGCTGCACAATACCCACTGGGGTCTTGTCTGTCCGGCCGAAACGCCCGAAGGCCAGGCCTGCGGTC
TGGTGAAGAACCTATCGCTTATGTGCTCCATCAGCGTGGGTACCTCGACGGATCCTATCGTAGACT
ACATGATTACTAGGAATATGGAAGTCTTAGAGGAATACGAGCCGATGCGATACCCTAACGCCACC
AAGATCTTCCTCAACGGATCCTGGATCGGTGTGCATCAGGATCCCAAGTCTCTCGTCAGAGATGTC
CAGCAGCTTCGTCGGGCTAACCAAATCCCCTCCGAAGTATCTCTCGTTCGTGATATCCGTGATCGCG
AGTTCAAGATCTTTTCGGACGCCGGTCGTGTCATGCGGCCCTTGTTCGTGGTGCAGCAAGAGGAT---
----

GATCCCGAGGCTGGTATCACGAAGGGCTCGCTGGCTCTTACCAAGGAAATGATCCAGAGGTTGGA
GGCGAGTGTTGATGTCGACGCGGAGAGCGAAGAGTACTTTGGCTGGCAAAGTCTTGTCAACGCGG
GTGTTATCGAGTACCTCGACGCGGAGGAGGAAGAAACGGCCATGATTTGCATGACCCCCGAAGAT
TTAGAAACCTACCGGATGTCTAAACTCGGATATGATGTGTCTCAGGACAACGGG------
GACGAGATTAACAAGCGGCTCAAGACCAAGTTGAATCTTACGACGCACATGTACACGCATTGCGA
GATCCATCCCAGCATGCTCCTGGGTATCTGCGCGAGCATCATCCCTTTCCCCGATCACAATCAGGTA
-------------------------TGAATTCTGCCCCTCACGCATAAACACGACAACCGC-ACAT----
CAACATTACACGCTGCAACCACGTCAAATTTTATTACAGCTTGAAAAAATAGTCGAGCTAACCGCG
TTTT-----TTCAATAGGTTCACCTTCAAACTGGCCAATGTGTAAGTAGAGGTAG----
AACGATGAGGGAAAGG--
ACCGCGAATAGCAATAGCGGGGCTCATACGAAGATGATAGGGTAACCAAATCGGTGCTGCTTTCTG
GCAAACCATCTCCAGCGAGCACGGCCTCGACAGCAATGGCGTGTATGTATTTGAATTGTTAATTCT
ATCGCCGAGGATATCAACTGACCAACGATGGATAGCTACAACGGTACTTCCGAGCTTCAGCTCGAG
CGCATGAGCGTCTACTTCAACGAGGTACGAATTTCCAGATACCAAGGATTACTATATAGAATCAGT
TGTTAATCGCTTCTACGCGTGCAGGCTTCCGGCAACAAGTATGTTCCTCGTGCCGTCCTCGTCGATC
TCGAGCCCGGTACTATGGACGCTGTCCGTGCTGGTCCCTTTGGCCAGCTCTTCCGACCCGACAACTT
CGTTTTTGGCCAGTCCGGTGCTGGAAACAACTGGGCCAAGGGTCACTATACCGAGGGTGCTGAATT
GGTTGACAACGTTCTCGACGTCGTTCGTCGCGAGGCTGAGGGCTGTGACTGCCTCCAGGGTTTCCA
GATCACCCACTCCCTCGGTGGTGGTACTGGTGCCGGTATGGGTACCCTGCTGATCTCCAAGATCCGT
GAGGAGTTCCCCGACCGTATGATGGCTACCTTCTCCGTCATGCCCTCCCCCAAGGTGTCCGACACC
GTCGTTGAGCCTTACAACGCCACCCTCTCCGTCCACCAGCTGGTCGAGAACTCGGACGAGACCTTC
TGTATCGACAACGAGGCTCTGTACGACATCTGCATGCGTACCCTGAAGCTGTCTAACCCCTCGTAC
GGTGACCTGAACCACCTGGTCTCCGCCGTCATGTCCGGTGTTACCACTTGCTTGCGTTTCCCCGGTC
AGCTAAACTCGGACCTGCGCAAGCTTGCCGTGAACATGGTTCCTTTCCCTCGTCTCCACTTCTTCAT
GGTCGGCTTCGCTCCCCTGACCAGCCGTGGCGCTCACTCTTTCCGTGCCGTCACCGTTCCCGAGTTG
ACTCAGCAGATGTTCGACCCCAAGAACATGATGGCTGCTTCTGACTTCCGCAACGGTCGTTACCTG
ACGTGCTCTGCCATCTTGTACGATATTCCCCCTTTCCCCTCATGCGCTATTGTTTTGCTAACTTGAAC
CGCGTAGCCGTGGCAAGGTCTCCA

>Durotheca_comedens_YMJ90071615

-
CATTAGCGAGTCGTAAAAACCTTCCACCCACCGCGAACGTTAAACCCGTTGCCTCGGCGCAGTGCG
CGGCTGCCTGGCGACCGCCCCC------------GGGGGGGCGGGGGGCCCGGAGCCGCCCTGC-
GGTCCGCGCGACGGCCCGCCGCAGGACCATCAAACTCTGTCTGCC-GAGAG-
GCTTCTGAGCTGTATAGCAGTAAATGTTAAAACTTTCAACAACGGATCTCTTGGCTCTGGCATCGAT
GAAGAACGCAGCGAAATGCGATAAGTAGTGTGAATTGCAGAATTCAGTGAATCATCGAATCTTTG
AACGCACATTGCGCCCGCCAGCATTCTGGCGGGCATGCCTGTTCGAGCGTCATTTCGACCCTTAAG
CCCCCGCGGCTTAGCGTTGGGAGCATACGAGCCGAGGCGTAGCTCCTCAAATACAGTGGCGGCGTC
GGAGTCCGTCCCGAGCGTAGTAGTTCTCCTCGCTCCGGCGGCGGACGGAGAAGCCGGCCGTAAAA
ACGCGTCGA---CTAATGGTTGACCTCGGATCAGGTAGGGCTACCCGCTGAACTTAAGCATA-----------
----

GCGGTGCATCGATTCGAACCGGCGTTTCCACATCGAGCTCGCCGCCAAGCCCTCTATCATCACGAA
CGGCCTGAAATACTCTCTGGCTACCGGCAACTGGGGTGACCAGAAGAAGGCGATGAGCTCGACCG
CCGGCGTGTCGCAAGTGCTCAACCGTTATACCT------TCGCCTCGA---
CCCTGTCGCATTTGCGGAGAACCAATACGCCTATAGGGCGCGATGGGAAGCTCGCGAAACCTCGGC
AGCTTCACAACACCCACTGGGGTTTGGTTTGCCCAGCCGAAACACCAGAGGGCCAGGCTTGCGGGC
TGGTCAAAAACTTGTCGTTGATGTGCTCCATCAGCGTCGGCACATCGACGGACCCCATTGTCGACT
ACATGATCACGCGAAATATGGAGGTCCTGGAGGAGTACGAGCCTATGCGATACCCCAACGCCACC
AAGATCTTCCTCAACGGCTCTTGGATCGGCGTACACCAGGATCCCAAGGCCTTGGTTAGGGACGTC
CAGAACTTGCGCCGGGCCAATCAGATCCCGGCCGAGGTGTCTCTGATTCGCGATATTCGCGATCGC
GAATTCAAGATCTTTTCGGACGCCGGTCGGGTCATGCGTCCCCTGTTCGTCGTCGAGCAGGAGGGC
GAG---
AGGAAGGGGTCTTTGATTCTTACCAAGGACATGATCCACAGACTGGAGGCAGACGTGGATATATCC
CCGGACGCCGAGGATTATTTCGGCTGGCAGGGTCTCGTCAACGAGGGTGTCATCGAATTTCTCGAC
GCCGAGGAGGAAGAGACAGCCATGATCTGCATGACGCCCGAGGATCTTGAAGCCTACCGTCAGGC
CAAGGCCGGTTACGCGCCGGTCGAGGATGACAGC------
GAGGAGATTAATCGTCGTCTGAAGACCAAGATGAACCCTACCACACATATGTACACCCACTGTGAG
ATCCATCCGAGCATGTTACTAGGCATCTGCGCTAGCATCATCCCGTTCCCCGACCACAACCAGGTTT
CCCCCAGCAAGTAC---GACCCCCTCAC-
ACACGCTCTGCACGCACACATGACACGAGCACCCCATCCCGTGGCCCTCGACCTCGGGCTGTCAAC
AACCCGA--
CCCGACAGATGGAAAATGCAAAGCTAACCGTGTCTCGCTCTCTCGATAGGTTCACCTCCAGACCGG
CCAATGCGTAAGTGCCTCCCA----
ACCGGCAGAACACCCCCCATCGCGCTAGACTCTGGCGTCGCTCACACGAGATTCGCAGGGCAACCA
AATTGGTGCTGCTTTCTGGCAGACCATCTCGGGCGAGCACGGTCTCGACAGCAATGGCGTGTACGT
ATATGACGGGTCGACGCCGGGGACGGTAACGCTGACGGACGGCCGGCGGATAGCTACAATGGCAC
CTCGGAGCTCCAGCTCGAGCGTATGAGCGTCTACTTTAACGAGGTATGTCGTTGCGGCCGCGATGG
ATCCATACACA-----GAATGTTGA-
CGTGCCGCCCTTGACAGGCCTCTGGTAACAAGTACGTGCCTCGCGCCGTGCTGGTCGATCTCGAGC
CTGGCACCATGGACGCCGTCCGTGCTGGCCCCTTCGGTCAGCTCTTCCGGCCCGACAACTTCGTCTT
CGGCCAGTCGGGCGCCGGCAACAACTGGGCCAAGGGTCACTACACGGAGGGCGCTGAGCTCGTCG
ACCAGGTCCTCGACGTCGTCCGCCGCGAGGCTGAGGGCTGCGACTGCCTCCAGGGCTTCCAGATCA
CCCACTCCCTCGGCGGTGGTACCGGTGCCGGTATGGGTACCCTGCTGATTTCCAAGATCCGCGAGG
AGTTCCCGGACCGCATGATGGCCACCTTCTCCGTCGTCCCCTCCCCCAAGGTCTCCGACACCGTTGT
CGAGCCTTACAATGCTACCCTCTCCGTCCACCAGCTGGTCGAGAACTCTGACGAGACTTTCTGTATC
GATAACGAGGCCCTGTACGACATTTGCATGCGCACTTTGAAGCTGTCGAACCCCTCGTATGGCGAT
CTCAACCACCTCGTCTCCGCTGTTATGTCCGGCGTCACTACCTGCCTGCGCTTCCCCGGTCAGCTGA
ACTCTGACTTGCGCAAGCTTGCCGTGAACATGGTGCCCTTCCCTCGTCTGCACTTCTTCATGGTCGG
GTTTGCACCCCTGACCAGCCGCGGTGCTCACTCTTTCCGCGCCGTCACCGTTCCCGAGTTGACCCAG
CAGATGTTCGACCCCAAGAACATGATGGCTGCTTCGGATTTCCGCAACGGTCGATATCTGACGTGT
TCTGCCATCTTGTAAGCCTCGTGTTCACTCTCTTC--TGTGGGCCCGGGTGCTAACCTCAAC-
AACTAGCCGTGGCAAGGTCTCGA

>Durotheca_guizhouensis_GMBC0065

AACGTTAACCTTGTCGCCTCGGCGTAGTGCGCAGCTGCCTGGTAGCTCTCTTCAGGCCCTGTAGAG
AGGGATAGGCGGAAGAAAGACTGCCCTGC-
GGCCCGCGCGACGGCCCGTCGTAGGACTACTAAACTCTGGTTTTCAGAGAGCGTCTCTGAATTTTT
ATAAAGACATGTTAAAAACTTTCAACAACGGATCTCTTGGCTCTGGCATCGATGAAGAACGCAGCG
AAATGCGATAAGTAGTGTGAATTGCAGAATTCAGTGAATCATCGAATCTTTGAACGCACATTGCGC
CCACTAGCATTCTGGTGGGCATGCCTATTCGAGCGTCATTACGACCCTTAAGCCTCTACGGCTTAGC
GTTGGGTGCGTGCGAGCCGGACCGCAGCTTCTTAAATATAGTGGCGGCGTCGGAGTCTGTCCCGAG
CGTAGTAGT--

TTTGAAATCTGGCTCCCCCTCTTACCGGGGCCCGAGTTGTAATTTGCAGAGGATGCTTTGGGCGAG
GCGGCCTTCCGAGTTCCCTGGAACGGGACGCCGTAGAGGGTGAGAGCCCCGTACGGTTGGT-
CGCCGAGCTCGCGTATAGCGCCTTCGACGAGTCGAGTAGTTTGGGAATGCTGCTCTAAATGGGAGG

TAAATTTCTTCTAAAGCTAAATACCGGCCAGAGACC-
GATAGCGCACAAGTAGAGTGATCGAAAGATGAAAAGTACTTTGAAAAGAGGGTTAAACAGCACGT
GAAATTGTTGAAAGGGAAGCG-TTTGCGACCAGACCTTTTCCGGGCGGATCATCCGGCGTTC--
TCGCCGGTGCACTCCGCCCGG--TCTAGGCTAGCATCGGTTCCCG-
TCCGGGGGGAGAAAGGCCGGGGGAAAGTGGCTC----CTCCGGGAGTGTT-
ACAGCCCTCGGCGGAATGCCCTCGG-GGGAACCGAGGACCGCGCT-TCG---
GCAAGGATGCTGGCGTAATGGTCGTCAACGACCCGTCTTGAAACACGGACCAAGGAGTCGAACAG
CTGTGCGAGTGTTCGGGTGACTAAAGCCCCGGCGCGTAATGAAGGTGAACGGAGGTGAGAGCCCC
CTGTAGCGGG-
GGGCGCATCATCGACCGATCCTGATGTTCTTCGGACGGATTTGAGTAAGAGCACGGCTGTTCGGAC
CCGAAAGATGGTGAACTATGCGTGGATAGGGTGAAGCCAGAGGAAACTCTGGTGGAGGCTCGCAG
CGGTTCTGACGTGCAAATCG--

ACGGTGTATCGATTCGAACCGGCGTTTCCATATCGAGCTTGCTGCTAAGCCTTCCATCATCACGAAC
GGCCTGAAATACTCTCTGGCTACCGGCAACTGGGGCGACCAGAAGAAGGCGATGAGCTCGACCGC
CGGTGTATCGCAAGTACTTAATCGCTATACCT------TCGCCTCGA---
CCCTGTCCCATTTGCGGAGAACCAATACGCCTATAGGACGCGACGGGAAGCTCGCGAAACCTCGGC
AGCTTCACAACACCCACTGGGGGTTGGTTTGCCCAGCCGAGACACCAGAGGGCCAGGCCTGCGGG
CTGGTCAAGAACTTGTCTTTGATGTGCTCCATCAGCGTCGGCACATCAACAGATCCTATCGTCGACT
ACATGATCACCCGAAATATGGAGGTTCTGGAGGAGTACGAGCCTATGCGATACCCCAACGCTACCA
AGATCTTCCTCAACGGCTCTTGGATCGGTGTACACCAAGATCCCAAGGCCTTGGTTAGAGACGTCC
AGAACTTGCGCCGGGCCAATCAAATCCCGGCCGAGGTATCCCTGATTCGCGATATTCGCGATCGCG
AGTTCAAGATCTTTTCGGACGCCGGTCGGGTCATGCGTCCTCTGTTCGTCGTCGAGCAGGAGGGCG
AG---
AGGAAGGGGTCTTTGATTCTTACCAAGGACATGATCCACAGGCTAGAGGCGGACGTCGACCTATCT
CCGGATAGCGATGACTATTTCGGCTGGCAGGGTCTGGTCAACGAGGGTGTCATCGAATTCCTAGAC
GCCGAGGAGGAAGAGACGGCCATGATTTGCATGACGCCCGAGGATCTGGAAGCCTACCGTCAAGC
CAAGGCCGGTTACGCGCCGGTCGAGGACGACAGC------
GAAGAGATTAACCGTCGTCTCAAGACCAAGATGAACCCTACCACACATATGTACACGCACTGCGA
GATTCATCCGAGCATGCTGCTAGGTATCTGCGCTAGCATTATCCCGTTCCCCGACCACAACCAGGTT

TGTGTTGACACTGGGGACGGTAACGCTGACAGACGGTCGGTGGACAGTTACAATGGCACCTCGGA
GCTCCAGCTTGAGCGCATGAGCGTCTACTTTAACGAGGTACGTGGTTGCGGCGATGAGGGATCCAT
GGACAGGACGAGACGCTAA-
CGTGCCCCGGGTGACAGGCCTCTGGTAACAAGTACGTGCCTCGCGCCGTCCTTGTCGATCTCGAGC
CCGGCACCATGGACGCCGTCCGTGCTGGCCCCTTCGGCCAGCTCTTCCGCCCCGACAACTTCGTCTT
CGGCCAGTCGGGTGCCGGCAACAACTGGGCCAAGGGTCACTACACGGAGGGCGCCGAGCTCGTCG
ACCAGGTCCTTGACGTCGTCCGCCGCGAGGCTGAGGGCTGCGACTGCCTTCAGGGCTTCCAGATCA
CCCACTCTCTCGGCGGCGGTACCGGTGCCGGTATGGGCACCCTGCTGATCTCCAAGATCCGCGAGG
AGTTCCCAGACCGCATGATGGCTACCTTCTCCGTCGTCCCCTCCCCCAAGGTCTCCGACACCGTCGT
CGAGCCTTACAACGCCACCCTCTCCGTCCACCAGCTGGTCGAGAACTCTGACGAGACTTTCTGTATC
GATAACGAGGCCCTGTATGACATTTGCATGCGCACTCTGAAGCTGTCGAACCCCTCGTATGGTGAT
CTCAACCACCTCGTCTCTGCCGTCATGTCCGGTGTCACCACTTGCCTGCGCTTCCCCGGTCAGCTAA
ACTCTGACCTGCGCAAGCTTGCCGTGAACATGGTGCCCTTCCCTCGTCTACACTTCTTCATGGTCGG
CTTCGCACCCCTGACCAGCCGCGGTGCCCACTCTTTCCGCGCTGTCACCGTTCCCGAGTTGACCCAG
CAGATGTTTGACCCCAAGAACATGATGGCTGCTTCGGATTTCCGCAACGGTCGATATCTAACGTGC
TCTGCCATCTTGTAAGCCTTGTGTTCACTCTCTTC--
AACAAACCCAACTGCTAACCCTAACAAACTAGCCGTGGCAAGGTCTCGA

>Durotheca_rogersii_YMJ92031201

-
CATTAGCGAGTTGTAAAAACCTTCTACCCGATGTGAACGTTAACGTCGTTGCCTCGGCGTAGTGCG
TAGCTGCCTGGTAGCTCTCCTCAGGGTTGTTAGGGGGGGATTAGAGTAAAGAGAGCTACCCTGT-

AGTCTGCGCGACGGCCCGCCGTAGGACCACTAAACTCTGTTTTTC-
GAGAGCGTCTCTGAATTTTTTAGAAGAACACGTTAAAACTTTCAACAACGGATCTCTTGGCTCTGG
CATCGATGAAGAACGCAGCGAAATGCGATAAGTAGTGTGAATTGCAGAATTCAGTGAATCATCGA
ATCTTTGAACGCACATTGCGCCCACTAGCATTCTGGTGGGCATGCCTATTCGAGCGTCATTACGACC
TTTAAGCCTCTGCAGCTTAGCGTTGGGTGCGTGCGAGCTGGGCCGCAGCTCCTTAAATACAGTGGC
GGCGTCGGAGTCCGTCCCGAGCGTAGTAGTTCTCCTCGCTCTGGTGGCGGTCAAGAAAGCTGGCCG
TAAAATAAAGTTTATTTCTAGTGGTTGACCTCGGATTAGGTAGGGTTACCCGCTGAACTTAAGCAT
A---

ACGGTGTATCGATTCGAACCGGCGTTTCCATATCGAGCTTGCTGCTAAGCCTTCCATCATCACGAAC
GGCCTGAAATACTCTCTGGCTACCGGCAACTGGGGCGACCAGAAGAAGGCGATGAGCTCGACCGC
CGGTGTATCGCAAGTCCTTAATCGTTATACCT------TCGCGTCGA---
CCCTGTCCCATCTGCGGAGAACCAATACGCCTATAGGACGCGACGGGAAGCTCGCGAAACCTCGG
CAGCTTCACAACACCCACTGGGGGTTGGTTTGCCCAGCCGAAACACCAGAGGGCCAGGCCTGCGG
GCTGGTCAAGAACTTGTCTTTGATGTGCTCCATCAGCGTCGGCACATCGACGGATCCTATCGTCGAC
TACATGATCACCCGAAATATGGAGGTTCTGGAGGAGTACGAGCCCATGCGATACCCCAACGCTACC
AAGATCTTCCTCAACGGCTCTTGGATCGGCGTACACCAAGATCCGAAGGCCTTGGTTAGAGACGTC
CAGAACTTGCGCCGGGCCAATCAGATCCCGGCCGAGGTGTCCCTAATTCGCGATATTCGCGATCGC
GAGTTCAAGATCTTTTCGGACGCCGGTCGGGTCATGCGTCCCCTGTTCGTCGTCGAGCAGGAGGGC
GAG---
AGGAAGGGGTCTTTGATTCTTACCAAGGACATGATCCACAGGCTAGAGGCGGACGTGGACCTATCT
CCAGATAGCGATGACTATTTCGGCTGGCAGGGTCTGGTCAACGAGGGTGTCATCGAATTCCTAGAC
GCCGAGGAGGAAGAGACGGCCATGATTTGCATGACGCCCGAGGATCTGGAAGCCTACCGTCAAGC
CAAAGCCGGTTACGCGCCGGTCGAGGACGACAGC------
GAGGAGATTAACCGGCGTCTCAAGACCAAGATGAACCCTACCACACACATGTACACACACTGCGA
GATTCATCCGAGCATGTTGCTAGGTATCTGTGCTAGCATTATCCCGTTCCCCGACCACAACCAGGTT
AAAAAAACCGAGTAC---GACCCCCTCAC-
ACACGCTCTGCACGCATACATGACACGACCACCGCGTCTTGTGGTCCTCGGCGTTGAGCTGTCAAC
AACCCAA--
CACAACAGATGAAAATTGCAAAGCTAACCGTGTCTCGTTCTCTTGATAGGTTCACCTCCAGACCGG
CCAATGCGTAAGTGCC----------
ACAACCAACGAACCCCCATCGCGCTAGAATCTGGCAGCGCTCACACAGGATTCGCAGGGCAACCA
AATTGGTGCTGCTTTCTGGCAGACCATCTCGGGCGAGCACGGTCTCGACAGCAATGGCGTGTACGT
ATATGATGTGTCGACACCAGGGACGGTAATGCTGACAGACGGTCGGTGGACAGTTACAATGGCAC
CTCGGAGCTCCAGCTTGAGCGCATGAGCGTCTACTTCAACGAGGTACGTGGTTGCGGCCATGAGGG
ATCCATGGACGGGACGAGACGCTAA-
CGTGCCCCGGGTGACAGGCCTCTGGTAACAAGTACGTGCCTCGCGCCGTCCTTGTCGATCTCGAGC
CCGGCACCATGGATGCCGTCCGTGCTGGCCCCTTCGGCCAGCTCTTCCGCCCCGACAACTTCGTCTT
CGGCCAGTCGGGTGCTGGCAACAACTGGGCCAAGGGCCACTACACCGAGGGCGCCGAGCTCGTCG
ACCAGGTCCTCGACGTCGTCCGCCGCGAAGCTGAGGGCTGCGACTGCCTTCAGGGCTTCCAGATCA
CCCACTCTCTCGGCGGTGGTACCGGTGCCGGTATGGGCACCCTGCTGATCTCCAAGATCCGCGAGG
AATTCCCAGACCGCATGATGGCCACCTTCTCCGTCGTCCCCTCCCCCAAGGTCTCCGACACCGTCGT
CGAGCCTTACAACGCCACCCTCTCCGTCCACCAGCTGGTCGAGAACTCTGACGAGACTTTCTGTATC
GATAACGAGGCCCTGTACGACATTTGCATGCGCACTCTGAAGCTGTCGAACCCCTCGTACGGTGAT
CTCAACCACCTCGTCTCTGCCGTCATGTCCGGTGTCACCACTTGCCTGCGCTTCCCCGGTCAGCTAA
ACTCTGACCTGCGCAAGCTTGCCGTGAACATGGTGCCCTTCCCTCGTCTACACTTCTTCATGGTCGG
CTTCGCACCCCTGACCAGTCGCGGTGCCCACTCTTTCCGCGCTGTCACCGTTCCCGAGTTGACCCAG
CAGATGTTTGACCCCAAGAACATGATGGCTGCTTCGGATTTCCGCAACGGTCGATATCTCACGTGC

TCTGCCATCTTGTAAGCCTCGTGTTCACTCTCTTCCAAACAAACCCAACTGCTAACCCTAACAAATT
AGCCGTGGCAAGGTCTCGA

>Durotheca_crateriformis_GMBC0205

TTCTACCCATTGCGAACGTTAAACTTATTGCCTCGGCGTAGTGCGCGGCTGCCTGGTACTTTTTTTC
AAGTTCTCTAAGAGAAAAGAAGCTAAAAGATAGCTACCCTGC-
AGTCCGTGCGACGGCCCGCCGTAGGACCATCAAACTCTGTTTTTC-
GAGAGAATTTCTGAACTGTTATACGAAATATATTAAAACTTTCAACAACGGATCTCTTGGCTCTGG
CATCGATGAAAAACGCAGCGAAATGCGATAAGTAGTGTGAATTGCAGAATTCAGTGAATCATCGA
ATCTTTGAACGCACATTGCGCCCACTAGTATTCTGGGGGGCATGCCTATTCGAGCGTCATTACGACC
CTTAAGCCTCTATAGCTTATAGCTGGGAGAGTGCGGGCCGGACCGCACCTTCTTAAAAACACTGGG
GGCGTCTCAG--
------------------CAACAGCTCAAA-TTTGAAATCTGGCC----
CTCTTGTCGGGGTCCGAGTTGTAATTTGCAGAGGATGCTTTAGGCGCGGCGGCCTTCCGAGTTCCCT
GGAACGGGACGCCGTAGAGGGTGAGAGCCCCGTACGGTTGGA-
CGCTAAGCTTATATATAGCGCCTTCGACGAGTCGAGTAGTTTGGGAATGCTGCTCTAAATGGGAGG
TAAATTTCTTCTAAAGCTAAATACCGGCCAGAGACC-
GATAGCGCACAAGTAGAGTGATCGAAAGATGAAAAGTACTTTGAAAAGAGGGTTAAACAGCACGT
GAAATTGTTGAAAGGGAAGCG-TTTGCGACCAGACCTTTTCCGGGCGGATCATCCGGCGTTC--
TCGCCGGTGCACTCCGCCCGG--
TTTAGGCCAGCATCGGTTCCTGTTTCGGGGGGAGAAAGGCCGGGGGAAAGTAGCTC----
CTTCGGGAGTGTT-ACAGCCCTCGGCGGAATGCCCTCGG-GGGGACCGAGGACCGCGCC-TTCGG-
GCAAGGATGCTGGCGTAATGGTCGTCAACGACCCGTCTTGAAACACGGACCAAGGAGTCGAACAG
CTGTGCGAGTGTTCGGGTGGCTAAAGCCCTTGCGCGTAATGAAGGTGAACGGAGGTGAGAGCCTCC
TGTAACGGG-
GGGTGCATCATCGACCGATCCTGATGTTCTTCGGATGGATTTGAGTAAGAGCATAGCTGTTCGGAC
CCGAAAGATGGTGAACTATGCGTGGATAGGGTGAAGCCAGAGGAAACTCTGGTGGAGGCTCGCAG
CGGTTCTGACGTGCAAATCGATCGTCAAAT--

GCATCGATTCGAACCGGCGTTTCCATATCGAGCTTGCCGCTAAGCCTTCCATCATCACGAATGGCCT
GAAATACTCTCTGGCTACCGGCAACTGGGGCGACCAGAAGAAGGCGATGAGCTCGACAGCCGGCG
TATCGCAAGTCCTCAACCGTTATACCT------TCGCCTCGA---
CCCTGTCCCGTTTGCGGAGAACCAATACGCCTATTGGACGCGACGGAAAGCTCGCGAAACCTCGGC
AGCTTCACAACACCCACTGGGGTTTGGTTTGCCCAGCCGAGACACCAGAGGGTCAGGCCTGCGGGC
TGGTCAAGAACTTGTCGTTGATGTGCTCTATCAGCGTCGGTACATCGACGGATCCCATCGTCGACTA
CATGATCACCCGAAATATGGAGGTTCTGGAGGAGTACGAGCCTATGCGATACCCCAACGCCACTAA
GATCTTCCTCAACGGCTCTTGGATCGGCGTACACCAGGATCCCAAGGCCCTGGTTAGAGACGTCCA
GAACTTGCGCCGGGACAATCAGATCCCGGCCGAGGTGTCCCTGATTCGCGATATTCGCGATCGCGA
GTTCAAGATCTTTTCGGACGCCGGTCGAGTCATGCGTCCCCTGTTCGTCGTCGAGCAGGAGGGCGA
G--
AGGAAGGGGTCTTTGATTCTTACCAAGGACATGATCCACAGGCTAGAGGCGGACGTGGACCTATCT
CCGGATGGCGATGACTATTTCGGCTGGCAGGGTCTGGTCAACGAGGGTGTCATCGAATTCCTAGAC
GCCGAGGAGGAAGAGACGGCCATGATTTGCATGACGTCCGAGGATCTCGAAGCCTACCGTCAAGC
CAAGGCTGGTTACGCGCCGGTCGAGGACGATAGC------
GAGGAGATCAACCGTCGCCTTAAGACAAAGATGAACCCTACCACACATATGTACACGCACTGTGA
GATTCATCCGAGCATGTTGCTAGGTATCTGCGCTAGCATTATCCCATTCCCCGCCCA-----------------------
----

ATGTATCAACACCGGGAACGGTAACGCTGATAGATGGTTGGTGGATAGTTACAATGGCACCTCGGA
GCTCCAGCTCGAGCGCATGAGCGTCTACTTCAATGAGGTAAGTGGTTGCGGCGATGACGGATCCAT
AGACA-----AGACGCTAA-
CGTGCCGCCGATGATAGGCCTCTGGTAACAAGTACGTGCCTCGCGCCGTTCTTGTCGATCTCGAGC
CCGGCACCATGGACGCCGTCCGTGCTGGCCCCTTCGGCCAACTCTTCCGCCCCGACAACTTCGTCTT

CGGCCAGTCGGGTGCCGGCAACAACTGGGCCAAGGGCCACTACACGGAGGGCGCTGAGCTCGTCG
ACCAGGTCCTCGACGTCGTCCGCCGCGAGGCTGAGGGCTGCGACTGCCTCCAGGGCTTCCAGATCA
CCCACTCTCTCGGCGGTGGTACCGGTGCCGGTATGGGTACCCTGCTGATCTCCAAGATCCGTGAGG
AGTTCCCAGACCGCATGATGGCTACCTTCTCCGTCGTCCCCTCCCCCAAGGTCTCCGACACCGTCGT
CGAGCCTTACAATGCTACCCTCTCCGTCCACCAGCTGGTCGAGAACTCTGACGAGACTTTCTGTATC
GATAACGAGGCCCTGTACGACATTTGCATGCGCACTCTGAAGCTGTCGAACCCCTCGTATGGTGAT
CTCAACCACCTCGTCTCCGCCGTCATGTCTGGTGTCACCACTTGCCTGCGCTTCCCCGGTCAGCTAA
ACTCTGATCTGCGCAAGCTTGCCGTGAACATGGTGCCCTTCCCTCGTCTACACTTCTTCATGGTCGG
CTTCGCACCCCTGACCAGCCGCGGCGCCCACTCTTTCCGCGCCGTCACCGTTCCCGAGTTGACCCAG
CAGATGTTTGACCCCAAGAACATGATGGCTGCCTCGGATTTCCGAAACGGTCGATATCTAACGTGC
TCTGCCATCTTGTAAGCCTTATGTTCACTCACTTC--
AACAAACCCAACTGCTAACCCCGACAAACTAGCCGTGGCAAG-------

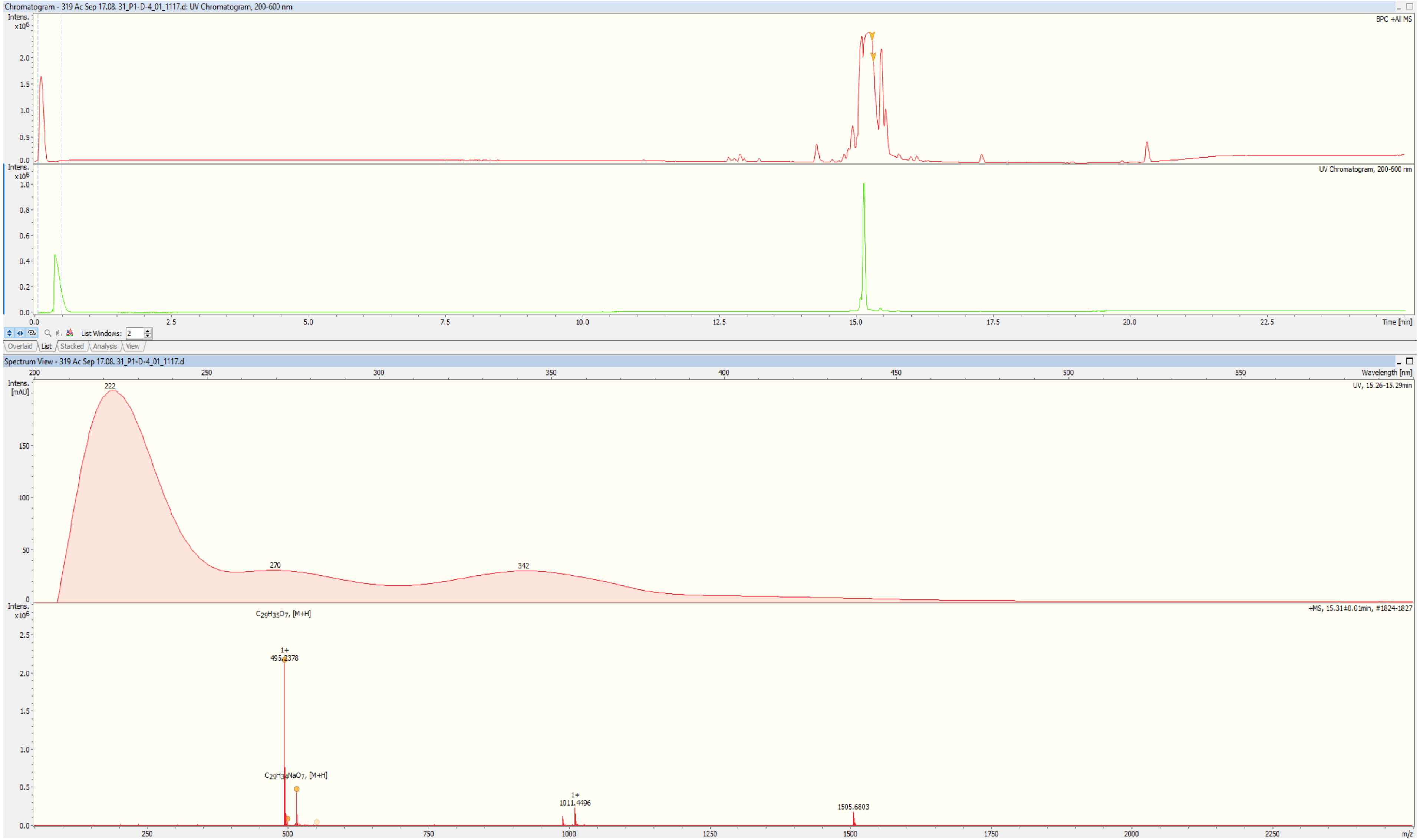

Figure S1: HRESIMS data of minutellin E (1).

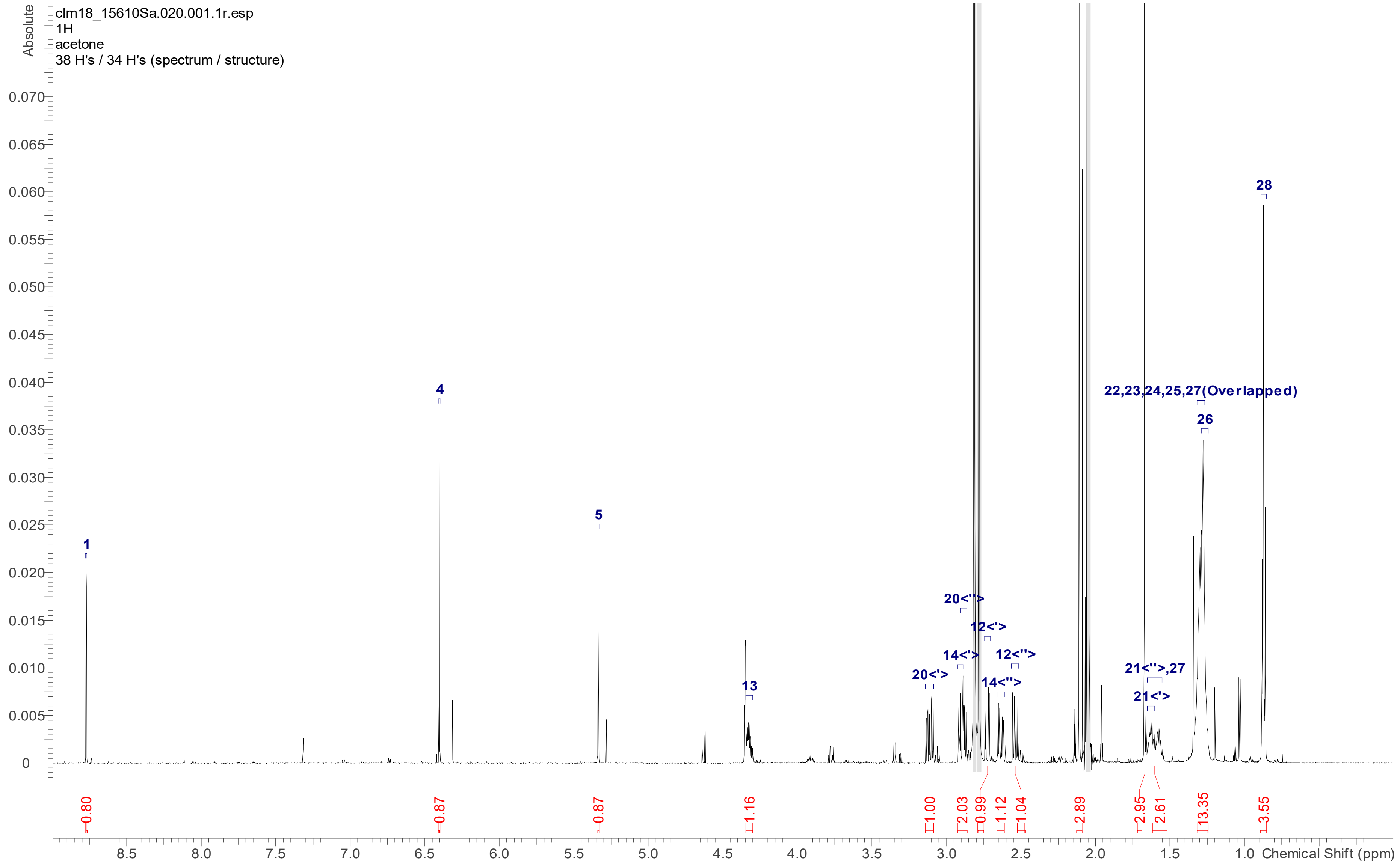

Figure S2: ^{1}H NMR spectrum (700 MHz, acetone-d_6) of minutellin E (1).

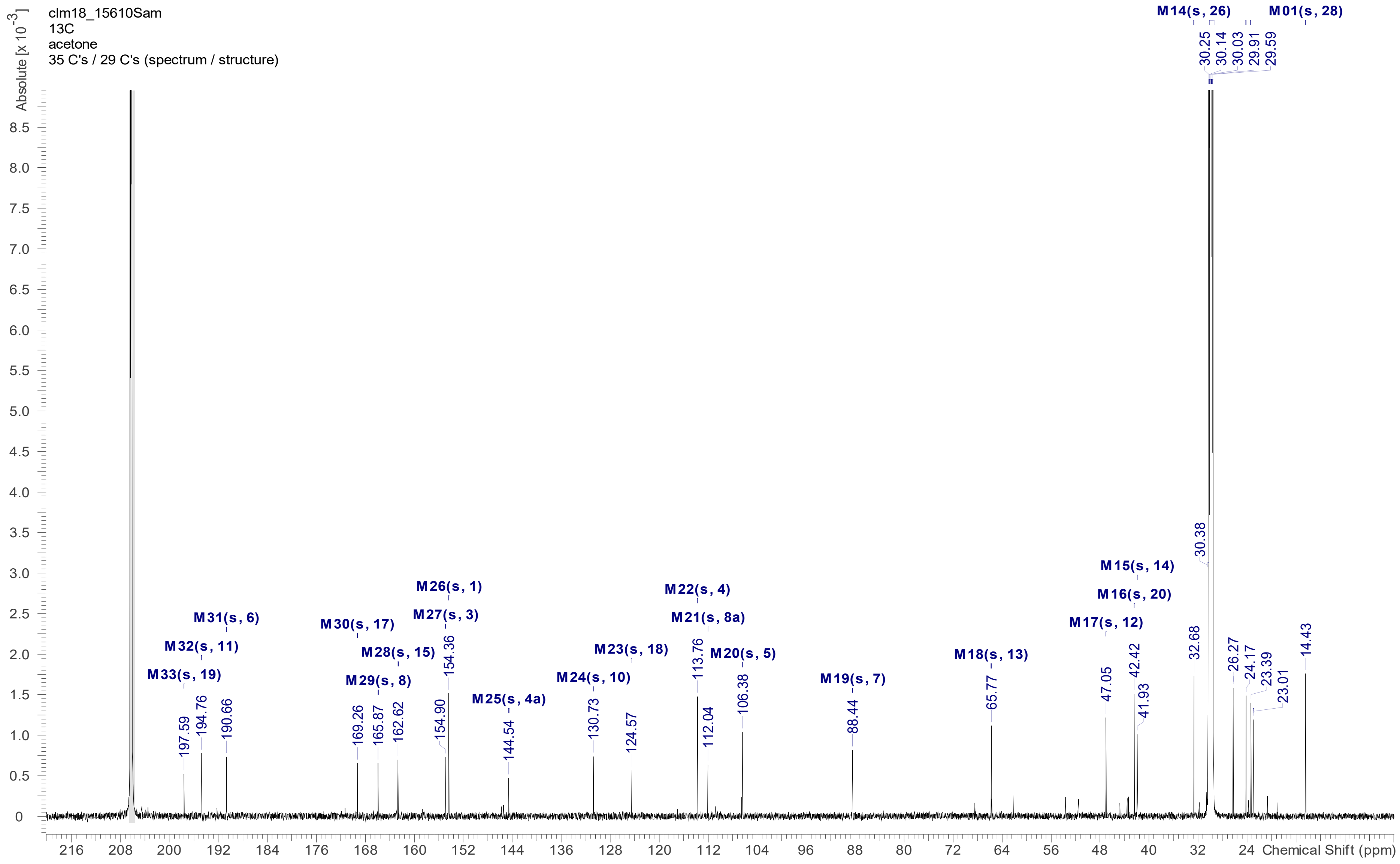

Figure S3: ^{13}C NMR spectrum (175 MHz, acetone-d_6) of minutellin E (**1**).

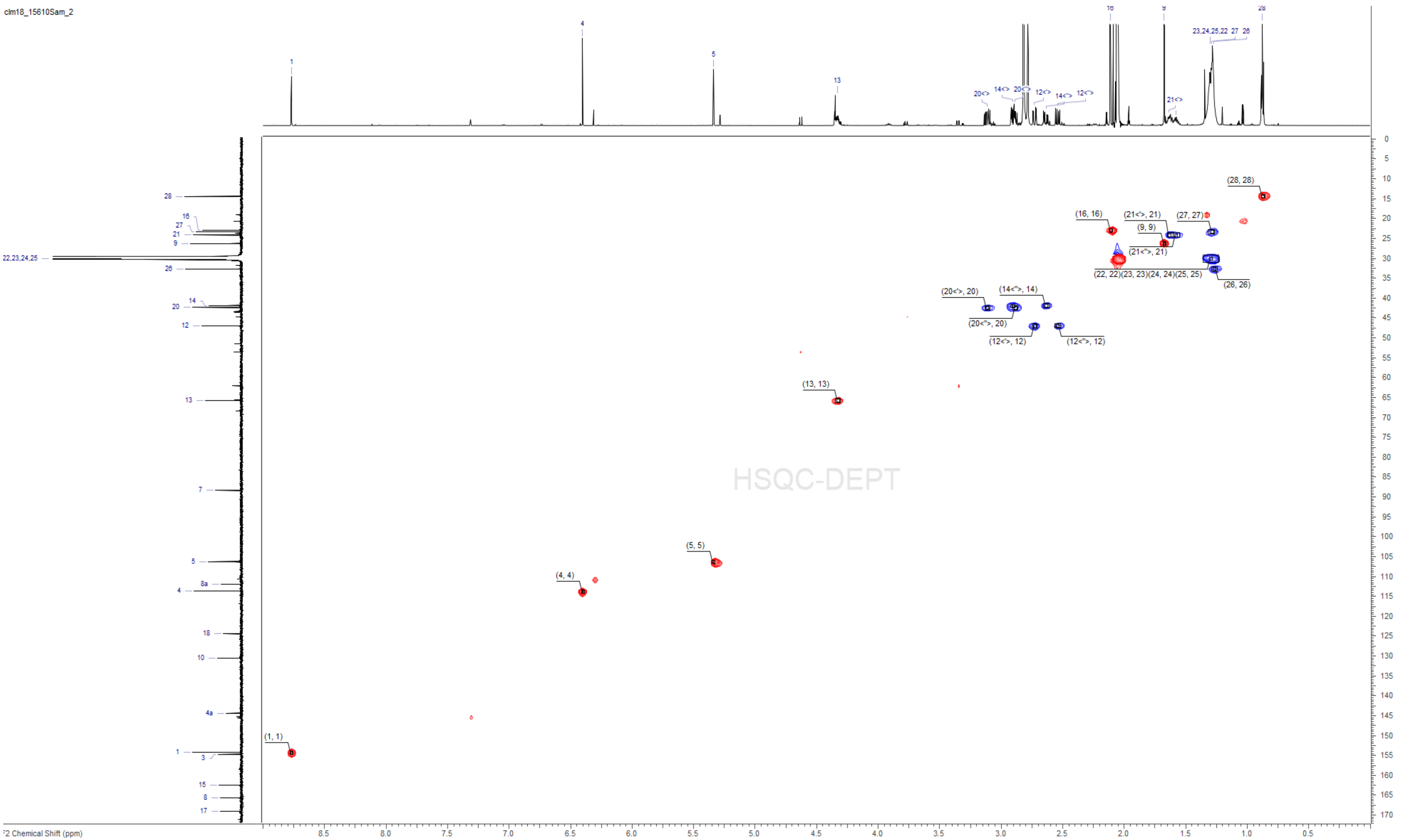

Figure S4: HSQC NMR spectrum (700 MHz, acetone-d_6) of minutellin E (**1**).

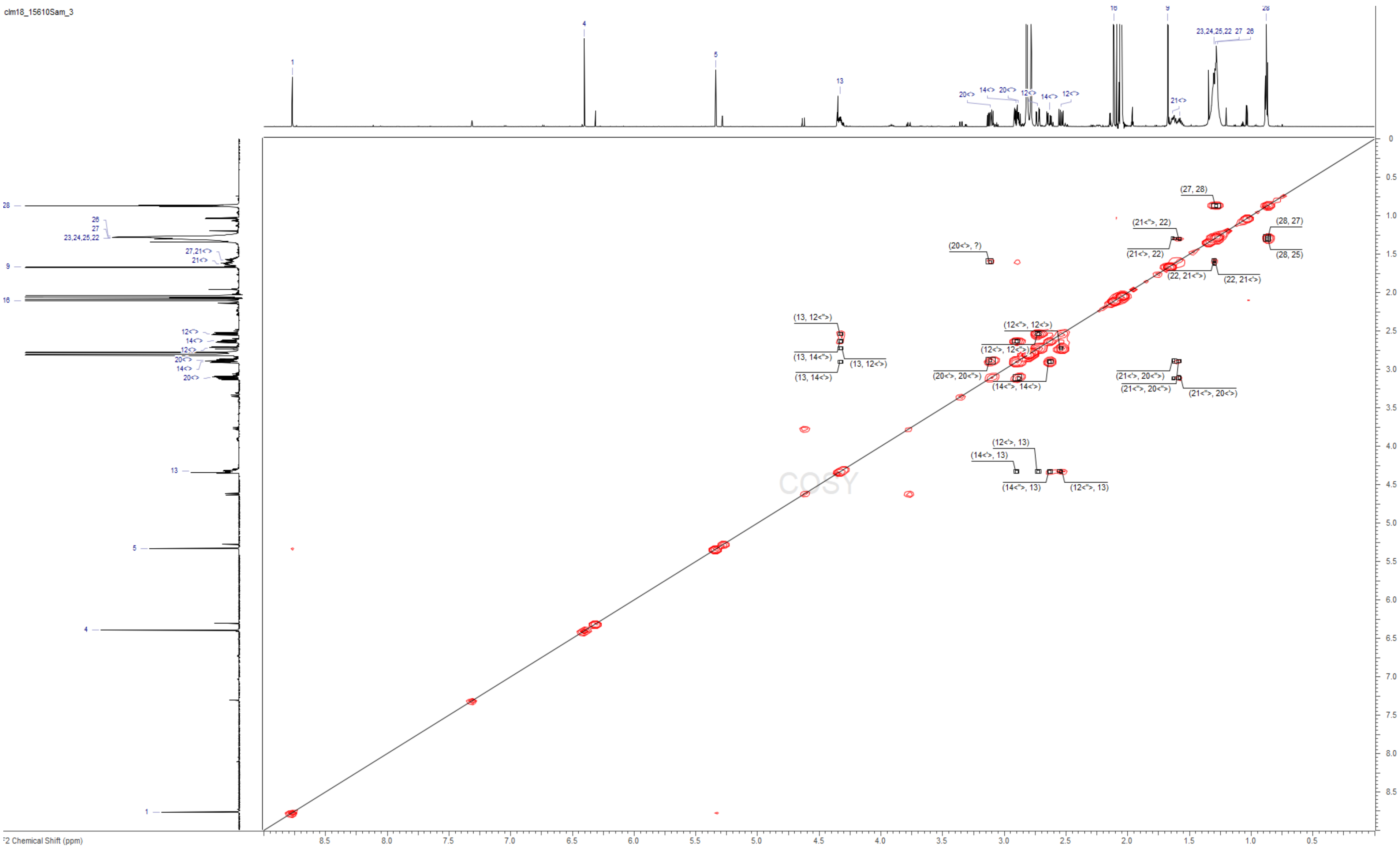

Figure S5: COSY NMR spectrum (700 MHz, acetone-d_6) of minutellin E (**1**).

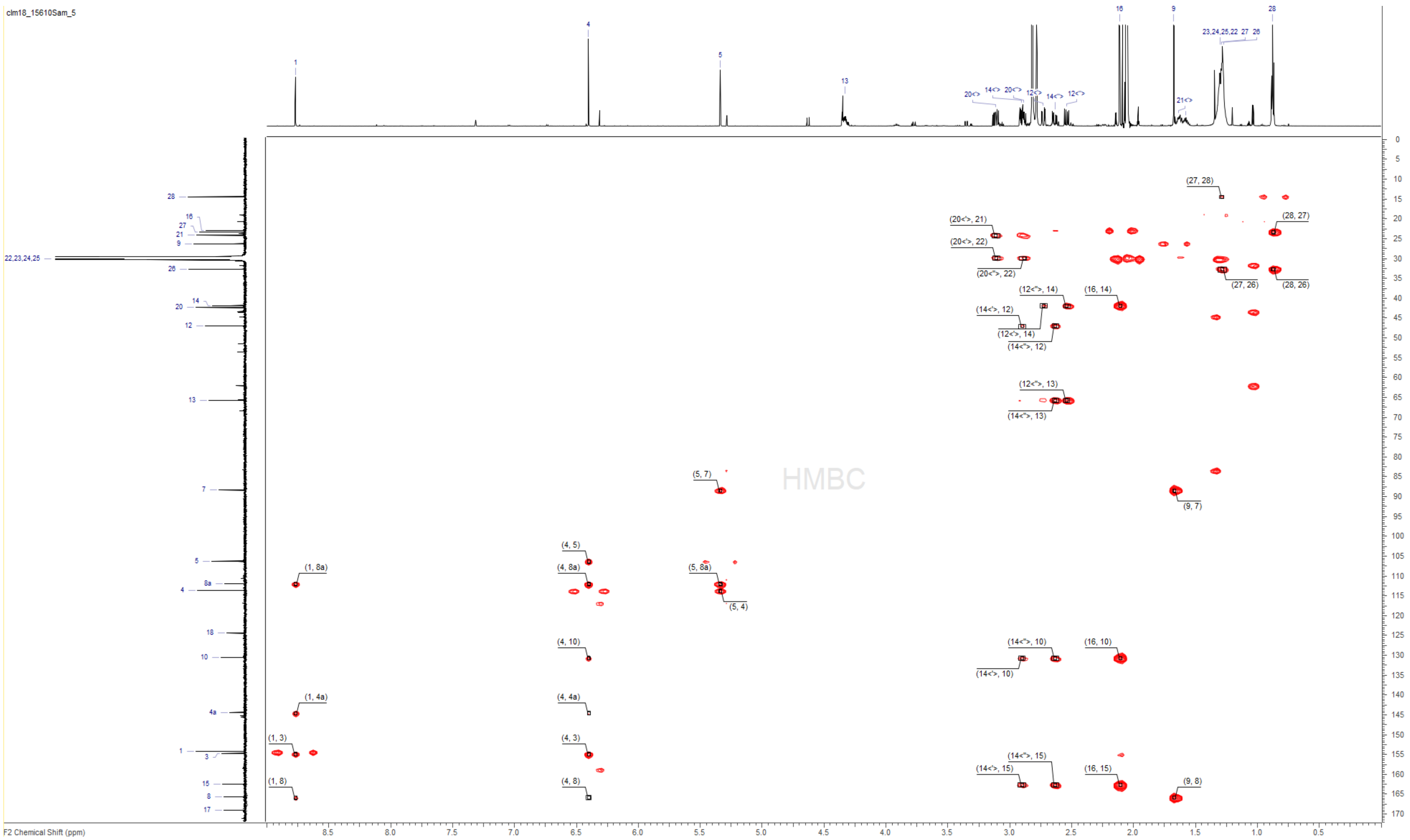

Figure S6: HMBC NMR spectrum (700 MHz, acetone-d_6) of minutellin E (**1**).

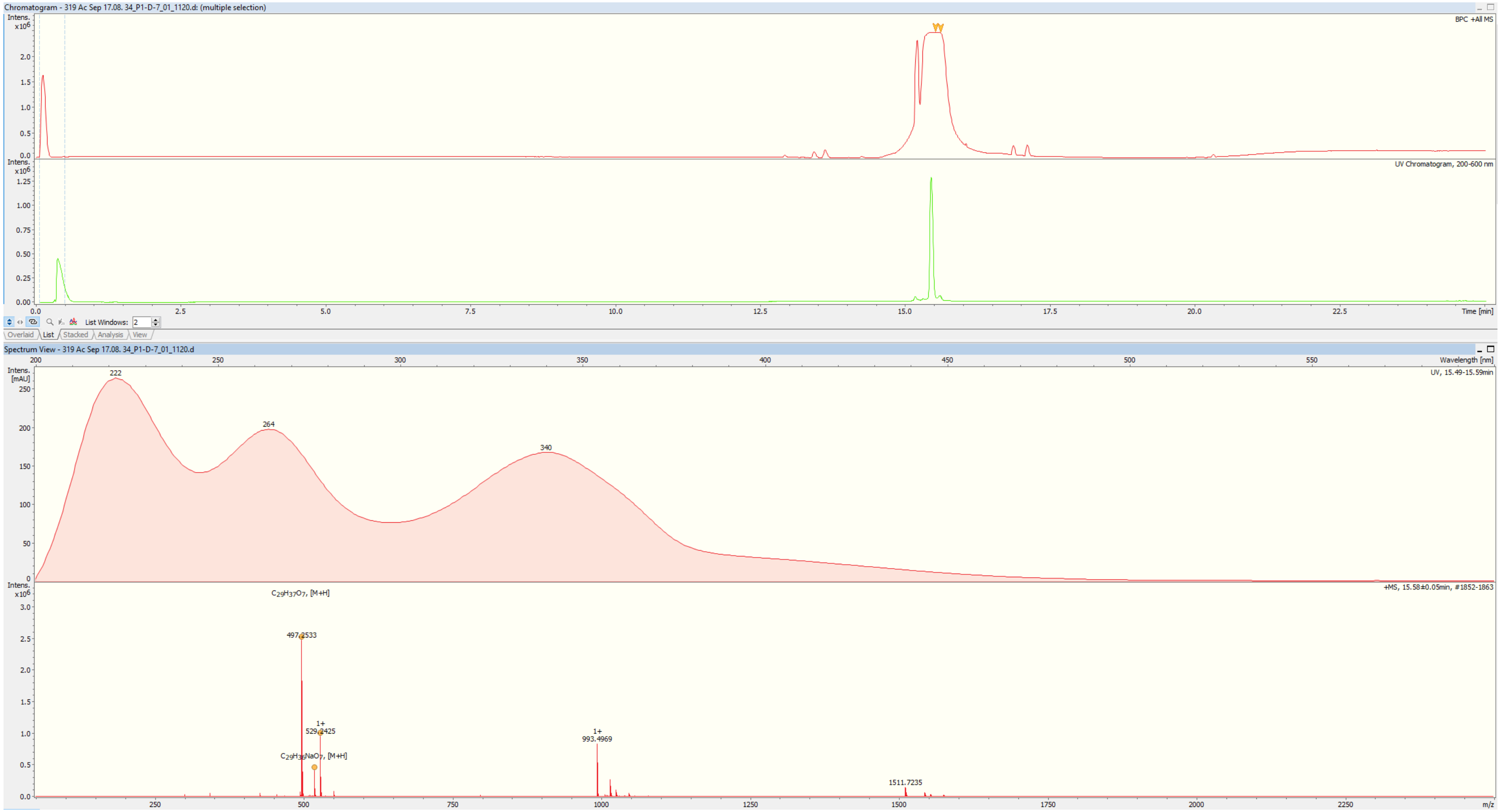

Figure S7: HRESIMS data of minutellin F (**2**).

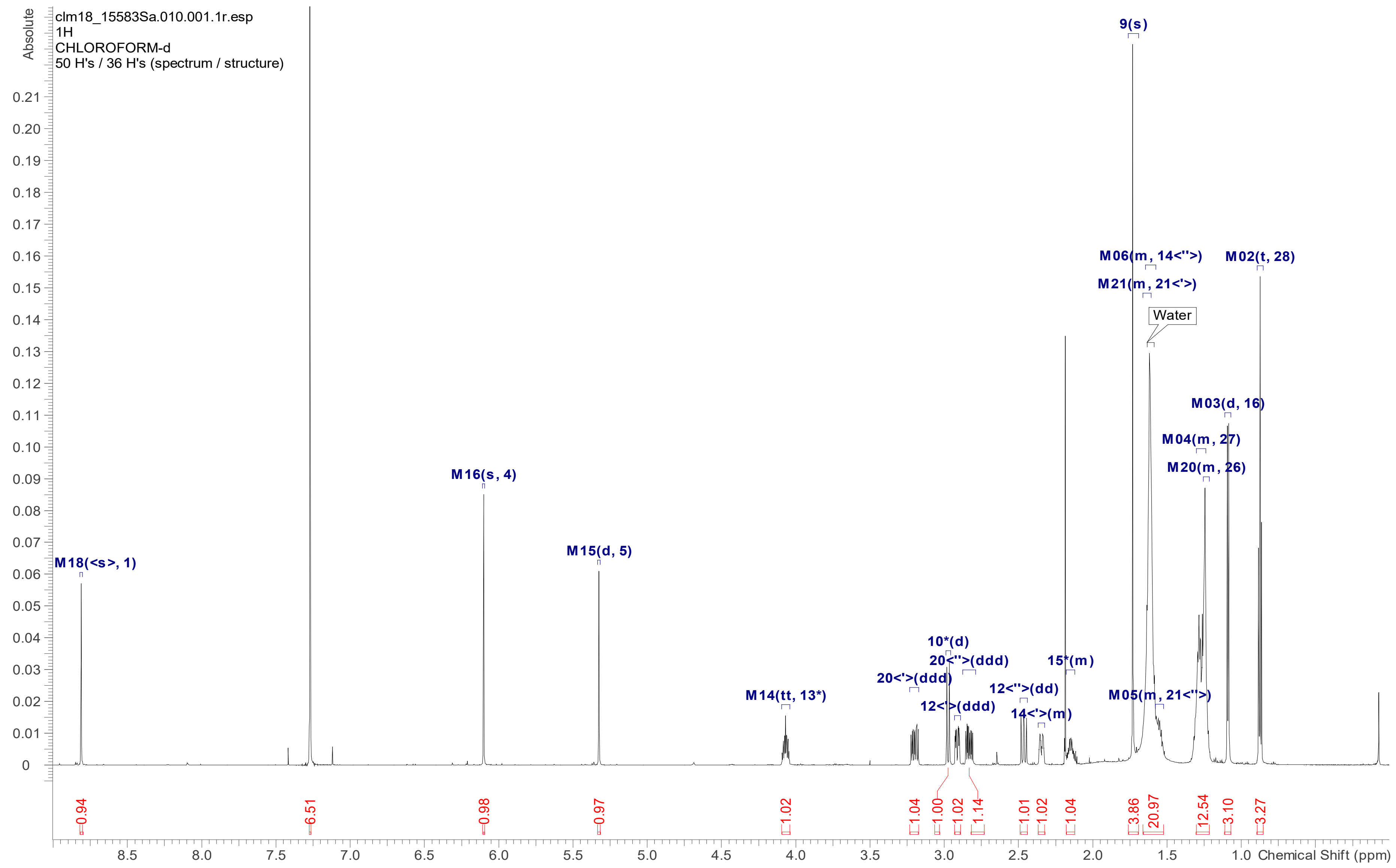

Figure S8: ^{1}H NMR spectrum (700 MHz, $CHCl_3$-*d*) of minutellin F (**2**).

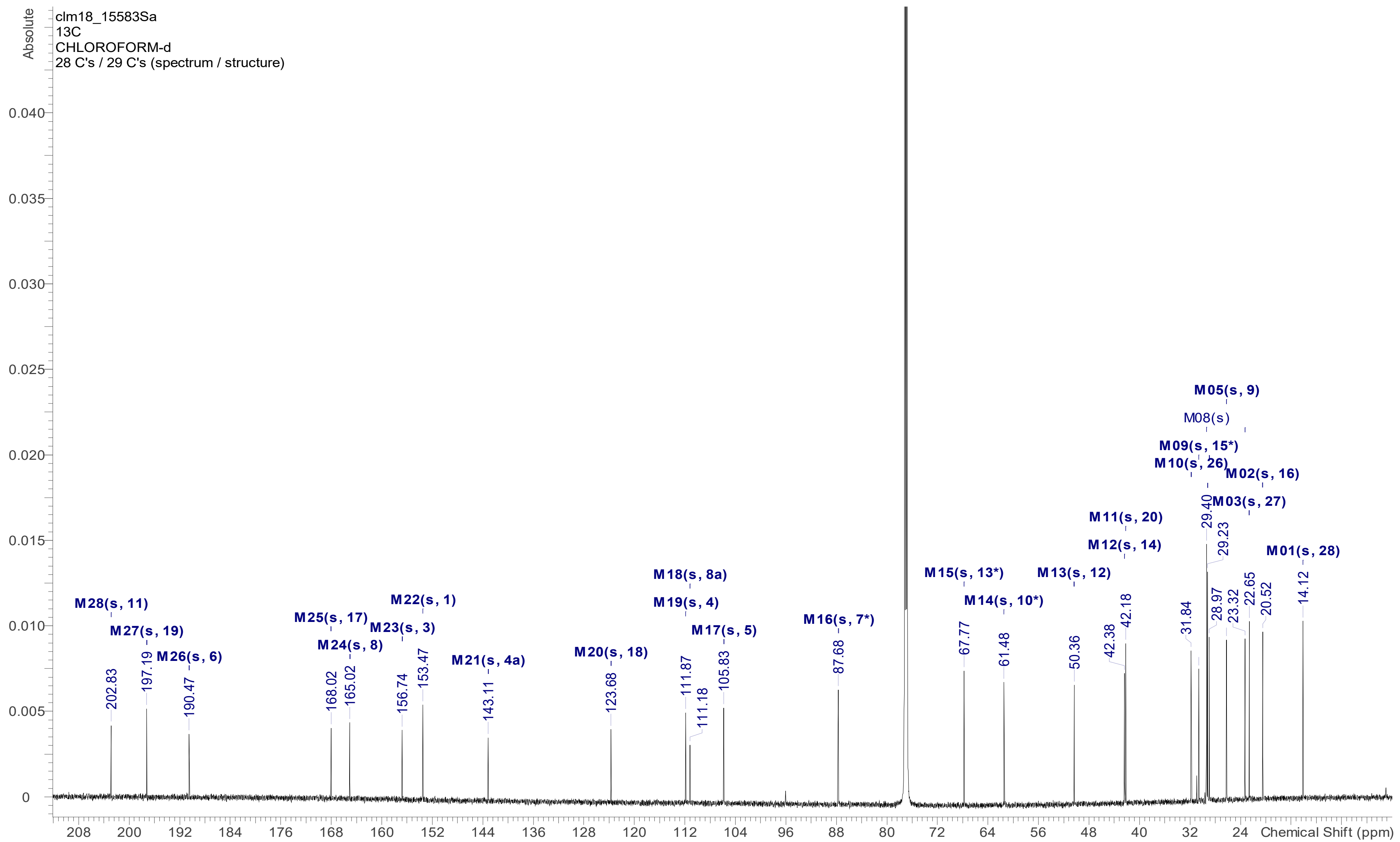

Figure S9: ^{13}C NMR spectrum (175 MHz, $CHCl_3$-*d*) of minutellin F (**2**).

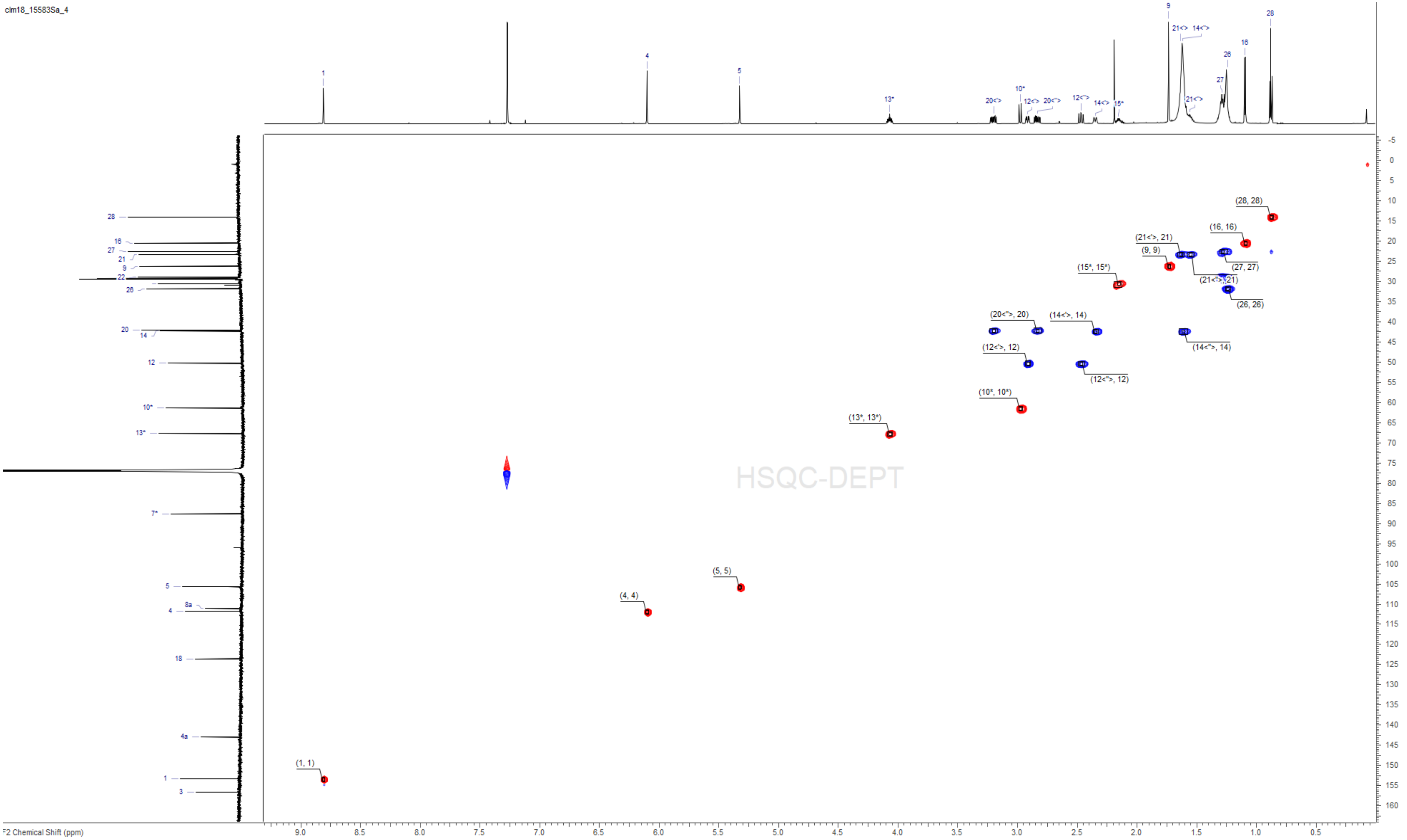

Figure S10: HSQC NMR spectrum (700 MHz, $CHCl_3$-*d*) of minutellin F (**2**).

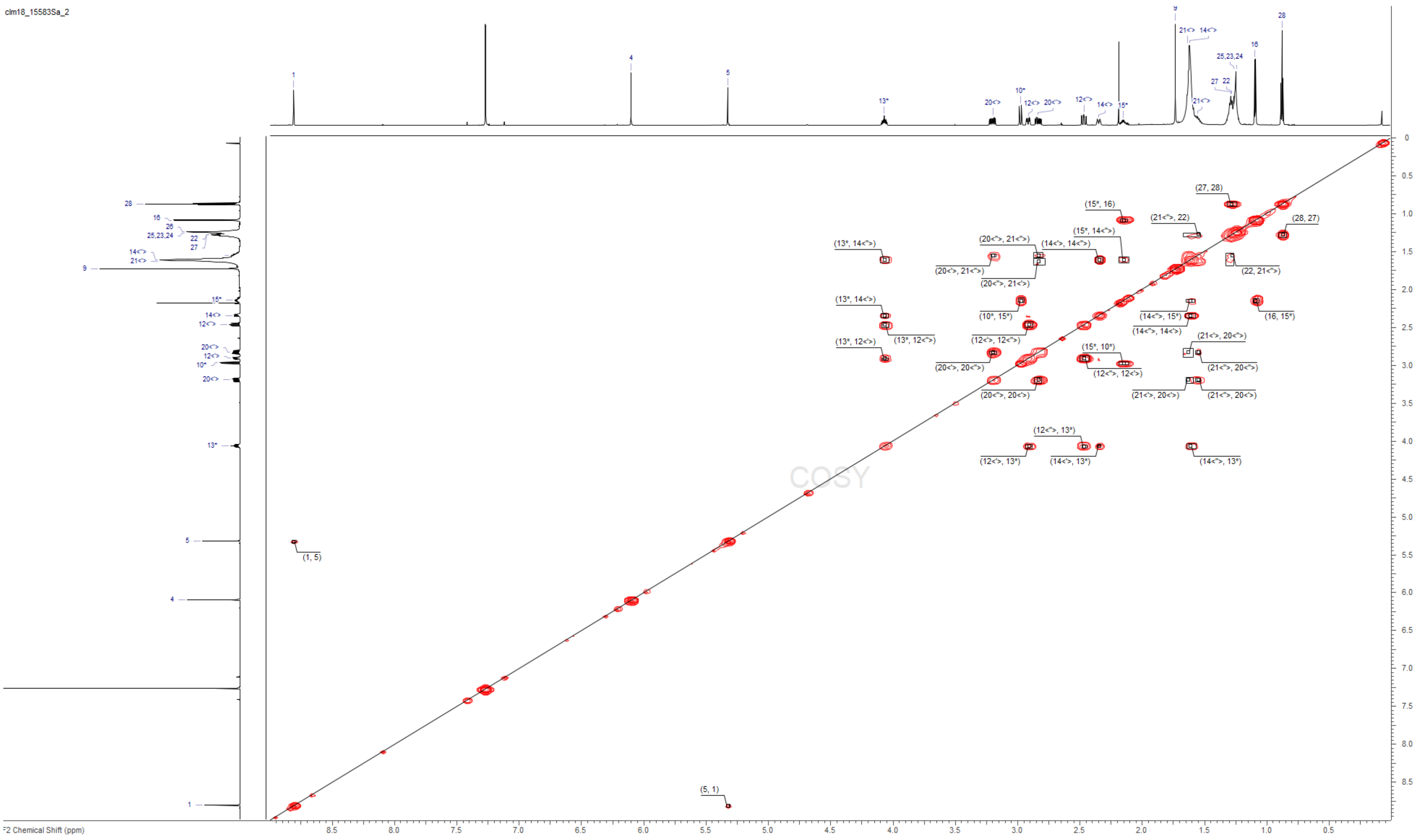

Figure S11: COSY NMR spectrum (700 MHz, $CHCl_3$-*d*) of minutellin F (**2**).

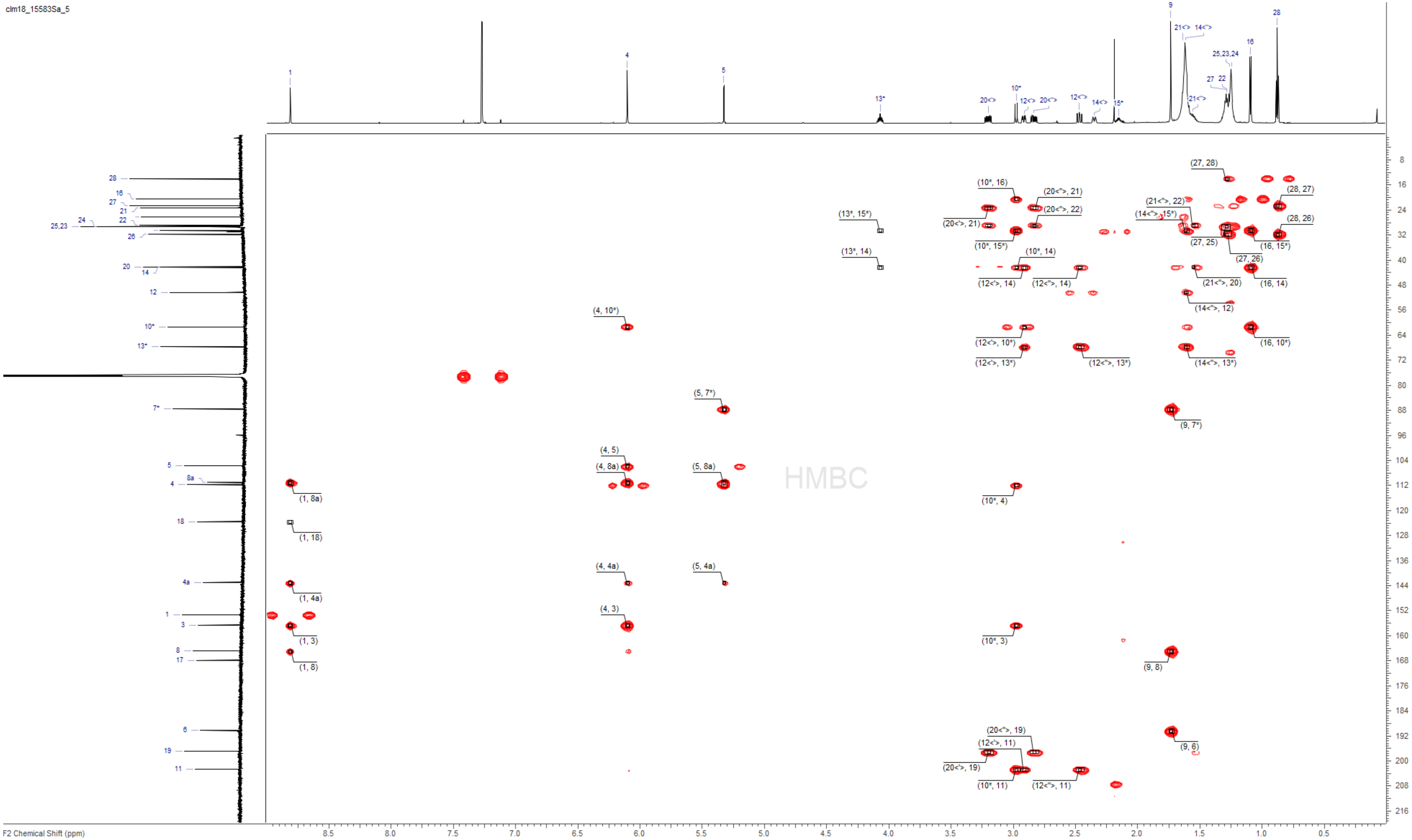

Figure S12: HMBC NMR spectrum (700 MHz, $CHCl_3$-*d*) of minutellin F (**2**).

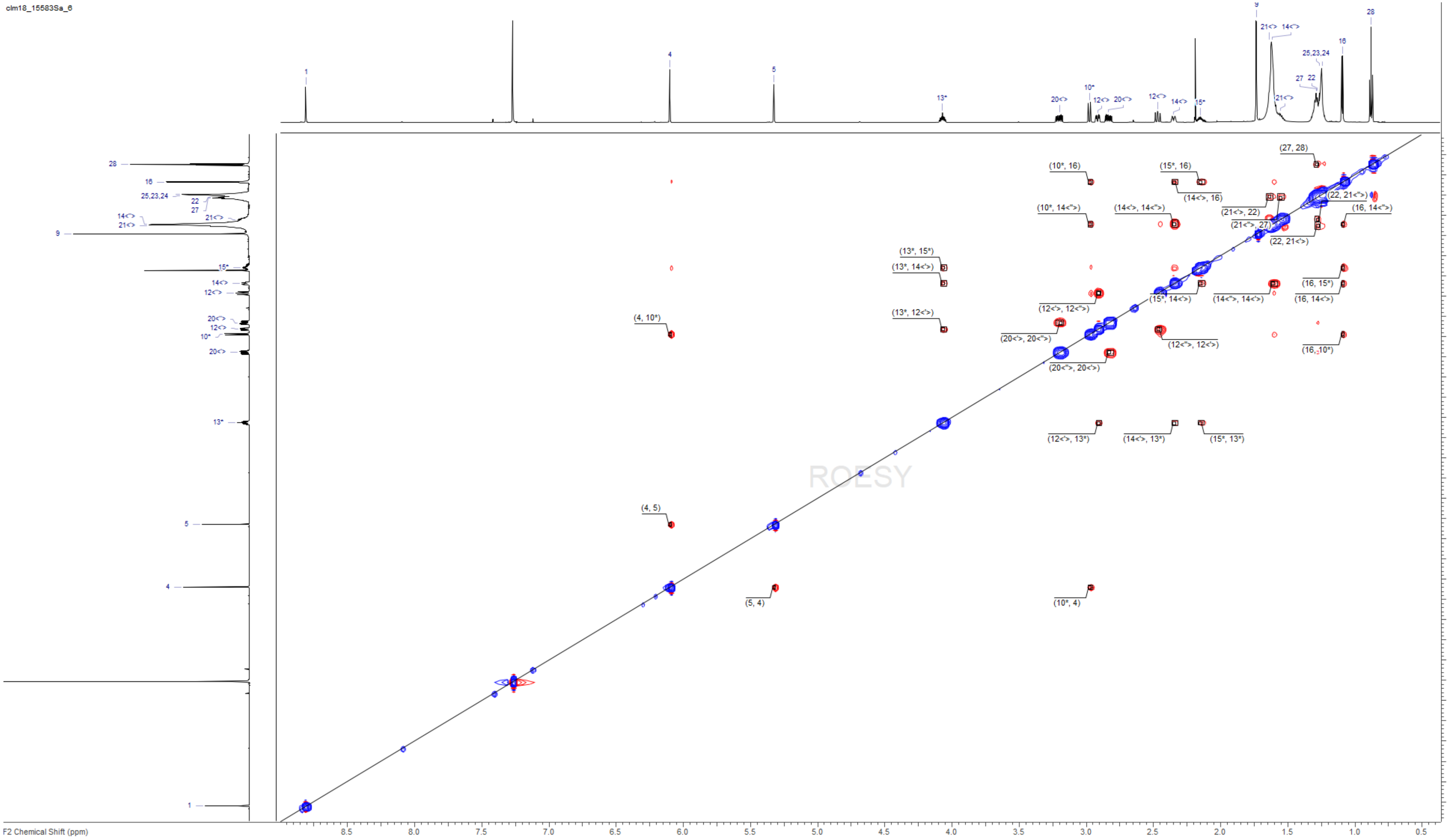

Figure S13: ROESY NMR spectrum (700 MHz, $CHCl_3$-*d*) of minutellin F (**2**).

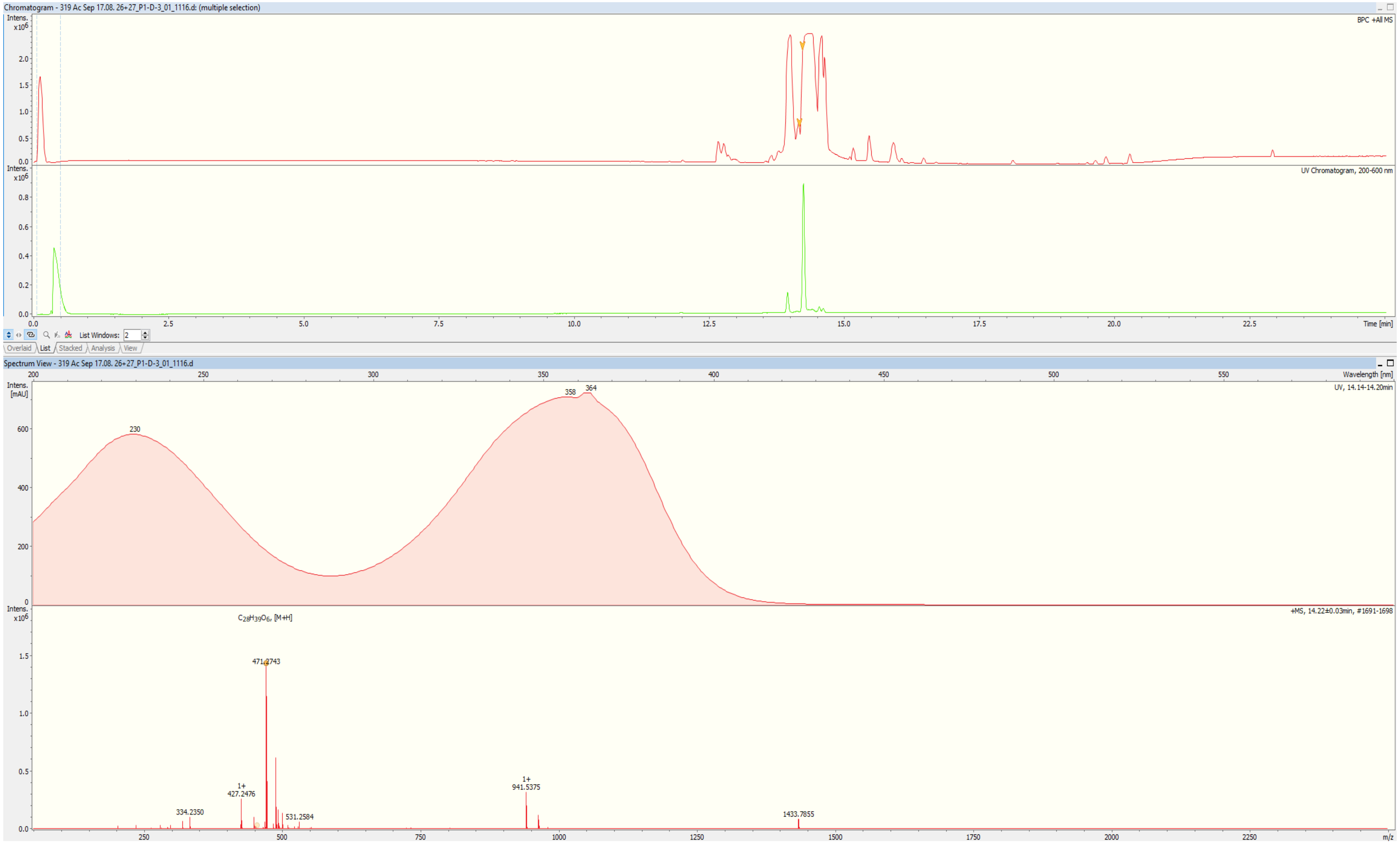

Figure S14: HRESIMS data of minutellin G (**3**).

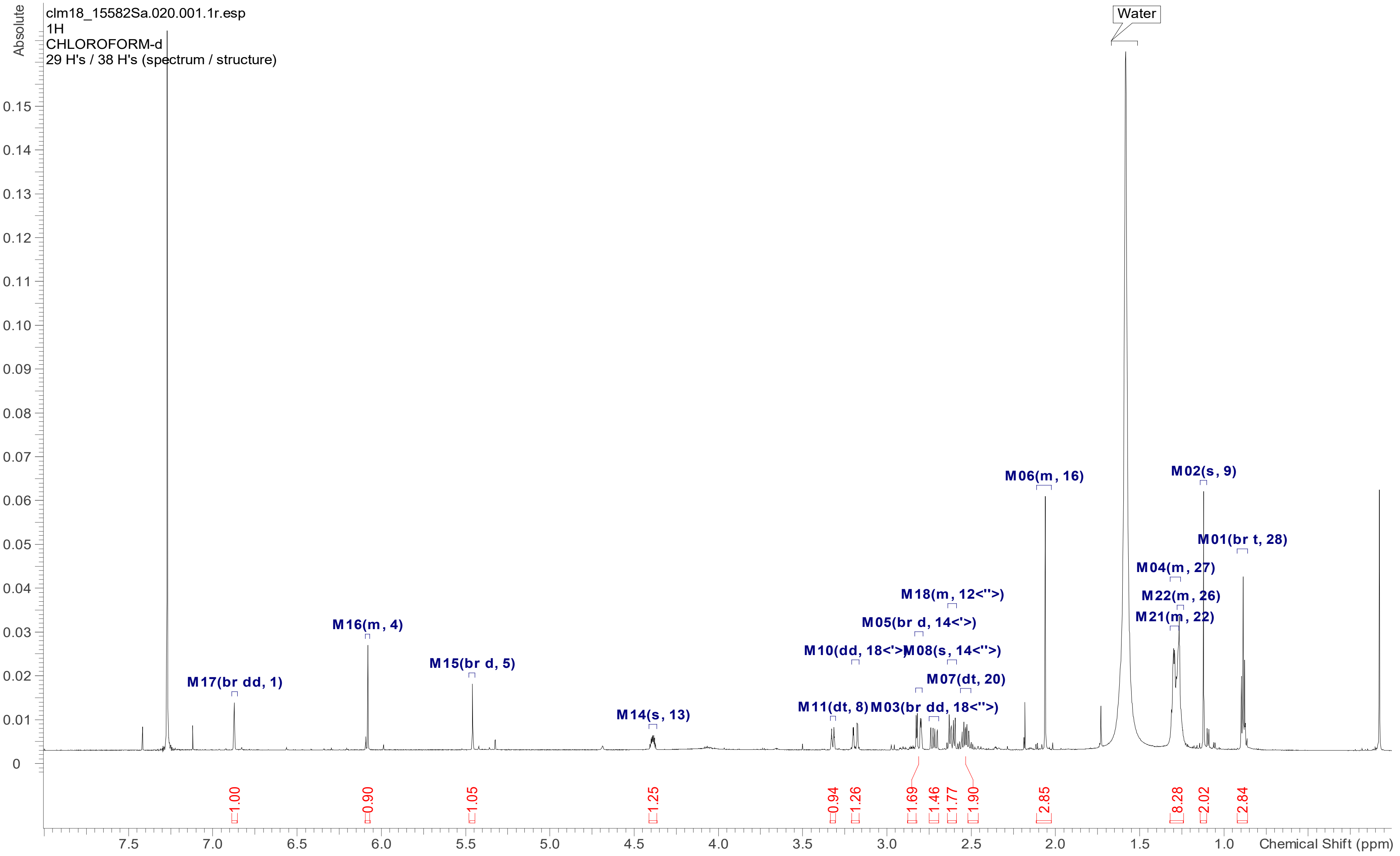

Figure S15: ^{1}H NMR spectrum (700 MHz, $CHCl_3$-*d*) of minutellin G (**3**).

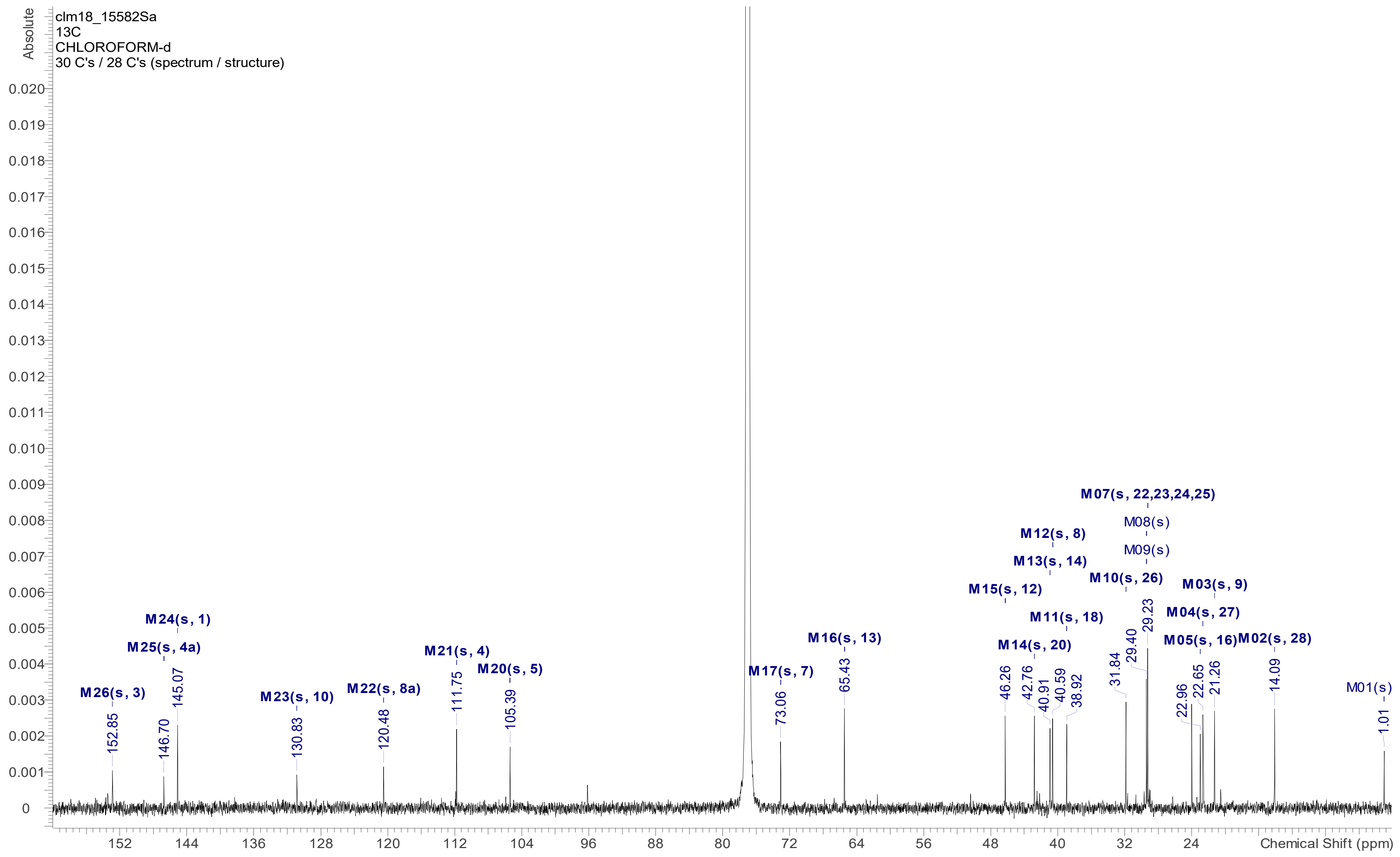

Figure S16: ^{13}C NMR spectrum (175 MHz, $CHCl_3$-*d*) of minutellin G (**3**).

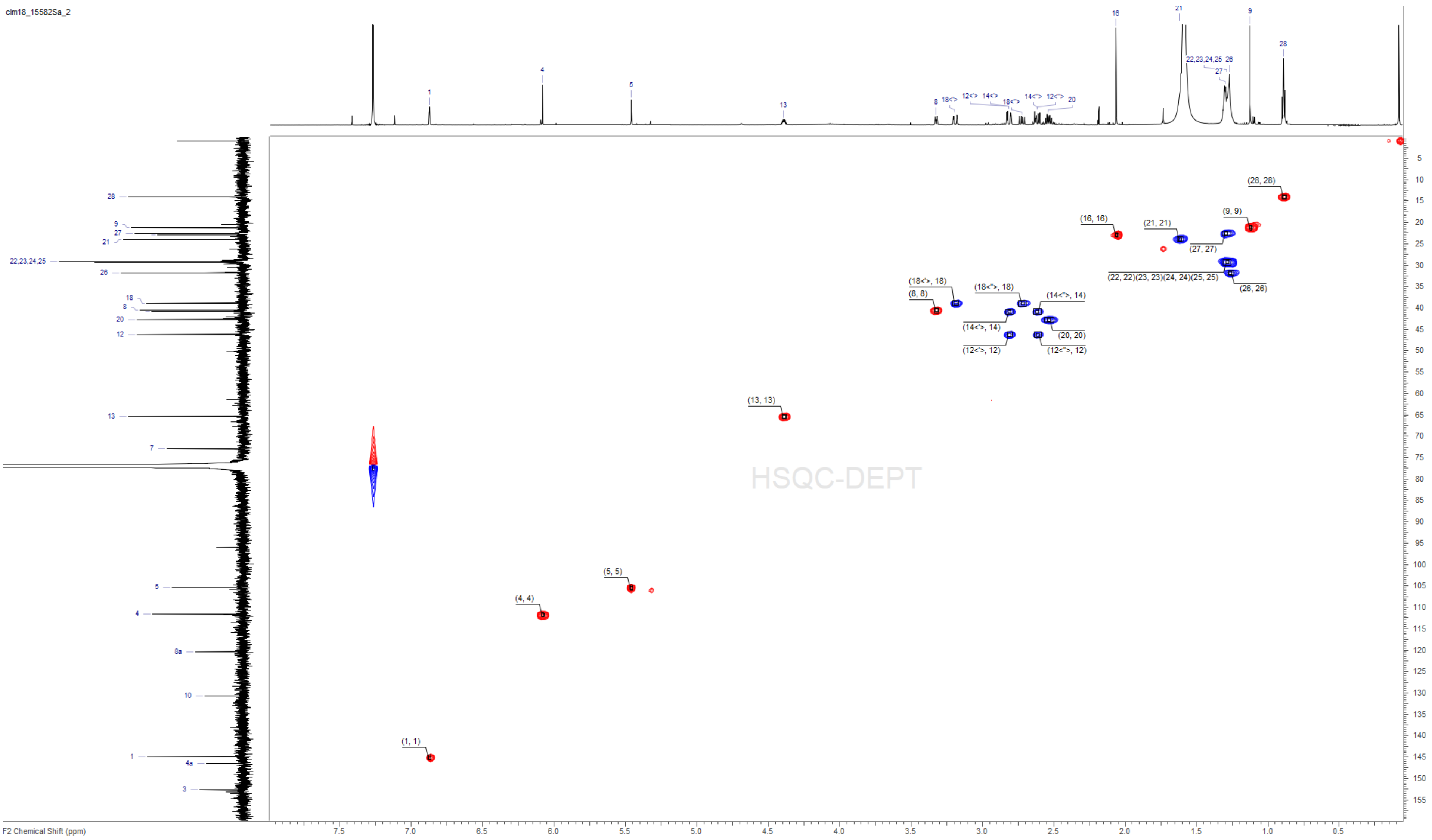

Figure S17: HSQC NMR spectrum (700 MHz, $CHCl_3$-*d*) of minutellin G (**3**).

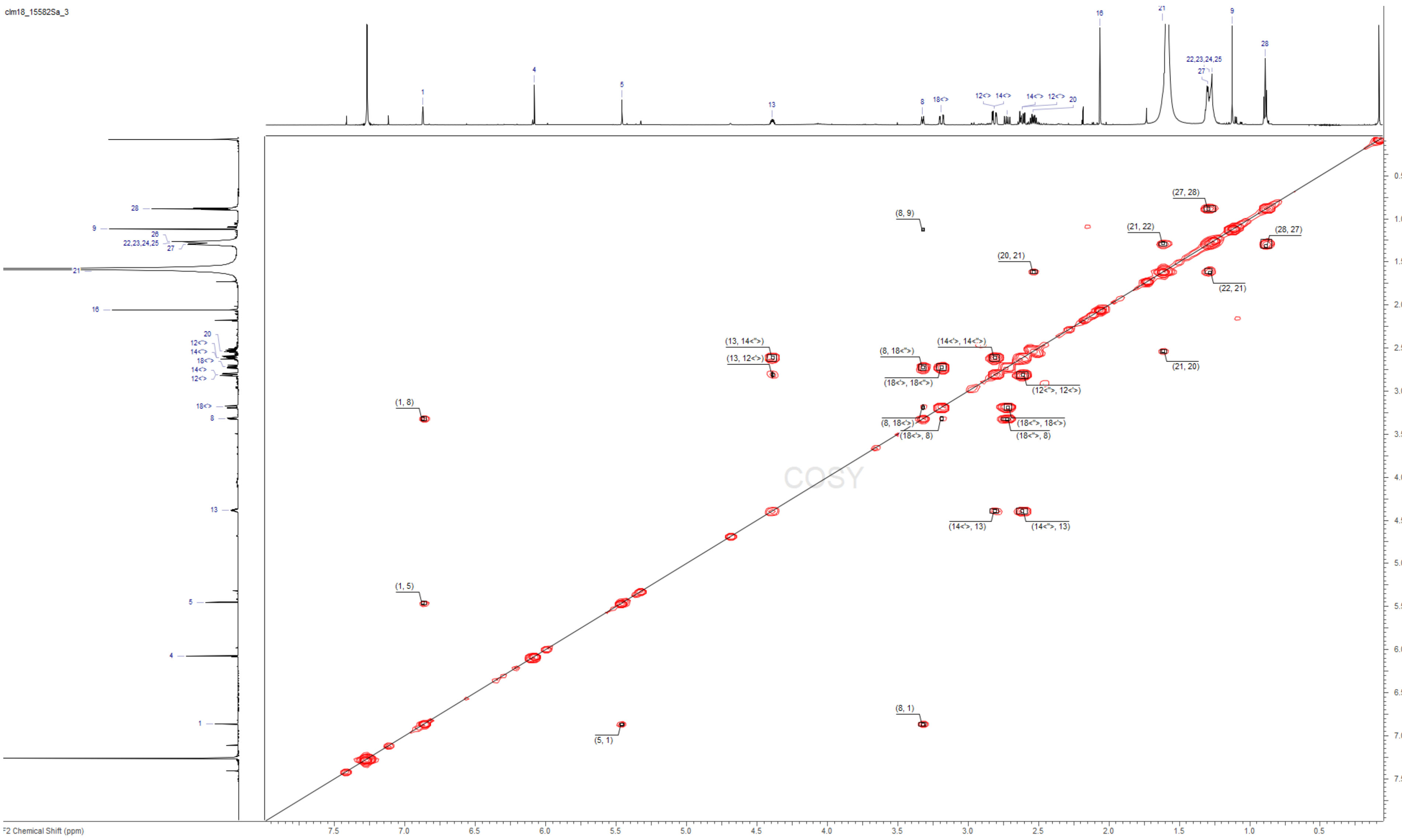

Figure S18: COSY NMR spectrum (700 MHz, $CHCl_3$-*d*) of minutellin G (**3**).

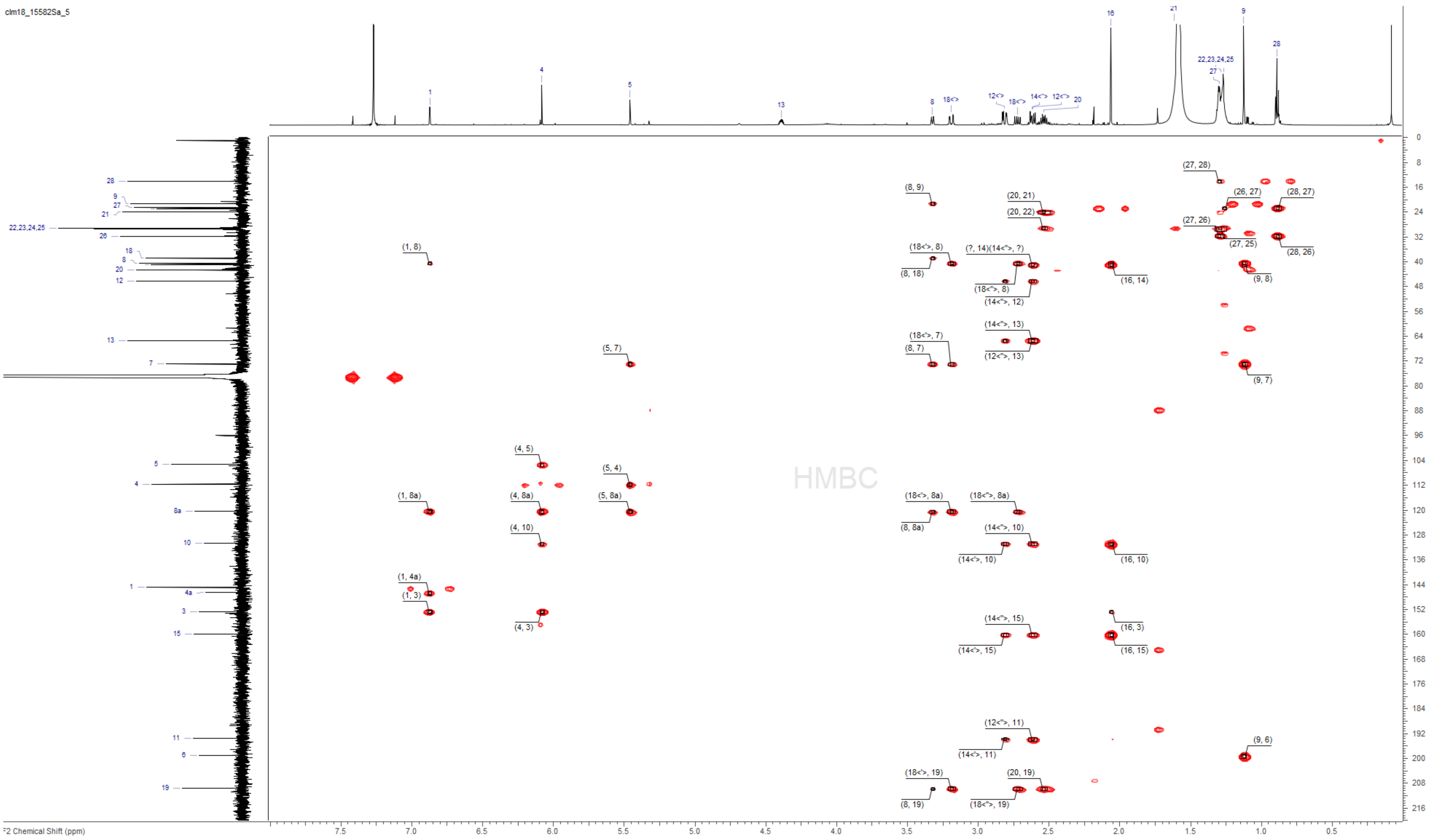

Figure S19: HMBC NMR spectrum (700 MHz, $CHCl_3$-*d*) of minutellin G (**3**).

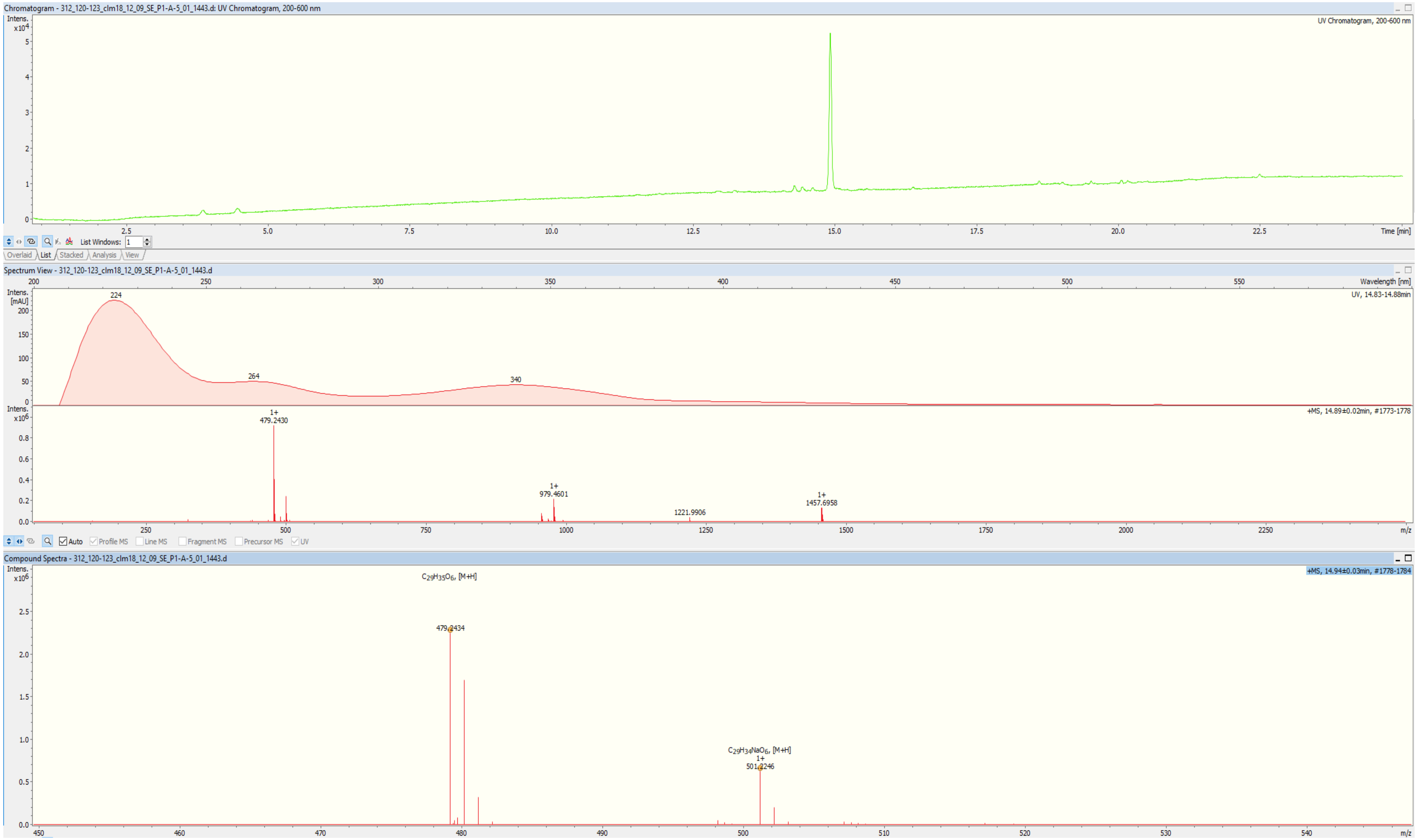

Figure S20: HRESIMS data of minutellin H (**4**).

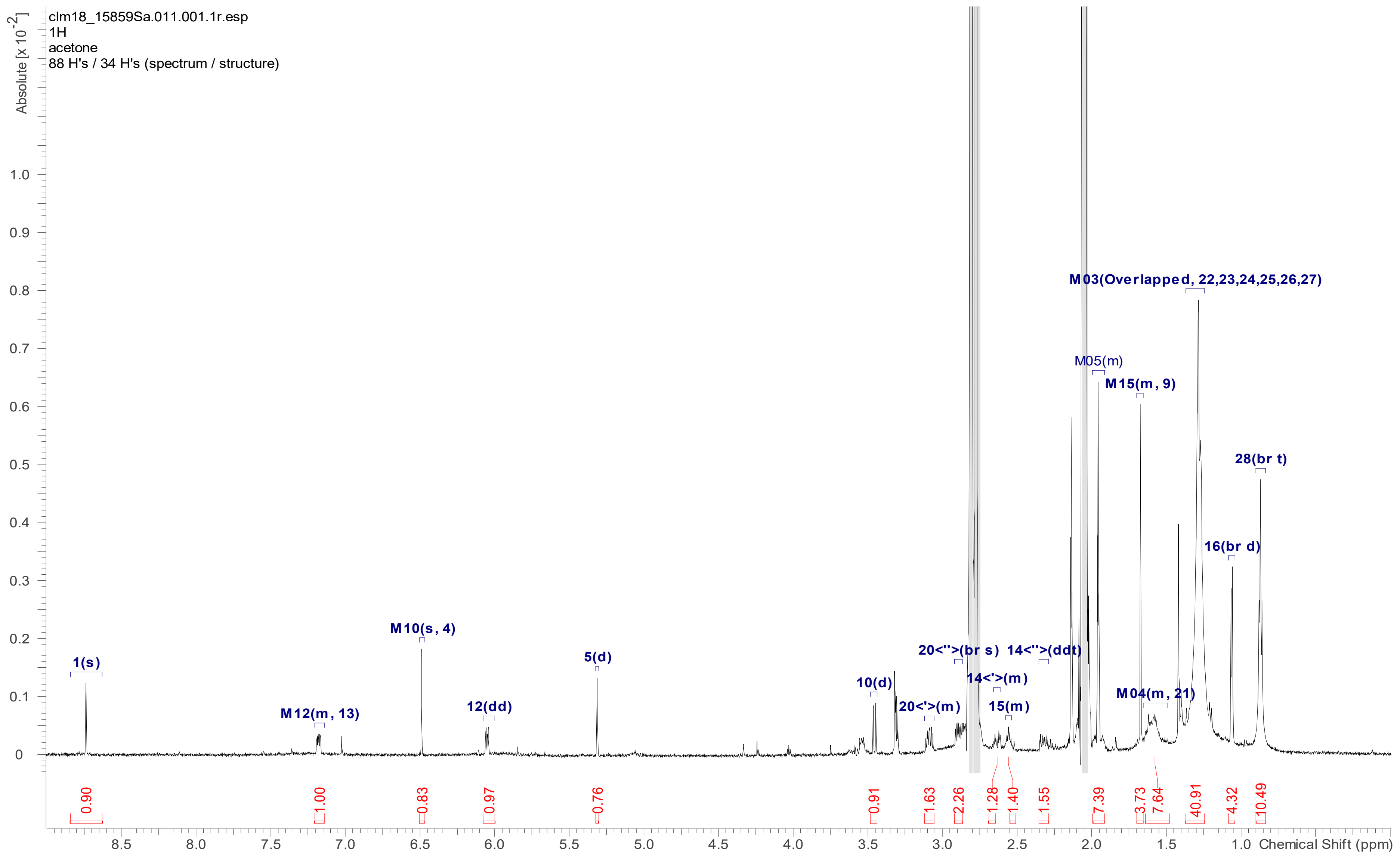

Figure S21: ^{1}H NMR spectrum (700 MHz, acetone-d_6) of minutellin H (**4**).

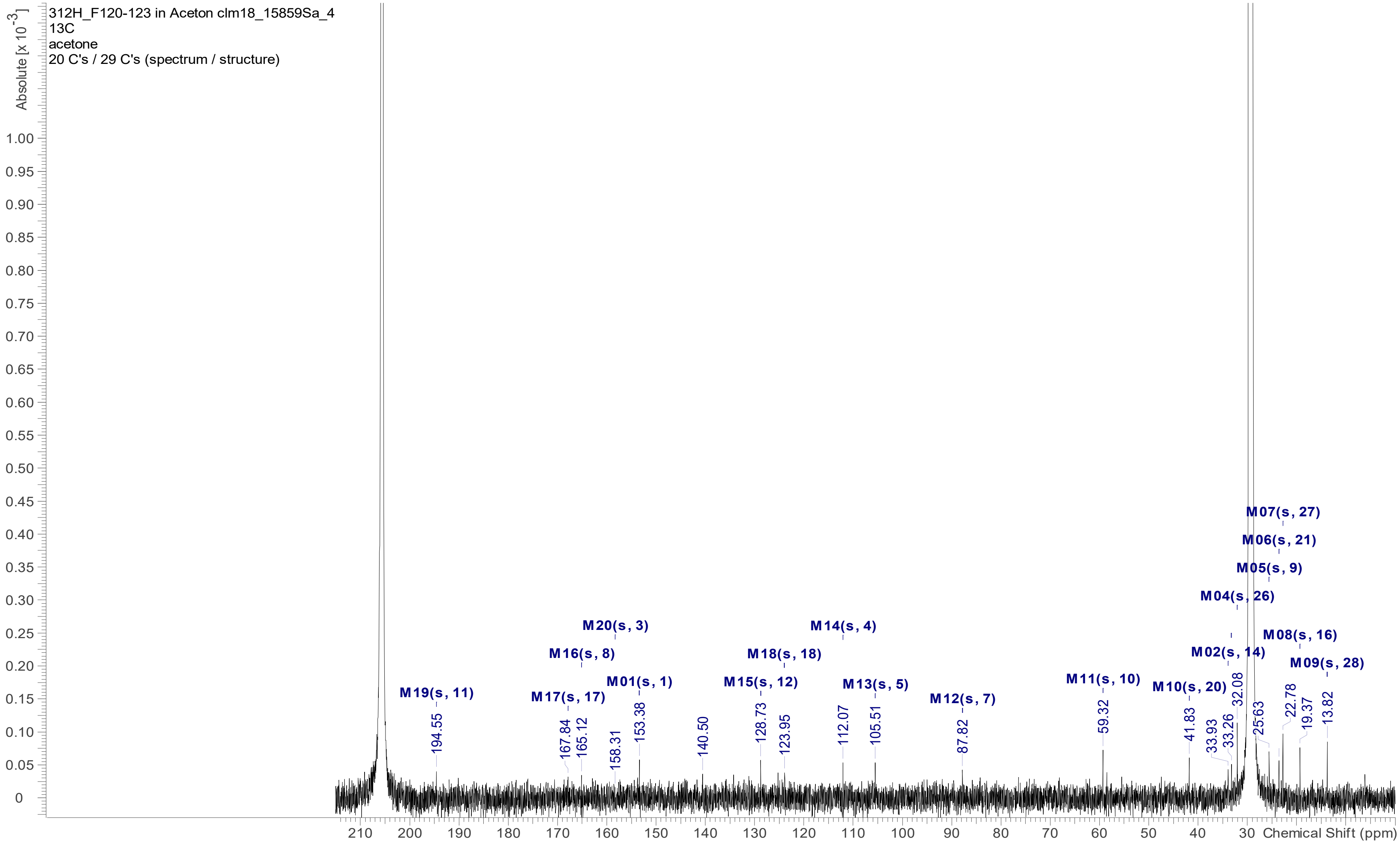

Figure S22: ^{13}C NMR spectrum (175 MHz, acetone-d_6) of minutellin H (**4**).

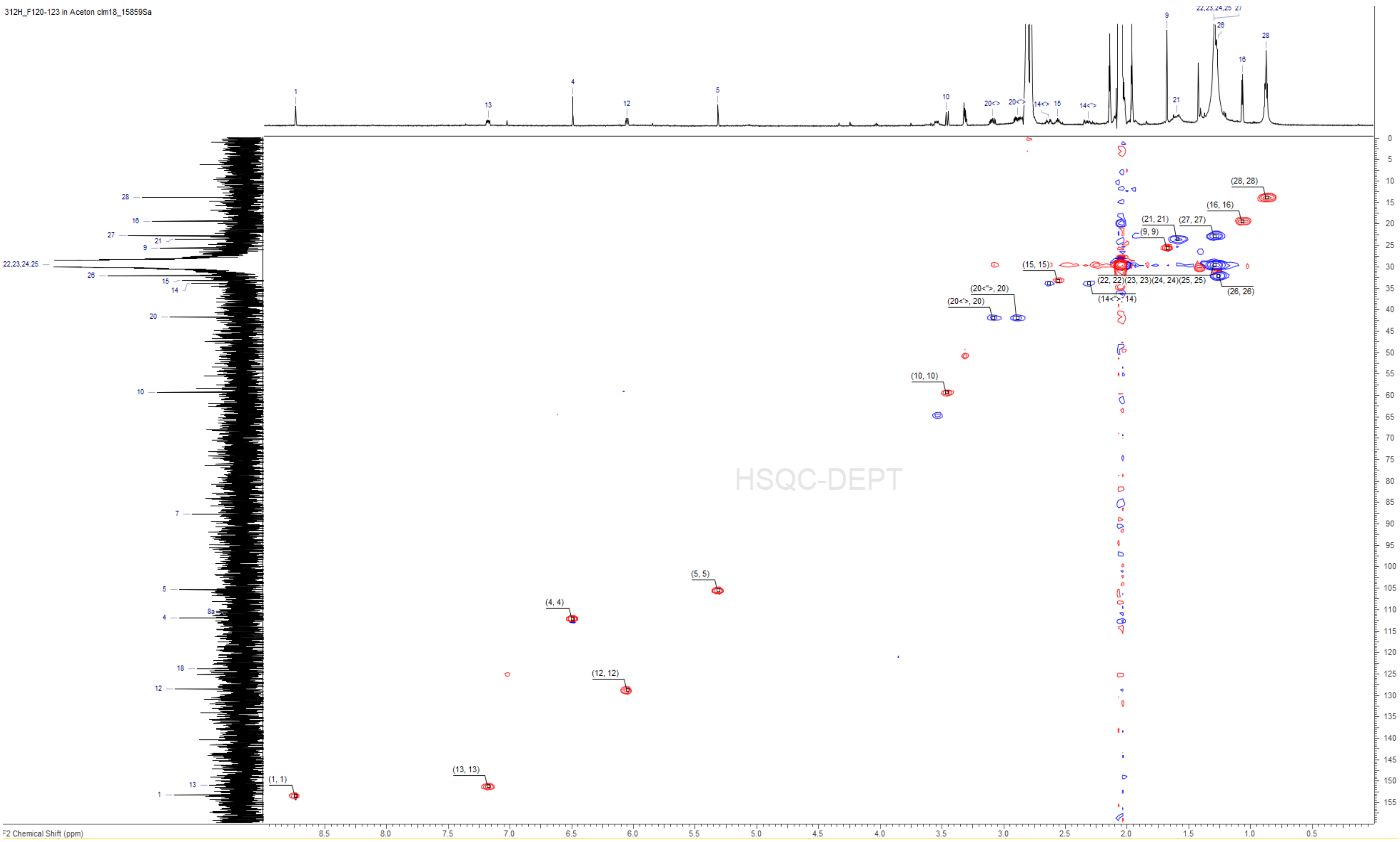

Figure S23: HSQC NMR spectrum (700 MHz, acetone-d_6) of minutellin H (**4**).

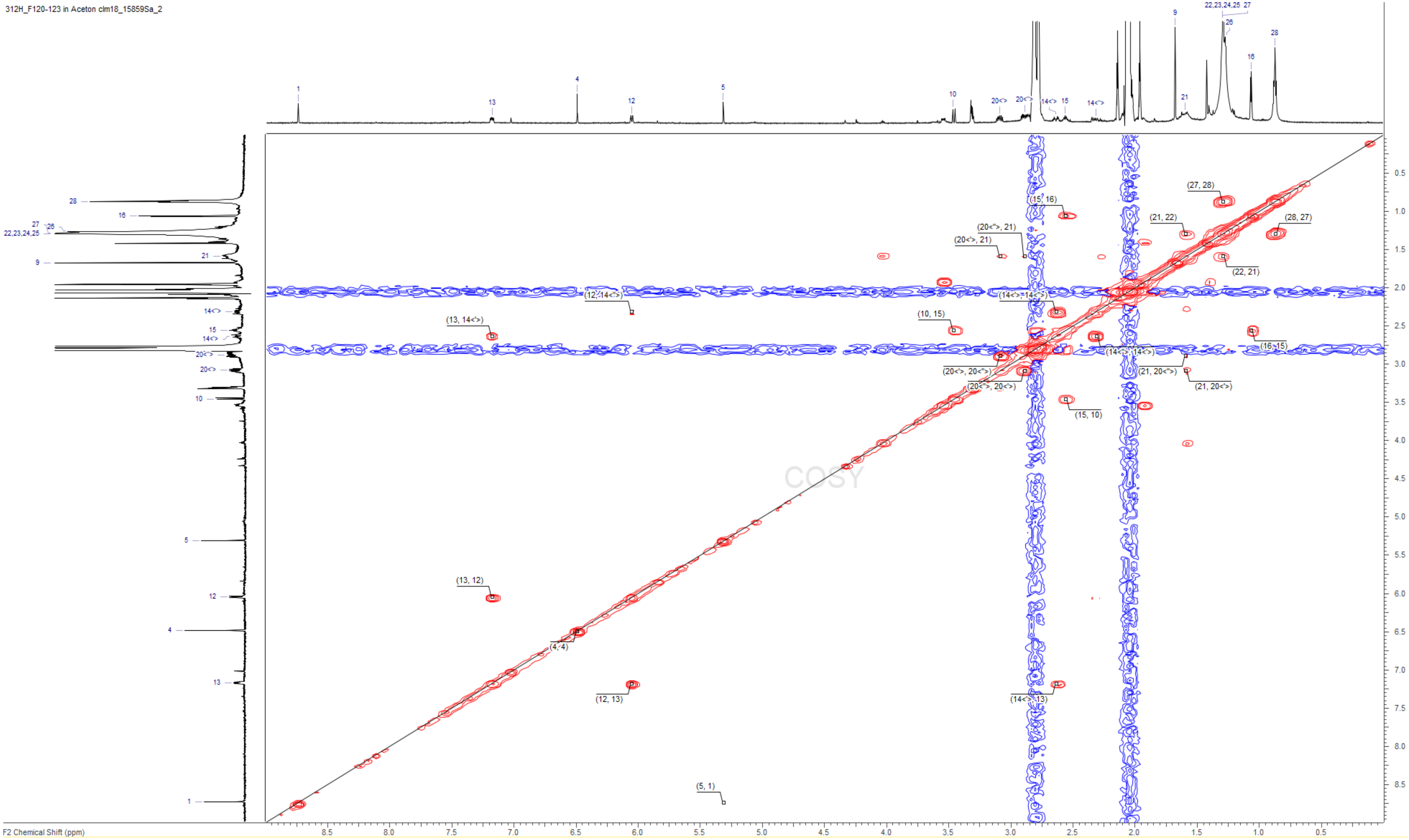

Figure S24: COSY NMR spectrum (700 MHz, acetone-d_6) of minutellin H (**4**).

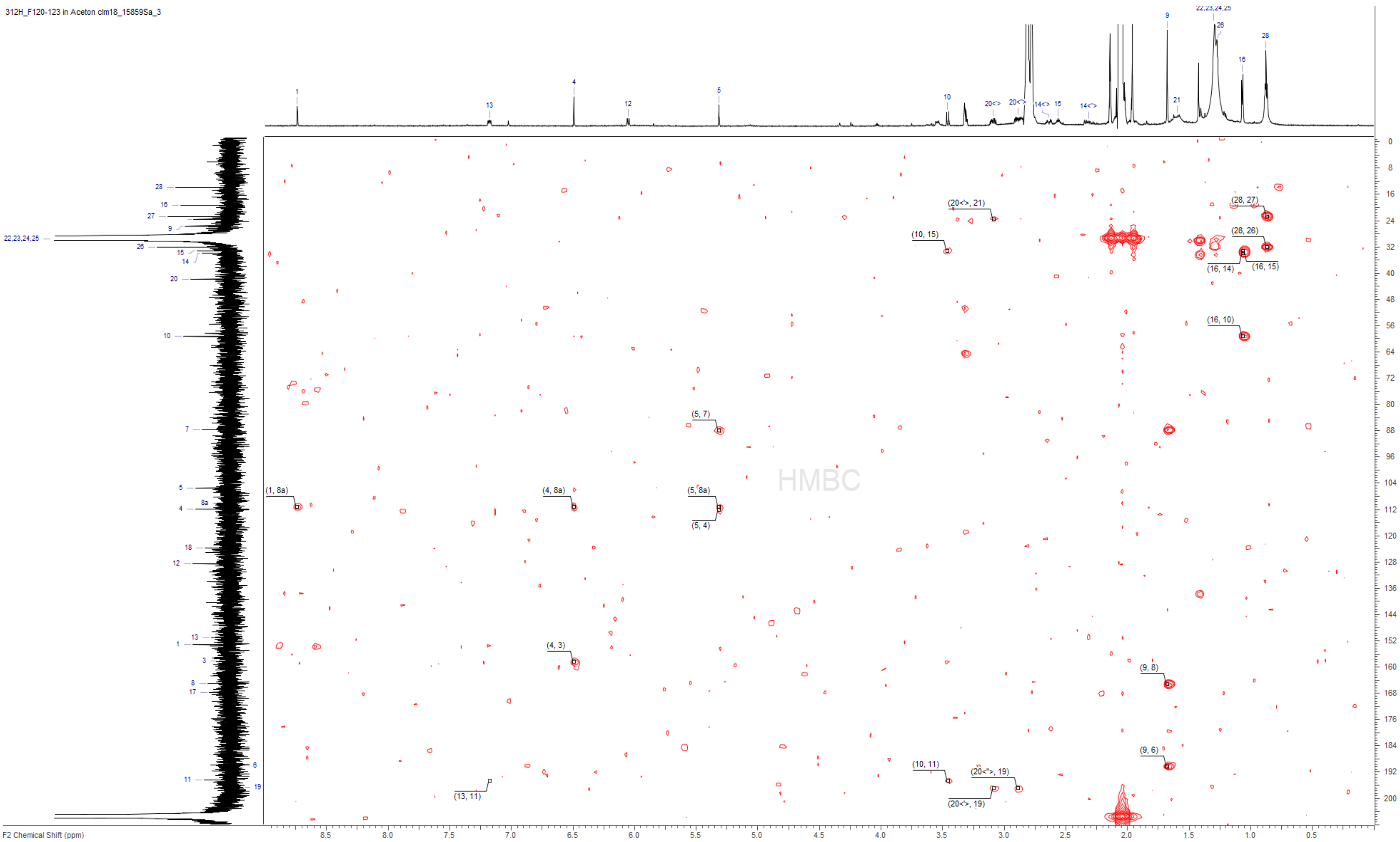

Figure S25: HMBC NMR spectrum (700 MHz, acetone-d_6) of minutellin H (**4**).

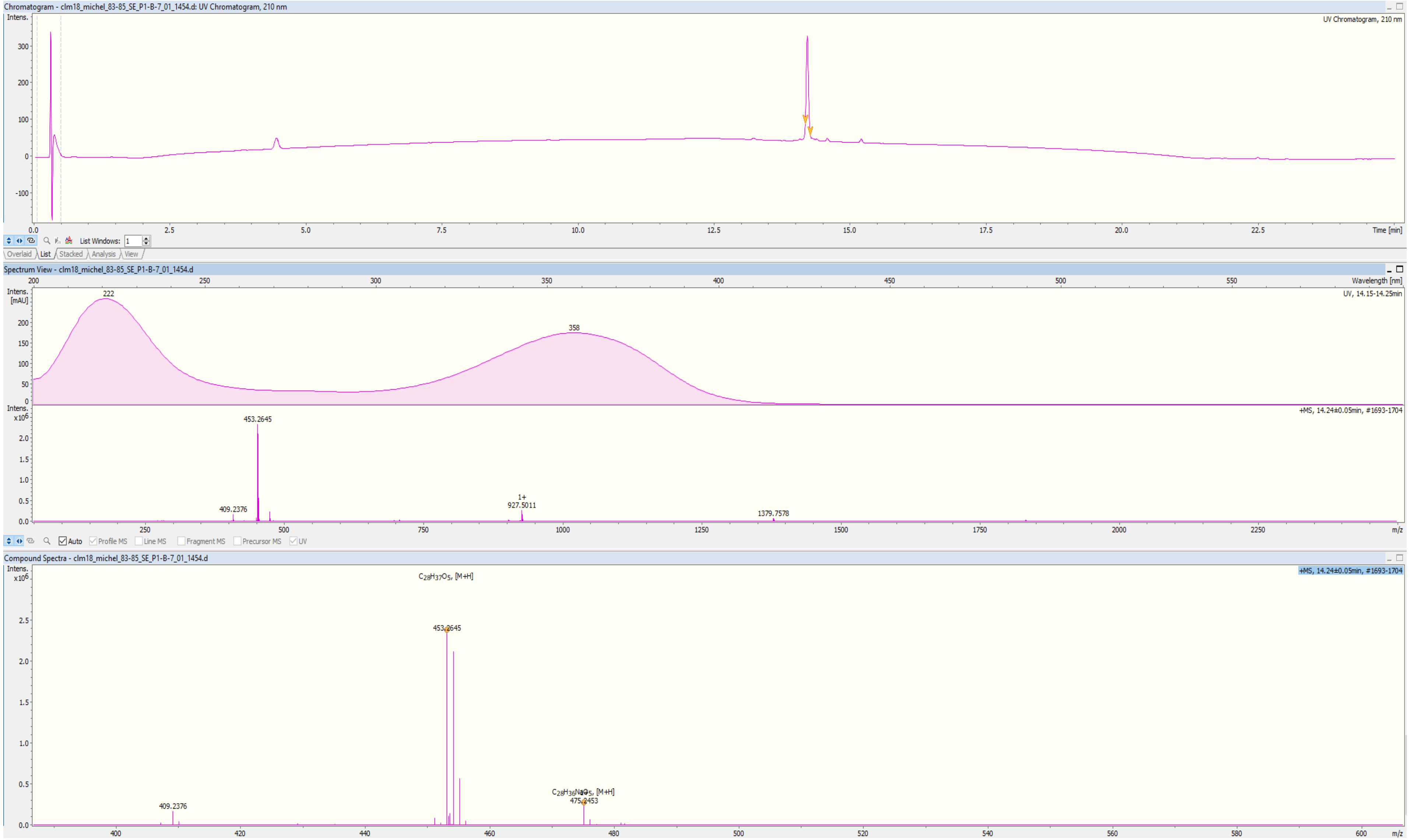

Figure S26: HRESIMS data of minutellin I (**5**).

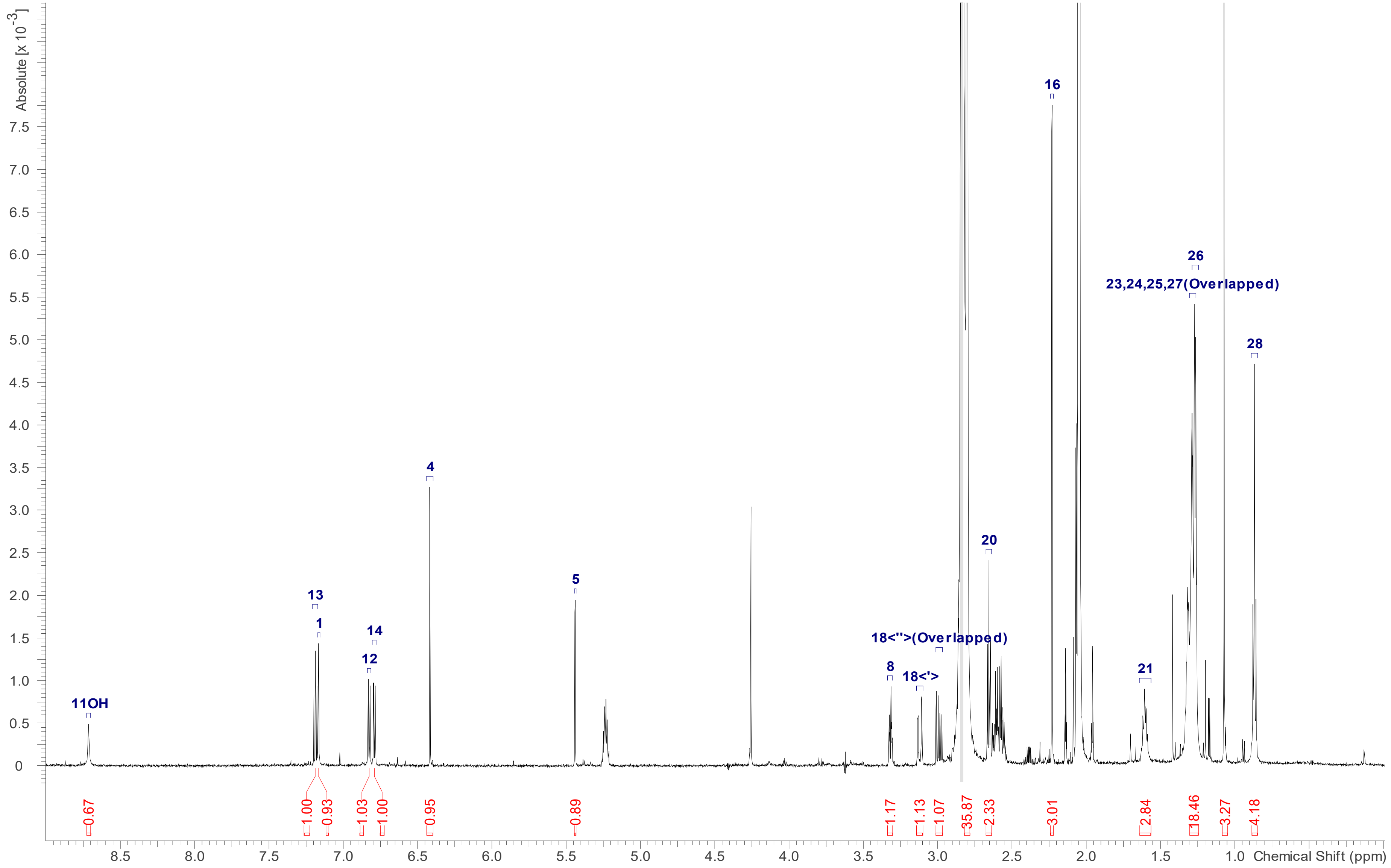

Figure S27: ^{1}H NMR spectrum (700 MHz, acetone-d_6) of minutellin I (**5**).

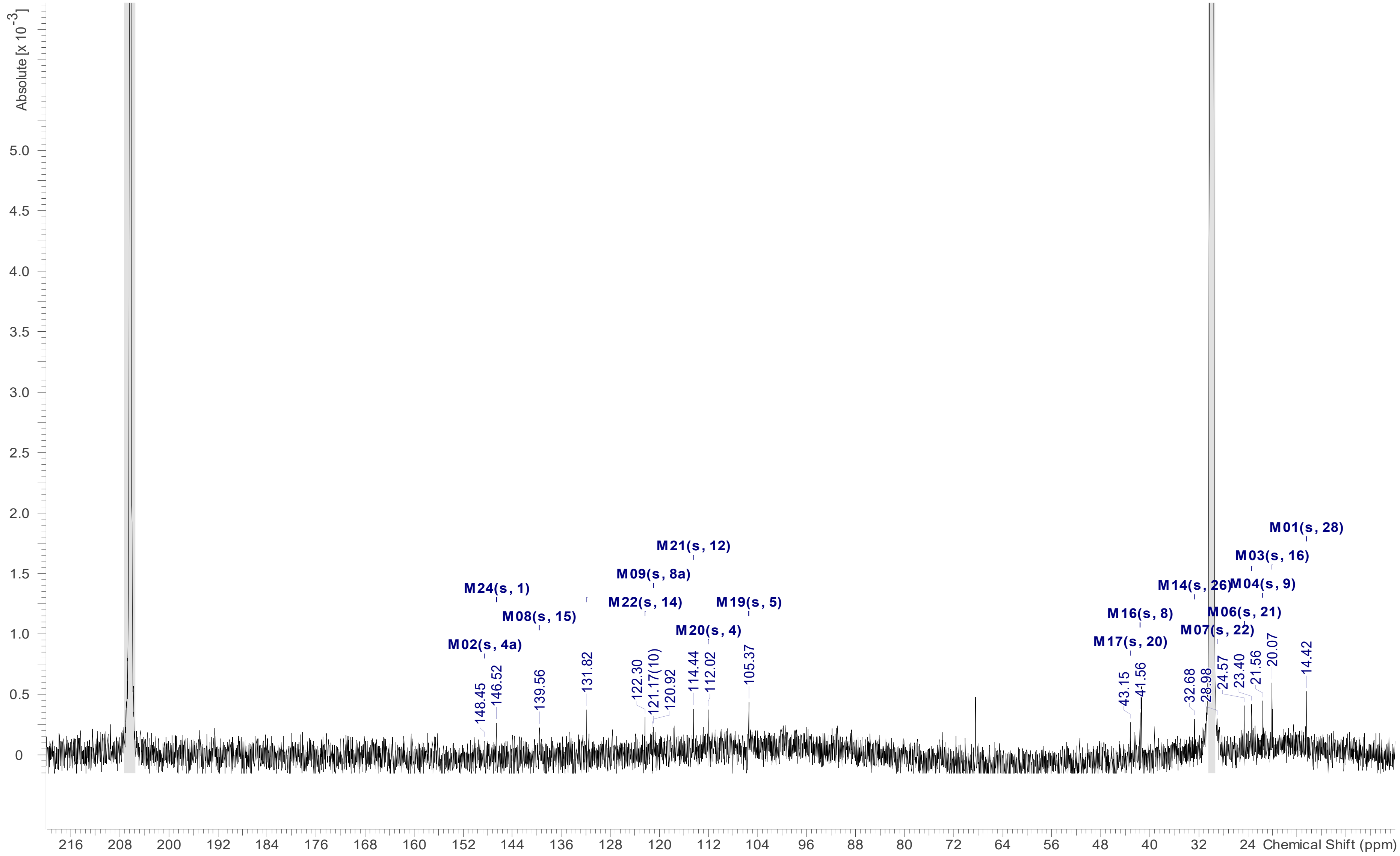

Figure S28: ^{13}C NMR spectrum (175 MHz, acetone-d_6) of minutellin I (**5**).

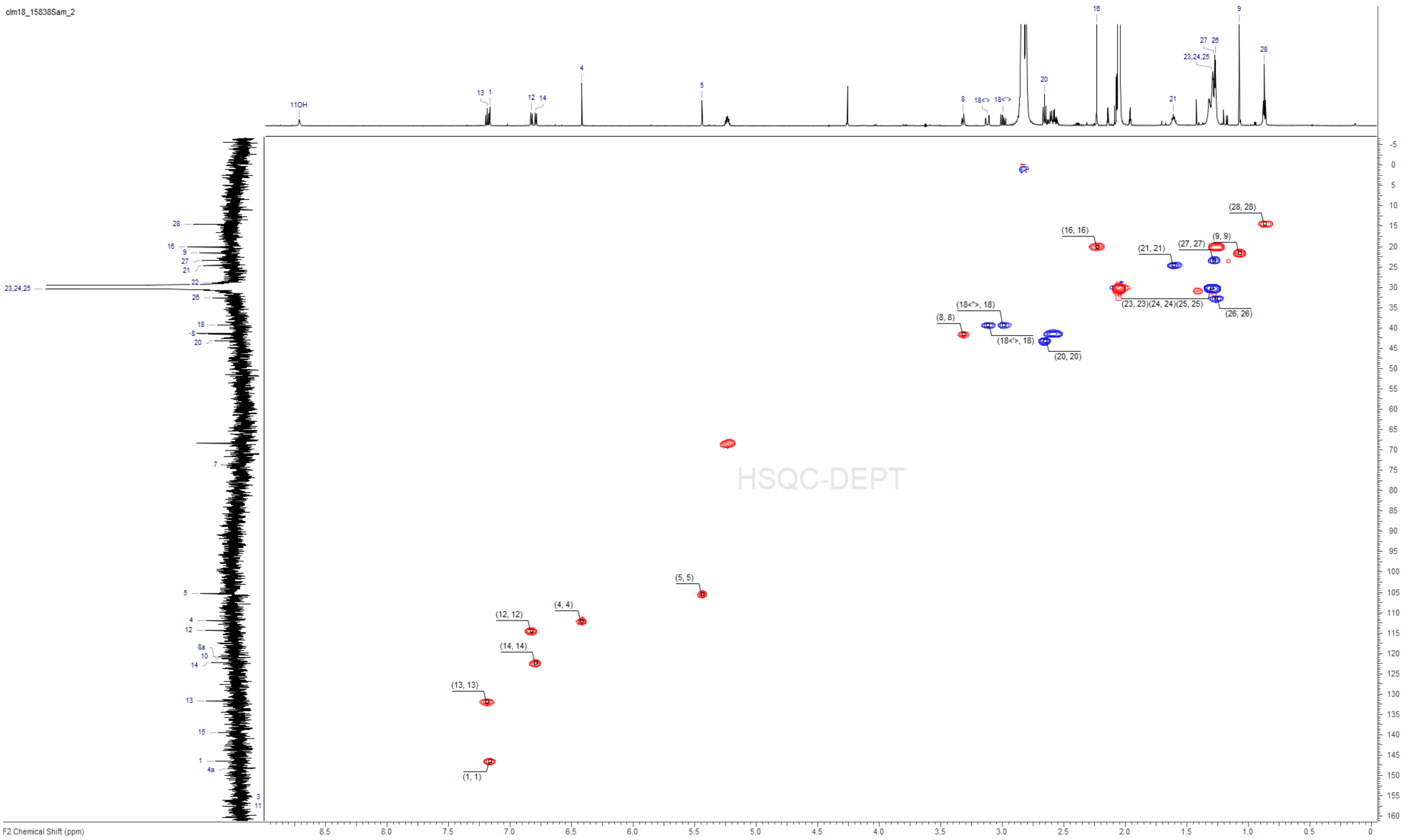

Figure S29: HSQC NMR spectrum (700 MHz, acetone-d_6) of minutellin I (**5**).

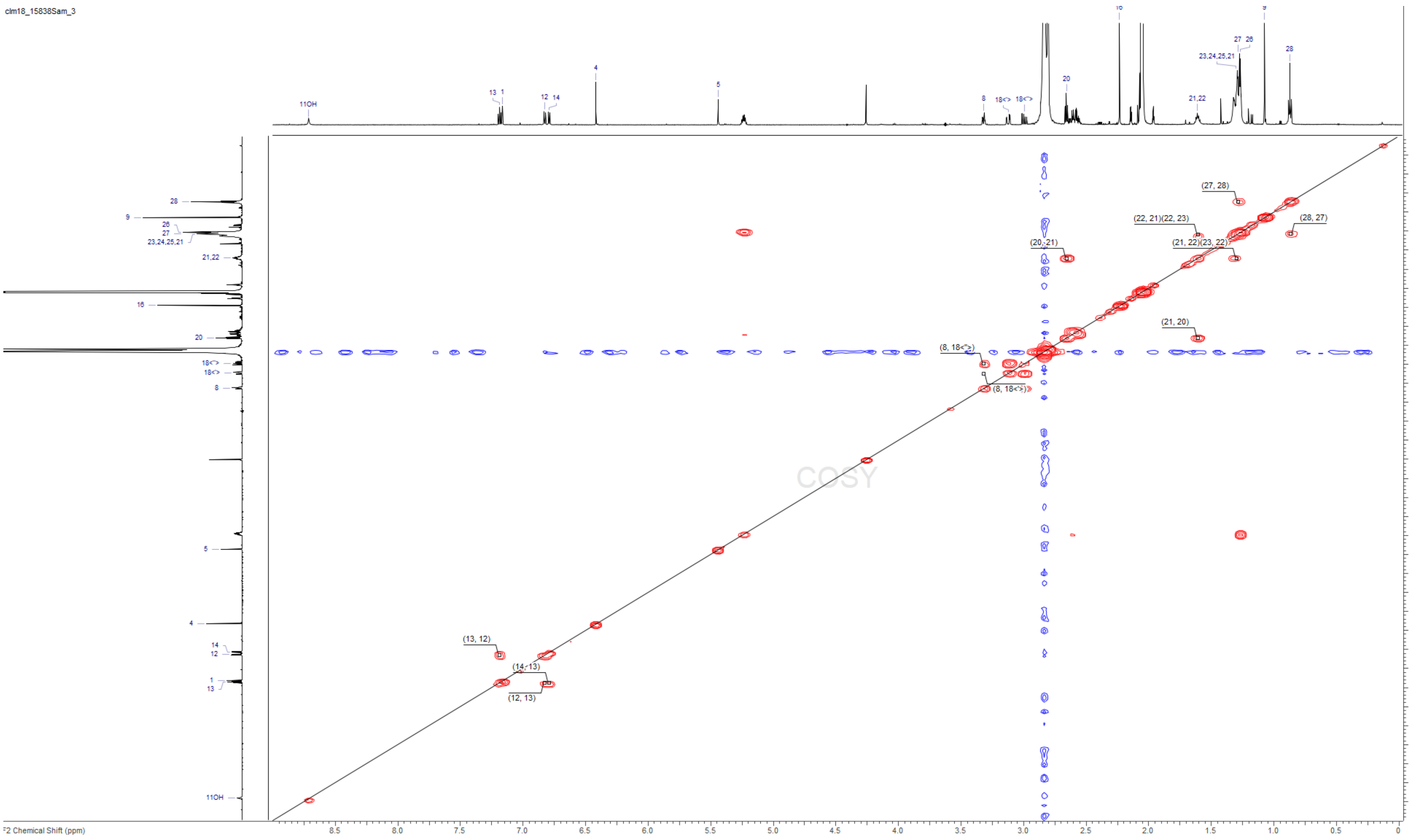

Figure S30: COSY NMR spectrum (700 MHz, acetone-d_6) of minutellin I (**5**).

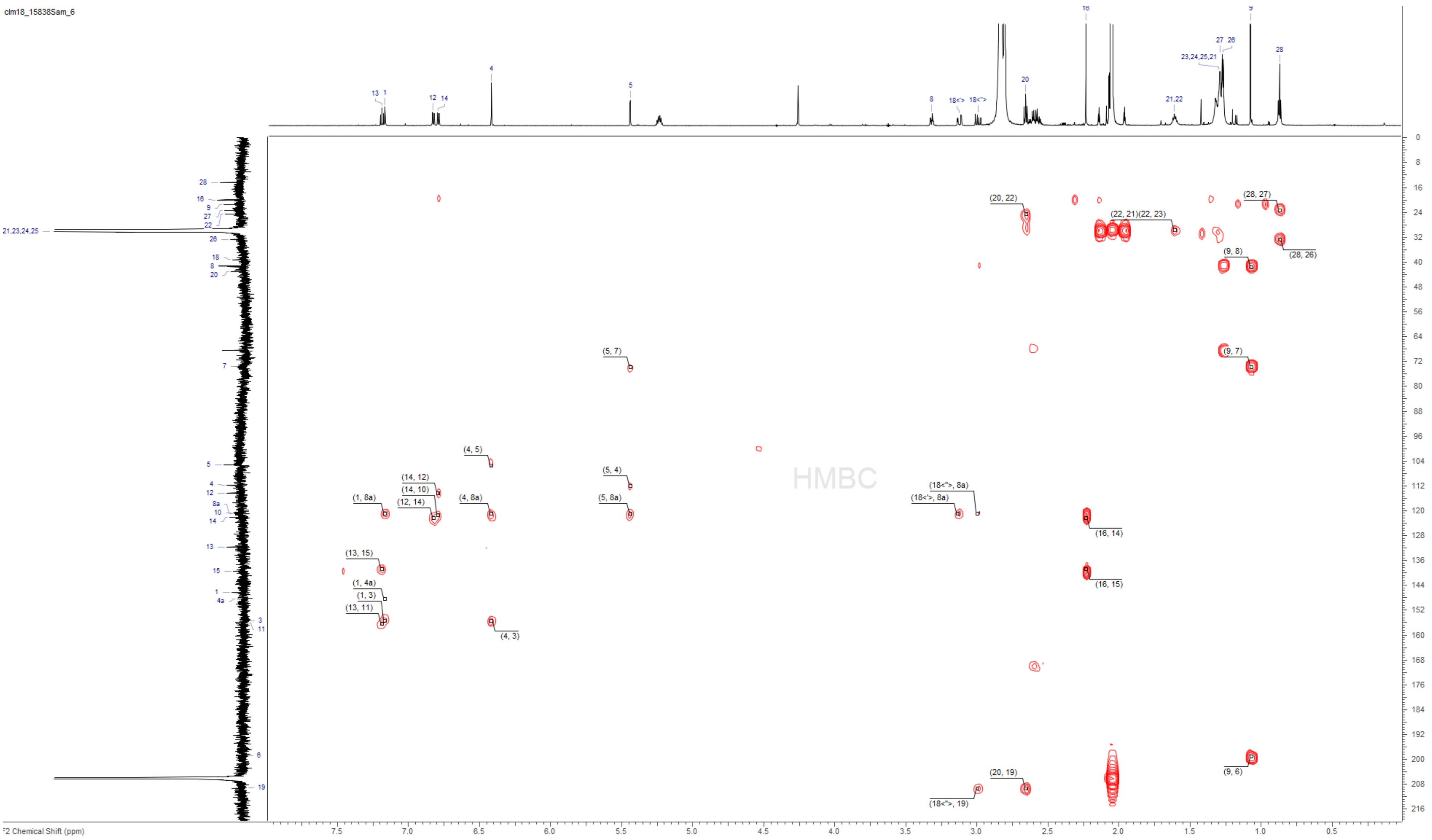

Figure S31: HMBC NMR spectrum (700 MHz, acetone-d_6) of minutellin I (**5**).

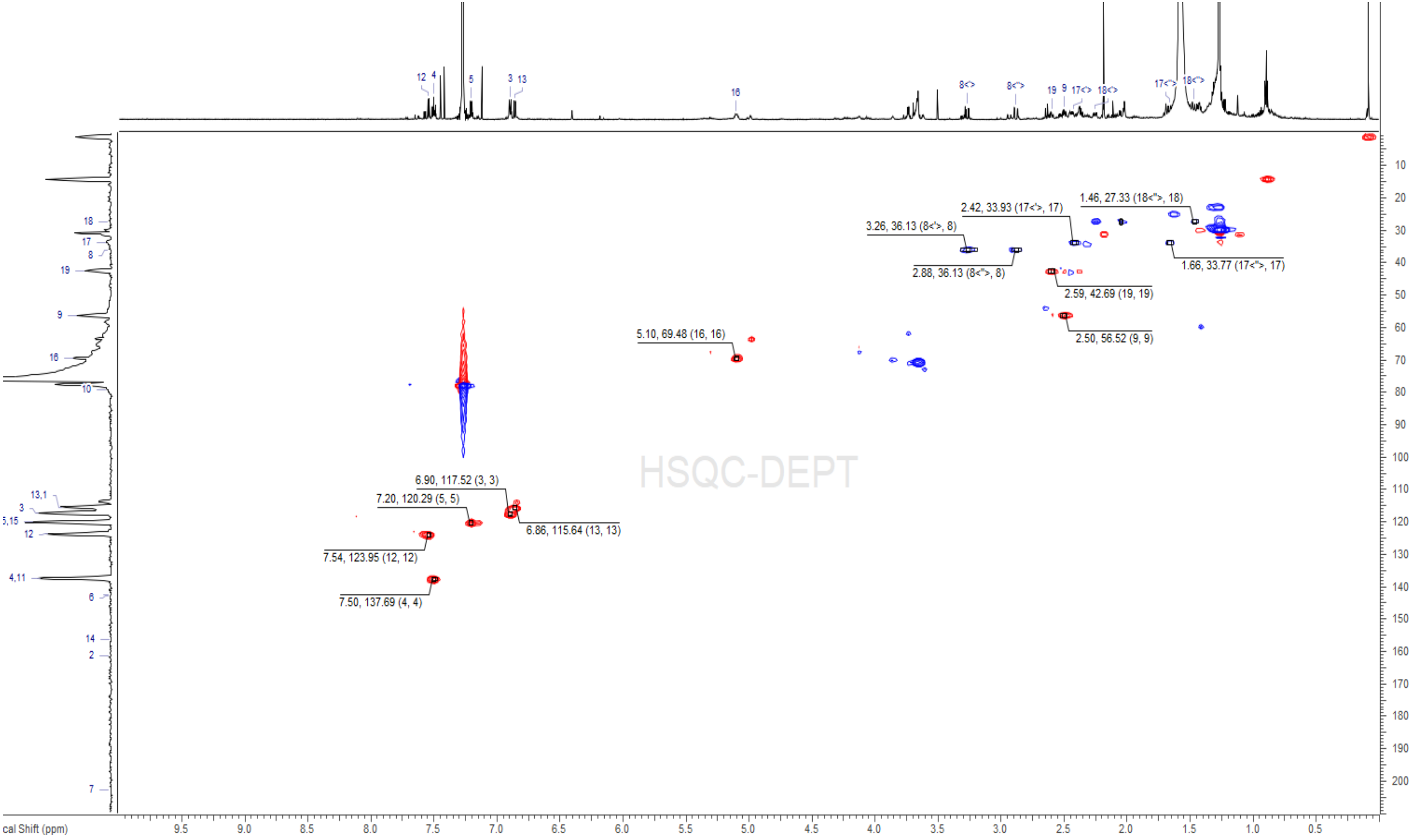

Figure S32: HSQC NMR spectrum (700 MHz, $CHCl_3$-*d*) of daldinon F (**7**).

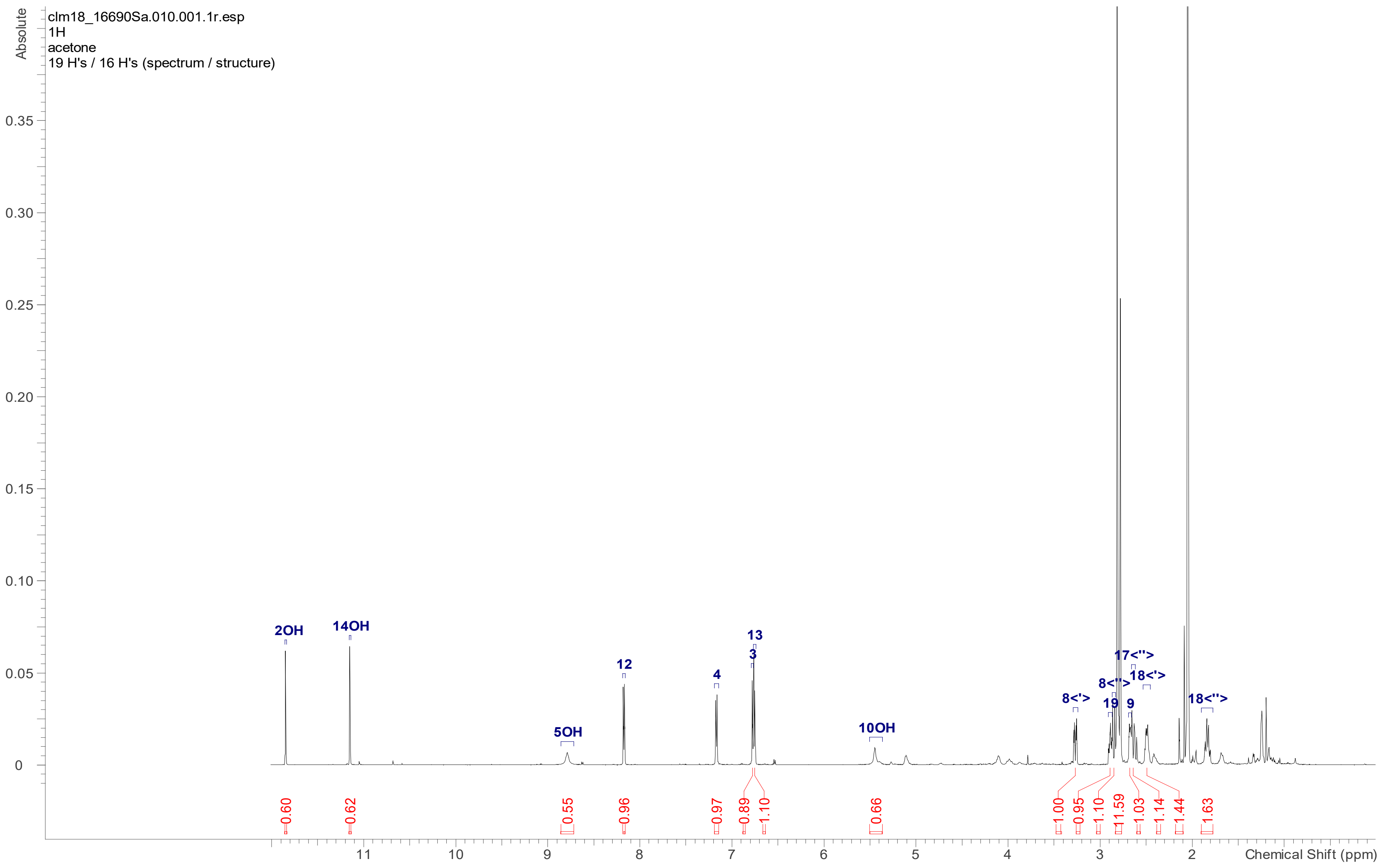

Figure S33: ^{1}H NMR spectrum (700 MHz, aceton-d_6) of daldinon L (**8**).

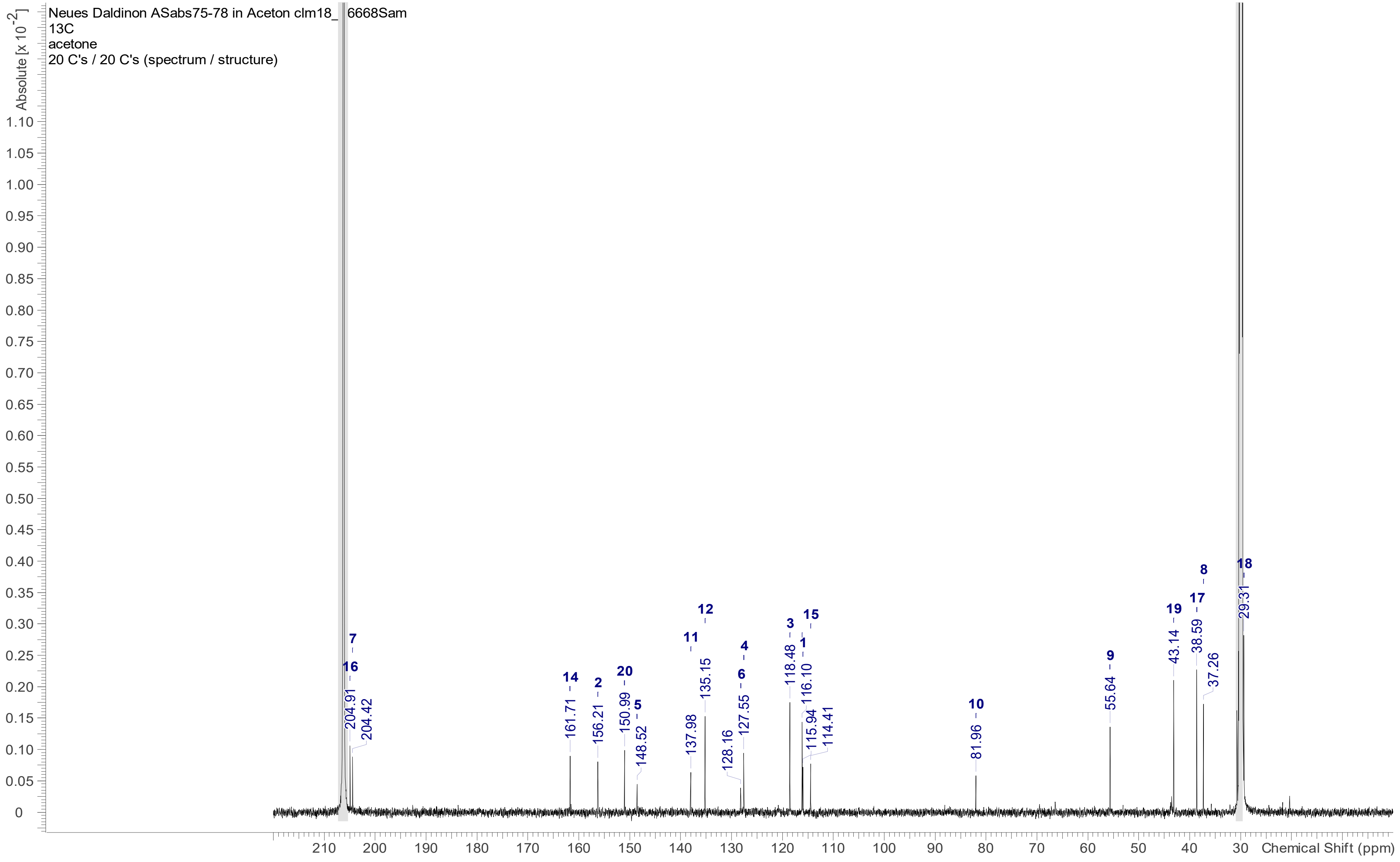

Figure S34: ^{13}C NMR spectrum (175 MHz, aceton-d_6) of daldinon L (**8**).

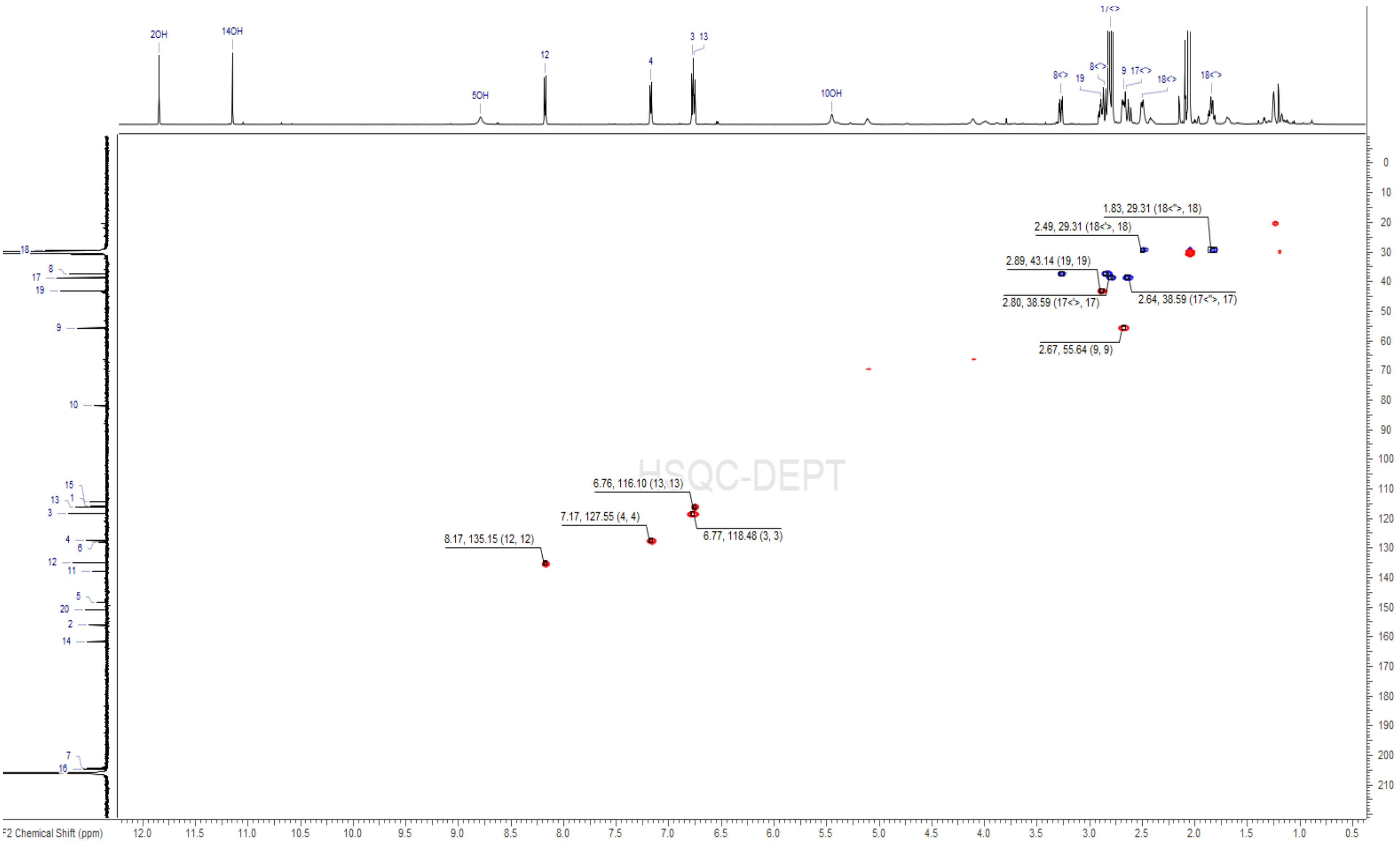

Figure S35: HSQC NMR spectrum (700 MHz, aceton-d_6) of daldinon L (**8**).

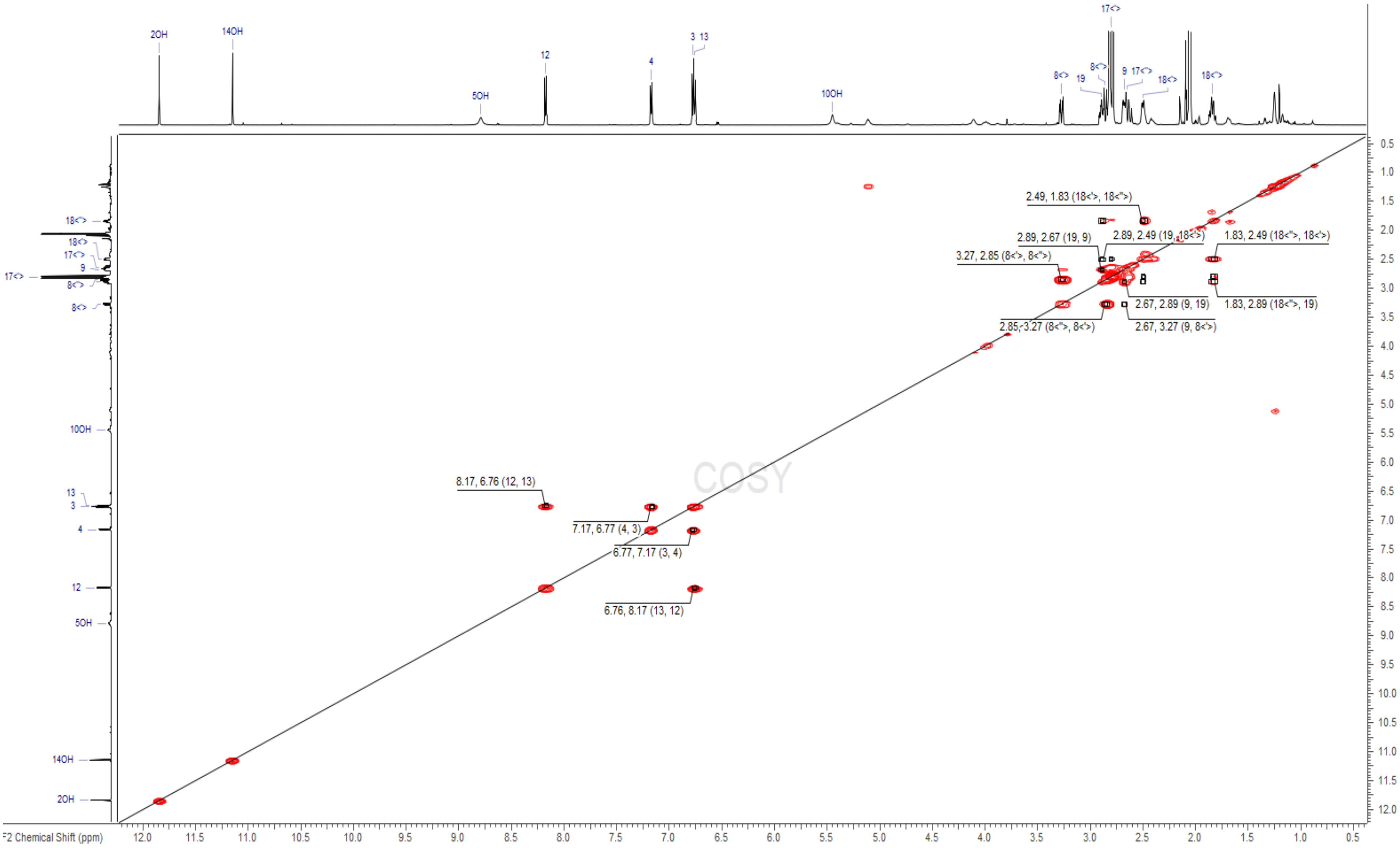

Figure S36: COSY NMR spectrum (700 MHz, aceton-d_6) of daldinon L (**8**).

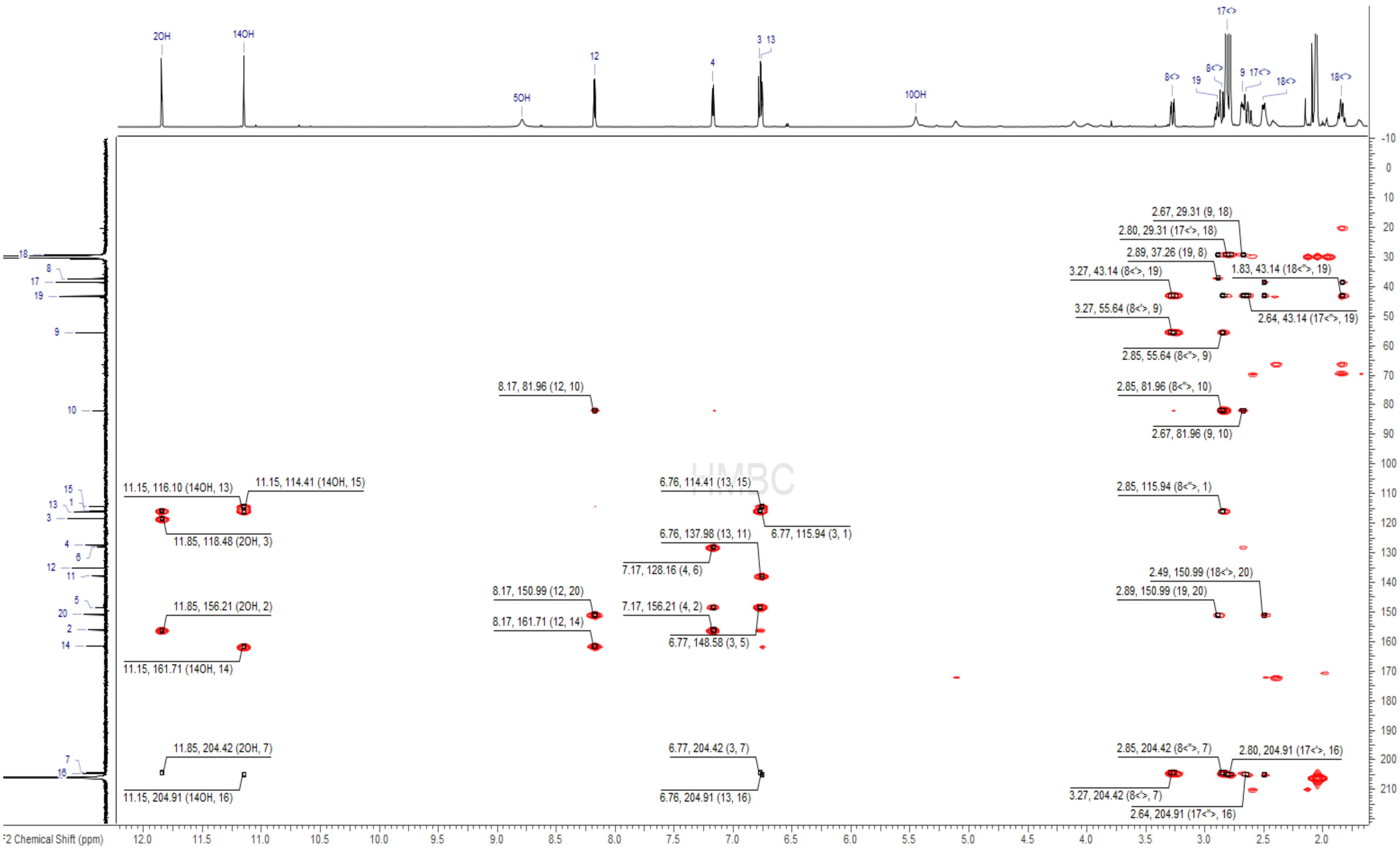

Figure S37: HMBC NMR spectrum (700 MHz, aceton-d_6) of daldinon L (**8**).

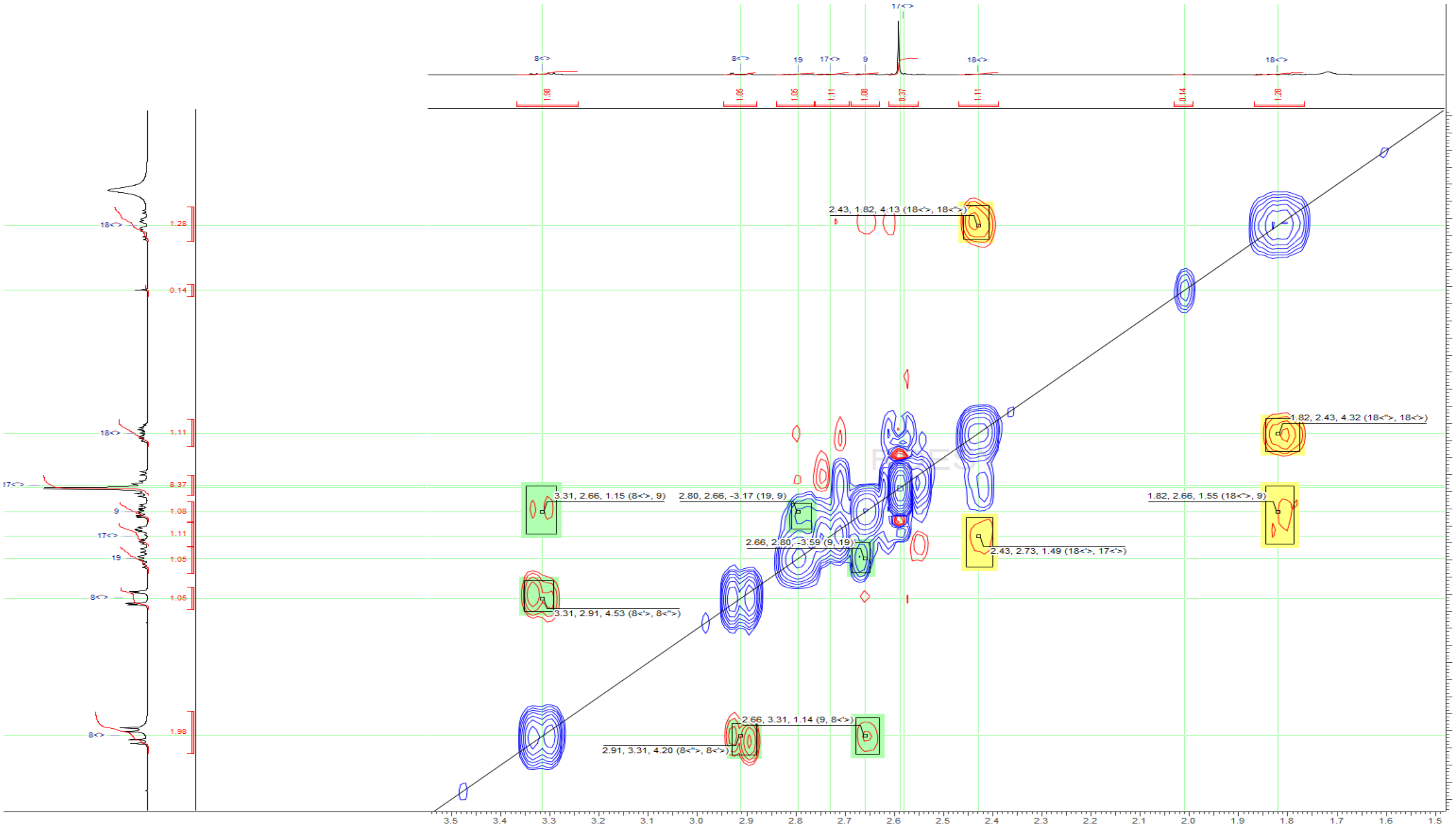

Figure S38: Key section of the ROESY NMR spectrum (500 MHz, chloroform-*d*) of **9**.

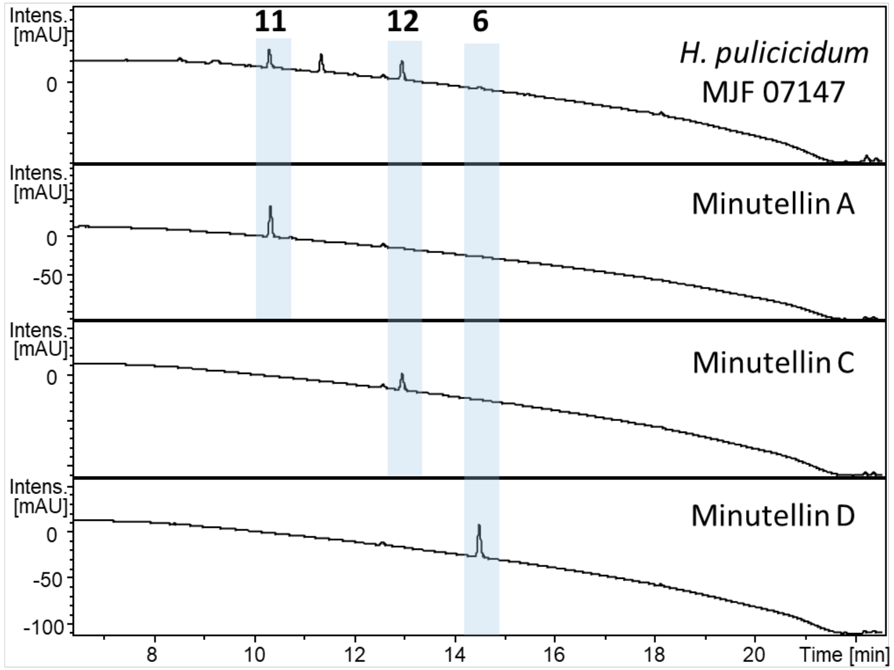

Figure S39: HPLC-UV/Vis chromatogram (210 nm) of an acetone stroma extract of the holotype of *H. pulicicidum* MJF 07147 (top panel) and standards of minutellins A, C and D (below). Numbers correspond to the structure number in the main manuscript.

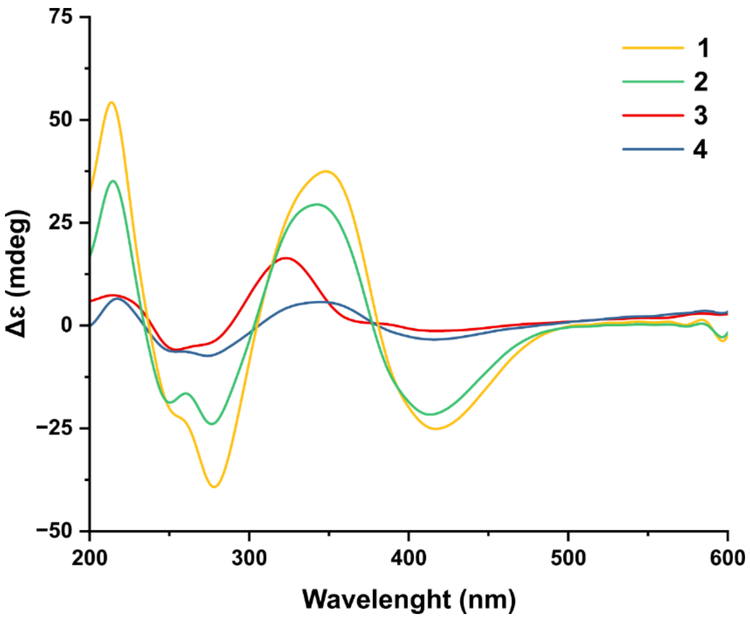

Figure S40: Experimental ECD spectra fort he new minutellins **1**–**4**.
